# *De novo* transcriptome assembly and analysis to identify potential gene targets for RNAi-mediated control of the tomato leafminer (*Tuta absoluta*)

**DOI:** 10.1186/s12864-015-1841-5

**Published:** 2015-08-26

**Authors:** Roberto de A. Camargo, Roberto H. Herai, Luana N. Santos, Flavia M M Bento, Joni E. Lima, Henrique Marques-Souza, Antonio Figueira

**Affiliations:** Centro de Energia Nuclear na Agricultura, Universidade de São Paulo, Av. Centenário, 303, CP 96, Piracicaba, SP 13400-970 Brazil; Escola Superior de Agricultura “Luiz de Queiroz” (ESALQ), Universidade de São Paulo, Av. Pádua Dias, 11, CP 09, Piracicaba, SP 13418-900 Brazil; Department of Pediatrics, Cellular and Molecular Medicine, School of Medicine, University of California San Diego, Torrey Pines Scenic Dr, La Jolla, CA 92093-0695 USA; Graduate Program in Health Science, School of Medicine, Pontifícia Universidade Católica do Paraná, R. Imaculada Conceição, 1155, Prado Velho, Curitiba, PR 80215-901 Brazil; Instituto de Biologia, Departamento de Histologia e Embriologia, Universidade Estadual de Campinas, R. Charles Darwin, CP 6109, Campinas, SP 13083-863 Brazil

**Keywords:** Gelechiidae, Gene silencing, Hormone synthesis, Lepidoptera, RNA-seq, Solanaceae

## Abstract

**Background:**

Providing double-stranded RNA (dsRNA) to insects has been proven to silence target genes, and this approach has emerged as a potential method to control agricultural pests by engineering plants to express insect dsRNAs. A critical step of this technology is the screening of effective target genes essential for insect development and/or survival. The tomato leafminer (*Tuta absoluta* Meyrick) is a major *Solanum lycopersicum* (tomato) pest that causes significant yield losses and has recently invaded Europe, from where it is spreading at an alarming rate. To explore RNA interference (RNAi) against *T. absoluta*, sequence information on potential target genes is necessary, but only a few sequences are available in public databases.

**Results:**

We sequenced six libraries from RNA samples from eggs, adults, and larvae at four stages, obtaining an overall total of around 245 million reads. The assembled *T. absoluta* transcriptome contained 93,477 contigs with an average size of 1,574 bp, 59.8 % of which presented positive Blast hits, with 19,995 (21.4 %) annotated by gene ontology. From the transcriptome, most of the core genes of the RNAi mechanism of Lepidoptera were identified indicating the potential suitability of *T. absoluta* for gene silencing. No contigs displayed significant similarity with a RNA-dependent RNA Polymerase. Genes from the juvenile hormone and ecdysteroid biosynthetic pathways were identified, representing potential target genes for systemic silencing. Comparisons of transcript profiles among stages revealed 1,577 genes differentially expressed at earlier larval stages, from which potential gene targets were identified. Five of these genes were evaluated using *in vitro* transcribed dsRNA absorbed by tomato leaflets, which were fed to 1^st^ instar *T. absoluta* larvae, resulting in significant reduction of larval body weight while exhibiting significant knockdown for three of the genes.

**Conclusions:**

The transcriptome we generated represents a valuable genomic resource for screening potential gene targets that affect the development or survival of *T. absoluta* larvae. Five novel genes that showed greater expression at the 1^st^ larval stage were demonstrated to be effective potential RNAi targets by reducing larval weight and can be considered good candidates for use in RNAi-mediated crop protection.

**Electronic supplementary material:**

The online version of this article (doi:10.1186/s12864-015-1841-5) contains supplementary material, which is available to authorized users.

## Background

Since the discovery that providing double-stranded RNA (dsRNA) to a wide variety of organisms, including insects, can induce RNA interference (RNAi), this method has become a common tool for functional genomic studies, particularly in non-model systems [1, 2]. Early studies used microinjection to deliver dsRNA into insect bodies, but the demonstration that dsRNA uptake through ingestion was sufficient to knock target genes down opened the possibility of applying this approach on a larger scale [2]. The potential application of RNAi for controlling agricultural insect pests soon became evident [3]. Application of dsRNA by crop spraying resembles current insecticide delivery methods, but RNA production costs and stability with current technology may restrict its efficacy [4]. However, the demonstration that transgenic plants modified to express insect dsRNA can successfully control target pests [5, 6] raised the possibility of developing RNAi-mediated crop protection [3, 4, 7]. Transgenic plants expressing dsRNA matching specific insect target genes have been demonstrated to control Lepidoptera, Coleoptera and Hemiptera agricultural pests [4], including *Helicoverpa armigera* in cotton [8] and tobacco [9, 10], *Diabrotica virgifera virgifera* in maize [5], *Nilaparvata lugens* in rice [11], *Myzus persicae* in *Nicotiana benthamiana* and *Arabidopsis thaliana* [12], and *Sitobion avenae* in wheat [13].

A critical requisite of this technology is the availability of a large number of potential target gene sequences to be screened for effectiveness [14]. Given that agricultural insect pests are generally non-model systems, little genomic information is available for most of them. Originally, cDNA libraries were used to screen for insect target genes [5], but the advent of massive RNA sequencing by Next-Generation Sequencing platforms has enabled the reconstruction of almost full transcriptomes under any biological condition for non-model organisms [15]. This approach to identifying potential gene targets for silencing has been successfully used for various agricultural pests, including the eastern corn borer *Ostrinia furnacalis* [16], the aphid *Sitobion avenae* [17], the beet armyworm *Spodoptera exigua* [18], the brown planthopper *N. lugens* [19], and the cotton boll weevil *Anthonomus grandis* [20], to name a few.

In addition, a full transcriptome from insects can be used to reveal genes of the core mechanism of RNAi, which is mostly conserved among Eukaryotes but tends to exhibit peculiarities among taxa. The basic repertoire of genes includes those encoding transmembrane channel proteins (e.g. SID-1) involved in dsRNA uptake and systemic spread, nucleases such as Dicer and Dicer-like that cleave dsRNAs into small interfering RNAs, and those belonging to the RNA-induced silencing complex (or RISC), including the Argonaute protein family, that recognizes dsRNA and degrades target mRNA, and other factors involved in RISC assembly, such as *R2D2*, and putative RNA helicases such as *Armitage* and *SpindleE* [1, 21]. A critical factor for efficient RNAi in insects is the presence of effective proteins involved in dsRNA uptake and systemic spread, as the members of Insecta apparently lacks the canonical RNA-dependent RNA polymerase (RdRP) activity required for amplification of the siRNA signal [7]. Lepidopteran species, together with other more derived insects such as the dipterans, are believed to be more refractory to systemic RNAi [22], and variable sensitivity to systemic RNAi has been reported among the Lepidoptera [7, 22, 23].

The transcriptional analysis of insects can also shed light on the molecular basis of development. In insects, the control of development, molting and other processes is largely regulated by the balance between the acyclic sesquiterpenoid juvenile hormone (JH), produced at the *corpus allatum*, and ecdysteroids synthesized at the prothoracic glands [24]. Synthesis of JHs derives from the mevalonate pathway by the combination of isopentyl pyrophosphate (IPP) and dimethylallyl pyrophosphate (DMAP) to form farnesyl pyrophosphate, which is dephosphorylated to farnesol, oxidized to farnesoic acid, methylated to methyl farnesoate, and converted by epoxidation to JH [24]. Degradation of JH mainly occurs by the action of juvenile hormone esterase (JHE) and/or juvenile hormone epoxide hydrolase (JHEH), both of which lead to a decrease in signaling [24]. In the case of ecdysteroids, since arthropods lack the squalene synthase enzyme, synthesis of ecdysteroids depends on an exogenous sterol source derived from the diet, which usually requires dealkylation and a series of hydroxylation steps [24], catalyzed by cytochrome P450 enzymes encoded by the Halloween genes (*phantom*, *disembodied*, *shadow* and *shade*) [25].

*Tuta absoluta* Meyrick (Lepidoptera: Gelechiidae), known as the South American tomato leafminer or pinworm, is a major Solanaceae pest, particularly of tomato (*Solanum lycopersicum*). This multivoltine microlepidoptera may attack all stages of the host plant, but female adults lay eggs preferentially in leaves, where emergent larvae penetrate and feed, forming galleries in the leaf mesophyll and leading to severe damage of photosynthetic tissues. Major losses also derive from the attack of fruits, aggravated by secondary infection with opportunistic pathogens [26]. This Neotropical species, once restricted to America, invaded Europe and Northern Africa in 2006/2007, from where it is spreading at an alarming rate, threatening major tomato exporters such as India, China, the United States and Mexico [27]. Furthermore, insecticide resistance has been reported in *T. absoluta*, making the development of alternative means for control even more urgent [28].

In this study, large-scale RNA sequencing (RNA-seq) was conducted to establish a transcriptome profile of six developmental stages of *T. absoluta,* enabling the identification of potential gene targets for knockdown using the RNAi approach. In addition, the core genes of the basic RNAi mechanism were investigated to determine the suitability of gene silencing in *T. absoluta*. To validate the coverage of the transcriptome, we searched for genes from insect hormone biosynthetic pathways as potential targets for systemic silencing. Furthermore, comparisons of transcript profiles among stages revealed differentially expressed transcripts (DET) at earlier larval instar stages, which allowed identification of potential gene targets. Five such genes were evaluated using *in vitro* transcribed dsRNA absorbed by tomato leaflets, fed to 1^st^ instar *T. absoluta* larvae, resulting in significant reduction of larval body weight while exhibiting knockdown of the genes.

## Results

### Transcriptome sequencing and assembling

Six libraries were generated from *T. absoluta* samples from eggs, adults, and larvae putatively from the 1^st^, 2^nd^, 3^rd^, and 4^th^ larval stages. An overall total of *ca*. 245 million reads was obtained, ranging from *ca*. 34 to 55 million per library, with an average 44 % GC content (Table 1). In general, all libraries presented good quality, with an average of 94.2 % of reads with base call quality at 99 % probability (Q20) and 87 % at 99.9 % (Q30) (Table 1). The sequences were filtered for adaptors and sequencing artifacts, reducing the number of reads per library by 5 to 15 % (filtered reads; Table 1), before transcriptome assembly. The absence of a reference genome for *T. absoluta*, together with the high coverage in sequenced RNA libraries, led us to the use of a *de novo* transcriptome assembly to generate a reference for subsequent analyses. The high quality reads were *in silico* normalized to reduce sequencing coverage. The normalized data were assembled with a minimum fragment overlap of 35 bp and only contigs longer than 300 bp were included in the assembly. The assembled *T. absoluta* transcriptome contained 93,477 contigs. The assembled reference transcriptome contained 147,141,189 nucleotides in contigs, with an average size of 1,574 bp, an N50 of 2,427 bp and an N95 of 480 bp. The data were deposited under a NCBI Bioproject [GenBank:PRJNA291932] and under a Sequence Read Archives accession [GenBank:SRS794929].

**Table 1:** Sequenced libraries for each stage of development with total number of generated bases, total of reads per library, percent G + C, percent of reads with quality above Q20 or above Q30

Libraries	Total bases	(G + C)%	Q20 (%)	Q30 (%)	Total reads	Filtered reads	Assembled
Eggs	5,626,900,284	43.53	94.4	87.4	55,711,884	52,617,684	19,261,127
1^st^ Stage	3,718,827,070	41.23	94.8	88.0	36,820,070	28,657,483	12,957,321
2^nd^ Stage	3,497,118,738	43.91	94.2	87.0	34,624,938	32,621,354	12,777,709
3^rd^ Stage	4,469,182,734	45.21	93.9	86.4	44,249,334	41,546,244	13,442,005
4^th^ Stage	3,759,123,848	45.56	94.1	86.6	37,219,048	35,045,786	9,689,430
Adults	3,668,050,734	44.01	94.0	86.5	36,317,334	34,105,903	14,378,063
Total	24,739,203,408				244,942,608		82,505,655
Mean		44.0	94.2	87.0			

### Transcriptome annotation

**Fig. 1:**
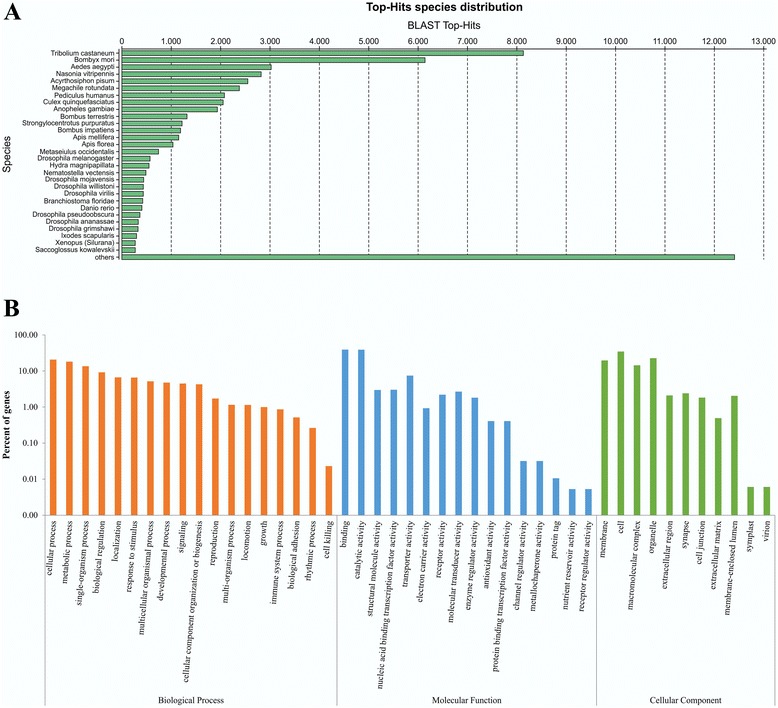
Most represented species in Top Blastx-matches and gene ontology classification of the *Tuta absoluta* transcriptome. **a**. The analysis of the 93,477 contigs was conducted by Blast2Go [29] using Blastx at an *E*-value cutoff of e^−3^. Top-hits included insect species with complete genome sequences, including Coleopterae (*T. castaneum*), Lepitodptera (*B. mori*), Diptera (*A. aegypti*, C. *quinquefasciatus*), Hymenoptera (*N. vitripennis*, *M. rotundata*), Hemiptera (*A. pisum*), and Phthiraptera (*P. humanus*). **b**. The analysis of the 93,477 contigs was conducted by Blast2Go [29], using Blastx to recover annotations with significant homology from the NCBI. All terms ‘Biological Processes’, ‘Molecular Function’ and ‘Cellular Component’ at level 2 are represented as percent over total number of sequences, in decreasing order

The assembled *T. absoluta* transcriptome was analyzed for gene ontology by Blastx searches against the NCBI non-redundant protein database (nr) using Blast2Go [29]. From the 93,477 contigs, *ca.* 55,900 (59.8 %) presented positive Blast hits, with 19,995 (21.4 %) successfully annotated by gene ontology. The predominant positive Blast hits identified were from insects, particularly *Tribolium castaneum*, *Bombyx mori*, *Aedes aegypti*, *Nasonia vitripennis*, *Acyrthosiphon pisum*, *Megachile rotundata*, *Pediculus humanus*, and *Culex quinquefasciatus* (Fig. 1a). In this pair-wise comparison, our dataset showed the highest level of similarity to the *Tribolium* genome, with over 8,000 hits. Given that the Lepidoptera *B. mori* was the second most common top-hit species, with over 6,000 hits, we consider this effect to be more a consequence of the completeness of the *Tribolium* genome [30] rather than representative of biological genomic conservation.

Annotation of the *T. absoluta* transcriptome by Blast2Go revealed the main GO categories under the ‘Biological Processes’, ‘Molecular Function’ and ‘Cellular Component’ ontologies (Fig. 1b). The most enriched terms for ‘Biological Process’ were cellular (20.6 % of total number of sequences), metabolic (18.1 %) and single organism processes (13.5 %), while the dominant categories for ‘Molecular Function’ were binding (39.2 %), catalytic (38.8 %) and transporter activity (7.4 %) (Fig. 1b). Under ‘Cellular Component’, cell comprised 34.5 % of the sequences, organelle 22.7 % and membrane 19.6 % (Fig. 1b).

### Differential expression during development

**Fig. 2:**
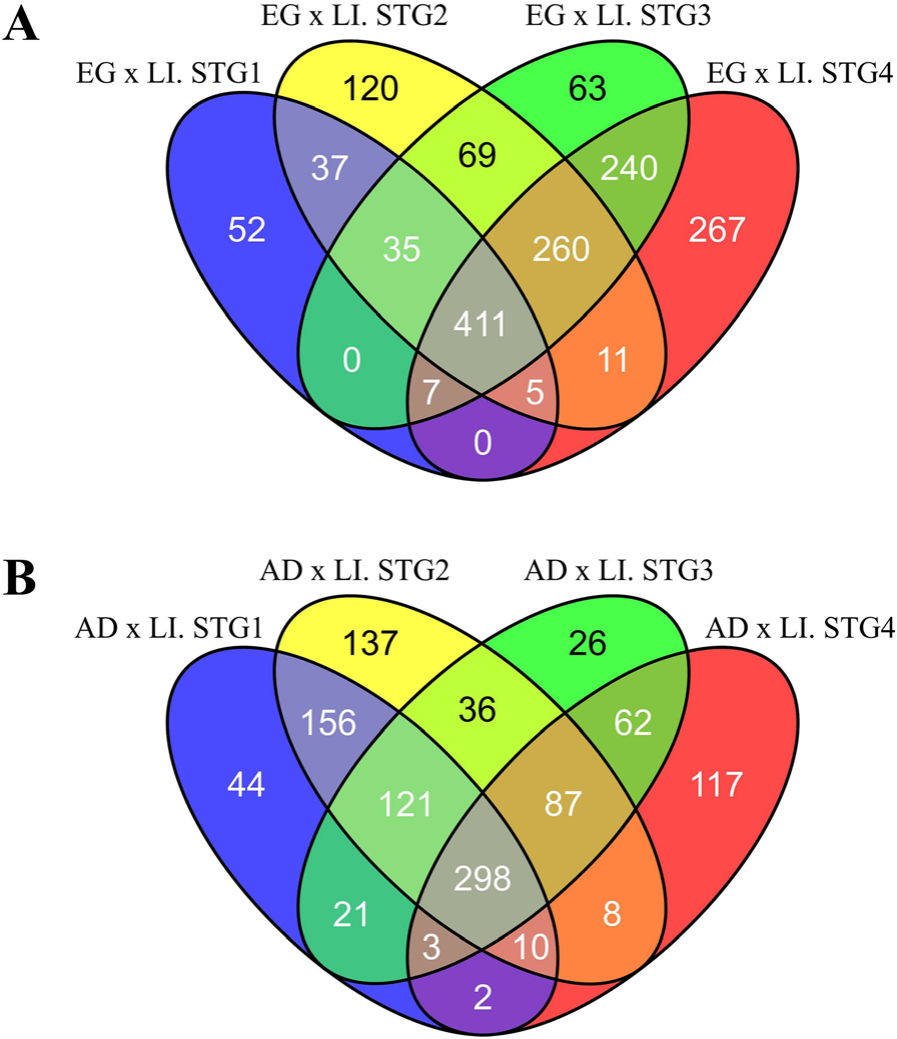
Venn diagrams representing number of differentially expressed genes among *Tuta absoluta* developmental stages. **a**. Number of genes differentially expressed between the developmental stages egg (EG) and larvae stage 1 (LI.STG1), stage 2 (LI.STG2), stage 3 (LI.STG3) and stage 4 (LI.STG4), commonly shared or not among stages; and **b**. Number of genes differentially expressed between adults (AD) and larvae stage 1 (LI.STG1), stage 2 (LI.STG2), stage 3 (LI.STG3) and stage 4 (LI.STG4), commonly shared or not among stages

Differential expression between developmental stages of *T. absoluta* was detected by screening genes with >2-fold variation with statistical significance*.* In total, the analysis found 3,917 significant DET during development, with 1,577 DET between eggs and larval stages, 1,128 between larval stages and adults and 1,212 between eggs and adults. No significant difference in expression was detected between the larval stages. Venn diagrams of the DET among developmental stages allowed the identification of transcripts exclusive or common to the various stages of *T. absoluta* (Fig. 2). The first Venn diagram comprising DET between eggs and all analyzed larval stages identified 547, 948, 1,087 and 1,201 DET between eggs and the 1^st^, 2^nd^, 3^rd^ and 4^th^ stages, respectively (Fig. 2a). Comparing larval stages with eggs, we identified 411 DET shared among all four stages analyzed (Fig. 2a). Of the 547 DET between eggs and 1^st^ stage larvae, 52 were exclusive, whereas 37 DET were shared between 1^st^ and 2^nd^ stage larvae, and the remaining DET were shared among the three larval stages. Of the 948 DET between eggs and 2^nd^ stage larvae, 120 were exclusive, 69 were shared between the 2^nd^ and 3^rd^ stages, and 11 were shared between the 2^nd^ and 4^th^ stages. Moreover, 63 DET were exclusive to the 3^rd^ stage in comparison with eggs, while 267 were exclusive to the 4^th^ stage (Fig. 2a).

A Venn diagram of DET between adults and larval stages showed that there were 655, 853, 654 and 587 DET between adults and the 1^st^, 2^nd^, 3^rd^ and 4^th^ stages, respectively (Fig. 2b). In this case, 298 DET were commonly found between adults and all the larval stages (Fig. 2b). Of the 655 DET between adults and the 1^st^ stage, 44 were exclusive, 156 were differentially expressed between adults and the 1^st^ and 2^nd^ stage larvae, 21 were shared between 1^st^ and 3^rd^ stage larvae; and two were shared between the 1^st^ and 4^th^ stages (Fig. 2b). Of the 853 DET between adults and 2^nd^ stage larvae, 137 were exclusive, and 36 were also differentially expressed between adults and 3^rd^ stage larvae. Of the 654 DET between eggs and 3^rd^ larval stage, 26 were exclusive (Fig. 2b). Among the 587 DET between adults and 4^th^ larval stage, 117 were exclusive to this stage (Fig. 2b).

**Fig. 3:**
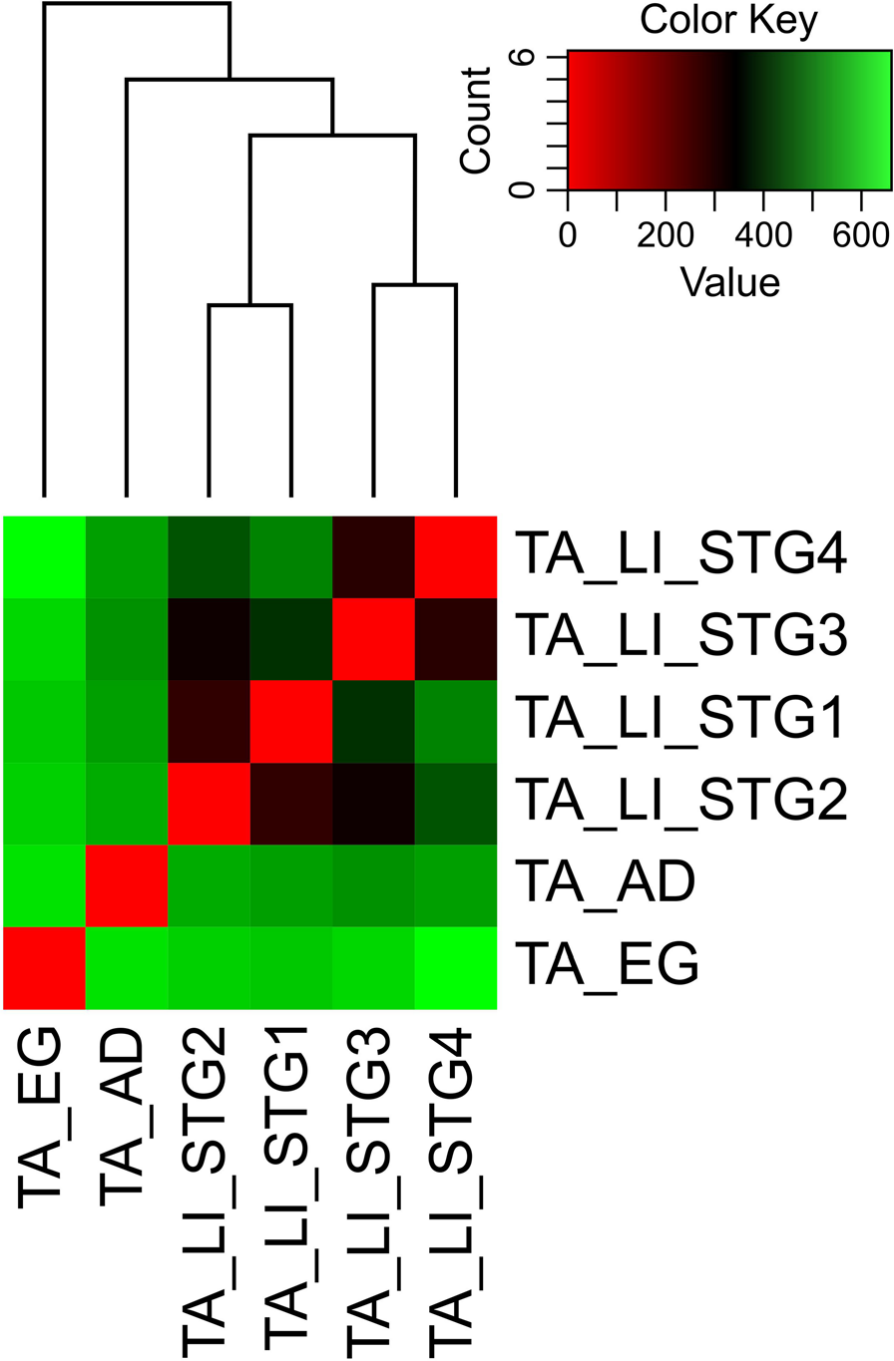
Heat map and clustering representing most similar transcriptome profiles among *Tuta absoluta* developmental stages. The count matrix for all sequenced samples was used to calculate an Euclidian distance matrix, which was used for hierarchical sample clustering, according to the most similar transcriptome profile using the single linkage method, to generate a dendrogram and a heatmap correlating all sample expression profiles into colors, ranging from red (identical profiles) to green (most different). The developmental stages analyzed were egg (TA_EG); 1^st^ stage larvae (TA_LI_STG1); 2^nd^ stage larvae (TA_LI_LSTG2); 3^rd^ stage larvae (TA_LI_LSTG3); 4^th^ stage larvae (TA_LI_STG4); and adults (TA_AD)

The normalized gene expression values from all the libraries were then used to estimate a Euclidian distance matrix based on transcript profiles to generate a dendrogram and a heatmap describing the similarities among the developmental stages of *T. absoluta* (Fig. 3). The heatmap and the dendrogram clearly indicated a similarity gradient between the developmental stages, with the transcript profile from 1^st^ stage larvae more similar to that of 2^nd^ stage larvae, and from the 3^rd^ stage more similar to the 4^th^. The gene expression profile from eggs was the most distinct from all the other stages, particularly from adults. There was less similarity among larvae and eggs than for larvae and adults. Among the larval stages, the closer the stages, the higher the similarity of transcripts profiles (Fig. 3).

Annotation of the DET between the developmental stages by Blast2Go revealed that the most enriched terms for the ‘Biological Process’ ontology were related to metabolic process and single-organism process (Additional file 1: Table S1). When comparing larval stages with the adults, the absolute number of ‘Biological Process’ terms decreased with development, while the opposite was observed for the larval stages when compared to the egg (Additional file 1: Table S1). A progressive decrease in genes involved with response to stimulus was detected over developing larvae in comparison to adults. In the case of ‘Molecular Function’, the two most frequent functions among the DET were catalytic activity and binding for all stage comparisons, while transporter activity was more prominent in comparisons between the egg and the larval stages (Additional file 2: Table S2). Under ‘Cellular Component’ ontology, cell, organelle, macromolecule complex and membrane were the most frequent compartments found for DET genes between eggs and larval stages, but they were less represented (in absolute numbers), or even absent, when larval stages were compared to adults (Additional file 3: Table S3).

The DET were also subjected to a KEGG-based metabolic pathway analysis using Blast2GO. Comparing eggs with the four larval stages, there was an increase in the number of biological pathways related to the DET, from two pathways in the egg *versus* 1^st^ stage larvae comparison to up to 17 pathways in the egg *versus* 4^th^ stage (Additional file 4: Table S4). When these larval stages were compared to adults, we observed a relative decrease in number of pathways from the earlier (8 or 7 pathways) to the later stages (3 or 4 pathways) (Additional file 4: Table S4). In the comparison between eggs and the four larval stages, the steroid hormone biosynthesis and tryptophan metabolism pathways appeared to be altered in the four larvae stages (Additional file 4: Table S4). When we performed a similar analysis by contrasting adults with larval stages, the starch and sucrose metabolism, steroid hormone biosynthesis, and galactose metabolism pathways were altered in all larval stages (Additional file 4: Table S4). The steroid hormone biosynthesis pathway was the only one altered in all four larvae instars compared to both adults and eggs.

### Validation of differently expressed transcripts (DET) during development using RT-qPCR

From the 411 DET between eggs and common to all larval stages (Fig. 2), the 37 DET that were differentially expressed between eggs and the 1^st^ and 2^nd^ larval stages only, and the 298 DET between adults and common to all larval stages, we chose 23 DET to validate the RNA-seq data: 15 among the 411 DET (contigs 10806, 11301, 12524, 12828, 13135, 16411, 16428, 17745, 20172, 21584, 23824, 38086, 50455, 55173, and 6681), five among the 37 (contigs 2406, 36279, 58512, 75835, and 81147), and three among the 298 DET (contigs 26572, 36206, and 77615) between adults and common to all larval stages. Differences in gene expression between developmental stages were estimated by RT-qPCR using gene-specific primers (Additional file 5: Table S5), with three biological replicates, normalized to three gene references (*Rpl5*, *Rpl23A* and *rRNA*; Additional file 5: Table S5).

**Fig. 4:**
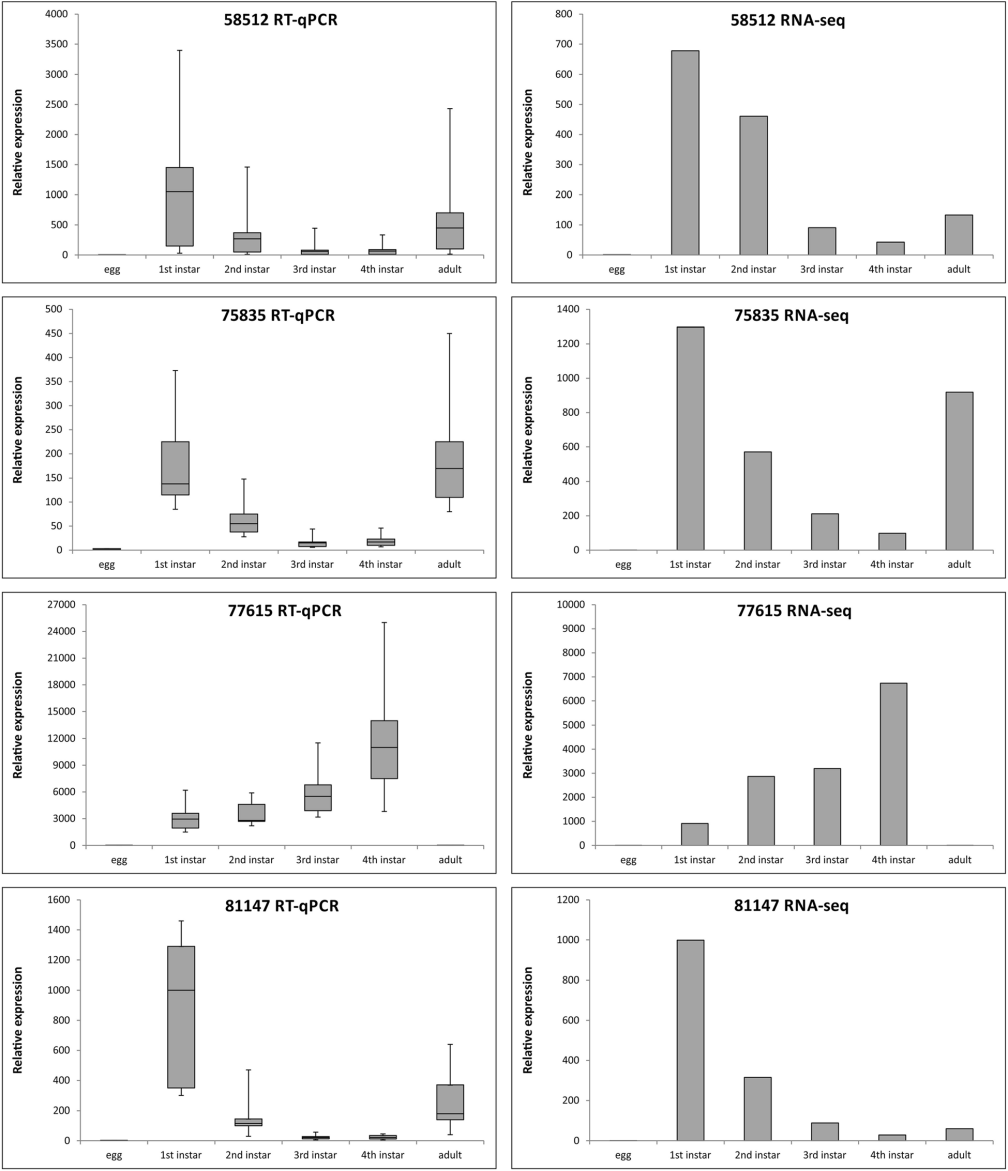
Comparison of relative gene expression based on RT-qPCR or RNA-seq among developmental stages. Based on the RNA-seq data, 23 contigs with significant differential expression between developmental stages (egg, larval and adults) were chosen to be validated by RT-qPCR. Relative expression of 4 genes (58512; 75835; 77615; and 81147; Additional file 5: Table S5) based on RT-qPCR is represented by whiskers-box plots with standard deviations, and RNA-seq data is represented by fold-differences. Whisker-box plots display average values from three biological replicates, and the box contain 50 % of the variation among samples, while the remaining 50 % are divided between the upper quartile (25 %) and the lower quartile (25 %), represented by error bars (whiskers). The other 18 genes are presented on Additional file 6: Figure S1

Of the 23 transcripts evaluated, 22 (95.6 %) were differently expressed between the distinct libraries, with only one contig (12524) showing no significant difference (not shown). Figure 4 illustrates the relative expression results of four DET (58512, 75835, 77615, and 81147) by RT-qPCR and RNA-seq, while the remaining 18 are presented on Additional file 6: Figure S1. Among the 22 transcripts differently expressed by RT-PCR, eight (2406, 12828, 21584, 23824, 36279, 58512, 77615 and 81147) presented a highly similar pattern of transcript accumulation to the one derived from the RNA-seq data, with comparable fold-change (Fig. 4; Additional file 6: Figure S1). For the contigs 11301, 20172, 36206, 38086, 50455 and 75835, the fold-change values were different, but transcript accumulation evaluated by RT-qPCR displayed a similar pattern to the RNA-seq analysis (Fig. 4; Additional file 6: Figure S1). For the remaining eight transcripts, the changes in transcript accumulation detected by RT-qPCR were not related to the pattern observed for the RNA-seq data, but there was clear differential expression between stages (Fig. 4; Additional file 6: Figure S1).

### Search for genes involved in the RNA interference mechanism

**Fig. 5:**
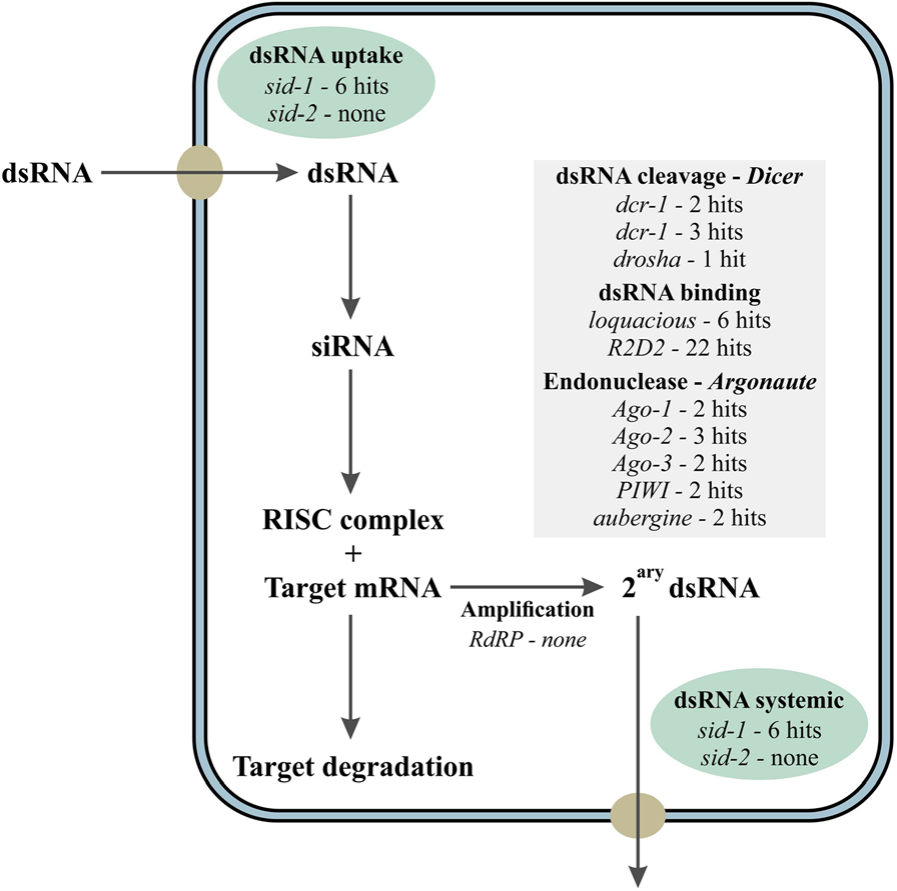
The core set of genes of RNAi mechanism identified in the *Tuta absoluta* transcriptome. The analysis of the transcriptome identified genes at minimum *e-value* < e^−30^ related with the process of RNAi, including dsRNA uptake (*sid-1* and *sid-2*); dsRNA cleavage into siRNA (*Dicer* or *drosha*); dsRNA binding (*loquacious* and *R2D2*); target degradation by endonuclease activity (*argonaute*; *PIWI*; *aubergine*); and signal amplification by RNA-dependent RNA polymerase (*RdRP*) with respective number of identified hits at *e* < e^−30^. Panel adapted from [1]

The *T. absoluta* transcriptome dataset was queried for the basic set of genes of the RNAi machinery using homologous genes from *Bombyx mori*, *Drosophila* and *Caenorhabditis elegans*. The main functional steps of siRNA and microRNA processing investigated for homologues included dsRNA uptake by SID-1 and SID-2, dsRNA cleavage into siRNA by Dicer, RISC assembly including putative helicases (*Armitage*, *SpindleE*, *Rm62*) and dsRNA binding (*R2D2* and *Loquacious*), target degradation by endonuclease activity (*Argonaute* family, *Aubergine*), and signal amplification by RNA-dependent RNA polymerase (*RdRP*) (Fig. 5; full list in Additional file 7: Table S6). In this analysis, we identified homologues for *sid-1-1* (4 contigs), *sid-1-2* (10 contigs), and *sid-1-3* (6 contigs), which together represented 11 distinct contigs. We could not find, however, homologous sequences for *sid-2*. Additionally, we searched for orthologues of three *C. elegans* genes (*rsd-2*, *rsd-3*, and *rsd-6*) involved in the germ-line related systemic RNAi response [31], but did not identify any in the *T. absoluta* transcriptome (Additional file 7: Table S6). Two homologue sequences were found for *dcr-1*, three for *dcr-2*, and one for *drosha* (Fig. 5). The search for dsRNA binding factors revealed putative homologues for *loquacious* (6 contigs) and *R2D2* (22 contigs), while the search for endonucleases identified homologues for *argonaute-1* (two contigs), *argonaute-2* (three contigs), *argonaute-3* (two contigs), *piwi* (two contigs), and *aubergine* (two contigs). No contigs displayed significant similarity with a RNA-dependent RNA Polymerase (*RdRP*) (Fig. 5). A complete list of the genes associated with the basic mechanism of siRNA and microRNA processing with respective contigs with similarity at an *E*-value < e^−30^ and per sample FPKM normalized expression values are presented as Additional file 7: Table S6, including those without significant matches. The original results from the Blastx searches are presented as Additional file 8: Table S7.

### Search for genes involved in hormone biosynthesis

**Fig. 6:**
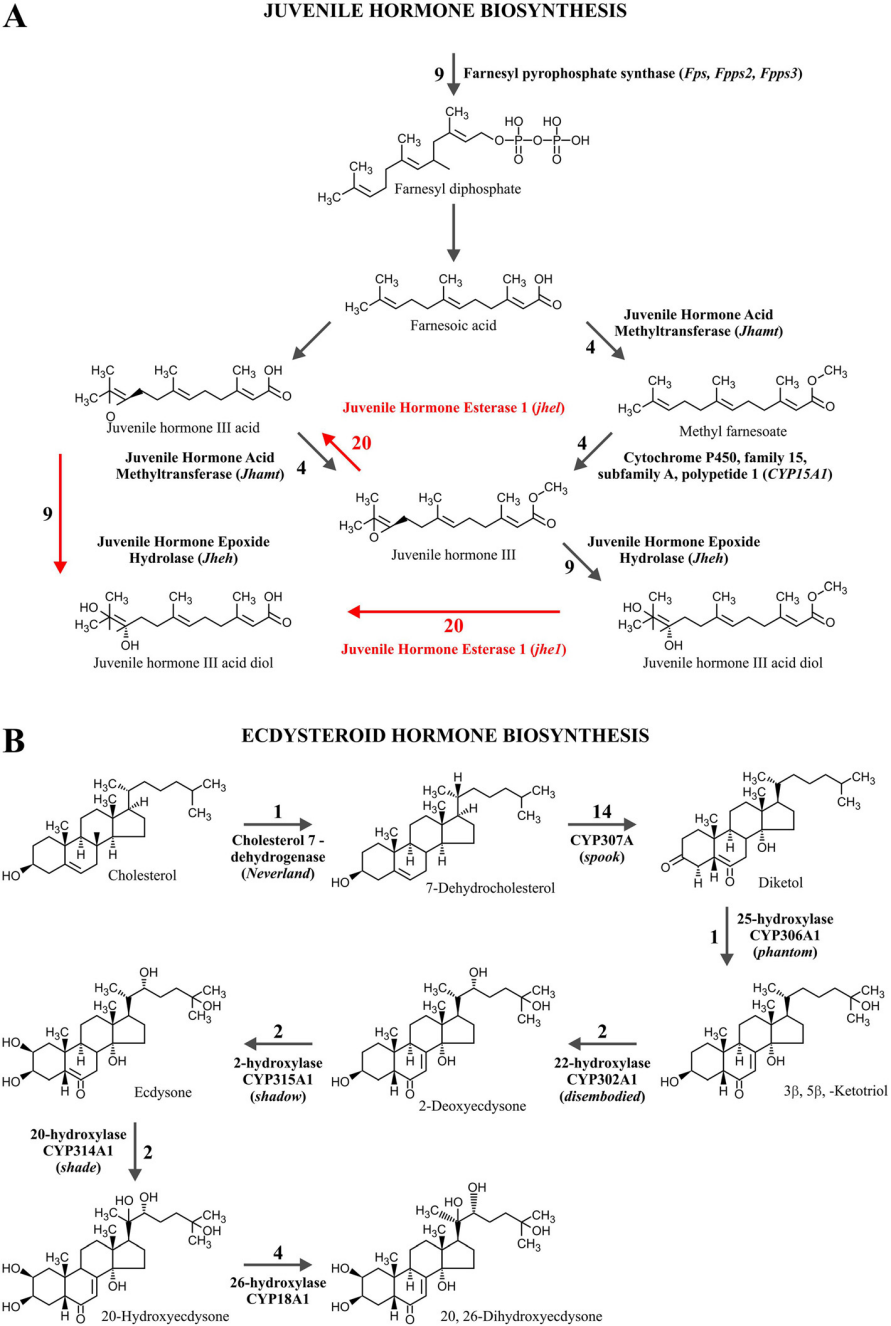
Juvenile hormone and ecdysteroid hormone biosynthesis with number of hits from the *Tuta absoluta* transcriptome. **a**. The analysis of the transcriptome identified homologues genes coding for enzymes of the Juvenile Hormone biosynthetic pathway, including Farnesylpyrophosphate synthase (*Fpps*); Juvenile Hormone Acid Methyltransferase (*Jhamt*); and Cytochrome P450 (*CYP15A1*); and for degradation of the Juvenile Hormone, with Juvenile Hormone Epoxide Hydrolase (*Jheh*); Juvenile Hormone Esterase 1 (*jhe1*), with respective number of identified hits at *e* < e^−30^ by Blastx. Chemical structures obtained from KEGG. **b**. The analysis of the transcriptome identified homologues genes coding for enzymes of the Ecdysteoid hormone biosynthetic pathway, including Cholesterol 7-dehydrogenase (*Neverland*); and a series of cytochrome P450 enzymes encoded by the Halloween genes, such as *spook* (*CYP307A*); *phantom* (*CYP306A1*); *disembodied* (*CYP302A1*); *shadow* (*CYP315A1*); and *shade* (*CYP314A1*). For ecdysteroid inactivation, a 26-dehydroxylase (*CYP18A1*), with respective number of identified hits at *e* < e^−30^ by Blastx. Chemical structures obtained from KEGG

A search was performed to identify genes encoding enzymes of the sesquiterpenoid juvenile hormone (JH) and ecdysteroid biosynthetic pathways (Fig. 6). For JH III biosynthesis, using homologues from *B. mori*, nine contigs with high similarity (*E* < e^−30^) to farnesylpyrophosphate synthase genes (*Fps*, *Fpps2*, *Fpps3*) were identified, four to juvenile hormone acid methyltransferase (*Jhamt*), and four to cytochrome P450 family 15, subfamily A, polypeptide 1 (*CYP15A1*). For JH III degradation, 20 contigs with similarity to juvenile hormone esterase (JHE) and nine to juvenile hormone epoxide hydrolase (JHEH) were identified (Fig. 6a). All contigs identified, and the respective per sample FPKM normalized expression values are presented as Additional file 9: Table S8. The original results from the Blastx searches are presented as Additional file 10: Table S9.

In the case of ecdysteroid biosynthesis, one contig was identified matching homologues of the *Neverland* gene (Cholesterol dehydrogenase). Subsequently, contigs with high similarity to cytochrome P450 enzymes encoded by the *Halloween* genes were detected, with 14 similar to *spook* (*CYP307A*), one to *phantom* (*CYP306A1*), two to *disembodied* (*CYP302A1*), two to *shadow* (*CYP315A1*), and two similar to *shade* (*CYP314A1)* (Fig. 6b). For ecdysteroid inactivation, four contigs with similarity to a 26-dehydroxylase (*CYP18A1*), which is putatively responsible for 20,26-Dihydroxyecdysone production, were identified. A complete list of the genes associated with ecdysteroid biosynthesis with respective contigs with similarity at *E* < e^−30^ and per sample FPKM normalized expression values are presented as Additional file 9: Table S8. The original results from the Blastx searches are presented as Additional file 10: Table S9.

### Gene silencing of novel RNAi candidate genes

From the transcriptome of *T. absoluta*, we selected ten genes that were highly expressed in the first larval stages and verified their homology to important genes from other species based on Blastx (Additional file 11: Table S10). The target sequences were then amplified from cDNA derived from larval and pupal stages using specific primers containing the recombination sequences *attL1* and *attL2* (Additional file 11: Table S10) for later recombination in binary vectors for plant transformation, followed by cloning in pGEM-T. Five sequences were successfully cloned and tested in RNAi assays conducted by providing *in vitro* transcribed dsRNA in solution to detached tomato leaflets. Together with the novel candidates, the experiment included three other target genes (*V-ATPase*, *AK*, *EcR*) that were previously evaluated by our group in *T. absoluta* (Camargo et al., unpublished method), together with a negative control (dsRNA specific for GFP).

**Fig. 7:**
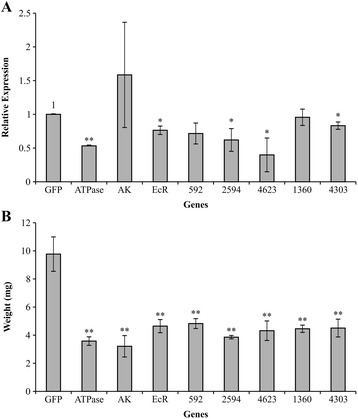
RNAi effects of 8 genes on *Tuta absoluta* 1^st^ instar larvae fed in tomato leaflets. **a**. Relative expression of target genes in larvae, five days after fed on tomato leaflets that absorbed 10 μg of dsRNA from each gene target (592; 2594; 4623; 1360; and 4303), and a *eGFP* control, plus three genes with previous positive results (*V-ATPase*; *AK*; *EcR*; Camargo et al., unpublished method) all in triplicates. Gene expression is expressed in relation to *eGFP*-fed control. **b**. Average weight of larvae (*n* = 30) under the same conditions as above. Bars represent standard deviation; ‘*” or ‘**”represent significant at *P* < 0.05 or *P* < 0.01, respectively by T-student test. Gene expression used *Rpl5* as gene reference

A feeding assay was conducted with biological triplicates, with fifty 1^st^ instar larvae feeding on one leaflet. After 5 days feeding on leaflets treated with dsRNA, the larvae were weighed and RNA was extracted to evaluate the effect of RNAi by RT-qPCR (Fig. 7). A significant reduction in larvae weight was detected for all of the genes tested compared with the GFP control, indicating a significant growth delay for the larvae (Fig. 7b). On the other hand, not all genes presented a significant decrease in transcript accumulation (Fig. 7a). The genes *AK* and the contigs 592 and 1360 did not show a difference in transcript accumulation compared with the GFP control. *AK* showed the lowest average larvae weight which was not reflected by a reduction in transcript accumulation, and in this case, a large variation in transcript accumulation was observed.

## Discussion

One of the main requisites for developing an RNAi-mediated pest control strategy is the identification of specific and effective target genes that have a significant impact on insect development or viability, minimizing potential crop losses. Many studies have explored RNAi as a tool to control insects, and over 90 target genes have been evaluated in more than 30 species in eight orders (reviewed in [14]). The selection of targets has been based on a candidate gene approach (by orthology to previously described genes) or a screening approach to discover novel target genes [14]. A recurrent problem related with the use of the candidate gene approach is low predictability, with a lack of correspondence of resulting phenotypes, even between close species [18]. One way to identify novel target genes is through genomic information. However, agricultural insect pests are mostly non-model organisms, and little or no genomic information is available for most of them. Recently thought, the development of RNA-seq platforms has enabled the quick assembly of large datasets.

In this study, we were able to build a transcriptome dataset from six developmental stages of *T. absoluta* based on an overall total of *ca*. 245 million reads, assembled into 93,477 contigs with an average size of 1,574 bp. The total number of contigs of the reference assembly was higher than expected based on the total number of genes found in Lepidoptera species with full genome sequences available, such as *B. mori* with 18,501 genes [32] and *Plutella xylostella* with 18,071 predicted genes [33]. However, *de novo* transcriptome assembly without a reference genome represents a computational challenge [34, 35], which may result in an imperfect assembly, particularly with short reads [36]. Using the Illumina sequencing platform, comparable results were obtained for the transcriptome of the Asian corn borer (*O. furnacalis*; Lepidoptera), from which 124,043 contigs were generated with a mean size of 198 bp and an N50 of 211 bp, and re-aligned into 79,825 scaffold sequences [16]. For another Lepidoptera, the beet armyworm (*S. exigua*), a transcriptome was built from pooled samples of all the developmental stages, with over 34 million reads, assembled into 31,414 contigs with an N50 of 542 bp [18]. Similarly, the transcriptome of the feeding canal of the grain aphid (*S. avenae*; Hemiptera) was assembled into over 83,000 (post-feeding) or 93,000 (pre-feeding) contigs, which were re-aligned into more than 40,000 scaffolds with a mean size of 421–432 bp [17]. Based on the Roche 454-pyrosequencing platform, a cotton ball weevil (*A. grandis*; Coleoptera) transcriptome was generated with over 500,000 reads, which were assembled into 20,841 contigs with an average size of 676 bp [20]. When directly comparing transcriptome assemblies, the distinct quality parameters adopted in each study must be considered [36]. Nevertheless, our *de novo* assembly of the *T. absoluta* transcriptome was comparable in size with previously published assemblies sequenced using the same sequencing platform. Additional quality parameters obtained were the N50 and N95 values, which were 2,427 bp and 480 bp, respectively, indicating the formation of large contigs. Additionally, GO annotation showed that the major biological processes, molecular functions, and cellular components were at similar proportions to other transcriptomes [18]. Thus, we considered the assembled transcriptome to have achieved our main objective: developing a resource for downstream applications, particularly the use of RNAi in crop protection.

To characterize the RNAi machinery in *T. absoluta*, we queried the transcriptome for the presence of the core genes involved in RNAi (Fig. 5) using insect or nematode homologues (Additional file 7: Table S6). Although it is a highly conserved cellular mechanism among eukaryotes, some gene homologues responsible for key functions in RNAi are absent in certain taxa. Also, difference responses to RNAi have been reported, particularly among Lepidoptera. Some of the genes involved in the RNAi mechanism, such as *ago1* and *dcr1*, have been used as gene targets for silencing, severely impairing ecdysis in the brown planthopper *N. lugens* [19].

Most of the expected members of the RNAi core gene set were identified with an *E*-value < e^−30^, generally with one to three contigs each, which reinforced the quality of the assembled transcriptome (Fig. 5). Some contigs may represent a single gene that could not be assembled under the parameters we set. Lepidopteran species, together with other more derived insects such as the dipterans, are believed to be more refractory to systemic RNAi [22]. One proposed reason for the poor RNAi response in *Drosophila* is the absence of a canonical 11-helix transmembrane channel protein, named SID-1 (*systemic RNA interferencedefective-1*), originally identified in *C. elegans* [31]. Another gene (*sid-2*), encoding a gut lumen transmembrane protein associated with dsRNA uptake identified in *C. elegans*, is also absent in *Drosophila* [1]. Lepidoptera contain three copies of SID-1 proteins, which are believed to be involved in dsRNA uptake and systemic spread [19], but there are arguments against this view [31]. In the *T. absoluta* transcriptome, four contigs showed homology to the *sid-1-1* gene of *B. mori*, 10 contigs to *sid-1-2*, and six to *sid-1-3*, but no *sid-2* was identified (Fig. 5; Additional file 7: Table S6). Thus, *T. absoluta* has the appropriate genes for dsRNA uptake and systemic spread, which is the primary step for effective gene silencing. A proposed alternative mechanism of systemic RNAi in *Drosophila* involves receptor-mediated endocytosis dsRNA uptake [37], and 23 orthologous genes have been identified in *N. lugens* [19].

Similar to *Drosophila*, two *Dicer* paralogues (*Dcr-1* and *Dcr-2*) recognized to differentially process miRNA precursors (*Dcr-1*) or long dsRNA (*Dcr-2*) [21] were identified in the transcriptome of *T. absoluta. Drosha*, which showed a single highly significant matched contig, shares similar functional features with *Dicer* but processes miRNA precursors in the nucleus. *R2D2*, *loquacious* and *pasha* contain a dsRNA-binding domain (dsRBD), mediate dsRNA binding to the RISC complex and are considered co-factors of Dicer (loquacious as a co-factor of DCR-1 and R2D2 of DCR-2) and Drosha (Pasha) [19]. We identified 22 contigs homologous to *R2D2* and six to *loquacious*, but only one to *pasha* that was below our cut-off value (Additional file 7: Table S6; Fig. 5). From the Argonaute (Ago) protein family associated with small RNA identification and binding, and target cleavage, we identified *Ago-1*, *Ago-2*, and *Ago-3*, *PIWI*, and *Aubergine*, all based on homologues from *B. mori* (Fig. 5; Additional file 7: Table S6). Ago-1 and Ago-2 are involved in microRNA and siRNA pathways, respectively, while Ago-3, PIWI and Aubergine are associated with piRNA [19]. Furthermore, we searched for a secondary Argonaute family from *C. elegans*, including *PPW-1*, *PPW-2*, *Sago1*, and *Sago2*, in our dataset (Additional file 7: Table S6), but no homologous sequences were found. This result is similar to what was reported for *N. lugens* [19]. We also identified putative helicases, such as the *Asp spindle* and *Rm62*, but not *armitage* or *tudor sn* nucleases (Additional file 7: Table S6). As previously described in other insects, no RNA-dependent RNA Polymerase (*RdRP*) was detected, even at low significance (*E* < e^−3^). According to the homologous genes identified, *T. absoluta* follows the pattern of other Lepidoptera species, with the RNAi machinery available for successful RNAi, even considering the lack of RdRP. Lepidoptera species are recognized to be recalcitrant to systemic RNAi [22], and variable sensitivity to systemic RNAi has been reported among the Lepidoptera, but the causes for this are still undefined [7, 22, 23].

Similarly, we were able to identify all the genes encoding enzymes of the sesquiterpenoid juvenile hormone and ecdysteroid biosynthetic pathways as potential targets for RNAi silencing (Fig. 6). In the JH III biosynthesis pathway, there were 4–9 contigs homologous to each gene from *B. mori*, while 9–20 contigs were identified for the degrading enzymes JHE and JHEH (Additional file 9: Table S8). The biosynthesis of ecdysteroid from sterols obtained from the diet depends on the successive action of a dehydrogenase (*Neverland*), followed by a series of cytochrome P450 enzymes encoded by the *Halloween* genes. All of these genes were identified in our dataset, matching mostly with one or two contigs, except for *spook*, with 14 contig hits (Fig. 6). As observed for genes of the RNAi machinery, individual detailed analysis of each of these contigs may reveal a single or few copies for each gene, which may derive from the parameters defined in the assembly process. Interfering with hormone biosynthesis would be an attractive RNAi approach to affect insect development [38, 39], if a systemic effect is feasible in *T. absoluta* since the two major classes of hormones are synthesized away from the midgut, where ingested dsRNA reach.

Major losses from *T. absoluta* infestation derive from galleries formed during larval herbivory [28]. Therefore, we prioritized genes that were more expressed and potentially more relevant at the first larval stages to develop an RNAi approach that would reduce the impact of insect attack. However, it is important to mention that other genes could have an effect on the pest population density. Of the overall 3,917 significantly DET, 1,577 were differentially expressed between eggs and all larval stages and 1,128 between larval stages and adults. Of these DET, 411 were differentially expressed between eggs and common to all larval stages, and another 37 were differentially expressed between eggs and the 1^st^ and 2^nd^ larval stages only. Another 298 were differentially expressed between adults and common to all larval stages (Fig. 2). To validate these differences in expression, we chose 23 DET: 15 among the 411 DET (contigs 10806, 11301, 12524, 12828, 13135, 16411, 16428, 17745, 20172, 21584, 23824, 38086, 50455, 55173, and 6681), five among the 37 (contigs 2406, 36279, 58512, 75835, and 81147), and three among the 298 DET (contigs 26572, 36206, and 77615) between adults and common to all larval stages. Differences in expression between the developmental stages tested were validated for all genes analyzed, except for one contig (12524). The DET between eggs and the 1^st^ and 2^nd^ larval stages (2406, 36279, 58512, 75835, 81147) clearly showed greater expression at the first two stages from the RT-qPCR data, corroborating the RNA-seq read count (Fig. 4; Additional file 6: Figure S1), demonstrating that the differences detected among libraries were *bona fide*. In general, the expression profiles among developmental stages were highly analogous, even for the fold-change values, between the RT-qPCR and RNA-seq data for eight contigs (2406, 12828, 21584, 23824, 36279, 58512, 77615 and 81147). For six contigs (11301, 20172, 36206, 38086, 50455 and 75835), the trend in expression, but not the fold change, was greatly similar. For the remaining eight contigs, there were differences in expression patterns among developmental stages, but there were definite differences in expression between eggs and larvae. Thus, the significant differential expression between stages, validated by RT-qPCR, may assist our choice of target genes to focus on those more expressed in the first larval stages.

Notably, no significant difference in gene expression was observed among any of the larval stages analyzed. One of the reasons for this may derive from the mode of larval sampling used to obtain RNA; they were not staged, which might have led to an overlap between larval instars sampled. We prioritized larval size and age instead of instar to center the RNAi effect at the early stages of herbivory. Particularly when comparing the number of DET between eggs or adults and each larval stage, the numbers differed in each comparison. In this case, it appears that besides the sampling bias, the lack of significant differences in transcripts between larval stages might have derived from a lack of statistical power to detect significance among the differences that occurred. The heatmap and cluster analyses showed a close resemblance in transcript profiles among the larval stages, particularly to the immediate previous or subsequent stage on a similarity gradient, which corroborated the potential lack of discrimination between stages during sampling and/or a biological cause. The gene expression profile from eggs was the most distinct from all the other stages, particularly from adults.

To prove the concept of using transcriptome analysis to discover novel RNAi target genes, we performed gene silencing of transcripts that were highly expressed in the first larval stages (Fig. 7). We previously developed an RNAi delivery assay based on feeding recently emerged larvae with detached tomato leaflets that had absorbed aqueous solution containing *in vitro* transcribed dsRNA of the target genes (Camargo et al., unpublished method). Using this assay, we successfully demonstrated a significant reduction in larvae weight for all the genes tested in comparison with the GFP control, 5 days after the treatments. Three of the five novel genes tested showed a significant decrease in transcript accumulation under the same conditions. However, the decrease in transcript accumulation in *T. absoluta* was not dramatic, possibly for a number of reasons. One possibility is the criterion we favored for selecting target genes considering that high levels of expression might indicate essentiality. Therefore, it could be that these highly expressed transcripts have a high functional threshold, and a mild reduction in expression would compromise gene function.

## Conclusion

We demonstrated that the transcriptome dataset generated here represents a valuable genomic resource for screening potential gene targets that affect the development or survival of *T. absoluta* larvae. We successfully identified all of the genes we expected to find in this species, indicating we had sufficient transcriptome sequencing coverage. From the assembled transcriptome, we detected core components of the *T. absoluta* RNAi machinery, enabling the design of dsRNA experiments for gene expression modulation. Finally, five novel genes identified as differentially more expressed at the 1^st^ stage in our analysis were demonstrated to be effective potential RNAi targets by reducing larval weight and can be considered good candidates for use in RNAi-mediated crop protection.

## Methods

### Biological material

The colony of *T. absoluta* was originally established from two distinct populations provided by the Insect Biology Laboratory, Dept. of Entomology (ESALQ/USP, Piracicaba, SP, Brazil) and from Bayer Crop Science (Uberlândia, MG, Brazil). The colony has been reared in whole tomato plants or detached leaves under laboratory conditions (temperature 25 ± 2 °C; 60 ± 10 % relative humidity; 14 h photoperiod) [40]. The ‘Santa-Clara’ tomato cultivar was used for *T. absoluta* maintenance and dsRNA uptake experiments.

### *Tuta absoluta* RNA extraction for sequencing

Total RNA was extracted separately from whole individuals (eggs, adults, and larval bodies at various stages). Larval staging in *T. absoluta* requires the measurement of the cephalic capsule [41], so we putatively categorized individuals by larval length and days post-emergence (DPE) as 1^st^ stage (0.6-1.7 mm long; emergence to 3–4 DPE), 2^nd^ stage (1.2-3.0 mm long; 5–6 DPE), 3^rd^ stage (2.1-5.0 mm long; 7–8 DPE) and 4^th^ stage (3.9-8.0 mm long; 10–11 DPE). To obtain approximately 50–100 mg of fresh weight, RNA was extracted from *ca.* 1,000 eggs; *ca.* 2,000 individuals of the 1^st^ instar; *ca.* 500 individuals of 2^nd^; *ca.* 100 individuals of 3^rd^; *ca.* 30 individuals of the 4^th^; and *ca.*20 adults, using TRIzol (Invitrogen, Carlsbad, CA, USA). An average of 10 μg total RNA from each developmental stage was sent on dry ice to Macrogen (Seoul, South Korea) for library construction and sequencing. Libraries were constructed using the TruSeq RNA Sample Prep kit (Illumina, San Diego, CA, USA) and sequenced using a HiSeq 2000 (Illumina) to generate inward paired-end reads of 100 bp.

### Data processing, transcriptome assembly and annotation

The absence of a reference genome for *T. absoluta*, together with a high coverage of sequenced RNA libraries, led to the use of a *de novo* transcriptome assembly pipeline. Initially, raw RNA-seq data were trimmed of adaptors and sequencing artifacts, as well as low quality fragments, with the NGS QC Toolkit [42]. High-quality reads were then subjected to *in silico* normalization prior to *de novo* assembly, to reduce the sequencing coverage of highly represented regions with a fragment density higher than 30×. This step was performed to reduce computational complexity without affecting the quality of the assembled transcriptome [35]. Using the *de novo* transcriptome assembler tool from Trinity [35], the normalized data were assembled with a minimum fragment overlap of 35 bp. Only contigs longer than 300 bp were included in the assembled *T. absoluta* transcriptome for further analyses, including gene ontology (GO) sequence annotation using Blast2Go [29] and KAAS (KEGG Automatic Annotation Server) [43] submission.

### Analysis of differentially expressed transcripts (DET) through developmental stages

Using the assembled transcriptome as a reference for *T. absoluta*, the sequenced filtered libraries, prior to *in silico* data normalization, were subjected to transcriptome expression analysis. The filtering coverage previously applied was not used at this stage to avoid interference with the read density when examining gene expression variation. The libraries were mapped to the reference transcriptome using Bowtie with default parameters [44]. Using a read-count methodology, the absolute number of mapped reads for each transcript was represented in a count matrix, with rows representing transcripts and columns representing fragment counts for a specific sample. We then applied a negative binomial distribution method for gene expression analysis with statistical significance. To control false-positives among the DET, a False Discovery Rate (FDR) correction [45] with a cut-off *p-value* <0.05 was applied over the calculated statistical significance. To perform this analysis, we included in our pipeline the R Bioconductor package DESeq [46]. Differential expression between developmental stages was screened by detecting genes with statistical significance. A list of differentially expressed or constitutive transcripts for each stage was produced and analyzed by Blast2GO for gene ontology annotations [29]. Quantitative differences in DET between the developmental stages were represented with Venn diagrams to identify common or exclusive genes among stages. The count matrix for all sequenced samples was also used to calculate a Euclidian distance matrix, which was used for hierarchical sample clustering. According to the most similar transcriptome profile calculated by a single linkage method, a dendrogram and a heatmap were generated, correlating sample expression profiles into colors ranging from red (identical profiles) to green (the most different profiles).

### Validation of differences in gene expression among developmental stages by quantitative amplification of reversed transcripts (RT-qPCR)

To validate differential expression among the developmental stages, 23 genes (Additional file 5: Table S5) were chosen for expression analysis by RT-qPCR. Total RNA from three biological replicates for each developmental stage (eggs, 1^sr^, 2^nd^, 3^rd^, and 4^th^ larval stages, and adults) was used to produce cDNA. Around 1 μg of total RNA was treated with DNase I and 20 U Ribolock (Fermentas, Burlington, Canada) at 37 °C for 30 min, with the reaction stopped by adding EDTA (50 mM) and heating to 65 °C for 10 min. One microgram DNAse-treated RNA samples were reversed transcribed in 20 μL reactions, containing 500 μM of each dNTP, 2.5 μM oligo dT, 5 mM DTT and 200 U Revertaid (Fermentas) in appropriate buffer at 50 °C for 30 min, followed by enzyme inactivation at 85 °C for 5 min. RT-qPCR reactions contained *ca*. 40 ng sample cDNA, 5 μL Fast SYBR Green Master (Invitrogen), and 0.2 μM of each gene-specific primer (Additional file 5: Table S5) in a 10 μL reaction volume. Amplifications were conducted starting at 50 °C for 10 min and 95 °C for 2 min, followed by 40 three-step cycles of 95 °C for 15 s, 60-61 °C for 25 s and 72 °C for 30 s in a Qiagen RotorGene-6000 (Qiagen, Valencia, CA, USA). Melting curves were determined after amplification between 72 °C and 95 °C. Reactions were conducted with technical triplicates and non-template controls. Primer efficiency was determined using a cDNA pool with serial dilutions (1, 10^−1^, 10^−2^ and 10^−3^). C_Q_ values were used to determine differences in expression based on [47]. Gene references were *RpL5* (large subunit 5 ribosomal protein), *Rpl23A* (large subunit 23A ribosomal protein) and *rRNA* (Additional file 5: Table S5). Data were analyzed using the software REST (Relative Expression Software Tool) [48].

### Searches for genes involved with the RNA interference mechanism and hormone biosynthesis

A list of genes encoding proteins associated with the RNAi mechanism was built based on the literature and *Bombyx mori* gene sequences were queried against the *T. absoluta* assembled transcriptome using Blastx with significant *E*-value < e^−30^. Similarly, genes from the juvenile hormone and ecdysteroid biosynthetic pathways were recovered from KEGG, and homologues from *B. mori* were used to search the *T. absoluta* transcriptome under similar conditions. RNA interference mechanism- and hormone biosynthesis-related transcripts were separately analyzed for each biological condition to show normalized FPKM expression values (Additional file 7: Table S6; Additional file 8: Table S7).

### Silencing novel target gene by RNAi

From the list of transcripts identified by RNA-seq, 10 genes highly expressed at the first larval stage were selected for evaluation as targets for RNAi. Primers were designed for each sequence (Additional file 11: Table S10) with flanking *attL1* and *attL2* border sequences to enable recombination with binary plasmids using Gateway® (Invitrogen) for future use in plant transformation assays. Amplification reactions were conducted in a 20 μL volume containing *ca*. 40 ng cDNA, 3 mM MgCl_2_, 100 μM of each dNTP, 0.2 μM each primer (Additional file 11: Table S10), and 1.5 U High Fidelity *Taq* DNA polymerase (Invitrogen) in appropriate buffer. Amplifications were conducted in a Veriti thermocycler (Applied Biosystems, Foster City, CA, USA) programmed to cycle at 95 °C for 2 min, followed by 35 cycles of 95 °C for 30 s; 45-60 °C for 60 s; 72 °C for 60 s according to the target (Additional file 11: Table S10), and a final cycle at 72 °C for 5 min. The products were electrophoresed in 1 % agarose gels, and the target fragments were excised, purified using a PureLink Quick Gel Extraction Kit (Invitrogen) and cloned into the pGEM-T Easy vector (Promega, Madison, WI, USA) using standard procedures. Identity was confirmed by sequencing three clones of each target gene in an ABI PRISM 3130 (Applied Biosystems).

### dsRNA synthesis

Confirmed clones containing fragments of *T. absoluta* target genes were used as a template for *in vitro* transcription reactions to produce dsRNA using T7 RNA polymerase (MegaScript T7, Life Technologies). Target sequences cloned into pGEM-T Easy were amplified using a T7 primer and a SP6 primer fused with a T7 sequence (TAATACGACTCACTATAGGGATTTAGGTGACACTATAG) to operate as a T7 promoter for *in vitro* transcription in both directions. Template amplification reactions contained 10 ng plasmid DNA, 1.5 mM MgCl_2_, 200 μM each dNTP, 0.5 μM of each primer, and 1 U *Taq* polymerase in a 20 μL volume. Temperature cycling for amplification started at 95 °C for 2 min, followed by 35 cycles of 15 s at 95 °C, 20 s at 60 °C, 30 s at 72 °C, and a final 5 min at 72 °C. Amplified fragments were run and purified from 1 % agarose gels as above. Purified products were quantified by fluorimetry and used for *in vitro* transcription in reactions containing 100 ng target DNA, 7.5 mM of each ribonucleotide, and 200 U MegaScript T7 in appropriate buffer in a final volume of 20 μL. The reactions were conducted at 37 °C for 16 h, followed by the addition of 2 U DNase for 15 min at 37 °C. Double-stranded RNA (dsRNA) was purified by precipitation with 7.5 M LiCl solution (30 μL) at −20 °C for 1 h, followed by centrifugation at 12,000 *g* for 15 min at 4 °C. The RNA pellet was washed with 70 % ethanol and resuspended in DEPC water. The green fluorescent protein (GFP) gene was used as a negative control. The vector pCAMBIA1302 was used as a template to amplify a negative control gene fragment (276 bp) using the primers GFP-F (TAATACGACTCACTATAGGGCAGTGGAGAGGGTGAA) and GFP-R (TAATACGACTCACTATAGGGTTGACGAGGGTGTCTC), both containing additional T7 sequences. Similar *in vitro* transcription was conducted using this template.

### RNAi delivery assays to feed *T. absoluta* larvae

Detached leaves from ‘Santa-Clara’ tomato had their petioles immersed into 200 μL water solution, containing 10 μg of dsRNA target genes (592, 2594, 4623, 1360, and 4303), the GFP control, or three genes with previous positive results (*V-ATPase*, *AK*, *EcR*; Camargo et al., unpublished method), all in triplicate. After uptake, 50 individuals at the 1^st^ larval instar were gently placed on the leaves for feeding. Negative controls used dsRNA derived from GFP and every treatment contained three replicates. After 5 days of treatment, 30 larvae were removed and weighed. Total RNA was extracted from these larvae and used to evaluate target gene expression by RT-qPCR.

### Availability of supporting data

The data sets supporting the results of this article are available in the National Center for Biotechnology Information (NCBI) repository (Bioproject PRJNA291932 http://www.ncbi.nlm.nih.gov/bioproject/PRJNA291932/, and Sequence Read Archives SRS794929 http://www.ncbi.nlm.nih.gov/sra/?term=SRS794929/).

## Additional files

Additional file 1: Table S1. Biological process category annotation by Gene Ontology for differentially expressed transcripts (DET) for pair-wise comparisons between developmental stages of *Tuta absoluta* by Blast2Go. (PDF 16 kb) Additional file 2: Table S2. Molecular function category annotation by Gene Ontology for differentially expressed transcripts (DET) for pair-wise comparisons between developmental stages of *Tuta absoluta* by Blast2Go. (PDF 8 kb) Additional file 3: Table S3. Cellular component category annotation by Gene Ontology for differentially expressed transcripts (DET) for pair-wise comparisons between developmental stages of *Tuta absoluta* by Blast2Go. (PDF 8 kb) Additional file 4: Table S4. Kegg-based metabolic pathway annotation for differentially expressed transcripts (DET) for pair-wise comparisons between developmental stages of *Tuta absoluta* by Blast2Go. (PDF 10 kb) Additional file 5: Table S5. Primer sequences of 23 genes differentially expressed between the developmental stages with expected product size, and three gene references used in RT-qPCR to validate differences in read counts among libraries. Annotation is derived from Blast2Go or manual. (PDF 53 kb) Additional file 6: Figure S1. Comparison of relative gene expression based on RT-qPCR or RNAseq among developmental stages. Based on the RNAseq data, 23 contigs with significant differential expression between developmental stages (egg, larval and adults) were chosen to be validated by RT-qPCR. Relative expression of 18 genes (2406, 6681, 10806, 11301, 12828, 13135, 16411, 16428, 17745, 20172, 21584, 23824, 26572, 36206, 36279, 38086, 50455, and 55173; Additional File 5: Table S5) based on RT-qPCR is represented by whiskers-box plots with standard deviations, and RNAseq data is represented by fold-differences. Whisker-box plots display average values from three biological replicates, and the box contain 50 % of the variation among samples, while the remaining 50 % are divided between the upper quartile (25 %) and the lower quartile (25 %), represented by error bars (whiskers). (PDF 66 kb) Additional file 7: Table S6. List of genes associated with RNAi mechanism identified in the *T. absoluta* assembled transcriptome at *E-*value < e^−30^ using homologues, particularly from *Bombyx mori*. FPKM values for each transcript normalized per library is presented. Red rows represent undetected genes within the *T. absoluta* transcriptome for all sequenced stages, while grey rows are those that did not reach the set *E-*value < e^−30^. (DOCX 66 kb) Additional file 8: Table S7. BlastX results with the list of genes associated with RNAi mechanism identified in the *T. absoluta* assembled transcriptome at *E-*value < e^−30^ using homologues, particularly from *Bombyx mori*. (PDF 263 kb) Additional file 9: Table S8. List of genes associated with biosynthesis of the two major hormone classes sesquiterpenoid juvenile (JH) and the ecdysteroid hormones identified in the *T. absoluta* assembled transcriptome at E-value < e^−30^ using homologues particularly from *Bombyx mori*. FPKM values for each transcript normalized per library is presented. Red rows represent undetected genes within the *T. absoluta* transcriptome for all sequenced stages, while grey rows are those that did not reach the set *E-*value < e^−30^. (PDF 118 kb) Additional file 10: Table S9. BlastX results with the list of genes associated with biosynthesis of the two major hormone classes sesquiterpenoid juvenile (JH) and the ecdysteroid hormones identified in the *T. absoluta* assembled transcriptome at E-value < e^−30^ using homologues particularly from *Bombyx mori*. (PDF 468 kb) Additional file 11: Table S10. Primers used to amplify target-gene fragments flanked by *attL1* and *attL2* sequences (underlined) for each gene, with expected amplicon size in base pairs (bp) and annealing temperature adopted. (PDF 49 kb)

## Abbreviations

dsRNA: Double-stranded RNA
RNAi: RNA interference
RISC: RNA-induced silencing complex
RdRP: RNA-dependent RNA polymerase
JH: Juvenile hormone
IPP: Isopentyl pyrophosphate
DMAP: Dimethylallyl pyrophosphate
JHE: Juvenile hormone esterase
JHEH: Juvenile hormone epoxide hydrolase
RNA-seq: Large-scale RNA sequencing
DET: Differentially expressed transcripts
GO: Gene ontology
RT-qPCR: Quantitative amplification reaction of reversed transcripts
SID: Systemic RNA interference defective
Ago: Argonaute
GFP: Green fluorescent protein
