# Supplementary material for: De novo transcriptome assembly and analysis to identify potential gene targets for RNAi-mediated control of the tomato leafminer (Tuta absoluta)
Source: BMC Genomics. 2015 Aug 26;16(1):635. doi: 10.1186/s12864-015-1841-5 (PMC4550053; doi:10.1186/s12864-015-1841-5)
Supplement: Additional file 1: Table S1. — Biological process category annotation by Gene Ontology for differentially expressed transcripts (DET) for pair-wise comparisons between developmental stages of Tuta absoluta by Blast2Go. (PDF 16 kb) [file 12864_2015_1841_MOESM1_ESM.pdf]

Table S1. Biological process category annotation by Gene Ontology for differentially expressed transcripts (DET) for pair-wise comparisons between developmental stages of *Tuta absoluta* by Blast2Go.

Compared stages	Gene Ontology Term	#Seq	Score
**Adults x Eggs**	single-organism process	108	40.22
	metabolic process	111	31.71
	cellular process	78	22.26
	developmental process	34	18.98
	multicellular organismal process	76	16.29
	response to stimulus	36	13.54
	biological regulation	64	9.74
	localization	30	5.99
	signaling	18	4.46
	cellular component organization or biogenesis	21	3.43
	cell killing	4	1.44
	locomotion	8	1.42
	reproduction	4	1.3
	immune system process	8	1.27
	multi-organism process	2	0.96
	biological adhesion	2	0.73
**Eggs x 1st stage larvae**	metabolic process	52	19.88
	single-organism process	72	14.45
	cellular process	33	7.88
	response to stimulus	12	5.9
	multicellular organismal process	59	5.67
	biological regulation	54	4.93
	developmental process	14	3.98
	localization	20	3.85
**Eggs x 2nd stage larvae**	metabolic process	93	39.14
	single-organism process	96	26.53
	cellular process	53	13.12
	developmental process	23	8.74
	response to stimulus	13	7.77
	multicellular organismal process	71	7.48
	localization	30	6.16
	biological regulation	56	5.38
	reproduction	2	1.2
	immune system process	4	1.14
	multi-organism process	2	0.96
	cellular component organization or biogenesis	9	0.94
	signaling	6	0.75
	biological adhesion	2	0.26
	locomotion	1	0.13
**Eggs x 3rd stage larvae**	metabolic process	126	55.51
	single-organism process	101	36.54
	cellular process	61	20.41
	response to stimulus	27	14.42
	developmental process	23	11.46

	multicellular organismal process	66	9.07
	biological regulation	55	6.9
	localization	30	6.55
	cellular component organization or biogenesis	21	4.56
**Eggs x 4th stage larvae**	metabolic process	130	52.48
	single-organism process	104	35.69
	cellular process	66	21.79
	response to stimulus	31	17.21
	developmental process	21	8.61
	multicellular organismal process	64	7.81
	localization	32	7.5
	biological regulation	55	6.84
	cellular component organization or biogenesis	26	5.34
	immune system process	11	2.92
**Adults x 1st stage larvae**	single-organism process	35	19.78
	metabolic process	67	17.12
	response to stimulus	23	10.67
	cellular process	33	9.77
	multicellular organismal process	18	8.95
	developmental process	13	8.32
	biological regulation	12	3.45
	localization	13	3.44
	cellular component organization or biogenesis	18	2.5
	cell killing	4	1.44
	immune system process	8	1.36
	reproduction	1	0.8
	signaling	5	0.63
	locomotion	3	0.56
	multi-organism process	1	0.36
**Adults x 2nd stage larvae**	metabolic process	82	22.89
	single-organism process	41	22.43
	cellular process	37	12.74
	developmental process	18	11.3
	multicellular organismal process	22	9.17
	response to stimulus	21	7.82
	localization	15	3.81
	biological regulation	13	2.91
	cellular component organization or biogenesis	17	2.28
	cell killing	4	1.44
	immune system process	8	1.36
	reproduction	1	0.8
	locomotion	3	0.56
	multi-organism process	1	0.36
	signaling	3	0.34
**Adults x 3rd stage larvae**	metabolic process	63	22.77
	single-organism process	15	8.54
	response to stimulus	14	7.03
	multicellular organismal process	8	3.52

	localization	10	3.09
	developmental process	5	3.06
	cellular process	9	2.16
	biological regulation	9	1.96
	immune system process	4	1.14
	cellular component organization or biogenesis	5	0.42
	multi-organism process	1	0.36
	locomotion	2	0.35
	signaling	2	0.21
**Adults x 4th stage larvae**	metabolic process	50	18.72
	single-organism process	11	6.92
	response to stimulus	13	6.48
	localization	9	2.96
	developmental process	3	2.62
	biological regulation	8	1.86
	multicellular organismal process	5	1.83
	cellular process	7	1.75
	immune system process	5	1.5
	multi-organism process	2	0.58
	cellular component organization or biogenesis	4	0.34
	locomotion	1	0.13
	signaling	1	0.08
