# Supplementary material for: De novo transcriptome assembly and analysis to identify potential gene targets for RNAi-mediated control of the tomato leafminer (Tuta absoluta)
Source: BMC Genomics. 2015 Aug 26;16(1):635. doi: 10.1186/s12864-015-1841-5 (PMC4550053; doi:10.1186/s12864-015-1841-5)
Supplement: Additional file 2: Table S2. — Molecular function category annotation by Gene Ontology for differentially expressed transcripts (DET) for pair-wise comparisons between developmental stages of Tuta absoluta by Blast2Go. (PDF 8 kb) [file 12864_2015_1841_MOESM2_ESM.pdf]

Table S2. Molecular function category annotation by Gene Ontology for differentially expressed transcripts (DET) for pair-wise comparisons between developmental stages of *Tuta absoluta* by Blast2Go.

Compared stages	GO Term	#Seq	Score
**Adults x Eggs**	catalytic activity	169	57.89
	binding	92	32.52
	structural molecule activity	15	8.76
**Eggs x 1st stage larvae**	catalytic activity	87	34.25
	binding	65	15.83
	transporter activity	12	5.19
**Eggs x 2nd stage larvae**	catalytic activity	175	68.23
	binding	95	27.81
	structural molecule activity	38	20.71
	transporter activity	16	4.97
	developmental process	23	8.74
	response to stimulus	13	7.77
	multicellular organismal process	71	7.48
	localization	30	6.16
	biological regulation	56	5.38
**Eggs x 3rd stage larvae**	catalytic activity	223	85.49
	binding	117	40.68
	structural molecule activity	33	18.22
	transporter activity	16	3.77
**Eggs x 4th stage larvae**	catalytic activity	231	90.89
	binding	119	39.71
	structural molecule activity	27	15.24
	transporter activity	18	6.94
**Adults x 1st stage larvae**	catalytic activity	111	35.84
	binding	50	22.39
**Adults x 2nd stage larvae**	catalytic activity	148	50.15
	binding	64	24.12
	structural molecule activity	26	14.26
**Adults x 3rd stage larvae**	catalytic activity	132	49.43
	binding	47	20.81
**Adults x 4th stage larvae**	catalytic activity	122	47.8
	binding	39	17.47
