# Supplementary material for: De novo transcriptome assembly and analysis to identify potential gene targets for RNAi-mediated control of the tomato leafminer (Tuta absoluta)
Source: BMC Genomics. 2015 Aug 26;16(1):635. doi: 10.1186/s12864-015-1841-5 (PMC4550053; doi:10.1186/s12864-015-1841-5)
Supplement: Additional file 3: Table S3. — Cellular component category annotation by Gene Ontology for differentially expressed transcripts (DET) for pair-wise comparisons between developmental stages of Tuta absoluta by Blast2Go. (PDF 8 kb) [file 12864_2015_1841_MOESM3_ESM.pdf]

Table S3. Cellular component category annotation by Gene Ontology for differentially expressed transcripts (DET) for pair-wise comparisons between developmental stages of *Tuta absoluta* by Blast2Go.

Compared samples	GO Term	#Seq	Score
**Adults x Eggs**	macromolecular complex	55	23.16
	membrane	32	17.39
	cell	93	12.53
	organelle	83	11.72
**Eggs x 1st stage larvae**	membrane	32	20.35
	macromolecular complex	43	15.48
	extracellular region	14	12.72
	cell	71	6.04
	organelle	63	5.95
**Eggs x 2nd stage larvae**	membrane	49	28.28
	macromolecular complex	52	18.85
	extracellular region	20	18.08
	organelle	77	8.75
	cell	87	8.02
**Eggs x 3rd stage larvae**	membrane	44	26.94
	extracellular region	28	26.08
	macromolecular complex	59	22.31
	organelle	82	10.82
	cell	91	8.97
**Eggs x 4th stage larvae**	extracellular region	31	27.8
	macromolecular complex	63	24.25
	membrane	40	24.1
	organelle	82	10.6
	cell	91	9.73
**Adults x 1st stage larvae**	macromolecular complex	18	7.47
	cell	26	5.85
	organelle	21	4.63
**Adults x 2nd stage larvae**	membrane	17	10.24
	macromolecular complex	16	6.75
	cell	29	6.47
	organelle	23	5.12
**Adults x 3rd stage larvae**	extracellular region	16	15.36
	cell	12	1.96
**Adults x 4th stage larvae**	extracellular region	17	16.36
