# Supplementary material for: De novo transcriptome assembly and analysis to identify potential gene targets for RNAi-mediated control of the tomato leafminer (Tuta absoluta)
Source: BMC Genomics. 2015 Aug 26;16(1):635. doi: 10.1186/s12864-015-1841-5 (PMC4550053; doi:10.1186/s12864-015-1841-5)
Supplement: Additional file 4: Table S4. — Kegg-based metabolic pathway annotation for differentially expressed transcripts (DET) for pair-wise comparisons between developmental stages of Tuta absoluta by Blast2Go. (PDF 10 kb) [file 12864_2015_1841_MOESM4_ESM.pdf]

Table S4. Kegg-based metabolic pathway annotation for differentially expressed transcripts (DET) for pair-wise comparisons between developmental stages of *Tuta absoluta* by Blast2Go.

Compared samples	Kegg Pathways	Total transcripts
**Adults x Eggs**	Galactose metabolism	7
	Starch and sucrose metabolism	7
	Biosynthesis of antibiotics	4
	Glyoxylate and dicarboxylate metabolism	4
	Arginine and proline metabolism	3
	Alanine, aspartate and glutamate metabolism	3
	Fatty acid biosynthesis	3
	Nitrogen metabolism	3
	Glycolysis / Gluconeogenesis	3
	Pyruvate metabolism	2
	Tyrosine metabolism	2
	Tryptophan metabolism	2
**Eggs x 1st stage larvae**	Steroid hormone biosynthesis	2
	Tryptophan metabolism	2
**Eggs x 2nd stage larvae**	Drug metabolism - cytochrome P450	3
	Metabolism of xenobiotics by cytochrome P450	3
	Glutathione metabolism	3
	Tryptophan metabolism	3
	Steroid hormone biosynthesis	2
	Fatty acid biosynthesis	2
	Glycine, serine and threonine metabolism	2
**Eggs x 3rd stage larvae**	Starch and sucrose metabolism	6
	Drug metabolism - cytochrome P450	5
	Metabolism of xenobiotics by cytochrome P450	5
	Glutathione metabolism	5
	Galactose metabolism	4
	Biosynthesis of antibiotics	3
	Tryptophan metabolism	3
	Ubiquinone and other terpenoid-quinone biosynthesis	2
	Phenylalanine metabolism	2
	Steroid hormone biosynthesis	2
	Fatty acid biosynthesis	2
	Glycine, serine and threonine metabolism	2
	Glyoxylate and dicarboxylate metabolism	2
	Tyrosine metabolism	2
**Eggs x 4th stage larvae**	Fatty acid biosynthesis	7
	Starch and sucrose metabolism	6
	Galactose metabolism	4
	Drug metabolism - cytochrome P450	4
	Metabolism of xenobiotics by cytochrome P450	4
	Biosynthesis of antibiotics	4
	Glutathione metabolism	4
	Glyoxylate and dicarboxylate metabolism	4

	Arginine and proline metabolism	3
	Alanine, aspartate and glutamate metabolism	3
	Pyruvate metabolism	3
	Nitrogen metabolism	3
	Glycolysis / Gluconeogenesis	3
	Tryptophan metabolism	3
	Steroid hormone biosynthesis	2
	Purine metabolism	2
	Glycine, serine and threonine metabolism	2
**Adults x 1st stage larvae**	Nitrogen metabolism	3
	Arginine and proline metabolism	3
	Alanine, aspartate and glutamate metabolism	3
	Starch and sucrose metabolism	3
	Galactose metabolism	3
	Glyoxylate and dicarboxylate metabolism	3
	Steroid hormone biosynthesis	2
	Fatty acid biosynthesis	2
**Adults x 2nd stage larvae**	Starch and sucrose metabolism	6
	Galactose metabolism	6
	Nitrogen metabolism	2
	Arginine and proline metabolism	2
	Alanine, aspartate and glutamate metabolism	2
	Steroid hormone biosynthesis	2
	Glyoxylate and dicarboxylate metabolism	2
**Adults x 3rd stage larvae**	Starch and sucrose metabolism	5
	Galactose metabolism	5
	Steroid hormone biosynthesis	2
**Adults x 4th stage larvae**	Fatty acid biosynthesis	5
	Starch and sucrose metabolism	2
	Steroid hormone biosynthesis	2
	Galactose metabolism	2
