# Supplementary material for: De novo transcriptome assembly and analysis to identify potential gene targets for RNAi-mediated control of the tomato leafminer (Tuta absoluta)
Source: BMC Genomics. 2015 Aug 26;16(1):635. doi: 10.1186/s12864-015-1841-5 (PMC4550053; doi:10.1186/s12864-015-1841-5)
Supplement: Additional file 5: Table S5. — Primer sequences of 23 genes differentially expressed between the developmental stages with expected product size, and three gene references used in RT-qPCR to validate differences in read counts among libraries. Annotation is derived from Blast2Go or manual. (PDF 53 kb) [file 12864_2015_1841_MOESM5_ESM.pdf]

Table S5. Primer sequences of 23 genes differentially expressed between the developmental stages with expected product size, and three gene references used in RT-qPCR to validate differences in read counts among libraries. Annotation is derived from Blast2Go or manual.

Contig	Foward	Reverse	Size	Annotation
**2406**	GACCAAGGACGGCAAGTTCA	GTAGTTCCAGGCGGTTTGCT	139	sericotropin precursor
**6681**	TTCTGCCTATGCGGGAGGAG	GACGACGAGTGCCGATCTTG	119	fibroin heavy chain [*Ephestia kuehniella*]
**10806**	GAATCCAACTGCGGAAAG	TTGTCACCTCCGTTGTTT	161	insect intestinal mucin precursor
**11301**	TGAGAGTGAGAGCGACAGTA	GCCACAAACGGAGAACTTTC	189	glutathione s-transferase epsilon 2
**12524**	CGTACTCGTATCTCATCATATCAC	CGTTCACTAGTTGCGACTC	114	ABC transporter F family member 4-like [*Bombyx mori*]
**12828**	TCTCCATCATCTTGAACTGT	AGAAGGAATGCCAGGAAT	174	muscular protein 20
**13135**	GCATAGGTCTGCATCGCTGATAA	CGAACGTGCTCGGTCCAA	119	JH-inducible protein [*Danaus plexippus*]
**16411**	GCTGTGGGAGCATGTGGTT	GTCAACCTGGCTTTCTCTTTGG	171	uncharacterized protein LOC101735588 [*Bombyx mori*]
**16428**	GTTGAAGACGGTGTTGGGGG	GAAGGCACCGAATGGCAAGA	147	Predicted: mucin-2-like *Bombyx mori*
**17745**	TCGGAGCGCATCTTGGAAAC	CACCAAAGATAGGGCCAGCG	180	lipase-1
**20172**	TGGCAAGGGAATGAATGGAG	TTGGCTCTGGCTGTTTGGTT	133	fibroin light chain [*Corcyra cephalonica*]
**21584**	GCACGCATTTGTTCGCTCTG	GAGATGCGAGTCCCTTTGCC	137	Predicted: chymotrypsin inhibitor-like [*Apis mellifera*]
**23824**	ACGGTGCTGGCAAGGTGT	CCACTGCTTGAGGGTCTTCC	185	estradiol 17-beta-dehydrogenase isoforma a
**26572**	GCCATAATTCAAAGTGGACCGAT	GGTCGCTGTTTCTACAACTTGAA	174	carboxylesterase ae17 [*Bombyx mori*]

**36206**	CCATTCAAGTTGCAGAGA	GGTAGGGAGAATTGTTGTT	105	No similarity
**36279**	TCCTCACTGTCCCGTCCAAG	GAAGAGAACCAACCACCAGCA	101	FMRFamide [*Helicoverpa armigera*]
**38086**	ACAACCGGCACAACAATCCA	CCCGTTTTCCCAAGGGGATG	189	zinc carboxypeptidase
**50455**	CGCACAGACATTCGGAGCTT	AATGGCAGCGAAGGTTCTGG	111	peritrophin 1 [*Mamestra configurata*]
**55173**	GGTCGTCGTAACAATACCAGCA	GCGAACAAGGGCACCATC	141	alkaline phosphatase
**58512**	GGGCATGTGGAACGAGTACG	GAGGAGCAGACCTGGGTGAA	141	sarcoplasmic calcium-binding protein 2 cg14904-pa
**75835**	TTGTCGGATTCAAGACGATTCTC	CGTGCGTTTGTCTGGAAGT	128	thrombospondin type-1 domain-containing protein 7A-like [*Bombyx mori*
**77615**	CAGAACGCTGAAGGAGTC	CACCTTCTACAACGCCAAC	100	No similarity
**81147**	CCTACTCCGCTTACCGTCAA	ACTCTCAAGAAGAAGACTCCACTT	165	serotonin transporter
RpL 5	CAGTCGTCGAGCCAGCAACA	TCCCGCATTGAAGGAGACCA	129	-
Rpl23A	TTGACGCCATAACGTGGCAGT	CGCAAACGCCTGACTGTTCA	170	-
rRNA	TATGTTGTGAGGCGACGATG	GATCCACCGTCCAGGGTAAT	155	-
