# Supplementary material for: De novo transcriptome assembly and analysis to identify potential gene targets for RNAi-mediated control of the tomato leafminer (Tuta absoluta)
Source: BMC Genomics. 2015 Aug 26;16(1):635. doi: 10.1186/s12864-015-1841-5 (PMC4550053; doi:10.1186/s12864-015-1841-5)
Supplement: Additional file 6: Figure S1. — Comparison of relative gene expression based on RT-qPCR or RNAseq among developmental stages. Based on the RNAseq data, 23 contigs with significant differential expression between developmental stages (egg, larval and adults) were chosen to be validated by RT-qPCR. Relative expression of 18 genes (2406, 6681, 10806, 11301, 12828, 13135, 16411, 16428, 17745, 20172, 21584, 23824, 26572, 36206, 36279, 38086, 50455, and 55173; Additional File 5: Table S5) based on RT-qPCR is represented by whiskers-box plots with standard deviations, and RNAseq data is represented by fold-differences. Whisker-box plots display average values from three biological replicates, and the box contain 50 % of the variation among samples, while the remaining 50 % are divided between the upper quartile (25 %) and the lower quartile (25 %), represented by error bars (whiskers). (PDF 66 kb) [file 12864_2015_1841_MOESM6_ESM.pdf]

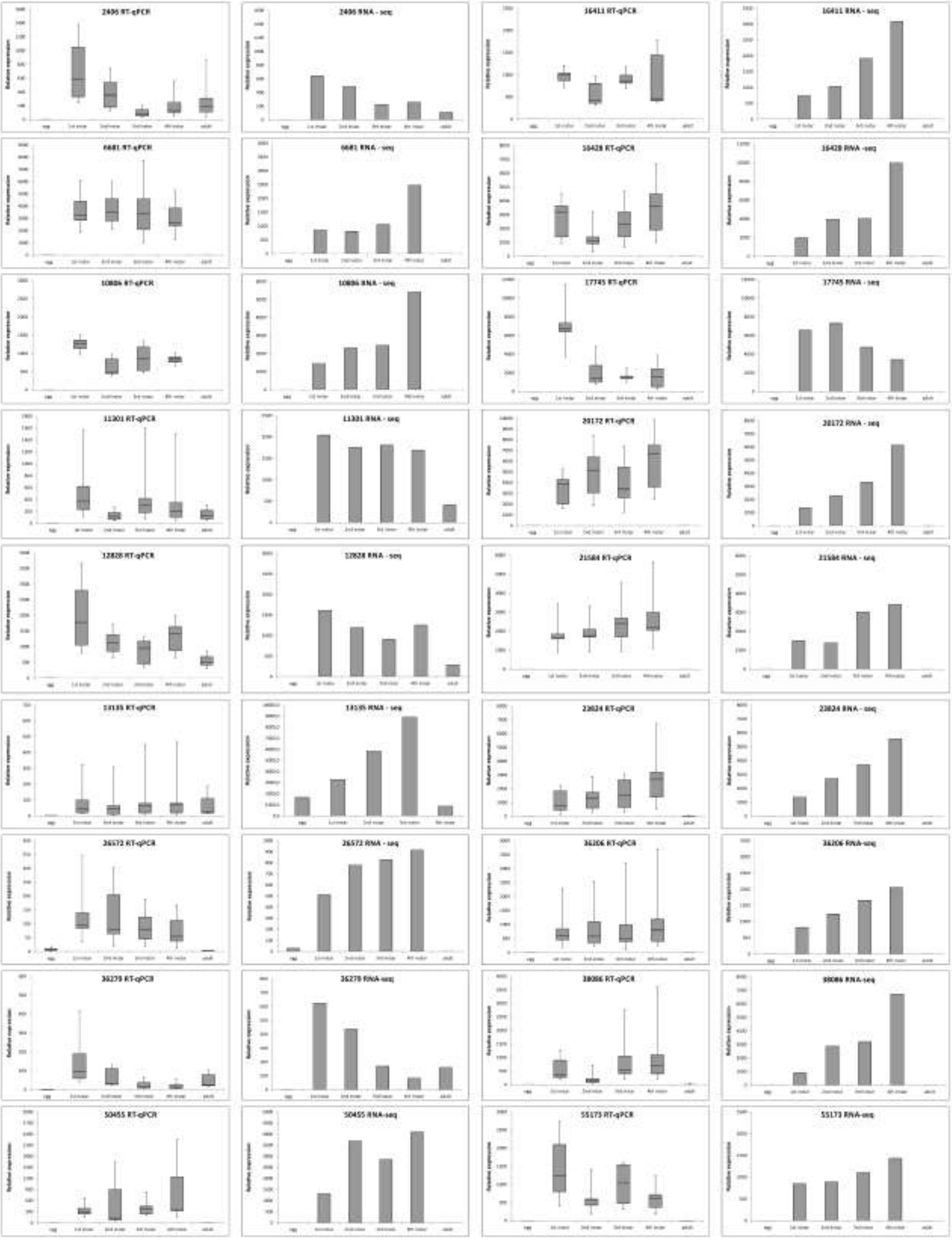

Figure S1. Comparison of relative gene expression based on RT-qPCR or RNAseq among developmental stages. Based on the RNAseq data, 23 contigs with significant differential expression between developmental stages (egg, larval and adults) were chosen to be validated by RT-qPCR.

Relative expression of 18 genes (2406, 6681, 10806, 11301, 12828, 13135, 16411, 16428, 17745, 20172, 21584, 23824, 26572, 36206, 36279, 38086, 50455, and 55173; Supplementary Table 5) based on RT-qPCR is represented by whiskers-box plots with standard deviations, and RNAseq data is represented by fold-differences. Whisker-box plots display average values from three biological replicates, and the box contain 50% of the variation among samples, while the remaining 50% are divided between the upper quartile (25%) and the lower quartile (25%), represented by error bars (whiskers).
