# Supplementary material for: De novo transcriptome assembly and analysis to identify potential gene targets for RNAi-mediated control of the tomato leafminer (Tuta absoluta)
Source: BMC Genomics. 2015 Aug 26;16(1):635. doi: 10.1186/s12864-015-1841-5 (PMC4550053; doi:10.1186/s12864-015-1841-5)
Supplement: Additional file 7: Table S6. — List of genes associated with RNAi mechanism identified in the T. absoluta assembled transcriptome at E-value < e−30 using homologues, particularly from Bombyx mori. FPKM values for each transcript normalized per library is presented. Red rows represent undetected genes within the T. absoluta transcriptome for all sequenced stages, while grey rows are those that did not reach the set E-value < e−30. (DOCX 66 kb) [file 12864_2015_1841_MOESM7_ESM.docx]

**Table S6**. List of genes associated with RNAi mechanism identified in the *T. absoluta* assembled transcriptome at *E-*value < e^-30^ using homologues, particularly from *Bombyx mori*. FPKM values for each transcript normalized per library is presented. Red rows represent undetected genes within the *T. absoluta* transcriptome for all sequenced stages, while grey rows are those that did not reach the set *E-*value < e^-30^.

| **GENE** | **BAIT** | **TRANSCRIPTS** | ***E* VALUE** | **FPKM values normalized per library** | | | | | |  |
| --- | --- | --- | --- | --- | --- | --- | --- | --- | --- | --- |
|  |  |  |  | **eggs** | **1^st^ stage** | **2^nd^ stage** | **3^rd^ stage** | **4^th^ stage** | **adult** |  |
| **dsRNA uptake** | | | | | | | | | |  |
| *sid-1-1* | >gi\|164448645:280-2625 *Bombyx mori* sid-1-related gene1 (*Sir-1*) | ta_transcript74094  ta_transcript44271  ta_transcript44268  ta_transcript44264 | 1e^-127^  2e^-33^  8e^-32^  8e^-31^ | 133.5112  7.4324  11.9329  9.1282 | 34.5310  19.7815  42.1216  56.0653 | 67.3349  19.1107  53.7831  115.9250 | 144.7193  38.9895  102.9476  226.8508 | 158.6020  66.6620  79.7173  216.4624 | 47.9097  19.9142  45.6753  42.6097 |  |
| *sid-1-2* | >gi\|512925015:122-4090 : *Bombyx mori* sid-1-related gene2 (*Sir-2*) | ta_transcript74094  ta_transcript84451  ta_transcript84449  ta_transcript84450  ta_transcript84448  ta_transcript84453  ta_transcript44271  ta_transcript44268  ta_transcript44264  ta_transcript44270 | 0.0  0.0  0.0  0.0  0.0  3e^-150^  2e^-41^  9e^-40^  7e^-39^  5e^-34^ | 133.5112  79.1241  40.4764  52.1392  47.5696  78.6533  7.4324  11.9329  9.1282  19.9191 | 34.5310  8.9703  1.1404  6.3492  3.1416  11.5020  19.7815  42.1216  56.0653  21.1455 | 67.3349  29.8217  11.1396  12.8748  10.5948  4.6300  19.1107  53.7831  115.9250  23.7117 | 144.7193  25.4807  12.7371  18.7715  14.7924  14.8231  38.9895  102.9476  226.8508  35.7241 | 158.6020  24.9657  8.6596  11.8214  12.2339  10.8531  158.6020  79.7173  216.4624  50.2884 | 47.9097  9.3719  14.5691  8.9028  6.1999  14.0605  47.9097  45.6753  42.6097  24.0026 |  |
| *sid1-3* | >gi\|164448647:159-2792 *Bombyx mori* sid-1-related gene3 (*Sir-3*) | ta_transcript74094  ta_transcript44271  ta_transcript44268  ta_transcript44264  ta_transcript44270  ta_transcript44272 | 1e^-111^  2e^-64^  8e^-62^  8e^-60^  7e^-53^  4e^-32^ | 133.5112  7.4324  11.9329  9.1282  19.9191  9.7787 | 34.5310  19.7815  42.1216  56.0653  21.1455  2.8600 | 67.3349  19.1107  53.7831  115.9250  23.7117  14.9665 | 144.7193  38.9895  102.9476  226.8508  35.7241  16.9114 | 158.6020  66.6620  79.7173  216.4624  50.2884  5.0118 | 47.9097  19.9142  45.6753  42.6097  24.0026  88.2271 |  |
| *Sid-2* | >gi\|392897213\|ref\|NM_067422.6\| *Caenorhabditis elegans* Protein SID-2 (sid-2) | No hits found | - | - | - | - | - | - | - |  |
| **dsRNA cleavage** | | | | | | | | | |  |
| *Dcr-1* | >gi\|442620537\|ref\|NM_079729.3\| *Drosophila melanogaster* Dicer-1 (Dcr-1) | ta_transcript89347  ta_transcript89348 | 1e^-38^  2e^-38^ | 55.19058  58.57380 | 12.27688  10.96405 | 12.84901  16.25625 | 23.30168  23.18604 | 10.35837  5.20352 | 6.16327  10.24920 |  |
| *Dcr-2* | >gi\|300669732\|dbj\|AB566386.1\| *Bombyx mori* Dicer-2 mRNA for DICER-2 | ta_transcript86357  ta_transcript86358  ta_transcript86359 | 4e^-79^  3e^-77^  2e^-36^ | 58.34365  48.89353  30.92611 | 30.49739  24.83981  6.48113 | 28.92626  24.83333  18.26237 | 224.16310  211.54930  141.00967 | 123.02902  118.89082  72.51191 | 67.70883  67.23480  21.43800 |  |
| *drosha* | >gi\|665399372\|ref\|NM_058088.4\| *Drosophila melanogaster* drosha (drosha) | ta_transcript89214 | 0.0 | 195.83069 | 112.02077 | 119.55819 | 82.68884 | 80.69288 | 97.05990 |  |
| **dsRNA binding** | | | | | | | | | |  |
| *loquacious* | >gi\|304307736\|ref\|NM_001195079.1\| *Bombyx mori* loquacious (Loqs) | ta_transcript67781  ta_transcript67771  ta_transcript67782  ta_transcript67778  ta_transcript67779  ta_transcript67775 | 0.0  0.0  2e^-170^  7e^-167^  8e^-43^  3e^-41^ | 565.67736  411.14363  445.79294  355.00437  448.09923  356.28820 | 256.65933  287.67806  199.79814  295.71827  212.75449  284.72493 | 320.36259  337.80207  285.60312  317.34140  277.23513  309.49185 | 340.01705  293.28485  314.89910  260.17231  292.96496  250.37313 | 264.20331  218.77537  232.33978  247.28555  202.01667  196.52827 | 378.89840  250.82765  336.11988  210.12994  351.45082  220.16053 |  |
| *R2D2* | >gi\|300669730\|dbj\|AB566385.1\| *Bombyx mori* R2D2 mRNA for R2D2 | ta_transcript64766  ta_transcript64768  ta_transcript64758  ta_transcript64763  ta_transcript64759  ta_transcript64764  ta_transcript64762  ta_transcript64751  ta_transcript64749  ta_transcript64767  ta_transcript64769  ta_transcript64761  ta_transcript64760  ta_transcript64765  ta_transcript64753  ta_transcript64755  ta_transcript64752  ta_transcript64750  ta_transcript64747  ta_transcript64757  ta_transcript64756  ta_transcript64754  ta_transcript64748 | 4e^-87^  6e^-87^  7e^-87^  9e^-87^  1e^-86^  1e^-86^  2e^-86^  2e^-84^  2e^-84^  3e^-84^  4e^-84^  4e^-84^  6e^-84^  7e^-84^  2e^-83^  6e^-82^  7e^-82^  7e^-82^  4e^-81^  6e^-81^  6e^-81^  8e^-81^  1e^-78^ | 9.5526467  12.013070  14.776712  17.594145  5.2431284  8.333268-  5.7286871  124.25071  120.21981  6.66730  7.2982542  15.254050  6.8147260  14.563356  129.16541  118.93890  135.07633  130.28869  128.08503  123.91047  127.48773  115.91317  118.62020 | 3.37195  10.54054  13.23554  18.33789  6.31433  5.24950  8.47055  11.09389  9.08009  8.87256  7.82669  13.38425  1.26836  11.92634  13.09510  11.59552  15.42868  12.01893  12.50552  12.99870  10.24792  9.41400  13.51331 | 12.70473  7.35450  24.40651  26.04692  13.21718  10.30150  6.81946  30.14757  23.45481  10.71466  9.68078  20.67846  9.95599  17.33630  23.72869  23.62166  22.07532  24.34652  28.59290  19.30957  27.14206  27.50550  20.60846 | 22.59696  19.39052  20.49726  40.66225  12.44563  14.22689  7.06351  40.27986  34.65161  17.48794  18.23133  23.24000  10.62478  31.34264  30.62605  39.58801  33.48965  37.44465  39.24806  32.85225  34.00816  30.53865  35.95715 | 7.09066  33.24753  27.61126  28.69162  25.44948  29.89682  23.97792  30.35449  39.60974  17.93993  23.68988  54.72613  16.66965  27.86569  34.59230  38.82486  36.27701  31.59227  34.38842  27.55456  36.28262  35.39926  26.38653 | 19.54304  11.16708  28.86937  25.10117  11.80528  21.46915  4.26369  34.75053  30.52095  22.33021  17.29330  19.06531  13.63510  29.73000  27.77133  35.03482  32.49681  31.35011  34.71079  37.62951  41.25162  24.56727  36.99445 |  |
| *C3PO* | >gi\|161579134\|gb\|EU273921.1\| *Tribolium* *castaneum* C3PO protein (C3PO) | Low score | - | - | - | - | - | - | - |  |
| *Rde-4* | >gi\|21912829\|gb\|AY071926.1\| *Caenorhabditis elegans* RNA interference promoting factor (rde-4) | Low score | - | - | - | - | - | - | - |  |
| *pasha* | >gi\|442622090\|ref\|NM_001276221.1\| *Drosophila melanogaster* partner of drosha (pasha), transcript variant | Low score | - | - | - | - | - | - | - |  |
| **Endonuclease activity** | | | | | | | | | |  |
| *Ago-1* | >gi\|156255205\|ref\|NM_001102461.1\| *Bombyx mori* argonaute 1 (Ago1) | ta_transcript56183  ta_transcript56182  ta_transcript51551  ta_transcript44491 | 2e^-80^  3e^-80^  2e^-43^  6e^-37^ | 212.96905  220.08097  1706.44174  91.77477 | 84.93814  103.84509  356.33502  8.73918 | 95.93407  94.46068  548.68874  28.96373 | 96.26319  103.37032  604.32862  31.57924 | 58.91677  51.90539  603.40934  10.20947 | 57.23433  49.18873  1096.71439  39.48538 |  |
| *Ago-2* | >gi\|166706853\|ref\|NM_001043530.2\| *Bombyx mori* argonaute 2 (Ago2) | ta_transcript85453  ta_transcript85452  ta_transcript51551 | 6e^-105^  6e^-105^  2e^-33^ | 192.37287  225.32124  1706.44174 | 104.04914  134.79985  356.33502 | 135.55669  160.36461  548.68874 | 209.81859  197.89023  604.32862 | 154.96095  186.35895  603.40934 | 119.05440  158.49478  1096.71439 |  |
| *Ago-3* | >gi\|166706857\|ref\|NM_001104597.2\| *Bombyx mori* argonaute 3 (Ago3) | ta_transcript51551  ta_transcript44491 | 1e^-155^  4e^-141^ | 1706.44174  91.77477 | 356.33502  8.73918 | 548.68874  28.96373 | 604.32862  31.57924 | 603.40934  10.20947 | 1096.71439  39.48538 |  |
| *Alg-1* | >gi\|577028025\|gb\|KF579958.1\| *Caenorhabditis japonica* strain VX0158 ALG-1 (alg-1) gene | No hits found | - | - | - | - | - | - | - |  |
| *Alg-2* | >gi\|114051845\|ref\|NM_001046722.1\| *Bombyx mori* apoptosis-linked protein 2 (Alg-2) | ta_transcript79570  ta_transcript79596  ta_transcript79595  ta_transcript79592  ta_transcript79591 | 2e^-47^  5e^-32^  2e^-31^  2e^-31^  2e^-31^ | 59.71996  71.85163  94.48618  66.95559  64.43721 | 54.69789  51.44992  83.09895  75.60888  80.38135 | 80.53856  92.52685  101.94204  92.64282  86.05387 | 104.90363  138.54399  136.26721  116.31308  104.00928 | 53.09366  67.30170  127.71542  51.70503  58.06329 | 39.35129  52.15981  101.23992  45.97023  57.35919 |  |
| *Rde-1* | >gi\|392920128\|ref\|NM_171525.4\| *Caenorhabditis elegans* Protein RDE-1 (rde-1) | Low score | - | - | - | - | - | - | - |  |
| *Ergo-1* | >gi\|392918268\|ref\|NM_070961.7\| *Caenorhabditis elegans* Protein ERGO-1 (ergo1) | Low score | - | - | - | - | - | - | - |  |
| *PIWI* | >gi\|157674347\|gb\|EU143547.1\| *Bombyx* *mori* PIWI | ta_transcript51551  ta_transcript44491 | 0.0  2e^-61^ | 1706.44174  91.77477 | 356.33502  8.7391778 | 548.68874  28.963731 | 604.32862  31.579237 | 603.40934  10.20947 | 1096.71439  39.48538 |  |
| *Sago-1* | >gi\|392919217\|ref\|NM_072209.3\| *Caenorhabditis elegans* Protein SAGO-1 (sago-1) | Low score | - | - | - | - | - | - | - |  |
| *Sago-2* | >gi\|392884665\|ref\|NM_058357.3\| *Caenorhabditis elegans* Protein SAGO-2, isoform a (sago-2) | Low score | - | - | - | - | - | - | - |  |
| *PPW-1* | >gi\|392885315\|ref\|NM_170850.3\| *Caenorhabditis elegans* Protein PPW-1, isoform b (ppw-1) | Low score | - | - | - | - | - | - | - |  |
| *PPW-2* | >gi\|392885541\|ref\|NM_059134.5\| *Caenorhabditis elegans* Protein PPW-2 (ppw-2) | Low score | - | - | - | - | - | - | - |  |
| *Aub* | >gi\|429892795\|gb\|KC116214.1\| *Drosophila melanogaster* isolate 3893 aubergine (Aub) gene | ta_transcript51551  ta_transcript44491 | 3e^-90^  5e^-51^ | 1706.44174  91.77477 | 356.33502  8.7391778 | 548.68874  28.963731 | 604.32862  31.579237 | 603.40934  10.20947 | 1096.71439  39.48538 |  |
| *Aubergine* | >gi\|166706855\|ref\|NM_001104596.2\| *Bombyx mori* aubergine protein (Aubergine) | ta_transcript51551  ta_transcript44491 | 0.0  3e^-62^ | 1706.44174  91.77477 | 356.33502  8.7391778 | 548.68874  28.963731 | 604.32862  31.579237 | 603.40934  10.20947 | 1096.71439  39.48538 |  |
| *armitage* | >gi\|665409862\|ref\|NM_139559.4\| *Drosophila melanogaster* armitage (armi), transcript variant D | Low score | - | - | - | - | - | - | - |  |
| *Asp spindleE* | >gi\|665394979\|ref\|NM_079764.3\| *Drosophila melanogaster* abnormal spindle (asp), mRNA | ta_transcript82361 | 2e^-141^ | 1321.21768 | 22.55203 | 54.48922 | 82.29668 | 70.45732 | 199.73362 |  |
| *Spindle E* | >gi\|562758882\|gb\|GABY01018532.1\| TSA: *Anthonomus grandis* A_grandis_454_rep_c7409 mRNA sequence | Low score | - | - | - | - | - | - | - |  |
| *Rm62* | >gi\|665393316\|ref\|NM_169121.2\| *Drosophila melanogaster* Rm62 (Rm62), transcript variant F, mRNA | ta_transcript16727  ta_transcript71371  ta_transcript47090  ta_transcript35263  ta_transcript35262  ta_transcript47089  ta_transcript65883  ta_transcript35264  ta_transcript16729  ta_transcript20008  ta_transcript20007 | 0.0  7e^-50^  1e^-49^  1e^-49^  1e^-49^  2e^-49^  3e^-48^  4e^-36^  5e^-34^  9e^-33^  2e^-32^ | 2304.95943  468.37030  133.24814  1044.53499  1343.72072  127.56607  1591.00484  1921.33406  214.93400  193.61839  243.07562 | 627.34327  375.24506  178.87765  594.95780  629.94778  159.22350  1420.6705  656.04166  91.10176  73.87433  120.22641 | 643.83556  606.13950  97.19650  789.49136  1005.63931  107.24591  3004.1631  1116.01005  68.36221  155.26264  191.56088 | 727.38780  538.66349  115.96338  657.28416  728.78614  103.05694  2507.78669  1195.92291  130.43713  147.97490  143.83126 | 427.74399  223.87066  106.33544  666.49043  805.69501  116.02696  1216.62667  783.14385  69.27915  98.93276  102.71970 | 540.37109  423.37046  298.91517  859.22281  883.94290  292.28092  1813.13449  842.38757  78.73466  516.52960  539.57286 | |

| **Amplification** | | | | | | | | | |
| --- | --- | --- | --- | --- | --- | --- | --- | --- | --- |
| *Ego-1* | >gi\|392886220\|ref\|NM_059731.4\| *Caenorhabditis elegans* Protein EGO-1 (ego-1) mRNA, complete cds | No hits found | - | - | - | - | - | - | - |
| *RRF1* | >gi\|392886216:1414-3303 *Caenorhabditis elegans* Protein RRF-1, isoform a | No hits found | - | - | - | - | - | - | - |
| *RRF2* | >gi\|392887358:1372-3231 *Caenorhabditis elegans* Protein RRF-2 (rrf-2) mRNA, complete cds | No hits found | - | - | - | - | - | - | - |
| *RRF3* | >gi\|392890784:2059-3819 *Caenorhabditis elegan*s Protein RRF-3 (rrf-3) mRNA, complete cds | No hits found | - | - | - | - | - | - | - |
| Rdrp (Dwil\GK16260) | >gi\|195438743\|ref\|XM_002067256.1\| *Drosophila willistoni* GK16260 (Dwil\GK16260)(Rdrp), mRNA | ta_transcript24699  ta_transcript13924 | 3e^-34^  1e^-32^ | 38.08362  254.47810 | 683.16006  1689.61464 | 1056.95169  1857.82698 | 3066.25515  4969.15141 | 4220.36549  7368.82931 | 977.59521  4094.51441 |
| **Degradation** | | | | | | | | | |
| *Snp* | >gi\|665402677\|ref\|NM_166503.3\| *Drosophila melanogaster* snipper (Snp), transcript variant G, mRNA | No hits found | - | - | - | - | - | - | - |
| *Eri-1* | >gi\|392898168\|ref\|NM_171245.4\| *Caenorhabditis elegans* Protein ERI-1, isoform a (eri-1) mRNA, complete cds | No hits found | - | - | - | - | - | - | - |
| *ERI-5* | >gi\|392898456\|ref\|NM_067798.5\| *Caenorhabditis elegans* Protein ERI-5 (eri-5) mRNA, complete cds | Low score | - | - | - | - | - | - | - |
| **Others** | | | | | | | | | |
| *rsd-2* | >gi\|392901601\|ref\|NM_001268821.1\| *Caenorhabditis elegans* Protein RSD-2, isoform c (rsd-2) mRNA, complete cds | Low score | - | - | - | - | - | - | - |
| *rsd-3* | >gi\|392927450\|ref\|NM_077572.5\| *Caenorhabditis elegans* Protein RSD-3 (rsd-3) mRNA, complete cds | No hits found | - | - | - | - | - | - | - |
| *rsd-6* | >gi\|392886390\|ref\|NM_059868.6\| *Caenorhabditis elegans* Protein RSD-6 (rsd-6) mRNA, complete cds | Low score | - | - | - | - | - | - | - |
| *tudor sn gene* | >gi\|304307738\|ref\|NM_001195080.1\| *Bombyx mori* tudor staphylococcus/micrococcal nuclease (Tudor-SN), mRNA | ta_transcript15886 | 3e^-99^ | 1201.48713 | 974.92558 | 2397.91876 | 3089.83192 | 2461.80687 | 861.58357 |
| *Mut-7* | >gi\|512926474\|ref\|XM_004931038.1\| PREDICTED: *Bombyx mori* probable exonuclease mut-7 homolog (LOC101745346), mRNA | Low score | - | - | - | - | - | - | - |
