# Supplementary material for: De novo transcriptome assembly and analysis to identify potential gene targets for RNAi-mediated control of the tomato leafminer (Tuta absoluta)
Source: BMC Genomics. 2015 Aug 26;16(1):635. doi: 10.1186/s12864-015-1841-5 (PMC4550053; doi:10.1186/s12864-015-1841-5)
Supplement: Additional file 8: Table S7. — BlastX results with the list of genes associated with RNAi mechanism identified in the T. absoluta assembled transcriptome at E-value < e−30 using homologues, particularly from Bombyx mori. (PDF 263 kb) [file 12864_2015_1841_MOESM8_ESM.pdf]

Table S7. BlastX results with the list of genes associated with RNAi mechanism identified in the *T. absoluta* assembled transcriptome at *E*-value < e^{-30} using homologues, particularly from *Bombyx mori*.

```
BLASTX 2.2.29+

Reference: Stephen F. Altschul, Thomas L. Madden, Alejandro A.
Schaffer, Jinghui Zhang, Zheng Zhang, Webb Miller, and David J.
Lipman (1997), "Gapped BLAST and PSI-BLAST: a new generation of
protein database search programs", Nucleic Acids Res. 25:3389-3402.



Database: herai/heraiprot.txt
           93,476 sequences; 49,077,216 total letters

Query= gi|164448645:280-2625 Bombyx mori sid-1-related gene1 (Sir-1), mRNA

Length=2346
                                                                      Score     E
Sequences producing significant alignments:                          (Bits)  Value

  ta_transcript74094_1                                                  407   9e-127
  ta_transcript44271_1                                                  131   3e-33
  ta_transcript44268_1                                                  132   4e-32
  ta_transcript44264_1                                                  134   1e-31


> ta_transcript74094_1
Length=937

 Score =   407 bits (1045),  Expect = 9e-127, Method: Compositional matrix adjust.
 Identities = 215/425 (51%), Positives = 289/425 (68%), Gaps = 7/425 (2%)
 Frame = +1

Query  1078   RED-SAETDTQPILEAGAADESWSREHA--LTVGKLTRAPPDTLARRSDRYFWGALTLAV  1248
              RED S+ +DT+  +      +     +A  L V  L+R  P  L  RS  Y W  LT+AV
Sbjct  503    REDCSSPSDTESEISTVVEPDPDKTTYAGRLCVANLSRCRPRVLQARSKMYLWNILTVAV  562

Query  1249   VYALPVVQLLLTYQRMVFQTGDQDLCYYNFLCAHPLGTLSDFNHVFSNVGYVLLGAVFAG  1428
               Y LPV+QL++TYQR++ Q+G+QDLCY+NFLCAHPL  LSDFNHV+SN+GYV+LG++F
Sbjct  563    FYTLPVIQLVVTYQRLLNQSGNQDLCYFNFLCAHPLMVLSDFNHVYSNLGYVILGSLFLF  622

Query  1429   QVRFRQVKSRQRP---ENLGIPQHYGLLYSMGLALSMEGLLSACYHLCPNKMNFQFDSSF  1599
              QV  R       P    + LGIPQH+GLLY+MG+AL  EGLLSA YH+CPN MNFQFD+SF
Sbjct  623    QVWRRHSYHTIMPAHRKELGIPQHFGLLYAMGIALVSEGLLSAAYHVCPNSMNFQFDTSF  682

Query  1600   MYVIAVLVTLKLYQNRHSDIIPSAHSTFMILAVIMTIGLFGILHPSAGFAASFTLLHLGA  1779
              MYV +VL  +K+YQ+RH DI   AH+TF +LA+I+ IGL G+L+ +  F  FT+LHL
Sbjct  683    MYVTSVLCMVKIYQSRHPDINARAHATFGVLALIIFIGLVGVLNANFYFWIMFTVLHLLT  742

Query  1780   CLVLTLKIYYAGRFKMDRRVLLRAYAHVAARGWRSLLPAHPYRagllglanlanwslagY  1959
              CLV+T +IYY GRF++D  V+ RA   +  R   ++ P H  R  +L LANL+NW++A Y
Sbjct  743    CLVMTFQIYYLGRFRLDGGVVYRAARELIRRPLAAITPTHCGRCVMLLLANLSNWAIAAY  802

Query  1960   SVYSHHNTDLARQLLAILMGNAILYTMFYMVMKLVNRERILARTWMYCILAHVAWFLALR  2139
               V S H+ D A  LL +LM N  LYT+FY+VMKL++RE I   +W++  L +  WF +
Sbjct  803    GV-SQHSRDFASHLLLVLMSNLFLYTLFYIVMKLLHRETIGWYSWVFIALTYSVWFSSSY  861

Query  2140   LFLDSKTKWSETPAQSRQHNAPCSSLSFYDTHDLWHGVSAAALFLSFNMLLTMDDALRDT  2319
               +LD  T W+ +PA+SRQ N  CS L  YD+HD+WH +S+ A+F SFNM LT+DD L +
Sbjct  862    FYLDQSTNWALSPAESRQSNRQCSLLQLYDSHDIWHFMSSIAMFFSFNMYLTIDDGLLNV  921

Query  2320   PRDQI  2334
               RD+I
Sbjct  922    GRDEI  926


 Score =   223 bits (569),  Expect = 1e-60, Method: Compositional matrix adjust.
 Identities = 135/330 (41%), Positives = 181/330 (55%), Gaps = 48/330 (15%)
 Frame = +1

Query  91    FQYN-IYNYDTWINLQVNNTIEQILDFTEDSDKLLGFPTRVHVTTNSTLTSDHPLFITAT  267
             F YN  Y Y+     + V+    E I++F  D +K     P RV V ++       +PLFITA
Sbjct  83    FHYNNTYKYNVEYVVDVDKHTEYIMEFAGD-EKAYDTPARVTVASDFA-NRTYPLFITAR  140
```

```
Query  268  QQKGVSSWELPLVLQTDDYFLMLNDMGRTLCPH-------DAGSDIRRESPPTVQLTTSS  426
            QQKGV SW+LP+++QT       N++ RTLCPH       +   D+   + P V LT++S
Sbjct  141  QQKGVFSWQLPMLIQTPMSLEQFNNISRTLCPHNNLYNEDEESCDVTTINTPIVHLTSAS  200

Query  427  SANVSVDIKLKRVEDFYIELGKVNEVIVNPSSPRYYYFSFDQNPW---------------  561
               + V I ++ V DFYI   K   + V PS P+YY++ F QNP
Sbjct  201  PEPIRVTIVVESVLDFYIVTNKTTNMTVTPSQPKYYFYPFKQNPELDLHTQKIKKKYKCN  260

Query  562  -NVSHAAGGPLDG--------TQRYNY-NIPKSVILVIESDDEICATVSIQNNSCPVFDN  711
             + H     LD          ++Y++   PKSVI++IESDD+ICA VSIQN SCPVFDN
Sbjct  261  EELDHKELISLDRYFLKSNALKRQYSWLTRPKSVIVMIESDDDICAVVSIQNFSCPVFDN  320

Query  712  EREVKYKGYHLTMSSQGGITLTQAMFPSGFYVVLIVRQSDADCT--GASETE--------  861
            ER++ Y GY+LTM+ +GGITLTQ MFP GFY+V IV+ SD DC   G S+
Sbjct  321  ERDILYDGYYLTMTRRGGITLTQDMFPLGFYIVFIVKSSDDDCVMGGVSDVNVSRITVRW  380

Query  862  ---DAPKSFPAKRSKTFRLKIIATISYQEY  942
               A  +    R KTF  +II TISY+EY
Sbjct  381  QHGKATATSDDGRVKTFSFRIIETISYREY  410


> ta_transcript44271_1
Length=224

 Score =   131 bits (329),  Expect = 3e-33, Method: Compositional matrix adjust.
 Identities = 87/175 (50%), Positives = 105/175 (60%), Gaps = 2/175 (1%)
 Frame = -2

Query  1712  PIVMMTARIMKVECALGMMSECLFWYSLSVTSTAITYMNDESNWKFILLGQRW*QADRRP  1533
             P+ M TA   KV CA  +MS C  WY  ++ STA TYMN  SNWK   LGQ W* A+
Sbjct  20    PMAMSTASAAKVACARALMSGCRDWYIFTMLSTASTYMNAVSNWKLSRLGQMW*VAESSA  79

Query  1532  SMLRARPIEYSSP*CWGMPKFS--GRCLLLTCLKRTWPANTAPSST*PTLENTWLKSESV  1359
             + +   P   SSP*C GMP  S  G  L      +RT   N+APSS *P LENTWLKSE+
Sbjct  80    TTIMLAPSAVSSP*CAGMPYCSWRGLRLRRIRRRRTCSMNSAPSSK*PRLENTWLKSETP  139

Query  1358  PSGWAHRKL**HRSWSPVWNTMRWYVSSSCTTGSAYTTASVRAPQKYLSDLLASV  1194
             P G AHRKL**HRS  P    + W V+++C TGSA  T +V +  +YLS+   SV
Sbjct  140   PHGCAHRKL**HRSSEPDTFNISWKVNTNCMTGSA*NTDTVYSVCRYLSERADSV  194


> ta_transcript44268_1
Length=404

 Score =   132 bits (331),  Expect = 4e-32, Method: Compositional matrix adjust.
 Identities = 87/175 (50%), Positives = 105/175 (60%), Gaps = 2/175 (1%)
 Frame = -2

Query  1712  PIVMMTARIMKVECALGMMSECLFWYSLSVTSTAITYMNDESNWKFILLGQRW*QADRRP  1533
             P+ M TA   KV CA  +MS C  WY  ++ STA TYMN  SNWK   LGQ W* A+
Sbjct  20    PMAMSTASAAKVACARALMSGCRDWYIFTMLSTASTYMNAVSNWKLSRLGQMW*VAESSA  79

Query  1532  SMLRARPIEYSSP*CWGMPKFS--GRCLLLTCLKRTWPANTAPSST*PTLENTWLKSESV  1359
             + +   P   SSP*C GMP  S  G  L      +RT   N+APSS *P LENTWLKSE+
Sbjct  80    TTIMLAPSAVSSP*CAGMPYCSWRGLRLRRIRRRRTCSMNSAPSSK*PRLENTWLKSETP  139

Query  1358  PSGWAHRKL**HRSWSPVWNTMRWYVSSSCTTGSAYTTASVRAPQKYLSDLLASV  1194
             P G AHRKL**HRS  P    + W V+++C TGSA  T +V +  +YLS+   SV
Sbjct  140   PHGCAHRKL**HRSSEPDTFNISWKVNTNCMTGSA*NTDTVYSVCRYLSERADSV  194


> ta_transcript44264_1
Length=747

 Score =   134 bits (337),  Expect = 1e-31, Method: Compositional matrix adjust.
 Identities = 87/175 (50%), Positives = 105/175 (60%), Gaps = 2/175 (1%)
 Frame = -2

Query  1712  PIVMMTARIMKVECALGMMSECLFWYSLSVTSTAITYMNDESNWKFILLGQRW*QADRRP  1533
             P+ M TA   KV CA  +MS C  WY  ++ STA TYMN  SNWK   LGQ W* A+
Sbjct  20    PMAMSTASAAKVACARALMSGCRDWYIFTMLSTASTYMNAVSNWKLSRLGQMW*VAESSA  79

Query  1532  SMLRARPIEYSSP*CWGMPKFS--GRCLLLTCLKRTWPANTAPSST*PTLENTWLKSESV  1359
             + +   P   SSP*C GMP  S  G  L      +RT   N+APSS *P LENTWLKSE+
Sbjct  80    TTIMLAPSAVSSP*CAGMPYCSWRGLRLRRIRRRRTCSMNSAPSSK*PRLENTWLKSETP  139
```

```
Query  1358  PSGWAHRKL**HRSWSPVWNTMRWYVSSSCTTGSAYTTASVRAPQKYLSDLLASV  1194
             P G AHRKL**HRS  P    + W V+++C TGSA  T +V +  +YLS+   SV
Sbjct  140   PHGCAHRKL**HRSSEPDTFNISWKVNTNCMTGSA*NTDTVYSVCRYLSERADSV  194

***********************************************************************************************
*********************************************************************

Query= gi|512925015:122-4090 PREDICTED: Bombyx mori sid-1-related gene2 (Sir-2), mRNA

Length=3969
                                                                      Score     E
Sequences producing significant alignments:                          (Bits)  Value

  ta_transcript74094_1                                                 1033   0.0
  ta_transcript84451_1                                                  709   0.0
  ta_transcript84449_1                                                  714   0.0
  ta_transcript84450_1                                                  712   0.0
  ta_transcript84448_1                                                  713   0.0
  ta_transcript84453_1                                                  464   3e-150
  ta_transcript44271_1                                                  156   2e-41
  ta_transcript44268_1                                                  156   9e-40
  ta_transcript44264_1                                                  158   7e-39
  ta_transcript44270_1                                                  135   5e-34

> ta_transcript74094_1
Length=937

 Score =  1033 bits (2672),  Expect = 0.0, Method: Compositional matrix adjust.
 Identities = 519/821 (63%), Positives = 609/821 (74%), Gaps = 58/821 (7%)
 Frame = +1

Query  1651  YDVPARVTVACDSANRTYPLFVTARQQKGVFSWQLPMLVQTPVTTEQFTSISRTLCPHNN  1830
             YD PARVTVA D ANRTYPLF+TARQQKGVFSWQLPML+QTP++ EQF +ISRTLCPHNN
Sbjct  116   YDTPARVTVASDFANRTYPLFITARQQKGVFSWQLPMLIQTPMSLEQFNNISRTLCPHNN  175

Query  1831  MFDEDAEACDAPSLNTPIVHLTSSSSEQLKVTILVEKVQDFYIKINQTINITVSPSQPKY  2010
             +++ED E+CD  ++NTPIVHLTS+S E ++VTI+VE V DFYI  N+T N+TV+PSQPKY
Sbjct  176   LYNEDEESCDVTTINTPIVHLTSASPEPIRVTIVVESVLDFYIVTNKTTNMTVTPSQPKY  235

Query  2011  YFYPFKKGSGKTVDFDEQKMRKEYICGASGGRGHGEV--------QRNEVG--YGWLSRP  2160
             YFYPFK+     +D   QK++K+Y C       H E+        + N +   Y WL+RP
Sbjct  236   YFYPFKQN--PELDLHTQKIKKKYKCNEE--LDHKELISLDRYFLKSNALKRQYSWLTRP  291

Query  2161  ENVIFMIESDDELCAVVSIQNFSCPVFDNERDILYDGYYLTMTRRGGITLTQDTFPIGFY  2340
             ++VI MIESDD++CAVVSIQNFSCPVFDNERDILYDGYYLTMTRRGGITLTQD FP+GFY
Sbjct  292   KSVIVMIESDDDICAVVSIQNFSCPVFDNERDILYDGYYLTMTRRGGITLTQDMFPLGFY  351

Query  2341  IVFIVKTSDEDCKEPGTNGSVPAVARLLGWGDNIQVSSTEGRVKNFRFKIVETISYREYL  2520
             IVFIVK+SD+DC   G +    +   +         +S +GRVK F F+I+ETISYREYL
Sbjct  352   IVFIVKSSDDDCVMGGVSDVNVSRITVRWQHGKATATSDDGRVKTFSFRIIETISYREYL  411

Query  2521  IAAGATVLFYASFYLAFFVFVLYQSRKSTGVEHQDQET----------------------  2634
             IAAGA + F+ SFY  F + V+YQ  +  G+   D +T
Sbjct  412   IAAGAVLGFFMSFYAGFAIVVVYQ--RVQGLRRSDSDTLDSSEEGRSTPPTASYDDTATQ  469

Query  2635  -----IVRsshgsegqssgegpsLPRPT-DGSSVD---SQSDTESEYSTLDDVTTDKELY  2787
                  +V +   S   +S +     R   DG + +   S SDTESE ST+  V  D +
Sbjct  470   TTRRRVVNAMSASVESTSRQSDLQQRLVGDGDAREDCSSPSDTESEISTV--VEPDPDKT  527

Query  2788  RYGTKLCLADLSRCRARVLATRSNRYLWTVLTVSVFYTLPVLQLVVTYQRLLNQSGNQDL  2967
              Y  +LC+A+LSRCR RVL  RS   YLW +LTV+VFYTLPV+QLVVTYQRLLNQSGNQDL
Sbjct  528   TYAGRLCVANLSRCRPRVLQARSKMYLWNILTVAVFYTLPVIQLVVTYQRLLNQSGNQDL  587

Query  2968  CYFNFFCAHPLMMLSDFNHVFSNLGYVVLGALFLLQVWRRQRIMRNEPEEKKQKGIPQHF  3147
             CYFNF CAHPLM+LSDFNHV+SNLGYV+LG+LFL QVWRR       P  +K+ GIPQHF
Sbjct  588   CYFNFLCAHPLMVLSDFNHVYSNLGYVILGSLFLFQVWRRHSYHTIMPAHRKELGIPQHF  647

Query  3148  GLLYAMGVALISEGFLSAAYHVCPNSMNFQFDTSFMYVTSALCMVKIYQSRHPDINARAH  3327
             GLLYAMG+AL+SEG LSAAYHVCPNSMNFQFDTSFMYVTS LCMVKIYQSRHPDINARAH
Sbjct  648   GLLYAMGIALVSEGLLSAAYHVCPNSMNFQFDTSFMYVTSVLCMVKIYQSRHPDINARAH  707

Query  3328  ATFGVLALIIFIGLVGVLNANVYFWVAFTALHLLTCFFITFQIYYLGRFKLDMGWVRAAS  3507
             ATFGVLALIIFIGLVGVLNAN YFW+ FT LHLLTC  +TFQIYYLGRF+LD G V  A+
Sbjct  708   ATFGVLALIIFIGLVGVLNANFYFWIMFTVLHLLTCLVMTFQIYYLGRFRLDGGVVYRAA  767

Query  3508  RGAALRP---------SRGLMLLLANLINWGLAGYGVAQHSRDFASHLLLVLMTNLFLYT  3660
             R    RP          R +MLLLANL NW +A YGV+QHSRDFASHLLLVLM+NLFLYT
```

```
Sbjct  768   RELIRRPLAAITPTHCGRCVMLLLANLSNWAIAAYGVSQHSRDFASHLLLVLMSNLFLYT  827

Query  3661  LFYIVMKLLHRETITCYTWVFIVLTYSSWAGSSYFYLDQNTNWALSPAQSRERNAACSVL  3840
             LFYIVMKLLHRETI  Y+WVFI LTYS W  SSYFYLDQ+TNWALSPA+SR+ N  CS+L
Sbjct  828   LFYIVMKLLHRETIGWYSWVFIALTYSVWFSSSYFYLDQSTNWALSPAESRQSNRQCSLL  887

Query  3841  RLFDAHDAWHAMSAVAMFLSFNMYLTLDDGLAGADRARVPV  3963
             +L+D+HD WH MS++AMF SFNMYLT+DDGL    R  + V
Sbjct  888   QLYDSHDIWHFMSSIAMFFSFNMYLTIDDGLLNVGRDEIAV  928


> ta_transcript84451_1
Length=645

 Score =   709 bits (1831),  Expect = 0.0, Method: Compositional matrix adjust.
 Identities = 402/606 (66%), Positives = 455/606 (75%), Gaps = 56/606 (9%)
 Frame = +1

Query  1     MTEWYRMKTGLNNKHNSSELSVETSCDMSDPFNIFSDNLSPIPASIVPRKLDFSNLDDDD  180
             M E YRMK G N+KHNSSELSVETSCD SDPFN+FSDNLSPIPASIVPRKLDF+N+DD+D
Sbjct  34    MAETYRMKDGYNHKHNSSELSVETSCDTSDPFNMFSDNLSPIPASIVPRKLDFTNVDDED  93

Query  181   GIRDESQAPVSHSPPYKRVRALRLFDSPHTPKTLLEKCSTPTHHPPRTRLFPPKINVQTg  360
              ++D S AP+S SPPYKRVRALRLFDSPHTPKTLLEKCSTPTH+  RTRLFPPK++V T
Sbjct  94    PMKDASMAPMSLSPPYKRVRALRLFDSPHTPKTLLEKCSTPTHNNTRTRLFPPKLSV-TN  152

Query  361   mpsgsshhlhppsgdddsALGSLPPDELDESRLTVRRPIANINPFTPDGQALNKKKRALS  540
                  S H   P  DDDSALGSL PD+   S L +RR +ANINPFTP GQALNKKKRALS
Sbjct  153   GIPSGSSHHLHPPSDDDSALGSLLPDD---SGLAIRRSVANINPFTPQGQALNKKKRALS  209

Query  541   KTPTWDGTPEQPAKRLRESNISRYNVEFIELGVIGRGQFGRVTRCVNKLDGCVYALKRSL  720
             KTPTWD +PE PAKRLRESNISRY VEF+EL VIGRG+FG VT+CVNKLDGCVYA+KRS+
Sbjct  210   KTPTWDASPELPAKRLRESNISRYTVEFVELEVIGRGEFGLVTKCVNKLDGCVYAIKRSI  269

Query  721   RPVAGSAAERAALTEVYAHAALGKHPHVVRYYSAWAEDDHMIIQNEYCDGGSLQLKMED-  897
             +PVAGSAAERAALTEVYAHAALGKHPHVVRYYSAWAEDDHM+IQNEYCDGGSLQ  + D
Sbjct  270   KPVAGSAAERAALTEVYAHAALGKHPHVVRYYSAWAEDDHMVIQNEYCDGGSLQQYVTDN  329

Query  898   GplpesellillshiaaGLAYIHSQQLVHMDVKPGNIFICSGDVDACRESDDGYDDEEPA  1077
             GPLPESELL+LL+HIA GLAYIHS QLVHMDVKPGNIFI   D      S D  DDE P
Sbjct  330   GPLPESELLVLLAHIAHGLAYIHSLQLVHMDVKPGNIFISRSDTPPPGNSSDSEDDEAPQ  389

Query  1078  PNHKYKIGDLGHVTCVSSPAVEEGDCRYLPKEVLHEDFSQLPKADIFAFGLTLFEAAGGG  1257
             P HKYKIGDLGHVTC  +P VEEGDCRYLP+EVL EDF+ L KADIFAFGLTL+EAAGGG
Sbjct  390   PKHKYKIGDLGHVTCAWAPTVEEGDCRYLPREVLQEDFTHLAKADIFAFGLTLYEAAGGG  449

Query  1258  PLPKNGQKWHDYRDGKLPDLPNLSREFNDLLKSMVDpspsarpsarrlrrmawrggawrg  1437
             PLP+NGQ WHD RDGKLP+L NLSREFN LLK MV P PS RPS+R
Sbjct  450   PLPQNGQDWHDIRDGKLPELTNLSREFNQLLKQMVHPDPSQRPSSRG-------------  496

Query  1438  vaaPIKSRAQLRRELAAA------------------NMKNELLARKLHDAAKCIKSLTPS  1563
                 ++  + LRR +A+                   NMKNELL R+L +A++CIKSLTP+
Sbjct  497   ----LRKHSILRRGVASGSDTRRASRAALARELAATNMKNELLTRQLQEASRCIKSLTPN  552

Query  1564  L----ESSKFRTRSAA------------KRTQKPRIDTCLSD*IKYDVPARVTVACDSAN  1695
             +    ES+KFRTRSA             +R+ KP+ D  L+D ++     R     +++
Sbjct  553   IQINQESAKFRTRSAKRLQPTESTHRSDRRSTKPKADAQLADMLQAVTSPRRIDRRNNSR  612

Query  1696  RTYPLF  1713
             RT  L
Sbjct  613   RTKSLI  618


> ta_transcript84449_1
Length=896

 Score =   714 bits (1844),  Expect = 0.0, Method: Compositional matrix adjust.
 Identities = 398/582 (68%), Positives = 446/582 (77%), Gaps = 56/582 (10%)
 Frame = +1

Query  1     MTEWYRMKTGLNNKHNSSELSVETSCDMSDPFNIFSDNLSPIPASIVPRKLDFSNLDDDD  180
             M E YRMK G N+KHNSSELSVETSCD SDPFN+FSDNLSPIPASIVPRKLDF+N+DD+D
Sbjct  34    MAETYRMKDGYNHKHNSSELSVETSCDTSDPFNMFSDNLSPIPASIVPRKLDFTNVDDED  93

Query  181   GIRDESQAPVSHSPPYKRVRALRLFDSPHTPKTLLEKCSTPTHHPPRTRLFPPKINVQTg  360
              ++D S AP+S SPPYKRVRALRLFDSPHTPKTLLEKCSTPTH+  RTRLFPPK++V T
Sbjct  94    PMKDASMAPMSLSPPYKRVRALRLFDSPHTPKTLLEKCSTPTHNNTRTRLFPPKLSV-TN  152
```

```
Query  361   mpsgsshhlhppsgdddsALGSLPPDELDESRLTVRRPIANINPFTPDGQALNKKKRALS  540
                  S H   P  DDDSALGSL PD+   S L +RR +ANINPFTP GQALNKKKRALS
Sbjct  153   GIPSGSSHHLHPPSDDDSALGSLLPDD---SGLAIRRSVANINPFTPQGQALNKKKRALS  209

Query  541   KTPTWDGTPEQPAKRLRESNISRYNVEFIELGVIGRGQFGRVTRCVNKLDGCVYALKRSL  720
             KTPTWD +PE PAKRLRESNISRY VEF+EL VIGRG+FG VT+CVNKLDGCVYA+KRS+
Sbjct  210   KTPTWDASPELPAKRLRESNISRYTVEFVELEVIGRGEFGLVTKCVNKLDGCVYAIKRSI  269

Query  721   RPVAGSAAERAALTEVYAHAALGKHPHVVRYYSAWAEDDHMIIQNEYCDGGSLQLKMED-  897
             +PVAGSAAERAALTEVYAHAALGKHPHVVRYYSAWAEDDHM+IQNEYCDGGSLQ  + D
Sbjct  270   KPVAGSAAERAALTEVYAHAALGKHPHVVRYYSAWAEDDHMVIQNEYCDGGSLQQYVTDN  329

Query  898   GplpesellillshiaaGLAYIHSQQLVHMDVKPGNIFICSGDVDACRESDDGYDDEEPA  1077
             GPLPESELL+LL+HIA GLAYIHS QLVHMDVKPGNIFI   D      S D  DDE P
Sbjct  330   GPLPESELLVLLAHIAHGLAYIHSLQLVHMDVKPGNIFISRSDTPPPGNSSDSEDDEAPQ  389

Query  1078  PNHKYKIGDLGHVTCVSSPAVEEGDCRYLPKEVLHEDFSQLPKADIFAFGLTLFEAAGGG  1257
             P HKYKIGDLGHVTC  +P VEEGDCRYLP+EVL EDF+ L KADIFAFGLTL+EAAGGG
Sbjct  390   PKHKYKIGDLGHVTCAWAPTVEEGDCRYLPREVLQEDFTHLAKADIFAFGLTLYEAAGGG  449

Query  1258  PLPKNGQKWHDYRDGKLPDLPNLSREFNDLLKSMVDpspsarpsarrlrrmawrggawrg  1437
             PLP+NGQ WHD RDGKLP+L NLSREFN LLK MV P PS RPS+R
Sbjct  450   PLPQNGQDWHDIRDGKLPELTNLSREFNQLLKQMVHPDPSQRPSSRG-------------  496

Query  1438  vaaPIKSRAQLRRELAAA------------------NMKNELLARKLHDAAKCIKSLTPS  1563
                 ++  + LRR +A+                   NMKNELL R+L +A++CIKSLTP+
Sbjct  497   ----LRKHSILRRGVASGSDTRRASRAALARELAATNMKNELLTRQLQEASRCIKSLTPN  552

Query  1564  L----ESSKFRTRSAA------------KRTQKPRIDTCLSD  1641
             +    ES+KFRTRSA             +R+ KP+ D  L+D
Sbjct  553   IQINQESAKFRTRSAKRLQPTESTHRSDRRSTKPKADAQLAD  594
```

```
> ta_transcript84450_1
Length=858

 Score =   712 bits (1839),  Expect = 0.0, Method: Compositional matrix adjust.
 Identities = 398/582 (68%), Positives = 446/582 (77%), Gaps = 56/582 (10%)
 Frame = +1

Query  1     MTEWYRMKTGLNNKHNSSELSVETSCDMSDPFNIFSDNLSPIPASIVPRKLDFSNLDDDD  180
             M E YRMK G N+KHNSSELSVETSCD SDPFN+FSDNLSPIPASIVPRKLDF+N+DD+D
Sbjct  34    MAETYRMKDGYNHKHNSSELSVETSCDTSDPFNMFSDNLSPIPASIVPRKLDFTNVDDED  93

Query  181   GIRDESQAPVSHSPPYKRVRALRLFDSPHTPKTLLEKCSTPTHHPPRTRLFPPKINVQTg  360
              ++D S AP+S SPPYKRVRALRLFDSPHTPKTLLEKCSTPTH+  RTRLFPPK++V T
Sbjct  94    PMKDASMAPMSLSPPYKRVRALRLFDSPHTPKTLLEKCSTPTHNNTRTRLFPPKLSV-TN  152

Query  361   mpsgsshhlhppsgdddsALGSLPPDELDESRLTVRRPIANINPFTPDGQALNKKKRALS  540
                  S H   P  DDDSALGSL PD+   S L +RR +ANINPFTP GQALNKKKRALS
Sbjct  153   GIPSGSSHHLHPPSDDDSALGSLLPDD---SGLAIRRSVANINPFTPQGQALNKKKRALS  209

Query  541   KTPTWDGTPEQPAKRLRESNISRYNVEFIELGVIGRGQFGRVTRCVNKLDGCVYALKRSL  720
             KTPTWD +PE PAKRLRESNISRY VEF+EL VIGRG+FG VT+CVNKLDGCVYA+KRS+
Sbjct  210   KTPTWDASPELPAKRLRESNISRYTVEFVELEVIGRGEFGLVTKCVNKLDGCVYAIKRSI  269

Query  721   RPVAGSAAERAALTEVYAHAALGKHPHVVRYYSAWAEDDHMIIQNEYCDGGSLQLKMED-  897
             +PVAGSAAERAALTEVYAHAALGKHPHVVRYYSAWAEDDHM+IQNEYCDGGSLQ  + D
Sbjct  270   KPVAGSAAERAALTEVYAHAALGKHPHVVRYYSAWAEDDHMVIQNEYCDGGSLQQYVTDN  329

Query  898   GplpesellillshiaaGLAYIHSQQLVHMDVKPGNIFICSGDVDACRESDDGYDDEEPA  1077
             GPLPESELL+LL+HIA GLAYIHS QLVHMDVKPGNIFI   D      S D  DDE P
Sbjct  330   GPLPESELLVLLAHIAHGLAYIHSLQLVHMDVKPGNIFISRSDTPPPGNSSDSEDDEAPQ  389

Query  1078  PNHKYKIGDLGHVTCVSSPAVEEGDCRYLPKEVLHEDFSQLPKADIFAFGLTLFEAAGGG  1257
             P HKYKIGDLGHVTC  +P VEEGDCRYLP+EVL EDF+ L KADIFAFGLTL+EAAGGG
Sbjct  390   PKHKYKIGDLGHVTCAWAPTVEEGDCRYLPREVLQEDFTHLAKADIFAFGLTLYEAAGGG  449

Query  1258  PLPKNGQKWHDYRDGKLPDLPNLSREFNDLLKSMVDpspsarpsarrlrrmawrggawrg  1437
             PLP+NGQ WHD RDGKLP+L NLSREFN LLK MV P PS RPS+R
Sbjct  450   PLPQNGQDWHDIRDGKLPELTNLSREFNQLLKQMVHPDPSQRPSSRG-------------  496

Query  1438  vaaPIKSRAQLRRELAAA------------------NMKNELLARKLHDAAKCIKSLTPS  1563
                 ++  + LRR +A+                   NMKNELL R+L +A++CIKSLTP+
Sbjct  497   ----LRKHSILRRGVASGSDTRRASRAALARELAATNMKNELLTRQLQEASRCIKSLTPN  552
```

```
Query  1564  L----ESSKFRTRSAA------------KRTQKPRIDTCLSD  1641
             +    ES+KFRTRSA             +R+ KP+ D  L+D
Sbjct  553   IQINQESAKFRTRSAKRLQPTESTHRSDRRSTKPKADAQLAD  594


> ta_transcript84448_1
Length=975

 Score =   713 bits (1840),  Expect = 0.0, Method: Compositional matrix adjust.
 Identities = 398/582 (68%), Positives = 446/582 (77%), Gaps = 56/582 (10%)
 Frame = +1

Query  1     MTEWYRMKTGLNNKHNSSELSVETSCDMSDPFNIFSDNLSPIPASIVPRKLDFSNLDDDD  180
             M E YRMK G N+KHNSSELSVETSCD SDPFN+FSDNLSPIPASIVPRKLDF+N+DD+D
Sbjct  34    MAETYRMKDGYNHKHNSSELSVETSCDTSDPFNMFSDNLSPIPASIVPRKLDFTNVDDED  93

Query  181   GIRDESQAPVSHSPPYKRVRALRLFDSPHTPKTLLEKCSTPTHHPPRTRLFPPKINVQTg  360
              ++D S AP+S SPPYKRVRALRLFDSPHTPKTLLEKCSTPTH+  RTRLFPPK++V T
Sbjct  94    PMKDASMAPMSLSPPYKRVRALRLFDSPHTPKTLLEKCSTPTHNNTRTRLFPPKLSV-TN  152

Query  361   mpsgsshhlhppsgdddsALGSLPPDELDESRLTVRRPIANINPFTPDGQALNKKKRALS  540
                  S H   P  DDDSALGSL PD+   S L +RR +ANINPFTP GQALNKKKRALS
Sbjct  153   GIPSGSSHHLHPPSDDDSALGSLLPDD---SGLAIRRSVANINPFTPQGQALNKKKRALS  209

Query  541   KTPTWDGTPEQPAKRLRESNISRYNVEFIELGVIGRGQFGRVTRCVNKLDGCVYALKRSL  720
             KTPTWD +PE PAKRLRESNISRY VEF+EL VIGRG+FG VT+CVNKLDGCVYA+KRS+
Sbjct  210   KTPTWDASPELPAKRLRESNISRYTVEFVELEVIGRGEFGLVTKCVNKLDGCVYAIKRSI  269

Query  721   RPVAGSAAERAALTEVYAHAALGKHPHVVRYYSAWAEDDHMIIQNEYCDGGSLQLKMED-  897
             +PVAGSAAERAALTEVYAHAALGKHPHVVRYYSAWAEDDHM+IQNEYCDGGSLQ  + D
Sbjct  270   KPVAGSAAERAALTEVYAHAALGKHPHVVRYYSAWAEDDHMVIQNEYCDGGSLQQYVTDN  329

Query  898   GplpesellillshiaaGLAYIHSQQLVHMDVKPGNIFICSGDVDACRESDDGYDDEEPA  1077
             GPLPESELL+LL+HIA GLAYIHS QLVHMDVKPGNIFI   D      S D  DDE P
Sbjct  330   GPLPESELLVLLAHIAHGLAYIHSLQLVHMDVKPGNIFISRSDTPPPGNSSDSEDDEAPQ  389

Query  1078  PNHKYKIGDLGHVTCVSSPAVEEGDCRYLPKEVLHEDFSQLPKADIFAFGLTLFEAAGGG  1257
             P HKYKIGDLGHVTC  +P VEEGDCRYLP+EVL EDF+ L KADIFAFGLTL+EAAGGG
Sbjct  390   PKHKYKIGDLGHVTCAWAPTVEEGDCRYLPREVLQEDFTHLAKADIFAFGLTLYEAAGGG  449

Query  1258  PLPKNGQKWHDYRDGKLPDLPNLSREFNDLLKSMVDpspsarpsarrlrrmawrggawrg  1437
             PLP+NGQ WHD RDGKLP+L NLSREFN LLK MV P PS RPS+R
Sbjct  450   PLPQNGQDWHDIRDGKLPELTNLSREFNQLLKQMVHPDPSQRPSSRG-------------  496

Query  1438  vaaPIKSRAQLRRELAAA------------------NMKNELLARKLHDAAKCIKSLTPS  1563
                 ++  + LRR +A+                   NMKNELL R+L +A++CIKSLTP+
Sbjct  497   ----LRKHSILRRGVASGSDTRRASRAALARELAATNMKNELLTRQLQEASRCIKSLTPN  552

Query  1564  L----ESSKFRTRSAA------------KRTQKPRIDTCLSD  1641
             +    ES+KFRTRSA             +R+ KP+ D  L+D
Sbjct  553   IQINQESAKFRTRSAKRLQPTESTHRSDRRSTKPKADAQLAD  594


> ta_transcript84453_1
Length=385

 Score =   464 bits (1195),  Expect = 3e-150, Method: Compositional matrix adjust.
 Identities = 236/299 (79%), Positives = 259/299 (87%), Gaps = 4/299 (1%)
 Frame = +1

Query  1    MTEWYRMKTGLNNKHNSSELSVETSCDMSDPFNIFSDNLSPIPASIVPRKLDFSNLDDDD  180
            M E YRMK G N+KHNSSELSVETSCD SDPFN+FSDNLSPIPASIVPRKLDF+N+DD+D
Sbjct  34   MAETYRMKDGYNHKHNSSELSVETSCDTSDPFNMFSDNLSPIPASIVPRKLDFTNVDDED  93

Query  181  GIRDESQAPVSHSPPYKRVRALRLFDSPHTPKTLLEKCSTPTHHPPRTRLFPPKINVQTg  360
             ++D S AP+S SPPYKRVRALRLFDSPHTPKTLLEKCSTPTH+  RTRLFPPK++V T
Sbjct  94   PMKDASMAPMSLSPPYKRVRALRLFDSPHTPKTLLEKCSTPTHNNTRTRLFPPKLSV-TN  152

Query  361  mpsgsshhlhppsgdddsALGSLPPDELDESRLTVRRPIANINPFTPDGQALNKKKRALS  540
                 S H   P  DDDSALGSL PD+   S L +RR +ANINPFTP GQALNKKKRALS
Sbjct  153  GIPSGSSHHLHPPSDDDSALGSLLPDD---SGLAIRRSVANINPFTPQGQALNKKKRALS  209

Query  541  KTPTWDGTPEQPAKRLRESNISRYNVEFIELGVIGRGQFGRVTRCVNKLDGCVYALKRSL  720
            KTPTWD +PE PAKRLRESNISRY VEF+EL VIGRG+FG VT+CVNKLDGCVYA+KRS+
Sbjct  210  KTPTWDASPELPAKRLRESNISRYTVEFVELEVIGRGEFGLVTKCVNKLDGCVYAIKRSI  269

Query  721  RPVAGSAAERAALTEVYAHAALGKHPHVVRYYSAWAEDDHMIIQNEYCDGGSLQLKMED  897
```

```
            +PVAGSAAERAALTEVYAHAALGKHPHVVRYYSAWAEDDHM+IQNEYCDGGSLQ  + D
Sbjct  270  KPVAGSAAERAALTEVYAHAALGKHPHVVRYYSAWAEDDHMVIQNEYCDGGSLQQYVTD  328


> ta_transcript44271_1
Length=224

 Score =   156 bits (395),  Expect = 2e-41, Method: Compositional matrix adjust.
 Identities = 101/188 (54%), Positives = 117/188 (62%), Gaps = 1/188 (1%)
 Frame = -2

Query  3365  PINMMSARTPKVACALALMSGCRDW*IFTMQSADVTYMNDVSNWKFMLLGQTW*AADRKP  3186
             P+ M +A   KVACA ALMSGCRDW IFTM S   TYMN VSNWK   LGQ W* A+
Sbjct  20    PMAMSTASAAKVACARALMSGCRDWYIFTMLSTASTYMNAVSNWKLSRLGQMW*VAESSA  79

Query  3185  SLIRATPIAYSSPKCWGMPFCFFSSGSFLMILCLLQTCSRNRAPSTT*PRFENTWLKSDS  3006
             + I   P A SSP C GMP+C +       I     +TCS N APS+ *PR ENTWLKS++
Sbjct  80    TTIMLAPSAVSSP*CAGMPYCSWRGLRLRRIR-RRRTCSMNSAPSSK*PRLENTWLKSET  138

Query  3005  IISGCAQKKLK*HRSWFPDWFRSRW*vttscstgsv*NTETVSTVHRYRLERVASTRARH  2826
                GCA +KL *HRS  PD F   W V T+C TGS *NT+TV +V RY  ER  S R+
Sbjct  139   PPHGCAHRKL**HRSSEPDTFNISWKVNTNCMTGSA*NTDTVYSVCRYLSERADSVRSVR  198

Query  2825  LDRSARHS  2802
              DR+A  S
Sbjct  199   RDRAATFS  206


> ta_transcript44268_1
Length=404

 Score =   156 bits (395),  Expect = 9e-40, Method: Compositional matrix adjust.
 Identities = 101/188 (54%), Positives = 117/188 (62%), Gaps = 1/188 (1%)
 Frame = -2

Query  3365  PINMMSARTPKVACALALMSGCRDW*IFTMQSADVTYMNDVSNWKFMLLGQTW*AADRKP  3186
             P+ M +A   KVACA ALMSGCRDW IFTM S   TYMN VSNWK   LGQ W* A+
Sbjct  20    PMAMSTASAAKVACARALMSGCRDWYIFTMLSTASTYMNAVSNWKLSRLGQMW*VAESSA  79

Query  3185  SLIRATPIAYSSPKCWGMPFCFFSSGSFLMILCLLQTCSRNRAPSTT*PRFENTWLKSDS  3006
             + I   P A SSP C GMP+C +       I     +TCS N APS+ *PR ENTWLKS++
Sbjct  80    TTIMLAPSAVSSP*CAGMPYCSWRGLRLRRIR-RRRTCSMNSAPSSK*PRLENTWLKSET  138

Query  3005  IISGCAQKKLK*HRSWFPDWFRSRW*vttscstgsv*NTETVSTVHRYRLERVASTRARH  2826
                GCA +KL *HRS  PD F   W V T+C TGS *NT+TV +V RY  ER  S R+
Sbjct  139   PPHGCAHRKL**HRSSEPDTFNISWKVNTNCMTGSA*NTDTVYSVCRYLSERADSVRSVR  198

Query  2825  LDRSARHS  2802
              DR+A  S
Sbjct  199   RDRAATFS  206


> ta_transcript44264_1
Length=747

 Score =   158 bits (399),  Expect = 7e-39, Method: Compositional matrix adjust.
 Identities = 101/188 (54%), Positives = 117/188 (62%), Gaps = 1/188 (1%)
 Frame = -2

Query  3365  PINMMSARTPKVACALALMSGCRDW*IFTMQSADVTYMNDVSNWKFMLLGQTW*AADRKP  3186
             P+ M +A   KVACA ALMSGCRDW IFTM S   TYMN VSNWK   LGQ W* A+
Sbjct  20    PMAMSTASAAKVACARALMSGCRDWYIFTMLSTASTYMNAVSNWKLSRLGQMW*VAESSA  79

Query  3185  SLIRATPIAYSSPKCWGMPFCFFSSGSFLMILCLLQTCSRNRAPSTT*PRFENTWLKSDS  3006
             + I   P A SSP C GMP+C +       I     +TCS N APS+ *PR ENTWLKS++
Sbjct  80    TTIMLAPSAVSSP*CAGMPYCSWRGLRLRRIR-RRRTCSMNSAPSSK*PRLENTWLKSET  138

Query  3005  IISGCAQKKLK*HRSWFPDWFRSRW*vttscstgsv*NTETVSTVHRYRLERVASTRARH  2826
                GCA +KL *HRS  PD F   W V T+C TGS *NT+TV +V RY  ER  S R+
Sbjct  139   PPHGCAHRKL**HRSSEPDTFNISWKVNTNCMTGSA*NTDTVYSVCRYLSERADSVRSVR  198

Query  2825  LDRSARHS  2802
              DR+A  S
Sbjct  199   RDRAATFS  206


> ta_transcript44270_1
```

```
Length=226

 Score =   135 bits (339),  Expect = 5e-34, Method: Compositional matrix adjust.
 Identities = 78/142 (55%), Positives = 89/142 (63%), Gaps = 1/142 (1%)
 Frame = -2

Query  3365  PINMMSARTPKVACALALMSGCRDW*IFTMQSADVTYMNDVSNWKFMLLGQTW*AADRKP  3186
             P+ M +A   KVACA ALMSGCRDW IFTM S   TYMN VSNWK   LGQ W* A+
Sbjct  20    PMAMSTASAAKVACARALMSGCRDWYIFTMLSTASTYMNAVSNWKLSRLGQMW*VAESSA  79

Query  3185  SLIRATPIAYSSPKCWGMPFCFFSSGSFLMILCLLQTCSRNRAPSTT*PRFENTWLKSDS  3006
             + I   P A SSP C GMP+C +        I     +TCS N APS+ *PR ENTWLKS++
Sbjct  80    TTIMLAPSAVSSP*CAGMPYCSWRGLRLRRIR-RRRTCSMNSAPSSK*PRLENTWLKSET  138

Query  3005  IISGCAQKKLK*HRSWFPDWFR  2940
                GCA +KL *HRS  PD F
Sbjct  139   PPHGCAHRKL**HRSSEPDTFN  160


****************************************************************************************************
**********************************************************************
Query= gi|164448647:159-2792 Bombyx mori sid-1-related gene3 (Sir-3), mRNA

Length=2634
                                                                      Score     E
Sequences producing significant alignments:                          (Bits)  Value

  ta_transcript74094_1                                                  369   1e-111
  ta_transcript44271_1                                                  219   2e-64
  ta_transcript44268_1                                                  218   8e-62
  ta_transcript44264_1                                                  220   8e-60
  ta_transcript44270_1                                                  187   7e-53
  ta_transcript44272_1                                                  125   4e-32


> ta_transcript74094_1
Length=937

 Score =   369 bits (947),  Expect = 1e-111, Method: Compositional matrix adjust.
 Identities = 213/379 (56%), Positives = 274/379 (72%), Gaps = 7/379 (2%)
 Frame = +1

Query  1504  SDRYLHTLYTVAVFYALPVLQFVAAFQVMLNISGSLDMCYYNFLCAHPAGGLSDFNHVFS  1683
             S  YL   + TVAVFY LPV+Q V   +Q +LN SG+ D+CY+NFLCAHP   LSDFNHV+S
Sbjct  550   SKMYLWNILTVAVFYTLPVIQLVVTYQRLLNQSGNQDLCYFNFLCAHPLMVLSDFNHVYS  609

Query  1684  NlgylllgalfmlqlqrrkrnrkrAPRH-EEYGIPAHYGLLSSLGAAMMVVALLSASYHV  1860
             NLGY++LG+LF+ Q+ RR        P H +E GIP H+GLL ++G A++   LLSA+YHV
Sbjct  610   NLGYVILGSLFLFQVWRRHSYHTIMPAHRKELGIPQHFGLLYAMGIALVSEGLLSAAYHV  669

Query  1861  CPNSLNFQFDTAFMYVLAVLCMVKIYQSRHPDINARAHATFGVLAVFIAlvvwgvlgggp  2040
             CPNS+NFQFDT+FMYV +VLCMVKIYQSRHPDINARAHATFGVLA+ I + + GVL
Sbjct  670   CPNSMNFQFDTSFMYVTSVLCMVKIYQSRHPDINARAHATFGVLALIIFIGLVGVLNANF  729

Query  2041  lFWsvftvlhvftflllslRIYYVGQFRLEKSSLAVAARGLRARPL--YTP----RLVML  2202
              FW +FTVLH+ T L+++ +IYY+G+FRL+    +  AAR L  RPL    TP    R VML
Sbjct  730   YFWIMFTVLHLLTCLVMTFQIYYLGRFRLDGGVVYRAARELIRRPLAAITPTHCGRCVML  789

Query  2203  LIANAANWGFAIYGLLTHAGDIATHllnvllcntllYIVFYVLMKLLHGERIRWYSWCFL  2382
             L+AN +NW  A YG+  H+ D A+HLL VL+ N  LY +FY++MKLLH E I WYSW F+
Sbjct  790   LLANLSNWAIAAYGVSQHSRDFASHLLLVLMSNLFLYTLFYIVMKLLHRETIGWYSWVFI  849

Query  2383  aaaaaCWVPALYFFTSGSTDWSATPARSRHRNHECRVLQFYDSHDLWHMLSAAALYFTFN  2562
             A    + W  + YF+   ST+W+ +PA SR   N +C +LQ YDSHD+WH +S+ A++F+FN
Sbjct  850   ALTYSVWFSSSYFYLDQSTNWALSPAESRQSNRQCSLLQLYDSHDIWHFMSSIAMFFSFN  909

Query  2563  VMLTWDDGLSAVKRTEIAV  2619
             + LT DDGL  V R EIAV
Sbjct  910   MYLTIDDGLLNVGRDEIAV  928


 Score =   135 bits (340),  Expect = 8e-32, Method: Compositional matrix adjust.
 Identities = 92/382 (24%), Positives = 163/382 (43%), Gaps = 60/382 (16%)
 Frame = +1

Query  19    LALCVSVVLASNITVEQRILNLEE------EYTLVVTPSIEFILQFVPNEDQAEFPSRLW  180
             L +C+ V  A  +  ++ + +         EY + V    E+I++F  +E   + P+R+
```

```
Sbjct  64   LFVCLHVSYAQKLKRDEIVFHYNNTYKYNVEYVVDVDKHTEYIMEFAGDEKAYDTPARVT  123

Query  181  VRSVGGDTSRPLLLTARTKTGATTWQLPY--QSGSML--MSELERTLCWDGSPTDAVGAP  348
            V S   + + PL +TAR + G  +WQLP   Q+   L   + + RTLC      +
Sbjct  124  VASDFANRTYPLFITARQQKGVFSWQLPMLIQTPMSLEQFNNISRTLC---PHNNLYNED  180

Query  349  SECEGAGSQRGFTLHLASACAAPLTVTLRAAPARDWLLGFQARTTVTATQTGPAVNYYDF  528
             E     +     +HL SA   P+ VT+      D+ +     T +T T + P   +Y F
Sbjct  181  EESCDVTTINTPIVHLTSASPEPIRVTIVVESVLDFYIVTNKTTNMTVTPSQPKYYFYPF  240

Query  529  -----------------------------------------------IPGQNSVRLIVES  567
                                                           +    SV +++ES
Sbjct  241  KQNPELDLHTQKIKKKYKCNEELDHKELISLDRYFLKSNALKRQYSWLTRPKSVIVMIES  300

Query  568  EDEVCATISVQRYTCPLAETIEDIDLTTLRMTVMRSGAVQLSRSLYPMGFYVVSLVRPDD  747
            +D++CA +S+Q ++CP+ +   DI      +T+ R G + L++ ++P+GFY+V +V+  D
Sbjct  301  DDDICAVVSIQNFSCPVFDNERDILYDGYYLTMTRRGGITLTQDMFPLGFYIVFIVKSSD  360

Query  748  AACSGEPAPEDDWLLEAALWAHTDRPSPPATLRQKTFTLTVRASLSRAQYMVGAGVTVAV  927
              C      + +       W H    +     R KTF+  +  ++S  +Y++ AG  +
Sbjct  361  DDCVMGGVSDVNVSRITVRWQHGKATATSDDGRVKTFSFRIIETISYREYLIAAGAVLGF  420

Query  928  FLLFYAGFAALVLAQRWPACAR  993
            F+ FYAGFA +V+ QR     R
Sbjct  421  FMSFYAGFAIVVVYQRVQGLRR  442


> ta_transcript44271_1
Length=224

 Score =   219 bits (558),  Expect = 2e-64, Method: Compositional matrix adjust.
 Identities = 121/163 (74%), Positives = 130/163 (80%), Gaps = 0/163 (0%)
 Frame = -2

Query  2006  AMKTASTPKVACARALMSGCRDW*IFTMHSTASTYMKAVSNWKLRLLGQTW*LAESSATT  1827
             AM TAS  KVACARALMSGCRDW IFTM STASTYM AVSNWKL  LGQ W*+AESSATT
Sbjct  22    AMSTASAAKVACARALMSGCRDWYIFTMLSTASTYMNAVSNWKLSRLGQMW*VAESSATT  81

Query  1826  IMAAPRDDSSP*CAGIPYSSCRGARFLFRFLRCSCSMKSAPSSR*PRFENTWLKSDSPPA  1647
             IM AP   SSP*CAG+PY S RG R      R +CSM SAPSS+*PR ENTWLKS++PP
Sbjct  82    IMLAPSAVSSP*CAGMPYCSWRGLRLRRIRRRRTCSMNSAPSSK*PRLENTWLKSETPPH  141

Query  1646  GCAHRKL**HMSSDPDMFSITWKAATNCSTGSA*NTATV*SVC  1518
             GCAHRKL**H SS+PD F+I+WK  TNC TGSA*NT TV SVC
Sbjct  142   GCAHRKL**HRSSEPDTFNISWKVNTNCMTGSA*NTDTVYSVC  184


> ta_transcript44268_1
Length=404

 Score =   218 bits (555),  Expect = 8e-62, Method: Compositional matrix adjust.
 Identities = 121/163 (74%), Positives = 130/163 (80%), Gaps = 0/163 (0%)
 Frame = -2

Query  2006  AMKTASTPKVACARALMSGCRDW*IFTMHSTASTYMKAVSNWKLRLLGQTW*LAESSATT  1827
             AM TAS  KVACARALMSGCRDW IFTM STASTYM AVSNWKL  LGQ W*+AESSATT
Sbjct  22    AMSTASAAKVACARALMSGCRDWYIFTMLSTASTYMNAVSNWKLSRLGQMW*VAESSATT  81

Query  1826  IMAAPRDDSSP*CAGIPYSSCRGARFLFRFLRCSCSMKSAPSSR*PRFENTWLKSDSPPA  1647
             IM AP   SSP*CAG+PY S RG R      R +CSM SAPSS+*PR ENTWLKS++PP
Sbjct  82    IMLAPSAVSSP*CAGMPYCSWRGLRLRRIRRRRTCSMNSAPSSK*PRLENTWLKSETPPH  141

Query  1646  GCAHRKL**HMSSDPDMFSITWKAATNCSTGSA*NTATV*SVC  1518
             GCAHRKL**H SS+PD F+I+WK  TNC TGSA*NT TV SVC
Sbjct  142   GCAHRKL**HRSSEPDTFNISWKVNTNCMTGSA*NTDTVYSVC  184


> ta_transcript44264_1
Length=747

 Score =   220 bits (560),  Expect = 8e-60, Method: Compositional matrix adjust.
 Identities = 121/163 (74%), Positives = 130/163 (80%), Gaps = 0/163 (0%)
 Frame = -2

Query  2006  AMKTASTPKVACARALMSGCRDW*IFTMHSTASTYMKAVSNWKLRLLGQTW*LAESSATT  1827
             AM TAS  KVACARALMSGCRDW IFTM STASTYM AVSNWKL  LGQ W*+AESSATT
Sbjct  22    AMSTASAAKVACARALMSGCRDWYIFTMLSTASTYMNAVSNWKLSRLGQMW*VAESSATT  81
```

```
Query  1826  IMAAPRDDSSP*CAGIPYSSCRGARFLFRFLRCSCSMKSAPSSR*PRFENTWLKSDSPPA  1647
             IM AP   SSP*CAG+PY S RG R      R +CSM SAPSS+*PR ENTWLKS++PP
Sbjct  82    IMLAPSAVSSP*CAGMPYCSWRGLRLRRIRRRRTCSMNSAPSSK*PRLENTWLKSETPPH  141

Query  1646  GCAHRKL**HMSSDPDMFSITWKAATNCSTGSA*NTATV*SVC  1518
             GCAHRKL**H SS+PD F+I+WK  TNC TGSA*NT TV SVC
Sbjct  142   GCAHRKL**HRSSEPDTFNISWKVNTNCMTGSA*NTDTVYSVC  184


 Score = 88.6 bits (218),  Expect = 3e-17, Method: Compositional matrix adjust.
 Identities = 119/300 (40%), Positives = 144/300 (48%), Gaps = 21/300 (7%)
 Frame = -2

Query  917  VTPAPTMYCARDSDALTVSVKVFCRRVAGGEG------RSVWaqraasssqsssgagSPE  756
            VT   T YCARDS      V V    ++GG G      R      AA  SQS++   SP
Sbjct  369  VTAHATTYCARDSAGFRSMVSVLA--LSGGAGVVVVVVRPSLPTAAAWYSQSAARGCSPA  426

Query  755  HAASSGRTSDTT*NPIGYRLRESCTAPERMTV--ILrvvrsmssmvSANGHVYLCTEMVA  582
            H +S   T+  T  P G  LR+ C  PER TV  I    R  +  VSA GH
Sbjct  427  HGSSDDSTTTDTKKPRGNSLRDICAPPERSTVMNIPSAGRVTACCVSATGHE*SRMATTE  486

Query  581  HTSSSDSTISRTLFCPGMKS**FTAGPVCVAVTVVRAWKPSSQSLAGAARSVTVRGAAQA  402
            HTSSSD+T SRT  C   K **+T   +CV  T + A K S QS AG+ R+VTV  A  A
Sbjct  487  HTSSSDATTSRTSCCSAEKL**YTRDDICVTRTFLVAPKLSIQSRAGSTRAVTVTDALLA  546

Query  401  DARCRVKPRCEPAPSHSDGAPTASVGEPSQHSVRSSSDISIDPDWY--GSCHvvapvlvl  228
            D  C V       P  SD           +H+VR SS     P     GSCHV APV+
Sbjct  547  DDTCSVNRSVLEPPPFSDT---------RKHNVRWSSYNCSSPSAAM*GSCHV*APVVCR  597

Query  227  avssSGREVSPPTLRTHNRLGNSA*SSFGTNCKMNSMDGVTTNVYSSSRFNILCSTVMLL  48
            AVSS+GR  SP +     R G++A S+ G+N KM SM   T  V S S   +  ST+  L
Sbjct  598  AVSSTGRATSPASAVRRMRAGHAACSAVGSNSKMYSMVPSTVIVCSLSNGTVYLSTLTAL  657


> ta_transcript44270_1
Length=226

 Score =   187 bits (474),  Expect = 7e-53, Method: Compositional matrix adjust.
 Identities = 104/140 (74%), Positives = 112/140 (80%), Gaps = 0/140 (0%)
 Frame = -2

Query  2006  AMKTASTPKVACARALMSGCRDW*IFTMHSTASTYMKAVSNWKLRLLGQTW*LAESSATT  1827
             AM TAS  KVACARALMSGCRDW IFTM STASTYM AVSNWKL  LGQ W*+AESSATT
Sbjct  22    AMSTASAAKVACARALMSGCRDWYIFTMLSTASTYMNAVSNWKLSRLGQMW*VAESSATT  81

Query  1826  IMAAPRDDSSP*CAGIPYSSCRGARFLFRFLRCSCSMKSAPSSR*PRFENTWLKSDSPPA  1647
             IM AP   SSP*CAG+PY S RG R      R +CSM SAPSS+*PR ENTWLKS++PP
Sbjct  82    IMLAPSAVSSP*CAGMPYCSWRGLRLRRIRRRRTCSMNSAPSSK*PRLENTWLKSETPPH  141

Query  1646  GCAHRKL**HMSSDPDMFSI  1587
             GCAHRKL**H SS+PD F+I
Sbjct  142   GCAHRKL**HRSSEPDTFNI  161


> ta_transcript44272_1
Length=119

 Score =   125 bits (313),  Expect = 4e-32, Method: Compositional matrix adjust.
 Identities = 67/86 (78%), Positives = 69/86 (80%), Gaps = 0/86 (0%)
 Frame = -2

Query  2006  AMKTASTPKVACARALMSGCRDW*IFTMHSTASTYMKAVSNWKLRLLGQTW*LAESSATT  1827
             AM TAS  KVACARALMSGCRDW IFTM STASTYM AVSNWKL  LGQ W*+AESSATT
Sbjct  22    AMSTASAAKVACARALMSGCRDWYIFTMLSTASTYMNAVSNWKLSRLGQMW*VAESSATT  81

Query  1826  IMAAPRDDSSP*CAGIPYSSCRGARF  1749
             IM AP   SSP*CAG+PY S RG R
Sbjct  82    IMLAPSAVSSP*CAGMPYCSWRGLRL  107


************************************************************************************************
*********************************************************************
Query= gi|392897213|ref|NM_067422.6| Caenorhabditis elegans Protein SID-2
(sid-2) mRNA, complete cds

Length=936
```

```
***** No hits found *****
************************************************************************************************
**********************************************************************
Query= gi|442620537|ref|NM_079729.3| Drosophila melanogaster Dicer-1 (Dcr-1), mRNA

Length=6913
                                                                      Score     E
Sequences producing significant alignments:                          (Bits)  Value

  ta_transcript89347_1                                                  160   1e-38
  ta_transcript89348_1                                                  160   2e-38


> ta_transcript89347_1
Length=1497

 Score =   160 bits (406),  Expect = 1e-38, Method: Compositional matrix adjust.
 Identities = 98/210 (47%), Positives = 127/210 (60%), Gaps = 21/210 (10%)
 Frame = +2

Query  4865  QVAKVSM----MELLKQLLPYVNEDVLAKKLGDRRELLLSDLVELN-----ADWVARHEQ  5017
             Q++K+++     +LL ++ PY N D+   KL D    +  +++E N     A+      +
Sbjct  1291  QMSKLAIKDNTTDLLNEIFPYGNTDL---KLTDENGQISLEIIEKNKRVILAELKKSLPE  1347

Query  5018  ETYNVMGCGDSFD------NYNDHHRLNLDEKQLKLQYERIE-IEPPTSTKA--ITSAIL  5170
             E    + C    D      NY +   +N+    +    E++E    P   K   + S +
Sbjct  1348  EEVKKLHCFSMIDVDIDAPNYVNEKVVNVGFDNIDSYKEKVEKWRHPDEYKPYYMESKVG  1407

Query  5171  PAGFSFDRQPDLVGHPGPSPSIILQALTMSNANDGINLERLETIGDSFLKYAITTYLYIT  5350
                F FD QPDL GHPGPSPS+ILQALTMSNANDGINLERLETIGDSFLK+AIT YLY
Sbjct  1408  GKEFDFDYQPDLEGHPGPSPSVILQALTMSNANDGINLERLETIGDSFLKFAITAYLYCA  1467

Query  5351  YENVHEGKLSHLRSKQVANLNLYRLGRRKR  5440
             +  VHEGKLSH+RSKQV+NLNLYRLGR KR
Sbjct  1468  HPTVHEGKLSHMRSKQVSNLNLYRLGRNKR  1497


 Score = 37.7 bits (86),  Expect = 0.54, Method: Compositional matrix adjust.
 Identities = 23/50 (46%), Positives = 28/50 (56%), Gaps = 9/50 (18%)
 Frame = +2

Query  4166  LQEADDF---IEIGTWSNDMADDIASFNQEDDDEDDAFHLPVLPANVKFC  4306
             LQE + +    EIGTWSN+MA  I        DDE D +  P LP N+ FC
Sbjct  1010  LQEKECYENAFEIGTWSNEMASSIPL-----DDEYDDYTQP-LPPNLTFC  1053


 Score = 37.0 bits (84),  Expect = 0.91, Method: Compositional matrix adjust.
 Identities = 21/40 (53%), Positives = 24/40 (60%), Gaps = 0/40 (0%)
 Frame = +3

Query  3417  RSARRSRLIRNASRMPSLCRGIATRINRSISMWRRYVHIY  3536
             R  +R  L+R  +  PS  RGI TRINRS S  RRYV  Y
Sbjct  764   RLEKRLFLMRTNTERPSSHRGIGTRINRSFS**RRYVGNY  803


> ta_transcript89348_1
Length=1490

 Score =   160 bits (406),  Expect = 2e-38, Method: Compositional matrix adjust.
 Identities = 98/210 (47%), Positives = 127/210 (60%), Gaps = 21/210 (10%)
 Frame = +2

Query  4865  QVAKVSM----MELLKQLLPYVNEDVLAKKLGDRRELLLSDLVELN-----ADWVARHEQ  5017
             Q++K+++     +LL ++ PY N D+   KL D    +  +++E N     A+      +
Sbjct  1284  QMSKLAIKDNTTDLLNEIFPYGNTDL---KLTDENGQISLEIIEKNKRVILAELKKSLPE  1340

Query  5018  ETYNVMGCGDSFD------NYNDHHRLNLDEKQLKLQYERIE-IEPPTSTKA--ITSAIL  5170
             E    + C    D      NY +   +N+    +    E++E    P   K   + S +
Sbjct  1341  EEVKKLHCFSMIDVDIDAPNYVNEKVVNVGFDNIDSYKEKVEKWRHPDEYKPYYMESKVG  1400

Query  5171  PAGFSFDRQPDLVGHPGPSPSIILQALTMSNANDGINLERLETIGDSFLKYAITTYLYIT  5350
                F FD QPDL GHPGPSPS+ILQALTMSNANDGINLERLETIGDSFLK+AIT YLY
Sbjct  1401  GKEFDFDYQPDLEGHPGPSPSVILQALTMSNANDGINLERLETIGDSFLKFAITAYLYCA  1460

Query  5351  YENVHEGKLSHLRSKQVANLNLYRLGRRKR  5440
```

```
             +  VHEGKLSH+RSKQV+NLNLYRLGR KR
Sbjct  1461  HPTVHEGKLSHMRSKQVSNLNLYRLGRNKR  1490


 Score = 37.4 bits (85),  Expect = 0.58, Method: Compositional matrix adjust.
 Identities = 23/50 (46%), Positives = 28/50 (56%), Gaps = 9/50 (18%)
 Frame = +2

Query  4166  LQEADDF---IEIGTWSNDMADDIASFNQEDDDEDDAFHLPVLPANVKFC  4306
             LQE + +    EIGTWSN+MA  I       DDE D +  P LP N+ FC
Sbjct  1010  LQEKECYENAFEIGTWSNEMASSIPL-----DDEYDDYTQP-LPPNLTFC  1053


 Score = 37.0 bits (84),  Expect = 0.84, Method: Compositional matrix adjust.
 Identities = 21/40 (53%), Positives = 24/40 (60%), Gaps = 0/40 (0%)
 Frame = +3

Query  3417  RSARRSRLIRNASRMPSLCRGIATRINRSISMWRRYVHIY  3536
             R  +R  L+R  +  PS  RGI TRINRS S  RRYV  Y
Sbjct  764   RLEKRLFLMRTNTERPSSHRGIGTRINRSFS**RRYVGNY  803

************************************************************************************************
**********************************************************************

Query= gi|300669732|dbj|AB566386.1| Bombyx mori Dicer-2 mRNA for DICER-2,complete cds

Length=5034
                                                                      Score     E
Sequences producing significant alignments:                          (Bits)  Value

  ta_transcript86357_1                                                  288   4e-79
  ta_transcript86358_1                                                  280   3e-77
  ta_transcript86359_1                                                  150   2e-36



> ta_transcript86357_1
Length=1072

 Score =   288 bits (736),  Expect = 4e-79, Method: Compositional matrix adjust.
 Identities = 337/1061 (32%), Positives = 498/1061 (47%), Gaps = 34/1061 (3%)
 Frame = -1

Query  4977  VDADSVGGETLVVLRERNRHHYLRDVRIDDYLFFENRTSVYHFV*NFDGLLRYAFRERID  4798
             V   +V G     L    RHH+L +V + +  F   RT VY  +     L      ER++
Sbjct  1     VGPAAVHGVLFAALGHCYRHHHLLEVLVGEPGFAYLRTGVYILIQQSHWLFGDVS*ERVN  60

Query  4797  LDVHYLVQNRPHRLQIPARVQVHGAD*CFENVAQHLRHINIFSDLTIDFADK*QNVELNL  4618
             L  H  V++ P+ L + A VQV+ A * FE +A++L H ++FSD TI   ++ Q++ L +
Sbjct  61    LVAHDSVEDCPNSL*VSAAVQVYSAG*RFEYIAENLGHFHVFSDHTILLVNQIQHIFLYI  120

Query  4617  VISFYLMFDEFHYRLLQRRVQLRAEVLVQSKFDGVGRERDVIDEGGSQVSHFAGLQFATV  4438
             ++   L+  E HY  L   VQL A V VQ+ FD +  E DV+DE  S +   +G+Q  T
Sbjct  121   MLPSLLVLYERHYSRL*AGVQLVA*VFVQAHFDNIRGESDVVDESRS*IR*VSGIQRGTA  180

Query  4437  LEDV*RHQEIDYGVPEELEALvrarqpvpdvrrvrqsv*QEG*IFERVV*DLFDISSRIV  4258
              +DV  + +I  GV E+L + V   Q V DVRRV +    +  I E ++  LF+I   +V
Sbjct  181   FKDVRSY*KIQNGVAEKL*SFVGTGQSVADVRRVGKRFK*KRWILEFIIEYLFNILPAVV  240

Query  4257  **KVNISFGS*ILR*RCLHRLVEHVCKVILWRKHLHPFK*FYSC*YPSTK*ICADQSFHG  4078
                V+   G  IL     +   E   K++LW K+  PF  F +      +*I +   FH
Sbjct  241   HYSVHFGRGGLILGKNRWNLPGE*RLKIVLWWKNFEPF*HFDTAQDSIAE*ISSH**FHA  300

Query  4077  IGYRFVRNCL*MYIALHRILGTAFGFCLSDAIL---EIIHHRFPAFFT*FTGHYFLFGEL  3907
             +  RFVR+ L  Y+ LH  L T         ++   +++ ++  A F      +F + E
Sbjct  301   VRQRFVRHSLRSYVTLHCNLHTVERLLFQRRLIIS*QLLTYQLKALFGEVPT*HFFWREF  360

Query  3906  YAD*ECWTNLSLFNKFFQFRGRKETWDEEVTSRAELLHLHASTQVEFRTSVI*IPVTDQL  3727
              AD  C  +L +FN       R++  +E V+SR+EL H H +T+++F T V  +PV+++
Sbjct  361   *ADETCRPDLFIFNHLLHLFRREKPGNEVVSSRSELFHFHTTTELDFGTCVEQVPVSNES  420

Query  3726  ALDVR*LTLVHVRKLVVQVQAGGELQERVPQCLRSLQIEGVV*VDRGERGEHVSQFRTSP  3547
             +LDV  L+ +H  +LV+Q+    +LQE +     S Q+E +V V       +V+QFR S
Sbjct  421   SLDVGKLSFIHRWELVMQI*RSSKLQEGIS*RFGSFQVESIVRVYCSNCC*YVAQFRPSG  480

Query  3546  GRRLL*YFDFINVLDGRRCYSWGLRNDSrrgtrvvrcrryrsRHKLVVTFYVH*RDGRQV  3367
```

```
                 L *YFDF   L  RR Y   L          V   R+RSR + VV FYVH    R V
Sbjct  481   RHFLF*YFDFHEFLYRRRQYGCELPRRRAVRRGSVGRDRHRSRDEFVVAFYVHHGYRRYV  540

Query  3366  TLYVDGFDSLIPGVLFVDFLSLQFI*ALF*RYNFLQTIICAF----VYSVNSVDMKW-FF  3202
              L V     LIP +LF   L LQ I AL   YN +    C F    VYSVN V M++ +
Sbjct  541   ALDVQRLGFLIPVILFAYLLFLQLIQALLHGYN-VHRPRCVFVNRVVYSVNIVRMRFIWL  599

Query  3201  NLGQ**IYGYIPVHLYRLK*FRFFQNWLPVFSGV*YFHEQFMCH*QHVNSVNNTGQ*RCL  3022
             +  Q  ++ +  +H YRL      Q     F     F  Q +CH*QH + V++T Q
Sbjct  600   DERQKGLHSHFNIHFYRLPLLSCRQMHDTDFCLPRDFSAQLICH*QHGHPVHDTRQHTRF  659

Query  3021  IPQYSWVIVFNAEFRYKYLCKSLRFIVAERNRYHLQLLVCERFE-RCCSG*EAVHPRAYV  2845
             +PQ +   IV + E  Y+ L K  + + A R   H  L   +  + R C  *     R
Sbjct  660   VPQSAREIVADTELWYENLWKPFKLVFA*RQGNHFHLFF*KSLDGRGCRQ*TVESTRG*P  719

Query  2844  TYLQQWLTFTS*YiqvivvciiicirfiavsIELRFRRHVFENLRHDVPFR*NSPAIPGR  2665
               L+Q L   S Y  V+ + I++ +R   +  +LR   HV +N+ + VP R N  +IP
Sbjct  720   LDLKQGLVLVSHYF*VVFIRIVVGVRSF*IRRKLRLGSHVLQNI*YYVPRRKNDSSIPRG  779

Query  2664  YNAIRVVFFCDYQRFSFLN*RRGDRWHRVVSLHCCPVNVVSIMFQY------RDNVHAVF  2503
             + AI V F  D  R SFL      R +  V LH  PV++V     +      R +V  V
Sbjct  780   HYAISVAFLRDT*RPSFLKRGLHHRPYLCVRLHNFPVDIVVQCACHVGSRSDRHSVKRVI  839

Query  2502  SSLEYQKLLIDRQDLVE*KIMELLH*LEYNRGQFYNCVIDFY*R-------GDFSNGRER  2344
              ++EY KLL+DR  LVE + +E  H       QF + VI F  R        D +
Sbjct  840   ETVEYGKLLLDR*HLVEYEPVEAFH-------QFKSLVIQFDCRIVHEQLGVDLAFSHAY  892

Query  2343  WELAQFRQRLLRQKPVAFVSLYKIVQSLFTIIVGFLECRLHFYYVKIEYRTI--WYCV*V  2170
              ELA  RQ L  ++   F  L   VQ   + I  F   R+   +V++E+     W    V
Sbjct  893   GELANPRQSLHCKQSETFPFLQ*SVQGSRSGIAWFGISRV*CDHVEVEFARFAHW*AFEV  952

Query  2169  *RELYFMLSFSFLGTRLGYILFDLGLAIFSPVRKAATYVISFVINFRVFVRE*C*RMKFA  1990
              RE + +   SFLG   G+++      I   PV +A        +   V  R *   +  +
Sbjct  953   LREHFLVFPHSFLGA--GFLVAVAFFLIRRPVGEAGHQFWLLEVYRSVIFR**SQIV*LS  1010

Query  1989  QFM*LNARLQCSGAFSGFRI*VRVTLNFFFDGGSYGKYDGD  1867
              FM* +A +Q   A    ++ +R T    FD       D D
Sbjct  1011  SFM*RDAGVQSCRALRSLQLGLRFTGYQIFDRAGDRHVDLD  1051


> ta_transcript86358_1
Length=918

 Score =  280 bits (716),  Expect = 3e-77, Method: Compositional matrix adjust.
 Identities = 305/926 (33%), Positives = 446/926 (48%), Gaps = 30/926 (3%)
 Frame = -1

Query  4977  VDADSVGGETLVVLRERNRHHYLRDVRIDDYLFFENRTSVYHFV*NFDGLLRYAFRERID  4798
             V   +V G     L     RHH+L +V + +   F   RT VY   +      L      ER++
Sbjct  1     VGPAAVHGVLFAALGHCYRHHHLLEVLVGEPGFAYLRTGVYILIQQSHWLFGDVS*ERVN  60

Query  4797  LDVHYLVQNRPHRLQIPARVQVHGAD*CFENVAQHLRHINIFSDLTIDFADK*QNVELNL  4618
             L  H  V++ P+ L + A VQV+ A * FE +A++L H ++FSD TI   ++ Q++ L +
Sbjct  61    LVAHDSVEDCPNSL*VSAAVQVYSAG*RFEYIAENLGHFHVFSDHTILLVNQIQHIFLYI  120

Query  4617  VISFYLMFDEFHYRLLQRRVQLRAEVLVQSKFDGVGRERDVIDEGGSQVSHFAGLQFATV  4438
             ++    L+  E HY  L    VQL A V VQ+ FD +  E DV+DE  S +   +G+Q  T
Sbjct  121   MLPSLLVLYERHYSRL*AGVQLVA*VFVQAHFDNIRGESDVVDESRS*IR*VSGIQRGTA  180

Query  4437  LEDV*RHQEIDYGVPEELEALvrarqpvpdvrrvrqsv*QEG*IFERVV*DLFDISSRIV  4258
              +DV   + +I  GV E+L + V   Q V DVRRV +    +  I E ++  LF+I   +V
Sbjct  181   FKDVRSY*KIQNGVAEKL*SFVGTGQSVADVRRVGKRFK*KRWILEFIIEYLFNILPAVV  240

Query  4257  **KVNISFGS*ILR*RCLHRLVEHVCKVILWRKHLHPFK*FYSC*YPSTK*ICADQSFHG  4078
                V+    G  IL      +   E   K++LW K+  PF  F  +       +*I +   FH
Sbjct  241   HYSVHFGRGGLILGKNRWNLPGE*RLKIVLWWKNFEPF*HFDTAQDSIAE*ISSH**FHA  300

Query  4077  IGYRFVRNCL*MYIALHRILGTAFGFCLSDAIL---EIIHHRFPAFFT*FTGHYFLFGEL  3907
             +  RFVR+ L  Y+ LH  L T         ++   +++ ++  A F      +F + E
Sbjct  301   VRQRFVRHSLRSYVTLHCNLHTVERLLFQRRLIIS*QLLTYQLKALFGEVPT*HFFWREF  360

Query  3906  YAD*ECWTNLSLFNKFFQFRGRKETWDEEVTSRAELLHLHASTQVEFRTSVI*IPVTDQL  3727
              AD  C  +L +FN       R++  +E V+SR+EL H H +T+++F T V  +PV+++
Sbjct  361   *ADETCRPDLFIFNHLLHLFRREKPGNEVVSSRSELFHFHTTTELDFGTCVEQVPVSNES  420

Query  3726  ALDVR*LTLVHVRKLVVQVQAGGELQERVPQCLRSLQIEGVV*VDRGERGEHVSQFRTSP  3547
             +LDV  L+ +H  +LV+Q+    +LQE +     S Q+E +V V       +V+QFR S
```

```
Sbjct  421   SLDVGKLSFIHRWELVMQI*RSSKLQEGIS*RFGSFQVESIVRVYCSNCC*YVAQFRPSG  480

Query  3546  GRRLL*YFDFINVLDGRRCYSWGLRNDSrrgtrvvrcrryrsRHKLVVTFYVH*RDGRQV  3367
                L *YFDF   L  RR Y   L          V   R+RSR + VV FYVH    R V
Sbjct  481   RHFLF*YFDFHEFLYRRRQYGCELPRRRAVRRGSVGRDRHRSRDEFVVAFYVHHGYRRYV  540

Query  3366  TLYVDGFDSLIPGVLFVDFLSLQFI*ALF*RYNFLQTIICAF----VYSVNSVDMKW-FF  3202
              L V     LIP +LF   L LQ I AL   YN +    C F    VYSVN V M++ +
Sbjct  541   ALDVQRLGFLIPVILFAYLLFLQLIQALLHGYN-VHRPRCVFVNRVVYSVNIVRMRFIWL  599

Query  3201  NLGQ**IYGYIPVHLYRLK*FRFFQNWLPVFSGV*YFHEQFMCH*QHVNSVNNTGQ*RCL  3022
             +  Q  ++ +  +H YRL      Q     F     F  Q +CH*QH + V++T Q
Sbjct  600   DERQKGLHSHFNIHFYRLPLLSCRQMHDTDFCLPRDFSAQLICH*QHGHPVHDTRQHTRF  659

Query  3021  IPQYSWVIVFNAEFRYKYLCKSLRFIVAERNRYHLQLLVCERFE-RCCSG*EAVHPRAYV  2845
             +PQ +  IV + E  Y+ L K  + + A R   H  L   +  + R C  *     R
Sbjct  660   VPQSAREIVADTELWYENLWKPFKLVFA*RQGNHFHLFF*KSLDGRGCRQ*TVESTRG*P  719

Query  2844  TYLQQWLTFTS*YiqvivvciiicirfiavsIELRFRRHVFENLRHDVPFR*NSPAIPGR  2665
               L+Q L   S Y  V+ + I++ +R   +  +LR   HV +N+ + VP R N  +IP
Sbjct  720   LDLKQGLVLVSHYF*VVFIRIVVGVRSF*IRRKLRLGSHVLQNI*YYVPRRKNDSSIPRG  779

Query  2664  YNAIRVVFFCDYQRFSFLN*RRGDRWHRVVSLHCCPVNVVSIMFQY------RDNVHAVF  2503
             + AI V F  D  R SFL      R +  V LH  PV++V     +      R +V  V
Sbjct  780   HYAISVAFLRDT*RPSFLKRGLHHRPYLCVRLHNFPVDIVVQCACHVGSRSDRHSVKRVI  839

Query  2502  SSLEYQKLLIDRQDLVE*KIMELLH*LEYNRGQFYNCVIDFY*R-------GDFSNGRER  2344
              ++EY KLL+DR  LVE + +E  H       QF + VI F  R        D +
Sbjct  840   ETVEYGKLLLDR*HLVEYEPVEAFH-------QFKSLVIQFDCRIVHEQLGVDLAFSHAY  892

Query  2343  WELAQFRQRLLRQKPVAFVSLYKIVQ  2266
              ELA  RQ L  Q+   F  L + VQ
Sbjct  893   GELANPRQSLHCQQSETFPFLQESVQ  918


> ta_transcript86359_1
Length=683

 Score =   150 bits (379),  Expect = 2e-36, Method: Compositional matrix adjust.
 Identities = 153/528 (29%), Positives = 254/528 (48%), Gaps = 1/528 (0%)
 Frame = -1

Query  1809  ELIVW*NRAIPA**TNC*YGI*YIAIRRFVWGNLVVIIQVLMNVLSCGSGAAV-IADYEI  1633
             ELIVW*+R +P       +G     +   V  + V+++Q + ++L C S A + +A+  +
Sbjct  3     ELIVW*HRTVPVEEAYSLHG*QAGTVLCDVRRDFVLVVQRIFDILLCWSVAVLSVANQHL  62

Query  1632  TNTFFGFLERVVFY*ERLFVFNINEYNETGFRTSCTTFALDIRSIFGWRIVSDN*RTLRY  1453
              N  FG+LE +  + E + V  I++ +E    T CTTFALDIRS+F  R+VS +   LR
Sbjct  63    LNIMFGYLEALELFHEFVSVTLIDQQDEC*I*TPCTTFALDIRSVFERRVVSQHEHALRD  122

Query  1452  VQAFFDDARRYQTVQVSVIKSQNGflg*ff*imfFPCTFKGIDTEADNKIMFQKFKLVRS  1273
             V  F   A  YQTVQ  V + Q G L  F  I  FP  F  +  E +++++FQK +  R
Sbjct  123   VHTFLYHAC*YQTVQFLVRELQQGSLARFLRIKLFPGRFVRVYIEPNDEVVFQKAEFFRV  182

Query  1272  SFFQVH*DIIQNL*SKPLFRENYSTERFS*VVYFQQFSRLFQYQRVIFLQQVNQL*NLFR  1093
             SFF +   I+ +    +LFRE  S      +VY +   R     RV  LQ + Q *N F
Sbjct  183   SFFDILEKIVNDFSCESLFREYDSG*WLFSLVYSEMS*RFVVIFRVELLQNIKQQ*NFF*  242

Query  1092  *MFYYFIVPSLVFHGVQEYHASFCGMGDDS*IQQIflfc*cftf*sF*QDNVRQNTQRST  913
              M     +  +  H   +YH+    M + + +Q  FL    F F S *+  +R  T  +
Sbjct  243   RMSDDSFITLVAIH*S*KYHSCHSNMSNCNFVQMFFLRSNGFAFQSL*EY*IRDTT*LAA  302

Query  912   IQTHLFEICYHSYYKIFYRLHNLVRVVRNISLAWCLFKIF*WQRDRQLNTHHKIL*RIK*  733
             +++ LF   Y    +  + L++ + V  ++ L      +           H   L* +
Sbjct  303   VRSQLFNEHYQ**HHFTHFLYDFLWVSADVPLPPFQLNVRVASHGG*SYFHDDHL*FL*Y  362

Query  732   TYRFLSCFRHLRLPEVLYKLLGVGRVSHNFTKIINSCDGCMKRVFQLLKCFLDLRY*YIP  553
                F S     R     + L+ V RV+   T+ I+S +G MKR  Q    FL     ++
Sbjct  363   ADGFPSRRSRWRAAV*FHALIRVRRVAQYLTEFIDSGNGGMKRGLQFA*RFLHSIDIHVG  422

Query  552   IQ*CRR*T*NSGLFVSFTIQKVLHYNSHWMVFHSMMTFIKYNQINILDSYICMCKHVT*Y  373
             +Q C R + N  LF +    K+ H  SH MV   +M FI+ +++++    I + +HV+
Sbjct  423   VQKCCRQSQNPRLFFNRNTLKMFHDKSHGMVDDRVMAFIEDQKVDVMYPDIRVGQHVSQD  482

Query  372   LRGHYNYLVSCQFRVPIGFLPIVYPIFATIGTNTVDGTHHFNYIFLLI  229
             L  H+++L+  +  +P   +P+++ + + +   T+ GTH   + F ++
Sbjct  483   LTRHHDHLMF*ELGIPFILVPVIHAVLSAVSGYTLHGTHFLYHFFSVV  530
```

```
*************************************************************************************************
***********************************************************************
Query= gi|665399372|ref|NM_058088.4| Drosophila melanogaster drosha (drosha), mRNA

Length=4290
                                                                      Score     E
Sequences producing significant alignments:                          (Bits)  Value

  ta_transcript89214_1                                                 1231   0.0

> ta_transcript89214_1
Length=1538

 Score =  1231 bits (3185),  Expect = 0.0, Method: Compositional matrix adjust.
 Identities = 670/1139 (59%), Positives = 822/1139 (72%), Gaps = 62/1139 (5%)
 Frame = +1

Query  559   YEQRHDQEHRQIQDSRYAHEPRHGHYAHRQAKGSQHGYYGSAARNQVSDDYSP----RGH  726
             YE+RH   HR+ +     ++PR     HR                  S  YSP    R H
Sbjct  324   YERRHSL-HREHRSHSRVYDPRDERKYHRSP----------------SRKYSPGRDSRSH  366

Query  727   --HERERNETLEKTRAKPKVETERDRLLRQWCSNFCEKPEDYVKKMNALS-EADAPVES-  894
               H   R  T  ++ +K  + T+R+++L ++  N+C   +D ++KM   + E D+  E
Sbjct  367   SRHPSVRPSTRHESVSKRPL-TDREKILEEYRKNYCRTSDDIIEKMEQWTKEHDSDEEET  425

Query  895   --WVRSSPAELYYERTKSENEVRGRARLQKLCTLFDEELLQRAKRVREKLPVYVPPPRKA  1068
               W RSSPAELYY+  +    V   A+LQ+LC  F + L+ R ++ R ++   +PP +
Sbjct  426   KIWYRSSPAELYYKPVEGGAGVAQTAKLQRLCDAFQKHLIDRGRKARPEVDE-LPPLKPP  484

Query  1069  RRRVCKHKHKSEACssssssdddsdEDAFKIEQDCCMEELSRKVQHPQRVHADLWHNDAG  1248
             + ++CKH+ +  + SSS S D+  DED    +   D  M EL RK  HP+R+H ++W N+ G
Sbjct  485   KSKLCKHRLEEGSSSSSESEDECLDEDGLQGYTDRIMLELQRKQSHPRRLHPEMWFNNTG  544

Query  1249  EMNDGPLCRCSAKSRRIGIRHGIYPGETGYKLCDPNSNNAGKLFHYRISISPPTNFLTKT  1428
             EMN GPLCRCSA+++R G+RHG+Y GE   +  C P  +N  KL+HYRI++SPPTNFL K+
Sbjct  545   EMNGGPLCRCSARAKRHGMRHGVYAGEQTFPKCVPTQSNVDKLYHYRITVSPPTNFLIKS  604

Query  1429  PTIIKHDEHEFLFEGFSLLSHVRLSDLPVCKVIRFNIEYTIEYEEEKMPENFTIHELDIF  1608
             PTII+HDEHEFLF GFS+ SH +L+ LP CKVIRFNIEYTI Y EE  P+NF+I ELD+F
Sbjct  605   PTIIRHDEHEFLFSGFSMFSHYKLARLPTCKVIRFNIEYTILYIEEPPPQNFSIRELDLF  664

Query  1609  FKYLFHELLELVDFNLMPNLPSGNVEESCPAFHFFPRFVRDLPDNGKEVLAMVEVLRYLL  1788
              ++LF ELLEL+D +L       + + +C  FHF PRFVR LP+ G EVL+M EVL+YLL
Sbjct  665   EEFLFTELLELIDLDL-----GKSTDTACSQFHFIPRFVRSLPEGGCEVLSMCEVLKYLL  719

Query  1789  DNSAQLVERQQLLHLNQISQSEWQNYVDFIKGMLVTKPGYKPCSLRVDQLDRN---NSDL  1959
             D S  LV    L  ++ +    WQ +VD IKGM+VT PG KPCS+RVDQLDR+   N D
Sbjct  720   DESGLLVPPASLADIHDMDHYTWQKFVDRIKGMIVTYPGKKPCSVRVDQLDRSPPANEDG  779

Query  1960  PECVDRETGISHPAIVHFGICHPQLSYAGNPEYQKAWREYVKYRHLMANMSKPSFKDKRK  2139
                 D+     +P IVHFGI  PQLSYAGNPEYQKAWR YVKYRHL+ANM+KPSFK++++
Sbjct  780   RVPKDKNF---YPEIVHFGIRPPQLSYAGNPEYQKAWRYYVKYRHLIANMAKPSFKERQR  836

Query  2140  LEEKEQRLQEMRTQGRMKRNITVAISSEGFYRTGIMCDVVQHAMLIPVLTGHLRFHKSLD  2319
             L  K+ +LQEMRTQ +MKR++TVAISS+GF+ TG+MCDVVQHAMLIPVL  HLRFHKSL+
Sbjct  837   LAAKQAKLQEMRTQTKMKRDVTVAISSQGFHTTGLMCDVVQHAMLIPVLVRHLRFHKSLE  896

Query  2320  LLEESIGYRFKNRYLLQLALTHPSYKENYGTNPDHARNSLTNCGIRQPEYGDRKIHYMNT  2499
              LE  +GY F+ R LLQ ALTHPSY+EN+GTNPDHARNSLTNCGIRQPEYGDR+IHY  T
Sbjct  897   SLERKVGYTFRQRLLLQTALTHPSYRENFGTNPDHARNSLTNCGIRQPEYGDRRIHY--T  954

Query  2500  RKRGINTLVSIMSRFGKEHETVSNITHNERLEFLGDAVVEFLSSIHLFFMFPELEEGGLA  2679
             RK+GI TL++IMSRFGK  ET S I HNERLEFLGDAVVEFLSSIHLF MFP L EGGLA
Sbjct  955   RKKGIVTLINIMSRFGKLSETESEIKHNERLEFLGDAVVEFLSSIHLFRMFPGLAEGGLA  1014

Query  2680  TYRAAIVQNqhlallakklqleeFMLYAHGSDLCHELELRHAMANCFEALMGALLLDGGI  2859
             TYRA+IVQNQHLA LAK +QLE++MLYAHGSDLC E+ +RHAMANCFEALMGAL LD G+
Sbjct  1015  TYRASIVQNQHLAQLAKNIQLEQYMLYAHGSDLCREVVMRHAMANCFEALMGALFLDAGL  1074

Query  2860  KVADEVFTDALFRQDEKLLSIWKNLPEHPLQEQEPLGDRSCIDSYRVLKELTKFEDSIGI  3039
             KVAD VF+ AL+  + +LL +W     HPLQEQEPLGDR  I  +  L++LT+FE+SIG+
Sbjct  1075  KVADRVFSLALWYNEPELLHVWTKERSHPLQEQEPLGDRKYIKDFEFLQKLTEFEESIGV  1134

Query  3040  KFKHIRLLARAFTDRSIGFTHLTLGSNQRLEFLGDTVLQLICSEYLYRHFPEHHEGHlsl  3219
             +FKHIRLLARAFTDRS+GFTHLTLGSNQRLEFLGDTVLQL+ S+ LYRHFP+HHEGHLSL
Sbjct  1135  QFKHIRLLARAFTDRSVGFTHLTLGSNQRLEFLGDTVLQLVVSDRLYRHFPDHHEGHLSL  1194
```

```
Query  3220  lrsslVNNRTQAVVCDDLGMPKYAVYANPKADLKTKD-RADLLEAFLGALYVDKGLLYCE  3396
             LRSSLVNNRTQ++VCDDL M  YA+Y NPKA   TK  +ADLLEAFLGALYVDK L YC+
Sbjct  1195  LRSSLVNNRTQSMVCDDLNMASYAIYNNPKAKPTTKKHKADLLEAFLGALYVDKNLEYCQ  1254

Query  3397  QFCHVCLFPRLQLFIMNQDWNDPKSKLQQCCLTLRTMDGGEPDIPYYKVVEASGPTNTRV  3576
              FC+ CLFPRLQ FIMNQDWNDPKSKLQQCCLTLR+M+GGEPDIP YKV+E  GPTNTRV
Sbjct  1255  AFCNACLFPRLQEFIMNQDWNDPKSKLQQCCLTLRSMEGGEPDIPLYKVIECLGPTNTRV  1314

Query  3577  YKVAVYFRSKRLATSSGSSIQQAEMNAAKQALENSRDLFPQLDHQKRVIAKSIKKQTGNE  3756
             Y V VYFR  RLA + G SIQ+AEMNAA+ AL   + +LFPQLDHQKRVIAK +KK+
Sbjct  1315  YTVGVYFRGTRLAAARGHSIQEAEMNAAESALNTAHELFPQLDHQKRVIAKRMKKKKARG  1374

Query  3757  LDNDSDRQHQEEKIKRPKYATPLQDESHLPKQYRMHENISSDELPEDEDFESTAPKSPT  3933
              D  +D+  Q               E  +PK YR+      D  P D   E++    P+
Sbjct  1375  -DTTADKTKQNTL------------EDRVPKAYRL------DNQPSDSSDETSHSNPPS  1414

*********************************************************************************************
*******************************************************************
Query= gi|304307736|ref|NM_001195079.1| Bombyx mori loquacious (Loqs), mRNA
Length=1149
                                                                      Score     E
Sequences producing significant alignments:                          (Bits)  Value

  ta_transcript67781_1                                                 609   0.0
  ta_transcript67777_1                                                 612   0.0
  ta_transcript67782_1                                                 506   2e-170
  ta_transcript67778_1                                                 508   7e-167
  ta_transcript67779_1                                                 163   8e-43
  ta_transcript67775_1                                                 159   3e-41


> ta_transcript67781_1
Length=971

 Score =   609 bits (1571),  Expect = 0.0, Method: Compositional matrix adjust.
 Identities = 318/388 (82%), Positives = 343/388 (88%), Gaps = 13/388 (3%)
 Frame = +1

Query  1     MEGNTVHPHPSVVPGLPPGVIHGMTVHGSGPNGEHFPHGPRRRYQTRPKP-NNLQRLPLD  177
             M+G  VHPHP VV G  PGV+HGM VHG+GP+GEH PHGPRRRYQ RPKP NN++RLPLD
Sbjct  55    MDGGVVHPHPGVVHGSVPGVVHGMPVHGAGPDGEHAPHGPRRRYQNRPKPPNNVERLPLD  114

Query  178   EAAKRKMETLPTKTPVSVLQELLARRETVPKYELLQIEGMIHEPTFRYRVTVADLFAMGT  357
             EAAKR+ME+LP KTPVSVLQELLARR TVPKYEL+QIEGMIHEPTFRYRVTVADL AMGT
Sbjct  115   EAAKREMESLPMKTPVSVLQELLARRGTVPKYELVQIEGMIHEPTFRYRVTVADLVAMGT  174

Query  358   GRskkeakhsaakaLLDKLTGATPSDQTTNGSVPETGAVVPTFEDKLMGNPVGWLRSCVC  537
             GRSKKEAKHSAAKALLDKLTGATP+DQ TNG+VPETGAVV +FEDKLMGNPVGWL+  +C
Sbjct  175   GRSKKEAKHSAAKALLDKLTGATPADQGTNGNVPETGAVVTSFEDKLMGNPVGWLQE-LC  233

Query  538   HDSGH----HHLTMLKTMTMLIDQFQVCPHERQFTIICTLLKRREVGTGKSKKLAKRQAA  705
                      +H      +   +      PHERQFTI+CTLLKRRE+GTGKSKKLAKRQAA
Sbjct  234   MSRFWPPPSYHAENDDNVNRRL------PHERQFTIVCTLLKRREIGTGKSKKLAKRQAA  287

Query  706   YKMWQALQDNPPESFQNDDEGGVAARYADLKDSKISTLTTSHSHKVSQFHKHLKQSVGPN  885
             YKMWQALQDNPPESFQ D+EG +AARYADLKDSKISTLTTSHSHKVSQFHKHLKQSVGPN
Sbjct  288   YKMWQALQDNPPESFQPDEEG-MAARYADLKDSKISTLTTSHSHKVSQFHKHLKQSVGPN  346

Query  886   LAKLQVTPLNNKDFNFVQFLQEIASEQSFEVTYVDIEEKSMTGLCQCLVQLSTLPVAVCH  1065
             LAKLQVTPLNNKDFNFVQFLQEIASEQSFEVTYVDIEEKSM+G  QCLVQLSTLPVAVC+
Sbjct  347   LAKLQVTPLNNKDFNFVQFLQEIASEQSFEVTYVDIEEKSMSGRSQCLVQLSTLPVAVCY  406

Query  1066  GAGLTSKDAQSSAAQNALEYLKIMTKK*  1149
             GAGL+SKDAQ+SAAQNALEYLKIMTKK*
Sbjct  407   GAGLSSKDAQASAAQNALEYLKIMTKK*  434


> ta_transcript67777_1
Length=1407

 Score =   612 bits (1579),  Expect = 0.0, Method: Compositional matrix adjust.
 Identities = 318/388 (82%), Positives = 343/388 (88%), Gaps = 13/388 (3%)
 Frame = +1

Query  1     MEGNTVHPHPSVVPGLPPGVIHGMTVHGSGPNGEHFPHGPRRRYQTRPKP-NNLQRLPLD  177
             M+G  VHPHP VV G  PGV+HGM VHG+GP+GEH PHGPRRRYQ RPKP NN++RLPLD
```

```
Sbjct  55    MDGGVVHPHPGVVHGSVPGVVHGMPVHGAGPDGEHAPHGPRRRYQNRPKPPNNVERLPLD  114

Query  178   EAAKRKMETLPTKTPVSVLQELLARRETVPKYELLQIEGMIHEPTFRYRVTVADLFAMGT  357
             EAAKR+ME+LP KTPVSVLQELLARR TVPKYEL+QIEGMIHEPTFRYRVTVADL AMGT
Sbjct  115   EAAKREMESLPMKTPVSVLQELLARRGTVPKYELVQIEGMIHEPTFRYRVTVADLVAMGT  174

Query  358   GRskkeakhsaakaLLDKLTGATPSDQTTNGSVPETGAVVPTFEDKLMGNPVGWLRSCVC  537
             GRSKKEAKHSAAKALLDKLTGATP+DQ TNG+VPETGAVV +FEDKLMGNPVGWL+  +C
Sbjct  175   GRSKKEAKHSAAKALLDKLTGATPADQGTNGNVPETGAVVTSFEDKLMGNPVGWLQE-LC  233

Query  538   HDSGH----HHLTMLKTMTMLIDQFQVCPHERQFTIICTLLKRREVGTGKSKKLAKRQAA  705
                      +H      +   +      PHERQFTI+CTLLKRRE+GTGKSKKLAKRQAA
Sbjct  234   MSRFWPPPSYHAENDDNVNRRL------PHERQFTIVCTLLKRREIGTGKSKKLAKRQAA  287

Query  706   YKMWQALQDNPPESFQNDDEGGVAARYADLKDSKISTLTTSHSHKVSQFHKHLKQSVGPN  885
             YKMWQALQDNPPESFQ D+EG +AARYADLKDSKISTLTTSHSHKVSQFHKHLKQSVGPN
Sbjct  288   YKMWQALQDNPPESFQPDEEG-MAARYADLKDSKISTLTTSHSHKVSQFHKHLKQSVGPN  346

Query  886   LAKLQVTPLNNKDFNFVQFLQEIASEQSFEVTYVDIEEKSMTGLCQCLVQLSTLPVAVCH  1065
             LAKLQVTPLNNKDFNFVQFLQEIASEQSFEVTYVDIEEKSM+G  QCLVQLSTLPVAVC+
Sbjct  347   LAKLQVTPLNNKDFNFVQFLQEIASEQSFEVTYVDIEEKSMSGRSQCLVQLSTLPVAVCY  406

Query  1066  GAGLTSKDAQSSAAQNALEYLKIMTKK*  1149
             GAGL+SKDAQ+SAAQNALEYLKIMTKK*
Sbjct  407   GAGLSSKDAQASAAQNALEYLKIMTKK*  434


> ta_transcript67782_1
Length=927

 Score =   506 bits (1302),  Expect = 2e-170, Method: Compositional matrix adjust.
 Identities = 275/388 (71%), Positives = 299/388 (77%), Gaps = 57/388 (15%)
 Frame = +1

Query  1     MEGNTVHPHPSVVPGLPPGVIHGMTVHGSGPNGEHFPHGPRRRYQTRPKP-NNLQRLPLD  177
             M+G  VHPHP VV G  PGV+HGM VHG+GP+GEH PHGPRRRYQ RPKP NN++RLPLD
Sbjct  55    MDGGVVHPHPGVVHGSVPGVVHGMPVHGAGPDGEHAPHGPRRRYQNRPKPPNNVERLPLD  114

Query  178   EAAKRKMETLPTKTPVSVLQELLARRETVPKYELLQIEGMIHEPTFRYRVTVADLFAMGT  357
             EAAKR+ME+LP KTPVSVLQELLARR TVPKYEL+QIEGMIHEPTFRYRVTVADL AMGT
Sbjct  115   EAAKREMESLPMKTPVSVLQELLARRGTVPKYELVQIEGMIHEPTFRYRVTVADLVAMGT  174

Query  358   GRskkeakhsaakaLLDKLTGATPSDQTTNGSVPETGAVVPTFEDKLMGNPVGWLRSCVC  537
             GRSKKEAKHSAAKALLDKLTGATP+DQ TNG+VPETGAVV +FEDKLMGNPVGWL+  +C
Sbjct  175   GRSKKEAKHSAAKALLDKLTGATPADQGTNGNVPETGAVVTSFEDKLMGNPVGWLQE-LC  233

Query  538   HDSGH----HHLTMLKTMTMLIDQFQVCPHERQFTIICTLLKRREVGTGKSKKLAKRQAA  705
                      +H      +   +      PHERQFTI+CTLLKRRE+GTGKSKKLAKRQAA
Sbjct  234   MSRFWPPPSYHAENDDNVNRRL------PHERQFTIVCTLLKRREIGTGKSKKLAKRQAA  287

Query  706   YKMWQALQDNPPESFQNDDEGGVAARYADLKDSKISTLTTSHSHKVSQFHKHLKQSVGPN  885
             YKMWQALQDNPPESFQ D+E
Sbjct  288   YKMWQALQDNPPESFQPDEE----------------------------------------  307

Query  886   LAKLQVTPLNNKDFNFVQFLQEIASEQSFEVTYVDIEEKSMTGLCQCLVQLSTLPVAVCH  1065
                  VTPLNNKDFNFVQFLQEIASEQSFEVTYVDIEEKSM+G  QCLVQLSTLPVAVC+
Sbjct  308   -----VTPLNNKDFNFVQFLQEIASEQSFEVTYVDIEEKSMSGRSQCLVQLSTLPVAVCY  362

Query  1066  GAGLTSKDAQSSAAQNALEYLKIMTKK*  1149
             GAGL+SKDAQ+SAAQNALEYLKIMTKK*
Sbjct  363   GAGLSSKDAQASAAQNALEYLKIMTKK*  390


> ta_transcript67778_1
Length=1363

 Score =   508 bits (1307),  Expect = 7e-167, Method: Compositional matrix adjust.
 Identities = 275/388 (71%), Positives = 299/388 (77%), Gaps = 57/388 (15%)
 Frame = +1

Query  1     MEGNTVHPHPSVVPGLPPGVIHGMTVHGSGPNGEHFPHGPRRRYQTRPKP-NNLQRLPLD  177
             M+G  VHPHP VV G  PGV+HGM VHG+GP+GEH PHGPRRRYQ RPKP NN++RLPLD
Sbjct  55    MDGGVVHPHPGVVHGSVPGVVHGMPVHGAGPDGEHAPHGPRRRYQNRPKPPNNVERLPLD  114

Query  178   EAAKRKMETLPTKTPVSVLQELLARRETVPKYELLQIEGMIHEPTFRYRVTVADLFAMGT  357
             EAAKR+ME+LP KTPVSVLQELLARR TVPKYEL+QIEGMIHEPTFRYRVTVADL AMGT
Sbjct  115   EAAKREMESLPMKTPVSVLQELLARRGTVPKYELVQIEGMIHEPTFRYRVTVADLVAMGT  174
```

```
Query  358   GRskkeakhsaakaLLDKLTGATPSDQTTNGSVPETGAVVPTFEDKLMGNPVGWLRSCVC  537
             GRSKKEAKHSAAKALLDKLTGATP+DQ TNG+VPETGAVV +FEDKLMGNPVGWL+  +C
Sbjct  175   GRSKKEAKHSAAKALLDKLTGATPADQGTNGNVPETGAVVTSFEDKLMGNPVGWLQE-LC  233

Query  538   HDSGH----HHLTMLKTMTMLIDQFQVCPHERQFTIICTLLKRREVGTGKSKKLAKRQAA  705
                      +H      +   +      PHERQFTI+CTLLKRRE+GTGKSKKLAKRQAA
Sbjct  234   MSRFWPPPSYHAENDDNVNRRL------PHERQFTIVCTLLKRREIGTGKSKKLAKRQAA  287

Query  706   YKMWQALQDNPPESFQNDDEGGVAARYADLKDSKISTLTTSHSHKVSQFHKHLKQSVGPN  885
             YKMWQALQDNPPESFQ D+E
Sbjct  288   YKMWQALQDNPPESFQPDEE----------------------------------------  307

Query  886   LAKLQVTPLNNKDFNFVQFLQEIASEQSFEVTYVDIEEKSMTGLCQCLVQLSTLPVAVCH  1065
                  VTPLNNKDFNFVQFLQEIASEQSFEVTYVDIEEKSM+G  QCLVQLSTLPVAVC+
Sbjct  308   -----VTPLNNKDFNFVQFLQEIASEQSFEVTYVDIEEKSMSGRSQCLVQLSTLPVAVCY  362

Query  1066  GAGLTSKDAQSSAAQNALEYLKIMTKK*  1149
             GAGL+SKDAQ+SAAQNALEYLKIMTKK*
Sbjct  363   GAGLSSKDAQASAAQNALEYLKIMTKK*  390


> ta_transcript67779_1
Length=1051

 Score =   163 bits (412),  Expect = 8e-43, Method: Compositional matrix adjust.
 Identities = 132/326 (40%), Positives = 181/326 (56%), Gaps = 14/326 (4%)
 Frame = +3

Query  186   QKENGNTSYKDSCLCFARAVGTSGNCS*I*ttpdrrhdt*thFPVQSNCG*FICNGHWSL  365
             Q+ NG ++ +DS +  AR  G +   + + T  DR HD+* HFP+Q +  *  C GHW+
Sbjct  198   QEGNGISTNEDSGVSTARTAGETWYRAQVRTGADRGHDS*AHFPLQGHSC*SCCYGHWTF  257

Query  366   KKRGKALCSQGFTGQVNWCYPI*SDYQWQCP*NWCCSTYI*R*TNG*SCWMASE-----L  530
             K+RG+AL   G  GQ +WCY  *S  + +C  NWC    I*R  +G    +A+      L
Sbjct  258   KERGQALGC*GSPGQTDWCYSC*SRNKRKCTRNWCSGYVI*RQADGQPGGLAARAVHVSL  317

Query  531   CMSRFWPPPSYHAENDDNVNRPVSGLPP*TSIYDHLHIAQAP*SWYRQVKEIS*TASCLQ  710
               S F P          + +R          ++  +H A+A    +RQV+E     S LQ
Sbjct  318   LASSFIPC*KRRQRQPSSASR--------APVHHCVHSAEAAGDRHRQVQEACQAPSRLQ  369

Query  711   DVAGLTGQPTRILPE***GRCCGPLCRFER**NLHIDHEP*P*SVAVPQTSQTVCWPQPG  890
             DVA   GQP+ ILP ** G   G LCR ER**NLH D++  P S+AVPQT QT+  PQPG
Sbjct  370   DVASSAGQPSGILPA**RGNG-GSLCRPER**NLHTDYQSQPQSLAVPQTPQTIRGPQPG  428

Query  891   QVAGDTTKQ*GF*LRSVLARNSIRAIF*GDLCRHRRKVYDWSMSVSCPAFHIASSCMPRS  1070
             QVAGD+ +Q G  LR+V A + + A+  GD+  HR +V   +++V   A H A   + R
Sbjct  429   QVAGDSAEQQGLQLRAVPAGDRVGAVVRGDVRGHRGEVDVGALAVPRAAVHAARGRVLRR  488

Query  1071  RPHQQGCPVICCSERLGVSQDYDQKV  1148
             RP QQG   +  +ER  V QD+DQ+V
Sbjct  489   RPLQQGRAGLRRAERARVPQDHDQEV  514


> ta_transcript67775_1
Length=1487

 Score =   159 bits (402),  Expect = 3e-41, Method: Compositional matrix adjust.
 Identities = 132/326 (40%), Positives = 181/326 (56%), Gaps = 14/326 (4%)
 Frame = +3

Query  186   QKENGNTSYKDSCLCFARAVGTSGNCS*I*ttpdrrhdt*thFPVQSNCG*FICNGHWSL  365
             Q+ NG ++ +DS +  AR  G +   + + T  DR HD+* HFP+Q +  *  C GHW+
Sbjct  198   QEGNGISTNEDSGVSTARTAGETWYRAQVRTGADRGHDS*AHFPLQGHSC*SCCYGHWTF  257

Query  366   KKRGKALCSQGFTGQVNWCYPI*SDYQWQCP*NWCCSTYI*R*TNG*SCWMASE-----L  530
             K+RG+AL   G  GQ +WCY  *S  + +C  NWC    I*R  +G    +A+      L
Sbjct  258   KERGQALGC*GSPGQTDWCYSC*SRNKRKCTRNWCSGYVI*RQADGQPGGLAARAVHVSL  317

Query  531   CMSRFWPPPSYHAENDDNVNRPVSGLPP*TSIYDHLHIAQAP*SWYRQVKEIS*TASCLQ  710
               S F P          + +R          ++  +H A+A    +RQV+E     S LQ
Sbjct  318   LASSFIPC*KRRQRQPSSASR--------APVHHCVHSAEAAGDRHRQVQEACQAPSRLQ  369

Query  711   DVAGLTGQPTRILPE***GRCCGPLCRFER**NLHIDHEP*P*SVAVPQTSQTVCWPQPG  890
             DVA   GQP+ ILP ** G   G LCR ER**NLH D++  P S+AVPQT QT+  PQPG
Sbjct  370   DVASSAGQPSGILPA**RGNG-GSLCRPER**NLHTDYQSQPQSLAVPQTPQTIRGPQPG  428
```

```
Query  891   QVAGDTTKQ*GF*LRSVLARNSIRAIF*GDLCRHRRKVYDWSMSVSCPAFHIASSCMPRS  1070
             QVAGD+ +Q G  LR+V A + + A+  GD+  HR +V   +++V   A H A   + R
Sbjct  429   QVAGDSAEQQGLQLRAVPAGDRVGAVVRGDVRGHRGEVDVGALAVPRAAVHAARGRVLRR  488

Query  1071  RPHQQGCPVICCSERLGVSQDYDQKV  1148
             RP QQG   +  +ER  V QD+DQ+V
Sbjct  489   RPLQQGRAGLRRAERARVPQDHDQEV  514


************************************************************************************************
*******************************************************************
Query= gi|300669730|dbj|AB566385.1| Bombyx mori R2D2 mRNA for R2D2,complete cds

Length=1023
                                                                      Score     E
Sequences producing significant alignments:                          (Bits)  Value

  ta_transcript64766_1                                                  276   4e-87
  ta_transcript64768_1                                                  275   6e-87
  ta_transcript64758_1                                                  276   7e-87
  ta_transcript64763_1                                                  275   9e-87
  ta_transcript64759_1                                                  276   1e-86
  ta_transcript64764_1                                                  275   1e-86
  ta_transcript64762_1                                                  275   2e-86
  ta_transcript64751_1                                                  283   2e-84
  ta_transcript64749_1                                                  283   2e-84
  ta_transcript64767_1                                                  268   3e-84
  ta_transcript64769_1                                                  267   4e-84
  ta_transcript64761_1                                                  269   4e-84
  ta_transcript64760_1                                                  269   6e-84
  ta_transcript64765_1                                                  268   7e-84
  ta_transcript64753_1                                                  280   2e-83
  ta_transcript64755_1                                                  276   6e-82
  ta_transcript64752_1                                                  276   7e-82
  ta_transcript64750_1                                                  276   7e-82
  ta_transcript64747_1                                                  273   4e-81
  ta_transcript64757_1                                                  273   6e-81
  ta_transcript64756_1                                                  273   6e-81
  ta_transcript64754_1                                                  273   8e-81
  ta_transcript64748_1                                                  266   1e-78


> ta_transcript64766_1
Length=505

 Score =   276 bits (705),  Expect = 4e-87, Method: Compositional matrix adjust.
 Identities = 160/328 (49%), Positives = 203/328 (62%), Gaps = 45/328 (14%)
 Frame = +1

Query  4    KTPITVLQEMMMKLGQIPEYECVAQSGPQHQATFEFRCKALGESVSASARSKREAKQEAA  183
            KT +TVLQE+M+K+G  PEY+CV+QSGPQH +TFE+RC+ALGE V+A+ARSK+EAKQE A
Sbjct  77   KTAVTVLQELMVKMGHTPEYDCVSQSGPQHLSTFEYRCQALGELVTATARSKKEAKQEVA  136

Query  184  RAMLLCLSTIGHRVPPPFATEFTQPSHSNQ-SAGECSEGKAPTVDSRSYVALLKELCEEY  360
            + ML  L+  GHRVPPP+ +    P  S Q S+    E +   V S SYV LLK+LCEEY
Sbjct  137  KLMLFQLARRGHRVPPPYNS---GPQLSTQQSSASKKEAEPSIVGSHSYVVLLKDLCEEY  193

Query  361  KLPGVEYALVADTGPAHMRLFSVRASIGLHSRDASGTTKRQARQKAAADLYLFLRXNLSR  540
             LPGVEY L+ DTGP HMR F++R  IG H R A+ TTK+ ARQ AA  LY ++R NL R
Sbjct  194  HLPGVEYTLIGDTGPPHMREFTIRVRIGQHERTATSTTKKAARQMAAEQLYKYMRENLHR  253

Query  541  LTHDFVEEQALVRAHERAMERLVETTPAP--WKPDLGQRVSEYHYGLISHTGECTARSRR  714
            +T DF EE+ALVRAHERAM+R VE       W+PDLGQ++++Y  GL +H
Sbjct  254  VTKDFTEEEALVRAHERAMDRYVEMRDPELVWRPDLGQKIADYPIGLKTH----------  303

Query  715  RLPVVLDQCLRRAMVDTDPEKRMAARAVLSETDDIeea--------aeraaaalelQTCW  870
                             DPEK   AR VLS  D+I+ +            AAAL L   W
Sbjct  304  ----------------IDPEKCQLARTVLSSLDEIQSSDPQVVEAALTATAAALGLTINW  347

Query  871  ETLD----PLHVLKLD-AAPALAFSGHS  939
              L      L +L+L   +PALAF+G +
Sbjct  348  SELQAERGKLQMLELTPTSPALAFAGEN  375


> ta_transcript64768_1
Length=485
```

```
 Score =   275 bits (702),  Expect = 6e-87, Method: Compositional matrix adjust.
 Identities = 160/328 (49%), Positives = 203/328 (62%), Gaps = 45/328 (14%)
 Frame = +1

Query  4    KTPITVLQEMMMKLGQIPEYECVAQSGPQHQATFEFRCKALGESVSASARSKREAKQEAA  183
            KT +TVLQE+M+K+G  PEY+CV+QSGPQH +TFE+RC+ALGE V+A+ARSK+EAKQE A
Sbjct  77   KTAVTVLQELMVKMGHTPEYDCVSQSGPQHLSTFEYRCQALGELVTATARSKKEAKQEVA  136

Query  184  RAMLLCLSTIGHRVPPPFATEFTQPSHSNQ-SAGECSEGKAPTVDSRSYVALLKELCEEY  360
            + ML  L+  GHRVPPP+ +    P  S Q S+    E +   V S SYV LLK+LCEEY
Sbjct  137  KLMLFQLARRGHRVPPPYNS---GPQLSTQQSSASKKEAEPSIVGSHSYVVLLKDLCEEY  193

Query  361  KLPGVEYALVADTGPAHMRLFSVRASIGLHSRDASGTTKRQARQKAAADLYLFLRXNLSR  540
             LPGVEY L+ DTGP HMR F++R  IG H R A+ TTK+ ARQ AA  LY ++R NL R
Sbjct  194  HLPGVEYTLIGDTGPPHMREFTIRVRIGQHERTATSTTKKAARQMAAEQLYKYMRENLHR  253

Query  541  LTHDFVEEQALVRAHERAMERLVETTPAP--WKPDLGQRVSEYHYGLISHTGECTARSRR  714
            +T DF EE+ALVRAHERAM+R VE       W+PDLGQ++++Y  GL +H
Sbjct  254  VTKDFTEEEALVRAHERAMDRYVEMRDPELVWRPDLGQKIADYPIGLKTH----------  303

Query  715  RLPVVLDQCLRRAMVDTDPEKRMAARAVLSETDDIeea--------aeraaaalelQTCW  870
                             DPEK   AR VLS  D+I+ +            AAAL L   W
Sbjct  304  ----------------IDPEKCQLARTVLSSLDEIQSSDPQVVEAALTATAAALGLTINW  347

Query  871  ETLD----PLHVLKLD-AAPALAFSGHS  939
              L      L +L+L   +PALAF+G +
Sbjct  348  SELQAERGKLQMLELTPTSPALAFAGEN  375


> ta_transcript64758_1
Length=540

 Score =   276 bits (706),  Expect = 7e-87, Method: Compositional matrix adjust.
 Identities = 160/328 (49%), Positives = 203/328 (62%), Gaps = 45/328 (14%)
 Frame = +1

Query  4    KTPITVLQEMMMKLGQIPEYECVAQSGPQHQATFEFRCKALGESVSASARSKREAKQEAA  183
            KT +TVLQE+M+K+G  PEY+CV+QSGPQH +TFE+RC+ALGE V+A+ARSK+EAKQE A
Sbjct  77   KTAVTVLQELMVKMGHTPEYDCVSQSGPQHLSTFEYRCQALGELVTATARSKKEAKQEVA  136

Query  184  RAMLLCLSTIGHRVPPPFATEFTQPSHSNQ-SAGECSEGKAPTVDSRSYVALLKELCEEY  360
            + ML  L+  GHRVPPP+ +    P  S Q S+    E +   V S SYV LLK+LCEEY
Sbjct  137  KLMLFQLARRGHRVPPPYNS---GPQLSTQQSSASKKEAEPSIVGSHSYVVLLKDLCEEY  193

Query  361  KLPGVEYALVADTGPAHMRLFSVRASIGLHSRDASGTTKRQARQKAAADLYLFLRXNLSR  540
             LPGVEY L+ DTGP HMR F++R  IG H R A+ TTK+ ARQ AA  LY ++R NL R
Sbjct  194  HLPGVEYTLIGDTGPPHMREFTIRVRIGQHERTATSTTKKAARQMAAEQLYKYMRENLHR  253

Query  541  LTHDFVEEQALVRAHERAMERLVETTPAP--WKPDLGQRVSEYHYGLISHTGECTARSRR  714
            +T DF EE+ALVRAHERAM+R VE       W+PDLGQ++++Y  GL +H
Sbjct  254  VTKDFTEEEALVRAHERAMDRYVEMRDPELVWRPDLGQKIADYPIGLKTH----------  303

Query  715  RLPVVLDQCLRRAMVDTDPEKRMAARAVLSETDDIeea--------aeraaaalelQTCW  870
                             DPEK   AR VLS  D+I+ +            AAAL L   W
Sbjct  304  ----------------IDPEKCQLARTVLSSLDEIQSSDPQVVEAALTATAAALGLTINW  347

Query  871  ETLD----PLHVLKLD-AAPALAFSGHS  939
              L      L +L+L   +PALAF+G +
Sbjct  348  SELQAERGKLQMLELTPTSPALAFAGEN  375


> ta_transcript64763_1
Length=520

 Score =   275 bits (704),  Expect = 9e-87, Method: Compositional matrix adjust.
 Identities = 160/328 (49%), Positives = 203/328 (62%), Gaps = 45/328 (14%)
 Frame = +1

Query  4    KTPITVLQEMMMKLGQIPEYECVAQSGPQHQATFEFRCKALGESVSASARSKREAKQEAA  183
            KT +TVLQE+M+K+G  PEY+CV+QSGPQH +TFE+RC+ALGE V+A+ARSK+EAKQE A
Sbjct  77   KTAVTVLQELMVKMGHTPEYDCVSQSGPQHLSTFEYRCQALGELVTATARSKKEAKQEVA  136

Query  184  RAMLLCLSTIGHRVPPPFATEFTQPSHSNQ-SAGECSEGKAPTVDSRSYVALLKELCEEY  360
            + ML  L+  GHRVPPP+ +    P  S Q S+    E +   V S SYV LLK+LCEEY
Sbjct  137  KLMLFQLARRGHRVPPPYNS---GPQLSTQQSSASKKEAEPSIVGSHSYVVLLKDLCEEY  193

Query  361  KLPGVEYALVADTGPAHMRLFSVRASIGLHSRDASGTTKRQARQKAAADLYLFLRXNLSR  540
```

```
             LPGVEY L+ DTGP HMR F++R  IG H R A+ TTK+ ARQ AA  LY ++R NL R
Sbjct  194  HLPGVEYTLIGDTGPPHMREFTIRVRIGQHERTATSTTKKAARQMAAEQLYKYMRENLHR  253

Query  541  LTHDFVEEQALVRAHERAMERLVETTPAP--WKPDLGQRVSEYHYGLISHTGECTARSRR  714
            +T DF EE+ALVRAHERAM+R VE       W+PDLGQ++++Y  GL +H
Sbjct  254  VTKDFTEEEALVRAHERAMDRYVEMRDPELVWRPDLGQKIADYPIGLKTH----------  303

Query  715  RLPVVLDQCLRRAMVDTDPEKRMAARAVLSETDDIeea--------aeraaaalelQTCW  870
                             DPEK   AR VLS  D+I+ +            AAAL L   W
Sbjct  304  ----------------IDPEKCQLARTVLSSLDEIQSSDPQVVEAALTATAAALGLTINW  347

Query  871  ETLD----PLHVLKLD-AAPALAFSGHS  939
              L      L +L+L   +PALAF+G +
Sbjct  348  SELQAERGKLQMLELTPTSPALAFAGEN  375
```

```
> ta_transcript64759_1
Length=539

 Score =   276 bits (705),  Expect = 1e-86, Method: Compositional matrix adjust.
 Identities = 160/328 (49%), Positives = 203/328 (62%), Gaps = 45/328 (14%)
 Frame = +1

Query  4    KTPITVLQEMMMKLGQIPEYECVAQSGPQHQATFEFRCKALGESVSASARSKREAKQEAA  183
            KT +TVLQE+M+K+G  PEY+CV+QSGPQH +TFE+RC+ALGE V+A+ARSK+EAKQE A
Sbjct  77   KTAVTVLQELMVKMGHTPEYDCVSQSGPQHLSTFEYRCQALGELVTATARSKKEAKQEVA  136

Query  184  RAMLLCLSTIGHRVPPPFATEFTQPSHSNQ-SAGECSEGKAPTVDSRSYVALLKELCEEY  360
            + ML  L+  GHRVPPP+ +    P  S Q S+    E +   V S SYV LLK+LCEEY
Sbjct  137  KLMLFQLARRGHRVPPPYNS---GPQLSTQQSSASKKEAEPSIVGSHSYVVLLKDLCEEY  193

Query  361  KLPGVEYALVADTGPAHMRLFSVRASIGLHSRDASGTTKRQARQKAADLYLFLRXNLSR  540
             LPGVEY L+ DTGP HMR F++R  IG H R A+ TTK+ ARQ AA  LY ++R NL R
Sbjct  194  HLPGVEYTLIGDTGPPHMREFTIRVRIGQHERTATSTTKKAARQMAAEQLYKYMRENLHR  253

Query  541  LTHDFVEEQALVRAHERAMERLVETTPAP--WKPDLGQRVSEYHYGLISHTGECTARSRR  714
            +T DF EE+ALVRAHERAM+R VE       W+PDLGQ++++Y  GL +H
Sbjct  254  VTKDFTEEEALVRAHERAMDRYVEMRDPELVWRPDLGQKIADYPIGLKTH----------  303

Query  715  RLPVVLDQCLRRAMVDTDPEKRMAARAVLSETDDIeea--------aeraaaalelQTCW  870
                             DPEK   AR VLS  D+I+ +            AAAL L   W
Sbjct  304  ----------------IDPEKCQLARTVLSSLDEIQSSDPQVVEAALTATAAALGLTINW  347

Query  871  ETLD----PLHVLKLD-AAPALAFSGHS  939
              L      L +L+L   +PALAF+G +
Sbjct  348  SELQAERGKLQMLELTPTSPALAFAGEN  375
```

```
> ta_transcript64764_1
Length=519

 Score =   275 bits (703),  Expect = 1e-86, Method: Compositional matrix adjust.
 Identities = 160/328 (49%), Positives = 203/328 (62%), Gaps = 45/328 (14%)
 Frame = +1

Query  4    KTPITVLQEMMMKLGQIPEYECVAQSGPQHQATFEFRCKALGESVSASARSKREAKQEAA  183
            KT +TVLQE+M+K+G  PEY+CV+QSGPQH +TFE+RC+ALGE V+A+ARSK+EAKQE A
Sbjct  77   KTAVTVLQELMVKMGHTPEYDCVSQSGPQHLSTFEYRCQALGELVTATARSKKEAKQEVA  136

Query  184  RAMLLCLSTIGHRVPPPFATEFTQPSHSNQ-SAGECSEGKAPTVDSRSYVALLKELCEEY  360
            + ML  L+  GHRVPPP+ +    P  S Q S+    E +   V S SYV LLK+LCEEY
Sbjct  137  KLMLFQLARRGHRVPPPYNS---GPQLSTQQSSASKKEAEPSIVGSHSYVVLLKDLCEEY  193

Query  361  KLPGVEYALVADTGPAHMRLFSVRASIGLHSRDASGTTKRQARQKAADLYLFLRXNLSR  540
             LPGVEY L+ DTGP HMR F++R  IG H R A+ TTK+ ARQ AA  LY ++R NL R
Sbjct  194  HLPGVEYTLIGDTGPPHMREFTIRVRIGQHERTATSTTKKAARQMAAEQLYKYMRENLHR  253

Query  541  LTHDFVEEQALVRAHERAMERLVETTPAP--WKPDLGQRVSEYHYGLISHTGECTARSRR  714
            +T DF EE+ALVRAHERAM+R VE       W+PDLGQ++++Y  GL +H
Sbjct  254  VTKDFTEEEALVRAHERAMDRYVEMRDPELVWRPDLGQKIADYPIGLKTH----------  303

Query  715  RLPVVLDQCLRRAMVDTDPEKRMAARAVLSETDDIeea--------aeraaaalelQTCW  870
                             DPEK   AR VLS  D+I+ +            AAAL L   W
Sbjct  304  ----------------IDPEKCQLARTVLSSLDEIQSSDPQVVEAALTATAAALGLTINW  347

Query  871  ETLD----PLHVLKLD-AAPALAFSGHS  939
              L      L +L+L   +PALAF+G +
```

```
Sbjct  348  SELQAERGKLQMLELTPTSPALAFAGEN  375


> ta_transcript64762_1
Length=523

 Score =   275 bits (703),  Expect = 2e-86, Method: Compositional matrix adjust.
 Identities = 160/328 (49%), Positives = 203/328 (62%), Gaps = 45/328 (14%)
 Frame = +1

Query  4    KTPITVLQEMMMKLGQIPEYECVAQSGPQHQATFEFRCKALGESVSASARSKREAKQEAA  183
            KT +TVLQE+M+K+G  PEY+CV+QSGPQH +TFE+RC+ALGE V+A+ARSK+EAKQE A
Sbjct  77   KTAVTVLQELMVKMGHTPEYDCVSQSGPQHLSTFEYRCQALGELVTATARSKKEAKQEVA  136

Query  184  RAMLLCLSTIGHRVPPPFATEFTQPSHSNQ-SAGECSEGKAPTVDSRSYVALLKELCEEY  360
            + ML  L+  GHRVPPP+ +    P  S Q S+    E +   V S SYV LLK+LCEEY
Sbjct  137  KLMLFQLARRGHRVPPPYNS---GPQLSTQQSSASKKEAEPSIVGSHSYVVLLKDLCEEY  193

Query  361  KLPGVEYALVADTGPAHMRLFSVRASIGLHSRDASGTTKRQARQKAAADLYLFLRXNLSR  540
             LPGVEY L+ DTGP HMR F++R  IG H R A+ TTK+ ARQ AA  LY ++R NL R
Sbjct  194  HLPGVEYTLIGDTGPPHMREFTIRVRIGQHERTATSTTKKAARQMAAEQLYKYMRENLHR  253

Query  541  LTHDFVEEQALVRAHERAMERLVETTPAP--WKPDLGQRVSEYHYGLISHTGECTARSRR  714
            +T DF EE+ALVRAHERAM+R VE       W+PDLGQ++++Y  GL +H
Sbjct  254  VTKDFTEEEALVRAHERAMDRYVEMRDPELVWRPDLGQKIADYPIGLKTH----------  303

Query  715  RLPVVLDQCLRRAMVDTDPEKRMAARAVLSETDDIeea--------aeraaaalelQTCW  870
                             DPEK   AR VLS  D+I+ +            AAAL L   W
Sbjct  304  ----------------IDPEKCQLARTVLSSLDEIQSSDPQVVEAALTATAAALGLTINW  347

Query  871  ETLD----PLHVLKLD-AAPALAFSGHS  939
              L      L +L+L   +PALAF+G +
Sbjct  348  SELQAERGKLQMLELTPTSPALAFAGEN  375


> ta_transcript64751_1
Length=1749

 Score =   283 bits (723),  Expect = 2e-84, Method: Composition-based stats.
 Identities = 157/328 (48%), Positives = 201/328 (61%), Gaps = 45/328 (14%)
 Frame = +1

Query  4    KTPITVLQEMMMKLGQIPEYECVAQSGPQHQATFEFRCKALGESVSASARSKREAKQEAA  183
            KT +TVLQE+M+K+G  PEY+CV+QSGPQH +TFE+RC+ALGE V+A+ARSK+EAKQE A
Sbjct  77   KTAVTVLQELMVKMGHTPEYDCVSQSGPQHLSTFEYRCQALGELVTATARSKKEAKQEVA  136

Query  184  RAMLLCLSTIGHRVPPPFATEFTQPSHS-NQSAGECSEGKAPTVDSRSYVALLKELCEEY  360
            + ML  L+  GHRVPPP+ +    P  S  QS+    E +   V S SYV LLK+LCEEY
Sbjct  137  KLMLFQLARRGHRVPPPYNS---GPQLSTQQSSASKKEAEPSIVGSHSYVVLLKDLCEEY  193

Query  361  KLPGVEYALVADTGPAHMRLFSVRASIGLHSRDASGTTKRQARQKAAADLYLFLRXNLSR  540
             LPGVEY L+ DTGP HMR F++R  IG H R A+ TTK+ ARQ AA  LY ++R NL R
Sbjct  194  HLPGVEYTLIGDTGPPHMREFTIRVRIGQHERTATSTTKKAARQMAAEQLYKYMRENLHR  253

Query  541  LTHDFVEEQALVRAHERAMERLVETTPAP--WKPDLGQRVSEYHYGLISHTGECTARSRR  714
            +T DF EE+ALVRAHERAM+R VE       W+PDLGQ++++Y  GL +H
Sbjct  254  VTKDFTEEEALVRAHERAMDRYVEMRDPELVWRPDLGQKIADYPIGLKTH----------  303

Query  715  RLPVVLDQCLRRAMVDTDPEKRMAARAVLSETDDIeeaaeraaaalelQTC--------W  870
                             DPEK   AR VLS  D+I+ +  +   A    T         W
Sbjct  304  ----------------IDPEKCQLARTVLSSLDEIQSSDPQVVEAALTATAAALGLTINW  347

Query  871  ETLDP----LHVLKLD-AAPALAFSGHS  939
              L      L +L+L   +PALAF+G +
Sbjct  348  SELQAERGKLQMLELTPTSPALAFAGEN  375


> ta_transcript64749_1
Length=1762

 Score =   283 bits (723),  Expect = 2e-84, Method: Composition-based stats.
 Identities = 157/328 (48%), Positives = 201/328 (61%), Gaps = 45/328 (14%)
 Frame = +1

Query  4    KTPITVLQEMMMKLGQIPEYECVAQSGPQHQATFEFRCKALGESVSASARSKREAKQEAA  183
            KT +TVLQE+M+K+G  PEY+CV+QSGPQH +TFE+RC+ALGE V+A+ARSK+EAKQE A
Sbjct  77   KTAVTVLQELMVKMGHTPEYDCVSQSGPQHLSTFEYRCQALGELVTATARSKKEAKQEVA  136
```

```
Query  184  RAMLLCLSTIGHRVPPPFATEFTQPSHS-NQSAGECSEGKAPTVDSRSYVALLKELCEEY  360
            + ML  L+  GHRVPPP+ +    P  S  QS+    E +   V S SYV LLK+LCEEY
Sbjct  137  KLMLFQLARRGHRVPPPYNS---GPQLSTQQSSASKKEAEPSIVGSHSYVVLLKDLCEEY  193

Query  361  KLPGVEYALVADTGPAHMRLFSVRASIGLHSRDASGTTKRQARQKAAADLYLFLRXNLSR  540
             LPGVEY L+ DTGP HMR F++R  IG H R A+ TTK+ ARQ AA  LY ++R NL R
Sbjct  194  HLPGVEYTLIGDTGPPHMREFTIRVRIGQHERTATSTTKKAARQMAAEQLYKYMRENLHR  253

Query  541  LTHDFVEEQALVRAHERAMERLVETTPAP--WKPDLGQRVSEYHYGLISHTGECTARSRR  714
            +T DF EE+ALVRAHERAM+R VE       W+PDLGQ++++Y  GL +H
Sbjct  254  VTKDFTEEEALVRAHERAMDRYVEMRDPELVWRPDLGQKIADYPIGLKTH----------  303

Query  715  RLPVVLDQCLRRAMVDTDPEKRMAARAVLSETDDIeeaaeraaaalelQTC--------W  870
                             DPEK   AR VLS  D+I+ +  +   A    T         W
Sbjct  304  ----------------IDPEKCQLARTVLSSLDEIQSSDPQVVEAALTATAAALGLTINW  347

Query  871  ETLDP----LHVLKLD-AAPALAFSGHS  939
              L      L +L+L   +PALAF+G +
Sbjct  348  SELQAERGKLQMLELTPTSPALAFAGEN  375


> ta_transcript64767_1
Length=499

 Score =   268 bits (686),  Expect = 3e-84, Method: Compositional matrix adjust.
 Identities = 158/328 (48%), Positives = 201/328 (61%), Gaps = 51/328 (16%)
 Frame = +1

Query  4    KTPITVLQEMMMKLGQIPEYECVAQSGPQHQATFEFRCKALGESVSASARSKREAKQEAA  183
            KT +TVLQE+M+K+G  PEY+CV+QSGPQH +TFE+RC+ALGE V+A+ARSK+EAKQE A
Sbjct  77   KTAVTVLQELMVKMGHTPEYDCVSQSGPQHLSTFEYRCQALGELVTATARSKKEAKQEVA  136

Query  184  RAMLLCLSTIGHRVPPPFATEFTQPSHSNQ-SAGECSEGKAPTVDSRSYVALLKELCEEY  360
            + ML  L+  GHRVPPP+ +    P  S Q S+    E +   V S SYV LLK+LCEEY
Sbjct  137  KLMLFQLARRGHRVPPPYNS---GPQLSTQQSSASKKEAEPSIVGSHSYVVLLKDLCEEY  193

Query  361  KLPGVEYALVADTGPAHMRLFSVRASIGLHSRDASGTTKRQARQKAAADLYLFLRXNLSR  540
             LPGVEY L+ DTGP HMR F++R  IG H R A+ TTK+ ARQ AA  LY ++R NL R
Sbjct  194  HLPGVEYTLIGDTGPPHMREFTIRVRIGQHERTATSTTKKAARQMAAEQLYKYMRENLHR  253

Query  541  LTHDFVEEQALVRAHERAMERLVETTPAP--WKPDLGQRVSEYHYGLISHTGECTARSRR  714
            +T DF EE+ALVRAHERAM+R VE       W+PDLGQ++++Y
Sbjct  254  VTKDFTEEEALVRAHERAMDRYVEMRDPELVWRPDLGQKIADY-----------------  296

Query  715  RLPVVLDQCLRRAMVDTDPEKRMAARAVLSETDDIeea--------aeraaaalelQTCW  870
              P+             DPEK   AR VLS  D+I+ +             AAAL L   W
Sbjct  297  --PI-------------DPEKCQLARTVLSSLDEIQSSDPQVVEAALTATAAALGLTINW  341

Query  871  ETLD----PLHVLKLD-AAPALAFSGHS  939
              L      L +L+L   +PALAF+G +
Sbjct  342  SELQAERGKLQMLELTPTSPALAFAGEN  369


> ta_transcript64769_1
Length=479

 Score =   267 bits (683),  Expect = 4e-84, Method: Compositional matrix adjust.
 Identities = 158/328 (48%), Positives = 201/328 (61%), Gaps = 51/328 (16%)
 Frame = +1

Query  4    KTPITVLQEMMMKLGQIPEYECVAQSGPQHQATFEFRCKALGESVSASARSKREAKQEAA  183
            KT +TVLQE+M+K+G  PEY+CV+QSGPQH +TFE+RC+ALGE V+A+ARSK+EAKQE A
Sbjct  77   KTAVTVLQELMVKMGHTPEYDCVSQSGPQHLSTFEYRCQALGELVTATARSKKEAKQEVA  136

Query  184  RAMLLCLSTIGHRVPPPFATEFTQPSHSNQ-SAGECSEGKAPTVDSRSYVALLKELCEEY  360
            + ML  L+  GHRVPPP+ +    P  S Q S+    E +   V S SYV LLK+LCEEY
Sbjct  137  KLMLFQLARRGHRVPPPYNS---GPQLSTQQSSASKKEAEPSIVGSHSYVVLLKDLCEEY  193

Query  361  KLPGVEYALVADTGPAHMRLFSVRASIGLHSRDASGTTKRQARQKAAADLYLFLRXNLSR  540
             LPGVEY L+ DTGP HMR F++R  IG H R A+ TTK+ ARQ AA  LY ++R NL R
Sbjct  194  HLPGVEYTLIGDTGPPHMREFTIRVRIGQHERTATSTTKKAARQMAAEQLYKYMRENLHR  253

Query  541  LTHDFVEEQALVRAHERAMERLVETTPAP--WKPDLGQRVSEYHYGLISHTGECTARSRR  714
            +T DF EE+ALVRAHERAM+R VE       W+PDLGQ++++Y
Sbjct  254  VTKDFTEEEALVRAHERAMDRYVEMRDPELVWRPDLGQKIADY-----------------  296
```

```
Query  715  RLPVVLDQCLRRAMVDTDPEKRMAARAVLSETDDIeea--------aeraaaalelQTCW  870
              P+             DPEK   AR VLS  D+I+ +            AAAL L   W
Sbjct  297  --PI-------------DPEKCQLARTVLSSLDEIQSSDPQVVEAALTATAAALGLTINW  341

Query  871  ETLD----PLHVLKLD-AAPALAFSGHS  939
              L      L +L+L   +PALAF+G +
Sbjct  342  SELQAERGKLQMLELTPTSPALAFAGEN  369


> ta_transcript64761_1
Length=534

 Score =   269 bits (687),  Expect = 4e-84, Method: Compositional matrix adjust.
 Identities = 158/328 (48%), Positives = 201/328 (61%), Gaps = 51/328 (16%)
 Frame = +1

Query  4    KTPITVLQEMMMKLGQIPEYECVAQSGPQHQATFEFRCKALGESVSASARSKREAKQEAA  183
            KT +TVLQE+M+K+G  PEY+CV+QSGPQH +TFE+RC+ALGE V+A+ARSK+EAKQE A
Sbjct  77   KTAVTVLQELMVKMGHTPEYDCVSQSGPQHLSTFEYRCQALGELVTATARSKKEAKQEVA  136

Query  184  RAMLLCLSTIGHRVPPPFATEFTQPSHSNQ-SAGECSEGKAPTVDSRSYVALLKELCEEY  360
            + ML  L+  GHRVPPP+ +    P  S Q S+    E +   V S SYV LLK+LCEEY
Sbjct  137  KLMLFQLARRGHRVPPPYNS---GPQLSTQQSSASKKEAEPSIVGSHSYVVLLKDLCEEY  193

Query  361  KLPGVEYALVADTGPAHMRLFSVRASIGLHSRDASGTTKRQARQKAAADLYLFLRXNLSR  540
             LPGVEY L+ DTGP HMR F++R  IG H R A+ TTK+ ARQ AA  LY ++R NL R
Sbjct  194  HLPGVEYTLIGDTGPPHMREFTIRVRIGQHERTATSTTKKAARQMAAEQLYKYMRENLHR  253

Query  541  LTHDFVEEQALVRAHERAMERLVETTPAP--WKPDLGQRVSEYHYGLISHTGECTARSRR  714
            +T DF EE+ALVRAHERAM+R VE       W+PDLGQ++++Y
Sbjct  254  VTKDFTEEEALVRAHERAMDRYVEMRDPELVWRPDLGQKIADY-----------------  296

Query  715  RLPVVLDQCLRRAMVDTDPEKRMAARAVLSETDDIeea--------aeraaaalelQTCW  870
              P+             DPEK   AR VLS  D+I+ +            AAAL L   W
Sbjct  297  --PI-------------DPEKCQLARTVLSSLDEIQSSDPQVVEAALTATAAALGLTINW  341

Query  871  ETLD----PLHVLKLD-AAPALAFSGHS  939
              L      L +L+L   +PALAF+G +
Sbjct  342  SELQAERGKLQMLELTPTSPALAFAGEN  369


> ta_transcript64760_1
Length=537

 Score =   269 bits (687),  Expect = 6e-84, Method: Compositional matrix adjust.
 Identities = 158/328 (48%), Positives = 201/328 (61%), Gaps = 51/328 (16%)
 Frame = +1

Query  4    KTPITVLQEMMMKLGQIPEYECVAQSGPQHQATFEFRCKALGESVSASARSKREAKQEAA  183
            KT +TVLQE+M+K+G  PEY+CV+QSGPQH +TFE+RC+ALGE V+A+ARSK+EAKQE A
Sbjct  77   KTAVTVLQELMVKMGHTPEYDCVSQSGPQHLSTFEYRCQALGELVTATARSKKEAKQEVA  136

Query  184  RAMLLCLSTIGHRVPPPFATEFTQPSHSNQ-SAGECSEGKAPTVDSRSYVALLKELCEEY  360
            + ML  L+  GHRVPPP+ +    P  S Q S+    E +   V S SYV LLK+LCEEY
Sbjct  137  KLMLFQLARRGHRVPPPYNS---GPQLSTQQSSASKKEAEPSIVGSHSYVVLLKDLCEEY  193

Query  361  KLPGVEYALVADTGPAHMRLFSVRASIGLHSRDASGTTKRQARQKAAADLYLFLRXNLSR  540
             LPGVEY L+ DTGP HMR F++R  IG H R A+ TTK+ ARQ AA  LY ++R NL R
Sbjct  194  HLPGVEYTLIGDTGPPHMREFTIRVRIGQHERTATSTTKKAARQMAAEQLYKYMRENLHR  253

Query  541  LTHDFVEEQALVRAHERAMERLVETTPAP--WKPDLGQRVSEYHYGLISHTGECTARSRR  714
            +T DF EE+ALVRAHERAM+R VE       W+PDLGQ++++Y
Sbjct  254  VTKDFTEEEALVRAHERAMDRYVEMRDPELVWRPDLGQKIADY-----------------  296

Query  715  RLPVVLDQCLRRAMVDTDPEKRMAARAVLSETDDIeea--------aeraaaalelQTCW  870
              P+             DPEK   AR VLS  D+I+ +            AAAL L   W
Sbjct  297  --PI-------------DPEKCQLARTVLSSLDEIQSSDPQVVEAALTATAAALGLTINW  341

Query  871  ETLD----PLHVLKLD-AAPALAFSGHS  939
              L      L +L+L   +PALAF+G +
Sbjct  342  SELQAERGKLQMLELTPTSPALAFAGEN  369


> ta_transcript64765_1
Length=514

 Score =   268 bits (684),  Expect = 7e-84, Method: Compositional matrix adjust.
```

```
 Identities = 158/328 (48%), Positives = 201/328 (61%), Gaps = 51/328 (16%)
 Frame = +1

Query  4    KTPITVLQEMMMKLGQIPEYECVAQSGPQHQATFEFRCKALGESVSASARSKREAKQEAA  183
            KT +TVLQE+M+K+G  PEY+CV+QSGPQH +TFE+RC+ALGE V+A+ARSK+EAKQE A
Sbjct  77   KTAVTVLQELMVKMGHTPEYDCVSQSGPQHLSTFEYRCQALGELVTATARSKKEAKQEVA  136

Query  184  RAMLLCLSTIGHRVPPPFATEFTQPSHSNQ-SAGECSEGKAPTVDSRSYVALLKELCEEY  360
            + ML  L+  GHRVPPP+ +    P  S Q S+    E +   V S SYV LLK+LCEEY
Sbjct  137  KLMLFQLARRGHRVPPPYNS---GPQLSTQQSSASKKEAEPSIVGSHSYVVLLKDLCEEY  193

Query  361  KLPGVEYALVADTGPAHMRLFSVRASIGLHSRDASGTTKRQARQKAAADLYLFLRXNLSR  540
             LPGVEY L+ DTGP HMR F++R  IG H R A+ TTK+ ARQ AA  LY ++R NL R
Sbjct  194  HLPGVEYTLIGDTGPPHMREFTIRVRIGQHERTATSTTKKAARQMAAEQLYKYMRENLHR  253

Query  541  LTHDFVEEQALVRAHERAMERLVETTPAP--WKPDLGQRVSEYHYGLISHTGECTARSRR  714
            +T DF EE+ALVRAHERAM+R VE       W+PDLGQ++++Y
Sbjct  254  VTKDFTEEEALVRAHERAMDRYVEMRDPELVWRPDLGQKIADY-----------------  296

Query  715  RLPVVLDQCLRRAMVDTDPEKRMAARAVLSETDDIeea--------aeraaaalelQTCW  870
              P+             DPEK   AR VLS  D+I+ +            AAAL L   W
Sbjct  297  --PI-------------DPEKCQLARTVLSSLDEIQSSDPQVVEAALTATAAALGLTINW  341

Query  871  ETLD----PLHVLKLD-AAPALAFSGHS  939
              L      L +L+L   +PALAF+G +
Sbjct  342  SELQAERGKLQMLELTPTSPALAFAGEN  369


> ta_transcript64753_1
Length=1742

 Score =  280 bits (716),  Expect = 2e-83, Method: Composition-based stats.
 Identities = 157/328 (48%), Positives = 201/328 (61%), Gaps = 45/328 (14%)
 Frame = +1

Query  4    KTPITVLQEMMMKLGQIPEYECVAQSGPQHQATFEFRCKALGESVSASARSKREAKQEAA  183
            KT +TVLQE+M+K+G  PEY+CV+QSGPQH +TFE+RC+ALGE V+A+ARSK+EAKQE A
Sbjct  77   KTAVTVLQELMVKMGHTPEYDCVSQSGPQHLSTFEYRCQALGELVTATARSKKEAKQEVA  136

Query  184  RAMLLCLSTIGHRVPPPFATEFTQPSHS-NQSAGECSEGKAPTVDSRSYVALLKELCEEY  360
            + ML  L+  GHRVPPP+ +    P  S  QS+    E +   V S SYV LLK+LCEEY
Sbjct  137  KLMLFQLARRGHRVPPPYNS---GPQLSTQQSSASKKEAEPSIVGSHSYVVLLKDLCEEY  193

Query  361  KLPGVEYALVADTGPAHMRLFSVRASIGLHSRDASGTTKRQARQKAAADLYLFLRXNLSR  540
             LPGVEY L+ DTGP HMR F++R  IG H R A+ TTK+ ARQ AA  LY ++R NL R
Sbjct  194  HLPGVEYTLIGDTGPPHMREFTIRVRIGQHERTATSTTKKAARQMAAEQLYKYMRENLHR  253

Query  541  LTHDFVEEQALVRAHERAMERLVETTPAP--WKPDLGQRVSEYHYGLISHTGECTARSRR  714
            +T DF EE+ALVRAHERAM+R VE       W+PDLGQ++++Y  GL +H
Sbjct  254  VTKDFTEEEALVRAHERAMDRYVEMRDPELVWRPDLGQKIADYPIGLKTH----------  303

Query  715  RLPVVLDQCLRRAMVDTDPEKRMAARAVLSETDDIeeaaeraaaalelQTC--------W  870
                            DPEK   AR VLS  D+I+ +  +   A    T         W
Sbjct  304  ----------------IDPEKCQLARTVLSSLDEIQSSDPQVVEAALTATAAALGLTINW  347

Query  871  ETLDP----LHVLKLD-AAPALAFSGHS  939
              L      L +L+L   +PALAF+G +
Sbjct  348  SELQAERGKLQMLELTPTSPALAFAGEN  375


> ta_transcript64755_1
Length=1644

 Score =  276 bits (705),  Expect = 6e-82, Method: Composition-based stats.
 Identities = 155/328 (47%), Positives = 199/328 (61%), Gaps = 51/328 (16%)
 Frame = +1

Query  4    KTPITVLQEMMMKLGQIPEYECVAQSGPQHQATFEFRCKALGESVSASARSKREAKQEAA  183
            KT +TVLQE+M+K+G  PEY+CV+QSGPQH +TFE+RC+ALGE V+A+ARSK+EAKQE A
Sbjct  77   KTAVTVLQELMVKMGHTPEYDCVSQSGPQHLSTFEYRCQALGELVTATARSKKEAKQEVA  136

Query  184  RAMLLCLSTIGHRVPPPFATEFTQPSHS-NQSAGECSEGKAPTVDSRSYVALLKELCEEY  360
            + ML  L+  GHRVPPP+ +    P  S  QS+    E +   V S SYV LLK+LCEEY
Sbjct  137  KLMLFQLARRGHRVPPPYNS---GPQLSTQQSSASKKEAEPSIVGSHSYVVLLKDLCEEY  193

Query  361  KLPGVEYALVADTGPAHMRLFSVRASIGLHSRDASGTTKRQARQKAAADLYLFLRXNLSR  540
             LPGVEY L+ DTGP HMR F++R  IG H R A+ TTK+ ARQ AA  LY ++R NL R
```

```
Sbjct  194  HLPGVEYTLIGDTGPPHMREFTIRVRIGQHERTATSTTKKAARQMAAEQLYKYMRENLHR  253

Query  541  LTHDFVEEQALVRAHERAMERLVETTPAP--WKPDLGQRVSEYHYGLISHTGECTARSRR  714
            +T DF EE+ALVRAHERAM+R VE       W+PDLGQ++++Y
Sbjct  254  VTKDFTEEEALVRAHERAMDRYVEMRDPELVWRPDLGQKIADY-----------------  296

Query  715  RLPVVLDQCLRRAMVDTDPEKRMAARAVLSETDDIeeaaeraaaalelQTC--------W  870
              P+             DPEK   AR VLS  D+I+ +  +   A    T         W
Sbjct  297  --PI-------------DPEKCQLARTVLSSLDEIQSSDPQVVEAALTATAAALGLTINW  341

Query  871  ETLDP----LHVLKLD-AAPALAFSGHS  939
              L      L +L+L   +PALAF+G +
Sbjct  342  SELQAERGKLQMLELTPTSPALAFAGEN  369


> ta_transcript64752_1
Length=1743

 Score =   276 bits (705),  Expect = 7e-82, Method: Composition-based stats.
 Identities = 155/328 (47%), Positives = 199/328 (61%), Gaps = 51/328 (16%)
 Frame = +1

Query  4    KTPITVLQEMMMKLGQIPEYECVAQSGPQHQATFEFRCKALGESVSASARSKREAKQEAA  183
            KT +TVLQE+M+K+G  PEY+CV+QSGPQH +TFE+RC+ALGE V+A+ARSK+EAKQE A
Sbjct  77   KTAVTVLQELMVKMGHTPEYDCVSQSGPQHLSTFEYRCQALGELVTATARSKKEAKQEVA  136

Query  184  RAMLLCLSTIGHRVPPPFATEFTQPSHS-NQSAGECSEGKAPTVDSRSYVALLKELCEEY  360
            + ML  L+  GHRVPPP+ +    P  S  QS+    E +   V S SYV LLK+LCEEY
Sbjct  137  KLMLFQLARRGHRVPPPYNS---GPQLSTQQSSASKKEAEPSIVGSHSYVVLLKDLCEEY  193

Query  361  KLPGVEYALVADTGPAHMRLFSVRASIGLHSRDASGTTKRQARQKAAADLYLFLRXNLSR  540
             LPGVEY L+ DTGP HMR F++R  IG H R A+ TTK+ ARQ AA  LY ++R NL R
Sbjct  194  HLPGVEYTLIGDTGPPHMREFTIRVRIGQHERTATSTTKKAARQMAAEQLYKYMRENLHR  253

Query  541  LTHDFVEEQALVRAHERAMERLVETTPAP--WKPDLGQRVSEYHYGLISHTGECTARSRR  714
            +T DF EE+ALVRAHERAM+R VE       W+PDLGQ++++Y
Sbjct  254  VTKDFTEEEALVRAHERAMDRYVEMRDPELVWRPDLGQKIADY-----------------  296

Query  715  RLPVVLDQCLRRAMVDTDPEKRMAARAVLSETDDIeeaaeraaaalelQTC--------W  870
              P+             DPEK   AR VLS  D+I+ +  +   A    T         W
Sbjct  297  --PI-------------DPEKCQLARTVLSSLDEIQSSDPQVVEAALTATAAALGLTINW  341

Query  871  ETLDP----LHVLKLD-AAPALAFSGHS  939
              L      L +L+L   +PALAF+G +
Sbjct  342  SELQAERGKLQMLELTPTSPALAFAGEN  369


> ta_transcript64750_1
Length=1756

 Score =   276 bits (705),  Expect = 7e-82, Method: Composition-based stats.
 Identities = 155/328 (47%), Positives = 199/328 (61%), Gaps = 51/328 (16%)
 Frame = +1

Query  4    KTPITVLQEMMMKLGQIPEYECVAQSGPQHQATFEFRCKALGESVSASARSKREAKQEAA  183
            KT +TVLQE+M+K+G  PEY+CV+QSGPQH +TFE+RC+ALGE V+A+ARSK+EAKQE A
Sbjct  77   KTAVTVLQELMVKMGHTPEYDCVSQSGPQHLSTFEYRCQALGELVTATARSKKEAKQEVA  136

Query  184  RAMLLCLSTIGHRVPPPFATEFTQPSHS-NQSAGECSEGKAPTVDSRSYVALLKELCEEY  360
            + ML  L+  GHRVPPP+ +    P  S  QS+    E +   V S SYV LLK+LCEEY
Sbjct  137  KLMLFQLARRGHRVPPPYNS---GPQLSTQQSSASKKEAEPSIVGSHSYVVLLKDLCEEY  193

Query  361  KLPGVEYALVADTGPAHMRLFSVRASIGLHSRDASGTTKRQARQKAAADLYLFLRXNLSR  540
             LPGVEY L+ DTGP HMR F++R  IG H R A+ TTK+ ARQ AA  LY ++R NL R
Sbjct  194  HLPGVEYTLIGDTGPPHMREFTIRVRIGQHERTATSTTKKAARQMAAEQLYKYMRENLHR  253

Query  541  LTHDFVEEQALVRAHERAMERLVETTPAP--WKPDLGQRVSEYHYGLISHTGECTARSRR  714
            +T DF EE+ALVRAHERAM+R VE       W+PDLGQ++++Y
Sbjct  254  VTKDFTEEEALVRAHERAMDRYVEMRDPELVWRPDLGQKIADY-----------------  296

Query  715  RLPVVLDQCLRRAMVDTDPEKRMAARAVLSETDDIeeaaeraaaalelQTC--------W  870
              P+             DPEK   AR VLS  D+I+ +  +   A    T         W
Sbjct  297  --PI-------------DPEKCQLARTVLSSLDEIQSSDPQVVEAALTATAAALGLTINW  341

Query  871  ETLDP----LHVLKLD-AAPALAFSGHS  939
              L      L +L+L   +PALAF+G +
Sbjct  342  SELQAERGKLQMLELTPTSPALAFAGEN  369
```

```
> ta_transcript64747_1
Length=1769

 Score =   273 bits (699),  Expect = 4e-81, Method: Compositional matrix adjust.
 Identities = 144/274 (53%), Positives = 181/274 (66%), Gaps = 30/274 (11%)
 Frame = +1

Query  4    KTPITVLQEMMMKLGQIPEYECVAQSGPQHQATFEFRCKALGESVSASARSKREAKQEAA  183
            KT +TVLQE+M+K+G  PEY+CV+QSGPQH +TFE+RC+ALGE V+A+ARSK+EAKQE A
Sbjct  77   KTAVTVLQELMVKMGHTPEYDCVSQSGPQHLSTFEYRCQALGELVTATARSKKEAKQEVA  136

Query  184  RAMLLCLSTIGHRVPPPFATEFTQPSHSNQSAGECSEGKAPTVDSRSYVALLKELCEEYK  363
            + ML  L+  GHRVPPP+ +   Q S + QS+    E +   V S SYV LLK+LCEEY
Sbjct  137  KLMLFQLARRGHRVPPPYNSG-PQLS-TQQSSASKKEAEPSIVGSHSYVVLLKDLCEEYH  194

Query  364  LPGVEYALVADTGPAHMRLFSVRASIGLHSRDASGTTKRQARQKAAADLYLFLRXNLSRL  543
            LPGVEY L+ DTGP HMR F++R  IG H R A+ TTK+ ARQ AA  LY ++R NL R+
Sbjct  195  LPGVEYTLIGDTGPPHMREFTIRVRIGQHERTATSTTKKAARQMAAEQLYKYMRENLHRV  254

Query  544  THDFVEEQALVRAHERAMERLVETTPAP--WKPDLGQRVSEYHYGLISHTGECTARSRRR  717
            T DF EE+ALVRAHERAM+R VE       W+PDLGQ++++Y  GL +H
Sbjct  255  TKDFTEEEALVRAHERAMDRYVEMRDPELVWRPDLGQKIADYPIGLKTH-----------  303

Query  718  LPVVLDQCLRRAMVDTDPEKRMAARAVLSETDDI  819
                            DPEK   AR VLS  D+I
Sbjct  304  ---------------IDPEKCQLARTVLSSLDEI  322


> ta_transcript64757_1
Length=1624

 Score =   273 bits (697),  Expect = 6e-81, Method: Composition-based stats.
 Identities = 155/328 (47%), Positives = 199/328 (61%), Gaps = 51/328 (16%)
 Frame = +1

Query  4    KTPITVLQEMMMKLGQIPEYECVAQSGPQHQATFEFRCKALGESVSASARSKREAKQEAA  183
            KT +TVLQE+M+K+G  PEY+CV+QSGPQH +TFE+RC+ALGE V+A+ARSK+EAKQE A
Sbjct  77   KTAVTVLQELMVKMGHTPEYDCVSQSGPQHLSTFEYRCQALGELVTATARSKKEAKQEVA  136

Query  184  RAMLLCLSTIGHRVPPPFATEFTQPSHS-NQSAGECSEGKAPTVDSRSYVALLKELCEEY  360
            + ML  L+  GHRVPPP+ +    P  S  QS+    E +   V S SYV LLK+LCEEY
Sbjct  137  KLMLFQLARRGHRVPPPYNS---GPQLSTQQSSASKKEAEPSIVGSHSYVVLLKDLCEEY  193

Query  361  KLPGVEYALVADTGPAHMRLFSVRASIGLHSRDASGTTKRQARQKAAADLYLFLRXNLSR  540
             LPGVEY L+ DTGP HMR F++R  IG H R A+ TTK+ ARQ AA  LY ++R NL R
Sbjct  194  HLPGVEYTLIGDTGPPHMREFTIRVRIGQHERTATSTTKKAARQMAAEQLYKYMRENLHR  253

Query  541  LTHDFVEEQALVRAHERAMERLVETTPAP--WKPDLGQRVSEYHYGLISHTGECTARSRR  714
            +T DF EE+ALVRAHERAM+R VE       W+PDLGQ++++Y
Sbjct  254  VTKDFTEEEALVRAHERAMDRYVEMRDPELVWRPDLGQKIADY-----------------  296

Query  715  RLPVVLDQCLRRAMVDTDPEKRMAARAVLSETDDIeeaaeraaaalelQTC--------W  870
              P+             DPEK   AR VLS  D+I+ +  +   A    T         W
Sbjct  297  --PI-------------DPEKCQLARTVLSSLDEIQSSDPQVVEAALTATAAALGLTINW  341

Query  871  ETLDP----LHVLKLD-AAPALAFSGHS  939
              L      L +L+L   +PALAF+G +
Sbjct  342  SELQAERGKLQMLELTPTSPALAFAGEN  369


> ta_transcript64756_1
Length=1628

 Score =   273 bits (697),  Expect = 6e-81, Method: Composition-based stats.
 Identities = 155/328 (47%), Positives = 199/328 (61%), Gaps = 51/328 (16%)
 Frame = +1

Query  4    KTPITVLQEMMMKLGQIPEYECVAQSGPQHQATFEFRCKALGESVSASARSKREAKQEAA  183
            KT +TVLQE+M+K+G  PEY+CV+QSGPQH +TFE+RC+ALGE V+A+ARSK+EAKQE A
Sbjct  77   KTAVTVLQELMVKMGHTPEYDCVSQSGPQHLSTFEYRCQALGELVTATARSKKEAKQEVA  136

Query  184  RAMLLCLSTIGHRVPPPFATEFTQPSHS-NQSAGECSEGKAPTVDSRSYVALLKELCEEY  360
            + ML  L+  GHRVPPP+ +    P  S  QS+    E +   V S SYV LLK+LCEEY
Sbjct  137  KLMLFQLARRGHRVPPPYNS---GPQLSTQQSSASKKEAEPSIVGSHSYVVLLKDLCEEY  193
```

```
Query  361  KLPGVEYALVADTGPAHMRLFSVRASIGLHSRDASGTTKRQARQKAAADLYLFLRXNLSR  540
             LPGVEY L+ DTGP HMR F++R  IG H R A+ TTK+ ARQ AA  LY ++R NL R
Sbjct  194  HLPGVEYTLIGDTGPPHMREFTIRVRIGQHERTATSTTKKAARQMAAEQLYKYMRENLHR  253

Query  541  LTHDFVEEQALVRAHERAMERLVETTPAP--WKPDLGQRVSEYHYGLISHTGECTARSRR  714
            +T DF EE+ALVRAHERAM+R VE       W+PDLGQ++++Y
Sbjct  254  VTKDFTEEEALVRAHERAMDRYVEMRDPELVWRPDLGQKIADY-----------------  296

Query  715  RLPVVLDQCLRRAMVDTDPEKRMAARAVLSETDDIeeaaeraaaalelQTC--------W  870
              P+             DPEK   AR VLS  D+I+ +  +   A    T         W
Sbjct  297  --PI-------------DPEKCQLARTVLSSLDEIQSSDPQVVEAALTATAAALGLTINW  341

Query  871  ETLDP----LHVLKLD-AAPALAFSGHS  939
              L      L +L+L   +PALAF+G +
Sbjct  342  SELQAERGKLQMLELTPTSPALAFAGEN  369


> ta_transcript64754_1
Length=1736

 Score =   273 bits (697),  Expect = 8e-81, Method: Composition-based stats.
 Identities = 155/328 (47%), Positives = 199/328 (61%), Gaps = 51/328 (16%)
 Frame = +1

Query  4    KTPITVLQEMMMKLGQIPEYECVAQSGPQHQATFEFRCKALGESVSASARSKREAKQEAA  183
            KT +TVLQE+M+K+G  PEY+CV+QSGPQH +TFE+RC+ALGE V+A+ARSK+EAKQE A
Sbjct  77   KTAVTVLQELMVKMGHTPEYDCVSQSGPQHLSTFEYRCQALGELVTATARSKKEAKQEVA  136

Query  184  RAMLLCLSTIGHRVPPPFATEFTQPSHS-NQSAGECSEGKAPTVDSRSYVALLKELCEEY  360
            + ML  L+  GHRVPPP+ +    P  S  QS+    E +   V S SYV LLK+LCEEY
Sbjct  137  KLMLFQLARRGHRVPPPYNS---GPQLSTQQSSASKKEAEPSIVGSHSYVVLLKDLCEEY  193

Query  361  KLPGVEYALVADTGPAHMRLFSVRASIGLHSRDASGTTKRQARQKAAADLYLFLRXNLSR  540
             LPGVEY L+ DTGP HMR F++R  IG H R A+ TTK+ ARQ AA  LY ++R NL R
Sbjct  194  HLPGVEYTLIGDTGPPHMREFTIRVRIGQHERTATSTTKKAARQMAAEQLYKYMRENLHR  253

Query  541  LTHDFVEEQALVRAHERAMERLVETTPAP--WKPDLGQRVSEYHYGLISHTGECTARSRR  714
            +T DF EE+ALVRAHERAM+R VE       W+PDLGQ++++Y
Sbjct  254  VTKDFTEEEALVRAHERAMDRYVEMRDPELVWRPDLGQKIADY-----------------  296

Query  715  RLPVVLDQCLRRAMVDTDPEKRMAARAVLSETDDIeeaaeraaaalelQTC--------W  870
              P+             DPEK   AR VLS  D+I+ +  +   A    T         W
Sbjct  297  --PI-------------DPEKCQLARTVLSSLDEIQSSDPQVVEAALTATAAALGLTINW  341

Query  871  ETLDP----LHVLKLD-AAPALAFSGHS  939
              L      L +L+L   +PALAF+G +
Sbjct  342  SELQAERGKLQMLELTPTSPALAFAGEN  369


> ta_transcript64748_1
Length=1763

 Score =   266 bits (681),  Expect = 1e-78, Method: Compositional matrix adjust.
 Identities = 142/274 (52%), Positives = 179/274 (65%), Gaps = 36/274 (13%)
 Frame = +1

Query  4    KTPITVLQEMMMKLGQIPEYECVAQSGPQHQATFEFRCKALGESVSASARSKREAKQEAA  183
            KT +TVLQE+M+K+G  PEY+CV+QSGPQH +TFE+RC+ALGE V+A+ARSK+EAKQE A
Sbjct  77   KTAVTVLQELMVKMGHTPEYDCVSQSGPQHLSTFEYRCQALGELVTATARSKKEAKQEVA  136

Query  184  RAMLLCLSTIGHRVPPPFATEFTQPSHSNQSAGECSEGKAPTVDSRSYVALLKELCEEYK  363
            + ML  L+  GHRVPPP+ +   Q S + QS+    E +   V S SYV LLK+LCEEY
Sbjct  137  KLMLFQLARRGHRVPPPYNSG-PQLS-TQQSSASKKEAEPSIVGSHSYVVLLKDLCEEYH  194

Query  364  LPGVEYALVADTGPAHMRLFSVRASIGLHSRDASGTTKRQARQKAAADLYLFLRXNLSRL  543
            LPGVEY L+ DTGP HMR F++R  IG H R A+ TTK+ ARQ AA  LY ++R NL R+
Sbjct  195  LPGVEYTLIGDTGPPHMREFTIRVRIGQHERTATSTTKKAARQMAAEQLYKYMRENLHRV  254

Query  544  THDFVEEQALVRAHERAMERLVETTPAP--WKPDLGQRVSEYHYGLISHTGECTARSRRR  717
            T DF EE+ALVRAHERAM+R VE       W+PDLGQ++++Y
Sbjct  255  TKDFTEEEALVRAHERAMDRYVEMRDPELVWRPDLGQKIADY------------------  296

Query  718  LPVVLDQCLRRAMVDTDPEKRMAARAVLSETDDI  819
             P+             DPEK   AR VLS  D+I
Sbjct  297  -PI-------------DPEKCQLARTVLSSLDEI  316
```

```
************************************************************************************************
*********************************************************************
Query= gi|161579134|gb|EU273921.1| Tribolium castaneum C3PO protein (C3PO)mRNA, partial cds

Length=353
                                                                      Score     E
Sequences producing significant alignments:                          (Bits)  Value

  ta_transcript67781_1                                                 32.3    0.20
  ta_transcript67782_1                                                 32.3    0.20
  ta_transcript67778_1                                                 32.0    0.27
  ta_transcript67777_1                                                 32.0    0.28
  ta_transcript14282_1                                                 29.6    1.3
  ta_transcript53466_1                                                 28.9    3.1
  ta_transcript53468_1                                                 28.9    3.8
  ta_transcript54484_1                                                 27.7    9.4
  ta_transcript54486_1                                                 27.3    9.9
  ta_transcript54485_1                                                 27.7    9.9


> ta_transcript67781_1
Length=971

 Score = 32.3 bits (72),  Expect = 0.20, Method: Composition-based stats.
 Identities = 29/86 (34%), Positives = 37/86 (43%), Gaps = 11/86 (13%)
 Frame = +2

Query  41   SFKCTVKAGGVTACGYGTSKKNAKHESAKNALVKLRREDKS--------PEIKPRAEN--  190
            +F+  V    + A G G SKK AKH +AK  L KL     +        PE      +
Sbjct  159  TFRYRVTVADLVAMGTGRSKKEAKHSAAKALLDKLTGATPADQGTNGNVPETGAVVTSFE  218

Query  191  NNLFRNFVGELNEYAASRYGAKYPSY  268
            + L  N VG L E   SR+    PSY
Sbjct  219  DKLMGNPVGWLQELCMSRFWPP-PSY  243


> ta_transcript67782_1
Length=927

 Score = 32.3 bits (72),  Expect = 0.20, Method: Composition-based stats.
 Identities = 29/86 (34%), Positives = 37/86 (43%), Gaps = 11/86 (13%)
 Frame = +2

Query  41   SFKCTVKAGGVTACGYGTSKKNAKHESAKNALVKLRREDKS--------PEIKPRAEN--  190
            +F+  V    + A G G SKK AKH +AK  L KL     +        PE      +
Sbjct  159  TFRYRVTVADLVAMGTGRSKKEAKHSAAKALLDKLTGATPADQGTNGNVPETGAVVTSFE  218

Query  191  NNLFRNFVGELNEYAASRYGAKYPSY  268
            + L  N VG L E   SR+    PSY
Sbjct  219  DKLMGNPVGWLQELCMSRFWPP-PSY  243


> ta_transcript67778_1
Length=1363

 Score = 32.0 bits (71),  Expect = 0.27, Method: Composition-based stats.
 Identities = 29/86 (34%), Positives = 37/86 (43%), Gaps = 11/86 (13%)
 Frame = +2

Query  41   SFKCTVKAGGVTACGYGTSKKNAKHESAKNALVKLRREDKS--------PEIKPRAEN--  190
            +F+  V    + A G G SKK AKH +AK  L KL     +        PE      +
Sbjct  159  TFRYRVTVADLVAMGTGRSKKEAKHSAAKALLDKLTGATPADQGTNGNVPETGAVVTSFE  218

Query  191  NNLFRNFVGELNEYAASRYGAKYPSY  268
            + L  N VG L E   SR+    PSY
Sbjct  219  DKLMGNPVGWLQELCMSRFWPP-PSY  243


> ta_transcript67777_1
Length=1407

 Score = 32.0 bits (71),  Expect = 0.28, Method: Composition-based stats.
 Identities = 29/86 (34%), Positives = 37/86 (43%), Gaps = 11/86 (13%)
 Frame = +2

Query  41   SFKCTVKAGGVTACGYGTSKKNAKHESAKNALVKLRREDKS--------PEIKPRAEN--  190
            +F+  V    + A G G SKK AKH +AK  L KL     +        PE      +
```

```
Sbjct  159  TFRYRVTVADLVAMGTGRSKKEAKHSAAKALLDKLTGATPADQGTNGNVPETGAVVTSFE  218

Query  191  NNLFRNFVGELNEYAASRYGAKYPSY  268
            + L  N VG L E   SR+    PSY
Sbjct  219  DKLMGNPVGWLQELCMSRFWPP-PSY  243


> ta_transcript14282_1
Length=138

 Score = 29.6 bits (65),  Expect = 1.3, Method: Compositional matrix adjust.
 Identities = 14/39 (36%), Positives = 18/39 (46%), Gaps = 0/39 (0%)
 Frame = +2

Query  2    ISLPVYIEEGFGTSFKCTVKAGGVTACGYGTSKKNAKHE  118
            + LP+ IE GF        +  GV  CG  T   N +HE
Sbjct  37   VFLPLPIESGFQMGVHQLPQVQGVPVCG*KTQGVNEQHE  75


> ta_transcript53466_1
Length=1278

 Score = 28.9 bits (63),  Expect = 3.1, Method: Composition-based stats.
 Identities = 18/68 (26%), Positives = 30/68 (44%), Gaps = 1/68 (1%)
 Frame = +2

Query  47   KCTVKAGGVTACGYGTSKKNAKHESAK-NALVKLRREDKSPEIKPRAENNNLFRNFVGEL  223
            KC      VT  GY  +K+  K + +K  A++K+ R +   E++      N F  F+
Sbjct  252  KCVFLVNEVTYLGYVVTKEGIKADKSKIEAILKMSRPENVSELRSFLGLVNFFAKFIKNF  311

Query  224  NEYAASRY  247
            +   A  Y
Sbjct  312  SSVLAPLY  319


> ta_transcript53468_1
Length=720

 Score = 28.9 bits (63),  Expect = 3.8, Method: Composition-based stats.
 Identities = 18/68 (26%), Positives = 30/68 (44%), Gaps = 1/68 (1%)
 Frame = +2

Query  47   KCTVKAGGVTACGYGTSKKNAKHESAK-NALVKLRREDKSPEIKPRAENNNLFRNFVGEL  223
            KC      VT  GY  +K+  K + +K  A++K+ R +   E++      N F  F+
Sbjct  252  KCVFLVNEVTYLGYVVTKEGIKADKSKIEAILKMSRPENVSELRSFLGLVNFFAKFIKNF  311

Query  224  NEYAASRY  247
            +   A  Y
Sbjct  312  SSVLAPLY  319


> ta_transcript54484_1
Length=785

 Score = 27.7 bits (60),  Expect = 9.4, Method: Composition-based stats.
 Identities = 21/63 (33%), Positives = 28/63 (44%), Gaps = 3/63 (5%)
 Frame = +2

Query  95   SKKNAKHESAKNALV--KLRREDKSPEIKPRAENNNLFRNFVGELNEYAASRY-GAKYPS  265
            SK   K     NAL+   +  + K  ++ P     NLFRNF   L  Y  S+Y G K
Sbjct  382  SKNKIKRMEGPNALLYTDMMVQ*KVVQLTPTIHPVNLFRNFPNILYHYCYSKYLGVKKGY  441

Query  266  YDF  274
            Y +
Sbjct  442  YRY  444


> ta_transcript54486_1
Length=486

 Score = 27.3 bits (59),  Expect = 9.9, Method: Composition-based stats.
 Identities = 21/63 (33%), Positives = 28/63 (44%), Gaps = 3/63 (5%)
 Frame = +2

Query  95   SKKNAKHESAKNALV--KLRREDKSPEIKPRAENNNLFRNFVGELNEYAASRY-GAKYPS  265
            SK   K     NAL+   +  + K  ++ P     NLFRNF   L  Y  S+Y G K
Sbjct  83   SKNKIKRMEGPNALLYTDMMVQ*KVVQLTPTIHPVNLFRNFPNILYHYCYSKYLGVKKGY  142
```

```
Query  266  YDF  274
            Y +
Sbjct  143  YRY  145


> ta_transcript54485_1
Length=775

 Score = 27.7 bits (60),  Expect = 9.9, Method: Composition-based stats.
 Identities = 21/63 (33%), Positives = 28/63 (44%), Gaps = 3/63 (5%)
 Frame = +2

Query  95   SKKNAKHESAKNALV--KLRREDKSPEIKPRAENNNLFRNFVGELNEYAASRY-GAKYPS  265
            SK   K     NAL+   +  + K  ++ P     NLFRNF   L  Y  S+Y G K
Sbjct  382  SKNKIKRMEGPNALLYTDMMVQ*KVVQLTPTIHPVNLFRNFPNILYHYCYSKYLGVKKGY  441

Query  266  YDF  274
            Y +
Sbjct  442  YRY  444

************************************************************************************************
*********************************************************************
Query= gi|21912829|gb|AY071926.1| Caenorhabditis elegans RNA interference promoting factor (rde-
4) mRNA, complete cds

Length=1222
                                                                      Score     E
Sequences producing significant alignments:                          (Bits)  Value

  ta_transcript67778_1                                                43.9    6e-04
  ta_transcript67777_1                                                43.9    6e-04
  ta_transcript67781_1                                                38.5    0.037
  ta_transcript67782_1                                                38.1    0.047


> ta_transcript67778_1
Length=1363

 Score = 43.9 bits (102),  Expect = 6e-04, Method: Composition-based stats.
 Identities = 58/243 (24%), Positives = 89/243 (37%), Gaps = 55/243 (23%)
 Frame = +1

Query  40   GSDVPMKPSRSEDN--KTPRN----------RTDLEMFLKKTPLMVLEE-----AAKAVY  168
            G   P  P R   N  K P N          + ++E    KTP+ VL+E          Y
Sbjct  87   GEHAPHGPRRRYQNRPKPPNNVERLPLDEAAKREMESLPMKTPVSVLQELLARRGTVPKY  146

Query  169  QKTPTWGTVELPEGFEMTLILNEITVKGQATSKKAARQKAAVEYLRKVVEKGKHEIFFIP  348
            +     G +  P  F   + + ++   G   SKK A+  AA   L K+
Sbjct  147  ELVQIEGMIHEPT-FRYRVTVADLVAMGTGRSKKEAKHSAAKALLDKKLT-----------  194

Query  349  GTTKEEALSNIDQISDKAEELKRSTSDAVQDNDND--DSIPTSAEFPPGISPTENWVGkl  522
            G T  +  +N +     A  +  S  D +  N       +  S  +PP     EN
Sbjct  195  GATPADQGTNGNVPETGA--VVTSFEDKLMGNPVGWLQELCMSRFWPPPSYHAEN-----  247

Query  523  qeksqksklqAPIYEDSKNER---TERFLVICTMCNQKTRGIRSKKKDAKNLAAWLMWKA  693
                          +D+ N R     +F ++CT+  ++  G    KK AK  AA+ MW+A
Sbjct  248  --------------DDNVNRRLPHERQFTIVCTLLKRREIGTGKSKKLAKRQAAYKMWQA  293

Query  694  LED  702
            L+D
Sbjct  294  LQD  296


> ta_transcript67777_1
Length=1407

 Score = 43.9 bits (102),  Expect = 6e-04, Method: Composition-based stats.
 Identities = 58/243 (24%), Positives = 89/243 (37%), Gaps = 55/243 (23%)
 Frame = +1

Query  40   GSDVPMKPSRSEDN--KTPRN----------RTDLEMFLKKTPLMVLEE-----AAKAVY  168
            G   P  P R   N  K P N          + ++E    KTP+ VL+E          Y
Sbjct  87   GEHAPHGPRRRYQNRPKPPNNVERLPLDEAAKREMESLPMKTPVSVLQELLARRGTVPKY  146

Query  169  QKTPTWGTVELPEGFEMTLILNEITVKGQATSKKAARQKAAVEYLRKVVEKGKHEIFFIP  348
            +     G +  P  F   + + ++   G   SKK A+  AA   L K+
```

```
Sbjct  147  ELVQIEGMIHEPT-FRYRVTVADLVAMGTGRSKKEAKHSAAKALLDKLT-----------  194

Query  349  GTTKEEALSNIDQISDKAEELKRSTSDAVQDNDND--DSIPTSAEFPPGISPTENWVGkl  522
            G T  +  +N +     A  +  S  D +  N       +  S  +PP     EN
Sbjct  195  GATPADQGTNGNVPETGA--VVTSFEDKLMGNPVGWLQELCMSRFWPPPSYHAEN-----  247

Query  523  qeksqksklqAPIYEDSKNER---TERFLVICTMCNQKTRGIRSKKKDAKNLAAWLMWKA  693
                          +D+ N R     +F ++CT+  ++   G    KK AK  AA+ MW+A
Sbjct  248  --------------DDNVNRRLPHERQFTIVCTLLKRREIGTGKSKKLAKRQAAYKMWQA  293

Query  694  LED  702
            L+D
Sbjct  294  LQD  296


> ta_transcript67781_1
Length=971

 Score = 38.5 bits (88),  Expect = 0.037, Method: Compositional matrix adjust.
 Identities = 60/244 (25%), Positives = 92/244 (38%), Gaps = 57/244 (23%)
 Frame = +1

Query  40   GSDVPMKPSRSEDN--KTPRN----------RTDLEMFLKKTPLMVLEEAAKAVYQKTPT  183
            G   P  P R   N  K P N          + ++E    KTP+ VL+E   A     P
Sbjct  87   GEHAPHGPRRRYQNRPKPPNNVERLPLDEAAKREMESLPMKTPVSVLQELL-ARRGTVPK  145

Query  184  WGTVELPEG------FEMTLILNEITVKGQATSKKAARQKAAVEYLRKVVEKGKHEIFFI  345
            +  V++ EG      F   + + ++   G   SKK A+  AA   L K+
Sbjct  146  YELVQI-EGMIHEPTFRYRVTVADLVAMGTGRSKKEAKHSAAKALLDKLT----------  194

Query  346  PGTTKEEALSNIDQISDKAEELKRSTSDAVQDNDND--DSIPTSAEFPPGISPTENWVGk  519
             G T  +  +N +     A  +  S  D +  N       +  S  +PP     EN
Sbjct  195  -GATPADQGTNGNVPETGA--VVTSFEDKLMGNPVGWLQELCMSRFWPPPSYHAEN----  247

Query  520  lqeksqksklqAPIYEDSKNERT---ERFLVICTMCNQKTRGIRSKKKDAKNLAAWLMWK  690
                           +D+ N R     +F ++CT+  ++   G    KK AK  AA+ MW+
Sbjct  248  ---------------DDNVNRRLPHERQFTIVCTLLKRREIGTGKSKKLAKRQAAYKMWQ  292

Query  691  ALED  702
            AL+D
Sbjct  293  ALQD  296


> ta_transcript67782_1
Length=927

 Score = 38.1 bits (87),  Expect = 0.047, Method: Compositional matrix adjust.
 Identities = 60/244 (25%), Positives = 92/244 (38%), Gaps = 57/244 (23%)
 Frame = +1

Query  40   GSDVPMKPSRSEDN--KTPRN----------RTDLEMFLKKTPLMVLEEAAKAVYQKTPT  183
            G   P  P R   N  K P N          + ++E    KTP+ VL+E   A     P
Sbjct  87   GEHAPHGPRRRYQNRPKPPNNVERLPLDEAAKREMESLPMKTPVSVLQELL-ARRGTVPK  145

Query  184  WGTVELPEG------FEMTLILNEITVKGQATSKKAARQKAAVEYLRKVVEKGKHEIFFI  345
            +  V++ EG      F   + + ++   G   SKK A+  AA   L K+
Sbjct  146  YELVQI-EGMIHEPTFRYRVTVADLVAMGTGRSKKEAKHSAAKALLDKLT----------  194

Query  346  PGTTKEEALSNIDQISDKAEELKRSTSDAVQDNDND--DSIPTSAEFPPGISPTENWVGk  519
             G T  +  +N +     A  +  S  D +  N       +  S  +PP     EN
Sbjct  195  -GATPADQGTNGNVPETGA--VVTSFEDKLMGNPVGWLQELCMSRFWPPPSYHAEN----  247

Query  520  lqeksqksklqAPIYEDSKNERT---ERFLVICTMCNQKTRGIRSKKKDAKNLAAWLMWK  690
                           +D+ N R     +F ++CT+  ++   G    KK AK  AA+ MW+
Sbjct  248  ---------------DDNVNRRLPHERQFTIVCTLLKRREIGTGKSKKLAKRQAAYKMWQ  292

Query  691  ALED  702
            AL+D
Sbjct  293  ALQD  296


************************************************************************************************
*********************************************************************
Query= gi|442622090|ref|NM_001276221.1| Drosophila melanogaster partner of drosha (pasha),
transcript variant C, mRNA
```

```
Length=2579
                                                                      Score     E
Sequences producing significant alignments:                          (Bits)  Value

  ta_transcript34188_1                                                89.4    2e-17
  ta_transcript34186_1                                                88.6    3e-17
  ta_transcript34187_1                                                88.6    4e-17


> ta_transcript34188_1
Length=760

 Score = 89.4 bits (220),  Expect = 2e-17, Method: Compositional matrix adjust.
 Identities = 121/348 (35%), Positives = 169/348 (49%), Gaps = 17/348 (5%)
 Frame = -1

Query  1934  QFQHLGFKFVQYGVVGLVDCRlalqhgdllllqllllegLYGVVAVEAQQGTPGLDVRMQ  1755
             QF H   + ++YGV+ L+  R  L+  DLL LQLLLL  L G  AV+AQQ  P + +R++
Sbjct  5     QFPHFVSQLLEYGVIRLIVARRVLEQSDLLFLQLLLLAALDGARAVQAQQRAPRVQLRVE  64

Query  1754  NLEDCLRSQLLALPLVFTHHFGSMFAHGHGKLVLVIRGAVDLLADLNI-AAVVALQASQQ  1578
              L   LR  LL   LV T   G + AH H +L L++   + L  ++ +  A V  QAS
Sbjct  65    RL*YSLRGALLRHLLVLTDDGGGVLAHFHRQLDLLVLQLLHLGINVLVPCAEVPEQASA*  124

Query  1577  NGVGRWLRRLVAEFGDSGIFDADVIENRQIALRFLRSAALLVLIVARYLVLDIGNEYFQR  1398
             NGV R L    AE  D+ + DA V+E  ++ +         LV  +A +L      + ++R
Sbjct  125   NGVRRGLTGRRAELRDARVLDAHVVEKAKVGVAGAAGVRALVFQLASHLR---DQDVYRR  181

Query  1397  FPSRL*LGLLFTGAIARAILEIVHRYGGGVRRGCVLELLELIGGLRFQSMLHVLVQDADE  1218
                   LG  F GA+  ++L       G V RG VL+LLELI GL  Q + HVLV+
Sbjct  182   LGCGR-LGGAFAGAVPHSVLHAADVDRGLVGRGRVLQLLELIDGLFLQRVPHVLVEHGQV  240

Query  1217  ALAIGIHDPLLPTARVR*SFASIR*YW--ELNKLSAIRQRGSLNILDVFAVFGELAASIP  1044
             A  + +H P+L        F SI  +   + ++L A R      IL   A  G L    P
Sbjct  241   AAPVHVHHPILFV-----RFPSIFLFVAGDGDELGAFR*SW---ILQGLARLGVLPPISP  292

Query  1043  TPKSQHTNHLEFEEFLAVGV*LLRCNGFLLGILVDRNNFSSRHKGSYS  900
             T +    ++LEFE+ LAV V L        L    D    F SR +  YS
Sbjct  293   TLEMHDVDNLEFEQILAVIVDLFMREMLRLPTCAD--GFHSRRRHDYS  338


 Score = 41.2 bits (95),  Expect = 0.012, Method: Compositional matrix adjust.
 Identities = 21/69 (30%), Positives = 36/69 (52%), Gaps = 0/69 (0%)
 Frame = -1

Query  740  SPPVVKTGDCSQGHCMLAGTSSAEKIGPRCATDASFAMQEERHSTVVGHLHPSLG*DFKV  561
            S  + +TGDC+    +  G + +E++G      ++ A+   RH+ V+ H HP      +
Sbjct  639  SSTISETGDCADRDVVSPGVAFSEEVGLGERAHSTGAVHIHRHAAVMPHHHPVQRQHLEC  698

Query  560  VEALFFQHS  534
            V AL FQH+
Sbjct  699  VHALLFQHN  707


> ta_transcript34186_1
Length=917

 Score = 88.6 bits (218),  Expect = 3e-17, Method: Compositional matrix adjust.
 Identities = 121/348 (35%), Positives = 169/348 (49%), Gaps = 17/348 (5%)
 Frame = -1

Query  1934  QFQHLGFKFVQYGVVGLVDCRlalqhgdllllqllllegLYGVVAVEAQQGTPGLDVRMQ  1755
             QF H   + ++YGV+ L+  R  L+  DLL LQLLLL  L G  AV+AQQ  P + +R++
Sbjct  5     QFPHFVSQLLEYGVIRLIVARRVLEQSDLLFLQLLLLAALDGARAVQAQQRAPRVQLRVE  64

Query  1754  NLEDCLRSQLLALPLVFTHHFGSMFAHGHGKLVLVIRGAVDLLADLNI-AAVVALQASQQ  1578
              L   LR  LL   LV T   G + AH H +L L++   + L  ++ +  A V  QAS
Sbjct  65    RL*YSLRGALLRHLLVLTDDGGGVLAHFHRQLDLLVLQLLHLGINVLVPCAEVPEQASA*  124

Query  1577  NGVGRWLRRLVAEFGDSGIFDADVIENRQIALRFLRSAALLVLIVARYLVLDIGNEYFQR  1398
             NGV R L    AE  D+ + DA V+E  ++ +         LV  +A +L      + ++R
Sbjct  125   NGVRRGLTGRRAELRDARVLDAHVVEKAKVGVAGAAGVRALVFQLASHLR---DQDVYRR  181

Query  1397  FPSRL*LGLLFTGAIARAILEIVHRYGGGVRRGCVLELLELIGGLRFQSMLHVLVQDADE  1218
                   LG  F GA+  ++L       G V RG VL+LLELI GL  Q + HVLV+
Sbjct  182   LGCGR-LGGAFAGAVPHSVLHAADVDRGLVGRGRVLQLLELIDGLFLQRVPHVLVEHGQV  240
```

```
Query  1217  ALAIGIHDPLLPTARVR*SFASIR*YW--ELNKLSAIRQRGSLNILDVFAVFGELAASIP  1044
             A  + +H P+L        F SI  +    + ++L A R      IL   A  G L    P
Sbjct  241   AAPVHVHHPILFV-----RFPSIFLFVAGDGDELGAFR*SW---ILQGLARLGVLPPISP  292

Query  1043  TPKSQHTNHLEFEEFLAVGV*LLRCNGFLLGILVDRNNFSSRHKGSYS  900
             T +    ++LEFE+ LAV V L       L    D    F SR +  YS
Sbjct  293   TLEMHDVDNLEFEQILAVIVDLFMREMLRLPTCAD--GFHSRRRHDYS  338


 Score = 41.2 bits (95),  Expect = 0.013, Method: Compositional matrix adjust.
 Identities = 21/69 (30%), Positives = 36/69 (52%), Gaps = 0/69 (0%)
 Frame = -1

Query  740  SPPVVKTGDCSQGHCMLAGTSSAEKIGPRCATDASFAMQEERHSTVVGHLHPSLG*DFKV  561
            S  + +TGDC+    +  G + +E++G      ++ A+    RH+ V+ H HP      +
Sbjct  639  SSTISETGDCADRDVVSPGVAFSEEVGLGERAHSTGAVHIHRHAAVMPHHHPVQRQHLEC  698

Query  560  VEALFFQHS  534
            V AL FQH+
Sbjct  699  VHALLFQHN  707


> ta_transcript34187_1
Length=901

 Score = 88.6 bits (218),  Expect = 4e-17, Method: Compositional matrix adjust.
 Identities = 121/348 (35%), Positives = 169/348 (49%), Gaps = 17/348 (5%)
 Frame = -1

Query  1934  QFQHLGFKFVQYGVVGLVDCRlalqhgdllllqllllegLYGVVAVEAQQGTPGLDVRMQ  1755
             QF H    + ++YGV+ L+  R   L+  DLL LQLLLL  L G  AV+AQQ  P + +R++
Sbjct  5     QFPHFVSQLLEYGVIRLIVARRVLEQSDLLFLQLLLLAALDGARAVQAQQRAPRVQLRVE  64

Query  1754  NLEDCLRSQLLALPLVFTHHFGSMFAHGHGKLVLVIRGAVDLLADLNI-AAVVALQASQQ  1578
              L   LR  LL   LV T   G + AH H +L L++    + L   ++ +  A V  QAS
Sbjct  65    RL*YSLRGALLRHLLVLTDDGGGVLAHFHRQLDLLVLQLLHLGINVLVPCAEVPEQASA*  124

Query  1577  NGVGRWLRRLVAEFGDSGIFDADVIENRQIALRFLRSAALLVLIVARYLVLDIGNEYFQR  1398
             NGV R L    AE  D+ + DA V+E  ++ +         LV  +A +L      + ++R
Sbjct  125   NGVRRGLTGRRAELRDARVLDAHVVEKAKVGVAGAAGVRALVFQLASHLR---DQDVYRR  181

Query  1397  FPSRL*LGLLFTGAIARAILEIVHRYGGGVRRGCVLELLELIGGLRFQSMLHVLVQDADE  1218
                   LG  F GA+  ++L       G V RG VL+LLELI GL  Q + HVLV+
Sbjct  182   LGCGR-LGGAFAGAVPHSVLHAADVDRGLVGRGRVLQLLELIDGLFLQRVPHVLVEHGQV  240

Query  1217  ALAIGIHDPLLPTARVR*SFASIR*YW--ELNKLSAIRQRGSLNILDVFAVFGELAASIP  1044
             A  + +H P+L        F SI  +    + ++L A R      IL   A  G L    P
Sbjct  241   AAPVHVHHPILFV-----RFPSIFLFVAGDGDELGAFR*SW---ILQGLARLGVLPPISP  292

Query  1043  TPKSQHTNHLEFEEFLAVGV*LLRCNGFLLGILVDRNNFSSRHKGSYS  900
             T +    ++LEFE+ LAV V L       L    D    F SR +  YS
Sbjct  293   TLEMHDVDNLEFEQILAVIVDLFMREMLRLPTCAD--GFHSRRRHDYS  338


 Score = 41.2 bits (95),  Expect = 0.012, Method: Compositional matrix adjust.
 Identities = 21/69 (30%), Positives = 36/69 (52%), Gaps = 0/69 (0%)
 Frame = -1

Query  740  SPPVVKTGDCSQGHCMLAGTSSAEKIGPRCATDASFAMQEERHSTVVGHLHPSLG*DFKV  561
            S  + +TGDC+    +  G + +E++G      ++ A+    RH+ V+ H HP      +
Sbjct  639  SSTISETGDCADRDVVSPGVAFSEEVGLGERAHSTGAVHIHRHAAVMPHHHPVQRQHLEC  698

Query  560  VEALFFQHS  534
            V AL FQH+
Sbjct  699  VHALLFQHN  707

************************************************************************************************
*********************************************************************
Query= gi|156255205|ref|NM_001102461.1| Bombyx mori argonaute 1 (Ago1), mRNA

Length=2739
                                                                      Score     E
Sequences producing significant alignments:                          (Bits)  Value

  ta_transcript56183_1                                                  284   2e-80
  ta_transcript56182_1                                                  284   3e-80
  ta_transcript51551_1                                                  173   2e-43
```

```
  ta_transcript44491_1                                          142   6e-37


> ta_transcript56183_1
Length=962

 Score =   284 bits (727),  Expect = 2e-80, Method: Compositional matrix adjust.
 Identities = 169/326 (52%), Positives = 219/326 (67%), Gaps = 0/326 (0%)
 Frame = +3

Query  231   TKPWPRGQANNAESKPFSNINAKRVCSSL*CEYST**MP*EGE*RNS*DNGALL**NIWS  410
             TK  P  QA  A+S    +INA  + +SL*C+Y+   +P*+G+*RN *+ G LL *+IW
Sbjct  118   TKSRP*RQAYYAQS*SLPDINA*GLRTSL*CQYTARQVP*KGK*RNC*NYGTLLQ*DIWC  177

Query  411   PQTSV*WKK*FVHKRPSSHRK**NGIRSNFTW*RKRQSVPCYY*MGSSGFIVCTGRGIGR  590
             PQ  V*W K*F+H R S++R+**+   S+F   RK  S+PCYY MGSSG  VCTGR + R
Sbjct  178   PQACV*W*K*FIH*RSSTNRQ**S*AGSDFAGRRKG*SIPCYYQMGSSGITVCTGRSVRR  237

Query  591   SHKTNPL*CNLSSGCSDETFTINDVYPCWTLIFLFTRGILSSPWWWTRSLVWFSSIGETK  770
             +HKT+ L*CN    C DET  +NDV+  WT++   +R +LSSP      LVWF S+ +T
Sbjct  238   AHKTDSL*CNTGFRCGDETLALNDVHTSWTVLLFVSRRLLSSPGRRKGGLVWFPSVRQT*  297

Query  771   PMENDA*Y*CVSNCFL*SSARYRVHV*SS*YTRY**PKKTINRLTES*VY*RN*RFENRN  950
             PMENDA Y*C  NC L S+A YRV+V*S  +  Y**PKK  +RL ES ++ RN*R ++RN
Sbjct  298   PMENDAEY*CFGNCILQSAASYRVYV*SIRH*GY**PKKATDRLPESEIHKRN*RSQDRN  357

Query  951   HALWYYEA*IQSVQCDT*ACSNAVFSSSTRKWTNSRMHSS*IFFGQI*DEA*IPSSAMFT  1130
             H LWY E  IQSVQCD   C+NA+   +   WTNSR++ S*IF GQ+ +E  IP+ AM T
Sbjct  358   HTLWYDEEKIQSVQCDAQTCTNAIIPPAA**WTNSRVYGS*IFPGQVQNETKIPTLAMLT  417

Query  1131  SRPGTQTHIPTS*SL*YCSGTEMYKK  1208
             S  GTQT+I    S+*+ S + +YKK
Sbjct  418   SWAGTQTYILAIGSV*HSSRSALYKK  443


 Score =   135 bits (341),  Expect = 8e-32, Method: Compositional matrix adjust.
 Identities = 173/389 (44%), Positives = 207/389 (53%), Gaps = 4/389 (1%)
 Frame = +2

Query  1148  NTHTYLLKFVIL---FRDRDV*KN*QICKHQP*SKQQHVPLQTGRGKSIIWSVEQTSILI  1318
              T TY+L    +    R     K+*QICK   *SK Q         KS  WSV +TS
Sbjct  421   GTQTYILAIGSV*HSSRSALYKKS*QICKPLQ*SKPQRGRRPIESAKSTTWSVARTSTPT  480

Query  1319  RM*KNLG*PYPIT*WRFAAECYLRRSCNMVVVCPHWVDSKHYRIREFGTCEENSFLWESR  1498
               *++ G P P T*WR AA C  R SC+     P   D K  +I+  GTC  +S  W SR
Sbjct  481   CT*RSSGSPSPTT*WRCAAACCRRPSCSTAAASPRSAD-KRCQIKACGTCGASSSSWASR  539

Query  1499  *EYGR*HVSRHKEQYERMLSRILLNNFKRYPTTLACQ**GNPVSASTLQGQTKWSLCLNT  1678
                G    SR      +M  R   ++ +R  TT AC   G+  SAST +G+T+ S C +T
Sbjct  540   SACGPSPASRRNAPSGKMRLRTSPSSCRRSRTTRACPSSGSRASASTRRGRTRSSPCSST  599

Query  1679  *slhlcsysslsscyQEKHLSMPK*KELVTQYWEWQLNVYKRRT*TKLHRRPSVIYV*KL  1858
              S    S S        +  S  +*    T+ W W+ +  + RT*T+  RR S     +
Sbjct  600   SSPPSYSCSWWWWSCPGRRRSTRR*SAWATRCWAWRRSACRPRT*TRPRRRRSPTSASRS  659

Query  1859  MLNWEALILFSFHLFVRRCstsp*ssWAWt*rtrppvttrslpsrpssAPWTRIRRGTPP  2038
               +W A    S      RCSTS * SWAWT*RTR P TTRS PSRPSSAPWTR  R TPP
Sbjct  660   T*SWAASTPSSCPRCAPRCSTSR*YSWAWT*RTRRPATTRSPPSRPSSAPWTRTPRATPP  719

Query  2039  PSGYSSTGKR*FTK*AAWSRSCSSCFTRAPADSNPTGSSCIATASPKASSCTSYSMS*LL  2218
             P   SSTG+R  T+*AAW RSC SC TRA A S+ T SSC ATAS + SS T  S S
Sbjct  720   PCACSSTGRRSCTR*AAWCRSC*SCSTRARAGSSRTASSCTATASARGSSSTCCSTSSPP  779

Query  2219  *GRLVSSWKRSTSRASLSSWYRSDITRGC  2305
               R  SSW+R+TSRAS SSW RS  TRGC
Sbjct  780   CARPASSWRRNTSRASRSSWCRSATTRGC  808


> ta_transcript56182_1
Length=977

 Score =   284 bits (726),  Expect = 3e-80, Method: Compositional matrix adjust.
 Identities = 169/326 (52%), Positives = 219/326 (67%), Gaps = 0/326 (0%)
 Frame = +3

Query  231   TKPWPRGQANNAESKPFSNINAKRVCSSL*CEYST**MP*EGE*RNS*DNGALL**NIWS  410
             TK  P  QA  A+S    +INA  + +SL*C+Y+   +P*+G+*RN *+ G LL *+IW
```

```
Sbjct  118   TKSRP*RQAYYAQS*SLPDINA*GLRTSL*CQYTARQVP*KGK*RNC*NYGTLLQ*DIWC  177

Query  411   PQTSV*WKK*FVHKRPSSHRK**NGIRSNFTW*RKRQSVPCYY*MGSSGFIVCTGRGIGR  590
             PQ  V*W K*F+H R S++R+***+   S+F   RK  S+PCYY MGSSG  VCTGR + R
Sbjct  178   PQACV*W*K*FIH*RSSTNRQ**S*AGSDFAGRRKG*SIPCYYQMGSSGITVCTGRSVRR  237

Query  591   SHKTNPL*CNLSSGCSDETFTINDVYPCWTLIFLFTRGILSSPWWWTRSLVWFSSIGETK  770
             +HKT+ L*CN    C DET  +NDV+  WT++   +R +LSSP      LVWF S+ +T
Sbjct  238   AHKTDSL*CNTGFRCGDETLALNDVHTSWTVLLFVSRRLLSSPGRRKGGLVWFPSVRQT*  297

Query  771   PMENDA*Y*CVSNCFL*SSARYRVHV*SS*YTRY**PKKTINRLTES*VY*RN*RFENRN  950
             PMENDA Y*C  NC L S+A YRV+V*S  +  Y**PKK  +RL ES ++ RN*R ++RN
Sbjct  298   PMENDAEY*CFGNCILQSAASYRVYV*SIRH*GY**PKKATDRLPESEIHKRN*RSQDRN  357

Query  951   HALWYYEA*IQSVQCDT*ACSNAVFSSSTRKWTNSRMHSS*IFFGQI*DEA*IPSSAMFT  1130
             H LWY E  IQSVQCD   C+NA+    +   WTNSR++ S*IF GQ+ +E  IP+ AM T
Sbjct  358   HTLWYDEEKIQSVQCDAQTCTNAIIPPAA**WTNSRVYGS*IFPGQVQNETKIPTLAMLT  417

Query  1131  SRPGTQTHIPTS*SL*YCSGTEMYKK  1208
             S  GTQT+I    S+*+ S + +YKK
Sbjct  418   SWAGTQTYILAIGSV*HSSRSALYKK  443


 Score =   123 bits (308),  Expect = 8e-28, Method: Compositional matrix adjust.
 Identities = 173/404 (43%), Positives = 206/404 (51%), Gaps = 19/404 (5%)
 Frame = +2

Query  1148  NTHTYLLKFVIL---FRDRDV*KN*QICKHQP*SKQQHVPLQTGRGKSIIWSVEQTSILI  1318
              T TY+L    +    R     K+*QICK   *SK Q         KS  WSV +TS
Sbjct  421   GTQTYILAIGSV*HSSRSALYKKS*QICKPLQ*SKPQRGRRPIESAKSTTWSVARTSTPT  480

Query  1319  RM*KNLG*PYPIT*WRFAAECYLRRSCNMVVVCPHWVDSKHYRIREFGTCEENSFLWESR  1498
               *++ G P P T*WR AA C  R SC+     P   D K  +I+  GTC  +S  W SR
Sbjct  481   CT*RSSGSPSPTT*WRCAAACCRRPSCSTAAASPRSAD-KRCQIKACGTCGASSSSWASR  539

Query  1499  *EYGR*HVSRHKEQYERMLSRILLNNFKRYPTTLACQ**GNPVSASTLQGQTKWSLCLNT  1678
                G    SR      +M  R   ++ +R  TT AC    G+  SAST +G+T+ S C +T
Sbjct  540   SACGPSPASRRNAPSGKMRLRTSPSSCRRSRTTRACPSSGSRASASTRRGRTRSSPCSST  599

Query  1679  *slhlcsysslsscyQEKHLSMPK*KELVTQYWEWQLNVYKRRT*TKLHRRPSVIYV*KL  1858
               S    S S         +  S  +*    T+ W W+ +  + RT*T+  RR S     +
Sbjct  600   SSPPSYSCSWWWWSCPGRRRSTRR*SAWATRCWAWRRSACRPRT*TRPRRRRSPTSASRS  659

Query  1859  MLNWEALILFSFHLFVRRCstsp*ssWAWt*rtrppvttrslpsrpssAPWTRIRRGTPP  2038
               +W A    S      RCSTS * SWAWT*RTR P TTRS PSRPSSAPWTR  R TPP
Sbjct  660   T*SWAASTPSSCPRCAPRCSTSR*YSWAWT*RTRRPATTRSPPSRPSSAPWTRTPRATPP  719

Query  2039  PSGYSSTGK---------------R*FTK*AAWSRSCSSCFTRAPADSNPTGSSCIATAS  2173
             P   SST K               R  T+*AAW RSC SC TRA A S+ T SSC ATAS
Sbjct  720   PCACSSTEKTSYPIGWIIPFKSSRRSCTR*AAWCRSC*SCSTRARAGSSRTASSCTATAS  779

Query  2174  PKASSCTSYSMS*LL*GRLVSSWKRSTSRASLSSWYRSDITRGC  2305
              + SS T  S S     R  SSW+R+TSRAS SSW RS  TRGC
Sbjct  780   ARGSSSTCCSTSSPPCARPASSWRRNTSRASRSSWCRSATTRGC  823


> ta_transcript51551_1
Length=1362

 Score =   173 bits (438),  Expect = 2e-43, Method: Compositional matrix adjust.
 Identities = 210/825 (25%), Positives = 362/825 (44%), Gaps = 110/825 (13%)
 Frame = +1

Query  241   GHEGRPIMLRANHFQI-SMPRGFVHHYDVNIQPDKCPRKVNREIVETMVHCYNKIFGALK  417
             G  G P+ + AN+F + + P+  ++ Y V+ QP++    + + ++   VH  N + G L
Sbjct  32    GSTGDPLDVCANYFTVETTPQWCLYQYHVDFQPEEDSTGLRKALLR--VHA-NTLGGYL-  87

Query  418   PVFDGRNNLYTR-----DPLPIGNDRMELEVILPGEGKDRVFRVTIKWVAQVSLFALEEA  582
               FDG   LYT      DP+ + +DR        GE       R+ IK    VS
Sbjct  88    --FDG-AILYTVKRLHPDPMELYSDRKH-----DGER----MRILIKLTCDVS-------  128

Query  583   LEGRTRQIPYDAILALDVVMRHLPSMM-YTPVGRSFFSSPEGYYHPLGGGREVWFGFHQS  759
              G       Y  I   ++++R   +++    VGR +F  P            ++W G+  +
Sbjct  129   -PG-----DYHYIQVFNIIIRKCFNLLNLQLVGRDYFD-PIAKVDIPEHKLQIWPGYKTT  181

Query  760   VRPSQWKMMLNIDVSATAFYKAQPVIEFMCEVLDIRDINDQRKPLTDSQRVKFTKEIKGL  939
             +   + ++++  ++ A    +   V++ + E    +  N ++           F +++ G
```

```
Sbjct  182   INQYEDRLLMVTEI-AHKVLRMDNVLQMLNEYAATKGSNYKKI---------FLEDVVG-  230

Query  940   KIEITHCGTMKRKYRVCNVTRRPAQMQSFPLQLENGQTVECTVAKYFLDKYKMKLRYPHL  1119
             KI +T     KR YRV +V    +   +F ++ E    ++     Y+  KY ++++ P+
Sbjct  231   KIVMTDYN--KRTYRVDDVHWETSPRSTFKMKDETVCYMD-----YYQKKYNIRIQDPNQ  283

Query  1120  PCLQVGQEHKH--------TYLPLEVCNIVPGQRCIKKLTDMQTSTMIKATARSAPD-RE  1272
             P L    + +          YL  E+C         + L + Q  + +    +  PD R
Sbjct  284   PLLVYRAKAREIRSGMPELVYLVPELCRQTGLSD--EMLANFQLMSALGRHTKIGPDLRI  341

Query  1273  REINNLVRRANFNTDSYVKE---FGLTISNNMMEVRGRVLPPPKLQYGGRVSSLGGQQAL  1443
             +++    RR    T   V+E   + L +SN+++  +GR LPP  +     V    G
Sbjct  342   QKLLQFNRRLT-QTKEVVQELASWSLKLSNDLVRFKGRQLPPEAIIQANNVKYAAGDTT-  399

Query  1444  PNQGVW--DMRGKQFFMGVEIRVWAIACFAPQRTVREDALKNFTQQLQKISNDAG--MPI  1611
               +G W  DMR K       +  W +    P+R  R DA ++F   + K  N  G  MP
Sbjct  400   --EG-WTRDMRSKPLLTIAPVGSWVV--ITPERQ-RRDA-ESFVDLIMKTGNGVGFRMP-  451

Query  1612  IGQPCFCKYA-TGPDQVEPMFKYLKSTFvqlqlvvvvlPGKTPVYAEVKRVGDTVLGMAT  1788
               +P  C  A  G      M + + +      ++ V+       Y  +K+       M T
Sbjct  452   --KPEICPIARDGHMDYANMCENVIARKNPSFILCVLRQKSADRYEAIKKKCTVDRAMPT  509

Query  1789  QCVQAKNVNKTSPQTLSN-LCLKINVKLGGIN-SILVPSLRPKVFNEPVIFLGVDVTHPP  1962
             QCV  +N+   S  +++  + ++IN KLGG   S+ VP          ++ +G DV H
Sbjct  510   QCVVGRNMTSKSAMSIATKIAIQINCKLGGAPWSVEVPL-------PTLMVIGYDVCHDT  562

Query  1963  AGDNKKPSIAAVVGSMDAHPSRYAATVRVQQHRQEIVHEMSSMVQELLIMFYKSTGGFKP  2142
                +K  S  A V ++D   + Y +TV      +E+   M   +    +  Y+      P
Sbjct  563   RAKDK--SFGAFVATLDKQMTHYYSTVNAHTSGEELSAHMGINIATA-VRKYRERNNVLP  619

Query  2143  HRIIMYRDGISEGQFLHVLQHELTAVREACIKLEAEYK---PGITFIVVQKRHHTRLFCA  2313
              RI +YRDG+ +GQ  +V  HE+  +++   +LE  Y+     + FI+V KR +TR+F
Sbjct  620   GRIFIYRDGVGDGQIPYVHSHEVEEIKK---QLETLYQGEPVKLAFIIVSKRINTRIFV-  675

Query  2314  DKKEQSGKSGNIP-AGTTVDLGITHPTEFDFYLCSHQGIQGTSRPSHYHVLWDDNHFGSD  2490
                   G+SG+ P  GT +D  IT P  +DFYL S    +GT  P+ Y+V++D      D
Sbjct  676   ----DRGRSGDNPRPGTIIDDVITLPERYDFYLVSQNVREGTIAPTSYNVIYDTTSLHPD  731

Query  2491  ELQCLTYQLCHTYVRCTRSVSIPAPAYYAHLVAFRARYHLVEKEH  2625
              +Q LTY+L H Y   + +V +P++   YAH +AF A   L  + H
Sbjct  732   RIQRLTYKLTHMYFNNSCAVRVPSVCQYAHKLAFLAANSLHNQPH  776


> ta_transcript44491_1
Length=234

 Score =   142 bits (359),  Expect = 6e-37, Method: Compositional matrix adjust.
 Identities = 89/245 (36%), Positives = 137/245 (56%), Gaps = 15/245 (6%)
 Frame = +1

Query  1849  LKINVKLGG-INSILVPSLRPKVFNEPVIFLGVDVTHPPAGDNKKPSIAAVVGSMDAHPS  2025
             L++N KLGG + SI +P      F   +I +G+D  H  A   K  S+ + + S +   +
Sbjct  2     LQMNCKLGGTLWSIAIP------FKTAMI-VGIDSYHDAA--RKSRSVCSFIASYNQSMT  52

Query  2026  RYAATVRVQQHRQEIVHEMSSMVQELLIMFYKSTGGFKPHRIIMYRDGISEGQFLHVLQH  2205
             ++ +    Q+  QE+V  + S + + L  + ++ G   P R+IMYRDG+ +GQ   + Q+
Sbjct  53    QWYSRAIFQERGQEVVDGLKSCLVDALTHYLRTNGKL-PDRVIMYRDGVGDGQLNTIKQY  111

Query  2206  ELTAVREACIKLEAEYKPGITFIVVQKRHHTRLFCADKKEQSGKSGNIPAGTTVDLGITH  2385
             E+  ++     +EA YKP +T++VVQKR +TR+F    K Q G     P GT VD  IT
Sbjct  112   EIPQMQVCFSLMEATYKPTLTYVVVQKRINTRIF---MKVQGGFENPHP-GTVVDHDITR  167

Query  2386  PTEFDFYLCSHQGIQGTSRPSHYHVLWDDNHFGSDELQCLTYQLCHTYVRCTRSVSIPAP  2565
                +DF + S +  QGT  P+HY V+ DD+   +D+ Q LTY+LCH Y     +V +PAP
Sbjct  168   RDWYDFLIASQKVNQGTVTPTHYVVVHDDSAMTADQCQRLTYKLCHLYYNWPGTVRVPAP  227

Query  2566  AYYAH  2580
               YAH
Sbjct  228   CQYAH  232
************************************************************************************************
*********************************************************************

Query= gi|166706853|ref|NM_001043530.2| Bombyx mori argonaute 2 (Ago2), mRNA

Length=4863
                                                                      Score     E
Sequences producing significant alignments:                          (Bits)  Value
```

```
  ta_transcript85453_1                                               370   6e-105
  ta_transcript85452_1                                               370   6e-105
  ta_transcript51551_1                                               143   2e-33

> ta_transcript85453_1
Length=1417

 Score =   370 bits (950),  Expect = 6e-105, Method: Compositional matrix adjust.
 Identities = 279/761 (37%), Positives = 421/761 (55%), Gaps = 6/761 (1%)
 Frame = -3

Query  3472  DEEHTALFQHAVEDPETFGILLEIVIIEYLAIGERASPqtgqvrvvgrrgv*hgahttRV  3293
              ++EH+A  QH V+ PE   ILL+++I+E L I ERA  QT Q+RVV RR V  G HT RV
Sbjct  226   NKEHSAEVQHFVQHPEPSWILLQVLIVELLPICERARAQTRQMRVVSRRRVGDGPHTARV  285

Query  3292  QMAQVVREVLHFVLRDPAVVADGVVARRTSRALDRLVRHQIEVQFTWMYYITVDHCPRID  3113
              Q+ QVV ++ H V+RD AVVA GVVA R SRAL   V +QIEV+   +  + VD    +
Sbjct  286   QVTQVVSQLFHLVVRDLAVVAHGVVASRASRALYSCVGYQIEVELLRVNDVCVDDGAGLH  345

Query  3112  VETGVVPGQ-EESRVMTFLYKNEQYFWFSVQSRDTLVGAMQSSQFTLHYLSKLTLRNPVT  2936
              V  G +  + EESRVM+FL++ E+     V S   L+ ++   QFTLHYLS+L+L N ++
Sbjct  346   VLIGDLRVRVEESRVMSFLHQ*EENLRLGVVSLHPLIRSVHRLQFTLHYLSELSLGNAIS  405

Query  2935  EHEHLLRQNPLTFLESMEMIHHYVLKLNDHLFLRCLYTQFDVVYVAFRIHRGGYGGDTRY  2756
              EHE L RQ  L      ++ H++VL+L DHLFL  L  + DVV+ AF I+R  +  +
Sbjct  406   EHEDLPRQRALLLFVHHQVFHYHVLELYDHLFLWSLNAELDVVHEAFLIYRRRHRCNAGQ  465

Query  2755  ITLIWRWMRDVSAHDHNSPTL*ALRNGTILQCLIYSHKLRVHL**NVPSCLWIHSARGGV  2576
                L+ R MR VSA  H++    A R    +  L+  H+LR+HL*  +   L +H  R
Sbjct  466   RGLLGRGMRHVSADHHHAAGRQAARQAAPV*SLVNPHQLRIHL*KYIFCRLRVHFTRCSE  525

Query  2575  FFEYMRQYTHFEFGNLFQLVIIIAAPSRNDHDQEIDLFF*SVQHTLVQVIYTDVEASHLW  2396
              F + + QYT+F    L   +II+ +  R+D D+E DL    ++  L+  ++ DV
Sbjct  526   FLDALCQYTNFPLCKLLHPMIIVVSSCRDDDDEETDLILQCIEEHLL*FLHLDVVVPLSR  585

Query  2395  LGHVHAHLSTGIH*LRHDVFIITSTWIQFYGNEAP*IQSFRFQKTVGLPYSLLWYSKLPW  2216
              L + HAHL +   *   D+  +T   I+ YG++ P +Q  RFQ+ V LP S  W+  LP
Sbjct  586   LRYPHAHLISLAF**LDDILAVTPVDIRLYGDDCPRLQGLRFQQLVCLPSSTEWFDGLPV  645

Query  2215  THF*CGRFENFRFNLIKLIGYLQAVCFEELILRVIHLLNHFLDLTFPYIRWGCSLSHHCR  2036
               H   G  + F  +  KL+  L A   E LI RVIH+   FL+ T   +R    L HHCR
Sbjct  646   AHVKVGWLQYFGLHADKLVLDLHAERLEILIARVIHVSYDFLNFTLAVVRRRRGLPHHCR  705

Query  2035  QLSVI*LLVTGLAICNFD*FHR*IDVLVFRADPQTVQVRILNPVFFLFHKIFVYGHPNRR  1856
              QL  + LLV  L+I +F *  R +D+LV R+ P TVQ+R+L   F  F ++    H
Sbjct  706   QLVFVQLLVQSLSIRDFQ*LKRVVDLLVARSHPHTVQIRVLQSDFLDFCEVVCDSHLYLL  765

Query  1855  SFVVLQRKCLT*RWVDNPVAYVLA*LSRRRSGTKYLRYDLKPSNEFIESFS-STAWLPIH  1679
              +FV+  R+     W+D  V       L+RRR+ +  + +     N+  E F+    W   +
Sbjct  766   AFVIGHREYDLVWWIDQLVTDKFPFLARRRAISTDICHYP*ILNKHFELFAHKPVW---Y  822

Query  1678  WTIGVQTKITRESINHRLILWETFVCNINVDEGFRCKYSRLE*TSPHLEPVAQVNRRCRT  1499
              WT+ V+ ++    S++H L+LWE  V +INVD    C+  RLE + PHL+ V++V+    T
Sbjct  823   WTV*VKLEVIS*SVDHPLLLWEPLVRHINVDVRTICEDRRLEQSRPHLQAVSKVDGSLWT  882

Query  1498  HEVLSACFHI*FQCSLF*HYVNTLYGVSWCIKG*STRCHMLQNIIVVYYTTSFE*HFKRH  1319
               E+L    ++ F+  L *  VN LY  SW ++G ++  +++ +   + +++ F+ +F+ H
Sbjct  883   FEILPPRLYVTFKSPLP*DDVNALYCFSWRVQGTASILYVI*DRTQIDHSSRFKGYFEIH  942

Query  1318  VFAVHIFDHHFYTISLSGDFWQRR-Q*ITIFCLIKGKDFFR  1199
                 + +   +   +      + ++R Q ITI  L+KG D FR
Sbjct  943   FPPIVVQKFNRCSKPFIACYIRKRLQRITILHLVKGDDIFR  983


> ta_transcript85452_1
Length=1460

 Score =   370 bits (951),  Expect = 6e-105, Method: Compositional matrix adjust.
 Identities = 279/761 (37%), Positives = 421/761 (55%), Gaps = 6/761 (1%)
 Frame = -3

Query  3472  DEEHTALFQHAVEDPETFGILLEIVIIEYLAIGERASPqtgqvrvvgrrgv*hgahttRV  3293
              ++EH+A  QH V+ PE   ILL+++I+E L I ERA  QT Q+RVV RR V  G HT RV
Sbjct  269   NKEHSAEVQHFVQHPEPSWILLQVLIVELLPICERARAQTRQMRVVSRRRVGDGPHTARV  328

Query  3292  QMAQVVREVLHFVLRDPAVVADGVVARRTSRALDRLVRHQIEVQFTWMYYITVDHCPRID  3113
```

```
             Q+ QVV ++ H V+RD AVVA GVVA R SRAL   V +QIEV+   +  + VD    +
Sbjct  329   QVTQVVSQLFHLVVRDLAVVAHGVVASRASRALYSCVGYQIEVELLRVNDVCVDDGAGLH  388

Query  3112  VETGVVPGQ-EESRVMTFLYKNEQYFWFSVQSRDTLVGAMQSSQFTLHYLSKLTLRNPVT  2936
             V  G +  + EESRVM+FL++ E+     V S   L+  ++  QFTLHYLS+L+L N ++
Sbjct  389   VLIGDLRVRVEESRVMSFLHQ*EENLRLGVVSLHPLIRSVHRLQFTLHYLSELSLGNAIS  448

Query  2935  EHEHLLRQNPLTFLESMEMIHHYVLKLNDHLFLRCLYTQFDVVYVAFRIHRGGYGGDTRY  2756
             EHE L RQ  L      ++ H++VL+L DHLFL  L  + DVV+ AF I+R  +  +
Sbjct  449   EHEDLPRQRALLLFVHHQVFHYHVLELYDHLFLWSLNAELDVVHEAFLIYRRRHRCNAGQ  508

Query  2755  ITLIWRWMRDVSAHDHNSPTL*ALRNGTILQCLIYSHKLRVHL**NVPSCLWIHSARGGV  2576
               L+ R MR VSA  H++    A R    +  L+  H+LR+HL*  +   L +H  R
Sbjct  509   RGLLGRGMRHVSADHHHAAGRQAARQAAPV*SLVNPHQLRIHL*KYIFCRLRVHFTRCSE  568

Query  2575  FFEYMRQYTHFEFGNLFQLVIIIAAPSRNDHDQEIDLFF*SVQHTLVQVIYTDVEASHLW  2396
             F + + QYT+F    L   +II+ +  R+D D+E DL    ++  L+  ++ DV
Sbjct  569   FLDALCQYTNFPLCKLLHPMIIVVSSCRDDDDEETDLILQCIEEHLL*FLHLDVVVPLSR  628

Query  2395  LGHVHAHLSTGIH*LRHDVFIITSTWIQFYGNEAP*IQSFRFQKTVGLPYSLLWYSKLPW  2216
             L + HAHL +   *   D+  +T   I+ YG++ P +Q  RFQ+ V LP S  W+  LP
Sbjct  629   LRYPHAHLISLAF**LDDILAVTPVDIRLYGDDCPRLQGLRFQQLVCLPSSTEWFDGLPV  688

Query  2215  THF*CGRFENFRFNLIKLIGYLQAVCFEELILRVIHLLNHFLDLTFPYIRWGCSLSHHCR  2036
              H   G  + F  +  KL+  L A   E LI RVIH+   FL+ T   +R    L HHCR
Sbjct  689   AHVKVGWLQYFGLHADKLVLDLHAERLEILIARVIHVSYDFLNFTLAVVRRRRGLPHHCR  748

Query  2035  QLSVI*LLVTGLAICNFD*FHR*IDVLVFRADPQTVQVRILNPVFFLFHKIFVYGHPNRR  1856
             QL  + LLV  L+I +F *  R +D+LV R+ P TVQ+R+L   F  F ++    H
Sbjct  749   QLVFVQLLVQSLSIRDFQ*LKRVVDLLVARSHPHTVQIRVLQSDFLDFCEVVCDSHLYLL  808

Query  1855  SFVVLQRKCLT*RWVDNPVAYVLA*LSRRRSGTKYLRYDLKPSNEFIESFS-STAWLPIH  1679
             +FV+  R+     W+D  V      L+RRR+ +  + +     N+  E F+    W   +
Sbjct  809   AFVIGHREYDLVWWIDQLVTDKFPFLARRRAISTDICHYP*ILNKHFELFAHKPVW---Y  865

Query  1678  WTIGVQTKITRESINHRLILWETFVCNINVDEGFRCKYSRLE*TSPHLEPVAQVNRRCRT  1499
             WT+ V+ ++   S++H L+LWE  V +INVD    C+  RLE + PHL+ V++V+    T
Sbjct  866   WTV*VKLEVIS*SVDHPLLLWEPLVRHINVDVRTICEDRRLEQSRPHLQAVSKVDGSLWT  925

Query  1498  HEVLSACFHI*FQCSLF*HYVNTLYGVSWCIKG*STRCHMLQNIIVVYYTTSFE*HFKRH  1319
              E+L    ++ F+  L *  VN LY  SW ++G ++  +++ +   + +++ F+ +F+ H
Sbjct  926   FEILPPRLYVTFKSPLP*DDVNALYCFSWRVQGTASILYVI*DRTQIDHSSRFKGYFEIH  985

Query  1318  VFAVHIFDHHFYTISLSGDFWQRR-Q*ITIFCLIKGKDFFR  1199
                + +   +  +      + ++R Q ITI  L+KG D FR
Sbjct  986   FPPIVVQKFNRCSKPFIACYIRKRLQRITILHLVKGDDIFR  1026


> ta_transcript51551_1
Length=1362

 Score =   143 bits (361),  Expect = 2e-33, Method: Compositional matrix adjust.
 Identities = 161/676 (24%), Positives = 298/676 (44%), Gaps = 65/676 (10%)
 Frame = +2

Query  1424  IQCIDIVLKQG-TLESYVKAGRQYFMRPASPIDLGD-GLEMWTGLFQSAI--FTSKAFIN  1591
             IQ  +I++++   L +    GR YF  P + +D+ +   L++W G +++ I  +  +  +
Sbjct  135   IQVFNIIIRKCFNLLNLQLVGRDYF-DPIAKVDIPEHKLQIWPG-YKTTINQYEDRLLMV  192

Query  1592  VDVAHKGFPKNQPMIDAFTRDFRLDPNRPVDRQPGRAAEAFNEFIRGLKVVSKILGTGPS  1771
              ++AHK                R+D    +  +      +  + I    VV KI+ T  +
Sbjct  193   TEIAHKVL--------------RMDNVLQMLNEYAATKGSNYKKIFLEDVVGKIVMTDYN  238

Query  1772  SGQLREHICNGVVDPPSRQTFTLENDKGPPVRMTVYEYFMKEKKYRIKYPDLNCLWVGPK  1951
                 R    +    P  R TF ++++      +   +Y+ K+   RI+ P+   L    K
Sbjct  239   KRTYRVDDVHWETSP--RSTFKMKDET-----VCYMDYYQKKYNIRIQDPNQPLLVYRAK  291

Query  1952  DKNIYLPM-ELVEVAYGQARNKQLNDRQL------STMVREAATPPDVRKRKIEEVIQKM  2110
              + I   M ELV +     R   L+D  L      S + R     PD+R +K+ +  +++
Sbjct  292   AREIRSGMPELVYLVPELCRQTGLSDEMLANFQLMSALGRHTKIGPDLRIQKLLQFNRRL  351

Query  2111  NYSKN--QFFKTYGLEIANEFYQVEAK------ILEAPTLEVGPRQFTVPKKGVWQANCL  2266
               +K   Q   ++ L+++N+  + + +      I++A  ++       T       ++  L
Sbjct  352   TQTKEVVQELASWSLKLSNDLVRFKGRQLPPEAIIQANNVKYAAGDTTEGWTRDMRSKPL  411

Query  2267  LKPEALNSWGFIAIELDPRGCNYEDIVSKLMNTGRQMGMNVTQPKMACFNIRINDLHKSM  2446
             L    + SW  I  E   R  + E  V  +M TG  +G  + +P++ C   R  D H
```

```
Sbjct  412   LTIAPVGSWVVITPERQRR--DAESFVDLIMKTGNGVGFRMPKPEI-CPIAR--DGHMDY  466

Query  2447  LHALE-----KQVNFLVVVVSGRGRDYYHKLKQIAELKVGILTHVFKEDTATRRMNPQTA  2611
              +  E     K  +F++ V+  +  D Y  +K+   +   + T        T +     A
Sbjct  467   ANMCENVIARKNPSFILCVLRQKSADRYEAIKKKCTVDRAMPTQCVVGRNMTSKSAMSIA  526

Query  2612  RNILLKVNSKLMGINQALENRSIPQCLKGGAVMIVGADVTHPSPDQSNIPSIAAVTASMD  2791
                I +++N KL G   ++E   +P       +M++G DV H +  ++   S  A  A++D
Sbjct  527   TKIAIQINCKLGGAPWSVE---VPL----PTLMVIGYDVCHDT--RAKDKSFGAFVATLD  577

Query  2792  TKCYIYNIELSIQTPKKEMIVQFEDIMVDHFHAFKKSQGILPKKVFVFRDGVSEGQFAEV  2971
               +   Y   ++  T  +E+       +      +++   +LP ++F++RDGV +GQ   V
Sbjct  578   KQMTHYYSTVNAHTSGEELSAHMGINIATAVRKYRERNNVLPGRIFIYRDGVGDGQIPYV  637

Query  2972  MKSELTGLHRAYQRVAGLNAKP-EVLFILVQKRHHTRFFLPGNNARFNVDPGTVVDRDIV  3148
                 E+  + +  + +     +P ++ FI+V KR +TR F+    +  N  PGT++D  I
Sbjct  638   HSHEVEEIKKQLETL--YQGEPVKLAFIIVSKRINTRIFVDRGRSGDNPRPGTIIDDVIT  695

Query  3149  HPRELDFYLVSHQAIKGTARPTRYHAVCNDGRIPENEVEHLAYYLCHLYARCMRAVSYPA  3328
               P   DFYLVS    +GT  PT Y+ + +   +  + ++ L Y L H+Y     AV  P+
Sbjct  696   LPERYDFYLVSQNVREGTIAPTSYNVIYDTTSLHPDRIQRLTYKLTHMYFNNSCAVRVPS  755

Query  3329  PTYYAH-LACLRARSL  3373
                 YAH LA L A SL
Sbjct  756   VCQYAHKLAFLAANSL  771

************************************************************************************************
*********************************************************************


Query= gi|166706857|ref|NM_001104597.2| Bombyx mori argonaute 3 (Ago3),mRNA

Length=2781
                                                                      Score     E
Sequences producing significant alignments:                          (Bits)  Value

  ta_transcript51551_1                                                  498   1e-155
  ta_transcript44491_1                                                  425   4e-141

> ta_transcript51551_1
Length=1362

 Score =   498 bits (1282),  Expect = 1e-155, Method: Compositional matrix adjust.
 Identities = 270/767 (35%), Positives = 421/767 (55%), Gaps = 16/767 (2%)
 Frame = +1

Query  502   KKGETGVPIEVTCNYIYLNFKEN-IVFEYEVKFEPDQDYKHLRFKLLNEHIEHFKEKTFD  678
             KKG TG P++V  NY  +       +++Y V F+P++D   LR  LL  H        FD
Sbjct  30    KKGSTGDPLDVCANYFTVETTPQWCLYQYHVDFQPEEDSTGLRKALLRVHANTLGGYLFD  89

Query  679   GTTLYVPHELPDAVRNLVSTNPYDQSKVNVSIIFRRTRRLS----EMIHIYNVMFKCIMK  846
             G  LY    L      L S   +D  ++   I+ + T  +S      I ++N++    +
Sbjct  90    GAILYTVKRLHPDPMELYSDRKHDGERMR--ILIKLTCDVSPGDYHYIQVFNIIIRKCFN  147

Query  847   DLKLIRFGRQHYNEHAAIQIPQHKLEVWPGYVTAVDEYEGGLMLTLDSTHRVLRTQTVLS  1026
              L L   GR +++  A + IP+HKL++WPGY T +++YE  L++  +  H+VLR   VL
Sbjct  148   LLNLQLVGRDYFDPIAKVDIPEHKLQIWPGYKTTINQYEDRLLMVTEIAHKVLRMDNVLQ  207

Query  1027  LIKEVVQTEGANWKRKMTDILIGASVMTTYNKKLFRVDTIDDKMSPRSTFEKTEKGETVQ  1206
             ++ E   T+G+N+K+   + ++G   VMT YNK+ +RVD +  + SPRSTF+   K ETV
Sbjct  208   MLNEYAATKGSNYKKIFLEDVVGKIVMTDYNKRTYRVDDVHWETSPRSTFKM--KDETV-  264

Query  1207  ISFIDYYKKNYGIEIMDWDQPLLISRDTKRMPGSDTPTDFMICLIPELCQLTGLTDDQRS  1386
               ++DYY+K Y I I D +QPLL+ R   R   S  P   ++ L+PELC+ TGL+D+  +
Sbjct  265   -CYMDYYQKKYNIRIQDPNQPLLVYRAKAREIRSGMPE--LVYLVPELCRQTGLSDEMLA  321

Query  1387  NFRLMKDVATYTRITPNQRHAAFKKYIESVMKNETAKSRLAGWGLSIAPETVNLTARTLP  1566
             NF+LM  +  +T+I P+ R     ++   + + +     LA W L ++ + V    R LP
Sbjct  322   NFQLMSALGRHTKIGPDLRIQKLLQFNRRLTQTKEVVQELASWSLKLSNDLVRFKGRQLP  381

Query  1567  PETLYFGDNVRVPGKPNAE-WNSEVTKHSVMQAVDIMRWVLLFTQRDKQVAMDFLSTLKR  1743
             PE +   +NV+       E W  ++    ++     +  WV++  +R ++ A  F+  + +
Sbjct  382   PEAIIQANNVKYAAGDTTEGWTRDMRSKPLLTIAPVGSWVVITPERQRRDAESFVDLIMK  441

Query  1744  NCRPMGIMVSDAELVPLANDRTDTYVLALKKCIT-SSVQLVVAICSTKRDDRYAAIKKVC  1920
                 +G   +   E+ P+A D    Y    +  I   +   ++ +   K  DRY AIKK C
Sbjct  442   TGNGVGFRMPKPEICPIARDGHMDYANMCENVIARKNPSFILCVLRQKSADRYEAIKKKC  501
```

```
Query  1921  CADNPVPSQVINARTLMNTNKIRSITQKILLQLNCKLGGTLWSISIPFKSAMIVGIDSYH  2100
               D  +P+Q +  R  M +    SI  KI +Q+NCKLGG  WS+ +P  + M++G D  H
Sbjct  502   TVDRAMPTQCVVGRN-MTSKSAMSIATKIAIQINCKLGGAPWSVEVPLPTLMVIGYDVCH  560

Query  2101  DPSRRNRSVCSFVASYNQSMTLWYSKVIFQEKGQEIVDGLKCCLVDALTHYLRSNGQLPD  2280
             D   +++S  +FVA+ ++ MT +YS V     G+E+   +   +  A+  Y   N  LP
Sbjct  561   DTRAKDKSFGAFVATLDKQMTHYYSTVNAHTSGEELSAHMGINIATAVRKYRERNNVLPG  620

Query  2281  RIIIYRDGVGDGQLKLLQQYEIPQMKICFTILGSNYQPTLTYVVVQKRINTRIFLKSRDG  2460
             RI IYRDGVGDGQ+  +  +E+ ++K     L       L +++V KRINTRIF+
Sbjct  621   RIFIYRDGVGDGQIPYVHSHEVEEIKKQLETLYQGEPVKLAFIIVSKRINTRIFVDRGRS  680

Query  2461  YDNPNPGTVVDHCITRRDWYDFLIVSQKVTQGTVTPTHYVVVYDDSGITPDQCQRLTYKM  2640
              DNP PGT++D  IT   + YDF +VSQ V +GT+ PT Y V+YD + + PD+ QRLTYK+
Sbjct  681   GDNPRPGTIIDDVITLPERYDFYLVSQNVREGTIAPTSYNVIYDTTSLHPDRIQRLTYKL  740

Query  2641  CHLYYNWPGTVRVPAPCQYAHKLSYLVGQCVHAQPSDVLVDKLFFL*  2781
              H+Y+N    VRVP+ CQYAHKL++L    +H QP   L + L+FL*
Sbjct  741   THMYFNNSCAVRVPSVCQYAHKLAFLAANSLHNQPHYTLNETLYFL*  787


> ta_transcript44491_1
Length=234

 Score =   425 bits (1092),  Expect = 4e-141, Method: Compositional matrix adjust.
 Identities = 188/234 (80%), Positives = 219/234 (94%), Gaps = 0/234 (0%)
 Frame = +1

Query  2008  LLQLNCKLGGTLWSISIPFKSAMIVGIDSYHDPSRRNRSVCSFVASYNQSMTLWYSKVIF  2187
             LLQ+NCKLGGTLWSI+IPFK+AMIVGIDSYHD +R++RSVCSF+ASYNQSMT WYS+ IF
Sbjct  1     LLQMNCKLGGTLWSIAIPFKTAMIVGIDSYHDAARKSRSVCSFIASYNQSMTQWYSRAIF  60

Query  2188  QEKGQEIVDGLKCCLVDALTHYLRSNGQLPDRIIIYRDGVGDGQLKLLQQYEIPQMKICF  2367
             QE+GQE+VDGLK CLVDALTHYLR+NG+LPDR+I+YRDGVGDGQL  ++QYEIPQM++CF
Sbjct  61    QERGQEVVDGLKSCLVDALTHYLRTNGKLPDRVIMYRDGVGDGQLNTIKQYEIPQMQVCF  120

Query  2368  TILGSNYQPTLTYVVVQKRINTRIFLKSRDGYDNPNPGTVVDHCITRRDWYDFLIVSQKV  2547
             +++ + Y+PTLTYVVVQKRINTRIF+K + G++NP+PGTVVDH ITRRDWYDFLI SQKV
Sbjct  121   SLMEATYKPTLTYVVVQKRINTRIFMKVQGGFENPHPGTVVDHDITRRDWYDFLIASQKV  180

Query  2548  TQGTVTPTHYVVVYDDSGITPDQCQRLTYKMCHLYYNWPGTVRVPAPCQYAHKL  2709
              QGTVTPTHYVVV+DDS +T DQCQRLTYK+CHLYYNWPGTVRVPAPCQYAHKL
Sbjct  181   NQGTVTPTHYVVVHDDSAMTADQCQRLTYKLCHLYYNWPGTVRVPAPCQYAHKL  234

***********************************************************************************************
*********************************************************************
Query= gi|577028025|gb|KF579958.1| Caenorhabditis japonica strain VX0158
ALG-1 (alg-1) gene, partial cds

Length=624


***** No hits found *****
***********************************************************************************************
*********************************************************************
Query= gi|114051845|ref|NM_001046722.1| Bombyx mori apoptosis-linked protein 2 (Alg-2), mRNA

Length=631
                                                                      Score     E
Sequences producing significant alignments:                          (Bits)  Value

  ta_transcript79570_1                                                  171   2e-47
  ta_transcript79596_1                                                  123   5e-32
  ta_transcript79595_1                                                  122   2e-31
  ta_transcript79592_1                                                  122   2e-31
  ta_transcript79591_1                                                  122   2e-31


> ta_transcript79570_1
Length=1270

 Score =   171 bits (432),  Expect = 2e-47, Method: Compositional matrix adjust.
 Identities = 95/176 (54%), Positives = 129/176 (73%), Gaps = 0/176 (0%)
 Frame = -2
```

```
Query  585   LYL*SKTHFKELLIMYRDDTILVRVVLAEC*CKSIKCDAALYEIIKQYCPSTESIKFLHH  406
              YL SK HF++LLI+Y D TIL+RVVLA+ *C  IKCD  L +IIK  C +++SI+FL+H
Sbjct  1028  FYLESKDHFQKLLIVYGDHTILIRVVLAKG*C*HIKCDTTLNKIIK*DCATSKSIEFLNH  1087

Query  405   YTNNIIRQSVSKGCKCILQFHSIYIPRIITVK*SETILPIRNIFPKCTKVLK*YDTPILF  226
             + +N IRQSVS+  K  L F  I IPR+I V+  ET+LPIR+IFP+ ++V+K*Y+T  L
Sbjct  1088  HLHNFIRQSVSECRKGTL*FTLINIPRVIPVEGPETVLPIRDIFPQRSEVIK*YNTSALL  1147

Query  225   VKHTNHQTYSFRIKWIPCSIGQSLLQFISRYVTTSIFVNTSKNVP*EISAWHWRLE  58
             VKH  H++  F ++ IPCSI +SLL  ISR V TS+ VN+S+ +P ++SAWH  LE
Sbjct  1148  VKHAYHESNCFWVERIPCSIRKSLL*LISRNVATSVLVNSSEYIPKKLSAWHG*LE  1203


> ta_transcript79596_1
Length=470

 Score =   123 bits (309),  Expect = 5e-32, Method: Compositional matrix adjust.
 Identities = 86/176 (49%), Positives = 99/176 (56%), Gaps = 0/176 (0%)
 Frame = -1

Query  586  FIPLIQNPF*GIAHNVS**HHLGPSRTGGMLM*EYKV*RSTV*NHQTILSLYRIYQISAP  407
             +P +Q PF  IAH+V * HHL P RTG  LM  YKV* +T *NHQ  L   +I +IS P
Sbjct  228  LLP*VQRPFSEIAHSVW*SHHLDPCRTGERLMLTYKV*HNTE*NHQIGLCHVQIDRISEP  287

Query  406  LYQQHHQTICIQRL*VHSSIPLYLYSQNYHGQMIGNNFANQKHISKVHQSPQMI*HPYFV  227
               Q H TICI+  * HS +  Y YSQ+Y  +  GN+FAN HIS   +S QMI*H
Sbjct  288  SSAQFHPTICIRMP*GHSLVHSYQYSQSYPCRRTGNSFANP*HISTKIRSHQMI*HLCLA  347

Query  226  CQTYQSSDVQFQD*MDSMFHWTEPVAVHQQICNHFYLCQHF*KCSIRNLCLALAIG  59
            CQT  S     F  * DSMFH  E    HQQ C+HF  CQ F       LCLA   G
Sbjct  348  CQTCLS*VELFLG*KDSMFH*KELAVTHQQKCSHFCPCQLFGIYPKETLCLAWVTG  403


> ta_transcript79595_1
Length=500

 Score =   122 bits (305),  Expect = 2e-31, Method: Compositional matrix adjust.
 Identities = 85/172 (49%), Positives = 97/172 (56%), Gaps = 0/172 (0%)
 Frame = -1

Query  574  IQNPF*GIAHNVS**HHLGPSRTGGMLM*EYKV*RSTV*NHQTILSLYRIYQISAPLYQQ  395
            +Q PF  IAH+V * HHL P RTG  LM  YKV* +T *NHQ  L   +I +IS P   Q
Sbjct  262  VQRPFSEIAHSVW*SHHLDPCRTGERLMLTYKV*HNTE*NHQIGLCHVQIDRISEPSSAQ  321

Query  394  HHQTICIQRL*VHSSIPLYLYSQNYHGQMIGNNFANQKHISKVHQSPQMI*HPYFVCQTY  215
             H TICI+  * HS +  Y YSQ+Y  +  GN+FAN HIS   +S QMI*H    CQT
Sbjct  322  FHPTICIRMP*GHSLVHSYQYSQSYPCRRTGNSFANP*HISTKIRSHQMI*HLCLACQTC  381

Query  214  QSSDVQFQD*MDSMFHWTEPVAVHQQICNHFYLCQHF*KCSIRNLCLALAIG  59
             S     F  * DSMFH  E    HQQ C+HF  CQ F       LCLA   G
Sbjct  382  LS*VELFLG*KDSMFH*KELAVTHQQKCSHFCPCQLFGIYPKETLCLAWVTG  433


> ta_transcript79592_1
Length=554

 Score =   122 bits (306),  Expect = 2e-31, Method: Compositional matrix adjust.
 Identities = 85/172 (49%), Positives = 97/172 (56%), Gaps = 0/172 (0%)
 Frame = -1

Query  574  IQNPF*GIAHNVS**HHLGPSRTGGMLM*EYKV*RSTV*NHQTILSLYRIYQISAPLYQQ  395
            +Q PF  IAH+V * HHL P RTG  LM  YKV* +T *NHQ  L   +I +IS P   Q
Sbjct  316  VQRPFSEIAHSVW*SHHLDPCRTGERLMLTYKV*HNTE*NHQIGLCHVQIDRISEPSSAQ  375

Query  394  HHQTICIQRL*VHSSIPLYLYSQNYHGQMIGNNFANQKHISKVHQSPQMI*HPYFVCQTY  215
             H TICI+  * HS +  Y YSQ+Y  +  GN+FAN HIS   +S QMI*H    CQT
Sbjct  376  FHPTICIRMP*GHSLVHSYQYSQSYPCRRTGNSFANP*HISTKIRSHQMI*HLCLACQTC  435

Query  214  QSSDVQFQD*MDSMFHWTEPVAVHQQICNHFYLCQHF*KCSIRNLCLALAIG  59
             S     F  * DSMFH  E    HQQ C+HF  CQ F       LCLA   G
Sbjct  436  LS*VELFLG*KDSMFH*KELAVTHQQKCSHFCPCQLFGIYPKETLCLAWVTG  487


> ta_transcript79591_1
Length=555
```

```
 Score =   122 bits (306),  Expect = 2e-31, Method: Compositional matrix adjust.
 Identities = 85/172 (49%), Positives = 97/172 (56%), Gaps = 0/172 (0%)
 Frame = -1

Query  574  IQNPF*GIAHNVS**HHLGPSRTGGMLM*EYKV*RSTV*NHQTILSLYRIYQISAPLYQQ  395
            +Q PF  IAH+V * HHL P RTG  LM  YKV* +T *NHQ  L   +I +IS P   Q
Sbjct  317  VQRPFSEIAHSVW*SHHLDPCRTGERLMLTYKV*HNTE*NHQIGLCHVQIDRISEPSSAQ  376

Query  394  HHQTICIQRL*VHSSIPLYLYSQNYHGQMIGNNFANQKHISKVHQSPQMI*HPYFVCQTY  215
             H TICI+  * HS +  Y YSQ+Y  +  GN+FAN  HIS   +S QMI*H    CQT
Sbjct  377  FHPTICIRMP*GHSLVHSYQYSQSYPCRRTGNSFANP*HISTKIRSHQMI*HLCLACQTC  436

Query  214  QSSDVQFQD*MDSMFHWTEPVAVHQQICNHFYLCQHF*KCSIRNLCLALAIG  59
             S    F  * DSMFH  E    HQQ C+HF  CQ F       LCLA   G
Sbjct  437  LS*VELFLG*KDSMFH*KELAVTHQQKCSHFCPCQLFGIYPKETLCLAWVTG  488


*************************************************************************************************
*********************************************************************
Query= gi|392920128|ref|NM_171525.4| Caenorhabditis elegans Protein RDE-1 (rde-1) mRNA, complete
cds

Length=3063
                                                                      Score     E
Sequences producing significant alignments:                          (Bits)  Value

  ta_transcript44491_1                                                94.7    2e-20
  ta_transcript51551_1                                                94.7    7e-19


> ta_transcript44491_1
Length=234

 Score = 94.7 bits (234),  Expect = 2e-20, Method: Compositional matrix adjust.
 Identities = 79/300 (26%), Positives = 123/300 (41%), Gaps = 71/300 (24%)
 Frame = +1

Query  2029  LKINAKLGGINQELDWSEIAEISPEEKERRKTMPL--TMYVGIDVTHPTSYSGIDYSIAA  2202
             L++N KLGG      WS IA            +P    M VGID  H  +      S+ +
Sbjct  2     LQMNCKLGGTL----WS-IA------------IPFKTAMIVGIDSYHDAARKS--RSVCS  42

Query  2203  VVASINPGGTIYRNMIVTQEECRPGERAVAHGRERTDILEAKFVKLLREFAENNDNRAPA  2382
              +AS N   T + +  + QE           G+E  D L++  V  L  +   N  + P
Sbjct  43    FIASYNQSMTQWYSRAIFQER----------GQEVVDGLKSCLVDALTHYLRTN-GKLPD  91

Query  2383  HIVVYRDGVSDSEMLRVSHDELRSLKSEVKQFMSERDGEDPEPKYTFIVIQKRHNTRLLR  2562
              +++YRDGV D ++  +   E+  ++       +       +P  T++V+QKR NTR+
Sbjct  92    RVIMYRDGVGDGQLNTIKQYEIPQMQVCFSLMEATY-----KPTLTYVVVQKRINTRIFM  146

Query  2563  RMEKDKPVVNKDLTPAETDVAVAAVKQWEEDMKESKETGIVNPSSGTTVDKLIVSKYKFD  2742
             +++                                   G  NP  GT VD  I  +  +D
Sbjct  147   KVQG----------------------------------GFENPHPGTVVDHDITRRDWYD  172

Query  2743  FFLASHHGVLGTSRPGHYTVMYDDKGMSQDEVYKMTYGLAFLSARCRKPISLPVPVHYAH  2922
             F +AS     GT  P HY V++DD  M+ D+  ++TY L  L       + +P P  YAH
Sbjct  173   FLIASQKVNQGTVTPTHYVVVHDDSAMTADQCQRLTYKLCHLYYNWPGTVRVPAPCQYAH  232


> ta_transcript51551_1
Length=1362

 Score = 94.7 bits (234),  Expect = 7e-19, Method: Compositional matrix adjust.
 Identities = 93/369 (25%), Positives = 146/369 (40%), Gaps = 75/369 (20%)
 Frame = +1

Query  1816  NMFERLPDKEQKVLMFIIISKRQLNAYGFVKHYCDHTIGVANQHITSETVTKALASLRHE  1995
             NM E +  ++    +  ++ ++  + Y  +K  C     +  Q +    +T
Sbjct  468   NMCENVIARKNPSFILCVLRQKSADRYEAIKKKCTVDRAMPTQCVVGRNMT--------S  519

Query  1996  KGSKRIFYQIALKINAKLGGINQELDWSEIAEISPEEKERRKTMPLTMYVGIDVTHPTSY  2175
             K +  I  +IA++IN KLGG      WS   E+          +P  M +G DV H T
Sbjct  520   KSAMSIATKIAIQINCKLGGA----PWS--VEVP---------LPTLMVIGYDVCHDTR-  563

Query  2176  SGIDYSIAAVVASINPGGTIYRNMIVTQEECRPGERAVAHGRERTDILEAKFVKLLREFA  2355
                D S  A VA+++   T Y + +        GE   AH       +       +R++
Sbjct  564   -AKDKSFGAFVATLDKQMTHYYSTVNAHTS---GEELSAH-------MGINIATAVRKYR  612
```

```
Query  2356  ENNDNRAPAHIVVYRDGVSDSEMLRVSHDELRSLKSEVKQFMSERDGEDPEPKYTFIVIQ  2535
             E N N  P  I +YRDGV D ++  V   E+  +K   KQ  +   GE    K  FI++
Sbjct  613   ERN-NVLPGRIFIYRDGVGDGQIPYVHSHEVEEIK---KQLETLYQGE--PVKLAFIIVS  666

Query  2536  KRHNTRLLRRMEKDKPVVNKDLTPAETDVAVAAVKQWEEDMKESKETGIVNPSSGTTVDK  2715
             KR NTR+                                D   S +    NP  GT +D
Sbjct  667   KRINTRIF------------------------------VDRGRSGD----NPRPGTIIDD  692

Query  2716  LIVSKYKFDFFLASHHGVLGTSRPGHYTVMYDDKGMSQDEVYKMTYGLAFLSARCRKPIS  2895
             +I    ++DF+L S +   GT  P  Y V+YD   +  D + ++TY L  +       +
Sbjct  693   VITLPERYDFYLVSQNVREGTIAPTSYNVIYDTTSLHPDRIQRLTYKLTHMYFNNSCAVR  752

Query  2896  LPVPVHYAH  2922
             +P    YAH
Sbjct  753   VPSVCQYAH  761

************************************************************************************************
*******************************************************************
Query= gi|392918268|ref|NM_070961.7| Caenorhabditis elegans Protein ERGO-1 (ergo-1) mRNA,
complete cds

Length=3366
                                                                      Score     E
Sequences producing significant alignments:                          (Bits)  Value

  ta_transcript44491_1                                                  103    3e-23
  ta_transcript51551_1                                                 94.4    1e-18


> ta_transcript44491_1
Length=234

 Score =   103 bits (257),  Expect = 3e-23, Method: Compositional matrix adjust.
 Identities = 67/243 (28%), Positives = 121/243 (50%), Gaps = 31/243 (13%)
 Frame = +1

Query  2506  WTIFTDPAKPTLVLGIDVSHPSTRDRETGNVLQKMSAATVVGNIDLDVTEFRASSRIQDT  2685
             W+I   P K  +++GID  H + R        +  S  + + + +  +T++ + +  Q+
Sbjct  13    WSIAI-PFKTAMIVGIDSYHDAAR--------KSRSVCSFIASYNQSMTQWYSRAIFQER  63

Query  2686  GVECLIDFSKEIDERIGEFIDHTGKRPAHIVVYRDGLSEGDF----QKYLFEERVCIEER  2853
             G E +      + + +  ++   GK P  +++YRDG+ +G      Q  + + +VC
Sbjct  64    GQEVVDGLKSCLVDALTHYLRTNGKLPDRVIMYRDGVGDGQLNTIKQYEIPQMQVCFS--  121

Query  2854  CLKIDTSFQPSITYIVVTKRHHTQFFLEDPSQGYESQGYNVLPGTLIEDAVTTNKYYDFF  3033
                ++ +++P++TY+VV KR +T+ F++    G+E    N  PGT+++  +T   +YDF
Sbjct  122   --LMEATYKPTLTYVVVQKRINTRIFMK-VQGGFE----NPHPGTVVDHDITRRDWYDFL  174

Query  3034  LSTQIGNEGCFRPTHYYVLHDTWTGKPDSFWPTVTHALTYNFCR----STTTVALPAPVL  3201
             +++Q  N+G   PTHY V+HD       D         LTY  C        TV +PAP
Sbjct  175   IASQKVNQGTVTPTHYVVVHDDSAMTADQ-----CQRLTYKLCHLYYNWPGTVRVPAPCQ  229

Query  3202  YAH  3210
             YAH
Sbjct  230   YAH  232


> ta_transcript51551_1
Length=1362

 Score = 94.4 bits (233),  Expect = 1e-18, Method: Compositional matrix adjust.
 Identities = 81/295 (27%), Positives = 137/295 (46%), Gaps = 26/295 (9%)
 Frame = +1

Query  2374  LEKSLGVVGPSPKNCALTRLMVEKVLGKVGT---THRKLERGGAHKTWTIFTDPAKPTLV  2544
             ++K   V    P  C + R M  K    + T        + GGA   W++   P    +V
Sbjct  497   IKKKCTVDRAMPTQCVVGRNMTSKSAMSIATKIAIQINCKLGGA--PWSVEV-PLPTLMV  553

Query  2545  LGIDVSHPSTRDRETGNVLQKMSAATVVGNIDLDVTEFRASSRIQDTGVECLIDFSKEID  2724
             +G DV H  TR ++        S    V  +D  +T + ++     +G E        I
Sbjct  554   IGYDVCH-DTRAKDK-------SFGAFVATLDKQMTHYYSTVNAHTSGEELSAHMGINIA  605

Query  2725  ERIGEFIDHTGKRPAHIVVYRDGLSEGDFQKYLFEERVCIEERCLKIDTSFQ---PSITY  2895
               + ++ +     P  I +YRDG+ +G    Y+    V  EE   +++T +Q     + +
Sbjct  606   TAVRKYRERNNVLPGRIFIYRDGVGDGQI-PYVHSHEV--EEIKKQLETLYQGEPVKLAF  662
```

```
Query  2896  IVVTKRHHTQFFLEDPSQGYESQGYNVLPGTLIEDAVTTNKYYDFFLSTQIGNEGCFRPT  3075
             I+V+KR +T+ F++   +G    G N  PGT+I+D +T  + YDF+L +Q   EG   PT
Sbjct  663  IIVSKRINTRIFVD---RG--RSGDNPRPGTIIDDVITLPERYDFYLVSQNVREGTIAPT  717

Query  3076  HYYVLHDTWTGKPDSFWPTVTHALTYNFCRSTTTVALPAPVLYAHLAAKRAKETL  3240
              Y V++DT +  PD     +T+ LT+ +   ++  V +P+   YAH  A  A  +L
Sbjct  718  SYNVIYDTTSLHPDRI-QRLTYKLTHMYFNNSCAVRVPSVCQYAHKLAFLAANSL  771

*************************************************************************************************
*********************************************************************
```

Query= gi|157674347|gb|EU143547.1| Bombyx mori PIWI mRNA, complete cds

```
Length=3533
                                                                      Score     E
Sequences producing significant alignments:                          (Bits)  Value

  ta_transcript51551_1                                                 1340   0.0
  ta_transcript44491_1                                                  213   2e-61



> ta_transcript51551_1
Length=1362

 Score =  1340 bits (3468),  Expect = 0.0, Method: Compositional matrix adjust.
 Identities = 625/777 (80%), Positives = 694/777 (89%), Gaps = 0/777 (0%)
 Frame = +1

Query  511   LPETISILRTRPEAVTSKKGTSGTPLDLLANYFTVETTPKWGLYQYHVDISPEEDSTGVR  690
             LPE ++ILRTRP  V SKKG++G PLD+ ANYFTVETTP+W LYQYHVD  PEEDSTG+R
Sbjct  13    LPEQMTILRTRPATVESKKGSTGDPLDVCANYFTVETTPQWCLYQYHVDFQPEEDSTGLR  72

Query  691   KALMRVHSKTLGGYLFDGTVLYTVNRLHPDPMELYSDRKTDNERMRILIKLTCEVSPGDY  870
             KAL+RVH+ TLGGYLFDG +LYTV RLHPDPMELYSDRK D ERMRILIKLTC+VSPGDY
Sbjct  73    KALLRVHANTLGGYLFDGAILYTVKRLHPDPMELYSDRKHDGERMRILIKLTCDVSPGDY  132

Query  871   HYIQIFNIIIRKCFNLLKLQLMGRDYFDPEAKIDIPEFKLQIWPGYKTTINQYEDRLLLV  1050
             HYIQ+FNIIIRKCFNLL LQL+GRDYFDP AK+DIPE KLQIWPGYKTTINQYEDRLL+V
Sbjct  133   HYIQVFNIIIRKCFNLLNLQLVGRDYFDPIAKVDIPEHKLQIWPGYKTTINQYEDRLLMV  192

Query  1051  TEIAHKVLRMDTVLQMLSEYAATKGNNYKKIFLEDVVGKIVMTDYNKRTYRVDDVAWNVS  1230
             TEIAHKVLRMD VLQML+EYAATKG+NYKKIFLEDVVGKIVMTDYNKRTYRVDDV W  S
Sbjct  193   TEIAHKVLRMDNVLQMLNEYAATKGSNYKKIFLEDVVGKIVMTDYNKRTYRVDDVHWETS  252

Query  1231  PKSTFKMRDENITYIEYYYKKYNLRIQDPGQPLLISRSKPREIRAGLPELIYLVPELCRQ  1410
             P+STFKM+DE + Y++YY KKYN+RIQDP QPLL+ R+K REIR+G+PEL+YLVPELCRQ
Sbjct  253   PRSTFKMKDETVCYMDYYQKKYNIRIQDPNQPLLVYRAKAREIRSGMPELVYLVPELCRQ  312

Query  1411  TGLSDEMRANFKLMRSLDVHTKIGPDKRIEKLNNFNRRFTSTPEVVEELATWSLKLSKEL  1590
             TGLSDEM ANF+LM +L  HTKIGPD RI+KL  FNRR T T EVV+ELA+WSLKLS +L
Sbjct  313   TGLSDEMLANFQLMSALGRHTKIGPDLRIQKLLQFNRRLTQTKEVVQELASWSLKLSNDL  372

Query  1591  VKIKGRQLPPENIIQANNVKYPAGDTTEGWTRDMRSKHLLAIAQLNSWVVITPERQRRDT  1770
             V+ KGRQLPPE IIQANNVKY AGDTTEGWTRDMRSK LL IA + SWVVITPERQRRD
Sbjct  373   VRFKGRQLPPEAIIQANNVKYAAGDTTEGWTRDMRSKPLLTIAPVGSWVVITPERQRRDA  432

Query  1771  ESFIDLIIKTGGGVGFRMRSPDLVVIRHDGPIEYANMCEEVIARKNPALILCVLARNYAD  1950
             ESF+DLI+KTG GVGFRM   P++   I   DG ++YANMCE VIARKNP+ ILCVL +   AD
Sbjct  433   ESFVDLIMKTGNGVGFRMPKPEICPIARDGHMDYANMCENVIARKNPSFILCVLRQKSAD  492

Query  1951  RYEAIKKKCTVDRAVPTQVVCARNMSSKSAMSIATKVAIQINCKLGGSPWTVDIPLPSLM  2130
             RYEAIKKKCTVDRA+PTQ V  RNM+SKSAMSIATK+AIQINCKLGG+PW+V++PLP+LM
Sbjct  493   RYEAIKKKCTVDRAMPTQCVVGRNMTSKSAMSIATKIAIQINCKLGGAPWSVEVPLPTLM  552

Query  2131  VVGYDVCHDTRSKEKSFGAFVATLDKQMTQYYSIVNAHTSGEELSSHMGFNIASAVKKFR  2310
             V+GYDVCHDTR+K+KSFGAFVATLDKQMT YYS VNAHTSGEELS+HMG NIA+AV+K+R
Sbjct  553   VIGYDVCHDTRAKDKSFGAFVATLDKQMTHYYSTVNAHTSGEELSAHMGINIATAVRKYR  612

Query  2311  EKNGTYPARIFIYRDGVGDGQIPYVHSHEVAEIKKKLAEIYAGVEIKLAFIIVSKRINTR  2490
             E+N    P RIFIYRDGVGDGQIPYVHSHEV EIKK+L  +Y G   +KLAFIIVSKRINTR
Sbjct  613   ERNNVLPGRIFIYRDGVGDGQIPYVHSHEVEEIKKQLETLYQGEPVKLAFIIVSKRINTR  672

Query  2491  IFVQRGRSGENPRPGTVIDDVVTLPERYDFYLVSQNVREGTIAPTSYNVIEDTTGLNPDR  2670
             IFV RGRSG+NPRPGT+IDDV+TLPERYDFYLVSQNVREGTIAPTSYNVI DTT L+PDR
Sbjct  673   IFVDRGRSGDNPRPGTIIDDVITLPERYDFYLVSQNVREGTIAPTSYNVIYDTTSLHPDR  732
```

```
Query  2671  IQRLTYKLTHLYFNCSSQVRVPSVCQYAHKLAFLAANSLHNQPHYSLNETLYFL*VF  2841
             IQRLTYKLTH+YFN S  VRVPSVCQYAHKLAFLAANSLHNQPHY+LNETLYFL*
Sbjct  733   IQRLTYKLTHMYFNNSCAVRVPSVCQYAHKLAFLAANSLHNQPHYTLNETLYFL*AL  789


> ta_transcript44491_1
Length=234

 Score =   213 bits (543),  Expect = 2e-61, Method: Compositional matrix adjust.
 Identities = 98/233 (42%), Positives = 147/233 (63%), Gaps = 0/233 (0%)
 Frame = +1

Query  2065  IQINCKLGGSPWTVDIPLPSLMVVGYDVCHDTRSKEKSFGAFVATLDKQMTQYYSIVNAH  2244
             +Q+NCKLGG+ W++ IP  + M+VG D  HD   K +S  +F+A+ ++ MTQ+YS
Sbjct  2     LQMNCKLGGTLWSIAIPFKTAMIVGIDSYHDAARKSRSVCSFIASYNQSMTQWYSRAIFQ  61

Query  2245  TSGEELSSHMGFNIASAVKKFREKNGTYPARIFIYRDGVGDGQIPYVHSHEVAEIKKKLA  2424
               G+E+   +   +  A+  +   NG  P R+ +YRDGVGDGQ+  +  +E+ +++   +
Sbjct  62    ERGQEVVDGLKSCLVDALTHYLRTNGKLPDRVIMYRDGVGDGQLNTIKQYEIPQMQVCFS  121

Query  2425  EIYAGVEIKLAFIIVSKRINTRIFVQRGRSGENPRPGTVIDDVVTLPERYDFYLVSQNVR  2604
              + A  +  L +++V KRINTRIF++     ENP PGTV+D  +T  + YDF + SQ V
Sbjct  122   LMEATYKPTLTYVVVQKRINTRIFMKVQGGFENPHPGTVVDHDITRRDWYDFLIASQKVN  181

Query  2605  EGTIAPTSYNVIEDTTGLNPDRIQRLTYKLTHLYFNCSSQVRVPSVCQYAHKL  2763
             +GT+ PT Y V+ D + +  D+ QRLTYKL HLY+N    VRVP+ CQYAHKL
Sbjct  182   QGTVTPTHYVVVHDDSAMTADQCQRLTYKLCHLYYNWPGTVRVPAPCQYAHKL  234


************************************************************************************************
**********************************************************************
Query= gi|392919217|ref|NM_072209.3| Caenorhabditis elegans Protein SAGO-1 (sago-1) mRNA,
complete cds

Length=2646
                                                                      Score     E
Sequences producing significant alignments:                          (Bits)  Value

  ta_transcript44491_1                                                63.5    2e-10
  ta_transcript51551_1                                                53.5    2e-06


> ta_transcript44491_1
Length=234

 Score = 63.5 bits (153),  Expect = 2e-10, Method: Compositional matrix adjust.
 Identities = 57/225 (25%), Positives = 99/225 (44%), Gaps = 20/225 (9%)
 Frame = +1

Query  1795  KTNMKLGGLNYELRSGVFSNSKRLIIGFETSQRGGLGDAPIAIGFAANMMSHSQQFAGGY  1974
             + N KLGG    L S        +I+G ++          +    A+   S +Q ++
Sbjct  3     QMNCKLGG---TLWSIAIPFKTAMIVGIDSYHDAARKSRSVCSFIASYNQSMTQWYSRA-  58

Query  1975  MFVKKSADNYGPVIPEILLTILKQAKAN--RPNDR-PDELLIYFSGVSEGQHALVNEYYA  2145
             +F ++     G  + + L + L  A  +  R N + PD +++Y  GV +GQ   + +Y
Sbjct  59    IFQER-----GQEVVDGLKSCLVDALTHYLRTNGKLPDRVIMYRDGVGDGQLNTIKQYEI  113

Query  2146  NQVKAACGLFNESFRPHITLILASKVHNTRVYKSENGGGVCNVEPGTVIDHTIVSPVLSE  2325
              Q++    L   +++P +T ++  K  NTR++     GG  N  PGTV+DH I      +
Sbjct  114   PQMQVCFSLMEATYKPTLTYVVVQKRINTRIFMKVQ-GGFENPHPGTVVDHDITR---RD  169

Query  2326  WYH---AGSLARQGTSKLVKYSLIFNTKKNEKLSVYERLTNELCY  2451
             WY    A     QGT     Y ++ +          +RLT +LC+
Sbjct  170   WYDFLIASQKVNQGTVTPTHYVVVHDDSAMTADQC-QRLTYKLCH  213


> ta_transcript51551_1
Length=1362

 Score = 53.5 bits (127),  Expect = 2e-06, Method: Compositional matrix adjust.
 Identities = 37/129 (29%), Positives = 59/129 (46%), Gaps = 2/129 (2%)
 Frame = +1

Query  2059  RPNDRPDELLIYFSGVSEGQHALVNEYYANQVKAACGLFNESFRPHITLILASKVHNTRV  2238
             R N  P  + IY  GV +GQ   V+ +   ++K       +     +  I+ SK  NTR+
```

```
Sbjct  614   RNNVLPGRIFIYRDGVGDGQIPYVHSHEVEEIKKQLETLYQGEPVKLAFIIVSKRINTRI  673

Query  2239  YKSENGGGVCNVEPGTVIDHTIVSPVLSEWYHAGSLARQGTSKLVKYSLIFNTKKNEKLS  2418
             +      G  N  PGT+ID  I  P   ++Y      R+GT     Y++I++T
Sbjct  674   FVDRGRSGD-NPRPGTIIDDVITLPERYDFYLVSQNVREGTIAPTSYNVIYDTTSLHPDR  732

Query  2419  VYERLTNEL  2445
             + +RLT +L
Sbjct  733   I-QRLTYKL  740
************************************************************************************************
***********************************************************************
Query= gi|392884665|ref|NM_058357.3| Caenorhabditis elegans Protein SAGO-2, isoform a (sago-2)
mRNA, complete cds

Length=2664
                                                                      Score     E
Sequences producing significant alignments:                          (Bits)  Value

  ta_transcript44491_1                                                60.5    3e-09
  ta_transcript51551_1                                                47.0    2e-04
  ta_transcript27100_1                                                32.3    6.9


> ta_transcript44491_1
Length=234

 Score = 60.5 bits (145),  Expect = 3e-09, Method: Compositional matrix adjust.
 Identities = 59/245 (24%), Positives = 106/245 (43%), Gaps = 15/245 (6%)
 Frame = +1

Query  1795  KTNMKLGGLNYAIGSEAFNKPNRLIVGFVTSQRVGGNPDYPISVGFAANMLKHHQKFAGG  1974
             + N KLGG  ++I   A      +IVG  +            S  F A+  +   ++
Sbjct  3     QMNCKLGGTLWSI---AIPFKTAMIVGIDSYHDAARKSRSVCS--FIASYNQSMTQWYSR  57

Query  1975  YVYVHRDRDVFGSIIKDTLLTIFKTCTEQRGR-PDDILLYFNGVSEGQFSMINEEFSARV  2151
              ++  R ++V   + K  L+          G+ PD +++Y +GV +GQ + I +++
Sbjct  58    AIFQERGQEVVDGL-KSCLVDALTHYLRTNGKLPDRVIMYRDGVGDGQLNTI-KQYEIPQ  115

Query  2152  KEACMAFQKEGTPPFRPHITIIASSKAHNERLYKSDKGRIVNLEPGTVVDHTIVSNVYTE  2331
              + C +    E T  ++P +T +   K  N R++   +G   N  PGTVVDH I    + +
Sbjct  116   MQVCFSLM-EAT--YKPTLTYVVVQKRINTRIFMKVQGGFENPHPGTVVDHDITRRDWYD  172

Query  2332  WYHASAVARQGTAKATKFTLIFTTKAGPQAEPLWHLEQLTNDLCYDHQIVFHPVGLPVPL  2511
             +  AS    QGT   T + ++    A    +     ++LT  LC+ +      V +P P
Sbjct  173   FLIASQKVNQGTVTPTHYVVVHDDSAMTADQ----CQRLTYKLCHLYYNWPGTVRVPAPC  228

Query  2512  YIADR  2526
               A +
Sbjct  229   QYAHK  233


> ta_transcript51551_1
Length=1362

 Score = 47.0 bits (110),  Expect = 2e-04, Method: Compositional matrix adjust.
 Identities = 107/486 (22%), Positives = 192/486 (40%), Gaps = 60/486 (12%)
 Frame = +1

Query  1054  DYFKKKYGITLKYPD--LFTIEAKGKQGKIHFPAEVLL---LCPNQTVTNDQMINNEQA-  1215
             DY++KKY I ++ P+  L    AK ++ +   P  V L   LC  QT  +D+M+ N Q
Sbjct  268   DYYQKKYNIRIQDPNQPLLVYRAKAREIRSGMPELVYLVPELC-RQTGLSDEMLANFQLM  326

Query  1216  -----------DMIKMSAAQPHIRKTTTDTIVRNVGLASNNIYGFIKVEDPVNLEGMVLP  1362
                        D+      Q + R T T  +V+ +   S  +       D V  +G  LP
Sbjct  327   SALGRHTKIGPDLRIQKLLQFNRRLTQTKEVVQELASWSLKLSN-----DLVRFKGRQLP  381

Query  1363  KPKIAFAGNRLADLANPKSRFPTDFNRAGQYYDAKELTKWELVFVQNEEVQGLAKQLADE  1542
              P+     N +   A   +   T   R+        +  W ++  + +      ++ A+
Sbjct  382   -PEAIIQANNVKYAAGDTTEGWTRDMRSKPLLTIAPVGSWVVITPERQ------RRDAES  434

Query  1543  MVNNGMKCSNPTMSFIIRGDLEPIFKKAKAAGTQLLFFVVKSRYNYHQQIKALEQK----  1710
              V+  MK  N     + + ++ PI +        +   V+ +R N    +  L QK
Sbjct  435   FVDLIMKTGNGVGFRMPKPEICPIARDGHMDYANMCENVI-ARKNPSFILCVLRQKSADR  493

Query  1711  YDVLTQEI---RAETAEKVFRQPQTR---LNIINKT----NMKLGGLNYAIGSEAFNKPN  1860
             Y+ + ++    RA   + V  +  T    ++I  K     N KLGG  +++       P
Sbjct  494   YEAIKKKCTVDRAMPTQCVVGRNMTSKSAMSIATKIAIQINCKLGGAPWSVEVPL---PT  550
```

```
Query  1861  RLIVGFVTSQRVGGNPDYPISVG-FAANMLKHHQKFAGGYVYVHRDRDVFGSIIKDTLLT  2037
              +++G+              S G F A + K    +    V  H   +   + +   + T
Sbjct  551   LMVIGYDVCHDTRAKDK---SFGAFVATLDKQMTHYYST-VNAHTSGEELSAHMGINIAT  606

Query  2038  IFKTCTEQRG-RPDDILLYFNGVSEGQFSMINEEFSARVKEACMAFQKEGTPPFRPHITI  2214
               +   E+    P  I +Y +GV +GQ   ++      +K+  +    +G P     +
Sbjct  607   AVRKYRERNNVLPGRIFIYRDGVGDGQIPYVHSHEVEEIKKQ-LETLYQGEPV---KLAF  662

Query  2215  IASSKAHNERLYKSDKGRI-VNLEPGTVVDHTIVSNVYTEWYHASAVARQGTAKATKFTL  2391
             I  SK  N R++  D+GR   N  PGT++D  I      ++Y  S   R+GT   T + +
Sbjct  663   IIVSKRINTRIF-VDRGRSGDNPRPGTIIDDVITLPERYDFYLVSQNVREGTIAPTSYNV  721

Query  2392  IFTTKA  2409
             I+ T +
Sbjct  722   IYDTTS  727


> ta_transcript27100_1
Length=877

 Score = 32.3 bits (72),  Expect = 6.9, Method: Compositional matrix adjust.
 Identities = 18/50 (36%), Positives = 26/50 (52%), Gaps = 0/50 (0%)
 Frame = +3

Query  2061  ARKTR*YPFVFQWSFRRSILNDQRGIQCPSEGGVHGIPKGGNPAIQTTHH  2210
             +R+ R  PFVF+   +  + N Q+  Q P  G VHG P+ G   +   HH
Sbjct  679   SRRHRA*PFVFRQFNQLRVFNYQQAAQQPDVGAVHGGPRPGADHVLREHH  728

************************************************************************************************
***********************************************************************
Query= gi|392885315|ref|NM_170850.3| Caenorhabditis elegans Protein PPW-1, isoform b (ppw-1)
mRNA, complete cds

Length=165
                                                                      Score     E
Sequences producing significant alignments:                          (Bits)  Value

  ta_transcript72377_1                                                  27.7    1.6
  ta_transcript72378_1                                                  27.7    1.8
  ta_transcript42528_1                                                  26.6    3.6
  ta_transcript60738_1                                                  26.6    4.4
  ta_transcript74966_1                                                  25.4    8.7


> ta_transcript72377_1
Length=1495

 Score = 27.7 bits (60),  Expect = 1.6, Method: Composition-based stats.
 Identities = 13/26 (50%), Positives = 18/26 (69%), Gaps = 5/26 (19%)
 Frame = +3

Query  3     GKTTRSYVRLGQTCCPSCPKAWYRSA  80
             G++TR    L QTCCP C  AWY+++
Sbjct  1173  GRSTRC---LEQTCCPRC--AWYQTS  1193


> ta_transcript72378_1
Length=1465

 Score = 27.7 bits (60),  Expect = 1.8, Method: Composition-based stats.
 Identities = 13/26 (50%), Positives = 18/26 (69%), Gaps = 5/26 (19%)
 Frame = +3

Query  3     GKTTRSYVRLGQTCCPSCPKAWYRSA  80
             G++TR    L QTCCP C  AWY+++
Sbjct  1143  GRSTRC---LEQTCCPRC--AWYQTS  1163


> ta_transcript42528_1
Length=450

 Score = 26.6 bits (57),  Expect = 3.6, Method: Compositional matrix adjust.
 Identities = 13/22 (59%), Positives = 16/22 (73%), Gaps = 1/22 (5%)
 Frame = +2

Query  56   PKSLVPLRSLQKRREMW-RGEP  118
```

```
            P SLVP R+L+  R MW +GEP
Sbjct  294  PSSLVPERNLRGVRMMWNKGEP  315


> ta_transcript60738_1
Length=610

 Score = 26.6 bits (57),  Expect = 4.4, Method: Composition-based stats.
 Identities = 12/33 (36%), Positives = 20/33 (61%), Gaps = 2/33 (6%)
 Frame = -2

Query  110  LSTFLVF--FAASGAVPSFWAAGAAGLSETNIA  18
            L+T+ +F  F A G + SFW+    GL  ++I+
Sbjct  198  LNTYYIFQLFYAQGLIVSFWSIAQCGLRNSSIS  230


> ta_transcript74966_1
Length=528

 Score = 25.4 bits (54),  Expect = 8.7, Method: Composition-based stats.
 Identities = 8/19 (42%), Positives = 12/19 (63%), Gaps = 0/19 (0%)
 Frame = +3

Query  36   QTCCPSCPKAWYRSARCKK  92
            Q  CPS P+ W+ S+ C +
Sbjct  213  QPTCPSKPRGWWTSSLCSR  231


***************************************************************************************************
***********************************************************************
Query= gi|392885541|ref|NM_059134.5| Caenorhabditis elegans Protein PPW-2 (ppw-2) mRNA, complete
cds

Length=2928
                                                                      Score     E
Sequences producing significant alignments:                          (Bits)  Value

  ta_transcript44491_1                                                63.2    3e-10
  ta_transcript51551_1                                                57.4    2e-07


> ta_transcript44491_1
Length=234

 Score = 63.2 bits (152),  Expect = 3e-10, Method: Compositional matrix adjust.
 Identities = 53/215 (25%), Positives = 86/215 (40%), Gaps = 29/215 (13%)
 Frame = +1

Query  2134  AYNGNGKQEFSGDFVLNAAGQETIAPIEDIVSYSIKGYKKFHDGKAPKRITIYRSGSSEG  2313
             +YN +  Q +S   +    GQE +  ++  +  ++   Y +  +GK P R+ +YR G  +G
Sbjct  46    SYNQSMTQWYSRA-IFQERGQEVVDGLKSCLVDALTHYLRT-NGKLPDRVIMYRDGVGDG  103

Query  2314  NHGPIISYEVPLARV--AMRNFSPDTQLLYIVVSKEHTYRFFKKEsggsssggsnsagas  2487
                 I  YE+P  +V  ++   +    L Y+VV K    R F K  GG
Sbjct  104   QLNTIKQYEIPQMQVCFSLMEATYKPTLTYVVVQKRINTRIFMKVQGGFE----------  153

Query  2488  nsgTLTSAPPKPWELNIGPGLTVDYGVTNPACKQFFLNSHMTLQGSAKTPLYTVLADDRN  2667
                            N  PG  VD+ +T      F + S    QG+     Y V+ DD
Sbjct  154   ---------------NPHPGTVVDHDITRRDWYDFLIASQKVNQGTVTPTHYVVVHDDSA  198

Query  2668  IGMSALEEFTFNLCHLHQIVGLPTSIPTPLYVANE  2772
             +     +  T+ LCHL+         +P P   A++
Sbjct  199   MTADQCQRLTYKLCHLYYNWPGTVRVPAPCQYAHK  233


> ta_transcript51551_1
Length=1362

 Score = 57.4 bits (137),  Expect = 2e-07, Method: Compositional matrix adjust.
 Identities = 129/608 (21%), Positives = 238/608 (39%), Gaps = 113/608 (19%)
 Frame = +1

Query  1090  SNYRGRTRHHKIESIHHEGAATARFELEGGGTCTVATYFKDKYKIQLRYPNANLIV--CK  1263
             ++Y  RT  ++++ +H E +  + F+++    C +  Y++ KY I+++ PN  L+V  K
Sbjct  235   TDYNKRT--YRVDDVHWETSPRSTFKMKDETVCYM-DYYQKKYNIRIQDPNQPLLVYRAK  291

Query  1264  ERGALNFYPMELITISPNqrvri-----tqqtssqsqrttKESAVLPDIR-QRLIMTGKI  1425
```

```
              R   +  P EL+ + P       +               + + + PD+R Q+L+   +
Sbjct  292   AREIRSGMP-ELVYLVPELCRQTGLSDEMLANFQLMSALGRHTKIGPDLRIQKLLQFNR-  349

Query  1426  AAKITAENEV---LGKMGVTVCDEPLVVKGRNLPA--------IRLASFETGEHLINPRD  1572
               ++T   EV   L    + + ++ +  KGR LP         ++ A+ +T E     RD
Sbjct  350   --RLTQTKEVVQELASWSLKLSNDLVRFKGRQLPPEAIIQANNVKYAAGDTTEGWT--RD  405

Query  1573  CKWRPQRYNRSAVAPK-VWALYGVGSPGSQMNRDVMRRFCDEFMNMSRSKGILFPPPGDV  1749
              + +P       +AP   W    V +P  Q  RD    F D  M      G   P P
Sbjct  406   MRSKPLL----TIAPVGSWV---VITPERQ-RRDA-ESFVDLIMKTGNGVGFRMPKPE--  454

Query  1750  NLLTPDAIENRLREA-------ANAGCTFVLCITEDNITCLHQKYKFIEHH--------T  1884
               + P A +  +  A       A    +F+LC+          +Y+ I+          T
Sbjct  455   --ICPIARDGHMDYANMCENVIARKNPSFILCVLRQKSA---DRYEAIKKKCTVDRAMPT  509

Query  1885  QMIV-QDMKLSKALSVVNASKKLTLENVINKTNVKLGGSNYVYLDTKNFLQEHLIIGVGI  2061
             Q +V ++M    A+S+   + K+ ++      N KLGG+ +     +  L   ++IG  +
Sbjct  510   QCVVGRNMTSKSAMSI---ATKIAIQ-----INCKLGGAPW---SVEVPLPTLMVIGYDV  558

Query  2062  SSPPPGTKYIMESRGILNPTIVGFAYNGNGKQEFSGDFVLNA--AGQETIAPIEDIVSYS  2235
                   T+   +S G    T+             KQ   +NA  +G+E  A +   ++ +
Sbjct  559   CH---DTRAKDKSFGAFVATL--------DKQMTHYYSTVNAHTSGEELSAHMGINIATA  607

Query  2236  IKGYKKFHDGKAPKRITIYRSGSSEGNHGPIISYEVPLARVAMRNF--SPDTQLLYIVVS  2409
             ++ Y++  +   P RI IYR G  +G    + S+EV   +  +         +L +I+VS
Sbjct  608   VRKYRE-RNNVLPGRIFIYRDGVGDGQIPYVHSHEVEEIKKQLETLYQGEPVKLAFIIVS  666

Query  2410  KEHTYRFFKKEsggsssggsnsagasnsgTLTSAPPKPWELNIGPGLTVDYGVTNPACKQ  2589
             K    R F                                 N  PG  +D  +T P
Sbjct  667   KRINTRIFVDRGRSGD-------------------------NPRPGTIIDDVITLPERYD  701

Query  2590  FFLNSHMTLQGSAKTPLYTVLADDRNIGMSALEEFTFNLCHLHQIVGLPTSIPTPLYVAN  2769
             F+L S    +G+     Y V+ D  ++    ++  T+ L H++        +P+    A+
Sbjct  702   FYLVSQNVREGTIAPTSYNVIYDTTSLHPDRIQRLTYKLTHMYFNNSCAVRVPSVCQYAH  761

Query  2770  EYAKRGRN  2793
             + A    N
Sbjct  762   KLAFLAAN  769

************************************************************************************************
**********************************************************************
```

Query= gi|429892795|gb|KC116214.1| Drosophila melanogaster isolate 3893 aubergine (Aub) gene, complete cds

```
Length=3434
                                                                      Score     E
Sequences producing significant alignments:                          (Bits)  Value

  ta_transcript51551_1                                                  320   3e-90
  ta_transcript44491_1                                                  184   5e-51


> ta_transcript51551_1
Length=1362

 Score =   320 bits (821),  Expect = 3e-90, Method: Compositional matrix adjust.
 Identities = 161/304 (53%), Positives = 208/304 (68%), Gaps = 14/304 (5%)
 Frame = +3

Query  2535  YSCIKKRTCVDRPVPSQVVTLKVIAPRQQKPTGLMSIATKVVIQMNAKLMGAPWQVVIPL  2714
             Y  IKK+  VDR +P+Q V       R       MSIATK+ IQ+N KL GAPW V +PL
Sbjct  494   YEAIKKKCTVDRAMPTQCV-----VGRNMTSKSAMSIATKIAIQINCKLGGAPWSVEVPL  548

Query  2715  HGLMTVGFDVCHSPKNKNKSYGAFVATMDQKESFRYFSTVNEHIKGQELSEQMSVNMACA  2894
               LM +G+DVCH  + K+KS+GAFVAT+D K+   Y+STVN H  G+ELS  M +N+A A
Sbjct  549   PTLMVIGYDVCHDTRAKDKSFGAFVATLD-KQMTHYYSTVNAHTSGEELSAHMGINIATA  607

Query  2895  LRSYQEQHRSLPERILFFRDGVGDGQLYQVVNSEVNTLKDRLDEIYKSAGKQEGCRMTFI  3074
             +R Y+E++  LP RI  +RDGVGDGQ+  V + EV  +K +L+ +Y+     E  ++ FI
Sbjct  608   VRKYRERNNVLPGRIFIYRDGVGDGQIPYVHSHEVEEIKKQLETLYQG----EPVKLAFI  663

Query  3075  IVSKRINSRYFTGH----RNPVPGTVVDDVITLPERYDFFLVSQAVRIGTVSPTSYNVIS  3242
             IVSKRIN+R F        NP PGT++DDVITLPERYDF+LVSQ VR GT++PTSYNVI
Sbjct  664   IVSKRINTRIFVDRGRSGDNPRPGTIIDDVITLPERYDFYLVSQNVREGTIAPTSYNVIY  723

Query  3243  DNMGLNADKLQMLSYKMTHMYYNYSGTIRVPAVCHYAHKLAFLVAESINRAPSAGLQNQL  3422
             D   L+ D++Q L+YK+THMY+N S  +RVP+VC YAHKLAFL A S++  P   L   L
```

```
Sbjct  724   DTTSLHPDRIQRLTYKLTHMYFNNSCAVRVPSVCQYAHKLAFLAANSLHNQPHYTLNETL  783

Query  3423  YFL*  3434
             YFL*
Sbjct  784   YFL*  787


 Score =   111 bits (278),  Expect(4) = 4e-74, Method: Compositional matrix adjust.
 Identities = 59/148 (40%), Positives = 83/148 (56%), Gaps = 6/148 (4%)
 Frame = +2

Query  761   SRPPGMTSKKGVVGTHITVQANYFKVLKRPNWTIYQYRVDFTPDVEATRLRRSFLYEHKG  940
             +RP  + SKKG  G  + V ANYF V   P W +YQY VDF P+ ++T LR++ L  H
Sbjct  22    TRPATVESKKGSTGDPLDVCANYFTVETTPQWCLYQYHVDFQPEEDSTGLRKALLRVHAN  81

Query  941   ILGGYIFDGTNMFCINQFKAVQDSPYVLELVT-KSRAGENIEIKIKAVGSVQSTDAEQFQ  1117
              LGGY+FDG  ++ + +       P  +EL + +   GE + I IK    V   D    Q
Sbjct  82    TLGGYLFDGAILYTVKRLH-----PDPMELYSDRKHDGERMRILIKLTCDVSPGDYHYIQ  136

Query  1118  VLNLILRRAMEGLDLKLVSRYYYDPQAK  1201
             V N+I+R+    L+L+LV R Y+DP AK
Sbjct  137   VFNIIIRKCFNLLNLQLVGRDYFDPIAK  164


 Score = 90.9 bits (224),  Expect(4) = 4e-74, Method: Compositional matrix adjust.
 Identities = 43/128 (34%), Positives = 69/128 (54%), Gaps = 1/128 (1%)
 Frame = +3

Query  1920  LRAMSEHTRLNPDRRIERLRMFNKRLKSCKQSVETLKSWNIELDSALVEIPARVLPPEKI  2099
             + A+  HT++ PD RI++L  FN+RL   K+ V+ L SW+++L + LV    R LPPE I
Sbjct  326   MSALGRHTKIGPDLRIQKLLQFNRRLTQTKEVVQELASWSLKLSNDLVRFKGRQLPPEAI  385

Query  2100  LFGNQ-KIFVCDARADWTNEFRTCSMFKNVHINRWYVITPSRNLRETQEFVQMCIRTASS  2276
             +  N  K    D    WT + R+  +     +  W VITP R  R+ + FV + ++T +
Sbjct  386   IQANNVKYAAGDTTEGWTRDMRSKPLLTIAPVGSWVVITPERQRRDAESFVDLIMKTGNG  445

Query  2277  MKMNICNP  2300
             +   +  P
Sbjct  446   VGFRMPKP  453


 Score = 88.6 bits (218),  Expect(4) = 4e-74, Method: Compositional matrix adjust.
 Identities = 41/110 (37%), Positives = 65/110 (59%), Gaps = 18/110 (16%)
 Frame = +2

Query  1517  VILTDYNNKTYRIDDVDFQSTPLCKFKTNDGEISYVDYYKKXXXXXXXXXXXXXXXXXXR  1696
             +++TDYN +TYR+DDV ++++P   FK  D  + Y+DYY+K                  +
Sbjct  232   IVMTDYNKRTYRVDDVHWETSPRSTFKMKDETVCYMDYYQK------------------K  273

Query  1697  YNIIIRDLKQPLVMSRPTDKNIRGGNDQAIMIIPELARATGMTDAMRADF  1846
             YNI I+D  QPL++ R   + IR G  + + ++PEL R TG++D M A+F
Sbjct  274   YNIRIQDPNQPLLVYRAKAREIRSGMPELVYLVPELCRQTGLSDEMLANF  323


 Score = 52.4 bits (124),  Expect(4) = 4e-74, Method: Compositional matrix adjust.
 Identities = 21/65 (32%), Positives = 43/65 (66%), Gaps = 1/65 (2%)
 Frame = +3

Query  1260  INLENFRMQLWPGYQTSIRQHENDILLCSEICHKVMRTETLYNILSD-AIRDSDDYQSTF  1436
             +++   ++Q+WPGY+T+I Q+E+ +L+ +EI HKV+R + +  +L++ A     +Y+  F
Sbjct  165   VDIPEHKLQIWPGYKTTINQYEDRLLMVTEIAHKVLRMDNVLQMLNEYAATKGSNYKKIF  224

Query  1437  KRAVM  1451
                V+
Sbjct  225   LEDVV  229


> ta_transcript44491_1
Length=234

 Score =   184 bits (466),  Expect = 5e-51, Method: Compositional matrix adjust.
 Identities = 96/242 (40%), Positives = 142/242 (59%), Gaps = 15/242 (6%)
 Frame = +3

Query  2658  VIQMNAKLMGAPWQVVIPLHGLMTVGFDVCHSPKNKNKSYGAFVATMDQKESFRYFSTVN  2837
             ++QMN KL G  W + IP    M VG D  H    K++S  +F+A+ +Q  + +++S
Sbjct  1     LLQMNCKLGGTLWSIAIPFKTAMIVGIDSYHDAARKSRSVCSFIASYNQSMT-QWYSRAI  59
```

```
Query  2838  EHIKGQELSEQMSVNMACALRSYQEQHRSLPERILFFRDGVGDGQLYQVVNSEVNTLK--  3011
                +GQE+ + +   +  AL  Y   +  LP+R++ +RDGVGDGQL  +   E+  ++
Sbjct  60    FQERGQEVVDGLKSCLVDALTHYLRTNGKLPDRVIMYRDGVGDGQLNTIKQYEIPQMQVC  119

Query  3012  -DRLDEIYKSAGKQEGCRMTFIIVSKRINSRYFT----GHRNPVPGTVVDDVITLPERYD  3176
                ++  YK         +T+++V KRIN+R F     G  NP PGTVVD  IT  + YD
Sbjct  120   FSLMEATYKPT-------LTYVVVQKRINTRIFMKVQGGFENPHPGTVVDHDITRRDWYD  172

Query  3177  FFLVSQAVRIGTVSPTSYNVISDNMGLNADKLQMLSYKMTHMYYNYSGTIRVPAVCHYAH  3356
             F + SQ V  GTV+PT Y V+ D+   + AD+ Q L+YK+ H+YYN+ GT+RVPA C YAH
Sbjct  173   FLIASQKVNQGTVTPTHYVVVHDDSAMTADQCQRLTYKLCHLYYNWPGTVRVPAPCQYAH  232

Query  3357  KL  3362
             KL
Sbjct  233   KL  234
```

****
****

Query= gi|166706855|ref|NM_001104596.2| Bombyx mori aubergine protein (Aubergine), mRNA

Length=2700

```
                                                                      Score     E
Sequences producing significant alignments:                          (Bits)  Value

  ta_transcript51551_1                                                 1340   0.0
  ta_transcript44491_1                                                  213   3e-62
```

> ta_transcript51551_1
Length=1362

 Score = 1340 bits (3468), Expect = 0.0, Method: Compositional matrix adjust.
 Identities = 625/775 (81%), Positives = 694/775 (90%), Gaps = 0/775 (0%)
 Frame = +1

```
Query  376   LPETISILRTRPEAVTSKKGTSGTPLDLLANYFTVETTPKWGLYQYHVDISPEEDSTGVR  555
             LPE ++ILRTRP  V SKKG++G PLD+ ANYFTVETTP+W LYQYHVD  PEEDSTG+R
Sbjct  13    LPEQMTILRTRPATVESKKGSTGDPLDVCANYFTVETTPQWCLYQYHVDFQPEEDSTGLR  72

Query  556   KALMRVHSKTLGGYLFDGTVLYTVNRLHPDPMELYSDRKTDNERMRILIKLTCEVSPGDY  735
             KAL+RVH+ TLGGYLFDG +LYTV RLHPDPMELYSDRK D ERMRILIKLTC+VSPGDY
Sbjct  73    KALLRVHANTLGGYLFDGAILYTVKRLHPDPMELYSDRKHDGERMRILIKLTCDVSPGDY  132

Query  736   HYIQIFNIIIRKCFNLLKLQLMGRDYFDPEAKIDIPEFKLQIWPGYKTTINQYEDRLLLV  915
             HYIQ+FNIIIRKCFNLL LQL+GRDYFDP AK+DIPE KLQIWPGYKTTINQYEDRLL+V
Sbjct  133   HYIQVFNIIIRKCFNLLNLQLVGRDYFDPIAKVDIPEHKLQIWPGYKTTINQYEDRLLMV  192

Query  916   TEIAHKVLRMDTVLQMLSEYAATKGNNYKKIFLEDVVGKIVMTDYNKRTYRVDDVAWNVS  1095
             TEIAHKVLRMD VLQML+EYAATKG+NYKKIFLEDVVGKIVMTDYNKRTYRVDDV W  S
Sbjct  193   TEIAHKVLRMDNVLQMLNEYAATKGSNYKKIFLEDVVGKIVMTDYNKRTYRVDDVHWETS  252

Query  1096  PKSTFKMRDENITYIEYYYKKYNLRIQDPGQPLLISRSKPREIRAGLPELIYLVPELCRQ  1275
             P+STFKM+DE  + Y++YY KKYN+RIQDP QPLL+ R+K REIR+G+PEL+YLVPELCRQ
Sbjct  253   PRSTFKMKDETVCYMDYYQKKYNIRIQDPNQPLLVYRAKAREIRSGMPELVYLVPELCRQ  312

Query  1276  TGLSDEMRANFKLMRSLDVHTKIGPDKRIEKLNNFNRRFTSTPEVVEELATWSLKLSKEL  1455
             TGLSDEM ANF+LM +L  HTKIGPD RI+KL  FNRR T T EVV+ELA+WSLKLS +L
Sbjct  313   TGLSDEMLANFQLMSALGRHTKIGPDLRIQKLLQFNRRLTQTKEVVQELASWSLKLSNDL  372

Query  1456  VKIKGRQLPPENIIQANNVKYPAGDTTEGWTRDMRSKHLLAIAQLNSWVVITPERQRRDT  1635
             V+ KGRQLPPE IIQANNVKY AGDTTEGWTRDMRSK LL IA + SWVVITPERQRRD
Sbjct  373   VRFKGRQLPPEAIIQANNVKYAAGDTTEGWTRDMRSKPLLTIAPVGSWVVITPERQRRDA  432

Query  1636  ESFIDLIIKTGGGVGFRMRSPDLVVIRHDGPIEYANMCEEVIARKNPALILCVLARNYAD  1815
             ESF+DLI+KTG GVGFRM  P++  I  DG ++YANMCE VIARKNP+ ILCVL +  AD
Sbjct  433   ESFVDLIMKTGNGVGFRMPKPEICPIARDGHMDYANMCENVIARKNPSFILCVLRQKSAD  492

Query  1816  RYEAIKKKCTVDRAVPTQVVCARNMSSKSAMSIATKVAIQINCKLGGSPWTVDIPLPSLM  1995
             RYEAIKKKCTVDRA+PTQ V  RNM+SKSAMSIATK+AIQINCKLGG+PW+V++PLP+LM
Sbjct  493   RYEAIKKKCTVDRAMPTQCVVGRNMTSKSAMSIATKIAIQINCKLGGAPWSVEVPLPTLM  552

Query  1996  VVGYDVCHDTRSKEKSFGAFVATLDKQMTQYYSIVNAHTSGEELSSHMGFNIASAVKKFR  2175
             V+GYDVCHDTR+K+KSFGAFVATLDKQMT YYS VNAHTSGEELS+HMG NIA+AV+K+R
Sbjct  553   VIGYDVCHDTRAKDKSFGAFVATLDKQMTHYYSTVNAHTSGEELSAHMGINIATAVRKYR  612

Query  2176  EKNGTYPARIFIYRDGVGDGQIPYVHSHEVAEIKKKLAEIYAGVEIKLAFIIVSKRINTR  2355
             E+N    P RIFIYRDGVGDGQIPYVHSHEV EIKK+L  +Y G  +KLAFIIVSKRINTR
Sbjct  613   ERNNVLPGRIFIYRDGVGDGQIPYVHSHEVEEIKKQLETLYQGEPVKLAFIIVSKRINTR  672
```

```
Query  2356   IFVQRGRSGENPRPGTVIDDVVTLPERYDFYLVSQNVREGTIAPTSYNVIEDTTGLNPDR  2535
              IFV RGRSG+NPRPGT+IDDV+TLPERYDFYLVSQNVREGTIAPTSYNVI DTT L+PDR
Sbjct  673    IFVDRGRSGDNPRPGTIIDDVITLPERYDFYLVSQNVREGTIAPTSYNVIYDTTSLHPDR  732

Query  2536   IQRLTYKLTHLYFNCSSQVRVPSVCQYAHKLAFLAANSLHNQPHYSLNETLYFL*  2700
              IQRLTYKLTH+YFN S  VRVPSVCQYAHKLAFLAANSLHNQPHY+LNETLYFL*
Sbjct  733    IQRLTYKLTHMYFNNSCAVRVPSVCQYAHKLAFLAANSLHNQPHYTLNETLYFL*  787


> ta_transcript44491_1
Length=234

 Score =   213 bits (543),  Expect = 3e-62, Method: Compositional matrix adjust.
 Identities = 98/233 (42%), Positives = 147/233 (63%), Gaps = 0/233 (0%)
 Frame = +1

Query  1930   IQINCKLGGSPWTVDIPLPSLMVVGYDVCHDTRSKEKSFGAFVATLDKQMTQYYSIVNAH  2109
              +Q+NCKLGG+ W++ IP  + M+VG D  HD   K +S  +F+A+ ++ MTQ+YS
Sbjct  2      LQMNCKLGGTLWSIAIPFKTAMIVGIDSYHDAARKSRSVCSFIASYNQSMTQWYSRAIFQ  61

Query  2110   TSGEELSSHMGFNIASAVKKFREKNGTYPARIFIYRDGVGDGQIPYVHSHEVAEIKKKLA  2289
                G+E+    +   +  A+  +    NG   P R+ +YRDGVGDGQ+  +  +E+ +++   +
Sbjct  62     ERGQEVVDGLKSCLVDALTHYLRTNGKLPDRVIMYRDGVGDGQLNTIKQYEIPQMQVCFS  121

Query  2290   EIYAGVEIKLAFIIVSKRINTRIFVQRGRSGENPRPGTVIDDVVTLPERYDFYLVSQNVR  2469
                + A  +  L +++V KRINTRIF++      ENP PGTV+D  +T  + YDF + SQ V
Sbjct  122    LMEATYKPTLTYVVVQKRINTRIFMKVQGGFENPHPGTVVDHDITRRDWYDFLIASQKVN  181

Query  2470   EGTIAPTSYNVIEDTTGLNPDRIQRLTYKLTHLYFNCSSQVRVPSVCQYAHKL  2628
              +GT+ PT Y V+ D + +  D+ QRLTYKL HLY+N     VRVP+ CQYAHKL
Sbjct  182    QGTVTPTHYVVVHDDSAMTADQCQRLTYKLCHLYYNWPGTVRVPAPCQYAHKL  234

***************************************************************************************************
************************************************************************
Query= gi|665409862|ref|NM_139559.4| Drosophila melanogaster armitage (armi), transcript variant
D, mRNA

Length=4151
                                                                      Score     E
Sequences producing significant alignments:                          (Bits)  Value

  ta_transcript31548_1                                                67.4    2e-10
  ta_transcript31554_1                                                52.8    6e-07
  ta_transcript55901_1                                                43.9    0.003
  ta_transcript55900_1                                                43.9    0.004
  ta_transcript31550_1                                                40.0    0.033


> ta_transcript31548_1
Length=534

 Score = 67.4 bits (163),  Expect = 2e-10, Method: Compositional matrix adjust.
 Identities = 69/248 (28%), Positives = 115/248 (46%), Gaps = 42/248 (17%)
 Frame = +1

Query  2020   TSERRQEMIKAIEQHYSF-LTEPLSIKTYMHRFRLLLHLEEIECFVNFRNYDRDRAHFLR  2196
               S++ QE+ K I+   ++   +TE     + Y+   F   LL  EE    +N +NY+       +R
Sbjct  307    ASKQEQELTKDIKDIFAVGVTE----ENYVRFFHNLLWYEETIVRINLKNYNMSSVPLVR  362

Query  2197   DGE-FLT--------------LQIENLAERRPSLVIGDTLRVINPWSDPDSQTTKSYEGI  2331
                + F+T                L +  LAE+RPSL++GD L      +  P         YE I
Sbjct  363    RVDAFVTVPGTVKKGTSYSYALVVPGLAEKRPSLMLGDLL-----FVKPVESDEVMYEAI  417

Query  2332   IHKVLFDRILLK-FHSSFQEKYN-GEDYRLE--FYFSRYSFRKQHHAISKIV-GVMGEDF  2496
              I ++   + + LK FH  F++ +  G + R +  F+ SR    + H A+    V G    +
Sbjct  418    ITEMEDNVVNLKGFHPEFEKHFKPGHEQRFDVRFFMSRMPLERMHRAVQAAVQGSSEQRR  477

Query  2497   LFPSKVTKRENPQLDVYMKDDDMYLYDSKLEWYNQSLNSIQKRAVFNILRGEAENIPYVL  2676
              +FP+  +   +P    ++    +L +   E           Q+ AV +I+ G A    PY+L
Sbjct  478    VFPAASS---DPPAQYQVQSFFNHLVEENPE---------QRSAVEHIVGGTAGAAPYLL  525

Query  2677   FGPPGSGK  2700
               GPPG+GK
Sbjct  526    HGPPGTGK  533
```

```
> ta_transcript31554_1
Length=123

 Score = 52.8 bits (125),  Expect = 6e-07, Method: Compositional matrix adjust.
 Identities = 29/78 (37%), Positives = 43/78 (55%), Gaps = 9/78 (12%)
 Frame = +1

Query  3754  QERDIILISTVRSSEEILRMDARFSLGFVRCSKRLNVAVSRARAMMIIFGNPHLLAVDEC  3933
             +E+ +I+ISTVRS  +         +GF+   +R NVA++RA+A  I+ GNP  L  D
Sbjct  1     KEKRVIIISTVRSGAQ---------MGFLVDDQRFNVALTRAKAKAIVIGNPLCLQRDLK  51

Query  3934  WRQLILFCVKNNAYFGCD  3987
             WR  +  C    +Y G D
Sbjct  52    WRAYMQLCRAYGSYCGHD  69


> ta_transcript55901_1
Length=1263

 Score = 43.9 bits (102),  Expect = 0.003, Method: Compositional matrix adjust.
 Identities = 39/114 (34%), Positives = 51/114 (45%), Gaps = 0/114 (0%)
 Frame = -1

Query  3986  SQPK*ALFLTQNRISWRQHSSTARRWGFPKMIIMARARETATLSRLLQRTNPKEKRASMR  3807
             S PK* +      +  R   S  R   FP +I +A AR +ATL+ L     P    AS
Sbjct  328   SWPK*QVLSLHKSM*HRHFLSLMRHAAFPIIISLALARVSATLNLLSSTRKPSLYLAS*S  387

Query  3806  RISSDERTVDISIMSRSCP*NSSTDPIFGMTTSVPMNILRSIFTCF**GVIIPI  3645
                   RTVD  +   S P N ST P      S  +N  R ++TC  *G +IP+
Sbjct  388   SRRQLARTVDTRMTLFSLPWNDSTVPTSTALYSFCVNQSRILYTCLR*GDMIPM  441


 Score = 35.8 bits (81),  Expect = 1.0, Method: Compositional matrix adjust.
 Identities = 66/219 (30%), Positives = 89/219 (41%), Gaps = 27/219 (12%)
 Frame = -1

Query  3257  SRKDMENPIFEAIRLVTIDCSWRGSPERTT*ERFFVSITMGTIVSGSVH*PASS------  3096
             SR D+ +P+F A R +    S  GSPE T+          MGT  +GS    ASS
Sbjct  554   SRSDIPSPLFAASREMHTGPSCLGSPESTSCPVGASRPLMGTSTAGSSACAASSMNRCVM  613

Query  3095  NST*VKWPAGNPNCKKFPKVVQVEMVMR*VPINLA*QRSFNPDSVTIILS-SQLPTAPTS  2919
             +S  + W   +P   +  KVV                R+    S+ I L  S+     T
Sbjct  614   DSLNLNWLESDPYLAECSKVVTT-------------IRNMERSSLCIDLKVSRFAPFDTG  660

Query  2918  LVAQ*LIISGGIKSFST*LYETRRMKSPWSKALLSM-SLFVTKSAELFDGVPTKIRAPGR  2742
                   I SGG+   S      RRM       LL   S+    SA + + V T  R  G
Sbjct  661   ------ISSGGMILHSRFSELARRMNLLILSCLLYFSSISAMWSAAMLESVHTSTRFFGF  714

Query  2741  FLTNWSSVSISVIVFPLPGGPNST*GMFSASPRSILKTA  2625
               T  +  S +VIV P+PGGPN+ *G+      +I  TA
Sbjct  715   IATICTIASTTVIVLPVPGGPNTM*GLLPDVLETICSTA  753


> ta_transcript55900_1
Length=1269

 Score = 43.9 bits (102),  Expect = 0.004, Method: Compositional matrix adjust.
 Identities = 39/114 (34%), Positives = 51/114 (45%), Gaps = 0/114 (0%)
 Frame = -1

Query  3986  SQPK*ALFLTQNRISWRQHSSTARRWGFPKMIIMARARETATLSRLLQRTNPKEKRASMR  3807
             S PK* +      +  R   S  R   FP +I +A AR +ATL+ L     P    AS
Sbjct  328   SWPK*QVLSLHKSM*HRHFLSLMRHAAFPIIISLALARVSATLNLLSSTRKPSLYLAS*S  387

Query  3806  RISSDERTVDISIMSRSCP*NSSTDPIFGMTTSVPMNILRSIFTCF**GVIIPI  3645
                   RTVD  +   S P N ST P      S  +N  R ++TC  *G +IP+
Sbjct  388   SRRQLARTVDTRMTLFSLPWNDSTVPTSTALYSFCVNQSRILYTCLR*GDMIPM  441


 Score = 35.8 bits (81),  Expect = 1.1, Method: Compositional matrix adjust.
 Identities = 66/219 (30%), Positives = 89/219 (41%), Gaps = 27/219 (12%)
 Frame = -1

Query  3257  SRKDMENPIFEAIRLVTIDCSWRGSPERTT*ERFFVSITMGTIVSGSVH*PASS------  3096
             SR D+ +P+F A R +    S  GSPE T+          MGT  +GS    ASS
Sbjct  554   SRSDIPSPLFAASREMHTGPSCLGSPESTSCPVGASRPLMGTSTAGSSACAASSMNRCVM  613
```

```
Query  3095  NST*VKWPAGNPNCKKFPKVVQVEMVMR*VPINLA*QRSFNPDSVTIILS-SQLPTAPTS  2919
             +S  + W   +P   +  KVV                R+    S+ I L  S+     T
Sbjct  614   DSLNLNWLESDPYLAECSKVVTT-------------IRNMERSSLCIDLKVSRFAPFDTG  660

Query  2918  LVAQ*LIISGGIKSFST*LYETRRMKSPWSKALLSM-SLFVTKSAELFDGVPTKIRAPGR  2742
                   I SGG+   S      RRM       LL   S+    SA + + V T  R  G
Sbjct  661   ------ISSGGMILHSRFSELARRMNLLILSCLLYFSSISAMWSAAMLESVHTSTRFFGF  714

Query  2741  FLTNWSSVSISVIVFPLPGGPNST*GMFSASPRSILKTA  2625
               T  +  S +VIV P+PGGPN+ *G+      +I  TA
Sbjct  715   IATICTIASTTVIVLPVPGGPNTM*GLLPDVLETICSTA  753


> ta_transcript31550_1
Length=295

 Score = 40.0 bits (92),  Expect = 0.033, Method: Compositional matrix adjust.
 Identities = 50/211 (24%), Positives = 96/211 (45%), Gaps = 32/211 (15%)
 Frame = +1

Query  2095  KTYMHRFRLLLHLEEIECFVNFRNYDRDRAHF---LRDGEFLTLQIENLAERRPSLVIGD  2265
             + Y+  F  LL  EE    ++   YD         L+  +   L + NL +   SL  G+
Sbjct  107   ENYVRFFHNLLWYEETTVNIDMMKYDMYNVSLQQNLQQADCYVLVVPNLDQL--SLKRGN  164

Query  2266  TL----RVINPWSDPDSQTTKSYEGIIHKVLFDRILLKFHSSFQEKYNGEDYRLEFYFSR  2433
              +    RV   W +  ++ T + + ++  +L  +   ++ +S   K++     +EF +SR
Sbjct  165   KVNVRPRVSEAWFE--ARITATDKDVVSLLLPYQQFRQYSNSHNMKFD-----VEFMWSR  217

Query  2434  YSFRKQHHAISKIVGVMGEDFLFPSKVTKRENPQLDVYMKDDDMYLYDSKLEWYNQSL--  2607
                 + H A++ +  +     +FP          LD  +    +  Y+ + +++N+ +
Sbjct  218   TCLERMHCAVAAMQHLSNRTRVFPV---------LDCSV----IAPYNIQ-KYFNKLIKE  263

Query  2608  NSIQKRAVFNILRGEAENIPYVLFGPPGSGK  2700
             N  Q+ AV +I+ G A   PY+L GPPG+GK
Sbjct  264   NPEQRSAVEHIVGGTAGAAPYLLHGPPGTGK  294
*************************************************************************************************
***********************************************************************
Query= gi|665394979|ref|NM_079764.3| Drosophila melanogaster abnormal spindle (asp), mRNA

Length=6338
                                                                      Score     E
Sequences producing significant alignments:                          (Bits)  Value

  ta_transcript82361_1                                                  495   2e-141

> ta_transcript82361_1
Length=2641

 Score =   495 bits (1274),  Expect = 2e-141, Method: Compositional matrix adjust.
 Identities = 278/705 (39%), Positives = 444/705 (63%), Gaps = 47/705 (7%)
 Frame = +1

Query  1984  FAATTTIDPFLASTMYLDEQAVDRHQADFKKWLNALVSIPADLDADLNNKIDVGKLFNEV  2163
             ++ ++T+DPFL+ + + DE AV + + +FK+WLN +++ PADL++++  KIDVGK + E
Sbjct  682   YSQSSTVDPFLSISYFYDEAAVHKFEEEFKRWLNYILTPPADLESNIEQKIDVGKAWLEN  741

Query  2164  RNKELVVAPTKEEQSMNYLTKYRLETLRKAAVELFFSEQMRLPCSKVAVYVNKQALRIRS  2343
             RNKE+ VAPTKE+    Y    RLE+LRK+A  L  S +M     K+ + + K+ + IRS
Sbjct  742   RNKEVPVAPTKEQVCSAYHNSRRLESLRKSARALLMSPEMAQVFQKLNLQIEKKLIAIRS  801

Query  2344  DRNLHLDVVMQRTILELLLCFNPLWLRLGLEVVFGEKIQMQSNRDIVGLSTFILNRLFRN  2523
             DRNLHLDV +Q+ I+ELLL +NPLWLR+GLE ++G  + ++SN DI GL+TFI+ R+F+N
Sbjct  802   DRNLHLDVGLQKIIMELLLSYNPLWLRIGLEAIYGTVLPLKSNSDIDGLTTFIIQRMFKN  861

Query  2524  KCEEQRYSKAYT---LTEEYAETIKKHSLQKIlflllflDQAKQKRIVKHNPCLFVKKSP  2694
                + ++SK+     L   Y E IKK +L+K   L+ FLDQAKQK+++ H+PCLF + +
Sbjct  862   PHLKNKHSKSSAPNMLLPAYMEAIKKFTLKKFFMLVFFLDQAKQKKLISHDPCLFCRNAV  921

Query  2695  HKETKDILLRFSSELLANIGDITRELRRLGYVLQHRQTFLDEFDYAFNNLAVDLRDGVRL  2874
              KE+++I++RF+ EL+A IGDIT+ LR +GYV+ H+Q++LDE+ YA  N+A+D+RDGVR+
Sbjct  922   CKESREIIIRFTRELIAGIGDITKHLRPIGYVVSHKQSYLDEYKYAVQNIALDIRDGVRI  981

Query  2875  TRVMEVILLRDDLTRQLRVPAISRLQRIFNVKLALGALGEANFQLGGDIAAQDIVDGHRE  3054
             T+VME+ILL++ L  QLR PAISRLQ+I NV++AL AL EANF + GDI AQDI DGHRE
Sbjct  982   TKVMEIILLKNGLLTQLRTPAISRLQKIHNVQVALNALKEANFSIVGDITAQDIADGHRE  1041

Query  3055  KTLSLLWQLIYKFRSPKFHAAATVLQKWWRRHWLHVVIQRRIRHKELMRRHRAATVIQAV  3234
```

```
             KTLSLLWQLI+  R+P F  AA V+Q WWR+ +  +V +R+   K   + + AA+VIQ
Sbjct  1042  KTLSLLWQLIHVLRAPLFEKAANVIQMWWRKKYEVIVEKRKEEEKLRQKLNTAASVIQCW  1101

Query  3235  FRGHQMRKYVKLFKTERTQAAIILQKFTRRYLAQKQLYQSYHSIITIQRWWRAQQLGRQH  3414
             +R  Q  + V+    +   A +++QK+ R +L + +L +    S+  I+ W+++
Sbjct  1102  WRRIQYNRLVEWQMKQIVTATVVIQKYCRMWLCRTRLMRQKRSVRKIEEWYKS-------  1154

Query  3415  RQRFVELREAAIFLQRIWrrlfakkllaaaetarlQRSQKQQAAASYIQMQWRSYQLGR  3594
                 + +R A + L+ +                 R QR + +Q +A+ +Q    SY
Sbjct  1155  ---VIMMRNAKVTLEAL-----------------RKQREELRQRSATTLQ----SYVRRW  1190

Query  3595  IQRQQFLRQRDLIMFVQRRMRSKWSMLEQRKEFQQLKRAAINIQQRWRAKLSMRKCNADY  3774
             +  +++  +   I+ +Q  +R        RK + Q K++   IQ+ ++ KL MR+    +
Sbjct  1191  LCMKEYSWKVRKIVLIQSLVR----CFLVRKHYIQYKKSVTYIQRLYKGKLVMREEMKKF  1246

Query  3775  LALRSSVLKVQAYRKATIQMRIDRNHYYSLRKNVICLQQRLRAIMKMREQRENYLRLRNA  3954
                R++ + +Q+Y K    M   R  +  L+  V  +++R  A++KMR + + YLRL+ +
Sbjct  1247  AEARNAAITIQSYYK----MFKQRKQFDELKNAVKTIEERYIALLKMRNEMKQYLRLKQS  1302

Query  3955  SILVQKRYRMRQQMIQDRNAYLRTRKCIINVQRRW-RATLQMRRE  4086
             ++ +Q ++R     I+ R  YL  R  I+ +Q++  R+++   RE
Sbjct  1303  TVRIQSQWR----GIECRKEYLNKRNMIVKLQKKSPRSSVNEERE  1343


 Score = 62.8 bits (151),  Expect = 1e-08, Method: Compositional matrix adjust.
 Identities = 56/210 (27%), Positives = 108/210 (51%), Gaps = 21/210 (10%)
 Frame = +1

Query  4192  KVTLVVQKRRRALLQMRKERQEYLHLREVTIK-LQRRFHAQKSMRFMRAKYRGTQAAVSC  4368
             K  ++++  +  L  +RK+R+E       T++   RR+   K   +   K    Q+ V C
Sbjct  1153  KSVIMMRNAKVTLEALRKQREELRQRSATTLQSYVRRWLCMKEYSWKVRKIVLIQSLVRC  1212

Query  4369  LQMHWRNHLLRKRERNSFLQLRQAAITLQRRYRARLNMIKQLKSYAQLKQAAITIQTRYR  4548
                     L+RK     ++Q +++   +QR Y+ +L M +++K +A+ + AAITIQ+ Y+
Sbjct  1213  F-------LVRKH----YIQYKKSVTYIQRLYKGKLVMREEMKKFAEARNAAITIQSYYK  1261

Query  4549  AKKAMQKQVVLYQKQREAIIKVQRRYRGNLEMRKQIEVYQKQRQAVIRLQKWWRSIRDMR  4728
                 M KQ   + + + A+  ++ RY   L+MR +++ Y + +Q+ +R+Q  WR I
Sbjct  1262  ----MFKQRKQFDELKNAVKTIEERYIALLKMRNEMKQYLRLKQSTVRIQSQWRGIE---  1314

Query  4729  LCKAGYrrirlsslsiQRKW-RATVQARRQ  4815
              C+  Y   R   + +Q+K  R++V   R+
Sbjct  1315  -CRKEYLNKRNMIVKLQKKSPRSSVNEERE  1343


 Score = 60.8 bits (146),  Expect = 4e-08, Method: Compositional matrix adjust.
 Identities = 69/267 (26%), Positives = 117/267 (44%), Gaps = 51/267 (19%)
 Frame = +1

Query  3532  QKQQAAASYIQMQWRSYQLGRIQRQQFLRQRDLIMFVQRRMRSKWSMLEQRKEFQQLKRA  3711
             QK   AAS IQ  WR  Q  R+                     +W M       +Q+  A
Sbjct  1089  QKLNTAASVIQCWWRRIQYNRL--------------------VEWQM-------KQIVTA  1121

Query  3712  AINIQQRWRAKLSMRKCNADYLALRSSVLKVQAYRKATIQMRIDRNHYYSLRK-------  3870
              + IQ+  R    M  C    +  + SV K++ + K+ I MR  +    +LRK
Sbjct  1122  TVVIQKYCR----MWLCRTRLMRQKRSVRKIEEWYKSVIMMRNAKVTLEALRKQREELRQ  1177

Query  3871  -NVICLQQRLRAIMKMREQRENYLRLRNASILVQKRYRMRQQMIQDRNAYLRTRKCIINV  4047
              +   LQ  +R  + M+E      ++    +L+Q   R        R  Y++ +K +  +
Sbjct  1178  RSATTLQSYVRRWLCMKEYSWKVRKI----VLIQSLVRC----FLVRKHYIQYKKSVTYI  1229

Query  4048  QRRWRATLQMRRERKNYLHLQTTTKRIQIKFRAKREMKKQRAEFLQLKKVTLVVQKRRRA  4227
             QR ++  L MR E K +   +     IQ  ++    M KQR +F +LK     +++R  A
Sbjct  1230  QRLYKGKLVMREEMKKFAEARNAAITIQSYYK----MFKQRKQFDELKNAVKTIEERYIA  1285

Query  4228  LLQMRKERQEYLHLREVTIKLQRRFHA  4308
             LL+MR E ++YL L++ T+++Q ++
Sbjct  1286  LLKMRNEMKQYLRLKQSTVRIQSQWRG  1312


 Score = 55.1 bits (131),  Expect = 2e-06, Method: Compositional matrix adjust.
 Identities = 40/145 (28%), Positives = 72/145 (50%), Gaps = 11/145 (8%)
 Frame = +1

Query  202  FEITVTPSRLKQKKRAE-------GREPAVVVMAPFSAKAIVQFEDVPITKTARRQVRVL  360
            FEI  TP +++  +R E         E   +++APFS    V FE+V +  T  R++ V
Sbjct  53   FEIENTPEHIRRARRTEVVPKKSPKEECPRLILAPFSRPPQVVFENVLLGTTCERELEVF  112
```

```
Query  361   NPSDDDIEVKVMKAIREEHNLSL--EWMEHTVPARDEVSMELVWSPVLEVACKETLQLID  534
             NPS     ++ + KA+      + L  EW+    +        + ++W+P     A +E+++  +
Sbjct  113   NPSKQVQQITLGKALPPGLIIQLPGEWL--VLEPETCYCLTMMWTPTQPTALRESIRFTN  170

Query  535   NRNFRKEVMIILKSKSNQPVKNPRK  609
              +  R +V+++LKS   N   KN  K
Sbjct  171   EQRGRYDVIVVLKSTVNLKGKNQAK  195


 Score = 50.4 bits (119),  Expect = 7e-05, Method: Compositional matrix adjust.
 Identities = 86/369 (23%), Positives = 155/369 (42%), Gaps = 87/369 (24%)
 Frame = +1

Query  4618  RRYRGNLEMRKQIE-VYQKQRQAVIRLQKWWRSIRDMRLCKAGYrrirlsslsiQ---RK  4785
             ++Y    +E RK+ E + QK    A    +Q WWR I+   RL +    ++I   +++ IQ    R
Sbjct  1072  KKYEVIVEKRKEEEKLRQKLNTAASVIQCWWRRIQYNRLVEWQMKQIVTATVVIQKYCRM  1131

Query  4786  WRATVQARRQREIFLSTIRKVRLMQAFIRATLLMRQQRREFE--------MKRRAAVVIQ  4941
             W     +   RQ+        R VR ++ + ++ ++MR   +    E         +++R+A  +Q
Sbjct  1132  WLCRTRLMRQK-------RSVRKIEEWYKSVIMMRNAKVTLEALRKQREELRQRSATTLQ  1184

Query  4942  RRFRARCAMLKARQDYQLIQSSVILVQRKFRANRSMKQARQEFVQLRTIAVHLQQKFRGK  5121
                R     M     ++Y       ++L+Q     R          R+ ++Q +     ++Q+ ++GK
Sbjct  1185  SYVRRWLCM----KEYSWKVRKIVLIQSLVRC----FLVRKHYIQYKKSVTYIQRLYKGK  1236

Query  5122  RLMIEQRNCFQLLRCSMPGFQARARGFMARKRFQAL----------------MTPEMMDL  5253
               +M E+     F     R +       Q+    + F     RK+F   L                   M   EM
Sbjct  1237  LVMREEMKKFAEARNAAITIQSYYKMFKQRKQFDELKNAVKTIEERYIALLKMRNEMKQY  1296

Query  5254  IRQKRAAKVIQRYWRGYLIRRRQKHQGLLDIRKRIAQLRQEA--KAVNSVRCKVQEAVRF  5427
             +R K++     IQ    WRG     R+          L+ R     I  +L++++     +VN   R
Sbjct  1297  LRLKQSTVRIQSQWRGIECRKE-----YLNKRNMIVKLQKKSPRSSVNEER---------  1342

Query  5428  LRGRFIASDA----LAVLSRL------DRLSRTVPHLLMWC--SEFMS------------  5535
               GRF  A +A     +A + ++        D+ S    P   + +C   + F S
Sbjct  1343  -EGRFPAFEAVYYHIAAICKVISSNEEDKTSLHTPGPVCYCHTASFPS*AIYENATSIVH  1401

Query  5536  ---TFCYGI  5553
                  FCYG+
Sbjct  1402  STEDFCYGL  1410
```

****
****
Query= gi|562758882|gb|GABY01018532.1| TSA: Anthonomus grandis A_grandis_454_rep_c7409 mRNA sequence

Length=901

```
                                                                      Score     E
Sequences producing significant alignments:                          (Bits)  Value

  ta_transcript29552_1                                                32.3    1.6
  ta_transcript39759_1                                                30.8    4.3
  ta_transcript39756_1                                                30.8    5.1
  ta_transcript39757_1                                                30.8    5.2
  ta_transcript39758_1                                                30.8    5.4
```

> ta_transcript29552_1
Length=722

```
 Score = 32.3 bits (72),  Expect = 1.6, Method: Compositional matrix adjust.
 Identities = 20/83 (24%), Positives = 33/83 (40%), Gaps = 0/83 (0%)
 Frame = +1

Query  208   DQGYADPCSPSFLSKQNHDLRLQIQQSNNPPEEEMLMTVDQIVSYTDFDSPDISGRDCIR  387
              + Y +P S            +RL     S++ P E M+       +         S  ++ R
Sbjct  470   SRWYTEPSSRPITPAPITSMRLGTDFSDSAPVESMICESSNVTPGNGVTSDPVAMRMFFV  529

Query  388   VPLRGPFNPLETKLFGCTIRGCP  456
             + +    PF+P   + L G TI  CP
Sbjct  530   LMVSTPFSPCTSTLLGATIEPCP  552
```

> ta_transcript39759_1
Length=585

```
 Score = 30.8 bits (68),  Expect = 4.3, Method: Compositional matrix adjust.
 Identities = 27/116 (23%), Positives = 50/116 (43%), Gaps = 3/116 (3%)
 Frame = +1

Query  64   PENINRLFKQWTDNVVLYIEIYSVVNEVVELELYRSVQKDRSINRYL--LDQGYADPCSP  237
            P + + L + W      Y+  +   ++ +++ L++++ +  S +RY+  L      PC P
Sbjct  144  PSSNSSLDRPWPFRSSRYMSAWRSSSKALKIGLHKNITESISSSRYMGPLSSITLVPCDP  203

Query  238  SFLSKQNHDLRLQIQQSNNPPEEEMLMTVDQIVSYTDFDSPDISGRDCIRVPLRGP  405
              LS  +      I  S+N     M  T+  + +   F    IS   C R+ L GP
Sbjct  204  RLLSLLSTLAGHLISSSSN-NIVCMHTTLSHVKAILGFLRRRISDTCCFRIRLIGP  258


> ta_transcript39756_1
Length=742

 Score = 30.8 bits (68),  Expect = 5.1, Method: Compositional matrix adjust.
 Identities = 27/116 (23%), Positives = 50/116 (43%), Gaps = 3/116 (3%)
 Frame = +1

Query  64   PENINRLFKQWTDNVVLYIEIYSVVNEVVELELYRSVQKDRSINRYL--LDQGYADPCSP  237
            P + + L + W      Y+  +   ++ +++ L++++ +  S +RY+  L      PC P
Sbjct  157  PSSNSSLDRPWPFRSSRYMSAWRSSSKALKIGLHKNITESISSSRYMGPLSSITLVPCDP  216

Query  238  SFLSKQNHDLRLQIQQSNNPPEEEMLMTVDQIVSYTDFDSPDISGRDCIRVPLRGP  405
              LS  +      I  S+N     M  T+  + +   F    IS   C R+ L GP
Sbjct  217  RLLSLLSTLAGHLISSSSN-NIVCMHTTLSHVKAILGFLRRRISDTCCFRIRLIGP  271


> ta_transcript39757_1
Length=729

 Score = 30.8 bits (68),  Expect = 5.2, Method: Compositional matrix adjust.
 Identities = 27/116 (23%), Positives = 50/116 (43%), Gaps = 3/116 (3%)
 Frame = +1

Query  64   PENINRLFKQWTDNVVLYIEIYSVVNEVVELELYRSVQKDRSINRYL--LDQGYADPCSP  237
            P + + L + W      Y+  +   ++ +++ L++++ +  S +RY+  L      PC P
Sbjct  144  PSSNSSLDRPWPFRSSRYMSAWRSSSKALKIGLHKNITESISSSRYMGPLSSITLVPCDP  203

Query  238  SFLSKQNHDLRLQIQQSNNPPEEEMLMTVDQIVSYTDFDSPDISGRDCIRVPLRGP  405
              LS  +      I  S+N     M  T+  + +   F    IS   C R+ L GP
Sbjct  204  RLLSLLSTLAGHLISSSSN-NIVCMHTTLSHVKAILGFLRRRISDTCCFRIRLIGP  258


> ta_transcript39758_1
Length=598

 Score = 30.8 bits (68),  Expect = 5.4, Method: Compositional matrix adjust.
 Identities = 27/116 (23%), Positives = 50/116 (43%), Gaps = 3/116 (3%)
 Frame = +1

Query  64   PENINRLFKQWTDNVVLYIEIYSVVNEVVELELYRSVQKDRSINRYL--LDQGYADPCSP  237
            P + + L + W      Y+  +   ++ +++ L++++ +  S +RY+  L      PC P
Sbjct  157  PSSNSSLDRPWPFRSSRYMSAWRSSSKALKIGLHKNITESISSSRYMGPLSSITLVPCDP  216

Query  238  SFLSKQNHDLRLQIQQSNNPPEEEMLMTVDQIVSYTDFDSPDISGRDCIRVPLRGP  405
              LS  +      I  S+N     M  T+  + +   F    IS   C R+ L GP
Sbjct  217  RLLSLLSTLAGHLISSSSN-NIVCMHTTLSHVKAILGFLRRRISDTCCFRIRLIGP  271


************************************************************************************************
***********************************************************************

Query= gi|665393316|ref|NM_169121.2| Drosophila melanogaster Rm62 (Rm62),transcript variant F,
mRNA

Length=2833
                                                                      Score     E
Sequences producing significant alignments:                          (Bits)  Value

  ta_transcript16727_1                                                  597   0.0
  ta_transcript71371_1                                                  190   7e-50
  ta_transcript47090_1                                                  189   1e-49
  ta_transcript35263_1                                                  190   1e-49
  ta_transcript35262_1                                                  190   1e-49
  ta_transcript47089_1                                                  188   2e-49
```

```
  ta_transcript65883_1                                               185   3e-48
  ta_transcript35264_1                                               146   4e-36
  ta_transcript16729_1                                               133   5e-34
  ta_transcript20008_1                                               136   9e-33
  ta_transcript20007_1                                               135   2e-32

> ta_transcript16727_1
Length=587

 Score =   597 bits (1539),  Expect = 0.0, Method: Compositional matrix adjust.
 Identities = 284/431 (66%), Positives = 342/431 (79%), Gaps = 3/431 (1%)
 Frame = +2

Query  644   DFSNLAPFKKNFYQEHPNVANRSPYEVQRYREEQEITVRGQ-VPNPIQDFSEVHLPDYVM  820
             D + L PFKK FY  HP+V NRS  EV+ +R E +IT++G+ +P P   F E   PDYVM
Sbjct  116   DLNRLKPFKKEFYVPHPDVENRSESEVEAWRSENDITLKGRNIPKPTLTFDEAGFPDYVM  175

Query  821   KEIRRQGYKAPTAIQAQGWPIAMSGSNFVGIAKTGSGKTLGYILPAIVHINNQQPLQRGD  1000
              EI + G+  PT IQAQGWPIA+SG + VGIA TGSGKTL YILPAIVHIN+Q    RGD
Sbjct  176   DEIDKMGFSKPTPIQAQGWPIALSGHDMVGIASTGSGKTLSYILPAIVHINHQPKSSRGD  235

Query  1001  GPIALVLAPTRELAQQIQQVATEFGSSSYVRNTCVFGGAPKGGQMRDLQRGCEIVIATPG  1180
             GPIALVLAPTRELAQQIQ+V  +F ++S + NTC+FGGAPKG Q RDL  G EIVIATPG
Sbjct  236   GPIALVLAPTRELAQQIQEVCDKFANTSKIHNTCLFGGAPKGPQARDLDAGVEIVIATPG  295

Query  1181  RLIDFLSAGSTNLKRCTYLVLDEADRMLDMGFEPQIRKIVSQIRPDRQTLMWSATWPKEV  1360
             RL+DFL +G TNLKR TYLVLDEADRMLDMGFEPQIRKI+ QIRPDRQTLMWSATWP+EV
Sbjct  296   RLLDFLESGRTNLKRTTYLVLDEADRMLDMGFEPQIRKIIEQIRPDRQTLMWSATWPREV  355

Query  1361  KQLAEDFLGNYIQINIGSLELSANHNIRQVVDVCDEFSKEEKLKTLLSDIYDTSESPGKI  1540
             + LA +FL +Y+QIN+GSL+L+ANHNI Q++DVC E+ KE KL TLL +I   +E   K
Sbjct  356   QSLASEFLKDYLQINVGSLQLAANHNILQIIDVCMEYEKETKLSTLLKEIM--AEKENKT  413

Query  1541  IIFVETKRRVDNLVRFIRSFGVRCGAIHGDKSQSERDFVLREFRSGKSNILVATDVAARG  1720
             IIF+ETKRRVD++ R ++  G     IHGDKSQ+ERD+VL++FR+GK+ ILVATDVAARG
Sbjct  414   IIFIETKRRVDDITRKMKRDGWPAVCIHGDKSQNERDWVLQDFRTGKAPILVATDVAARG  473

Query  1721  LDVDGIKYVINFDYPQNSEDYIHRIGRTGRSNTKGTSFAFFTKNNAKQAKALVDVLREAN  1900
             LDVD +K+VINFDYP NSEDY+HRIGRTGR+N  GT++ FFT +NA +A  LV VL+EA
Sbjct  474   LDVDDVKFVINFDYPSNSEDYVHRIGRTGRTNKTGTAYTFFTPSNAAKAGDLVAVLKEAK  533

Query  1901  QEINPALENLA  1933
             Q +NP L+ LA
Sbjct  534   QVVNPKLQELA  544


> ta_transcript71371_1
Length=680

 Score =   190 bits (482),  Expect = 7e-50, Method: Compositional matrix adjust.
 Identities = 121/408 (30%), Positives = 207/408 (51%), Gaps = 25/408 (6%)
 Frame = +2

Query  719   EVQRYREEQEITVRG-QVPNPIQDFSEV----HLPDYVMKEIRRQGYKAPTAIQAQGWPI  883
             E  R+R E  I   G  VP  + DFS++     +   ++  + + GY  PT +Q Q  P
Sbjct  149   EQNRFRNEHGIKAVGRHVPAALTDFSDLVSRYKVSTSLVDTVTQCGYTEPTPVQRQALPC  208

Query  884   AMSGSNFVGIAKTGSGKTLGYILPAIVHINNQQPLQRGDGPIALVLAPTRELAQQIQQVA  1063
              +     +  A TGSGKT  ++LP +  +   Q      GP  LVL PTRELA QI + A
Sbjct  209   MLEDRQILACAPTGSGKTAAFLLPMLHTLGAPQ-----GGPRGLVLCPTRELAHQIYREA  263

Query  1064  TEFGSSSYVRNTCVFGGAPKGGQMRDLQ-RGCEIVIATPGRLIDFLSAGST--NLKRCTY  1234
                 + + +R + +        + R+   R  +I+I TP RL   L+  +   +L +  +
Sbjct  264   IRLSAGTELRVSVLRTVKESKVKEREATIRKSDILICTPNRLCYLLNQDNVGISLDKVRW  323

Query  1235  LVLDEADRMLD------MGFEPQIRKIVSQI-RPDRQTLMWSATWPKEVKQLAEDFLGNY  1393
             L++DEAD+M +        F  Q+ +I S      R+  M+SAT    V +     +
Sbjct  324   LIIDEADKMFEGSSEEQSAFRQQLEQIFSSCSHKQRRVAMFSATHTPVVAKWCRHHMRGL  383

Query  1394  IQINIGSLELSANHNIRQVVDVCDEFSKEEKLKTLLSDIYDTSESPGKIIIFVETKRRVD  1573
             I I +G    +A   + Q +  C   S+  KL      +    + P  +++FV++K R
Sbjct  384   INITVGQRN-AATQLVEQELLYCG--SEAGKLVAFRQLVQQGLQPP--VLVFVQSKERAK  438

Query  1574  NLVRFIRSFGVRCGAIHGDKSQSERDFVLREFRSGKSNILVATDVAARGLDVDGIKYVIN  1753
              L + +   G+   AIH D++Q++RD V+R FR G+  +L+ T++  RG+D  G+  V+N
Sbjct  439   QLFKELIYDGINVDAIHADRTQAQRDNVVRSFRVGRIWVLICTELMGRGIDFRGVNLVVN  498
```

```
Query  1754  FDYPQNSEDYIHRIGRTGRSNTKGTSFAFFTKNNAKQAKALVDVLREA  1897
             +D+P ++  YIHR+GR GR+  KG +  FFT+++    +++  V++++
Sbjct  499   YDFPPSAISYIHRVGRAGRAGQKGKAITFFTQDDVANLRSIASVMKQS  546


> ta_transcript47090_1
Length=645

 Score =  189 bits (479),  Expect = 1e-49, Method: Compositional matrix adjust.
 Identities = 110/338 (33%), Positives = 187/338 (55%), Gaps = 15/338 (4%)
 Frame = +2

Query  815   VMKEIRRQGYKAPTAIQAQGWPIAMSGSNFVGIAKTGSGKTLGYILPAIVHINNQQPLQR  994
             ++  ++  G + P+ IQ +  P  +   + VG AKTGSGKTL +++P + ++ N Q   +
Sbjct  126   ILAALKTMGIEKPSRIQIETLPHLLQQKDLVGAAKTGSGKTLAFLIPIVNNLINLQ-FTK  184

Query  995   GDGPIALVLAPTRELAQQIQQVATEFGSSSYVRNTCVFGGAPKGGQMRDLQRGCEIVIAT  1174
               G   ++L+PTRELA Q  +V  +  ++  +  + + GG  K  ++  L++GC IV++T
Sbjct  185   KHGTGCIILSPTRELALQTYEVLKKLLANIDLTCSLIVGGEKKAKDLKSLKKGCNIVVST  244

Query  1175  PGRLIDFL-SAGSTNLKRCTYLVLDEADRMLDMGFEPQIRKIVSQIRPDRQTLMWSATWP  1351
             PGRL+D L +    N      LV+DEAD++L+ GFE  +  I+  +  DRQT+++SAT
Sbjct  245   PGRLLDHLQNTEGFNCNNLKCLVVDEADKLLEAGFEKHVTGIIKLLPKDRQTVLFSATMD  304

Query  1352  KEVKQLAEDFL-GNYIQINIGSLELSANHNIRQVVDVCDEFSKEEKLKTLLSDIYDTSES  1528
              +VK LA+  L  N + I I     S    ++Q   +C     E+++  L   +  + +
Sbjct  305   DKVKNLAKLALRSNPVTIAIRDNVQSTVEGLQQGYYIC---PVEKRIAWLYKMLKKSRKL  361

Query  1529  PGKIIIFVETKRRVDNLVRFIRSFGVRCGA----IHGDKSQSERDFVLREFRSGKSNILV  1696
               K+++F  + + VD    F R   V C A    IHG ++Q  R      F + ++  L
Sbjct  362   --KVMVFFSSCKSVDFHYEFFR---VHCKANVTSIHGKQTQPRRKEAYHSFTNAENGALF  416

Query  1697  ATDVAARGLDVDGIKYVINFDYPQNSEDYIHRIGRTGR  1810
              TD+AARGLD+  + +++ +D P + ++YIHR+GRT R
Sbjct  417   CTDIAARGLDIPCVDWIVQYDPPTDPKEYIHRVGRTAR  454


> ta_transcript35263_1
Length=752

 Score =  190 bits (482),  Expect = 1e-49, Method: Compositional matrix adjust.
 Identities = 106/227 (47%), Positives = 150/227 (66%), Gaps = 9/227 (4%)
 Frame = +2

Query  1253  DRMLDMGFEPQIRKIV-SQIRP---DRQTLMWSATWPKEVKQLAEDFLGNYIQINIGSLE  1420
             +RMLDMGFEPQIRKIV     P   +RQTLM+SAT+PK+++ LA+DFL NY+ + +G +
Sbjct  1     ERMLDMGFEPQIRKIVECHTMPKTGERQTLMFSATFPKQIQVLAQDFLSNYVFLAVGRVG  60

Query  1421  LSANHNIRQVVDVCDEFSKEEKLKTLL--SDIYDTS--ESPGKIIIFVETKRRVDNLVRF  1588
              S + NI Q V   +E  K   L  LL  S++   S  E     ++FVETK+  D L  +
Sbjct  61    -STSENITQKVVWVEESQKRSFLLDLLNASNLLQRSRPEEDQLTLVFVETKKGADQLEEY  119

Query  1589  IRSFGVRCGAIHGDKSQSERDFVLREFRSGKSNILVATDVAARGLDVDGIKYVINFDYPQ  1768
             + + G    +IHGD++Q ER+  LR FR+G++ ILVAT VAARGLD+  +++VINFD P
Sbjct  120   LDTEGYPVTSIHGDRTQREREEALRRFRTGQTPILVATAVAARGLDIPHVRHVINFDLPS  179

Query  1769  NSEDYIHRIGRTGRSNTKGTSFAFFTKNNAKQAKALVDVLREANQEI  1909
             + E+Y+HRIGRTGR    G + +FF  NN   ++ LV++L EA Q++
Sbjct  180   DVEEYVHRIGRTGRMGNLGVATSFFNDNNRGLSRDLVELLVEAKQDV  226


> ta_transcript35262_1
Length=758

 Score =  190 bits (482),  Expect = 1e-49, Method: Compositional matrix adjust.
 Identities = 108/235 (46%), Positives = 152/235 (65%), Gaps = 9/235 (4%)
 Frame = +2

Query  1253  DRMLDMGFEPQIRKIV-SQIRP---DRQTLMWSATWPKEVKQLAEDFLGNYIQINIGSLE  1420
             +RMLDMGFEPQIRKIV     P   +RQTLM+SAT+PK+++ LA+DFL NY+ + +G +
Sbjct  1     ERMLDMGFEPQIRKIVECHTMPKTGERQTLMFSATFPKQIQVLAQDFLSNYVFLAVGRVG  60

Query  1421  LSANHNIRQVVDVCDEFSKEEKLKTLL--SDIYDTS--ESPGKIIIFVETKRRVDNLVRF  1588
              S + NI Q V   +E  K   L  LL  S++   S  E     ++FVETK+  D L  +
Sbjct  61    -STSENITQKVVWVEESQKRSFLLDLLNASNLLQRSRPEEDQLTLVFVETKKGADQLEEY  119

Query  1589  IRSFGVRCGAIHGDKSQSERDFVLREFRSGKSNILVATDVAARGLDVDGIKYVINFDYPQ  1768
```

```
             + + G    +IHGD++Q ER+  LR FR+G++ ILVAT VAARGLD+  +++VINFD P
Sbjct  120   LDTEGYPVTSIHGDRTQREREEALRRFRTGQTPILVATAVAARGLDIPHVRHVINFDLPS  179

Query  1769  NSEDYIHRIGRTGRSNTKGTSFAFFTKNNAKQAKALVDVLREANQEINPALENLA  1933
             + E+Y+HRIGRTGR    G + +FF  NN    ++ LV++L EA Q++   L   A
Sbjct  180   DVEEYVHRIGRTGRMGNLGVATSFFNDNNRGLSRDLVELLVEAKQDVPNWLTETA  234


> ta_transcript47089_1
Length=648

 Score =   188 bits (478),  Expect = 2e-49, Method: Compositional matrix adjust.
 Identities = 119/369 (32%), Positives = 193/369 (52%), Gaps = 20/369 (5%)
 Frame = +2

Query  815   VMKEIRRQGYKAPTAIQAQGWPIAMSGSNFVGIAKTGSGKTLGYILPAIVHINNQQPLQR  994
             ++  ++  G + P+ IQ +  P  +   + VG AKTGSGKTL +++P + ++ N Q   +
Sbjct  126   ILAALKTMGIEKPSRIQIETLPHLLQQKDLVGAAKTGSGKTLAFLIPIVNNLINLQ-FTK  184

Query  995   GDGPIALVLAPTRELAQQIQQVATEFGSSSYVRNTCVFGGAPKGGQMRDLQRGCEIVIAT  1174
               G   ++L+PTRELA Q  +V  +  ++  +  + + GG  K   ++ L++GC IV++T
Sbjct  185   KHGTGCIILSPTRELALQTYEVLKKLLANIDLTCSLIVGGEKKAKDLKSLKKGCNIVVST  244

Query  1175  PGRLIDFL-SAGSTNLKRCTYLVLDEADRMLDMGFEPQIRKIVSQIRPDRQTLMWSATWP  1351
             PGRL+D L +    N      LV+DEAD++L+ GFE  +  I+  +  DRQT+++SAT
Sbjct  245   PGRLLDHLQNTEGFNCNNLKCLVVDEADKLLEAGFEKHVTGIIKLLPKDRQTVLFSATMD  304

Query  1352  KEVKQLAEDFL-GNYIQINIGSLELSANHNIRQVVDVCDEFSKEEKLKTLLSDIYDTSES  1528
              +VK LA+  L  N + I I     S    ++Q   +C      EK    L  +   S
Sbjct  305   DKVKNLAKLALRSNPVTIAIRDNVQSTVEGLQQGYYICPV----EKRIAWLYKMLKKSRK  360

Query  1529  PGKIIIFVETKRRVDNLVRFIRSFGVRCGA----IHGDKSQSERDFVLREFRSGKSNILV  1696
               K+++F  + + VD    F R   V C A    IHG ++Q  R      F + ++  L
Sbjct  361   L-KVMVFFSSCKSVDFHYEFFR---VHCKANVTSIHGKQTQPRRKEAYHSFTNAENGALF  416

Query  1697  ATDVAARGLDVDGIKYVINFDYPQNSEDYIHRIGRTGRS-NTKGTSFAFFTKNNAKQAKA  1873
              TD+AARGLD+  + +++ +D P + ++YIHR+GRT R  N  G +          K
Sbjct  417   CTDIAARGLDIPCVDWIVQYDPPTDPKEYIHRVGRTARGLNNTGNAVILLRPEEDK----  472

Query  1874  LVDVLREAN  1900
              V+ LR  N
Sbjct  473   FVEFLRNEN  481


> ta_transcript65883_1
Length=668

 Score =   185 bits (469),  Expect = 3e-48, Method: Compositional matrix adjust.
 Identities = 111/344 (32%), Positives = 189/344 (55%), Gaps = 12/344 (3%)
 Frame = +2

Query  827   IRRQGYKAPTAIQAQGWPIAMSGSNFVGIAKTGSGKTLGYILPAIVHINNQQPLQRGDGP  1006
             I+  G+   T IQ++   P  + G + VG A+TGSGKTL +++PA+  I   +   R +G
Sbjct  165   IKDMGFTTMTEIQSKAIPPLLEGRDLVGAARTGSGKTLAFLIPAVELIYKLKFKPR-NGT  223

Query  1007  IALVLAPTRELAQQIQQVATEFGSSSYVRNTCVFGGAPKGGQMRDLQRGCEIVIATPGRL  1186
               ++L+PTREL+ Q   V  E      +      V GGA +  + + L +G  I++ATPGRL
Sbjct  224   GVIILSPTRELSMQTFGVLMELMKYHHHTYGLVMGGANRSTEAQKLSKGINILVATPGRL  283

Query  1187  IDFLSAGSTNL-KRCTYLVLDEADRMLDMGFEPQIRKIVSQIRPDRQTLMWSATWPKEVK  1363
             +D L       L K    LV+DEADR+L++GFE ++++I+   +    RQT+M+SAT  K+++
Sbjct  284   LDHLQNTPDFLYKNLQCLVIDEADRILEIGFEEEVKQIIKLLPKRRQTMMFSATQTKKIE  343

Query  1364  QLAEDFLGN---YIQINIGSLELSANHNIRQVVDVCDEFSKEEKLKTLLSDIYDTSESPG  1534
              L    + +   Y+ ++    E +    ++ Q   VC     E++   L + +    +
Sbjct  344   ALTALAVKHEPVYVGVD-DHREQATVDSLEQGYIVC---PSEKRFLVLFTFLKKNRKK--  397

Query  1535  KIIIFVETKRRVDNLVRFIRSFGVRCGAIHGDKSQSERDFVLREFRSGKSNILVATDVAA  1714
             K+++F+ T    V        +     +    +IHG + QS+R     +F + ++ IL+ TDVAA
Sbjct  398   KVMVFLSTCMSVKYHHELLNYIDLPVMSIHGKQQQSKRTTTFFQFCNAETGILLCTDVAA  457

Query  1715  RGLDVDGIKYVINFDYPQNSEDYIHRIGRTGRS-NTKGTSFAFF  1843
             RGLD+  + +++ +D P + ++YIHR+GRT R   T G +  F
Sbjct  458   RGLDIPAVDWIVQYDPPDDPKEYIHRVGRTARGLGTSGHALLFL  501


> ta_transcript35264_1
```

```
Length=511

 Score =   146 bits (368),  Expect = 4e-36, Method: Compositional matrix adjust.
 Identities = 75/178 (42%), Positives = 106/178 (60%), Gaps = 7/178 (4%)
 Frame = +2

Query  764   QVPNPIQDFSEVHLPDYVMKEIRRQGYKAPTAIQAQGWPIAMSGSNFVGIAKTGSGKTLG  943
             +VP+ I  F +V+L + + + I    Y  PT +Q    PI +    + +  A+TGSGKT
Sbjct  334   RVPDFITSFEDVNLTEIMRQNIASARYDKPTPVQKYAIPIVLGHRDVMACAQTGSGKTAA  393

Query  944   YILPAIVHINNQQPLQ-------RGDGPIALVLAPTRELAQQIQQVATEFGSSSYVRNTC  1102
             +++P +  +    P++       R   P+ LVLAPTRELA QI   A +F   S VR
Sbjct  394   FLVPILNQMYEAGPVKHMGPYNRRKQYPLGLVLAPTRELATQIFDEARKFAYRSRVRPCV  453

Query  1103  VFGGAPKGGQMRDLQRGCEIVIATPGRLIDFLSAGSTNLKRCTYLVLDEADRMLDMGF  1276
             V+GG+P   Q R+L+RGC +++ATPGRL+D L+ G   L  C +LVLDEADRMLDMG
Sbjct  454   VYGGSPIHEQFRELERGCHLLVATPGRLVDMLARGRVALDHCRHLVLDEADRMLDMGL  511


> ta_transcript16729_1
Length=198

 Score =   133 bits (334),  Expect = 5e-34, Method: Compositional matrix adjust.
 Identities = 61/89 (69%), Positives = 74/89 (83%), Gaps = 0/89 (0%)
 Frame = +2

Query  1667  FRSGKSNILVATDVAARGLDVDGIKYVINFDYPQNSEDYIHRIGRTGRSNTKGTSFAFFT  1846
             FR+GK+ ILVATDVAARGLDVD +K+VINFDYP NSEDY+HRIGRTGR+N  GT++ FFT
Sbjct  67    FRTGKAPILVATDVAARGLDVDDVKFVINFDYPSNSEDYVHRIGRTGRTNKTGTAYTFFT  126

Query  1847  KNNAKQAKALVDVLREANQEINPALENLA  1933
              +NA +A  LV VL+EA Q +NP L+ LA
Sbjct  127   PSNAAKAGDLVAVLKEAKQVVNPKLQELA  155


> ta_transcript20008_1
Length=567

 Score =   136 bits (343),  Expect = 9e-33, Method: Compositional matrix adjust.
 Identities = 73/200 (37%), Positives = 121/200 (61%), Gaps = 7/200 (4%)
 Frame = +2

Query  1328  LMWSATWPKEVKQLAEDFLGNYIQINIGSLELSANHNIRQVVDVCDEFSKEEKLKTLLSD  1507
             LM+SAT+P++++ LA  FL NY+ + +G +   A+ ++ Q+     +F K+  LK L+ +
Sbjct  1     LMFSATFPEDIQHLAGRFLHNYLFVAVGIVG-GASTDVEQIFLQVAKFDKQNALKQLIEE  59

Query  1508  IYDTSESPGKIIIFVETKRRVDNLVRFIRSFGVRCGAIHGDKSQSERDFVLREFRSGKSN  1687
                       +I++FVETKR  D +    +    +IHGD+ Q ER+  L  F++G
Sbjct  60    -----NEKKRILVFVETKRNADFIASILSEQQLLTSSIHGDRMQREREEALHNFKTGHHC  114

Query  1688  ILVATDVAARGLDVDGIKYVINFDYPQNSEDYIHRIGRTGRSNTKGTSFAFFTKNNAKQA  1867
             ILVAT VAARGLD+  +  V+N+D P++ ++Y+HRIGRTGR   +G + +FF  +  +
Sbjct  115   ILVATAVAARGLDIKNVDIVVNYDLPKSIDEYVHRIGRTGRVGNRGKAVSFFDDSQDQAL  174

Query  1868  KA-LVDVLREANQEINPALE  1924
              A L  +LR+A+Q +   L+
Sbjct  175   CADLAKILRQADQPVPDFLQ  194


> ta_transcript20007_1
Length=569

 Score =   135 bits (341),  Expect = 2e-32, Method: Compositional matrix adjust.
 Identities = 73/200 (37%), Positives = 121/200 (61%), Gaps = 7/200 (4%)
 Frame = +2

Query  1328  LMWSATWPKEVKQLAEDFLGNYIQINIGSLELSANHNIRQVVDVCDEFSKEEKLKTLLSD  1507
             LM+SAT+P++++ LA  FL NY+ + +G +   A+ ++ Q+     +F K+  LK L+ +
Sbjct  1     LMFSATFPEDIQHLAGRFLHNYLFVAVGIVG-GASTDVEQIFLQVAKFDKQNALKQLIEE  59

Query  1508  IYDTSESPGKIIIFVETKRRVDNLVRFIRSFGVRCGAIHGDKSQSERDFVLREFRSGKSN  1687
                       +I++FVETKR  D +    +    +IHGD+ Q ER+  L  F++G
Sbjct  60    -----NEKKRILVFVETKRNADFIASILSEQQLLTSSIHGDRMQREREEALHNFKTGHHC  114

Query  1688  ILVATDVAARGLDVDGIKYVINFDYPQNSEDYIHRIGRTGRSNTKGTSFAFFTKNNAKQA  1867
             ILVAT VAARGLD+  +  V+N+D P++ ++Y+HRIGRTGR   +G + +FF  +  +
Sbjct  115   ILVATAVAARGLDIKNVDIVVNYDLPKSIDEYVHRIGRTGRVGNRGKAVSFFDDSQDQAL  174
```

```
Query  1868  KA-LVDVLREANQEINPALE  1924
              A L  +LR+A+Q +   L+
Sbjct  175   CADLAKILRQADQPVPDFLQ  194
***************************************************************************************************
************************************************************************
Query= gi|392886220|ref|NM_059731.4| Caenorhabditis elegans Protein EGO-1 (ego-1) mRNA, complete
cds

Length=4899


***** No hits found *****
***************************************************************************************************
************************************************************************
Query= gi|392886216:1414-3303 Caenorhabditis elegans Protein RRF-1, isoform a

Length=1890


***** No hits found *****
***************************************************************************************************
************************************************************************

Query= gi|392887358:1372-3231 Caenorhabditis elegans Protein RRF-2 (rrf-2) mRNA, complete cds

Length=1860


***** No hits found *****

***************************************************************************************************
***********************************************************************


Query= gi|392890784:2059-3819 Caenorhabditis elegans Protein RRF-3 (rrf-3) mRNA, complete cds

Length=1761


***** No hits found *****

***************************************************************************************************
***********************************************************************
Query= gi|195438743|ref|XM_002067256.1| Drosophila willistoni GK16260 (Dwil\GK16260)(Rdrp), mRNA

Length=558
                                                                       Score     E
Sequences producing significant alignments:                           (Bits)  Value

  ta_transcript24699_1                                                   125   3e-34
  ta_transcript13924_1                                                   124   1e-32

> ta_transcript24699_1
Length=275

 Score =   125 bits (315),  Expect = 3e-34, Method: Compositional matrix adjust.
 Identities = 67/151 (44%), Positives = 92/151 (61%), Gaps = 12/151 (8%)
 Frame = +1

Query  85   LDNPTFCCYLFWSTVLVAKMLLMSLLTALQRFRYKLLGLIPLALRRKIFPNEEDLFFKNL  264
            + +P    ++   S VL   K+L M+ LTA QR            +++K+F NEED
Sbjct  32   VSDPLAQTFVVHSAVLALKVLGMAFLTARQR------------MKKKVFANEEDAVKSGA  79

Query  265  EVKFDDPHVERVRRAHRNDMENILPYFIMSLIYISTNPSQAIACNLFRVASVARILHTLV  444
            +VKFDDP VERVRRAH ND+ENI   ++I+   +Y++T PS   +A   L R   + +R+LHTLV
Sbjct  80   KVKFDDPDVERVRRAHLNDLENIPAFWIVGGLYLTTGPSPVVATWLIRAFTASRVLHTLV  139

Query  445  YAVYPVPQPSRILAFATMLCITFYMAAVVAL  537
            YAV P+PQP+R LAF      IT YM   V +
Sbjct  140  YAVKPMPQPARGLAFGIANFITIYMGVKVVM  170


> ta_transcript13924_1
Length=422

 Score =   124 bits (311),  Expect = 1e-32, Method: Compositional matrix adjust.
```

```
 Identities = 63/147 (43%), Positives = 92/147 (63%), Gaps = 13/147 (9%)
 Frame = +1

Query  85   LDNPTFCCYLFWSTVLVAKMLLMSLLTALQRFRYKLLGLIPLALRRKIFPNEEDLFFK-N  261
            LD+P    ++ +S +L  K+L+M+ +T + R            +R+ +F N ED
Sbjct  80   LDSPAVQSFILYSAILALKVLVMAPMTGMTR------------MRKGVFANPEDAKLTPK  127

Query  262  LEVKFDDPHVERVRRAHRNDMENILPYFIMSLIYISTNPSQAIACNLFRVASVARILHTL  441
             +VKFDDP +ER+RRAH ND+ENI   ++++   +Y++T P  A A  LFRV ++ RILHT+
Sbjct  128  GKVKFDDPDIERIRRAHLNDLENIPAFWVLGALYLTTGPVAAWATLLFRVYAIGRILHTI  187

Query  442  VYAVYPVPQPSRILAFATMLCITFYMA  522
            VY + P+PQPSR LAFA  L I FYM
Sbjct  188  VYCIVPLPQPSRALAFAIPLFIKFYMG  214


*************************************************************************************************
**********************************************************************
```

Query= gi|665402677|ref|NM_166503.3| Drosophila melanogaster snipper (Snp),transcript variant G, mRNA

Length=1667

```
***** No hits found *****
*************************************************************************************************
***********************************************************************
```

Query= gi|392898168|ref|NM_171245.4| Caenorhabditis elegans Protein ERI-1, isoform a (eri-1) mRNA, complete cds

Length=1347

```
***** No hits found *****
*************************************************************************************************
***********************************************************************
```

Query= gi|392898456|ref|NM_067798.5| Caenorhabditis elegans Protein ERI-5 (eri-5) mRNA, complete cds

Length=1596

```
                                                                      Score     E
Sequences producing significant alignments:                          (Bits)  Value

  ta_transcript16301_1                                                30.8    8.5
  ta_transcript64721_1                                                31.2    8.9
  ta_transcript34177_1                                                31.2    8.9
  ta_transcript64718_1                                                31.2    9.0
  ta_transcript64722_1                                                31.2    9.4
  ta_transcript64724_1                                                31.2    9.6
  ta_transcript64720_1                                                31.2    9.8
  ta_transcript64716_1                                                31.2    9.8
  ta_transcript64723_1                                                31.2    9.9
  ta_transcript64717_1                                                31.2    9.9


> ta_transcript16301_1
Length=242

 Score = 30.8 bits (68),  Expect = 8.5, Method: Compositional matrix adjust.
 Identities = 22/71 (31%), Positives = 32/71 (45%), Gaps = 7/71 (10%)
 Frame = -1

Query  669  DHKISGKLRDHNILHRNRANL-LDRHFFIDFCS------IWQSVQVHEKRRAVRAKIHVT  511
            D +I G LR  N   R R     D     D C+       +  SV +HE+ R  R+ I +
Sbjct  124  DSEILGSLRPQNTSTRVRFFFAFDARCARDLCTHLLSG*LQDSVSIHEQMRLYRSDI*LN  183

Query  510  LVGGNLKFCKF  478
            +VG   + FC +
Sbjct  184  VVGLAIHFCTY  194


> ta_transcript64721_1
Length=1104

 Score = 31.2 bits (69),  Expect = 8.9, Method: Compositional matrix adjust.
 Identities = 13/31 (42%), Positives = 20/31 (65%), Gaps = 0/31 (0%)
```

```
 Frame = -3

Query  1033  SRNRRFFADFPSFLRAARKSNVHYQTSFPVP  941
             S+N + F D PS LR +R +   YQ+S+ +P
Sbjct  513   SKNMQVFCDIPSQLRGSRTTRGIYQSSWVMP  543


> ta_transcript34177_1
Length=729

 Score = 31.2 bits (69),  Expect = 8.9, Method: Compositional matrix adjust.
 Identities = 15/41 (37%), Positives = 22/41 (54%), Gaps = 0/41 (0%)
 Frame = +1

Query  940   LAPEKMSDNEHWIFEQLVKNSENQQKIDDFYSNLKNQRPLE  1062
             LAP    DN+H +F ++V+  E  Q I    +N K  +P E
Sbjct  662   LAPTPWLDNKHTVFGRVVRGMEVVQNIGTVKTNPKTDKPYE  702


> ta_transcript64718_1
Length=1109

 Score = 31.2 bits (69),  Expect = 9.0, Method: Compositional matrix adjust.
 Identities = 13/31 (42%), Positives = 20/31 (65%), Gaps = 0/31 (0%)
 Frame = -3

Query  1033  SRNRRFFADFPSFLRAARKSNVHYQTSFPVP  941
             S+N + F D PS LR +R +   YQ+S+ +P
Sbjct  518   SKNMQVFCDIPSQLRGSRTTRGIYQSSWVMP  548


> ta_transcript64722_1
Length=1102

 Score = 31.2 bits (69),  Expect = 9.4, Method: Compositional matrix adjust.
 Identities = 13/31 (42%), Positives = 20/31 (65%), Gaps = 0/31 (0%)
 Frame = -3

Query  1033  SRNRRFFADFPSFLRAARKSNVHYQTSFPVP  941
             S+N + F D PS LR +R +   YQ+S+ +P
Sbjct  518   SKNMQVFCDIPSQLRGSRTTRGIYQSSWVMP  548


> ta_transcript64724_1
Length=1097

 Score = 31.2 bits (69),  Expect = 9.6, Method: Compositional matrix adjust.
 Identities = 13/31 (42%), Positives = 20/31 (65%), Gaps = 0/31 (0%)
 Frame = -3

Query  1033  SRNRRFFADFPSFLRAARKSNVHYQTSFPVP  941
             S+N + F D PS LR +R +   YQ+S+ +P
Sbjct  513   SKNMQVFCDIPSQLRGSRTTRGIYQSSWVMP  543


> ta_transcript64720_1
Length=1106

 Score = 31.2 bits (69),  Expect = 9.8, Method: Compositional matrix adjust.
 Identities = 13/31 (42%), Positives = 20/31 (65%), Gaps = 0/31 (0%)
 Frame = -3

Query  1033  SRNRRFFADFPSFLRAARKSNVHYQTSFPVP  941
             S+N + F D PS LR +R +   YQ+S+ +P
Sbjct  518   SKNMQVFCDIPSQLRGSRTTRGIYQSSWVMP  548


> ta_transcript64716_1
Length=1120

 Score = 31.2 bits (69),  Expect = 9.8, Method: Compositional matrix adjust.
 Identities = 13/31 (42%), Positives = 20/31 (65%), Gaps = 0/31 (0%)
 Frame = -3

Query  1033  SRNRRFFADFPSFLRAARKSNVHYQTSFPVP  941
             S+N + F D PS LR +R +   YQ+S+ +P
Sbjct  513   SKNMQVFCDIPSQLRGSRTTRGIYQSSWVMP  543
```

```
> ta_transcript64723_1
Length=1101

 Score = 31.2 bits (69),  Expect = 9.9, Method: Compositional matrix adjust.
 Identities = 13/31 (42%), Positives = 20/31 (65%), Gaps = 0/31 (0%)
 Frame = -3

Query  1033  SRNRRFFADFPSFLRAARKSNVHYQTSFPVP  941
             S+N + F D PS LR +R +   YQ+S+ +P
Sbjct  513   SKNMQVFCDIPSQLRGSRTTRGIYQSSWVMP  543


> ta_transcript64717_1
Length=1113

 Score = 31.2 bits (69),  Expect = 9.9, Method: Compositional matrix adjust.
 Identities = 13/31 (42%), Positives = 20/31 (65%), Gaps = 0/31 (0%)
 Frame = -3

Query  1033  SRNRRFFADFPSFLRAARKSNVHYQTSFPVP  941
             S+N + F D PS LR +R +   YQ+S+ +P
Sbjct  518   SKNMQVFCDIPSQLRGSRTTRGIYQSSWVMP  548


***************************************************************************************************
***********************************************************************
Query= gi|392901601|ref|NM_001268821.1| Caenorhabditis elegans Protein RSD-2, isoform c (rsd-2)
mRNA, complete cds

Length=1254
                                                                      Score     E
Sequences producing significant alignments:                          (Bits)  Value

  ta_transcript74877_1                                                35.0    0.33
  ta_transcript74875_1                                                35.0    0.33


> ta_transcript74877_1
Length=624

 Score = 35.0 bits (79),  Expect = 0.33, Method: Compositional matrix adjust.
 Identities = 20/58 (34%), Positives = 30/58 (52%), Gaps = 8/58 (14%)
 Frame = +1

Query  172  SYIFMYRPIEVHKATHYAPFEITQVVEILNTENHGRVDRSEFSSLFTFDLCNVDQRYV  345
            S+ FMYRP+ + K   ++P E+T  +  LN          E    ++  D+ NVD RYV
Sbjct  153  SFFFMYRPVFLWKVV*FSPVELTLKIYALN--------*IE*DLIYVDDIANVDSRYV  202


> ta_transcript74875_1
Length=635

 Score = 35.0 bits (79),  Expect = 0.33, Method: Compositional matrix adjust.
 Identities = 20/58 (34%), Positives = 30/58 (52%), Gaps = 8/58 (14%)
 Frame = +1

Query  172  SYIFMYRPIEVHKATHYAPFEITQVVEILNTENHGRVDRSEFSSLFTFDLCNVDQRYV  345
            S+ FMYRP+ + K   ++P E+T  +  LN          E    ++  D+ NVD RYV
Sbjct  153  SFFFMYRPVFLWKVV*FSPVELTLKIYALN--------*IE*DLIYVDDIANVDSRYV  202


***************************************************************************************************
***********************************************************************
Query= gi|392927450|ref|NM_077572.5| Caenorhabditis elegans Protein RSD-3(rsd-3) mRNA, complete
cds

Length=1452


***** No hits found *****
***************************************************************************************************
***********************************************************************
Query= gi|392886390|ref|NM_059868.6| Caenorhabditis elegans Protein RSD-6 (rsd-6) mRNA, complete
cds
```

```
Length=2070
                                                                      Score     E
Sequences producing significant alignments:                          (Bits)  Value

  ta_transcript20037_1                                                30.4    8.0


> ta_transcript20037_1
Length=153

 Score = 30.4 bits (67),  Expect = 8.0, Method: Compositional matrix adjust.
 Identities = 18/37 (49%), Positives = 22/37 (59%), Gaps = 0/37 (0%)
 Frame = +2

Query  1511  QMHLSSVELFDIKMFKSLQSVLRSI*NHMKR*IKRFK  1621
             QM L  VE+F  +  K     LRSI* H+KR*+K F
Sbjct  17    QMALCYVEIFTFES*KHFTF*LRSI*VHLKR*VKNFP  53
************************************************************************************************
***********************************************************************
Query= gi|304307738|ref|NM_001195080.1| Bombyx mori tudor staphylococcus/micrococcal nuclease
(Tudor-SN), mRNA

Length=2667
                                                                      Score     E
Sequences producing significant alignments:                          (Bits)  Value

  ta_transcript15886_1                                                  246   3e-99


> ta_transcript15886_1
Length=953

 Score =   246 bits (628),  Expect(2) = 3e-99, Method: Compositional matrix adjust.
 Identities = 174/501 (35%), Positives = 265/501 (53%), Gaps = 2/501 (0%)
 Frame = -1

Query  2667  LATHPEFYRIVLGDVTVPPQVESRALGVLLSGAILGHQCRQSAVTNLLH*HEPIFDKVLA  2488
             LA   E  R+VLGDV V PQVE RALGVLL  A+L H+ RQ  V + LH H+  FD+VLA
Sbjct  15    LAARAELSRVVLGDVAVLPQVEPRALGVLLRRAVLAHERRQPVVAHTLHQHQTFFDQVLA  74

Query  2487  QINVCVRINKGNSSW*STDFYIQEQPLANDIIIETGYSFCSVIRVRGEFHTAENILCRKR  2308
              +     + + ++   +   ++Q+Q L   +  E  +   +V+ VRGE H A+ +L R
Sbjct  75    DVEAGGGLEERDAGRGAAHLHVQQQLLVERVDGERAHRLGAVVGVRGELHAAQRVLARVA  134

Query  2307  RIVLSTHRECCQSGYV*RLSISIVDVYNLDLPIVCYFFNLCPVPLVIGCKTSSTDGSLPW  2128
              + L   R+  Q      L++S+VDV  L   ++ +F +L PVPLV+  + S  +G+
Sbjct  135   GLALRAGRQRRQPRAAHHLTVSVVDVEYLGGFVIGHFLDLGPVPLVVEGEASGAEGASSR  194

Query  2127  SIRSRQWVTVSKLSVYSFHKTF*FHT*IDILCVEMTFRSDFSDDHAVVFKSTILYSCISL  1948
              + + Q     +L VY  H+       +D L  E         DH ++F   +L+  ++
Sbjct  195   HV*AGQRRVSLELLVYLLHEALELGAEVDALREERAVGRHLRHDHLLIFHHAVLHGRVAF  254

Query  1947  LfdfffffDIVFPYADTLFNCILFSGFYGACKLRRFRRVMRRGKAMFD*RNGQILVVNPE  1768
             LF  FF    V PY D   + +L  G   A +LRR   V+  G+ +   R+GQ+LVV P+
Sbjct  255   LFRLFFHLHKVLPYTDAFLDRVLLGGLDEARELRRLGGVVHAGEPVLHQRHGQVLVVRPQ  314

Query  1767  PTDEVPCFVNLFYAD*DVVL*ALFFRELQSFFSERLCILF-GSSANCRSRTSGTVNAGKQ  1591
             P DEV   V+L      VVL AL  RELQ  F+ERL +L   ++A+ R+R +   +AG+Q
Sbjct  315   PADEVARLVHLLDGHVHVVLQALLLRELQRLFAERLSVLHAAAAADRRARAARAADAGQQ  374

Query  1590  KGY*DGFFRNI*THTRSASNEFHSSLGFLGTfeeweeffcFRWISTGVLHTVGGHFFLVV  1411
             +        ++  H R A +E H  L  L   +E E+ F    +S GVLH V G   L V
Sbjct  375   ERDHHRLLGDVDAHARPARHELHGRLRALRPLQEREKLFRLGGVSAGVLHAVRGDVLLGV  434

Query  1410  NTNCGLNCFQFGLQ*Fiil*aslviivsiLHCCQ-PFSHQSFSYVRATAYDSTTCLFWEI  1234
             + +  L   + GLQ  +++ A LV++V +   C+  F+H  FSYV + +Y+   CLFW +
Sbjct  435   HADGRLLRLELGLQQLVVVRAPLVVVVPVPDDCRVTFAH*RFSYVNSLSYNCGACLFWVV  494

Query  1233  IFCWLNVIYCHINFFPNQFFS  1171
             +   L+V+  H++FF  Q FS
Sbjct  495   VLRGLDVVDSHVHFFCQQAFS  515


 Score =   137 bits (346),  Expect(2) = 3e-99, Method: Compositional matrix adjust.
 Identities = 173/405 (43%), Positives = 190/405 (47%), Gaps = 10/405 (2%)
 Frame = -3
```

```
Query  1192  FSQPVFF*EIHVPRTSMEYHTKV*ILLASV*VYCRRQHCSFHEEA*R*LRIFFSVHC-YV  1016
             F Q  F * I  PRTS E     *IL A V        CSF   A*   R   S  C +
Sbjct  509   FCQQAFS*GIPSPRTSRECRIAA*IL*ALVTFSPPHLGCSFRAAA*-CWRGISS*RCRWA  567

Query  1015  F*Qrarlr*lLPTRWP*IPCLSPGL*VHCLHNLSICAVL------LP*LHFQRLSM*THL  854
              *+RAR R*    R P* PCL P L   C  + S  A         P      L+ *  +
Sbjct  568   A*RRARRR*PPRARSP*TPCLWPRLWARCSRSSSRYAAFDLWRRARPHAASTHLTS*PRV  627

Query  853   IS*HISRTLVLVGPQRCYPAGAVSCPR-YCCY*RTPELLRRLSAAIWTPGRSVLLLRKAQ  677
              S HISRTL    P+RC P GA   PR YCC  R    LR LS    TPGR  LL RK
Sbjct  628   TSRHISRTLAAAEPRRCCPEGAGWYPRSYCCS-RILGSLRCLSEVAATPGRIGLLPRKVL  686

Query  676   IHRLVLQRDIEFQIASV*WECIQVTNKDVL*NHHAHIQ**PLQAYHCIC*RKLSDFR*TT  497
             IHRLV Q DI F  AS *    Q T K  L +H   IQ**   A HCIC R L   +*
Sbjct  687   IHRLVSQLDI*FLRASK*SGRNQATGKV*LSSHL*RIQ**L*SAVHCICSRMLLCSQ*CP  746

Query  496   *CLVHVYQHQYSKFLCLAIWLSLRSLPAF*AEVYF*HLLEL*RDLQPARLQ*HFHLWKDF  317
             *CL         K+LCL I    +        V F     L*+D QP  LQ*H   W
Sbjct  747   *CL*CDPEDLVPKYLCLEILPDPQFQLISLVGVCFCLPPSL*QDPQPEGLQ*HSLTW*GL  806

Query  316   CQPIRRHILYSLNSPVSQQ*R*PPCRLASSLGIHGLPTRMVHPSFLCHCWRSFGERV*VR  137
             C+P   H  +  +  VSQQ* *P C    S     LP +MV   F CHC +S G  V* R
Sbjct  807   CRPTPGHTPWLQS*VVSQQ*I*PLCLQVFSSDTPLLPKQMVRLLFQCHC*QSSGALV*AR  866

Query  136   *YPKGLSLSLEAARPEAVFLLPPYLPIVPASLFLFCKQVPVLEHS  2
             * P  LS SL A R  A  LLP      PA LF FCKQ  VLEHS
Sbjct  867   *CPTALSPSLVAGRLRAASLLPLCPLTTPALLFRFCKQGLVLEHS  911


************************************************************************************************
**********************************************************************
Query= gi|512926474|ref|XM_004931038.1| PREDICTED: Bombyx mori probable exonuclease mut-7
homolog (LOC101745346), mRNA

Length=1521
                                                                      Score     E
Sequences producing significant alignments:                          (Bits)  Value

  ta_transcript44087_1                                                52.0    3e-06


> ta_transcript44087_1
Length=1591

 Score = 52.0 bits (123),  Expect = 3e-06, Method: Compositional matrix adjust.
 Identities = 45/89 (51%), Positives = 57/89 (64%), Gaps = 0/89 (0%)
 Frame = +2

Query  1238  IVNGNHHLEQYNHKWP*SKLLLSIMFT**IH*F*TNKNIPVFGTLSINHLWKTVRLSN*D  1417
             IV+GN  LE ++ +  * K L  IM+T**I  F*T++N+ VFG  S NH  K ++   *
Sbjct  463   IVSGNLLLEPHSPRLH*YK*LHLIMYT**IPSF*TDRNM*VFGIHSTNHS*KMLK**R*A  522

Query  1418  LGLNRI*KK*KHL*MVWEK*MSKVKDY*I  1504
             L  N+I K+*K L* V E *  KVKDY*I
Sbjct  523   LAWNKILKR*KVL*QV*ET*K*KVKDY*I  551
```
