# Supplementary material for: De novo transcriptome assembly and analysis to identify potential gene targets for RNAi-mediated control of the tomato leafminer (Tuta absoluta)
Source: BMC Genomics. 2015 Aug 26;16(1):635. doi: 10.1186/s12864-015-1841-5 (PMC4550053; doi:10.1186/s12864-015-1841-5)
Supplement: Additional file 9: Table S8. — List of genes associated with biosynthesis of the two major hormone classes sesquiterpenoid juvenile (JH) and the ecdysteroid hormones identified in the T. absoluta assembled transcriptome at E-value < e−30 using homologues particularly from Bombyx mori. FPKM values for each transcript normalized per library is presented. Red rows represent undetected genes within the T. absoluta transcriptome for all sequenced stages, while grey rows are those that did not reach the set E-value < e−30. (PDF 118 kb) [file 12864_2015_1841_MOESM9_ESM.pdf]

Table S8. List of genes associated with biosynthesis of the two major hormone classes sesquiterpenoid juvenile (JH) and the ecdysteroid hormones identified in the *T. absoluta* assembled transcriptome at E-value < e^{-30} using homologues particularly from *Bombyx mori*. FPKM values for each transcript normalized per library is presented. Red rows represent undetected genes within the *T. absoluta* transcriptome for all sequenced stages, while grey rows are those that did not reach the set *E*-value < e^{-30}.

GENE	BAIT	TRANSCRIPTS	*E* VALUE	FPKM values normalized per library					
				eggs	1st stage	2nd stage	3rd stage	4th stage	adult
**Juvenile Hormone**									
Fps	gi|112983457:82-1365 *Bombyx mori* farnesyl pyrophosphate syntase (*Fps*)	ta_transcript80181	$3e^{-107}$	31,70226	41.17608	50.39921	39.20374	40.35811	31.31506
		ta_transcript80180	$2e^{-106}$	31.34419	22.87304	51.20281	46.33539	33.40154	12.47268
		ta_transcript80179	$1e^{-104}$	30.99913	28.94011	36.13398	25.01813	22.24278	19.45934
		ta_transcript80177	$2e^{-102}$	27.68555	37.91512	61.72762	46.04650	27.51597	32.57899
		ta_transcript80178	$2e^{-102}$	41.60657	57.04494	60.91355	55.45847	47.27998	46.47978
		ta_transcript39822	$1e^{-64}$	340.88604	284.09696	460.72136	511.31608	903.15349	1029.03219
		ta_transcript39820	$1e^{-64}$	513.47219	413.31366	732.69244	662.95399	1265.73330	1196.40182
		ta_transcript39821	$1e^{-62}$	258.68528	277.06268	402.14552	429.23462	837.16220	921.41593
		ta_transcript39819	$1e^{-62}$	417.39295	344.87277	715.96227	618.35945	1138.22696	1085.21163
Fpps2	gi|153791941:133-1281 *Bombyx mori* farnesyl diphosphate synthase 2 (*Fpps2*)	ta_transcript80181	$7e^{-119}$	31.70226	41.17608	50.39921	39.20374	40.35811	31.31506
		ta_transcript80180	$5e^{-118}$	31.34419	22.87304	51.20281	46.33539	33.40154	12.47268
		ta_transcript80179	$3e^{-116}$	30.99913	28.94011	36.13398	25.01813	22.24278	19.45934
		ta_transcript80178	$3e^{-114}$	41.60657	57.04494	60.91355	55.45847	47.27998	46.47978
		ta_transcript80177	$6e^{-114}$	27.68555	37.91512	61.72762	46.04650	27.51597	32.57899
Fpps3	gi|153791943:253-1410 *Bombyx mori* farnesyl diphosphate synthase 3 (*Fpps3*)	ta_transcript80181	$2e^{-152}$	31.70226	41.17608	50.39921	39.20374	40.35811	31.31506
		ta_transcript80180	$8e^{-152}$	31.34419	22.87304	51.20281	46.33539	33.40154	12.47268
		ta_transcript80179	$3e^{-150}$	30.99913	28.94011	36.13398	25.01813	22.24278	19.45934
		ta_transcript80178	$7e^{-148}$	41.60657	57.04494	60.91355	55.45847	47.27998	46.47978
		ta_transcript80177	$1e^{-147}$	27.68555	37.91512	61.72762	46.04650	27.51597	32.57899
Jhamt	gi|112982769:125-961 *Bombyx mori* juvenile hormone acid methyltransferase (*Jhamt*)	ta_transcript79838	$8e^{-109}$	0.0	10.14826	21.24237	25.00294	43.47058	118.58244
		ta_transcript79836	$1e^{-108}$	0.0	10.80452	28.83549	29.28181	35.02685	131.80595
		ta_transcript79837	$1e^{-108}$	0.0	5.47468	24.06519	22.12086	38.37446	132.54924
		ta_transcript79835	3e-^{108}	0.0	3.65212	26.21014	17.99593	53.02716	147.77743
P450	gi|226502343:35-1516 *Bombyx mori* cytochrome P450 (P450)	ta_transcript44033	$3e^{-94}$	16.65508	18.24978	36.69260	39.28365	58.06936	62.40334
		ta_transcript62517	$5e^{-73}$	10.51646	111.48048	211.81898	110.33630	58.69009	89.89964
		ta_transcript44034	$5e^{-47}$	7.42903	13.22574	19.37892	47.57431	83.43464	37.70846
		ta_transcript44035	$2e^{-37}$	9.81369	25.57134	22.93973	46.80136	78.41794	28.17270
Jheh-	gi|261245094:276-1658 *Bombyx*	ta_transcript53251	$1e^{-131}$	63.705321	5601.734328	4210.468942	9682.604283	7651.23707	8604.39549

lp1	*mori* juvenile hormone epoxide hydrolase-like protein 1 (Jheh-lp1)	ta_transcript53250	$2e^{-131}$	54.783956	5354.732496	4059.502700	9134.424151	7393.98405	8323.31674
		ta_transcript29071	$4e^{-100}$	0.640198	3126.650261	4556.335845	6016.708801	5709.85188	40.43270
		ta_transcript45224	$3e^{-97}$	6.679206	5861.220719	20973.245495	52889.366936	38048.65653	86.81723
		ta_transcript58145	$2e^{-75}$	4.513249	213.445520	181.808633	217.636929	199.16087	40.59681
		ta_transcript58144	$2e^{-74}$	20.401938	239.013202	191.054381	234.786841	193.83102	346.35215
		ta_transcript35275	$2e^{-34}$	136.324304	82.748089	105.197339	166.322703	144.18537	105.75941
Jheh2	gi|112984537:97-1482 *Bombyx mori* juvenile hormone epoxide hydrolase (Jheh2)	ta_transcript53250	0.0	54.783956	5354.732496	4059.502700	9134.424151	7393.98405	8323.31674
		ta_transcript53251	0.0	63.705321	5601.734328	4210.468942	9682.604283	7651.23707	8604.39549
		ta_transcript29071	$6e^{-154}$	0.640198	3126.650261	4556.335845	6016.708801	5709.85188	40.432704
		ta_transcript45224	$3e^{-148}$	6.679206	5861.220719	20973.245495	52889.366936	38048,65653	86.817226
		ta_transcript58145	$4e^{-129}$	4.513249	213.445520	181.808633	217.636929	199.16087	40.596808
		ta_transcript58144	$1e^{-128}$	20.401938	239.013202	191.054381	234.786841	193.83102	346.35215
		ta_transcript35275	$2e^{-56}$	136.324304	82.748089	105.197339	166.322703	144.18537	105.75942
		ta_transcript53254	$2e^{-31}$	772.150251	2120.798704	1861.582413	4017.091763	7762.13081	2847.28772
Jheh-lp2	gi|261245096:150-1556 *Bombyx mori* juvenile hormone epoxide hydrolase-like protein 2 (Jheh-lp2)	ta_transcript53250	$9e^{-178}$	54.78396	5354.732496	4059.502700	9134.424151	7393.98405	8323.31674
		ta_transcript53251	$2e^{-177}$	63.70532	5601.734328	4210.468942	9682.604283	7651.23707	8604.39549
		ta_transcript29071	$1e^{-145}$	0.64020	3126.650261	4556.335845	6016.708801	5709.85188	40.432704
		ta_transcript45224	$3e^{-134}$	6.67921	5861.220719	20973.245495	52889.366936	38048,65653	86.817226
		ta_transcript58145	$1e^{-103}$	4.51325	213.445520	181.808633	217.636929	199.16087	40.596808
		ta_transcript58144	$9e^{-103}$	20.40194	239.013202	191.054381	234.786841	193.83102	346.35215
		ta_transcript35275	$1e^{-33}$	136.32430	82.748089	105.197339	166.322703	144.18537	105.75942
Jheh-lp3	gi|261245098:126-2039 Bombyx mori juvenile hormone epoxide hydrolase-like protein 3 (Jheh-lp3),	ta_transcript53250	$7e^{-152}$	54.78396	5354.732496	4059.502700	9134.424151	7393.98405	8323.31674
		ta_transcript53251	$8e^{-152}$	63.70532	5601.734328	4210.468942	9682.604283	7651.23707	8604.39549
		ta_transcript35275	$1e^{-132}$	136.32430	82.748089	105.197339	166.322703	144.18537	105.75942
		ta_transcript29071	$8e^{-118}$	0.640202	3126.650261	4556.335845	6016.708801	5709.85188	40.432704
		ta_transcript45224	$8e^{-109}$	6.67921	5861.220719	20973.245495	52889.366936	38048,65653	86.817226
		ta_transcript58145	$3e^{-100}$	4.51325	213.445520	181.808633	217.636929	199.16087	40.596808
		ta_transcript58144	$2e^{-99}$	20.40194	239.013202	191.054381	234.786841	193.83102	346.35215
		ta_transcript59199	$9e^{-31}$	214.56009	1572.94780	1239.26372	1706.78796	2003.73428	1554.05773
		ta_transcript59195	$1e^{-30}$	216.94659	1266.85734	1028.63250	1315.57847	1669.92879	40.43270422
Jheh-lp4	gi|261245100:118-1602 *Bombyx mori* juvenile hormone epoxide hydrolase-like protein 4 (Jheh-lp4)	ta_transcript53250	$4e^{-126}$	54.78396	5354.732496	4059.502700	9134.424151	7393.98405	8323.31674
		ta_transcript53251	$6e^{-126}$	63.70532	5601.734328	4210.468942	9682.604283	7651.23707	8604.39549
		ta_transcript29071	$6e^{-110}$	0.640202	3126.650261	4556.335845	6016.708801	5709.85188	40.432704
		ta_transcript45224	$1e^{-102}$	6.67921	5861.220719	20973.245495	52889.366936	38048,65653	86.817226
		ta_transcript58145	$4e^{-83}$	4.51325	213.445520	181.808633	217.636929	199.16087	40.596808
		ta_transcript58144	$2e^{-82}$	20.40194	239.013202	191.054381	234.786841	193.83102	346.35215
		ta_transcript35275	$3e^{-34}$	136.32430	82.748089	105.197339	166.322703	144.18537	105.75942

jheh-lp5	gi|295424203:63-1451 *Bombyx mori* juvenile hormone epoxide hydrolase-like protein 5 (jheh-lp5)	ta_transcript53251	0.0	63.70532	5601.734328	4210.468942	9682.604283	7651.23707	8604.39549
		ta_transcript53250	0.0	54.78396	5354.732496	4059.502700	9134.424151	7393.98405	8323.31674
		ta_transcript29071	$2e^{-142}$	0.640202	3126.650261	4556.335845	6016.708801	5709.85188	40.432704
		ta_transcript45224	$2e^{-139}$	6.67921	5861.220719	20973.245495	52889.366936	38048,65653	86.817226
		ta_transcript58145	$3e^{-108}$	4.51325	213.445520	181.808633	217.636929	199.16087	40.596808
		ta_transcript58144	$2e^{-107}$	20.40194	239.013202	191.054381	234.786841	193.83102	346.35215
		ta_transcript35275	$9e^{-54}$	136.32430	82.748089	105.197339	166.322703	144.18537	105.75942
jhe1	gi|112983177:33-1745 *Bombyx mori* juvenile hormone esterase 1 (jhe1)	ta_transcript82619	$7e^{-65}$	5.18845	15.72119	30.68416	4145514	33282422	2859796
		ta_transcript82614	$2e^{-64}$	11.31262	27.08608	33.68933	50,67899	383,84424	46.23666
		ta_transcript82616	$1e^{-62}$	3.67698	65.37427	35.66178	56,.22702	424.91120	51.11178
		ta_transcript82623	$6e^{-57}$	7.68942	23.98889	15.69177	30.29031	269.30098	28.03104
		ta_transcript58915	$7e^{-57}$	37.34863	887.77646	1276.91744	1483.85579	1109.38866	246.49795
		ta_transcript58914	$1e^{-56}$	11.31262	27.08608	33.68933	50.67899	383.84424	46.23666
		ta_transcript75783	$8e^{-51}$	34.50366	407.57438	266.05792	520.28971	610.79065	125.98657
		ta_transcript48580	$3e^{-50}$	8.37505	5.71547	17.51818	13.67925	19.31574	51.52006
		ta_transcript48579	$4e^{-50}$	13.41914	21.82808	35.84351	15.20656	15.82788	87.60035
		ta_transcript48393	$4e^{-37}$	33.94650	266.24385	639.13316	753.41294	386.85609	106.05076
		ta_transcript84821	$7e^{-37}$	15.25286	1166.56966	1903.77027	1769.00719	1486.28483	350.81894
		ta_transcript84818	$2e^{-36}$	14.97503	1045.64713	1483.80138	1517.12646	1283.70485	303.53292
		ta_transcript84816	$1e^{-35}$	19.83621	874.03672	1185.21216	1222.27996	961.04666	218.70870
		ta_transcript29751	$2e^{-35}$	3.28967	320.58640	1753.05488	1025.47777	1431.60358	577.96267
Jhe-like	gi|512902947|ref|XM_004925442.1 | PREDICTED: *Bombyx mori* juvenile hormone esterase-like (LOC101744318)	ta_transcript58915	$4e^{-173}$	37.34863	887.77646	1276.91744	1483.85579	1109.38866	246.49795
		ta_transcript58914	$4e^{-172}$	11.31262	27.08608	33.68933	50.67899	383.84424	46.23666
		ta_transcript75783	$1e^{-127}$	34.50366	407.57438	266.05792	520.28971	610.79065	125.98657
		ta_transcript48580	$5e^{-86}$	8.37505	5.71547	17.51818	13.67925	19.31574	51.52006
		ta_transcript48579	$2e^{-85}$	13.41914	21.82808	35.84351	15.20656	15.82788	87.60035
		ta_transcript84821	$4e^{-54}$	15.25286	1166.56966	1903.77027	1769.00719	1486.28483	350.81894
		ta_transcript84818	$1e^{-53}$	14.97503	1045.64713	1483.80138	1517.12646	1283.70485	303.53292
		ta_transcript84816	$5e^{-53}$	19.83621	874.03672	1185.21216	1222.27996	961.04666	218.70870
		ta_transcript29751	$7e^{-50}$	3.28967	320.58640	1753.05488	1025.47777	1431.60358	577.96267
		ta_transcript48393	$6e^{-47}$	33.94650	266.24385	639.13316	753.41294	386.85609	106.05076
		ta_transcript49309	$4e^{-41}$	0.50122	168.80300	71.95660	18.20314	5.39460	64.26996
		ta_transcript30390	$9e^{-33}$	119.52487	191.94988	417.92985	1027.02579	1355.41858	145.67227
Jhe-like	gi|512903791:16-1749 PREDICTED: *Bombyx mori* juvenile hormone esterase-like (LOC101743778)	ta_transcript48580	$2e^{-73}$	8.37505	5.71547	17.51818	13.67925	19.31574	51.52006
		ta_transcript48579	$5e^{-73}$	13.41914	21.82808	35.84351	15.20656	15.82788	87.60035
		ta_transcript75783	$4e^{-68}$	34.50366	407.57438	266.05792	520.28971	610.79065	125.98657
		ta_transcript58915	$2e^{-67}$	37.34863	887.77646	1276.91744	1483.85579	1109.38866	246.49795
		ta_transcript58914	$3e^{-67}$	11.31262	27.08608	33.68933	50.67899	383.84424	46.23666
		ta_transcript84818	$1e^{-45}$	14.97503	1045.64713	1483.80138	1517.12646	1283.70485	303.53292
		ta_transcript84821	$2e^{-45}$	15.25286	1166.56966	1903.77027	1769.00719	1486.28483	350.81894
		ta_transcript84816	$4e^{-45}$	19.83621	874.03672	1185.21216	1222.27996	961.04666	218.70870

		ta_transcript48393	$8e^{-45}$	33.94650	266.24385	639.13316	753.41294	386.85609	106.05076
		ta_transcript29751	$6e^{-44}$	3.28967	320.58640	1753.05488	1025.47777	1431.60358	577.96267
		ta_transcript82619	$5e^{-41}$	5.18845	15.72119	30.68416	4145514	33282422	2859796
		ta_transcript82614	$9e^{-41}$	11.31262	27.08608	33.68933	50,67899	383,84424	46.23666
		ta_transcript82616	$9e^{-41}$	3.67698	65.37427	35.66178	56,.22702	424.91120	51.11178
		ta_transcript82623	$8e^{-34}$	7.68942	23.98889	15.69177	30.29031	269.30098	28.03104
		ta_transcript49309	$8e^{-30}$	0.50122	168.80300	71.95660	18.20314	5.39460	64.26996
Jhe-like	gi|512885949|ref|XM_004921624.1 | PREDICTED: *Bombyx mori* juvenile hormone esterase-like (LOC101739057)	ta_transcript48580	$5e^{-68}$	8.37505	5.71547	17.51818	13.67925	19.31574	51.52006
		ta_transcript48579	$1e^{-67}$	13.41914	21.82808	35.84351	15.20656	15.82788	87.60035
		ta_transcript75783	$1e^{-59}$	34.50366	407.57438	266.05792	520.28971	610.79065	125.98657
		ta_transcript58915	$4e^{-57}$	37.34863	887.77646	1276.91744	1483.85579	1109.38866	246.49795
		ta_transcript58914	$8e^{-57}$	11.31262	27.08608	33.68933	50.67899	383.84424	46.23666
		ta_transcript48393	$3e^{-54}$	33.94650	266.24385	639.13316	753.41294	386.85609	106.05076
		ta_transcript29751	$7e^{-49}$	3.28967	320.58640	1753.05488	1025.47777	1431.60358	577.96267
		ta_transcript84821	$1e^{-40}$	15.25286	1166.56966	1903.77027	1769.00719	1486.28483	350.81894
		ta_transcript84818	$3e^{-40}$	14.97503	1045.64713	1483.80138	1517.12646	1283.70485	303.53292
		ta_transcript84816	$2e^{-39}$	19.83621	874.03672	1185.21216	1222.27996	961.04666	218.70870
		ta_transcript15565	$6e^{-30}$	1.56952	34.54302	43.71965	17.41703	2.81544	18.02279
Jhe-like	gi|530233691|ref|NM_001281891.1 | *Bombyx mori* juvenile hormone esterase-like (LOC101743505)	ta_transcript58915	$8e^{-58}$	37.34863	887.77646	1276.91744	1483.85579	1109.38866	246.49795
		ta_transcript58914	$2e^{-57}$	11.31262	27.08608	33.68933	50.67899	383.84424	46.23666
		ta_transcript48393	$9e^{-55}$	33.94650	266.24385	639.13316	753.41294	386.85609	106.05076
		ta_transcript29751	$3e^{-54}$	3.28967	320.58640	1753.05488	1025.47777	1431.60358	577.96267
		ta_transcript75783	$1e^{-53}$	34.50366	407.57438	266.05792	520.28971	610.79065	125.98657
		ta_transcript48580	$4e^{-50}$	8.37505	5.71547	17.51818	13.67925	19.31574	51.52006
		ta_transcript48579	$1e^{-49}$	13.41914	21.82808	35.84351	15.20656	15.82788	87.60035
		ta_transcript84821	$2e^{-42}$	15.25286	1166.56966	1903.77027	1769.00719	1486.28483	350.81894
		ta_transcript84818	$3e^{-42}$	14.97503	1045.64713	1483.80138	1517.12646	1283.70485	303.53292
		ta_transcript84816	$1e^{-41}$	19.83621	874.03672	1185.21216	1222.27996	961.04666	218.70870
Jhe-like	gi|512897630:468-1769 PREDICTED: *Bombyx mori* juvenile hormone esterase-like (LOC101737052)	ta_transcript58915	$9e^{-54}$	37.34863	887.77646	1276.91744	1483.85579	1109.38866	246.49795
		ta_transcript58914	$1e^{-53}$	11.31262	27.08608	33.68933	50.67899	383.84424	46.23666
		ta_transcript75783	$4e^{-52}$	34.50366	407.57438	266.05792	520.28971	610.79065	125.98657
		ta_transcript48580	$2e^{-42}$	8.37505	5.71547	17.51818	13.67925	19.31574	51.52006
		ta_transcript48579	$3e^{-42}$	13.41914	21.82808	35.84351	15.20656	15.82788	87.60035
		ta_transcript29751	$2e^{-41}$	3.28967	320.58640	1753.05488	1025.47777	1431.60358	577.96267
		ta_transcript48393	$4e^{-39}$	33.94650	266.24385	639.13316	753.41294	386.85609	106.05076
		ta_transcript84821	$8e^{-38}$	15.25286	1166.56966	1903.77027	1769.00719	1486.28483	350.81894
		ta_transcript84818	$2e^{-37}$	14.97503	1045.64713	1483.80138	1517.12646	1283.70485	303.53292
		ta_transcript84816	$7e^{-37}$	19.83621	874.03672	1185.21216	1222.27996	961.04666	218.70870
		ta_transcript15565	$3e^{-34}$	1.56952	34.54302	43.71965	17.41703	2.81544	18.02279
Jhe-like	gi|512897844:207-1901 PREDICTED: *Bombyx mori*	ta_transcript58915	$5e^{-82}$	37.34863	887.77646	1276.91744	1483.85579	1109.38866	246.49795
		ta_transcript58914	$3e^{-81}$	11.31262	27.08608	33.68933	50.67899	383.84424	46.23666

var 2	juvenile hormone esterase-like (LOC101746607), transcript variant X2	ta_transcript75783	$1e^{-76}$	34.50366	407.57438	266.05792	520.28971	610.79065	125.98657
		ta_transcript74072	$3e^{-71}$	13.114726	125.90493	230.12694	239.22083	320.45542	33.23979
		ta_transcript74071	$1e^{-70}$	42.771857	183.77299	268.08750	430.61214	814.74793	171.23424
		ta_transcript84821	$8e^{-61}$	15.25286	1166.56966	1903.77027	1769.00719	1486.28483	350.81894
		ta_transcript20178	$8e^{-61}$	0.000	1.10921	12.76991	9.29163	257.54508	1.55533
		ta_transcript29751	$2e^{-60}$	3.28967	320.58640	1753.05488	1025.47777	1431.60358	577.96267
		ta_transcript84818	$9e^{-60}$	14.97503	1045.64713	1483.80138	1517.12646	1283.70485	303.53292
		ta_transcript84816	$4e^{-59}$	19.83621	874.03672	1185.21216	1222.27996	961.04666	218.70870
		ta_transcript48580	$4e^{-58}$	8.37505	5.71547	17.51818	13.67925	19.31574	51.52006
		ta_transcript48579	$4e^{-58}$	13.41914	21.82808	35.84351	15.20656	15.82788	87.60035
		ta_transcript48393	$4e^{-54}$	33.94650	266.24385	639.13316	753.41294	386.85609	106.05076
		ta_transcript15565	$6e^{-42}$	1.56952	34.54302	43.71965	17.41703	2.81544	18.02279
		ta_transcript49309	$3e^{-32}$	0.501220	168.80300	71.95660	18.20314	5.39460	64.26996
Jhe-like	gi|512885543:14-1618 PREDICTED: *Bombyx mori* juvenile hormone esterase-like (LOC101741415)	ta_transcript48580	$1e^{-62}$	8.37505	5.71547	17.51818	13.67925	19.31574	51.52006
		ta_transcript48579	$2e^{-62}$	13.41914	21.82808	35.84351	15.20656	15.82788	87.60035
		ta_transcript75783	$3e^{-57}$	34.50366	407.57438	266.05792	520.28971	610.79065	125.98657
		ta_transcript58915	$3e^{-56}$	37.34863	887.77646	1276.91744	1483.85579	1109.38866	246.49795
		ta_transcript58914	$7e^{-56}$	11.31262	27.08608	33.68933	50.67899	383.84424	46.23666
		ta_transcript48393	$1e^{-47}$	33.94650	266.24385	639.13316	753.41294	386.85609	106.05076
		ta_transcript29751	$2e^{-44}$	3.28967	320.58640	1753.05488	1025.47777	1431.60358	577.96267
		ta_transcript84821	$9e^{-38}$	15.25286	1166.56966	1903.77027	1769.00719	1486.28483	350.81894
		ta_transcript84818	$3e^{-37}$	14.97503	1045.64713	1483.80138	1517.12646	1283.70485	303.53292
		ta_transcript84816	$1e^{-36}$	19.83621	874.03672	1185.21216	1222.27996	961.04666	218.70870
		ta_transcript15565	$8e^{-33}$	1.56952	34.54302	43.71965	17.41703	2.81544	18.02279
Jhe-like	gi|512903969|ref|XM_004925690.1 | PREDICTED: *Bombyx mori* juvenile hormone esterase-like (LOC101737581)	ta_transcript75783	$3e^{-58}$	34.50366	407.57438	266.05792	520.28971	610.79065	125.98657
		ta_transcript58915	$4e^{-55}$	37.34863	887.77646	1276.91744	1483.85579	1109.38866	246.49795
		ta_transcript58914	$8e^{-55}$	11.31262	27.08608	33.68933	50.67899	383.84424	46.23666
		ta_transcript48580	$8e^{-54}$	8.37505	5.71547	17.51818	13.67925	19.31574	51.52006
		ta_transcript48579	$1e^{-53}$	13.41914	21.82808	35.84351	15.20656	15.82788	87.60035
		ta_transcript84818	$4e^{-42}$	14.97503	1045.64713	1483.80138	1517.12646	1283.70485	303.53292
		ta_transcript84821	$5e^{-42}$	15.25286	1166.56966	1903.77027	1769.00719	1486.28483	350.81894
		ta_transcript48393	$9e^{-42}$	33.94650	266.24385	639.13316	753.41294	386.85609	106.05076
		ta_transcript84816	$2e^{-41}$	19.83621	874.03672	1185.21216	1222.27996	961.04666	218.70870
		ta_transcript29751	$4e^{-36}$	3.28967	320.58640	1753.05488	1025.47777	1431.60358	577.96267
		ta_transcript82623	$8e^{-30}$	7.68942	23.98889	15.69177	30.29031	269.30098	28.03104
Jhe-like	gi|512931402|ref|XM_004932237.1 | PREDICTED: *Bombyx mori* juvenile hormone esterase-like (LOC101736068)	ta_transcript48580	$3e^{-67}$	8.37505	5.71547	17.51818	13.67925	19.31574	51.52006
		ta_transcript48579	$7e^{-67}$	13.41914	21.82808	35.84351	15.20656	15.82788	87.60035
		ta_transcript75783	$1e^{-52}$	34.50366	407.57438	266.05792	520.28971	610.79065	125.98657
		ta_transcript58915	$5e^{-52}$	37.34863	887.77646	1276.91744	1483.85579	1109.38866	246.49795
		ta_transcript58914	$6e^{-52}$	11.31262	27.08608	33.68933	50.67899	383.84424	46.23666
		ta_transcript84821	$2e^{-45}$	15.25286	1166.56966	1903.77027	1769.00719	1486.28483	350.81894

		ta_transcript84818<br>ta_transcript84816<br>ta_transcript29751<br>ta_transcript48393<br>ta_transcript15565	$3e^{-45}$<br>$2e^{-44}$<br>$4e^{-39}$<br>$1e^{-38}$<br>$2e^{-30}$	14.97503<br>19.83621<br>3.28967<br>33.94650<br>1.56952	1045.64713<br>874.03672<br>320.58640<br>266.24385<br>34.54302	1483.80138<br>1185.21216<br>1753.05488<br>639.13316<br>43.71965	1517.12646<br>1222.27996<br>1025.47777<br>753.41294<br>17.41703	1283.70485<br>961.04666<br>1431.60358<br>386.85609<br>2.81544	303.53292<br>218.70870<br>577.96267<br>106.05076<br>18.02279
Jhdk	gi|112983019:25-576 *Bombyx mori* juvenile hormone diol kinase (Jhdk)	ta_transcript24996	$2e^{-64}$	7.98318	2897.71770	4341.85397	8955.63041	17191.23626	18.87344
jhebp	gi|114052405:74-805 *Bombyx mori* juvenile hormone esterase binding protein (LOC733014)	ta_transcript22729	No hits found	-	-	-	-	-	-

Ecdysteroid Hormone									
Nvd	gi|112983363:40-1401 *Bombyx mori* neverland (Nvd)	ta_transcript14785	$4e^{-42}$	47.27870	18.73445	11.43771	32.31012	73.86644	23.35063
Cyp307a1	gi|162462655:125-1735 *Bombyx mori* cytochrome P450 (Cyp307a1)	ta_transcript75638<br>ta_transcript75639<br>ta_transcript75641<br>ta_transcript75637<br>ta_transcript75640<br>ta_transcript75642<br>ta_transcript75635<br>ta_transcript75636<br>ta_transcript75634<br>ta_transcript75632<br>ta_transcript75629<br>ta_transcript75630<br>ta_transcript75633<br>ta_transcript75631	$4e^{-38}$<br>$5e^{-38}$<br>$6e^{-38}$<br>$6e^{-38}$<br>$7e^{-38}$<br>$8e^{-38}$<br>$4e^{-37}$<br>$4e^{-37}$<br>$4e^{-37}$<br>$4e^{-37}$<br>$4e^{-37}$<br>$4e^{-37}$<br>$4e^{-37}$<br>$5e^{-37}$	15.84896<br>20.69867<br>25.23478<br>16.50092<br>19.87757<br>14.44189<br>21.70435<br>16.08231<br>16.15530<br>23.19600<br>22.55249<br>19.73040<br>11.45171<br>22.35448	6.12905<br>6.51951<br>5.35771<br>6.10963<br>6.54039<br>8.44776<br>5.96898<br>3.65840<br>7.27651<br>4.94684<br>5.89432<br>5.25456<br>2.97194<br>12.84104	22.64003<br>31.84221<br>24.43222<br>26.32969<br>27.00045<br>26.92622<br>19.78263<br>18.10022<br>21.46219<br>25.88685<br>28.44598<br>14.09231<br>16.93462<br>22.74370	30.20109<br>39.97789<br>31.68038<br>31.16796<br>39.38976<br>29.89549<br>24.83710<br>25.56544<br>31.29187<br>29.90087<br>32.27163<br>28.48124<br>25.70896<br>34.39546	26.53489<br>15.23272<br>17.43607<br>14.48498<br>21.01207<br>22.20531<br>27.89284<br>16.31852<br>23.18380<br>34.67460<br>24.67479<br>17.84030<br>19.09570<br>27.69495	7.07754<br>2.70864<br>13.59421<br>9.07087<br>5.09497<br>11.48651<br>4.95983<br>5.59616<br>8.03883<br>8.01546<br>13.77503<br>3.99097<br>9.56926<br>8.00254
Cyp306a1	gi|163838677:199-1815 *Bombyx mori* cytochrome P450 monooxygenase (Cyp306a1)	ta_transcript56351	$2e^{-49}$	125.65510	169.28259	286.91554	215.43291	255.37889	174.44381
Cyp302a1	gi|112983259:39-1592 *Bombyx mori* cytochrome P450 302A1 (Cyp302a1)	ta_transcript46148<br>ta_transcript46149	$9e^{-59}$<br>$3e^{-54}$	181.60988<br>200.81861	10.22001<br>17.20499	16.80844<br>19.86955	12.94957<br>18.12499	40.08238<br>48.86278	158.31792<br>134.21183
Cyp315a1	gi|163838681:81-1541 *Bombyx mori* cytochrome P450 (Cyp315a1)	ta_transcript32151<br>ta_transcript32147	$2e^{-34}$<br>$2e^{-34}$	88.11571<br>73.53156	5.87104<br>4.09640	8.93766<br>10.71825	7.36394<br>7.06480	24.31754<br>15.25404	18.95942<br>17.71058
Cyp314a1	gi|163838669:38-1588 *Bombyx mori* ecdysone 20-hydroxylase	ta_transcript86114<br>ta_transcript86113	$2e^{-36}$<br>$2e^{-36}$	123.49260<br>136.15536	23.63189<br>32.07751	75.89362<br>83.25953	115.48986<br>157.43079	242.79697<br>261.94521	52.05028<br>48.57745

	(Cyp314a1)								
Cyp18a1	gi|156119419:112-1737 *Bombyx mori* cytochrome P450 18a1 (Cyp18a1)	ta_transcript28089	$4e^{-114}$	12.55614	135.36042	284.59814	198.71248	841.11143	964.59469
		ta_transcript62517	$7e^{-48}$	10.51646	111.48048	211.81898	110.33630	58.69009	89.89964
		ta_transcript66869	$9e^{-32}$	5.42080	2.21962	25.55370	70.54542	161.41781	7.26217
		ta_transcript66866	$2e^{-31}$	1.65529	4.84129	11.65387	48.18832	124.71019	6.33589
ae40	gi|193082986:270-1907 *Bombyx mori* alpha-esterase 40 (ae40)	ta_transcript75783	$1e^{-93}$	34.50366	407.57438	266.05792	520.28971	610.79065	125.98657
		ta_transcript58915	$3e^{-93}$	37.34863	887.77646	1276.91744	1483.85579	1109.38866	246.49795
		ta_transcript58914	$8e^{-93}$	59.13909	797.21893	1194.23118	1468.53693	1208.72669	281.24140
		ta_transcript48580	$8e^{-93}$	8.37505	5.71547	17.51818	13.67925	19.31574	51.52006
		ta_transcript48579	$3e^{-92}$	13.41914	21.82808	35.84351	15.20656	15.82788	87.60035
		ta_transcript84821	$4e^{-79}$	15.25286	116656966	1903.77027	1769.00719	1486.28483	350.81894
		ta_transcript84818	$5e^{-78}$	14.97503	1045,64713	1483.80138	1517.12646	1283.70485	303.53292
		ta_transcript84816	$8e^{-77}$	19.83621	874,03672	1185.21216	1222.27996	961.04666	218.70870
		ta_transcript29751	$3e^{-70}$	3.28967	320,58640	1753.05488	1025.47777	1431.60358	577.96267
		ta_transcript48393	$9e^{-68}$	33.94650	266,24385	639.13316	753.41294	386.85609	106.05076
		ta_transcript49309	$6e^{-44}$	0.50122	0.50122	71.95660	18.20314	5.39460	64.26996
		ta_transcript15565	$2e^{-39}$	1.56952	34.54302	43.71965	17.41703	2.81544	18.02279
		ta_transcript20178	$3e^{-37}$	0.00000	1.10921	12.76991	9.29163	257.54508	1.55533
		ta_transcript74072	$2e^{-38}$	13.11473	125.90493	230.12694	239.22083	320.45542	33.23979
		ta_transcript74071	$6e^{-37}$	42.77186	183.77299	268.08750	430.61214	814.74793	171.23424
		ta_transcript46992	$4e^{-31}$	227.46690	86.70938	162.12679	98.41460	153.65348	224.04236
