# Supplementary material for: De novo transcriptome assembly and analysis to identify potential gene targets for RNAi-mediated control of the tomato leafminer (Tuta absoluta)
Source: BMC Genomics. 2015 Aug 26;16(1):635. doi: 10.1186/s12864-015-1841-5 (PMC4550053; doi:10.1186/s12864-015-1841-5)
Supplement: Additional file 10: Table S9. — BlastX results with the list of genes associated with biosynthesis of the two major hormone classes sesquiterpenoid juvenile (JH) and the ecdysteroid hormones identified in the T. absoluta assembled transcriptome at E-value < e−30 using homologues particularly from Bombyx mori. (PDF 468 kb) [file 12864_2015_1841_MOESM10_ESM.pdf]

Table S9. BlastX results with the list of genes associated with biosynthesis of the two major hormone classes sesquiterpenoid juvenile (JH) and the ecdysteroid hormones identified in the *T. absoluta* assembled transcriptome at E-value < e^{-30} using homologues particularly from *Bombyx mori*.

```
BLASTX 2.2.29+


Reference: Stephen F. Altschul, Thomas L. Madden, Alejandro A.
Schaffer, Jinghui Zhang, Zheng Zhang, Webb Miller, and David J.
Lipman (1997), "Gapped BLAST and PSI-BLAST: a new generation of
protein database search programs", Nucleic Acids Res. 25:3389-3402.



Database: herai/heraiprot.txt
           93,476 sequences; 49,077,216 total letters



Query= gi|112983457:82-1365 Bombyx mori farnesyl pyrophosphate syntase (Fps), mRNA

Length=1284
                                                                      Score     E
Sequences producing significant alignments:                          (Bits)  Value

  ta_transcript80181_1                                                  330   3e-107
  ta_transcript80180_1                                                  330   2e-106
  ta_transcript80179_1                                                  330   1e-104
  ta_transcript80177_1                                                  329   2e-102
  ta_transcript80178_1                                                  329   2e-102
  ta_transcript39822_1                                                  222   1e-64
  ta_transcript39820_1                                                  222   1e-64
  ta_transcript39821_1                                                  217   1e-62
  ta_transcript39819_1                                                  216   1e-62



> ta_transcript80181_1
Length=488

 Score =   330 bits (847),  Expect = 3e-107, Method: Compositional matrix adjust.
 Identities = 163/379 (43%), Positives = 241/379 (64%), Gaps = 13/379 (3%)
 Frame = +1

Query  178   LQKYHRFLSTLTPQEM-----PMATRGLA--VSKDQSRE---FMAVFPDIVRDLTETGKH  327
             + K  +F + + P  +      P   R LA  VS + S++   F  V P +V  L  + K
Sbjct  74    MTKTSKFCTNIQPVSVSRVLKPAGCRLLATNVSIEHSKDRETFQDVLPLVVDTLISSPKF  133

Query  328   IDVPEASKWLAKLLQYNVPNGKKNRGLATILAYKMLEKKENLTPENILLANVMGWCVEMF  507
              ++P+ + WL K+L+YN+  GKK RGL T+ AY+MLE  EN+T E++ LA +MGWCVEM
Sbjct  134   SEIPDVANWLKKILEYNLAGGKKARGLTTVFAYEMLETPENITDESMRLARIMGWCVEML  193

Query  508   HTHQLLLNDIMEGTTMRRGVPCWHRRPDVGLNGINDAALIQSAMYTSLKRHFNTKPYYNY  687
               + ++ +D+M+G+T RRGVPCW+R PDVGL  INDA LI S+++  + +HF   P Y
Sbjct  194   QAYFIVADDMMDGSTTRRGVPCWYRLPDVGLGAINDAILINSSLFEVINKHFRNYPQYAD  253

Query  688   VLETFNEMLMKCSMGHYVQKLMLK-TDKPDLSLFTMEKYEAITKYKTSYYTFQMPVSLAL  864
              L+ FNE ++  S G ++      +   K + SLFT+E+Y++I KYKT+YYT+++PVSL L
Sbjct  254   FLDLFNETILLTSAGQHLDFTTAQHLSKKNYSLFTIERYQSIVKYKTAYYTYRLPVSLGL  313

Query  865   LMTGVDDPETHRQAKTILLKMGEFFQIQDDFLDCFGDPTVTEKYGTDIQDGKCTWLAVVA  1044
             L+  V D  T +++  I   +GEFFQIQDD++D + D ++T K GTDIQ+GKC+WLAV A
Sbjct  314   LLANVTDKPTRQRSDEICFDIGEFFQIQDDYIDLYSDESLTGKAGTDIQEGKCSWLAVTA  373

Query  1045  LQRATPAQKQIMEDNYGVNKPEAIARIKDLYEELQLPHTYSVFEETTYDLLRTQIQQVTR  1224
             LQR TPAQ+ + E  Y    PE +A+IK LYE+L L    Y   E   Y+ +  +I  ++R
Sbjct  374   LQRCTPAQRALFEQCYASKNPEDVAQIKQLYEQLNLQKLYLEQERVKYEEIVRKIDALSR  433

Query  1225  --GLPHELFFKILDNIFRR  1275
                L  +LF K+++ I+ R
Sbjct  434   DTSLSPKLFLKLINMIYNR  452
```

```
> ta_transcript80180_1
Length=536

 Score =   330 bits (846),  Expect = 2e-106, Method: Compositional matrix adjust.
 Identities = 163/379 (43%), Positives = 241/379 (64%), Gaps = 13/379 (3%)
 Frame = +1

Query  178   LQKYHRFLSTLTPQEM-----PMATRGLA--VSKDQSRE---FMAVFPDIVRDLTETGKH  327
             + K  +F + + P  +     P   R LA  VS + S++   F  V P +V  L  + K
Sbjct  74    MTKTSKFCTNIQPVSVSRVLKPAGCRLLATNVSIEHSKDRETFQDVLPLVVDTLISSPKF  133

Query  328   IDVPEASKWLAKLLQYNVPNGKKNRGLATILAYKMLEKKENLTPENILLANVMGWCVEMF  507
              ++P+ + WL K+L+YN+  GKK RGL T+ AY+MLE  EN+T E++ LA +MGWCVEM
Sbjct  134   SEIPDVANWLKKILEYNLAGGKKARGLTTVFAYEMLETPENITDESMRLARIMGWCVEML  193

Query  508   HTHQLLLNDIMEGTTMRRGVPCWHRRPDVGLNGINDAALIQSAMYTSLKRHFNTKPYYNY  687
               + ++ +D+M+G+T RRGVPCW+R PDVGL  INDA LI S+++  + +HF   P Y
Sbjct  194   QAYFIVADDMMDGSTTRRGVPCWYRLPDVGLGAINDAILINSSLFEVINKHFRNYPQYAD  253

Query  688   VLETFNEMLMKCSMGHYVQKLMLK-TDKPDLSLFTMEKYEAITKYKTSYYTFQMPVSLAL  864
              L+ FNE ++  S G ++     +   K + SLFT+E+Y++I KYKT+YYT+++PVSL L
Sbjct  254   FLDLFNETILLTSAGQHLDFTTAQHLSKKNYSLFTIERYQSIVKYKTAYYTYRLPVSLGL  313

Query  865   LMTGVDDPETHRQAKTILLKMGEFFQIQDDFLDCFGDPTVTEKYGTDIQDGKCTWLAVVA  1044
             L+  V D  T +++  I   +GEFFQIQDD++D + D ++T K GTDIQ+GKC+WLAV A
Sbjct  314   LLANVTDKPTRQRSDEICFDIGEFFQIQDDYIDLYSDESLTGKAGTDIQEGKCSWLAVTA  373

Query  1045  LQRATPAQKQIMEDNYGVNKPEAIARIKDLYEELQLPHTYSVFEETTYDLLRTQIQQVTR  1224
             LQR TPAQ+ + E  Y    PE +A+IK LYE+L L   Y   E   Y+ +  +I  ++R
Sbjct  374   LQRCTPAQRALFEQCYASKNPEDVAQIKQLYEQLNLQKLYLEQERVKYEEIVRKIDALSR  433

Query  1225  --GLPHELFFKILDNIFRR  1275
                L  +LF K+++ I+ R
Sbjct  434   DTSLSPKLFLKLINMIYNR  452


> ta_transcript80179_1
Length=671

 Score =   330 bits (845),  Expect = 1e-104, Method: Compositional matrix adjust.
 Identities = 163/379 (43%), Positives = 241/379 (64%), Gaps = 13/379 (3%)
 Frame = +1

Query  178   LQKYHRFLSTLTPQEM-----PMATRGLA--VSKDQSRE---FMAVFPDIVRDLTETGKH  327
             + K  +F + + P  +     P   R LA  VS + S++   F  V P +V  L  + K
Sbjct  74    MTKTSKFCTNIQPVSVSRVLKPAGCRLLATNVSIEHSKDRETFQDVLPLVVDTLISSPKF  133

Query  328   IDVPEASKWLAKLLQYNVPNGKKNRGLATILAYKMLEKKENLTPENILLANVMGWCVEMF  507
              ++P+ + WL K+L+YN+  GKK RGL T+ AY+MLE  EN+T E++ LA +MGWCVEM
Sbjct  134   SEIPDVANWLKKILEYNLAGGKKARGLTTVFAYEMLETPENITDESMRLARIMGWCVEML  193

Query  508   HTHQLLLNDIMEGTTMRRGVPCWHRRPDVGLNGINDAALIQSAMYTSLKRHFNTKPYYNY  687
               + ++ +D+M+G+T RRGVPCW+R PDVGL  INDA LI S+++  + +HF   P Y
Sbjct  194   QAYFIVADDMMDGSTTRRGVPCWYRLPDVGLGAINDAILINSSLFEVINKHFRNYPQYAD  253

Query  688   VLETFNEMLMKCSMGHYVQKLMLK-TDKPDLSLFTMEKYEAITKYKTSYYTFQMPVSLAL  864
              L+ FNE ++  S G ++     +   K + SLFT+E+Y++I KYKT+YYT+++PVSL L
Sbjct  254   FLDLFNETILLTSAGQHLDFTTAQHLSKKNYSLFTIERYQSIVKYKTAYYTYRLPVSLGL  313

Query  865   LMTGVDDPETHRQAKTILLKMGEFFQIQDDFLDCFGDPTVTEKYGTDIQDGKCTWLAVVA  1044
             L+  V D  T +++  I   +GEFFQIQDD++D + D ++T K GTDIQ+GKC+WLAV A
Sbjct  314   LLANVTDKPTRQRSDEICFDIGEFFQIQDDYIDLYSDESLTGKAGTDIQEGKCSWLAVTA  373

Query  1045  LQRATPAQKQIMEDNYGVNKPEAIARIKDLYEELQLPHTYSVFEETTYDLLRTQIQQVTR  1224
             LQR TPAQ+ + E  Y    PE +A+IK LYE+L L   Y   E   Y+ +  +I  ++R
Sbjct  374   LQRCTPAQRALFEQCYASKNPEDVAQIKQLYEQLNLQKLYLEQERVKYEEIVRKIDALSR  433

Query  1225  --GLPHELFFKILDNIFRR  1275
                L  +LF K+++ I+ R
Sbjct  434   DTSLSPKLFLKLINMIYNR  452


> ta_transcript80177_1
Length=889
```

```
 Score =   329 bits (843),  Expect = 2e-102, Method: Compositional matrix adjust.
 Identities = 163/379 (43%), Positives = 241/379 (64%), Gaps = 13/379 (3%)
 Frame = +1

Query  178   LQKYHRFLSTLTPQEM-----PMATRGLA--VSKDQSRE---FMAVFPDIVRDLTETGKH  327
             + K  +F + + P  +     P   R LA  VS + S++   F  V P +V  L  + K
Sbjct  74    MTKTSKFCTNIQPVSVSRVLKPAGCRLLATNVSIEHSKDRETFQDVLPLVVDTLISSPKF  133

Query  328   IDVPEASKWLAKLLQYNVPNGKKNRGLATILAYKMLEKKENLTPENILLANVMGWCVEMF  507
              ++P+ + WL K+L+YN+  GKK RGL T+ AY+MLE  EN+T E++ LA +MGWCVEM
Sbjct  134   SEIPDVANWLKKILEYNLAGGKKARGLTTVFAYEMLETPENITDESMRLARIMGWCVEML  193

Query  508   HTHQLLLNDIMEGTTMRRGVPCWHRRPDVGLNGINDAALIQSAMYTSLKRHFNTKPYYNY  687
               + ++ +D+M+G+T RRGVPCW+R PDVGL  INDA LI S+++  + +HF   P Y
Sbjct  194   QAYFIVADDMMDGSTTRRGVPCWYRLPDVGLGAINDAILINSSLFEVINKHFRNYPQYAD  253

Query  688   VLETFNEMLMKCSMGHYVQKLMLK-TDKPDLSLFTMEKYEAITKYKTSYYTFQMPVSLAL  864
              L+ FNE ++  S G ++     +   K + SLFT+E+Y++I KYKT+YYT+++PVSL L
Sbjct  254   FLDLFNETILLTSAGQHLDFTTAQHLSKKNYSLFTIERYQSIVKYKTAYYTYRLPVSLGL  313

Query  865   LMTGVDDPETHRQAKTILLKMGEFFQIQDDFLDCFGDPTVTEKYGTDIQDGKCTWLAVVA  1044
             L+  V D  T +++  I   +GEFFQIQDD++D + D ++T K GTDIQ+GKC+WLAV A
Sbjct  314   LLANVTDKPTRQRSDEICFDIGEFFQIQDDYIDLYSDESLTGKAGTDIQEGKCSWLAVTA  373

Query  1045  LQRATPAQKQIMEDNYGVNKPEAIARIKDLYEELQLPHTYSVFEETTYDLLRTQIQQVTR  1224
             LQR TPAQ+ + E  Y    PE +A+IK LYE+L L   Y   E   Y+ +  +I  ++R
Sbjct  374   LQRCTPAQRALFEQCYASKNPEDVAQIKQLYEQLNLQKLYLEQERVKYEEIVRKIDALSR  433

Query  1225  --GLPHELFFKILDNIFRR  1275
                L  +LF K+++ I+ R
Sbjct  434   DTSLSPKLFLKLINMIYNR  452


> ta_transcript80178_1
Length=883

 Score =   329 bits (843),  Expect = 2e-102, Method: Compositional matrix adjust.
 Identities = 163/379 (43%), Positives = 241/379 (64%), Gaps = 13/379 (3%)
 Frame = +1

Query  178   LQKYHRFLSTLTPQEM-----PMATRGLA--VSKDQSRE---FMAVFPDIVRDLTETGKH  327
             + K  +F + + P  +     P   R LA  VS + S++   F  V P +V  L  + K
Sbjct  74    MTKTSKFCTNIQPVSVSRVLKPAGCRLLATNVSIEHSKDRETFQDVLPLVVDTLISSPKF  133

Query  328   IDVPEASKWLAKLLQYNVPNGKKNRGLATILAYKMLEKKENLTPENILLANVMGWCVEMF  507
              ++P+ + WL K+L+YN+  GKK RGL T+ AY+MLE  EN+T E++ LA +MGWCVEM
Sbjct  134   SEIPDVANWLKKILEYNLAGGKKARGLTTVFAYEMLETPENITDESMRLARIMGWCVEML  193

Query  508   HTHQLLLNDIMEGTTMRRGVPCWHRRPDVGLNGINDAALIQSAMYTSLKRHFNTKPYYNY  687
               + ++ +D+M+G+T RRGVPCW+R PDVGL  INDA LI S+++  + +HF   P Y
Sbjct  194   QAYFIVADDMMDGSTTRRGVPCWYRLPDVGLGAINDAILINSSLFEVINKHFRNYPQYAD  253

Query  688   VLETFNEMLMKCSMGHYVQKLMLK-TDKPDLSLFTMEKYEAITKYKTSYYTFQMPVSLAL  864
              L+ FNE ++  S G ++     +   K + SLFT+E+Y++I KYKT+YYT+++PVSL L
Sbjct  254   FLDLFNETILLTSAGQHLDFTTAQHLSKKNYSLFTIERYQSIVKYKTAYYTYRLPVSLGL  313

Query  865   LMTGVDDPETHRQAKTILLKMGEFFQIQDDFLDCFGDPTVTEKYGTDIQDGKCTWLAVVA  1044
             L+  V D  T +++  I   +GEFFQIQDD++D + D ++T K GTDIQ+GKC+WLAV A
Sbjct  314   LLANVTDKPTRQRSDEICFDIGEFFQIQDDYIDLYSDESLTGKAGTDIQEGKCSWLAVTA  373

Query  1045  LQRATPAQKQIMEDNYGVNKPEAIARIKDLYEELQLPHTYSVFEETTYDLLRTQIQQVTR  1224
             LQR TPAQ+ + E  Y    PE +A+IK LYE+L L   Y   E   Y+ +  +I  ++R
Sbjct  374   LQRCTPAQRALFEQCYASKNPEDVAQIKQLYEQLNLQKLYLEQERVKYEEIVRKIDALSR  433

Query  1225  --GLPHELFFKILDNIFRR  1275
                L  +LF K+++ I+ R
Sbjct  434   DTSLSPKLFLKLINMIYNR  452


> ta_transcript39822_1
Length=593

 Score =   222 bits (566),  Expect = 1e-64, Method: Compositional matrix adjust.
 Identities = 184/412 (45%), Positives = 225/412 (55%), Gaps = 0/412 (0%)
 Frame = +2

Query  2     CSRQRKA*KSLCRRTKMKYAGTSARQLASPILMPWPRGWTSRRASRHRLRKPDRRDC*SC  181
```

```
             CSRQ +A +  CR T+M+ AG SAR+   P    W  GWT    S  R    + R C*SC
Sbjct  41    CSRQERAWRRSCRSTRMRSAGRSARRPV*PTRTRWCPGWTRAAGSIRRKTSLEPRSC*SC  100

Query  182   KNITDSCRP*LLRKCRWLPEDWPFPRTSPGSSWLSFRTSSGISLRLASTLMFRKPANGWL  361
             +N T +C+ *   +CRW    WP  RTS  SSW + RTSSG S R AST    K  +GWL
Sbjct  101   RNTTGTCQR*RATRCRWRRAGWPSRRTSRASSWPASRTSSGTSRRPASTTTCPKLPSGWL  160

Query  362   SCCNIMYQTVRRIGDWPRS*LTKCLRRKKI*RQRTFCLQTLWDGASKCSTPTNFY*TTSW  541
             SCCN   +  RR     +   T+C RR+ I   RT   +  W G  +CST T+    TT W
Sbjct  161   SCCNTTCRMARRTAG*RQCWRTRCWRRRIISAPRTSTWRISWAGVLRCSTQTSSSRTT*W  220

Query  542   KVPRCVAAFRAGTDGLTSV*TASTTLP*SNPQCIPV*NGTSTPSHITITYLKPSMRC**N  721
             + P   A    GT G    * ASTT P  +P C P   GTS PS  T T  + SMRC**N
Sbjct  221   RTPSPAAGCPPGTRGPKWG*AASTTQPWYSPPCSPRSRGTSEPSLTTRTCWRCSMRC**N  280

Query  722   VQWVTTFKN*C*KPISQTYLCSQWRNTKRSLNTRLLITHSKCPSRWHC**PASTIQRPTD  901
              Q VTT ++       +T L S+WR TKR  +TR   T SKC S WHC *P S   R T
Sbjct  281   AQSVTT*RSR*PLQTGRTCLSSRWRTTKR*PSTRPRTTPSKCRSAWHCS*PGSMTPRRTG  340

Query  902   KLKLFC*RWESSSKFKMIFWTASGTPR*PKSTVPTFKMENALGWQSLHCRGQLLPKNKSW  1081
             + +  C RWESSSKFKM   T S T +*     PT K ENA G QS  CRG  L KN S
Sbjct  341   RPRTSCWRWESSSKFKMTSSTVSATQQ*SGKRAPTSKTENARGSQSSPCRGHHLRKNSSC  400

Query  1082  KTITESINLKLSPGLKISMKNFSYLIRTRSSKKLPTISLERRSSKSQGDYLT  1237
             KTIT   + L+  P  +    +  +Y  R+ S+K+L TISL R+S+KS   +LT
Sbjct  401   KTITPVLTLQT*PK*RTYTRTSNYHTRSPSTKRLLTISLGRKSNKSPEVFLT  452


> ta_transcript39820_1
Length=597

 Score =   222 bits (565),  Expect = 1e-64, Method: Compositional matrix adjust.
 Identities = 184/412 (45%), Positives = 225/412 (55%), Gaps = 0/412 (0%)
 Frame = +2

Query  2     CSRQRKA*KSLCRRTKMKYAGTSARQLASPILMPWPRGWTSRRASRHRLRKPDRRDC*SC  181
             CSRQ +A +  CR T+M+ AG SAR+   P    W  GWT    S  R    + R C*SC
Sbjct  45    CSRQERAWRRSCRSTRMRSAGRSARRPV*PTRTRWCPGWTRAAGSIRRKTSLEPRSC*SC  104

Query  182   KNITDSCRP*LLRKCRWLPEDWPFPRTSPGSSWLSFRTSSGISLRLASTLMFRKPANGWL  361
             +N T +C+ *   +CRW    WP  RTS  SSW + RTSSG S R AST    K  +GWL
Sbjct  105   RNTTGTCQR*RATRCRWRRAGWPSRRTSRASSWPASRTSSGTSRRPASTTTCPKLPSGWL  164

Query  362   SCCNIMYQTVRRIGDWPRS*LTKCLRRKKI*RQRTFCLQTLWDGASKCSTPTNFY*TTSW  541
             SCCN   +  RR     +   T+C RR+ I   RT   +  W G  +CST T+    TT W
Sbjct  165   SCCNTTCRMARRTAG*RQCWRTRCWRRRIISAPRTSTWRISWAGVLRCSTQTSSSRTT*W  224

Query  542   KVPRCVAAFRAGTDGLTSV*TASTTLP*SNPQCIPV*NGTSTPSHITITYLKPSMRC**N  721
             + P   A    GT G    * ASTT P  +P C P   GTS PS  T T  + SMRC**N
Sbjct  225   RTPSPAAGCPPGTRGPKWG*AASTTQPWYSPPCSPRSRGTSEPSLTTRTCWRCSMRC**N  284

Query  722   VQWVTTFKN*C*KPISQTYLCSQWRNTKRSLNTRLLITHSKCPSRWHC**PASTIQRPTD  901
              Q VTT ++       +T L S+WR TKR  +TR   T SKC S WHC *P S   R T
Sbjct  285   AQSVTT*RSR*PLQTGRTCLSSRWRTTKR*PSTRPRTTPSKCRSAWHCS*PGSMTPRRTG  344

Query  902   KLKLFC*RWESSSKFKMIFWTASGTPR*PKSTVPTFKMENALGWQSLHCRGQLLPKNKSW  1081
             + +  C RWESSSKFKM   T S T +*     PT K ENA G QS  CRG  L KN S
Sbjct  345   RPRTSCWRWESSSKFKMTSSTVSATQQ*SGKRAPTSKTENARGSQSSPCRGHHLRKNSSC  404

Query  1082  KTITESINLKLSPGLKISMKNFSYLIRTRSSKKLPTISLERRSSKSQGDYLT  1237
             KTIT   + L+  P  +    +  +Y  R+ S+K+L TISL R+S+KS   +LT
Sbjct  405   KTITPVLTLQT*PK*RTYTRTSNYHTRSPSTKRLLTISLGRKSNKSPEVFLT  456


> ta_transcript39821_1
Length=597

 Score =   217 bits (552),  Expect = 1e-62, Method: Compositional matrix adjust.
 Identities = 184/416 (44%), Positives = 225/416 (54%), Gaps = 4/416 (1%)
 Frame = +2

Query  2     CSRQRKA*KSLCRRTKMKYAGTSARQLASPILMPWPRGWTSRRASRHRLRKPDRRDC*SC  181
             CSRQ +A +  CR T+M+ AG SAR+   P    W  GWT    S  R    + R C*SC
Sbjct  41    CSRQERAWRRSCRSTRMRSAGRSARRPV*PTRTRWCPGWTRAAGSIRRKTSLEPRSC*SC  100

Query  182   KNITDSCRP*LLRKCRWLPEDWPFPRTSPGSSWLSFRTSSGISLRLASTLMFRKPANGWL  361
             +N T +C+ *   +CRW    WP  RTS  SSW + RTSSG S R AST    K  +GWL
```

```
Sbjct  101   RNTTGTCQR*RATRCRWRRAGWPSRRTSRASSWPASRTSSGTSRRPASTTTCPKLPSGWL  160

Query  362   S----CCNIMYQTVRRIGDWPRS*LTKCLRRKKI*RQRTFCLQTLWDGASKCSTPTNFY*  529
             S    CCN    +  RR      +    T+C RR+ I    RT    +  W G  +CST T+
Sbjct  161   SACCSCCNTTCRMARRTAG*RQCWRTRCWRRRIISAPRTSTWRISWAGVLRCSTQTSSSR  220

Query  530   TTSWKVPRCVAAFRAGTDGLTSV*TASTTLP*SNPQCIPV*NGTSTPSHITITYLKPSMR  709
             TT W+ P    A     GT G     * ASTT P   +P C P    GTS PS   T T   + SMR
Sbjct  221   TT*WRTPSPAAGCPPGTRGPKWG*AASTTQPWYSPPCSPRSRGTSEPSLTTRTCWRCSMR  280

Query  710   C**NVQWVTTFKN*C*KPISQTYLCSQWRNTKRSLNTRLLITHSKCPSRWHC**PASTIQ  889
             C**N Q VTT ++        +T L S+WR TKR  +TR    T SKC S WHC *P S
Sbjct  281   C**NAQSVTT*RSR*PLQTGRTCLSSRWRTTKR*PSTRPRTTPSKCRSAWHCS*PGSMTP  340

Query  890   RPTDKLKLFC*RWESSSKFKMIFWTASGTPR*PKSTVPTFKMENALGWQSLHCRGQLLPK  1069
             R T + +  C RWESSSKFKM    T S T +*      PT K ENA G QS   CRG  L K
Sbjct  341   RRTGRPRTSCWRWESSSKFKMTSSTVSATQQ*SGKRAPTSKTENARGSQSSPCRGHHLRK  400

Query  1070  NKSWKTITESINLKLSPGLKISMKNFSYLIRTRSSKKLPTISLERRSSKSQGDYLT  1237
             N S KTIT   + L+   P   +    +  +Y   R+ S+K+L TISL R+S+KS    +LT
Sbjct  401   NSSCKTITPVLTLQT*PK*RTYTRTSNYHTRSPSTKRLLTISLGRKSNKSPEVFLT  456


> ta_transcript39819_1
Length=601

 Score =   216 bits (551),  Expect = 1e-62, Method: Compositional matrix adjust.
 Identities = 184/416 (44%), Positives = 225/416 (54%), Gaps = 4/416 (1%)
 Frame = +2

Query  2     CSRQRKA*KSLCRRTKMKYAGTSARQLASPILMPWPRGWTSRRASRHRLRKPDRRDC*SC  181
             CSRQ +A +   CR T+M+ AG SAR+    P     W  GWT     S  R     + R C*SC
Sbjct  45    CSRQERAWRRSCRSTRMRSAGRSARRPV*PTRTRWCPGWTRAAGSIRRKTSLEPRSC*SC  104

Query  182   KNITDSCRP*LLRKCRWLPEDWPFPRTSPGSSWLSFRTSSGISLRLASTLMFRKPANGWL  361
             +N T +C+ *    +CRW    WP  RTS   SSW + RTSSG S R AST    K  +GWL
Sbjct  105   RNTTGTCQR*RATRCRWRRAGWPSRRTSRASSWPASRTSSGTSRRPASTTTCPKLPSGWL  164

Query  362   S----CCNIMYQTVRRIGDWPRS*LTKCLRRKKI*RQRTFCLQTLWDGASKCSTPTNFY*  529
             S    CCN    +  RR      +    T+C RR+ I    RT    +  W G  +CST T+
Sbjct  165   SACCSCCNTTCRMARRTAG*RQCWRTRCWRRRIISAPRTSTWRISWAGVLRCSTQTSSSR  224

Query  530   TTSWKVPRCVAAFRAGTDGLTSV*TASTTLP*SNPQCIPV*NGTSTPSHITITYLKPSMR  709
             TT W+ P    A     GT G     * ASTT P   +P C P    GTS PS   T T   + SMR
Sbjct  225   TT*WRTPSPAAGCPPGTRGPKWG*AASTTQPWYSPPCSPRSRGTSEPSLTTRTCWRCSMR  284

Query  710   C**NVQWVTTFKN*C*KPISQTYLCSQWRNTKRSLNTRLLITHSKCPSRWHC**PASTIQ  889
             C**N Q VTT ++        +T L S+WR TKR  +TR    T SKC S WHC *P S
Sbjct  285   C**NAQSVTT*RSR*PLQTGRTCLSSRWRTTKR*PSTRPRTTPSKCRSAWHCS*PGSMTP  344

Query  890   RPTDKLKLFC*RWESSSKFKMIFWTASGTPR*PKSTVPTFKMENALGWQSLHCRGQLLPK  1069
             R T + +  C RWESSSKFKM    T S T +*      PT K ENA G QS   CRG  L K
Sbjct  345   RRTGRPRTSCWRWESSSKFKMTSSTVSATQQ*SGKRAPTSKTENARGSQSSPCRGHHLRK  404

Query  1070  NKSWKTITESINLKLSPGLKISMKNFSYLIRTRSSKKLPTISLERRSSKSQGDYLT  1237
             N S KTIT   + L+   P   +    +  +Y   R+ S+K+L TISL R+S+KS    +LT
Sbjct  405   NSSCKTITPVLTLQT*PK*RTYTRTSNYHTRSPSTKRLLTISLGRKSNKSPEVFLT  460


***********************************************************************************************
*******************************************************

Query= gi|153791941:133-1281 Bombyx mori farnesyl diphosphate synthase 2 (Fpps2), mRNA

Length=1149
                                                                      Score     E
Sequences producing significant alignments:                          (Bits)  Value

  ta_transcript80181_1                                                  359    7e-119
  ta_transcript80180_1                                                  358    5e-118
  ta_transcript80179_1                                                  358    3e-116
  ta_transcript80178_1                                                  358    3e-114
  ta_transcript80177_1                                                  358    6e-114


> ta_transcript80181_1
Length=488
```

```
 Score =   359 bits (921),  Expect = 7e-119, Method: Compositional matrix adjust.
 Identities = 182/351 (52%), Positives = 249/351 (71%), Gaps = 5/351 (1%)
 Frame = +1

Query  109   NLEIINEKKMFDDLLPEVIMTLQNKSKLSEVPQIGDWLKKMLHYNLVGGKHTRGITTVIS  288
             ++E   +++ F D+LP V+ TL +  K SE+P + +WLKK+L YNL GGK  RG+TTV +
Sbjct  106   SIEHSKDRETFQDVLPLVVDTLISSPKFSEIPDVANWLKKILEYNLAGGKKARGLTTVFA  165

Query  289   YKTIEKPEKVTEHTLKMACKLGWCVEMFQAYCIVLDDIMDGSSVRRGMPCWYRRPEVGIT  468
             Y+ +E PE +T+ ++++A  +GWCVEM QAY IV DD+MDGS+ RRG+PCWYR P+VG+
Sbjct  166   YEMLETPENITDESMRLARIMGWCVEMLQAYFIVADDMMDGSTTRRGVPCWYRLPDVGLG  225

Query  469   CAFNDSLLIHSSLFEFLKTNFRTNPNYMKMFELFNETLWRTSMGQHLDHVTGNR--KTDY  642
              A ND++LI+SSLFE +  +FR  P Y    +LFNET+  TS GQHLD  T     K +Y
Sbjct  226   -AINDAILINSSLFEVINKHFRNYPQYADFLDLFNETILLTSAGQHLDFTTAQHLSKKNY  284

Query  643   SSFTLDRyytiikykaayytyNLPVSLGLLLAENVDEKIYKSAQDICLEIGTMFQIQDDF  822
             S FT++RY +I+KYK AYYTY LPVSLGLLLA   D+   + + +IC +IG  FQIQDD+
Sbjct  285   SLFTIERYQSIVKYKTAYYTYRLPVSLGLLLANVTDKPTRQRSDEICFDIGEFFQIQDDY  344

Query  823   IDCFGDEIKTGKVGTDIQERKCTWLAVQALQRCTEAQRTVFKACYGSSEPAHVERIKRLY  1002
             ID + DE  TGK GTDIQE KC+WLAV ALQRCT AQR +F+ CY S   P  V +IK+LY
Sbjct  345   IDLYSDESLTGKAGTDIQEGKCSWLAVTALQRCTPAQRALFEQCYASKNPEDVAQIKQLY  404

Query  1003  EDLHLPQIYKHQEKAMYDNIIRQIENIPIEAARV--LFKKLLDITYNRQH*  1149
             E L+L ++Y  QE+  Y+ I+R+I+ +  + +    LF KL+++ YNR+ *
Sbjct  405   EQLNLQKLYLEQERVKYEEIVRKIDALSRDTSLSPKLFLKLINMIYNRKR*  455


> ta_transcript80180_1
Length=536

 Score =   358 bits (919),  Expect = 5e-118, Method: Compositional matrix adjust.
 Identities = 183/363 (50%), Positives = 252/363 (69%), Gaps = 5/363 (1%)
 Frame = +1

Query  73    KNNSANMTTASKNLEIINEKKMFDDLLPEVIMTLQNKSKLSEVPQIGDWLKKMLHYNLVG  252
             K     +   + ++E   +++ F D+LP V+ TL +  K SE+P + +WLKK+L YNL G
Sbjct  94    KPAGCRLLATNVSIEHSKDRETFQDVLPLVVDTLISSPKFSEIPDVANWLKKILEYNLAG  153

Query  253   GKHTRGITTVISYKTIEKPEKVTEHTLKMACKLGWCVEMFQAYCIVLDDIMDGSSVRRGM  432
             GK  RG+TTV +Y+ +E PE +T+ ++++A  +GWCVEM QAY IV DD+MDGS+ RRG+
Sbjct  154   GKKARGLTTVFAYEMLETPENITDESMRLARIMGWCVEMLQAYFIVADDMMDGSTTRRGV  213

Query  433   PCWYRRPEVGITCAFNDSLLIHSSLFEFLKTNFRTNPNYMKMFELFNETLWRTSMGQHLD  612
             PCWYR P+VG+  A ND++LI+SSLFE +  +FR  P Y    +LFNET+  TS GQHLD
Sbjct  214   PCWYRLPDVGLG-AINDAILINSSLFEVINKHFRNYPQYADFLDLFNETILLTSAGQHLD  272

Query  613   HVTGNR--KTDYSSFTLDRyytiikykaayytyNLPVSLGLLLAENVDEKIYKSAQDICL  786
               T     K +YS FT++RY +I+KYK AYYTY LPVSLGLLLA   D+   + + +IC
Sbjct  273   FTTAQHLSKKNYSLFTIERYQSIVKYKTAYYTYRLPVSLGLLLANVTDKPTRQRSDEICF  332

Query  787   EIGTMFQIQDDFIDCFGDEIKTGKVGTDIQERKCTWLAVQALQRCTEAQRTVFKACYGSS  966
             +IG  FQIQDD+ID + DE  TGK GTDIQE KC+WLAV ALQRCT AQR +F+ CY S
Sbjct  333   DIGEFFQIQDDYIDLYSDESLTGKAGTDIQEGKCSWLAVTALQRCTPAQRALFEQCYASK  392

Query  967   EPAHVERIKRLYEDLHLPQIYKHQEKAMYDNIIRQIENIPIEAARV--LFKKLLDITYNR  1140
              P  V +IK+LYE L+L ++Y  QE+  Y+ I+R+I+ +  + +    LF KL+++ YNR
Sbjct  393   NPEDVAQIKQLYEQLNLQKLYLEQERVKYEEIVRKIDALSRDTSLSPKLFLKLINMIYNR  452

Query  1141  QH*  1149
             + *
Sbjct  453   KR*  455


> ta_transcript80179_1
Length=671

 Score =   358 bits (919),  Expect = 3e-116, Method: Compositional matrix adjust.
 Identities = 183/363 (50%), Positives = 252/363 (69%), Gaps = 5/363 (1%)
 Frame = +1

Query  73    KNNSANMTTASKNLEIINEKKMFDDLLPEVIMTLQNKSKLSEVPQIGDWLKKMLHYNLVG  252
             K     +   + ++E   +++ F D+LP V+ TL +  K SE+P + +WLKK+L YNL G
Sbjct  94    KPAGCRLLATNVSIEHSKDRETFQDVLPLVVDTLISSPKFSEIPDVANWLKKILEYNLAG  153
```

```
Query  253   GKHTRGITTVISYKTIEKPEKVTEHTLKMACKLGWCVEMFQAYCIVLDDIMDGSSVRRGM  432
             GK  RG+TTV +Y+ +E PE +T+ ++++A  +GWCVEM QAY IV DD+MDGS+ RRG+
Sbjct  154   GKKARGLTTVFAYEMLETPENITDESMRLARIMGWCVEMLQAYFIVADDMMDGSTTRRGV  213

Query  433   PCWYRRPEVGITCAFNDSLLIHSSLFEFLKTNFRTNPNYMKMFELFNETLWRTSMGQHLD  612
             PCWYR P+VG+  A ND++LI+SSLFE +  +FR  P Y    +LFNET+  TS GQHLD
Sbjct  214   PCWYRLPDVGLG-AINDAILINSSLFEVINKHFRNYPQYADFLDLFNETILLTSAGQHLD  272

Query  613   HVTGNR--KTDYSSFTLDRyytiikykaayytyNLPVSLGLLLAENVDEKIYKSAQDICL  786
               T     K +YS FT++RY +I+KYK AYYTY LPVSLGLLLA   D+   + + +IC
Sbjct  273   FTTAQHLSKKNYSLFTIERYQSIVKYKTAYYTYRLPVSLGLLLANVTDKPTRQRSDEICF  332

Query  787   EIGTMFQIQDDFIDCFGDEIKTGKVGTDIQERKCTWLAVQALQRCTEAQRTVFKACYGSS  966
             +IG  FQIQDD+ID + DE  TGK GTDIQE KC+WLAV ALQRCT AQR +F+ CY S
Sbjct  333   DIGEFFQIQDDYIDLYSDESLTGKAGTDIQEGKCSWLAVTALQRCTPAQRALFEQCYASK  392

Query  967   EPAHVERIKRLYEDLHLPQIYKHQEKAMYDNIIRQIENIPIEAARV--LFKKLLDITYNR  1140
              P  V +IK+LYE L+L ++Y  QE+  Y+ I+R+I+ +  + +    LF KL+++ YNR
Sbjct  393   NPEDVAQIKQLYEQLNLQKLYLEQERVKYEEIVRKIDALSRDTSLSPKLFLKLINMIYNR  452

Query  1141  QH*  1149
             + *
Sbjct  453   KR*  455


> ta_transcript80178_1
Length=883

 Score =   358 bits (919),  Expect = 3e-114, Method: Compositional matrix adjust.
 Identities = 183/363 (50%), Positives = 252/363 (69%), Gaps = 5/363 (1%)
 Frame = +1

Query  73    KNNSANMTTASKNLEIINEKKMFDDLLPEVIMTLQNKSKLSEVPQIGDWLKKMLHYNLVG  252
             K     +   + ++E   +++ F D+LP V+ TL +  K SE+P + +WLKK+L YNL G
Sbjct  94    KPAGCRLLATNVSIEHSKDRETFQDVLPLVVDTLISSPKFSEIPDVANWLKKILEYNLAG  153

Query  253   GKHTRGITTVISYKTIEKPEKVTEHTLKMACKLGWCVEMFQAYCIVLDDIMDGSSVRRGM  432
             GK  RG+TTV +Y+ +E PE +T+ ++++A  +GWCVEM QAY IV DD+MDGS+ RRG+
Sbjct  154   GKKARGLTTVFAYEMLETPENITDESMRLARIMGWCVEMLQAYFIVADDMMDGSTTRRGV  213

Query  433   PCWYRRPEVGITCAFNDSLLIHSSLFEFLKTNFRTNPNYMKMFELFNETLWRTSMGQHLD  612
             PCWYR P+VG+  A ND++LI+SSLFE +  +FR  P Y    +LFNET+  TS GQHLD
Sbjct  214   PCWYRLPDVGLG-AINDAILINSSLFEVINKHFRNYPQYADFLDLFNETILLTSAGQHLD  272

Query  613   HVTGNR--KTDYSSFTLDRyytiikykaayytyNLPVSLGLLLAENVDEKIYKSAQDICL  786
               T     K +YS FT++RY +I+KYK AYYTY LPVSLGLLLA   D+   + + +IC
Sbjct  273   FTTAQHLSKKNYSLFTIERYQSIVKYKTAYYTYRLPVSLGLLLANVTDKPTRQRSDEICF  332

Query  787   EIGTMFQIQDDFIDCFGDEIKTGKVGTDIQERKCTWLAVQALQRCTEAQRTVFKACYGSS  966
             +IG  FQIQDD+ID + DE  TGK GTDIQE KC+WLAV ALQRCT AQR +F+ CY S
Sbjct  333   DIGEFFQIQDDYIDLYSDESLTGKAGTDIQEGKCSWLAVTALQRCTPAQRALFEQCYASK  392

Query  967   EPAHVERIKRLYEDLHLPQIYKHQEKAMYDNIIRQIENIPIEAARV--LFKKLLDITYNR  1140
              P  V +IK+LYE L+L ++Y  QE+  Y+ I+R+I+ +  + +    LF KL+++ YNR
Sbjct  393   NPEDVAQIKQLYEQLNLQKLYLEQERVKYEEIVRKIDALSRDTSLSPKLFLKLINMIYNR  452

Query  1141  QH*  1149
             + *
Sbjct  453   KR*  455


> ta_transcript80177_1
Length=889

 Score =   358 bits (918),  Expect = 6e-114, Method: Compositional matrix adjust.
 Identities = 183/363 (50%), Positives = 252/363 (69%), Gaps = 5/363 (1%)
 Frame = +1

Query  73    KNNSANMTTASKNLEIINEKKMFDDLLPEVIMTLQNKSKLSEVPQIGDWLKKMLHYNLVG  252
             K     +   + ++E   +++ F D+LP V+ TL +  K SE+P + +WLKK+L YNL G
Sbjct  94    KPAGCRLLATNVSIEHSKDRETFQDVLPLVVDTLISSPKFSEIPDVANWLKKILEYNLAG  153

Query  253   GKHTRGITTVISYKTIEKPEKVTEHTLKMACKLGWCVEMFQAYCIVLDDIMDGSSVRRGM  432
             GK  RG+TTV +Y+ +E PE +T+ ++++A  +GWCVEM QAY IV DD+MDGS+ RRG+
Sbjct  154   GKKARGLTTVFAYEMLETPENITDESMRLARIMGWCVEMLQAYFIVADDMMDGSTTRRGV  213

Query  433   PCWYRRPEVGITCAFNDSLLIHSSLFEFLKTNFRTNPNYMKMFELFNETLWRTSMGQHLD  612
```

```
             PCWYR P+VG+  A ND++LI+SSLFE +  +FR  P Y    +LFNET+  TS GQHLD
Sbjct  214   PCWYRLPDVGLG-AINDAILINSSLFEVINKHFRNYPQYADFLDLFNETILLTSAGQHLD  272

Query  613   HVTGNR--KTDYSSFTLDRyytiikykaayytyNLPVSLGLLLAENVDEKIYKSAQDICL  786
               T     K +YS FT++RY +I+KYK AYYTY LPVSLGLLLA   D+   + + +IC
Sbjct  273   FTTAQHLSKKNYSLFTIERYQSIVKYKTAYYTYRLPVSLGLLLANVTDKPTRQRSDEICF  332

Query  787   EIGTMFQIQDDFIDCFGDEIKTGKVGTDIQERKCTWLAVQALQRCTEAQRTVFKACYGSS  966
             +IG  FQIQDD+ID + DE  TGK GTDIQE KC+WLAV ALQRCT AQR +F+ CY S
Sbjct  333   DIGEFFQIQDDYIDLYSDESLTGKAGTDIQEGKCSWLAVTALQRCTPAQRALFEQCYASK  392

Query  967   EPAHVERIKRLYEDLHLPQIYKHQEKAMYDNIIRQIENIPIEAARV--LFKKLLDITYNR  1140
              P  V +IK+LYE L+L ++Y  QE+  Y+ I+R+I+ +  + +    LF KL+++ YNR
Sbjct  393   NPEDVAQIKQLYEQLNLQKLYLEQERVKYEEIVRKIDALSRDTSLSPKLFLKLINMIYNR  452

Query  1141  QH*  1149
             + *
Sbjct  453   KR*  455

****************************************************************************************************
***********************************************************

Query= gi|153791943:253-1410 Bombyx mori farnesyl diphosphate synthase 3 (Fpps3), mRNA

Length=1158
                                                                      Score     E
Sequences producing significant alignments:                          (Bits)  Value

  ta_transcript80181_1                                                  445    2e-152
  ta_transcript80180_1                                                  445    8e-152
  ta_transcript80179_1                                                  446    3e-150
  ta_transcript80178_1                                                  446    7e-148
  ta_transcript80177_1                                                  446    1e-147


> ta_transcript80181_1
Length=488

 Score =   445 bits (1144),  Expect = 2e-152, Method: Compositional matrix adjust.
 Identities = 217/388 (56%), Positives = 287/388 (74%), Gaps = 10/388 (3%)
 Frame = +1

Query  10    TSMFLRLINRTRQ----IYPIN-SISFQSAGCRCSSAYAASVPQIAKERETFQDAWKGII  174
             TS FLR + +T +    I P++ S   + AGCR   A   S+   +K+RETFQD    ++
Sbjct  67    TSNFLRFMTKTSKFCTNIQPVSVSRVLKPAGCRLL-ATNVSIEH-SKDRETFQDVLPLVV  124

Query  175   DNLTTNKKFTQLPELGSWVKKVLEYNVKGGKKIRGITTVLAYELFEKPENVTEEMVRLAR  354
             D L ++ KF+++P++ +W+KK+LEYN+ GGKK RG+TTV AYE+ E PEN+T+E +RLAR
Sbjct  125   DTLISSPKFSEIPDVANWLKKILEYNLAGGKKARGLTTVFAYEMLETPENITDESMRLAR  184

Query  355   VAGWCTEMLQAYLIMNDDIMDGSSTRRGVPCWYRMPDVGLGAINDAILVYGSIYEILKIY  534
             + GWC EMLQAY I+ DD+MDGS+TRRGVPCWYR+PDVGLGAINDAIL+  S++E++  +
Sbjct  185   IMGWCVEMLQAYFIVADDMMDGSTTRRGVPCWYRLPDVGLGAINDAILINSSLFEVINKH  244

Query  535   FGKAKEYADILDIFNEALLYTSMGQHLDYAMA-HRNKQDYSLFTTERYYSIVKYKTSYYS  711
             F    +YAD LD+FNE +L TS GQHLD+  A H +K++YSLFT ERY SIVKYKT+YY+
Sbjct  245   FRNYPQYADFLDLFNETILLTSAGQHLDFTTAQHLSKKNYSLFTIERYQSIVKYKTAYYT  304

Query  712   IKLPVVLGLILTQNRQNAPIEDIEGICFEIGKLFQIQDDFMDCFGNETVTGKKGTDIQEG  891
              +LPV LGL+L         +  + ICF+IG+ FQIQDD++D + +E++TGK GTDIQEG
Sbjct  305   YRLPVSLGLLLANVTDKPTRQRSDEICFDIGEFFQIQDDYIDLYSDESLTGKAGTDIQEG  364

Query  892   KCSWLAVNALQRCDEAQRKLFAQNYASKNTDDVASIKRLYEDLQLPKLYKEQEDEIHSGI  1071
             KCSWLAV ALQRC  AQR LF Q YASKN +DVA IK+LYE L L KLY EQE   +  I
Sbjct  365   KCSWLAVTALQRCTPAQRALFEQCYASKNPEDVAQIKQLYEQLNLQKLYLEQERVKYEEI  424

Query  1072  LKKINALP--SSTSPAIFFKLLDMIFKR  1149
             ++KI+AL   +S SP +F KL++MI+ R
Sbjct  425   VRKIDALSRDTSLSPKLFLKLINMIYNR  452


> ta_transcript80180_1
Length=536

 Score =   445 bits (1145),  Expect = 8e-152, Method: Compositional matrix adjust.
 Identities = 215/388 (55%), Positives = 287/388 (74%), Gaps = 10/388 (3%)
```

```
 Frame = +1

Query  10    TSMFLRLINRTRQ----IYPIN-SISFQSAGCRCSSAYAASVPQIAKERETFQDAWKGII  174
             TS FLR + +T +    I P++ S   + AGCR  +   +   + +K+RETFQD    ++
Sbjct  67    TSNFLRFMTKTSKFCTNIQPVSVSRVLKPAGCRLLATNVSI--EHSKDRETFQDVLPLVV  124

Query  175   DNLTTNKKFTQLPELGSWVKKVLEYNVKGGKKIRGITTVLAYELFEKPENVTEEMVRLAR  354
             D L ++ KF+++P++ +W+KK+LEYN+ GGKK RG+TTV AYE+ E PEN+T+E +RLAR
Sbjct  125   DTLISSPKFSEIPDVANWLKKILEYNLAGGKKARGLTTVFAYEMLETPENITDESMRLAR  184

Query  355   VAGWCTEMLQAYLIMNDDIMDGSSTRRGVPCWYRMPDVGLGAINDAILVYGSIYEILKIY  534
             + GWC EMLQAY I+ DD+MDGS+TRRGVPCWYR+PDVGLGAINDAIL+  S++E++  +
Sbjct  185   IMGWCVEMLQAYFIVADDMMDGSTTRRGVPCWYRLPDVGLGAINDAILINSSLFEVINKH  244

Query  535   FGKAKEYADILDIFNEALLYTSMGQHLDYAMA-HRNKQDYSLFTTERYYSIVKYKTSYYS  711
             F    +YAD LD+FNE +L TS GQHLD+  A H +K++YSLFT ERY SIVKYKT+YY+
Sbjct  245   FRNYPQYADFLDLFNETILLTSAGQHLDFTTAQHLSKKNYSLFTIERYQSIVKYKTAYYT  304

Query  712   IKLPVVLGLILTQNRQNAPIEDIEGICFEIGKLFQIQDDFMDCFGNETVTGKKGTDIQEG  891
              +LPV LGL+L         +  + ICF+IG+ FQIQDD++D + +E++TGK GTDIQEG
Sbjct  305   YRLPVSLGLLLANVTDKPTRQRSDEICFDIGEFFQIQDDYIDLYSDESLTGKAGTDIQEG  364

Query  892   KCSWLAVNALQRCDEAQRKLFAQNYASKNTDDVASIKRLYEDLQLPKLYKEQEDEIHSGI  1071
             KCSWLAV ALQRC  AQR LF Q YASKN +DVA IK+LYE L L KLY EQE   +  I
Sbjct  365   KCSWLAVTALQRCTPAQRALFEQCYASKNPEDVAQIKQLYEQLNLQKLYLEQERVKYEEI  424

Query  1072  LKKINALP--SSTSPAIFFKLLDMIFKR  1149
             ++KI+AL   +S SP +F KL++MI+ R
Sbjct  425   VRKIDALSRDTSLSPKLFLKLINMIYNR  452


> ta_transcript80179_1
Length=671

 Score =   446 bits (1146),  Expect = 3e-150, Method: Compositional matrix adjust.
 Identities = 215/388 (55%), Positives = 287/388 (74%), Gaps = 10/388 (3%)
 Frame = +1

Query  10    TSMFLRLINRTRQ----IYPIN-SISFQSAGCRCSSAYAASVPQIAKERETFQDAWKGII  174
             TS FLR + +T +    I P++ S   + AGCR  +   +   + +K+RETFQD    ++
Sbjct  67    TSNFLRFMTKTSKFCTNIQPVSVSRVLKPAGCRLLATNVSI--EHSKDRETFQDVLPLVV  124

Query  175   DNLTTNKKFTQLPELGSWVKKVLEYNVKGGKKIRGITTVLAYELFEKPENVTEEMVRLAR  354
             D L ++ KF+++P++ +W+KK+LEYN+ GGKK RG+TTV AYE+ E PEN+T+E +RLAR
Sbjct  125   DTLISSPKFSEIPDVANWLKKILEYNLAGGKKARGLTTVFAYEMLETPENITDESMRLAR  184

Query  355   VAGWCTEMLQAYLIMNDDIMDGSSTRRGVPCWYRMPDVGLGAINDAILVYGSIYEILKIY  534
             + GWC EMLQAY I+ DD+MDGS+TRRGVPCWYR+PDVGLGAINDAIL+  S++E++  +
Sbjct  185   IMGWCVEMLQAYFIVADDMMDGSTTRRGVPCWYRLPDVGLGAINDAILINSSLFEVINKH  244

Query  535   FGKAKEYADILDIFNEALLYTSMGQHLDYAMA-HRNKQDYSLFTTERYYSIVKYKTSYYS  711
             F    +YAD LD+FNE +L TS GQHLD+  A H +K++YSLFT ERY SIVKYKT+YY+
Sbjct  245   FRNYPQYADFLDLFNETILLTSAGQHLDFTTAQHLSKKNYSLFTIERYQSIVKYKTAYYT  304

Query  712   IKLPVVLGLILTQNRQNAPIEDIEGICFEIGKLFQIQDDFMDCFGNETVTGKKGTDIQEG  891
              +LPV LGL+L         +  + ICF+IG+ FQIQDD++D + +E++TGK GTDIQEG
Sbjct  305   YRLPVSLGLLLANVTDKPTRQRSDEICFDIGEFFQIQDDYIDLYSDESLTGKAGTDIQEG  364

Query  892   KCSWLAVNALQRCDEAQRKLFAQNYASKNTDDVASIKRLYEDLQLPKLYKEQEDEIHSGI  1071
             KCSWLAV ALQRC  AQR LF Q YASKN +DVA IK+LYE L L KLY EQE   +  I
Sbjct  365   KCSWLAVTALQRCTPAQRALFEQCYASKNPEDVAQIKQLYEQLNLQKLYLEQERVKYEEI  424

Query  1072  LKKINALP--SSTSPAIFFKLLDMIFKR  1149
             ++KI+AL   +S SP +F KL++MI+ R
Sbjct  425   VRKIDALSRDTSLSPKLFLKLINMIYNR  452


> ta_transcript80178_1
Length=883

 Score =   446 bits (1147),  Expect = 7e-148, Method: Compositional matrix adjust.
 Identities = 217/388 (56%), Positives = 287/388 (74%), Gaps = 10/388 (3%)
 Frame = +1

Query  10    TSMFLRLINRTRQ----IYPIN-SISFQSAGCRCSSAYAASVPQIAKERETFQDAWKGII  174
             TS FLR + +T +    I P++ S   + AGCR   A   S+   +K+RETFQD    ++
Sbjct  67    TSNFLRFMTKTSKFCTNIQPVSVSRVLKPAGCRLL-ATNVSIEH-SKDRETFQDVLPLVV  124
```

```
Query  175    DNLTTNKKFTQLPELGSWVKKVLEYNVKGGKKIRGITTVLAYELFEKPENVTEEMVRLAR  354
              D L ++ KF+++P++ +W+KK+LEYN+ GGKK RG+TTV AYE+ E PEN+T+E +RLAR
Sbjct  125    DTLISSPKFSEIPDVANWLKKILEYNLAGGKKARGLTTVFAYEMLETPENITDESMRLAR  184

Query  355    VAGWCTEMLQAYLIMNDDIMDGSSTRRGVPCWYRMPDVGLGAINDAILVYGSIYEILKIY  534
              + GWC EMLQAY I+ DD+MDGS+TRRGVPCWYR+PDVGLGAINDAIL+  S++E++  +
Sbjct  185    IMGWCVEMLQAYFIVADDMMDGSTTRRGVPCWYRLPDVGLGAINDAILINSSLFEVINKH  244

Query  535    FGKAKEYADILDIFNEALLYTSMGQHLDYAMA-HRNKQDYSLFTTERYYSIVKYKTSYYS  711
              F    +YAD LD+FNE +L TS GQHLD+  A H +K++YSLFT ERY SIVKYKT+YY+
Sbjct  245    FRNYPQYADFLDLFNETILLTSAGQHLDFTTAQHLSKKNYSLFTIERYQSIVKYKTAYYT  304

Query  712    IKLPVVLGLILTQNRQNAPIEDIEGICFEIGKLFQIQDDFMDCFGNETVTGKKGTDIQEG  891
               +LPV LGL+L         +  + ICF+IG+ FQIQDD++D + +E++TGK GTDIQEG
Sbjct  305    YRLPVSLGLLLANVTDKPTRQRSDEICFDIGEFFQIQDDYIDLYSDESLTGKAGTDIQEG  364

Query  892    KCSWLAVNALQRCDEAQRKLFAQNYASKNTDDVASIKRLYEDLQLPKLYKEQEDEIHSGI  1071
              KCSWLAV ALQRC  AQR LF Q YASKN +DVA IK+LYE L L KLY EQE   +  I
Sbjct  365    KCSWLAVTALQRCTPAQRALFEQCYASKNPEDVAQIKQLYEQLNLQKLYLEQERVKYEEI  424

Query  1072  LKKINALP--SSTSPAIFFKLLDMIFKR  1149
              ++KI+AL   +S SP +F KL++MI+ R
Sbjct  425    VRKIDALSRDTSLSPKLFLKLINMIYNR  452


> ta_transcript80177_1
Length=889

 Score =   446 bits (1146),  Expect = 1e-147, Method: Compositional matrix adjust.
 Identities = 217/388 (56%), Positives = 287/388 (74%), Gaps = 10/388 (3%)
 Frame = +1

Query  10     TSMFLRLINRTRQ----IYPIN-SISFQSAGCRCSSAYAASVPQIAKERETFQDAWKGII  174
              TS FLR + +T +    I P++ S   + AGCR   A   S+   +K+RETFQD    ++
Sbjct  67     TSNFLRFMTKTSKFCTNIQPVSVSRVLKPAGCRLL-ATNVSIEH-SKDRETFQDVLPLVV  124

Query  175    DNLTTNKKFTQLPELGSWVKKVLEYNVKGGKKIRGITTVLAYELFEKPENVTEEMVRLAR  354
              D L ++ KF+++P++ +W+KK+LEYN+ GGKK RG+TTV AYE+ E PEN+T+E +RLAR
Sbjct  125    DTLISSPKFSEIPDVANWLKKILEYNLAGGKKARGLTTVFAYEMLETPENITDESMRLAR  184

Query  355    VAGWCTEMLQAYLIMNDDIMDGSSTRRGVPCWYRMPDVGLGAINDAILVYGSIYEILKIY  534
              + GWC EMLQAY I+ DD+MDGS+TRRGVPCWYR+PDVGLGAINDAIL+  S++E++  +
Sbjct  185    IMGWCVEMLQAYFIVADDMMDGSTTRRGVPCWYRLPDVGLGAINDAILINSSLFEVINKH  244

Query  535    FGKAKEYADILDIFNEALLYTSMGQHLDYAMA-HRNKQDYSLFTTERYYSIVKYKTSYYS  711
              F    +YAD LD+FNE +L TS GQHLD+  A H +K++YSLFT ERY SIVKYKT+YY+
Sbjct  245    FRNYPQYADFLDLFNETILLTSAGQHLDFTTAQHLSKKNYSLFTIERYQSIVKYKTAYYT  304

Query  712    IKLPVVLGLILTQNRQNAPIEDIEGICFEIGKLFQIQDDFMDCFGNETVTGKKGTDIQEG  891
               +LPV LGL+L         +  + ICF+IG+ FQIQDD++D + +E++TGK GTDIQEG
Sbjct  305    YRLPVSLGLLLANVTDKPTRQRSDEICFDIGEFFQIQDDYIDLYSDESLTGKAGTDIQEG  364

Query  892    KCSWLAVNALQRCDEAQRKLFAQNYASKNTDDVASIKRLYEDLQLPKLYKEQEDEIHSGI  1071
              KCSWLAV ALQRC  AQR LF Q YASKN +DVA IK+LYE L L KLY EQE   +  I
Sbjct  365    KCSWLAVTALQRCTPAQRALFEQCYASKNPEDVAQIKQLYEQLNLQKLYLEQERVKYEEI  424

Query  1072  LKKINALP--SSTSPAIFFKLLDMIFKR  1149
              ++KI+AL   +S SP +F KL++MI+ R
Sbjct  425    VRKIDALSRDTSLSPKLFLKLINMIYNR  452

************************************************************************************************
**********************************************************


Query= gi|112982769:125-961 Bombyx mori juvenile hormone acid methyltransferase (Jhamt), mRNA

Length=837
                                                                      Score     E
Sequences producing significant alignments:                          (Bits)  Value

  ta_transcript79838_1                                                  332    8e-109
  ta_transcript79836_1                                                  333    1e-108
  ta_transcript79837_1                                                  332    1e-108
  ta_transcript79835_1                                                  332    3e-108
```

```
> ta_transcript79838_1
Length=604

 Score =   332 bits (852),  Expect = 8e-109, Method: Compositional matrix adjust.
 Identities = 150/273 (55%), Positives = 212/273 (78%), Gaps = 4/273 (1%)
 Frame = +1

Query  1    MNNADLYRKSNSLQKRDALRCLEEHANKIKWKKIGDRVIDLGCADGSVT-DILKVYMPKN  177
            MN+A+LY++SN LQKRDAL+CL+E+  K++WK   +RV+D+GC DG VT +IL+ ++P++
Sbjct  138  MNDAELYQQSNKLQKRDALQCLKEYEKKLRWKS-SERVLDIGCGDGGVTTEILRQFIPED  196

Query  178  YGRLVGCDISEEMVKYANKHHGFGRTSFRVLDIEGDLTADLKQGFDHVFSFYTLHWIRDQ  357
            +  L GCDISE+MV +AN HHG  R  F VLDIEGDL AD  +GFDH +SFYTLHWI DQ
Sbjct  197  FAALTGCDISEKMVHFANAHHGDHRIQFVVLDIEGDLPADFVRGFDHAYSFYTLHWIHDQ  256

Query  358  ERAFRNIFNLLGDEGDCLLLFLGHTPIFDVYRTLSHTEKWHSWLEHVDRFISPYHDNEDP  537
            +RAF+NI++LL D+G+CLL+FLGH P+FDV+R+L+   KW  WL  VDRFISPYHD++DP
Sbjct  257  DRAFKNIYDLLADDGECLLIFLGHMPLFDVFRSLARWPKWREWLRDVDRFISPYHDSQDP  316

Query  538  EKEVKKIMERVGFSNIEVQCKTLFYVYDDLDVLKKSVAAINPFNIPKDILEDFLEDYIDV  717
            EK++K++M + GF NI V+ K   ++Y+ L   K +V+A+NPF + + + E+FL+DY+ +
Sbjct  317  EKDIKRLMAKTGFRNITVKIKEKSFIYNSLQDCKNAVSAVNPFKMSRSVEEEFLQDYMQL  376

Query  718  VREMRLLDRCNNNVGESVSIKFNYKVISVYARK  816
            VR+MRL+D+ NNN+ E  +IK +Y +I  Y RK
Sbjct  377  VRQMRLIDQVNNNLDE--TIKTDYTLIVAYGRK  407


> ta_transcript79836_1
Length=630

 Score =   333 bits (853),  Expect = 1e-108, Method: Compositional matrix adjust.
 Identities = 150/273 (55%), Positives = 212/273 (78%), Gaps = 4/273 (1%)
 Frame = +1

Query  1    MNNADLYRKSNSLQKRDALRCLEEHANKIKWKKIGDRVIDLGCADGSVT-DILKVYMPKN  177
            MN+A+LY++SN LQKRDAL+CL+E+  K++WK   +RV+D+GC DG VT +IL+ ++P++
Sbjct  138  MNDAELYQQSNKLQKRDALQCLKEYEKKLRWKS-SERVLDIGCGDGGVTTEILRQFIPED  196

Query  178  YGRLVGCDISEEMVKYANKHHGFGRTSFRVLDIEGDLTADLKQGFDHVFSFYTLHWIRDQ  357
            +  L GCDISE+MV +AN HHG  R  F VLDIEGDL AD  +GFDH +SFYTLHWI DQ
Sbjct  197  FAALTGCDISEKMVHFANAHHGDHRIQFVVLDIEGDLPADFVRGFDHAYSFYTLHWIHDQ  256

Query  358  ERAFRNIFNLLGDEGDCLLLFLGHTPIFDVYRTLSHTEKWHSWLEHVDRFISPYHDNEDP  537
            +RAF+NI++LL D+G+CLL+FLGH P+FDV+R+L+   KW  WL  VDRFISPYHD++DP
Sbjct  257  DRAFKNIYDLLADDGECLLIFLGHMPLFDVFRSLARWPKWREWLRDVDRFISPYHDSQDP  316

Query  538  EKEVKKIMERVGFSNIEVQCKTLFYVYDDLDVLKKSVAAINPFNIPKDILEDFLEDYIDV  717
            EK++K++M + GF NI V+ K   ++Y+ L   K +V+A+NPF + + + E+FL+DY+ +
Sbjct  317  EKDIKRLMAKTGFRNITVKIKEKSFIYNSLQDCKNAVSAVNPFKMSRSVEEEFLQDYMQL  376

Query  718  VREMRLLDRCNNNVGESVSIKFNYKVISVYARK  816
            VR+MRL+D+ NNN+ E  +IK +Y +I  Y RK
Sbjct  377  VRQMRLIDQVNNNLDE--TIKTDYTLIVAYGRK  407


> ta_transcript79837_1
Length=622

 Score =   332 bits (852),  Expect = 1e-108, Method: Compositional matrix adjust.
 Identities = 150/273 (55%), Positives = 212/273 (78%), Gaps = 4/273 (1%)
 Frame = +1

Query  1    MNNADLYRKSNSLQKRDALRCLEEHANKIKWKKIGDRVIDLGCADGSVT-DILKVYMPKN  177
            MN+A+LY++SN LQKRDAL+CL+E+  K++WK   +RV+D+GC DG VT +IL+ ++P++
Sbjct  138  MNDAELYQQSNKLQKRDALQCLKEYEKKLRWKS-SERVLDIGCGDGGVTTEILRQFIPED  196

Query  178  YGRLVGCDISEEMVKYANKHHGFGRTSFRVLDIEGDLTADLKQGFDHVFSFYTLHWIRDQ  357
            +  L GCDISE+MV +AN HHG  R  F VLDIEGDL AD  +GFDH +SFYTLHWI DQ
Sbjct  197  FAALTGCDISEKMVHFANAHHGDHRIQFVVLDIEGDLPADFVRGFDHAYSFYTLHWIHDQ  256

Query  358  ERAFRNIFNLLGDEGDCLLLFLGHTPIFDVYRTLSHTEKWHSWLEHVDRFISPYHDNEDP  537
            +RAF+NI++LL D+G+CLL+FLGH P+FDV+R+L+   KW  WL  VDRFISPYHD++DP
Sbjct  257  DRAFKNIYDLLADDGECLLIFLGHMPLFDVFRSLARWPKWREWLRDVDRFISPYHDSQDP  316

Query  538  EKEVKKIMERVGFSNIEVQCKTLFYVYDDLDVLKKSVAAINPFNIPKDILEDFLEDYIDV  717
            EK++K++M + GF NI V+ K   ++Y+ L   K +V+A+NPF + + + E+FL+DY+ +
```

```
Sbjct  317  EKDIKRLMAKTGFRNITVKIKEKSFIYNSLQDCKNAVSAVNPFKMSRSVEEEFLQDYMQL  376

Query  718  VREMRLLDRCNNNVGESVSIKFNYKVISVYARK  816
            VR+MRL+D+ NNN+ E  +IK +Y +I  Y RK
Sbjct  377  VRQMRLIDQVNNNLDE--TIKTDYTLIVAYGRK  407


> ta_transcript79835_1
Length=653

 Score =   332 bits (852),  Expect = 3e-108, Method: Compositional matrix adjust.
 Identities = 150/273 (55%), Positives = 212/273 (78%), Gaps = 4/273 (1%)
 Frame = +1

Query  1    MNNADLYRKSNSLQKRDALRCLEEHANKIKWKKIGDRVIDLGCADGSVT-DILKVYMPKN  177
            MN+A+LY++SN LQKRDAL+CL+E+   K++WK   +RV+D+GC DG VT +IL+ ++P++
Sbjct  138  MNDAELYQQSNKLQKRDALQCLKEYEKKLRWKS-SERVLDIGCGDGGVTTEILRQFIPED  196

Query  178  YGRLVGCDISEEMVKYANKHHGFGRTSFRVLDIEGDLTADLKQGFDHVFSFYTLHWIRDQ  357
            +  L GCDISE+MV +AN HHG  R  F VLDIEGDL AD  +GFDH +SFYTLHWI DQ
Sbjct  197  FAALTGCDISEKMVHFANAHHGDHRIQFVVLDIEGDLPADFVRGFDHAYSFYTLHWIHDQ  256

Query  358  ERAFRNIFNLLGDEGDCLLLFLGHTPIFDVYRTLSHTEKWHSWLEHVDRFISPYHDNEDP  537
            +RAF+NI++LL D+G+CLL+FLGH P+FDV+R+L+   KW  WL  VDRFISPYHD++DP
Sbjct  257  DRAFKNIYDLLADDGECLLIFLGHMPLFDVFRSLARWPKWREWLRDVDRFISPYHDSQDP  316

Query  538  EKEVKKIMERVGFSNIEVQCKTLFYVYDDLDVLKKSVAAINPFNIPKDILEDFLEDYIDV  717
            EK++K++M + GF NI V+ K    ++Y+ L    K +V+A+NPF + + + E+FL+DY+ +
Sbjct  317  EKDIKRLMAKTGFRNITVKIKEKSFIYNSLQDCKNAVSAVNPFKMSRSVEEEFLQDYMQL  376

Query  718  VREMRLLDRCNNNVGESVSIKFNYKVISVYARK  816
            VR+MRL+D+ NNN+ E  +IK +Y +I  Y RK
Sbjct  377  VRQMRLIDQVNNNLDE--TIKTDYTLIVAYGRK  407

*************************************************************************************************
******************************************************************
Query= gi|226502343:35-1516 Bombyx mori cytochrome P450 (P450), mRNA

Length=1482
                                                                      Score     E
Sequences producing significant alignments:                          (Bits)  Value

  ta_transcript44033_1                                                  306   3e-94
  ta_transcript62517_1                                                  249   5e-73
  ta_transcript44034_1                                                  174   5e-47
  ta_transcript44035_1                                                  144   2e-37


> ta_transcript44033_1
Length=709

 Score =   306 bits (783),  Expect = 3e-94, Method: Compositional matrix adjust.
 Identities = 237/456 (52%), Positives = 272/456 (60%), Gaps = 2/456 (0%)
 Frame = -2

Query  1460  LGSKIILNCLADNESPSMGSVDGNGSFLGILRWNF*RI*VRNMKSSERANPSPIQRRFPI  1281
             LG    LN  A  E+PSM SV+G+GS  GIL WNF RI*VRNMKSS  A  SP+QR  PI
Sbjct  215   LGWNTTLNGFAAKETPSMTSVEGSGSPGGILTWNFWRI*VRNMKSSCLARFSPMQRLLPI  274

Query  1280  PKGMSHSSCKMLPSLVRNRSGRNVSGSFQCGP*CSSYSDNKIKVPFGII*FPSLASLRAI  1101
             PKG+SHSS      SLVRNRSG  V GSFQ  P C S   ++IKV  GI+* PSLAS RAI
Sbjct  275   PKGISHSS*IRFMSLVRNRSGLKV*GSFQWLPLCKSCMASRIKVSLGIM*LPSLASSRAI  334

Query  1100  *GMPIDATVDILRVSRITASVYTililsssk*lrsRPIMSSISWCSFLCTSSFLTTCRSI  921
              G+P    T D+ RVS ITASVYTIL+LS S     S P+ SSIS C    CT  FLT C SI
Sbjct  335   WGIPTAGTEDMRRVSNITASVYTILVLSLSCGGLS*PMTSSISCCRRNCT*WFLTRCNSI  394

Query  920   NTAVLVTVSMPAssrsrqttcrsssvslss*dssismrk--aSITSFGAPSCKFSWCSFI  747
              TAVL+TVS PA SRS  TT  SS V L++       +    ASITS G  + + +WCS I
Sbjct  395   KTAVLLTVSTPAWSRSMHTTSSSSWVILNASSLLFLISMRNASITSRGFLTSRVAWCSSI  454

Query  746   ISRKYW*RALCIS*SSVNPISLGTKCLRNGRKLRIPPLISTSLNKRITRLHSFLSRWSSK  567
              S +  *R    S  SV P+  G   LR G K R+PPL+S +       R HS  SR SSK
Sbjct  455   TSLRNV*RES*TSLISV*PMRPGMMYLRKGMKFRMPPLMSRTAKALFIRWHSSFSRSSSK  514
```

```
Query  566   SYLFPATNLQSMLTIVMWNI*LTRIGSPASAKRSCTRASHSSPM*TFMKREL*PKFFKYF  387
             SYL PAT    M  I   N+*LT IG PAS   S T+A HSS M   M+RE *PK FKY
Sbjct  515   SYLLPATIRHKMFIIDTSNM*LTGIGWPASFVLSSTKALHSSAMFASMEREP*PKVFKYL  574

Query  386   STKRLVLCHVGPSEKTNPSFFPNDLMV*KNPSGLPSNTSRFETSLMSSIPVTTTTLMDPS  207
              T   V  H GPSEKT P+F+P D + *K P GLPS TS  ETS + S+P TTTT   PS
Sbjct  575   MTTLRVALHEGPSEKTIPNFWPKDRIT*KKPPGLPSKTSLVETSFIISLPETTTTSQSPS  634

Query  206   LKPNRFPYF*DHDCHIKW*HLNFLDSQSTESKFPTI  99
               PN  PY         W*+ +      T +K P I
Sbjct  635   FNPNTSPYCWAQAAQTLW*YFSSFSLNQTATKDPNI  670


> ta_transcript62517_1
Length=712

 Score =   249 bits (635),  Expect = 5e-73, Method: Compositional matrix adjust.
 Identities = 167/491 (34%), Positives = 264/491 (54%), Gaps = 18/491 (4%)
 Frame = +1

Query  4     LALIVLCFILFFYIISRRHRGLCYPPGPTPLPIVGNLLSVLWESRKFKCHHLIWQSWSQ-  180
             + L+V  FI    I+    +   YPPGP   P +G    V   S K   H   WQ+ SQ
Sbjct  44    ITLLVFAFIAL--IVKSLIKPNNYPPGPIWYPFIGCSTEVKNMSNK---HGSQWQALSQL  98

Query  181   --KYGN-LLGLRLGSINVVVVTGIELIREVSNREVFEGRPDGFFYTMRSFGKKLGLVFSD  351
               +Y   +LGL+LGS  VVVV G + IR++     FEGRP+ FF  +R  GK+LG+ F+D
Sbjct  99    AKQYSTQVLGLKLGSELVVVVYGEKNIRQIFTGLEFEGRPNSFFIRLRCLGKRLGITFTD  158

Query  352   GPTWHRTRRFVLKYLKNFGYNSRFMNVYIGEECEALVQLRLADAGEPILVNQMFHITIVN  531
             GP W   R F +K L+N G+    M   I  E   ++   + +   PI   ++   +++N
Sbjct  159   GPLWREHRCFTVKQLRNVGFGKSAMEKEIQGELRNILNY-IKNNENPISPRKILAKSVMN  217

Query  532   ILWRLVAGKRYDLEDQRLKKLCSLVMRLFKLVDMSGGILNFLPFLRHFVPRLIGFTELQE  711
             +LW+ VAG+R  +E++RL  L  L+    K   M+GG LN  P+ R  +P + G++ +++
Sbjct  218   VLWKFVAGER--IEEERLNSLLDLLNARSKAFSMAGGWLNQFPWSRFILPDISGYSLIKK  275

Query  712   IHNALHQYLREIIKEHQENLQLGAPKDVIDAFLIDMLESQDDKltlddlqvvcldllEAG  891
             ++  +   + E I++H+     G+  D I +F I+ +       T   L+ +CLDLL AG
Sbjct  276   MNEEISNIIEEAIQKHKSQSVEGS--DYIYSF-IEEINLNKTSFTEQQLKTICLDLLIAG  332

Query  892   METVTNTAVFMLLHVVRNEDVQRKLHQEIDDIIGRDRNHLLDDRIRMVYTEAVILETLRI  1071
              +T +N   F LL  +R++ +Q+K+H EI  +IG +     D+  R+ YT A +LE  R
Sbjct  333   SQTTSNALEFALLAALRHKHIQQKIHDEISKVIGNNIPCWADN-YRLTYTSAFLLEVQRF  391

Query  1072  STVASMGIPHMALNDAKLGNYIIPKGTFILLSLYELHHGPH-WKDPETFRPERFLTKEGN  1248
              T+  +  P   L    +  Y IPK T IL++  +LH  P  W +P  F+PERF+   G
Sbjct  392   YTIVPIAGPRRVLETTTIEGYTIPKETTILIATGDLHCDPELWDEPHIFKPERFIDANGT  451

Query  1249  ILQDEWLIPFGIGKRRCIGEGLARSELFMFLTHILQKFHLRIPKNEPLPSTEPIDGLSLS  1428
             +   E + PFG+G+RRC G+ LA+S +F+    I+QK+ +   +N   PS   P+ GL  +
Sbjct  452   LRSVEHMYPFGLGRRRCPGDSLAKSFIFITFVGIMQKYFIEC-RNGTYPSNNPVIGLIAA  510

Query  1429  AKQFRIIFEPR  1461
              + +   F P+
Sbjct  511   PEAYTADFIPK  521


> ta_transcript44034_1
Length=515

 Score =   174 bits (440),  Expect = 5e-47, Method: Compositional matrix adjust.
 Identities = 117/192 (61%), Positives = 130/192 (68%), Gaps = 0/192 (0%)
 Frame = -2

Query  1460  LGSKIILNCLADNESPSMGSVDGNGSFLGILRWNF*RI*VRNMKSSERANPSPIQRRFPI  1281
             LG    LN  A  E+PSM SV+G+GS  GIL WNF RI*VRNMKSS  A  SP+QR  PI
Sbjct  215   LGWNTTLNGFAAKETPSMTSVEGSGSPGGILTWNFWRI*VRNMKSSCLARFSPMQRLLPI  274

Query  1280  PKGMSHSSCKMLPSLVRNRSGRNVSGSFQCGP*CSSYSDNKIKVPFGII*FPSLASLRAI  1101
             PKG+SHSS     SLVRNRSG  V GSFQ  P C S    ++IKV  GI+* PSLAS RAI
Sbjct  275   PKGISHSS*IRFMSLVRNRSGLKV*GSFQWLPLCKSCMASRIKVSLGIM*LPSLASSRAI  334

Query  1100  *GMPIDATVDILRVSRITASVYTililsssk*lrsRPIMSSISWCSFLCTSSFLTTCRSI  921
              G+P   T D+ RVS ITASVYTIL+LS S    S P+ SSIS C   CT  FLT C SI
Sbjct  335   WGIPTAGTEDMRRVSNITASVYTILVLSLSCGGLS*PMTSSISCCRRNCT*WFLTRCNSI  394

Query  920   NTAVLVTVSMPA  885
```

```
              TAVL+TVS PA
Sbjct  395   KTAVLLTVSTPA  406


> ta_transcript44035_1
Length=373

 Score =   144 bits (363),  Expect = 2e-37, Method: Compositional matrix adjust.
 Identities = 88/143 (62%), Positives = 98/143 (69%), Gaps = 0/143 (0%)
 Frame = -2

Query  1460  LGSKIILNCLADNESPSMGSVDGNGSFLGILRWNF*RI*VRNMKSSERANPSPIQRRFPI  1281
             LG    LN  A  E+PSM SV+G+GS  GIL WNF RI*VRNMKSS  A  SP+QR  PI
Sbjct  215   LGWNTTLNGFAAKETPSMTSVEGSGSPGGILTWNFWRI*VRNMKSSCLARFSPMQRLLPI  274

Query  1280  PKGMSHSSCKMLPSLVRNRSGRNVSGSFQCGP*CSSYSDNKIKVPFGII*FPSLASLRAI  1101
             PKG+SHSS     SLVRNRSG  V GSFQ  P C S   ++IKV  GI+* PSLAS RAI
Sbjct  275   PKGISHSS*IRFMSLVRNRSGLKV*GSFQWLPLCKSCMASRIKVSLGIM*LPSLASSRAI  334

Query  1100  *GMPIDATVDILRVSRITASVYT  1032
              G+P   T D+ RVS ITASVYT
Sbjct  335   WGIPTAGTEDMRRVSNITASVYT  357


****************************************************************************************************
*******************************************************************
Query= gi|261245094:276-1658 Bombyx mori juvenile hormone epoxide hydrolase-like protein 1
(Jheh-lp1), mRNA

Length=1383
                                                                      Score     E
Sequences producing significant alignments:                          (Bits)  Value

  ta_transcript53251_1                                                  399   1e-131
  ta_transcript53250_1                                                  400   2e-131
  ta_transcript29071_1                                                  315   4e-100
  ta_transcript45224_1                                                  309   3e-97
  ta_transcript58145_1                                                  245   2e-75
  ta_transcript58144_1                                                  245   2e-74
  ta_transcript35275_1                                                  138   2e-34


> ta_transcript53251_1
Length=624

 Score =   399 bits (1026),  Expect = 1e-131, Method: Compositional matrix adjust.
 Identities = 201/452 (44%), Positives = 281/452 (62%), Gaps = 3/452 (1%)
 Frame = +1

Query  13    LIATFGLASGLAITYYLFKSPNPPELDLQKWWGSGSPVAVVDTSIRPFKIEFNYTMIKDL  192
               AT      + I + L ++P  PELDL  WWG  S     D S+RP K++F   MI+DL
Sbjct  104   FFATITAVVAVVIYFALLRAPPLPELDLNAWWGPDSLKTKQDDSVRPMKLKFKKPMIQDL  163

Query  193   KERLHNRRTFTKPLQGIQSEYGINTIYLETVLDYWVEDYDFKKRADLLNMFPHYKTNIQG  372
             ++ L   R F  PL+G+  EYG N+  +++ L YW E+Y+F++R    N +  Y+T IQG
Sbjct  164   QQYLKTTRKFAPPLEGVGFEYGFNSNNMDSWLKYWAEEYNFEERERFFNQYESYRTLIQG  223

Query  373   LDIHFIRVKPDV-EDVEVLPLLMLHGWPSSSKEFDKVIPMLTRPRVGYNFVFEVIAADLP  549
             L+IHFI VKP V   VEV+P+L+LHGWP S +EF + IP+LT      NF  E+I   LP
Sbjct  224   LNIHFIHVKPQVPAGVEVVPMLLLHGWPGSVREFYEAIPLLTAVDKTRNFALELIIPSLP  283

Query  550   GYGFSEGTNKPGLNPVQIGVIMRNLMMRLGFEKFYIQAGDWGSQCATHMATLFPEQVLGL  729
             GYG+S+   +PGL   ++ V+M+NLM RLG++++Y+Q GDWG+   T ++TLFP +VLG
Sbjct  284   GYGWSDAAVRPGLGAAEVAVVMKNLMNRLGYKQYYLQGGDWGAVICTALSTLFPNEVLGY  343

Query  730   HTNMPLSSKPVSTLKLILGALVPRLAVDRKYADRIYPLKNLFSYLLRESGYFHIQATKPD  909
             HTNM  +  P +T    L +L P LA++ +  DR YPL   +S+L+ E GY HIQATKPD
Sbjct  344   HTNMLFNMSPAATALEWLFSLWPTLAIEPELVDRAYPLSKTYSHLMEEMGYMHIQATKPD  403

Query  910   TIGVALTDSPSGLAAYIIEKMAICSSRIELDTPHGGL-QHldlddvldtvtITWMNNCIV  1086
             T+GVAL+DSP+GL AYI+EK +  + R  + +  GGL +H   + ++D + + W    I
Sbjct  404   TVGVALSDSPAGLLAYILEKFSTWTDRNLISSKDGGLTKHFTKEQLVDNLMVYWSTQSIT  463

Query  1087  TSMRLYAEGFALPEVQTVHD-IPTYVPTAAINFLYEVIYQPDWILRDKFKNLVRSTVIES  1263
             TSMRLYAE F    +    D IPT VP   I   +E+ YQP  ILR KF NL+ +TV+E
Sbjct  464   TSMRLYAESFNKRHLALELDSIPTTVPVWGIQAKHEISYQPPVILRLKFPNLLHTTVLEE  523
```

```
Query  1264  GGHFAAMQTPNLLTDDIFDSAVEFLKFHEKNK  1359
             GGHF A Q P +  +D+      F  +H+  K
Sbjct  524   GGHFFAFQLPKIFAEDVLKGVSAFRDWHKAKK  555


> ta_transcript53250_1
Length=647

 Score =   400 bits (1027),  Expect = 2e-131, Method: Compositional matrix adjust.
 Identities = 201/450 (45%), Positives = 281/450 (62%), Gaps = 3/450 (1%)
 Frame = +1

Query  19    ATFGLASGLAITYYLFKSPNPPELDLQKWWGSGSPVAVVDTSIRPFKIEFNYTMIKDLKE  198
             AT       + I + L ++P  PELDL  WWG  S      D S+RP K++F   MI+DL++
Sbjct  106   ATITAVVAVVIYFALLRAPPLPELDLNAWWGPDSLKTKQDDSVRPMKLKFKKPMIQDLQQ  165

Query  199   RLHNRRTFTKPLQGIQSEYGINTIYLETVLDYWVEDYDFKKRADLLNMFPHYKTNIQGLD  378
              L   R F  PL+G+  EYG N+  +++ L YW E+Y+F++R    N +  Y+T IQGL+
Sbjct  166   YLKTTRKFAPPLEGVGFEYGFNSNNMDSWLKYWAEEYNFEERERFFNQYESYRTLIQGLN  225

Query  379   IHFIRVKPDV-EDVEVLPLLMLHGWPSSSKEFDKVIPMLTRPRVGYNFVFEVIAADLPGY  555
             IHFI VKP V   VEV+P+L+LHGWP S +EF + IP+LT       NF  E+I   LPGY
Sbjct  226   IHFIHVKPQVPAGVEVVPMLLLHGWPGSVREFYEAIPLLTAVDKTRNFALELIIPSLPGY  285

Query  556   GFSEGTNKPGLNPVQIGVIMRNLMMRLGFEKFYIQAGDWGSQCATHMATLFPEQVLGLHT  735
             G+S+    +PGL   ++ V+M+NLM RLG++++Y+Q GDWG+   T ++TLFP +VLG HT
Sbjct  286   GWSDAAVRPGLGAAEVAVVMKNLMNRLGYKQYYLQGGDWGAVICTALSTLFPNEVLGYHT  345

Query  736   NMPLSSKPVSTLKLILGALVPRLAVDRKYADRIYPLKNLFSYLLRESGYFHIQATKPDTI  915
             NM  +  P +T    L +L P LA++ +  DR YPL   +S+L+ E GY HIQATKPDT+
Sbjct  346   NMLFNMSPAATALEWLFSLWPTLAIEPELVDRAYPLSKTYSHLMEEMGYMHIQATKPDTV  405

Query  916   GVALTDSPSGLAAYIIEKMAICSSRIELDTPHGGL-QHldlddvldtvtITWMNNCIVTS  1092
             GVAL+DSP+GL AYI+EK +   + R  + +  GGL +H   + ++D + + W    I TS
Sbjct  406   GVALSDSPAGLLAYILEKFSTWTDRNLISSKDGGLTKHFTKEQLVDNLMVYWSTQSITTS  465

Query  1093  MRLYAEGFALPEVQTVHD-IPTYVPTAAINFLYEVIYQPDWILRDKFKNLVRSTVIESGG  1269
             MRLYAE F    +    D IPT VP   I   +E+ YQP  ILR KF NL+ +TV+E GG
Sbjct  466   MRLYAESFNKRHLALELDSIPTTVPVWGIQAKHEISYQPPVILRLKFPNLLHTTVLEEGG  525

Query  1270  HFAAMQTPNLLTDDIFDSAVEFLKFHEKNK  1359
             HF A Q P +  +D+      F  +H+  K
Sbjct  526   HFFAFQLPKIFAEDVLKGVSAFRDWHKAKK  555


> ta_transcript29071_1
Length=520

 Score =   315 bits (806),  Expect = 4e-100, Method: Compositional matrix adjust.
 Identities = 183/428 (43%), Positives = 251/428 (59%), Gaps = 28/428 (7%)
 Frame = +1

Query  91    DLQKWWGSGSPVAVVDTSIRPFKIEFNYTMIKDLKERLHNRRTFTKPLQGIQSEYGINTI  270
             D+Q+WWG  S      DTSIRP KI F   MIKDL+ RL    +F  PL+      YG N+
Sbjct  43    DIQEWWGPKSLQGHEDTSIRPAKIHFENKMIKDLQGRLRKTISFQPPLEDSGFTYGFNSD  102

Query  271   YLETVLDYWVEDYDFKKRADLLNMFPHYKTNIQGLDIHFIRVKPDVEDVE-VLPLLMLHG  447
              +   L YW E+Y FK+R   LN FP +KTNIQGLDIHFI VKP V+   + V+PLL+LHG
Sbjct  103   SIGYWLKYWAEEYPFKEREAYLNQFPQFKTNIQGLDIHFIHVKPKVQTHKNVIPLLLLHG  162

Query  448   WPSSSKEFDKVIPMLTRPRVGYNFVFEVIAADLPGYGFSEGTNKPGLNPVQIGVIMRNLM  627
             W  S +EF   IP+LT       +F  E+IA  LPG+GFS+ T + GL   Q+ +IMRNLM
Sbjct  163   WAGSIREFYDAIPLLTADSKDRDFAVELIAPCLPGFGFSDPTLRQGLGAAQMAIIMRNLM  222

Query  628   MRLGFEKFYIQAGDWGSQCATHMATLFPEQVLGLHTNMPL--SSKPVSTLKLILGALVPR  801
              RLGF++FYIQ GDWG     + +ATLFP++VLG HTN  L  SS P       L +L P
Sbjct  223   HRLGFKRFYIQGGDWGGFIGSDIATLFPQEVLGFHTNWALVTSSAPA------LNSLQP-  275

Query  802   LAVDRKYADRIYPLKNLFSYLLRESGYFHIQATKPDTIGVALTDSPSGLAAYIIEKMAIC  981
                       + PL      ++ E+GY HI AT+PDT+G+ALTDSP+GL A+++EK +
Sbjct  276   ----------VAPL------IMEETGYLHIAATRPDTVGIALTDSPAGLLAFLLEKFSTS  319

Query  982   SSRIELDTPHGGLQHldl-ddvldtvtITWMNNCIVTSMRLYAEGFALP-EVQTVHDIPT  1155
                       GGL +    + ++D V   W    + TS+RLYAE F    +   V   PT
Sbjct  320   VKHENRQLADGGLHNTFTPETLIDDVMFYWTTRSMPTSLRLYAENFNKKYQALGVERTPT  379
```

```
Query  1156  YVPTAAINFLYEVIYQPDWILRDKFKNLVRSTVIESGGHFAAMQTPNLLTDDIFDSAVEF  1335
              VPT      YE+ +Q   IL+ K++NL+ +T ++ GGHF A++ P   ++++  +   F
Sbjct  380   TVPTWVTQGKYELTHQSAEILKTKYQNLLNATSLDFGGHFFALEQPKFFSENVLTALKAF  439

Query  1336  LKFHEKNK  1359
               +H+ +K
Sbjct  440   RIWHQTHK  447


> ta_transcript45224_1
Length=598

 Score =   309 bits (792),  Expect = 3e-97, Method: Compositional matrix adjust.
 Identities = 171/432 (40%), Positives = 248/432 (57%), Gaps = 33/432 (8%)
 Frame = +1

Query  76    NPPELDLQKWWGSGSPVAVVDTSIRPFKIEFNYTMIKDLKERLHNRRTFTKPLQGIQSEY  255
             N   +DLQ+WWG        DTSIR  KI F+ +M++DLK+RL     F  PL+     Y
Sbjct  42    NRSAIDLQEWWGPKDLQGNEDTSIRSAKIHFSKSMVRDLKQRLKQHVPFQPPLEDSGFNY  101

Query  256   GINTIYLETVLDYWVEDYDFKKRADLLNMFPHYKTNIQGLDIHFIRVKPDVE-DVEVLPL  432
             G N+  ++  + YW E+Y F++R   LN FP +KTNIQGLD+HFI VKP+V  +  V+PL
Sbjct  102   GFNSGAMDYWVKYWSEEYPFEEREAFLNQFPQFKTNIQGLDMHFIHVKPNVTGNKPVIPL  161

Query  433   LMLHGWPSSSKEFDKVIPMLTRPRVGYNFVFEVIAADLPGYGFSEGTNKPGLNPVQIGVI  612
             L+LHGWP S +EF   IP+LT      +F  EV+   LPG+GFS+GT+K GL  VQ+ VI
Sbjct  162   LILHGWPGSIREFYGAIPLLTADSPDRDFAVEVVVPCLPGFGFSDGTSKIGLGAVQMAVI  221

Query  613   MRNLMMRLGFEKFYIQAGDWGSQCATHMATLFPEQVLGLHTNMPLSSKPVSTLKLILGAL  792
             +RNLM RLG ++FY Q GDWG    +++AT+FPE+ LG H N  +    V      L +L
Sbjct  222   LRNLMHRLGHKQFYTQGGDWGGIIGSYIATIFPEENLGFHANWGVVLSSVG-----LNSL  276

Query  793   VPRLAVDRKYADRIYPLKNLFSYLLRESGYFHIQATKPDTIGVALTDSPSGLAAYIIEKM  972
              P+  +                 +L + GY H+ ATKPDT+G+ALTDSP+GL A+I+EK
Sbjct  277   KPQAEI-----------------VLLKGGYLHLAATKPDTVGMALTDSPTGLLAFILEKF  319

Query  973   AICSSRIELDTPHGGLQHld-lddvldtvtITWMNNCIVTSMRLYAEGF-----ALPEVQ  1134
             +   +    D   GGL++      ++D +   W    I  ++RLYAE F     AL   +
Sbjct  320   SGAVTNANNDLADGGLRNTFPAVALIDDLMFYWTERKITNTLRLYAETFNKKTRALGIDE  379

Query  1135  TVHDIPTYVPTAAINFLYEVIYQPDWILRDKFKNLVRSTVIESGGHFAAMQTPNLLTDDI  1314
                 +P +V      F  +    P+ IL+ K+ NL+ +  ++ G HF A++ P + TD++
Sbjct  380   ARSPVPIWVTQGKAEFTKQT---PE-ILKTKYDNLLHAESLQVGNHFLALELPQVYTDNV  435

Query  1315  FDSAVEFLKFHE  1350
             F+    F+ FH+
Sbjct  436   FNGLKAFIDFHK  447


> ta_transcript58145_1
Length=369

 Score =   245 bits (626),  Expect = 2e-75, Method: Compositional matrix adjust.
 Identities = 131/283 (46%), Positives = 181/283 (64%), Gaps = 4/283 (1%)
 Frame = +1

Query  514   NFVFEVIAADLPGYGFSEGTNKPGLNPVQIGVIMRNLMMRLGFEKFYIQAGDWGSQCATH  693
             NF  E+I   LPG+G+S+   +PGL   +I VIMRNLM RLG++++YIQ GD G+
Sbjct  7     NFALELIIPSLPGFGWSDAAVRPGLGAAEIAVIMRNLMHRLGYKQYYIQGGDAGATIGKI  66

Query  694   MATLFPEQVLGLHTNMP--LSSKPVSTLKLILGALVPRLAVDRKYADRIYPLKNLFSYLL  867
             M TLFP++VLG HTN    +SS P + L   + +  P L V+ +  DR+YPLK  +S+L+
Sbjct  67    MITLFPKEVLGYHTNFLFYVSSSPKALLLEWIYSFWPSLFVEPELVDRMYPLKKTYSFLI  126

Query  868   RESGYFHIQATKPDTIGVALTDSPSGLAAYIIEKMAICSSRIELDTPHGGL-QHldlddv  1044
              E+GYFHIQATKPDT+GVAL+DSP+GL AYI+EK +  ++R     P GGL ++   + +
Sbjct  127   EETGYFHIQATKPDTVGVALSDSPAGLLAYILEKFSTGTNRNFRSLPDGGLTKYFTKEQL  186

Query  1045  ldtvtITWMNNCIVTSMRLYAEGFALPEVQTVHD-IPTYVPTAAINFLYEVIYQPDWILR  1221
             +D + + W    + TSMR YAE F    +    D IPT VPT  +   +E+ YQP  ILR
Sbjct  187   IDNLMVYWSTQSVTTSMRYYAETFNKRHLGLKFDSIPTTVPTWGMQAKHEMSYQPASILR  246

Query  1222  DKFKNLVRSTVIESGGHFAAMQTPNLLTDDIFDSAVEFLKFHE  1350
              KF NLV  TV+E GGHF A+Q P+   DD+  S   F ++H+
Sbjct  247   LKFLNLVNLTVLEDGGHFMALQMPDTFADDVLKSVAAFREWHK  289
```

```
> ta_transcript58144_1
Length=440

 Score =   245 bits (626),  Expect = 2e-74, Method: Compositional matrix adjust.
 Identities = 131/283 (46%), Positives = 181/283 (64%), Gaps = 4/283 (1%)
 Frame = +1

Query  514   NFVFEVIAADLPGYGFSEGTNKPGLNPVQIGVIMRNLMMRLGFEKFYIQAGDWGSQCATH  693
             NF  E+I   LPG+G+S+   +PGL   +I VIMRNLM RLG++++YIQ GD G+
Sbjct  7     NFALELIIPSLPGFGWSDAAVRPGLGAAEIAVIMRNLMHRLGYKQYYIQGGDAGATIGKI  66

Query  694   MATLFPEQVLGLHTNMP--LSSKPVSTLKLILGALVPRLAVDRKYADRIYPLKNLFSYLL  867
             M TLFP++VLG HTN    +SS P + L   + +  P L V+ +  DR+YPLK  +S+L+
Sbjct  67    MITLFPKEVLGYHTNFLFYVSSSPKALLLEWIYSFWPSLFVEPELVDRMYPLKKTYSFLI  126

Query  868   RESGYFHIQATKPDTIGVALTDSPSGLAAYIIEKMAICSSRIELDTPHGGL-QHldlddv  1044
              E+GYFHIQATKPDT+GVAL+DSP+GL AYI+EK +  ++R     P GGL ++   + +
Sbjct  127   EETGYFHIQATKPDTVGVALSDSPAGLLAYILEKFSTGTNRNFRSLPDGGLTKYFTKEQL  186

Query  1045  ldtvtITWMNNCIVTSMRLYAEGFALPEVQTVHD-IPTYVPTAAINFLYEVIYQPDWILR  1221
             +D + + W    + TSMR YAE F    +    D IPT VPT  +   +E+ YQP  ILR
Sbjct  187   IDNLMVYWSTQSVTTSMRYYAETFNKRHLGLKFDSIPTTVPTWGMQAKHEMSYQPASILR  246

Query  1222  DKFKNLVRSTVIESGGHFAAMQTPNLLTDDIFDSAVEFLKFHE  1350
              KF NLV  TV+E GGHF A+Q P+   DD+ S   F ++H+
Sbjct  247   LKFLNLVNLTVLEDGGHFMALQMPDTFADDVLKSVAAFREWHK  289


> ta_transcript35275_1
Length=728

 Score =   138 bits (348),  Expect = 2e-34, Method: Compositional matrix adjust.
 Identities = 160/431 (37%), Positives = 213/431 (49%), Gaps = 4/431 (1%)
 Frame = -2

Query  1355  FFS*NFKNSTAESKMSSVSKLGVCMAAKCPPDSITVDLTKFLNLSLRIQSGW*ITSYKKF  1176
              F *N KN    SK+SS S  G  +AAK PP S  VD   F+    +  SG *I S
Sbjct  202   LFP*NSKNFPTASKISSASTAGNSIAAKWPPSSTAVD*R*FVYFCFKTMSGV*IMSCLNL  261

Query  1175  IAAVGT*VGMS*TVCTSGSANPSAYSLMDVTMQLFIH-VIvtvsktssksKC*RPPCGVS  999
             I AVGT VG+S ++C +     S  +L+DV M L    +I   S  S++    R P
Sbjct  262   IQAVGTGVGIS-SICKAKLFTASL*TLIDVVMLLLAQ*IITLSSSWFSENFWSRFPSAAC  320

Query  998   SSILLLQMAIFSIIYAAKPDGESVSATPIVSGFVA*I*KYPLSLSKYEKRFFNGYIL-SA  822
                 L+Q+  FS + AA+P GESVSATPIVSG VA +   P+S S+  +   +G    ++
Sbjct  321   RCWGLVQVENFSRM*AARPAGESVSATPIVSGLVACMCVNPVSWSRKARWSVSGARRGTS  380

Query  821   YFRSTANLGTKAPNINFNVLTGFDDSGMFVCNPRTCSGNKVAICVAHCEPQSPA*M*NFS  642
               RST   G   P    N       SG+ V +P T  G+ VA+      P SP  M*
Sbjct  381   SGRSTTQEGNTDPIK**NSACL*MTSGVLVWSPSTAVGSSVAMLDPTA*PTSPPCM*YPL  440

Query  641   KPSRIIKFRIITPI*TGLRPGLFVPSENP*PGKSAAITSKTKL*PTLGLVSIGITLSNSF  462
              P R I+  + T I TG RP    PSE P PG+   ITS   L P+ G++S G+    S
Sbjct  441   *PMRCIRLCMTTAIWTGPRPERTAPSEYPKPGRLGTITSNANLYPSRGVISFGMIS*YSL  500

Query  461   EELGHPCNIKSGRtst-sstSGFTLMK*MSSP*IFVL*WGNIFNRSALFLKs*sstq*sr  285
             ++ GHPC+I  G T T  S++G TL+K +SSP I VL*   +   S+   K     Q*S
Sbjct  501   KDPGHPCSISRGTTVTPLSSAGRTLIKCISSPCILVL**LYLLR*SSRSRKLYLFFQ*SS  560

Query  284   tvSK*MVLIPYSDWIP*SGFVNVRRLCNLSFKSLIIV*LNSILKGLIDVSTTATGDPLPH  105
                + +VL PY +    +G V  R     S +S  I   N I  GL++VS   +    PH
Sbjct  561   ICVRYIVLKPYVEPESSNGGVKGRW*RRRSRRSFTITSSNLISNGLMEVSGLVSSGYEPH  620

Query  104   HFWRSNSGGFG  72
             H  +S  G  G
Sbjct  621   HCSKSICGTSG  631

************************************************************************************************
********************************************************************

Query= gi|112984537:97-1482 Bombyx mori juvenile hormone epoxide hydrolase (Jheh2), mRNA

Length=1386
                                                                      Score     E
Sequences producing significant alignments:                          (Bits)  Value

  ta_transcript53250_1                                                  625    0.0
```

```
  ta_transcript53251_1                                               623   0.0
  ta_transcript29071_1                                               453   6e-154
  ta_transcript45224_1                                               441   3e-148
  ta_transcript58145_1                                               384   4e-129
  ta_transcript58144_1                                               385   1e-128
  ta_transcript35275_1                                               202   2e-56
  ta_transcript53254_1                                               128   2e-31


> ta_transcript53250_1
Length=647

 Score =   625 bits (1611),  Expect = 0.0, Method: Compositional matrix adjust.
 Identities = 286/458 (62%), Positives = 370/458 (81%), Gaps = 1/458 (0%)
 Frame = +1

Query  1     MSRllfialpllvlASIPLYLLVLKSPPPMPKLDLEEWWGPPELKQKQDTSIKPFEITFS  180
             M+RLL     +  + ++ +Y  +L++PP +P+LDL  WWGP  LK KQD S++P ++ F
Sbjct  98    MARLLLFFATITAVVAVVIYFALLRAPP-LPELDLNAWWGPDSLKTKQDDSVRPMKLKFK  156

Query  181   ETMVKELKERIKKRRPFAPPLEGVGFKYGFNSKQLDSWLKYWAEEYPFAERQKFLNQYPH  360
             + M+++L++ +K  R FAPPLEGVGF+YGFNS  +DSWLKYWAEEY F ER++F NQY
Sbjct  157   KPMIQDLQQYLKTTRKFAPPLEGVGFEYGFNSNNMDSWLKYWAEEYNFEERERFFNQYES  216

Query  361   FKTNIQGLNIHFMRITPKVPKDVEIVPLLLLHGWPGSVREFYEAIPHLTAVSRDRNFALE  540
             ++T IQGLNIHF+ + P+VP  VE+VP+LLLHGWPGSVREFYEAIP LTAV + RNFALE
Sbjct  217   YRTLIQGLNIHFIHVKPQVPAGVEVVPMLLLHGWPGSVREFYEAIPLLTAVDKTRNFALE  276

Query  541   IIAPSLPGYGFSDAAVRPGLAAAEVAVIFKNLMARLGYKQYYVQGGDWGALIGSAMATSF  720
             +I PSLPGYG+SDAAVRPGL AAEVAV+ KNLM RLGYKQYY+QGGDWGA+I +A++T F
Sbjct  277   LIIPSLPGYGWSDAAVRPGLGAAEVAVVMKNLMNRLGYKQYYLQGGDWGAVICTALSTLF  336

Query  721   PKEIIGFHSYMALTLSPAATFLEFVGALFPSLIVEPELANRLYPLSEKYSTLLEELGYMH  900
             P E++G+H+ M   +SPAAT LE++ +L+P+L +EPEL +R YPLS+ YS L+EE+GYMH
Sbjct  337   PNEVLGYHTNMLFNMSPAATALEWLFSLWPTLAIEPELVDRAYPLSKTYSHLMEEMGYMH  396

Query  901   IQATKPDTVGIGLTDSPAGLLAYILEKFSTWTNPDLRSKEDGGLSYRWTKDQLIDNLMLY  1080
             IQATKPDTVG+ L+DSPAGLLAYILEKFSTWT+ +L S +DGGL+  +TK+QL+DNLM+Y
Sbjct  397   IQATKPDTVGVALSDSPAGLLAYILEKFSTWTDRNLISSKDGGLTKHFTKEQLVDNLMVY  456

Query  1081  WSTKSIVTSMRLYAESFSSRHFDLKLDEIQVQVPTWVLQAKHELAYQPPCILKLKYTKLV  1260
             WST+SI TSMRLYAESF+ RH  L+LD I   VP W +QAKHE++YQPP IL+LK+  L+
Sbjct  457   WSTQSITTSMRLYAESFNKRHLALELDSIPTTVPVWGIQAKHEISYQPPVILRLKFPNLL  516

Query  1261  NASVIEDGGHFLAFELPEIFAKDVLKAIGEFRKLKNVK  1374
             + +V+E+GGHF AF+LP+IFA+DVLK +  FR     K
Sbjct  517   HTTVLEEGGHFFAFQLPKIFAEDVLKGVSAFRDWHKAK  554


> ta_transcript53251_1
Length=624

 Score =   623 bits (1607),  Expect = 0.0, Method: Compositional matrix adjust.
 Identities = 286/458 (62%), Positives = 370/458 (81%), Gaps = 1/458 (0%)
 Frame = +1

Query  1     MSRllfialpllvlASIPLYLLVLKSPPPMPKLDLEEWWGPPELKQKQDTSIKPFEITFS  180
             M+RLL     +  + ++ +Y  +L++PP +P+LDL  WWGP  LK KQD S++P ++ F
Sbjct  98    MARLLLFFATITAVVAVVIYFALLRAPP-LPELDLNAWWGPDSLKTKQDDSVRPMKLKFK  156

Query  181   ETMVKELKERIKKRRPFAPPLEGVGFKYGFNSKQLDSWLKYWAEEYPFAERQKFLNQYPH  360
             + M+++L++ +K  R FAPPLEGVGF+YGFNS  +DSWLKYWAEEY F ER++F NQY
Sbjct  157   KPMIQDLQQYLKTTRKFAPPLEGVGFEYGFNSNNMDSWLKYWAEEYNFEERERFFNQYES  216

Query  361   FKTNIQGLNIHFMRITPKVPKDVEIVPLLLLHGWPGSVREFYEAIPHLTAVSRDRNFALE  540
             ++T IQGLNIHF+ + P+VP  VE+VP+LLLHGWPGSVREFYEAIP LTAV + RNFALE
Sbjct  217   YRTLIQGLNIHFIHVKPQVPAGVEVVPMLLLHGWPGSVREFYEAIPLLTAVDKTRNFALE  276

Query  541   IIAPSLPGYGFSDAAVRPGLAAAEVAVIFKNLMARLGYKQYYVQGGDWGALIGSAMATSF  720
             +I PSLPGYG+SDAAVRPGL AAEVAV+ KNLM RLGYKQYY+QGGDWGA+I +A++T F
Sbjct  277   LIIPSLPGYGWSDAAVRPGLGAAEVAVVMKNLMNRLGYKQYYLQGGDWGAVICTALSTLF  336

Query  721   PKEIIGFHSYMALTLSPAATFLEFVGALFPSLIVEPELANRLYPLSEKYSTLLEELGYMH  900
             P E++G+H+ M   +SPAAT LE++ +L+P+L +EPEL +R YPLS+ YS L+EE+GYMH
Sbjct  337   PNEVLGYHTNMLFNMSPAATALEWLFSLWPTLAIEPELVDRAYPLSKTYSHLMEEMGYMH  396
```

```
Query  901   IQATKPDTVGIGLTDSPAGLLAYILEKFSTWTNPDLRSKEDGGLSYRWTKDQLIDNLMLY  1080
             IQATKPDTVG+ L+DSPAGLLAYILEKFSTWT+ +L S +DGGL+  +TK+QL+DNLM+Y
Sbjct  397   IQATKPDTVGVALSDSPAGLLAYILEKFSTWTDRNLISSKDGGLTKHFTKEQLVDNLMVY  456

Query  1081  WSTKSIVTSMRLYAESFSSRHFDLKLDEIQVQVPTWVLQAKHELAYQPPCILKLKYTKLV  1260
             WST+SI TSMRLYAESF+ RH  L+LD I   VP W +QAKHE++YQPP IL+LK+  L+
Sbjct  457   WSTQSITTSMRLYAESFNKRHLALELDSIPTTVPVWGIQAKHEISYQPPVILRLKFPNLL  516

Query  1261  NASVIEDGGHFLAFELPEIFAKDVLKAIGEFRKLKNVK  1374
             + +V+E+GGHF AF+LP+IFA+DVLK +  FR     K
Sbjct  517   HTTVLEEGGHFFAFQLPKIFAEDVLKGVSAFRDWHKAK  554


> ta_transcript29071_1
Length=520

 Score =   453 bits (1165),  Expect = 6e-154, Method: Compositional matrix adjust.
 Identities = 223/419 (53%), Positives = 299/419 (71%), Gaps = 21/419 (5%)
 Frame = +1

Query  100   DLEEWWGPPELKQKQDTSIKPFEITFSETMVKELKERIKKRRPFAPPLEGVGFKYGFNSK  279
             D++EWWGP  L+  +DTSI+P +I F   M+K+L+ R++K   F PPLE  GF YGFNS
Sbjct  43    DIQEWWGPKSLQGHEDTSIRPAKIHFENKMIKDLQGRLRKTISFQPPLEDSGFTYGFNSD  102

Query  280   QLDSWLKYWAEEYPFAERQKFLNQYPHFKTNIQGLNIHFMRITPKVPKDVEIVPLLLLHG  459
              +  WLKYWAEEYPF ER+ +LNQ+P FKTNIQGL+IHF+ + PKV     ++PLLLLHG
Sbjct  103   SIGYWLKYWAEEYPFKEREAYLNQFPQFKTNIQGLDIHFIHVKPKVQTHKNVIPLLLLHG  162

Query  460   WPGSVREFYEAIPHLTAVSRDRNFALEIIAPSLPGYGFSDAAVRPGLAAAEVAVIFKNLM  639
             W GS+REFY+AIP LTA S+DR+FA+E+IAP LPG+GFSD  +R GL AA++A+I +NLM
Sbjct  163   WAGSIREFYDAIPLLTADSKDRDFAVELIAPCLPGFGFSDPTLRQGLGAAQMAIIMRNLM  222

Query  640   ARLGYKQYYVQGGDWGALIGSAMATSFPKEIIGFHSYMALTLSPAATFLEFVGALFPSLI  819
              RLG+K++Y+QGGDWG  IGS +AT FP+E++GFH+  AL  S A           P+L
Sbjct  223   HRLGFKRFYIQGGDWGGFIGSDIATLFPQEVLGFHTNWALVTSSA-----------PAL-  270

Query  820   VEPELANRLYPLSEKYSTLLEELGYMHIQATKPDTVGIGLTDSPAGLLAYILEKFSTWTN  999
                   N L P++     ++EE GY+HI AT+PDTVGI LTDSPAGLLA++LEKFST
Sbjct  271   ------NSLQPVA---PLIMEETGYLHIAATRPDTVGIALTDSPAGLLAFLLEKFSTSVK  321

Query  1000  PDLRSKEDGGLSYRWTKDQLIDNLMLYWSTKSIVTSMRLYAESFSSRHFDLKLDEIQVQV  1179
              + R   DGGL   +T + LID++M YW+T+S+ TS+RLYAE+F+ ++  L ++     V
Sbjct  322   HENRQLADGGLHNTFTPETLIDDVMFYWTTRSMPTSLRLYAENFNKKYQALGVERTPTTV  381

Query  1180  PTWVLQAKHELAYQPPCILKLKYTKLVNASVIEDGGHFLAFELPEIFAKDVLKAIGEFR  1356
             PTWV Q K+EL +Q   ILK KY  L+NA+ ++ GGHF A E P+ F+++VL A+  FR
Sbjct  382   PTWVTQGKYELTHQSAEILKTKYQNLLNATSLDFGGHFFALEQPKFFSENVLTALKAFR  440


> ta_transcript45224_1
Length=598

 Score =   441 bits (1134),  Expect = 3e-148, Method: Compositional matrix adjust.
 Identities = 218/429 (51%), Positives = 290/429 (68%), Gaps = 31/429 (7%)
 Frame = +1

Query  97    LDLEEWWGPPELKQKQDTSIKPFEITFSETMVKELKERIKKRRPFAPPLEGVGFKYGFNS  276
             +DL+EWWGP +L+  +DTSI+  +I FS++MV++LK+R+K+  PF PPLE  GF YGFNS
Sbjct  46    IDLQEWWGPKDLQGNEDTSIRSAKIHFSKSMVRDLKQRLKQHVPFQPPLEDSGFNYGFNS  105

Query  277   KQLDSWLKYWAEEYPFAERQKFLNQYPHFKTNIQGLNIHFMRITPKVPKDVEIVPLLLLH  456
               +D W+KYW+EEYPF ER+ FLNQ+P FKTNIQGL++HF+ + P V  +  ++PLL+LH
Sbjct  106   GAMDYWVKYWSEEYPFEEREAFLNQFPQFKTNIQGLDMHFIHVKPNVTGNKPVIPLLILH  165

Query  457   GWPGSVREFYEAIPHLTAVSRDRNFALEIIAPSLPGYGFSDAAVRPGLAAAEVAVIFKNL  636
             GWPGS+REFY AIP LTA S DR+FA+E++ P LPG+GFSD   + GL A ++AVI +NL
Sbjct  166   GWPGSIREFYGAIPLLTADSPDRDFAVEVVVPCLPGFGFSDGTSKIGLGAVQMAVILRNL  225

Query  637   MARLGYKQYYVQGGDWGALIGSAMATSFPKEIIGFHSYMALTLSPAATFLEFVGALFPSL  816
             M RLG+KQ+Y QGGDWG +IGS +AT FP+E +GFH+   + LS
Sbjct  226   MHRLGHKQFYTQGGDWGGIIGSYIATIFPEENLGFHANWGVVLSSVG-------------  272

Query  817   IVEPELANRLYPLSEKYSTLLEELGYMHIQATKPDTVGIGLTDSPAGLLAYILEKFS---  987
                    N L P +E    +L + GY+H+ ATKPDTVG+ LTDSP GLLA+ILEKFS
Sbjct  273   ------LNSLKPQAE---IVLLKGGYLHLAATKPDTVGMALTDSPTGLLAFILEKFSGAV  323

Query  988   TWTNPDLRSKEDGGLSYRWTKDQLIDNLMLYWSTKSIVTSMRLYAESFSSRHFDLKLDEI  1167
```

```
            T  N DL    DGGL   +    LID+LM YW+ + I  ++RLYAE+F+ +   L +DE
Sbjct  324   TNANNDL---ADGGLRNTFPAVALIDDLMFYWTERKITNTLRLYAETFNKKTRALGIDEA  380

Query  1168  QVQVPTWVLQAKHELAYQPPCILKLKYTKLVNASVIEDGGHFLAFELPEIFAKDV---LK  1338
             +  VP WV Q K E   Q P ILK KY  L++A  ++ G HFLA ELP+++  +V   LK
Sbjct  381   RSPVPIWVTQGKAEFTKQTPEILKTKYDNLLHAESLQVGNHFLALELPQVYTDNVFNGLK  440

Query  1339  AIGEFRKLK  1365
             A  +F K K
Sbjct  441   AFIDFHKSK  449


> ta_transcript58145_1
Length=369

 Score =   384 bits (986),  Expect = 4e-129, Method: Compositional matrix adjust.
 Identities = 181/286 (63%), Positives = 227/286 (79%), Gaps = 2/286 (1%)
 Frame = +1

Query  508   AVSRDRNFALEIIAPSLPGYGFSDAAVRPGLAAAEVAVIFKNLMARLGYKQYYVQGGDWG  687
             AV + RNFALE+I PSLPG+G+SDAAVRPGL AAE+AVI +NLM RLGYKQYY+QGGD G
Sbjct  1     AVDKTRNFALELIIPSLPGFGWSDAAVRPGLGAAEIAVIMRNLMHRLGYKQYYIQGGDAG  60

Query  688   ALIGSAMATSFPKEIIGFHS--YMALTLSPAATFLEFVGALFPSLIVEPELANRLYPLSE  861
             A IG  M T FPKE++G+H+     ++ SP A  LE++ + +PSL VEPEL +R+YPL +
Sbjct  61    ATIGKIMITLFPKEVLGYHTNFLFYVSSSPKALLLEWIYSFWPSLFVEPELVDRMYPLKK  120

Query  862   KYSTLLEELGYMHIQATKPDTVGIGLTDSPAGLLAYILEKFSTWTNPDLRSKEDGGLSYR  1041
              YS L+EE GY HIQATKPDTVG+ L+DSPAGLLAYILEKFST TN + RS  DGGL+
Sbjct  121   TYSFLIEETGYFHIQATKPDTVGVALSDSPAGLLAYILEKFSTGTNRNFRSLPDGGLTKY  180

Query  1042  WTKDQLIDNLMLYWSTKSIVTSMRLYAESFSSRHFDLKLDEIQVQVPTWVLQAKHELAYQ  1221
             +TK+QLIDNLM+YWST+S+ TSMR YAE+F+ RH  LK D I   VPTW +QAKHE++YQ
Sbjct  181   FTKEQLIDNLMVYWSTQSVTTSMRYYAETFNKRHLGLKFDSIPTTVPTWGMQAKHEMSYQ  240

Query  1222  PPCILKLKYTKLVNASVIEDGGHFLAFELPEIFAKDVLKAIGEFRK  1359
             P  IL+LK+  LVN +V+EDGGHF+A ++P+ FA DVLK++  FR+
Sbjct  241   PASILRLKFLNLVNLTVLEDGGHFMALQMPDTFADDVLKSVAAFRE  286


> ta_transcript58144_1
Length=440

 Score =   385 bits (989),  Expect = 1e-128, Method: Compositional matrix adjust.
 Identities = 181/286 (63%), Positives = 227/286 (79%), Gaps = 2/286 (1%)
 Frame = +1

Query  508   AVSRDRNFALEIIAPSLPGYGFSDAAVRPGLAAAEVAVIFKNLMARLGYKQYYVQGGDWG  687
             AV + RNFALE+I PSLPG+G+SDAAVRPGL AAE+AVI +NLM RLGYKQYY+QGGD G
Sbjct  1     AVDKTRNFALELIIPSLPGFGWSDAAVRPGLGAAEIAVIMRNLMHRLGYKQYYIQGGDAG  60

Query  688   ALIGSAMATSFPKEIIGFHS--YMALTLSPAATFLEFVGALFPSLIVEPELANRLYPLSE  861
             A IG  M T FPKE++G+H+     ++ SP A  LE++ + +PSL VEPEL +R+YPL +
Sbjct  61    ATIGKIMITLFPKEVLGYHTNFLFYVSSSPKALLLEWIYSFWPSLFVEPELVDRMYPLKK  120

Query  862   KYSTLLEELGYMHIQATKPDTVGIGLTDSPAGLLAYILEKFSTWTNPDLRSKEDGGLSYR  1041
              YS L+EE GY HIQATKPDTVG+ L+DSPAGLLAYILEKFST TN + RS  DGGL+
Sbjct  121   TYSFLIEETGYFHIQATKPDTVGVALSDSPAGLLAYILEKFSTGTNRNFRSLPDGGLTKY  180

Query  1042  WTKDQLIDNLMLYWSTKSIVTSMRLYAESFSSRHFDLKLDEIQVQVPTWVLQAKHELAYQ  1221
             +TK+QLIDNLM+YWST+S+ TSMR YAE+F+ RH  LK D I   VPTW +QAKHE++YQ
Sbjct  181   FTKEQLIDNLMVYWSTQSVTTSMRYYAETFNKRHLGLKFDSIPTTVPTWGMQAKHEMSYQ  240

Query  1222  PPCILKLKYTKLVNASVIEDGGHFLAFELPEIFAKDVLKAIGEFRK  1359
             P  IL+LK+  LVN +V+EDGGHF+A ++P+ FA DVLK++  FR+
Sbjct  241   PASILRLKFLNLVNLTVLEDGGHFMALQMPDTFADDVLKSVAAFRE  286


> ta_transcript35275_1
Length=728

 Score =   202 bits (515),  Expect = 2e-56, Method: Compositional matrix adjust.
 Identities = 176/404 (44%), Positives = 210/404 (52%), Gaps = 3/404 (1%)
 Frame = -2

Query  1352  NSPIAFRTSLANISGSSNARKWPPSSMTLALTSFVYFNLRMQGGW*ANSCFAWSTHVGTC  1173
             N P A + S A+ +G+S A KWPPSS  +    FVYF  +   G *  SC      VGT
```

```
Sbjct  209   NFPTASKISSASTAGNSIAAKWPPSSTAVD*R*FVYFCFKTMSGV*IMSCLNLIQAVGTG  268

Query  1172  T*ISSSFKSKCLELKDSA*SLIEVTIDFVLQYSMRLSISWSFVHR*ERPPSSLLRKSGLV  993
               ISS  K+K      S *+LI+V +  + Q  + LS SW   +   R PS+  R  GLV
Sbjct  269   VGISSICKAKLFT--ASL*TLIDVVMLLLAQ*IITLSSSWFSENFWSRFPSAACRCWGLV  326

Query  992   QVENFSRI*ARRPAGESVNPIPTVSGFVA*ICM*PNSSRRVEYFSDNG*SLLASSG-STI  816
             QVENFSR+*A RPAGESV+  P VSG VA +C+ P S  R   +S +G     SSG ST
Sbjct  327   QVENFSRM*AARPAGESVSATPIVSGLVACMCVNPVSWSRKARWSVSGARRGTSSGRSTT  386

Query  815   RDGNKAPTNSRNVAAGLRVSAM*EWKPIISLGKEVAIALPMRAPQSPPWT*YCL*PSLAI  636
             ++GN  P    N A     S +  W P  ++G  VA+  P   P SPP  *Y L*P   I
Sbjct  387   QEGNTDPIK**NSACL*MTSGVLVWSPSTAVGSSVAMLDPTA*PTSPPCM*YPL*PMRCI  446

Query  635   RFLKMTATSAAARPGRTAASEKP*PGKLGAIISRAKFLSLLTAVR*GMAS*NSLTEPGHP  456
             R    TA     RP RTA SE P PG+LG I S A        +  GM S* SL +PGHP
Sbjct  447   RLCMTTAIWTGPRPERTAPSEYPKPGRLGTITSNANLYPSRGVISFGMIS*YSLKDPGHP  506

Query  455   WSNRSGTISTSLGTFGVILMK*IFKP*ILVLK*GYWLRNFCLSANGYSSAQYLSQLSNCF  276
              S   GT  T L + G  L+K I  P ILVL * Y LR    S   Y   Q  S
Sbjct  507   CSISRGTTVTPLSSAGRTLIKCISSPCILVL**LYLLR*SSRSRKLYLFFQ*SSICVRYI  566

Query  275   ELNPYLKPTPSNGGAKGLRFLMRSLSSFTIVSLKVISKGLMLVS  144
              L PY++P  SNGG KG     RS  SFTI S  +IS GLM VS
Sbjct  567   VLKPYVEPESSNGGVKGRW*RRRSRRSFTITSSNLISNGLMEVS  610


> ta_transcript53254_1
Length=515

 Score =  128 bits (321),  Expect = 2e-31, Method: Compositional matrix adjust.
 Identities = 124/390 (32%), Positives = 185/390 (47%), Gaps = 0/390 (0%)
 Frame = +3

Query  102   PGGVVGTART*AETGYQHQTLRDHFQ*NDGKRT*RTHQETKTFRPSIGGCWLQIRVQFET  281
             PG VVG  R   E   QHQ    + +* D KR+*R   +  +   S       +R+Q +T
Sbjct  55    PGRVVGAKRNKGEN*RQHQAFPSYIR*RDDKRS*RASPKAPSIHSSS*RRRFPVRLQRQT  114

Query  282   IGQLAQILGRRIPIR*KAEVP*PISSFQD*YSGFEYSLHEDYSKGP*RR*NSTTSVTPRM  461
             +G L ++LG  + + *+ EV  P+SS QD YS   ++ H+ Y+ G  +           +
Sbjct  115   VGFLGELLGHEVQLH*ETEVLQPVSSIQDQYSRIGHTFHQGYASGSSQYQGGAHPPDAWL  174

Query  462   AGLRQGVLRshssshsCQQRQELRSGNYRSKFTWLWLFrrggssrprcc*scsHFQKPDG  641
             A LR GVLR ++S+HS + R  LR    R++  W+W+ R     R          Q+PD
Sbjct  175   ARLR*GVLRGNTSAHSTKARVRLRIRGGRAQHPWIWILRSSSPPRVGSTPCWRGLQEPDA  234

Query  642   QTWLQAVLRPRR*LGSSHR*RYGYFLP*GNNRLPLLHGAYS*SSGDIP*VCRCLIPISDS  821
              +  Q VLRPRR*LG  H   +   +P* +  +P           D     R  + ++
Sbjct  235   SSRPQEVLRPRR*LGRGHCWSHFNHIP**STGIP*QPAYQPVPLCDDISTLRRHLSLAGD  294

Query  822   RTRTS*QTLPVIGEILNPSRGIGLHAYSSHEA*YCWNWINRLPGRSPRLYPGEVFHLDQP  1001
             R    *Q +P    +L  S G+ +HA + H  *Y W   N LP R  RL+P E+ HLD
Sbjct  295   RPEAG*QMVPARSLLLLHS*GVRIHASAVH*T*YRWCGPNGLPIRPTRLHPREILHLDLK  354

Query  1002  RLTQ*rrrrSLLPVDKRPADR*SHAVLEHEVNSHFNETLC*VFQF*ALRFKTR*DSSTSP  1181
              L     RR+   + +RPADR* H +L +  + H +E + *  +    R    *     P
Sbjct  355   GLQIPP*RRAREEIQQRPADR*PHGLLVY*FHHHLHEIIL*DNE*QG*RAHDV*RPHACP  414

Query  1182  NMGAPGETRVSLPAALHPQIEVHETGQCQC  1271
             ++G PG+ R  L  ++  + EVH+     C
Sbjct  415   DLGYPGQARDLLHPSVAAEEEVHQPAGHHC  444


***************************************************************************************************
*********************************************************************
Query= gi|261245096:150-1556 Bombyx mori juvenile hormone epoxide hydrolase-like protein 2
(Jheh-lp2), mRNA

Length=1407
                                                                      Score     E
Sequences producing significant alignments:                          (Bits)  Value

  ta_transcript53250_1                                                  519    9e-178
  ta_transcript53251_1                                                  517    2e-177
  ta_transcript29071_1                                                  432    1e-145
  ta_transcript45224_1                                                  405    3e-134
```

```
  ta_transcript58145_1                                               318   1e-103
  ta_transcript58144_1                                               319   9e-103
  ta_transcript35275_1                                               136   1e-33


> ta_transcript53250_1
Length=647

 Score =   519 bits (1336),  Expect = 9e-178, Method: Compositional matrix adjust.
 Identities = 245/449 (55%), Positives = 321/449 (71%), Gaps = 1/449 (0%)
 Frame = +1

Query  10    LFRLVVASCLTIELLASQNTEPAYKLDPNAWWGPECTKYHNDTSIRPFEIVFHDKMIADL  189
              F  + A    +   A     P  +LD NAWWGP+  K   D S+RP ++ F   MI DL
Sbjct  104   FFATITAVVAVVIYFALLRAPPLPELDLNAWWGPDSLKTKQDDSVRPMKLKFKKPMIQDL  163

Query  190   RYRLNNHRKPVPPLEGIGFEYGFNSNILDGWLQYWAEEYPFKEREIFLNKYPQYVTNIQG  369
             +  L   RK  PPLEG+GFEYGFNSN +D WL+YWAEEY F+ERE F N+Y  Y T IQG
Sbjct  164   QQYLKTTRKFAPPLEGVGFEYGFNSNNMDSWLKYWAEEYNFEERERFFNQYESYRTLIQG  223

Query  370   LDIHFIKYRQKAYAHQEIVPLLILHGWPGSVREFYETIPHLTSVNKSRDFAVEVIAPTLP  549
             L+IHFI  + +  A  E+VP+L+LHGWPGSVREFYE IP LT+V+K+R+FA+E+I P+LP
Sbjct  224   LNIHFIHVKPQVPAGVEVVPMLLLHGWPGSVREFYEAIPLLTAVDKTRNFALELIIPSLP  283

Query  550   GFGYSDAAVRPGLGMHEIAVVFSNLMKRLGYKKYYVQGGDWGAFIGSSIATSFPNEVLGY  729
             G+G+SDAAVRPGLG  E+AVV  NLM RLGYK+YY+QGGDWGA I ++++T FPNEVLGY
Sbjct  284   GYGWSDAAVRPGLGAAEVAVVMKNLMNRLGYKQYYLQGGDWGAVICTALSTLFPNEVLGY  343

Query  730   HTNLGLSLTQKALSLTLFGSLFPSPVVDPALADRTDLLSGHIRDAMITEFGYMHIQATKP  909
             HTN+  +++  A +L    SL+P+  ++P L DR   LS      M  E GYMHIQATKP
Sbjct  344   HTNMLFNMSPAATALEWLFSLWPTLAIEPELVDRAYPLSKTYSHLM-EEMGYMHIQATKP  402

Query  910   DTLGIAMTDSPGGLLAYFLQLISTGTRSYFLNLEDGGIDKYYTRDQLLDNIMMYWAPNSI  1089
             DT+G+A++DSP GLLAY L+  ST T    ++ +DGG+ K++T++QL+DN+M+YW+  SI
Sbjct  403   DTVGVALSDSPAGLLAYILEKFSTWTDRNLISSKDGGLTKHFTKEQLVDNLMVYWSTQSI  462

Query  1090  TTSFRIYAESLNRRTLALGISEIPTPVPTITIHASDEIAYQSPLLLRTKFTNLLYTTNID  1269
             TTS R+YAES N+R LAL +  IPT VP   I A  EI+YQ P++LR KF NLL+TT ++
Sbjct  463   TTSMRLYAESFNKRHLALELDSIPTTVPVWGIQAKHEISYQPPVILRLKFPNLLHTTVLE  522

Query  1270  TGGHFLALEMPILFANDVLNAIAEFRNWH  1356
              GGHF A ++P +FA DVL  ++ FR+WH
Sbjct  523   EGGHFFAFQLPKIFAEDVLKGVSAFRDWH  551


> ta_transcript53251_1
Length=624

 Score =   517 bits (1331),  Expect = 2e-177, Method: Compositional matrix adjust.
 Identities = 245/449 (55%), Positives = 321/449 (71%), Gaps = 1/449 (0%)
 Frame = +1

Query  10    LFRLVVASCLTIELLASQNTEPAYKLDPNAWWGPECTKYHNDTSIRPFEIVFHDKMIADL  189
              F  + A    +   A     P  +LD NAWWGP+  K   D S+RP ++ F   MI DL
Sbjct  104   FFATITAVVAVVIYFALLRAPPLPELDLNAWWGPDSLKTKQDDSVRPMKLKFKKPMIQDL  163

Query  190   RYRLNNHRKPVPPLEGIGFEYGFNSNILDGWLQYWAEEYPFKEREIFLNKYPQYVTNIQG  369
             +  L   RK  PPLEG+GFEYGFNSN +D WL+YWAEEY F+ERE F N+Y  Y T IQG
Sbjct  164   QQYLKTTRKFAPPLEGVGFEYGFNSNNMDSWLKYWAEEYNFEERERFFNQYESYRTLIQG  223

Query  370   LDIHFIKYRQKAYAHQEIVPLLILHGWPGSVREFYETIPHLTSVNKSRDFAVEVIAPTLP  549
             L+IHFI  + +  A  E+VP+L+LHGWPGSVREFYE IP LT+V+K+R+FA+E+I P+LP
Sbjct  224   LNIHFIHVKPQVPAGVEVVPMLLLHGWPGSVREFYEAIPLLTAVDKTRNFALELIIPSLP  283

Query  550   GFGYSDAAVRPGLGMHEIAVVFSNLMKRLGYKKYYVQGGDWGAFIGSSIATSFPNEVLGY  729
             G+G+SDAAVRPGLG  E+AVV  NLM RLGYK+YY+QGGDWGA I ++++T FPNEVLGY
Sbjct  284   GYGWSDAAVRPGLGAAEVAVVMKNLMNRLGYKQYYLQGGDWGAVICTALSTLFPNEVLGY  343

Query  730   HTNLGLSLTQKALSLTLFGSLFPSPVVDPALADRTDLLSGHIRDAMITEFGYMHIQATKP  909
             HTN+  +++  A +L    SL+P+  ++P L DR   LS      M  E GYMHIQATKP
Sbjct  344   HTNMLFNMSPAATALEWLFSLWPTLAIEPELVDRAYPLSKTYSHLM-EEMGYMHIQATKP  402

Query  910   DTLGIAMTDSPGGLLAYFLQLISTGTRSYFLNLEDGGIDKYYTRDQLLDNIMMYWAPNSI  1089
             DT+G+A++DSP GLLAY L+  ST T    ++ +DGG+ K++T++QL+DN+M+YW+  SI
Sbjct  403   DTVGVALSDSPAGLLAYILEKFSTWTDRNLISSKDGGLTKHFTKEQLVDNLMVYWSTQSI  462

Query  1090  TTSFRIYAESLNRRTLALGISEIPTPVPTITIHASDEIAYQSPLLLRTKFTNLLYTTNID  1269
```

```
             TTS R+YAES N+R LAL +  IPT VP   I A  EI+YQ P++LR KF NLL+TT ++
Sbjct  463   TTSMRLYAESFNKRHLALELDSIPTTVPVWGIQAKHEISYQPPVILRLKFPNLLHTTVLE  522

Query  1270  TGGHFLALEMPILFANDVLNAIAEFRNWH  1356
              GGHF A ++P +FA DVL  ++ FR+WH
Sbjct  523   EGGHFFAFQLPKIFAEDVLKGVSAFRDWH  551


> ta_transcript29071_1
Length=520

 Score =   432 bits (1110),  Expect = 1e-145, Method: Compositional matrix adjust.
 Identities = 218/423 (52%), Positives = 284/423 (67%), Gaps = 22/423 (5%)
 Frame = +1

Query  88    DPNAWWGPECTKYHNDTSIRPFEIVFHDKMIADLRYRLNNHRKPVPPLEGIGFEYGFNSN  267
             D   WWGP+  + H DTSIRP +I F +KMI DL+ RL       PPLE  GF YGFNS+
Sbjct  43    DIQEWWGPKSLQGHEDTSIRPAKIHFENKMIKDLQGRLRKTISFQPPLEDSGFTYGFNSD  102

Query  268   ILDGWLQYWAEEYPFKEREIFLNKYPQYVTNIQGLDIHFIKYRQKAYAHQEIVPLLILHG  447
              +  WL+YWAEEYPFKERE +LN++PQ+ TNIQGLDIHFI  + K   H+ ++PLL+LHG
Sbjct  103   SIGYWLKYWAEEYPFKEREAYLNQFPQFKTNIQGLDIHFIHVKPKVQTHKNVIPLLLLHG  162

Query  448   WPGSVREFYETIPHLTSVNKSRDFAVEVIAPTLPGFGYSDAAVRPGLGMHEIAVVFSNLM  627
             W GS+REFY+ IP LT+ +K RDFAVE+IAP LPGFG+SD  +R GLG  ++A++  NLM
Sbjct  163   WAGSIREFYDAIPLLTADSKDRDFAVELIAPCLPGFGFSDPTLRQGLGAAQMAIIMRNLM  222

Query  628   KRLGYKKYYVQGGDWGAFIGSSIATSFPNEVLGYHTNLGLSLTQKALSLTLFGSLFPSPV  807
              RLG+K++Y+QGGDWG FIGS IAT FP EVLG+HTN  L +T  A +L    SL P
Sbjct  223   HRLGFKRFYIQGGDWGGFIGSDIATLFPQEVLGFHTNWAL-VTSSAPALN---SLQP---  275

Query  808   VDPALADRTDLLSGHIRDAMITEFGYMHIQATKPDTLGIAMTDSPGGLLAYFLQLISTGT  987
             V P + + T               GY+HI AT+PDT+GIA+TDSP GLLA+ L+  ST
Sbjct  276   VAPLIMEET---------------GYLHIAATRPDTVGIALTDSPAGLLAFLLEKFSTSV  320

Query  988   RSYFLNLEDGGIDKYYTRDQLLDNIMMYWAPNSITTSFRIYAESLNRRTLALGISEIPTP  1167
             +     L DGG+   +T + L+D++M YW   S+ TS R+YAE+ N++  ALG+   PT
Sbjct  321   KHENRQLADGGLHNTFTPETLIDDVMFYWTTRSMPTSLRLYAENFNKKYQALGVERTPTT  380

Query  1168  VPTITIHASDEIAYQSPLLLRTKFTNLLYTTNIDTGGHFLALEMPILFANDVLNAIAEFR  1347
             VPT       E+ +QS  +L+TK+ NLL  T++D GGHF ALE P  F+ +VL A+  FR
Sbjct  381   VPTWVTQGKYELTHQSAEILKTKYQNLLNATSLDFGGHFFALEQPKFFSENVLTALKAFR  440

Query  1348  NWH  1356
              WH
Sbjct  441   IWH  443


> ta_transcript45224_1
Length=598

 Score =   405 bits (1042),  Expect = 3e-134, Method: Compositional matrix adjust.
 Identities = 198/424 (47%), Positives = 271/424 (64%), Gaps = 23/424 (5%)
 Frame = +1

Query  85    LDPNAWWGPECTKYHNDTSIRPFEIVFHDKMIADLRYRLNNHRKPVPPLEGIGFEYGFNS  264
             +D   WWGP+  + + DTSIR  +I F   M+ DL+ RL  H    PPLE  GF YGFNS
Sbjct  46    IDLQEWWGPKDLQGNEDTSIRSAKIHFSKSMVRDLKQRLKQHVPFQPPLEDSGFNYGFNS  105

Query  265   NILDGWLQYWAEEYPFKEREIFLNKYPQYVTNIQGLDIHFIKYRQKAYAHQEIVPLLILH  444
               +D W++YW+EEYPF+ERE FLN++PQ+ TNIQGLD+HFI  +     ++ ++PLLILH
Sbjct  106   GAMDYWVKYWSEEYPFEEREAFLNQFPQFKTNIQGLDMHFIHVKPNVTGNKPVIPLLILH  165

Query  445   GWPGSVREFYETIPHLTSVNKSRDFAVEVIAPTLPGFGYSDAAVRPGLGMHEIAVVFSNL  624
             GWPGS+REFY  IP LT+ +  RDFAVEV+ P LPGFG+SD   + GLG  ++AV+  NL
Sbjct  166   GWPGSIREFYGAIPLLTADSPDRDFAVEVVVPCLPGFGFSDGTSKIGLGAVQMAVILRNL  225

Query  625   MKRLGYKKYYVQGGDWGAFIGSSIATSFPNEVLGYHTNLGLSLTQKALSLTLFGSLFPSP  804
             M RLG+K++Y QGGDWG  IGS IAT FP E LG+H N G+ L+   L+     SL P
Sbjct  226   MHRLGHKQFYTQGGDWGGIIGSYIATIFPEENLGFHANWGVVLSSVGLN-----SLKPQA  280

Query  805   VVDPALADRTDLLSGHIRDAMITEFGYMHIQATKPDTLGIAMTDSPGGLLAYFLQLISTG  984
              +         LL G          GY+H+ ATKPDT+G+A+TDSP GLLA+ L+  S
Sbjct  281   EI--------VLLKG----------GYLHLAATKPDTVGMALTDSPTGLLAFILEKFSGA  322

Query  985   TRSYFLNLEDGGIDKYYTRDQLLDNIMMYWAPNSITTSFRIYAESLNRRTLALGISEIPT  1164
               +   +L DGG+   +    L+D++M YW    IT + R+YAE+ N++T ALGI E  +
```

```
Sbjct  323   VTNANNDLADGGLRNTFPAVALIDDLMFYWTERKITNTLRLYAETFNKKTRALGIDEARS  382

Query  1165  PVPTITIHASDEIAYQSPLLLRTKFTNLLYTTNIDTGGHFLALEMPILFANDVLNAIAEF  1344
             PVP        E   Q+P +L+TK+ NLL+  ++  G HFLALE+P ++ ++V N +  F
Sbjct  383   PVPIWVTQGKAEFTKQTPEILKTKYDNLLHAESLQVGNHFLALELPQVYTDNVFNGLKAF  442

Query  1345  RNWH  1356
              ++H
Sbjct  443   IDFH  446


> ta_transcript58145_1
Length=369

 Score =   318 bits (816),  Expect = 1e-103, Method: Compositional matrix adjust.
 Identities = 160/289 (55%), Positives = 206/289 (71%), Gaps = 3/289 (1%)
 Frame = +1

Query  496   SVNKSRDFAVEVIAPTLPGFGYSDAAVRPGLGMHEIAVVFSNLMKRLGYKKYYVQGGDWG  675
             +V+K+R+FA+E+I P+LPGFG+SDAAVRPGLG  EIAV+  NLM RLGYK+YY+QGGD G
Sbjct  1     AVDKTRNFALELIIPSLPGFGWSDAAVRPGLGAAEIAVIMRNLMHRLGYKQYYIQGGDAG  60

Query  676   AFIGSSIATSFPNEVLGYHTNL--GLSLTQKALSLTLFGSLFPSPVVDPALADRTDLLSG  849
             A IG  + T FP EVLGYHTN    +S + KAL L    S +PS  V+P L DR   L
Sbjct  61    ATIGKIMITLFPKEVLGYHTNFLFYVSSSPKALLLEWIYSFWPSLFVEPELVDRMYPLK-  119

Query  850   HIRDAMITEFGYMHIQATKPDTLGIAMTDSPGGLLAYFLQLISTGTRSYFLNLEDGGIDK  1029
                  +I E GY HIQATKPDT+G+A++DSP GLLAY L+  STGT   F +L DGG+ K
Sbjct  120   KTYSFLIEETGYFHIQATKPDTVGVALSDSPAGLLAYILEKFSTGTNRNFRSLPDGGLTK  179

Query  1030  YYTRDQLLDNIMMYWAPNSITTSFRIYAESLNRRTLALGISEIPTPVPTITIHASDEIAY  1209
             Y+T++QL+DN+M+YW+  S+TTS R YAE+ N+R L L    IPT VPT  + A  E++Y
Sbjct  180   YFTKEQLIDNLMVYWSTQSVTTSMRYYAETFNKRHLGLKFDSIPTTVPTWGMQAKHEMSY  239

Query  1210  QSPLLLRTKFTNLLYTTNIDTGGHFLALEMPILFANDVLNAIAEFRNWH  1356
             Q   +LR KF NL+  T ++ GGHF+AL+MP  FA+DVL ++A FR WH
Sbjct  240   QPASILRLKFLNLVNLTVLEDGGHFMALQMPDTFADDVLKSVAAFREWH  288


> ta_transcript58144_1
Length=440

 Score =   319 bits (817),  Expect = 9e-103, Method: Compositional matrix adjust.
 Identities = 160/289 (55%), Positives = 206/289 (71%), Gaps = 3/289 (1%)
 Frame = +1

Query  496   SVNKSRDFAVEVIAPTLPGFGYSDAAVRPGLGMHEIAVVFSNLMKRLGYKKYYVQGGDWG  675
             +V+K+R+FA+E+I P+LPGFG+SDAAVRPGLG  EIAV+  NLM RLGYK+YY+QGGD G
Sbjct  1     AVDKTRNFALELIIPSLPGFGWSDAAVRPGLGAAEIAVIMRNLMHRLGYKQYYIQGGDAG  60

Query  676   AFIGSSIATSFPNEVLGYHTNL--GLSLTQKALSLTLFGSLFPSPVVDPALADRTDLLSG  849
             A IG  + T FP EVLGYHTN    +S + KAL L    S +PS  V+P L DR   L
Sbjct  61    ATIGKIMITLFPKEVLGYHTNFLFYVSSSPKALLLEWIYSFWPSLFVEPELVDRMYPLK-  119

Query  850   HIRDAMITEFGYMHIQATKPDTLGIAMTDSPGGLLAYFLQLISTGTRSYFLNLEDGGIDK  1029
                  +I E GY HIQATKPDT+G+A++DSP GLLAY L+  STGT   F +L DGG+ K
Sbjct  120   KTYSFLIEETGYFHIQATKPDTVGVALSDSPAGLLAYILEKFSTGTNRNFRSLPDGGLTK  179

Query  1030  YYTRDQLLDNIMMYWAPNSITTSFRIYAESLNRRTLALGISEIPTPVPTITIHASDEIAY  1209
             Y+T++QL+DN+M+YW+  S+TTS R YAE+ N+R L L    IPT VPT  + A  E++Y
Sbjct  180   YFTKEQLIDNLMVYWSTQSVTTSMRYYAETFNKRHLGLKFDSIPTTVPTWGMQAKHEMSY  239

Query  1210  QSPLLLRTKFTNLLYTTNIDTGGHFLALEMPILFANDVLNAIAEFRNWH  1356
             Q   +LR KF NL+  T ++ GGHF+AL+MP  FA+DVL ++A FR WH
Sbjct  240   QPASILRLKFLNLVNLTVLEDGGHFMALQMPDTFADDVLKSVAAFREWH  288


> ta_transcript35275_1
Length=728

 Score =   136 bits (343),  Expect = 1e-33, Method: Compositional matrix adjust.
 Identities = 140/344 (41%), Positives = 178/344 (52%), Gaps = 4/344 (1%)
 Frame = -2

Query  1124  FRDSA*ILNEVVILFGAQYIMILSNN*SRV*YLSMPPSSRFKK*LRVPVEINWRKYASRP  945
             F  S * L +VV+L  AQ I+ LS++     + S  PS+  +    V VE   R  A+RP
Sbjct  280   FTASL*TLIDVVMLLLAQ*IITLSSSWFSENFWSRFPSAACRCWGLVQVENFSRM*AARP  339
```

```
Query  944   PGESVIAIPRVSGLVACMCMYPNSVIIASLMCPDNRSVRSANAGSTTGDGNREPNRVKDK  765
              GESV A P VSGLVACMC+ P S    +          +++  STT +GN +P +  +
Sbjct  340   AGESVSATPIVSGLVACMCVNPVSWSRKARWSVSGARRGTSSGRSTTQEGNTDPIK**NS  399

Query  764   AFCVSDNPKLV**PRTSLGNEVAIEEPIKAPQSPP*T*YFL*PSLFIKLLKTTAISCIPS  585
             A     +  LV  P T++G+ VA+ +P   P SPP  *Y L*P   I+L  TTAI   P
Sbjct  400   ACL*MTSGVLVWSPSTAVGSSVAMLDPTA*PTSPPCM*YPL*PMRCIRLCMTTAIWTGPR  459

Query  584   PGLTAASEYPNPGRVGAITSTAKSLLLFTLVKCGIVS*NSLTDPGQP*RINNGTIS*WA*  405
             P  TA SEYP PGR+G ITS A       ++  G++S* SL DPG P  I+ GT
Sbjct  460   PERTAPSEYPKPGRLGTITSNANLYPSRGVISFGMIS*YSLKDPGHPCSISRGTTVTPLS  519

Query  404   AFCLYLIKCMSNPCMLVTYWGYLFKNISLSLKGYSSAQY*SQPSKILELKPYSNPIPSNG  225
             +    LIKC+S+PC+LV    YL +  S S K Y   Q  S   + + LKPY  P  SNG
Sbjct  520   SAGRTLIKCISSPCILVL**LYLLR*SSRSRKLYLFFQ*SSICVRYIVLKPYVEPESSNG  579

Query  224   GTGlr*llsrylrsaiilSWKTISKGRMLVSLWYFVHSG--PHQ  99
             G   R    R  RS  I S   IS G M VS    V SG  PH
Sbjct  580   GVKGRW*RRRSRRSFTITSSNLISNGLMEVS--GLVSSGYEPHH  621


> ta_transcript53802_1
Length=503

 Score = 67.4 bits (163),  Expect = 3e-11, Method: Compositional matrix adjust.
 Identities = 48/156 (31%), Positives = 75/156 (48%), Gaps = 0/156 (0%)
 Frame = +3

Query  273  RWLASILGRRVSL*GKGNILEQIPPICDQHTGIGHTFY*VQTEGLCPSRNSSVIDSSRLA  452
            R +  +LG+ VSL       + +  + DQ TG G+TFY   T       +      +RLA
Sbjct  140  RAVDPVLGQPVSLQAA*GFSQPVSAVQDQCTGAGYTFYVG*TAEPTRQGSGPTTPDARLA  199

Query  453  RICEGILRNNTTLDKCK*KQRFCCRSNSSYSTRVWIFRRCGQTRARYARNRCSLQ*LNEK  632
             +  G+LR ++  D    ++ FC RS  + S   W+ RRC  +R     N    +  +E+
Sbjct  200  GVHSGVLRGDSDSDS*LSQK*FCNRSRGALSAWFWVLRRCSSSRNGRQSNCIHHEEPDEQ  259

Query  633  AWL*EILCSRRRLGCFYWFLNSNFISQ*SPWLSYQL  740
            AWL  +L  RRRLG  +W      +   SP +S++L
Sbjct  260  AWLPAVLHPRRRLGRPHWQCYVCVVPTRSPRISHEL  295


************************************************************************************************
*******************************************************************
Query= gi|261245098:126-2039 Bombyx mori juvenile hormone epoxide hydrolase-like protein 3
(Jheh-lp3), mRNA

Length=1914
                                                                      Score     E
Sequences producing significant alignments:                          (Bits)  Value

  ta_transcript53250_1                                                  459   7e-152
  ta_transcript53251_1                                                  458   8e-152
  ta_transcript35275_1                                                  412   1e-132
  ta_transcript29071_1                                                  366   8e-118
  ta_transcript45224_1                                                  345   8e-109
  ta_transcript58145_1                                                  315   3e-100
  ta_transcript58144_1                                                  316   2e-99
  ta_transcript59199_1                                                  129   9e-31
  ta_transcript59195_1                                                  129   1e-30


> ta_transcript53250_1
Length=647

 Score =   459 bits (1180),  Expect = 7e-152, Method: Compositional matrix adjust.
 Identities = 226/447 (51%), Positives = 307/447 (69%), Gaps = 3/447 (1%)
 Frame = +1

Query  31    KSFILITVIQVLSAYYLYRFFFIPPDMPALDTEQWWGPYPMDLDHDKSIRPLTIEFSDVM  210
             +  +    I  + A  +Y      P +P LD   WWGP  +    D S+RP+ ++F   M
Sbjct  100   RLLLFFATITAVVAVVIYFALLRAPPLPELDLNAWWGPDSLKTKQDDSVRPMKLKFKKPM  159

Query  211   INDlrerllhrrplqpplENVGFTYGFNPRYLNQVMEYWHNKYNFKEREQFLNQYDHFVT  390
             I DL++ L   R   PPLE VGF YGFN   ++  ++YW  +YNF+ERE+F NQY+ + T
Sbjct  160   IQDLQQYLKTTRKFAPPLEGVGFEYGFNSNNMDSWLKYWAEEYNFEERERFFNQYESYRT  219
```

```
Query  391   NIQGLDIHFMHIKPKNIAGLEVVPLIMLHGWPGSFREFYDVIPYLMAEQPEQKIAFEIIV  570
              IQGL+IHF+H+KP+  AG+EVVP+++LHGWPGS REFY+ IP L A    +  A E+I+
Sbjct  220   LIQGLNIHFIHVKPQVPAGVEVVPMLLLHGWPGSVREFYEAIPLLTAVDKTRNFALELII  279

Query  571   PSLPGFGYSQASVRPGLGPAEMAVIINNLMKRIGHDKYYIQGGDLGHTVGSIIATAFPEN  750
             PSLPG+G+S A+VRPGLG AE+AV++ NLM R+G+ +YY+QGGD G  + + ++T FP
Sbjct  280   PSLPGYGWSDAAVRPGLGAAEVAVVMKNLMNRLGYKQYYLQGGDWGAVICTALSTLFPNE  339

Query  751   VLGFHTNFPVLLSHRA-ALLYITCGSLIPSLIETKELQPRLYPLSEHWSRLIEESGYMHI  927
             VLG+HTN    +S  A AL ++   SL P+L    EL  R YPLS+ +S L+EE GYMHI
Sbjct  340   VLGYHTNMLFNMSPAATALEWLF--SLWPTLAIEPELVDRAYPLSKTYSHLMEEMGYMHI  397

Query  928   QATKPETVGVGLSDSPAGLAAYILEKFSTWTNPGNKKAGDGNLLKKFSLNQLLDNLMIYW  1107
             QATKP+TVGV LSDSPAGL AYILEKFSTWT+     + DG L K F+  QL+DNLM+YW
Sbjct  398   QATKPDTVGVALSDSPAGLLAYILEKFSTWTDRNLISSKDGGLTKHFTKEQLVDNLMVYW  457

Query  1108  ATNTVTTSMRVYAESFNEKQNFLIFDRIPTNVPTWGIKFKYELMYQPDTALSFKYKKYLQ  1287
             +T ++TTSMR+YAESFN++   L  D IPT VP WGI+ K+E+ YQP   L  K+   L
Sbjct  458   STQSITTSMRLYAESFNKRHLALELDSIPTTVPVWGIQAKHEISYQPPVILRLKFPNLLH  517

Query  1288  STVVEDGGHFAAMEYPELMAKDIIQAV  1368
             +TV+E+GGHF A + P++ A+D+++ V
Sbjct  518   TTVLEEGGHFFAFQLPKIFAEDVLKGV  544


> ta_transcript53251_1
Length=624

 Score =   458 bits (1178),  Expect = 8e-152, Method: Compositional matrix adjust.
 Identities = 226/447 (51%), Positives = 307/447 (69%), Gaps = 3/447 (1%)
 Frame = +1

Query  31    KSFILITVIQVLSAYYLYRFFFIPPDMPALDTEQWWGPYPMDLDHDKSIRPLTIEFSDVM  210
             +  +    I  + A  +Y      P +P LD   WWGP  +    D S+RP+ ++F   M
Sbjct  100   RLLLFFATITAVVAVVIYFALLRAPPLPELDLNAWWGPDSLKTKQDDSVRPMKLKFKKPM  159

Query  211   INDlrerllhrrplqpplENVGFTYGFNPRYLNQVMEYWHNKYNFKEREQFLNQYDHFVT  390
             I DL++ L   R   PPLE VGF YGFN   ++  ++YW  +YNF+ERE+F NQY+ + T
Sbjct  160   IQDLQQYLKTTRKFAPPLEGVGFEYGFNSNNMDSWLKYWAEEYNFEERERFFNQYESYRT  219

Query  391   NIQGLDIHFMHIKPKNIAGLEVVPLIMLHGWPGSFREFYDVIPYLMAEQPEQKIAFEIIV  570
              IQGL+IHF+H+KP+  AG+EVVP+++LHGWPGS REFY+ IP L A    +  A E+I+
Sbjct  220   LIQGLNIHFIHVKPQVPAGVEVVPMLLLHGWPGSVREFYEAIPLLTAVDKTRNFALELII  279

Query  571   PSLPGFGYSQASVRPGLGPAEMAVIINNLMKRIGHDKYYIQGGDLGHTVGSIIATAFPEN  750
             PSLPG+G+S A+VRPGLG AE+AV++ NLM R+G+ +YY+QGGD G  + + ++T FP
Sbjct  280   PSLPGYGWSDAAVRPGLGAAEVAVVMKNLMNRLGYKQYYLQGGDWGAVICTALSTLFPNE  339

Query  751   VLGFHTNFPVLLSHRA-ALLYITCGSLIPSLIETKELQPRLYPLSEHWSRLIEESGYMHI  927
             VLG+HTN    +S  A AL ++   SL P+L    EL  R YPLS+ +S L+EE GYMHI
Sbjct  340   VLGYHTNMLFNMSPAATALEWLF--SLWPTLAIEPELVDRAYPLSKTYSHLMEEMGYMHI  397

Query  928   QATKPETVGVGLSDSPAGLAAYILEKFSTWTNPGNKKAGDGNLLKKFSLNQLLDNLMIYW  1107
             QATKP+TVGV LSDSPAGL AYILEKFSTWT+     + DG L K F+  QL+DNLM+YW
Sbjct  398   QATKPDTVGVALSDSPAGLLAYILEKFSTWTDRNLISSKDGGLTKHFTKEQLVDNLMVYW  457

Query  1108  ATNTVTTSMRVYAESFNEKQNFLIFDRIPTNVPTWGIKFKYELMYQPDTALSFKYKKYLQ  1287
             +T ++TTSMR+YAESFN++   L  D IPT VP WGI+ K+E+ YQP   L  K+   L
Sbjct  458   STQSITTSMRLYAESFNKRHLALELDSIPTTVPVWGIQAKHEISYQPPVILRLKFPNLLH  517

Query  1288  STVVEDGGHFAAMEYPELMAKDIIQAV  1368
             +TV+E+GGHF A + P++ A+D+++ V
Sbjct  518   TTVLEEGGHFFAFQLPKIFAEDVLKGV  544


> ta_transcript35275_1
Length=728

 Score =   412 bits (1058),  Expect = 1e-132, Method: Compositional matrix adjust.
 Identities = 313/622 (50%), Positives = 377/622 (61%), Gaps = 9/622 (1%)
 Frame = -2

Query  1913  YQYLAKSFSRSSGLTCGAYLSTGTPFLSTTNLLKFHLMKSPIVPLCAFFRNFQSGCALSP  1734
             +QYLAK  ++SSG T GA LSTGTPFLS  NL KFHLMKSPIVPL   F+NF +GCA SP
Sbjct  29    HQYLAKYGTKSSGSTVGANLSTGTPFLSIINLEKFHLMKSPIVPL*ERFKNFHNGCAFSP  88
```

```
Query  1733   FTSTFSNRSNLTPLSFVKFRMSLDVPGSWPLNWLHGKASIRRPFWWYFscsslsc**lv-  1557
              FTSTF N+SN TP S  K  M+    PGS P N L G A+IR P    +   S      +
Sbjct  89    FTSTFWNKSNFTPRSLAKLNMASGPPGSCPPN*LQGNANIRSPLSTLYLSYSSLSCL*LF  148

Query  1556   vVRPH*EATLTMRSTFPL*SDSLTSRPFIDLTVNSYTVLAVSWRGGVSLDSCFSNFQ--N  1383
               VRPH +ATLTM +T PL*  S+T  P + +TVNS  VLAVS  GG     CF++    N
Sbjct  149   CVRPHCDATLTMTTTCPL*RSSVTFLPCMSVTVNS*IVLAVSHIGGSGF--CFADLFP*N  206

Query  1382   F*NLITACMISLAISSGYSMAAK*PPSSTTVLCKYFLYLKLNAVSGWYISSYLNFIPHVG  1203
                N  TA  IS A ++G S+AAK PPSST V  + F+Y     +SG  I S LN I  VG
Sbjct  207   SKNFPTASKISSASTAGNSIAAKWPPSSTAVD*R*FVYFCFKTMSGV*IMSCLNLIQAVG  266

Query  1202   TFVGILSKIRKFCFSLNDSAYTLMLVVTVLVAQYIIRLSnn*fkenffKRFPSPAFLLPG  1023
              T VGI S  +    F+   S  TL+ VV +L+AQ II LS++ F ENF+ RFPS A    G
Sbjct  267   TGVGISSICKAKLFT--ASL*TLIDVVMLLLAQ*IITLSSSWFSENFWSRFPSAACRCWG  324

Query  1022   FVQVENFSRM*AARPAGESDSPTPTVSGFVA*MCMYPDSSMSLDQCSDSGYRRGCNSFVS  843
               VQVENFSRM*AARPAGES S TP VSG VA MC+ P S      + S SG RRG +S  S
Sbjct  325   LVQVENFSRM*AARPAGESVSATPIVSGLVACMCVNPVSWSRKARWSVSGARRGTSSGRS  384

Query  842    I-RDGIKEPQVIYKRAAL*DRSTGKFV*NPNTFSGNAVAMMEPTVCPRSPPCM*YLSWPI  666
                ++G  +P      A L*  ++G  V +P+T  G++VAM++PT  P SPPCM*Y   P+
Sbjct  385   TTQEGNTDPIK**NSACL*-MTSGVLVWSPSTAVGSSVAMLDPTA*PTSPPCM*YPL*PM  443

Query  665    LFMRLLMITAISAGPKPGLTEACE*PKPGKLGTMISKAIFCSGCSAIKYGMTS*NSLNEP  486
                +RL M TAI  GP+P  T   E PKPG+LGT+ S A        I +GM S* SL +P
Sbjct  444   RCIRLCMTTAIWTGPRPERTAPSEYPKPGRLGTITSNANLYPSRGVISFGMIS*YSLKDP  503

Query  485    GHPCSMIRGTTSRPAMFLGFICMKCMSKPWILVTK*SYWFKNCSLSLKLYLLCQYSITWF  306
              GHPCS+ RGTT  P    G   +KC+S P ILV  * Y  +  S S KLYL  Q S
Sbjct  504   GHPCSISRGTTVTPLSSAGRTLIKCISSPCILVL**LYLLR*SSRSRKLYLFFQ*SSICV  563

Query  305    KYLGLKPYVNPTFSKggcrglrwrsrsrrSFIITSENSIVSGLIDLSWSKSIGYGPHHCS  126
              +Y+ LKPYV P  S GG +G   R RSRRSF ITS N I +GL+++S   S GY PHHCS
Sbjct  564   RYIVLKPYVEPESSNGGVKGRW*RRRSRRSFTITSSNLISNGLMEVSGLVSSGYEPHHCS  623

Query  125    VSSAGISGGMKKKRYR*YALST  60
               S  G SG       Y  YA +T
Sbjct  624   KSICGTSGCDVISSYMTYASTT  645


> ta_transcript29071_1
Length=520

 Score =   366 bits (940),  Expect = 8e-118, Method: Compositional matrix adjust.
 Identities = 198/416 (48%), Positives = 271/416 (65%), Gaps = 22/416 (5%)
 Frame = +1

Query  121    DTEQWWGPYPMDLDHDKSIRPLTIEFSDVMINDlrerllhrrplqpplENVGFTYGFNPR  300
              D ++WWGP  +    D SIRP  I F + MI DL+ RL      QPPLE+ GFTYGFN
Sbjct  43    DIQEWWGPKSLQGHEDTSIRPAKIHFENKMIKDLQGRLRKTISFQPPLEDSGFTYGFNSD  102

Query  301    YLNQVMEYWHNKYNFKEREQFLNQYDHFVTNIQGLDIHFMHIKPKNIAGLEVVPLIMLHG  480
               +   ++YW  +Y FKERE +LNQ+  F TNIQGLDIHF+H+KPK      V+PL++LHG
Sbjct  103   SIGYWLKYWAEEYPFKEREAYLNQFPQFKTNIQGLDIHFIHVKPKVQTHKNVIPLLLLHG  162

Query  481    WPGSFREFYDVIPYLMAEQPEQKIAFEIIVPSLPGFGYSQASVRPGLGPAEMAVIINNLM  660
              W GS REFYD IP L A+  ++  A E+I P LPGFG+S  ++R GLG A+MA+I+ NLM
Sbjct  163   WAGSIREFYDAIPLLTADSKDRDFAVELIAPCLPGFGFSDPTLRQGLGAAQMAIIMRNLM  222

Query  661    KRIGHDKYYIQGGDLGHTVGSIIATAFPENVLGFHTNFPVLLSHRAALLYITCGSLIPSL  840
               R+G  ++YIQGGD G  +GS IAT FP+ VLGFHTN+ ++ S   AL
Sbjct  223   HRLGFKRFYIQGGDWGGFIGSDIATLFPQEVLGFHTNWALVTSSAPAL------------  270

Query  841    IETKELQPRLYPLSEHWSRLIEESGYMHIQATKPETVGVGLSDSPAGLAAYILEKFSTWT  1020
                   LQP + PL      ++EE+GY+HI AT+P+TVG+ L+DSPAGL A++LEKFST
Sbjct  271   ---NSLQP-VAPL------IMEETGYLHIAATRPDTVGIALTDSPAGLLAFLLEKFSTSV  320

Query  1021   NPGNKKAGDGNLLKKFSLNQLLDNLMIYWATNTVTTSMRVYAESFNEKQNFLIFDRIPTN  1200
                N++  DG L    F+    L+D++M YW T ++ TS+R+YAE+FN+K    L  +R PT
Sbjct  321   KHENRQLADGGLHNTFTPETLIDDVMFYWTTRSMPTSLRLYAENFNKKYQALGVERTPTT  380

Query  1201   VPTWGIKFKYELMYQPDTALSFKYKKYLQSTVVEDGGHFAAMEYPELMAKDIIQAV  1368
              VPTW  + KYEL +Q     L  KY+  L +T ++ GGHF A+E P+  +++++ A+
Sbjct  381   VPTWVTQGKYELTHQSAEILKTKYQNLLNATSLDFGGHFFALEQPKFFSENVLTAL  436
```

```
> ta_transcript45224_1
Length=598

 Score =   345 bits (886),  Expect = 8e-109, Method: Compositional matrix adjust.
 Identities = 180/418 (43%), Positives = 249/418 (60%), Gaps = 23/418 (6%)
 Frame = +1

Query  115   ALDTEQWWGPYPMDLDHDKSIRPLTIEFSDVMINDlrerllhrrplqpplENVGFTYGFN  294
             A+D ++WWGP  +  + D SIR   I FS  M+ DL++RL    P QPPLE+ GF YGFN
Sbjct  45    AIDLQEWWGPKDLQGNEDTSIRSAKIHFSKSMVRDLKQRLKQHVPFQPPLEDSGFNYGFN  104

Query  295   PRYLNQVMEYWHNKYNFKEREQFLNQYDHFVTNIQGLDIHFMHIKPKNIAGLEVVPLIML  474
                ++  ++YW  +Y F+ERE FLNQ+  F TNIQGLD+HF+H+KP       V+PL++L
Sbjct  105   SGAMDYWVKYWSEEYPFEEREAFLNQFPQFKTNIQGLDMHFIHVKPNVTGNKPVIPLLIL  164

Query  475   HGWPGSFREFYDVIPYLMAEQPEQKIAFEIIVPSLPGFGYSQASVRPGLGPAEMAVIINN  654
             HGWPGS REFY  IP L A+ P++  A E++VP LPGFG+S  + + GLG  +MAVI+ N
Sbjct  165   HGWPGSIREFYGAIPLLTADSPDRDFAVEVVVPCLPGFGFSDGTSKIGLGAVQMAVILRN  224

Query  655   LMKRIGHDKYYIQGGDLGHTVGSIIATAFPENVLGFHTNFPVLLSHRAALLYITCGSLIP  834
             LM R+GH ++Y QGGD G  +GS IAT FPE  LGFH N+ V+LS
Sbjct  225   LMHRLGHKQFYTQGGDWGGIIGSYIATIFPEENLGFHANWGVVLSS--------------  270

Query  835   SLIETKELQPRLYPLSEHWSRLIEESGYMHIQATKPETVGVGLSDSPAGLAAYILEKFST  1014
               +    L+P+          ++ + GY+H+ ATKP+TVG+ L+DSP GL A+ILEKFS
Sbjct  271   --VGLNSLKPQA-------EIVLLKGGYLHLAATKPDTVGMALTDSPTGLLAFILEKFSG  321

Query  1015  WTNPGNKKAGDGNLLKKFSLNQLLDNLMIYWATNTVTTSMRVYAESFNEKQNFLIFDRIP  1194
                  N    DG L   F    L+D+LM YW    +T ++R+YAE+FN+K   L  D
Sbjct  322   AVTNANNDLADGGLRNTFPAVALIDDLMFYWTERKITNTLRLYAETFNKKTRALGIDEAR  381

Query  1195  TNVPTWGIKFKYELMYQPDTALSFKYKKYLQSTVVEDGGHFAAMEYPELMAKDIIQAV  1368
             + VP W  + K E   Q    L  KY   L +  ++ G HF A+E P++   ++   +
Sbjct  382   SPVPIWVTQGKAEFTKQTPEILKTKYDNLLHAESLQVGNHFLALELPQVYTDNVFNGL  439


> ta_transcript58145_1
Length=369

 Score =   315 bits (808),  Expect = 3e-100, Method: Compositional matrix adjust.
 Identities = 157/281 (56%), Positives = 201/281 (72%), Gaps = 1/281 (0%)
 Frame = +1

Query  529   AEQPEQKIAFEIIVPSLPGFGYSQASVRPGLGPAEMAVIINNLMKRIGHDKYYIQGGDLG  708
             A    +  A E+I+PSLPGFG+S A+VRPGLG AE+AVI+ NLM R+G+ +YYIQGGD G
Sbjct  1     AVDKTRNFALELIIPSLPGFGWSDAAVRPGLGAAEIAVIMRNLMHRLGYKQYYIQGGDAG  60

Query  709   HTVGSIIATAFPENVLGFHTNFPVLLSHR-AALLYITCGSLIPSLIETKELQPRLYPLSE  885
              T+G I+ T FP+ VLG+HTNF   +S    ALL     S  PSL    EL  R+YPL +
Sbjct  61    ATIGKIMITLFPKEVLGYHTNFLFYVSSSPKALLLEWIYSFWPSLFVEPELVDRMYPLKK  120

Query  886   HWSRLIEESGYMHIQATKPETVGVGLSDSPAGLAAYILEKFSTWTNPGNKKAGDGNLLKK  1065
              +S LIEE+GY HIQATKP+TVGV LSDSPAGL AYILEKFST TN   +    DG L K
Sbjct  121   TYSFLIEETGYFHIQATKPDTVGVALSDSPAGLLAYILEKFSTGTNRNFRSLPDGGLTKY  180

Query  1066  FSLNQLLDNLMIYWATNTVTTSMRVYAESFNEKQNFLIFDRIPTNVPTWGIKFKYELMYQ  1245
             F+  QL+DNLM+YW+T +VTTSMR YAE+FN++   L FD IPT VPTWG++ K+E+ YQ
Sbjct  181   FTKEQLIDNLMVYWSTQSVTTSMRYYAETFNKRHLGLKFDSIPTTVPTWGMQAKHEMSYQ  240

Query  1246  PDTALSFKYKKYLQSTVVEDGGHFAAMEYPELMAKDIIQAV  1368
             P + L  K+   +  TV+EDGGHF A++ P+  A D++++V
Sbjct  241   PASILRLKFLNLVNLTVLEDGGHFMALQMPDTFADDVLKSV  281


> ta_transcript58144_1
Length=440

 Score =   316 bits (810),  Expect = 2e-99, Method: Compositional matrix adjust.
 Identities = 157/281 (56%), Positives = 201/281 (72%), Gaps = 1/281 (0%)
 Frame = +1

Query  529   AEQPEQKIAFEIIVPSLPGFGYSQASVRPGLGPAEMAVIINNLMKRIGHDKYYIQGGDLG  708
             A    +  A E+I+PSLPGFG+S A+VRPGLG AE+AVI+ NLM R+G+ +YYIQGGD G
Sbjct  1     AVDKTRNFALELIIPSLPGFGWSDAAVRPGLGAAEIAVIMRNLMHRLGYKQYYIQGGDAG  60

Query  709   HTVGSIIATAFPENVLGFHTNFPVLLSHR-AALLYITCGSLIPSLIETKELQPRLYPLSE  885
              T+G I+ T FP+ VLG+HTNF   +S    ALL     S  PSL    EL  R+YPL +
```

```
Sbjct  61    ATIGKIMITLFPKEVLGYHTNFLFYVSSSPKALLLEWIYSFWPSLFVEPELVDRMYPLKK  120

Query  886   HWSRLIEESGYMHIQATKPETVGVGLSDSPAGLAAYILEKFSTWTNPGNKKAGDGNLLKK  1065
              +S LIEE+GY HIQATKP+TVGV LSDSPAGL AYILEKFST TN   +   DG L K
Sbjct  121   TYSFLIEETGYFHIQATKPDTVGVALSDSPAGLLAYILEKFSTGTNRNFRSLPDGGLTKY  180

Query  1066  FSLNQLLDNLMIYWATNTVTTSMRVYAESFNEKQNFLIFDRIPTNVPTWGIKFKYELMYQ  1245
             F+  QL+DNLM+YW+T +VTTSMR YAE+FN++   L FD IPT VPTWG++ K+E+ YQ
Sbjct  181   FTKEQLIDNLMVYWSTQSVTTSMRYYAETFNKRHLGLKFDSIPTTVPTWGMQAKHEMSYQ  240

Query  1246  PDTALSFKYKKYLQSTVVEDGGHFAAMEYPELMAKDIIQAV  1368
             P + L  K+   +  TV+EDGGHF A++ P+  A D++++V
Sbjct  241   PASILRLKFLNLVNLTVLEDGGHFMALQMPDTFADDVLKSV  281


> ta_transcript59199_1
Length=662

 Score =   129 bits (325),  Expect = 9e-31, Method: Compositional matrix adjust.
 Identities = 86/155 (55%), Positives = 100/155 (65%), Gaps = 1/155 (1%)
 Frame = -2

Query  1913  YQYLAKSFSRSSGLTCGAYLSTGTPFLSTTNLLKFHLMKSPIVPLCAFFRNFQSGCALSP  1734
             YQY ++  ++S G   G   STGTP LS  NL+KFHL+K P VP C  F+NFQSG ALSP
Sbjct  420   YQYFSRDLTKSIGSVLGP*RSTGTPSLSMMNLVKFHLIKLPRVPPCLCFKNFQSGWALSP  479

Query  1733  FTSTFSNRSNLTPLSFVKFRMSLDVPGSWPLNWLHGKASIRRPFWWYFscss-lsc**lv  1557
             FTSTFSN SNLT  S  K      +PGSWP NWLHG A IR P    +S +S  SC *L+
Sbjct  480   FTSTFSNMSNLTLRSAKKHCSCSGLPGSWPANWLHGNAKIRSPLLSAYSSNS*FSCL*LL  539

Query  1556  vVRPH*EATLTMRSTFPL*SDSLTSRPFIDLTVNS  1452
              VRPH +AT T+R T PL + S TS P    TVNS
Sbjct  540   AVRPHCDATFTIRHT*PLYTSSFTSSPLRFFTVNS  574


> ta_transcript59195_1
Length=695

 Score =   129 bits (325),  Expect = 1e-30, Method: Compositional matrix adjust.
 Identities = 86/155 (55%), Positives = 100/155 (65%), Gaps = 1/155 (1%)
 Frame = -2

Query  1913  YQYLAKSFSRSSGLTCGAYLSTGTPFLSTTNLLKFHLMKSPIVPLCAFFRNFQSGCALSP  1734
             YQY ++  ++S G   G   STGTP LS  NL+KFHL+K P VP C  F+NFQSG ALSP
Sbjct  420   YQYFSRDLTKSIGSVLGP*RSTGTPSLSMMNLVKFHLIKLPRVPPCLCFKNFQSGWALSP  479

Query  1733  FTSTFSNRSNLTPLSFVKFRMSLDVPGSWPLNWLHGKASIRRPFWWYFscss-lsc**lv  1557
             FTSTFSN SNLT  S  K      +PGSWP NWLHG A IR P    +S +S  SC *L+
Sbjct  480   FTSTFSNMSNLTLRSAKKHCSCSGLPGSWPANWLHGNAKIRSPLLSAYSSNS*FSCL*LL  539

Query  1556  vVRPH*EATLTMRSTFPL*SDSLTSRPFIDLTVNS  1452
              VRPH +AT T+R T PL + S TS P    TVNS
Sbjct  540   AVRPHCDATFTIRHT*PLYTSSFTSSPLRFFTVNS  574


***************************************************************************************************
**********************************************************************

Query= gi|261245100:118-1602 Bombyx mori juvenile hormone epoxide hydrolase-like protein 4
(Jheh-lp4), mRNA

Length=1485
                                                                      Score     E
Sequences producing significant alignments:                          (Bits)  Value

  ta_transcript53250_1                                                  387   4e-126
  ta_transcript53251_1                                                  386   6e-126
  ta_transcript29071_1                                                  341   6e-110
  ta_transcript45224_1                                                  325   1e-102
  ta_transcript58145_1                                                  266   4e-83
  ta_transcript58144_1                                                  267   2e-82
  ta_transcript35275_1                                                  139   3e-34


> ta_transcript53250_1
Length=647
```

```
 Score =   387 bits (994),  Expect = 4e-126, Method: Compositional matrix adjust.
 Identities = 196/471 (42%), Positives = 291/471 (62%), Gaps = 13/471 (3%)
 Frame = +1

Query  97    VFGVFAK-------IISYLFVIKALFTIYCIYLVYVSLTNVPDLPKVDVNVRWGVDN--N  249
             VF +F K       +++ L +  A  T     ++Y +L   P LP++D+N  WG D+
Sbjct  83    VFKLFVKFKCRKIIMMARLLLFFATITAVVAVVIYFALLRAPPLPELDLNAWWGPDSLKT  142

Query  250   THDTRIRPYRVIFSDAMESEIRALFEDYRLMERKIKSFKNTAWTYGVHSDAFAQFFSHWI  429
               D  +RP ++ F   M  +++   +  R   +     +   + YG +S+    +  +W
Sbjct  143   KQDDSVRPMKLKFKKPMIQDLQQYLKTTR---KFAPPLEGVGFEYGFNSNNMDSWLKYWA  199

Query  430   FKYKFRERVKFLNKYDHFLTNIQGLDIHFVRVKPKADKNVKVVPLLLLHGWPGSVREFYE  609
              +Y F ER +F N+Y+ + T IQGL+IHF+ VKP+    V+VVP+LLLHGWPGSVREFYE
Sbjct  200   EEYNFEERERFFNQYESYRTLIQGLNIHFIHVKPQVPAGVEVVPMLLLHGWPGSVREFYE  259

Query  610   AIPLLTTPRPDYDFVFEVIAPSLPGFVFSEAPTRPGLDTYEMAIIMRNLMRRLGYTQYYI  789
             AIPLLT      +F  E+I PSLPG+ +S+A  RPGL   E+A++M+NLM RLGY QYY+
Sbjct  260   AIPLLTAVDKTRNFALELIIPSLPGYGWSDAAVRPGLGAAEVAVVMKNLMNRLGYKQYYL  319

Query  790   QGGDFGHMIGSHIATIFPSEVLGFHTNFPVNtsklslltwllggllwPSYFGNGIEDRMY  969
             QGGD+G +I + ++T+FP+EVLG+HTN   N S  +     L  L         + DR Y
Sbjct  320   QGGDWGAVICTALSTLFPNEVLGYHTNMLFNMSPAATALEWLFSLWPTLAIEPELVDRAY  379

Query  970   PLKDKMEFYLEETGYSHLQSTKPDTIGIVLTDSPVALGSYILDRFMIFTNHTNKFEDEGG  1149
             PL       +EE GY H+Q+TKPDT+G+ L+DSP  L +YIL++F  +T+       +GG
Sbjct  380   PLSKTYSHLMEEMGYMHIQATKPDTVGVALSDSPAGLLAYILEKFSTWTDRNLISSKDGG  439

Query  1150  IDKYYDFDKLLDNIMLYWVSGSITTSLRIYKETFAGSRLN-NLAQVPTSVPTWALRLKYE  1326
             + K++  ++L+DN+M+YW + SITTS+R+Y E+F    L   L  +PT+VP W ++ K+E
Sbjct  440   LTKHFTKEQLVDNLMVYWSTQSITTSMRLYAESFNKRHLALELDSIPTTVPVWGIQAKHE  499

Query  1327  LFQHPDYMLRWKYTNLLGSTNLDYGGHFAAFERPKDFSDDVFKAVKAFRNF  1479
             +   P  +LR K+ NLL +T L+ GGHF AF+ PK F++DV K V AFR++
Sbjct  500   ISYQPPVILRLKFPNLLHTTVLEEGGHFFAFQLPKIFAEDVLKGVSAFRDW  550


> ta_transcript53251_1
Length=624

 Score =   386 bits (991),  Expect = 6e-126, Method: Compositional matrix adjust.
 Identities = 196/471 (42%), Positives = 291/471 (62%), Gaps = 13/471 (3%)
 Frame = +1

Query  97    VFGVFAK-------IISYLFVIKALFTIYCIYLVYVSLTNVPDLPKVDVNVRWGVDN--N  249
             VF +F K       +++ L +  A  T     ++Y +L   P LP++D+N  WG D+
Sbjct  83    VFKLFVKFKCRKIIMMARLLLFFATITAVVAVVIYFALLRAPPLPELDLNAWWGPDSLKT  142

Query  250   THDTRIRPYRVIFSDAMESEIRALFEDYRLMERKIKSFKNTAWTYGVHSDAFAQFFSHWI  429
               D  +RP ++ F   M  +++   +  R   +     +   + YG +S+    +  +W
Sbjct  143   KQDDSVRPMKLKFKKPMIQDLQQYLKTTR---KFAPPLEGVGFEYGFNSNNMDSWLKYWA  199

Query  430   FKYKFRERVKFLNKYDHFLTNIQGLDIHFVRVKPKADKNVKVVPLLLLHGWPGSVREFYE  609
              +Y F ER +F N+Y+ + T IQGL+IHF+ VKP+    V+VVP+LLLHGWPGSVREFYE
Sbjct  200   EEYNFEERERFFNQYESYRTLIQGLNIHFIHVKPQVPAGVEVVPMLLLHGWPGSVREFYE  259

Query  610   AIPLLTTPRPDYDFVFEVIAPSLPGFVFSEAPTRPGLDTYEMAIIMRNLMRRLGYTQYYI  789
             AIPLLT      +F  E+I PSLPG+ +S+A  RPGL   E+A++M+NLM RLGY QYY+
Sbjct  260   AIPLLTAVDKTRNFALELIIPSLPGYGWSDAAVRPGLGAAEVAVVMKNLMNRLGYKQYYL  319

Query  790   QGGDFGHMIGSHIATIFPSEVLGFHTNFPVNtsklslltwllggllwPSYFGNGIEDRMY  969
             QGGD+G +I + ++T+FP+EVLG+HTN   N S  +     L  L         + DR Y
Sbjct  320   QGGDWGAVICTALSTLFPNEVLGYHTNMLFNMSPAATALEWLFSLWPTLAIEPELVDRAY  379

Query  970   PLKDKMEFYLEETGYSHLQSTKPDTIGIVLTDSPVALGSYILDRFMIFTNHTNKFEDEGG  1149
             PL       +EE GY H+Q+TKPDT+G+ L+DSP  L +YIL++F  +T+       +GG
Sbjct  380   PLSKTYSHLMEEMGYMHIQATKPDTVGVALSDSPAGLLAYILEKFSTWTDRNLISSKDGG  439

Query  1150  IDKYYDFDKLLDNIMLYWVSGSITTSLRIYKETFAGSRLN-NLAQVPTSVPTWALRLKYE  1326
             + K++  ++L+DN+M+YW + SITTS+R+Y E+F    L   L  +PT+VP W ++ K+E
Sbjct  440   LTKHFTKEQLVDNLMVYWSTQSITTSMRLYAESFNKRHLALELDSIPTTVPVWGIQAKHE  499

Query  1327  LFQHPDYMLRWKYTNLLGSTNLDYGGHFAAFERPKDFSDDVFKAVKAFRNF  1479
             +   P  +LR K+ NLL +T L+ GGHF AF+ PK F++DV K V AFR++
Sbjct  500   ISYQPPVILRLKFPNLLHTTVLEEGGHFFAFQLPKIFAEDVLKGVSAFRDW  550
```

```
> ta_transcript29071_1
Length=520

 Score =   341 bits (875),  Expect = 6e-110, Method: Compositional matrix adjust.
 Identities = 191/435 (44%), Positives = 266/435 (61%), Gaps = 35/435 (8%)
 Frame = +1

Query  190   TNVPDLPKVDVNVRWGVDN--NTHDTRIRPYRVIFSDAMESEIRALFEDYRLMERKIKSF  363
              N+  L   D+   WG  +     DT IRP ++ F + M  +++      RL  RK  SF
Sbjct  34    CNITVLNCSDIQEWWGPKSLQGHEDTSIRPAKIHFENKMIKDLQG-----RL--RKTISF  86

Query  364   K----NTAWTYGVHSDAFAQFFSHWIFKYKFRERVKFLNKYDHFLTNIQGLDIHFVRVKP  531
             +    ++ +TYG +SD+   +  +W  +Y F+ER  +LN++  F TNIQGLDIHF+ VKP
Sbjct  87    QPPLEDSGFTYGFNSDSIGYWLKYWAEEYPFKEREAYLNQFPQFKTNIQGLDIHFIHVKP  146

Query  532   KADKNVKVVPLLLLHGWPGSVREFYEAIPLLTTPRPDYDFVFEVIAPSLPGFVFSEAPTR  711
             K   +  V+PLLLLHGW GS+REFY+AIPLLT    D DF  E+IAP LPGF FS+   R
Sbjct  147   KVQTHKNVIPLLLLHGWAGSIREFYDAIPLLTADSKDRDFAVELIAPCLPGFGFSDPTLR  206

Query  712   PGLDTYEMAIIMRNLMRRLGYTQYYIQGGDFGHMIGSHIATIFPSEVLGFHTNFPVNtsk  891
              GL   +MAIIMRNLM RLG+ ++YIQGGD+G  IGS IAT+FP EVLGFHTN+ +
Sbjct  207   QGLGAAQMAIIMRNLMHRLGFKRFYIQGGDWGGFIGSDIATLFPQEVLGFHTNWAL----  262

Query  892   lslltwllggllwPSYFGNGIEDRMYPLKDKMEFYLEETGYSHLQSTKPDTIGIVLTDSP  1071
                          P+         +  L+      +EETGY H+ +T+PDT+GI LTDSP
Sbjct  263   --------VTSSAPA---------LNSLQPVAPLIMEETGYLHIAATRPDTVGIALTDSP  305

Query  1072  VALGSYILDRFMIFTNHTNKFEDEGGIDKYYDFDKLLDNIMLYWVSGSITTSLRIYKETF  1251
               L +++L++F     H N+   +GG+   +  + L+D++M YW + S+ TSLR+Y E F
Sbjct  306   AGLLAFLLEKFSTSVKHENRQLADGGLHNTFTPETLIDDVMFYWTTRSMPTSLRLYAENF  365

Query  1252  AGS-RLNNLAQVPTSVPTWALRLKYELFQHPDYMLRWKYTNLLGSTNLDYGGHFAAFERP  1428
                 +   + + PT+VPTW  + KYEL      +L+ KY NLL +T+LD+GGHF A E+P
Sbjct  366   NKKYQALGVERTPTTVPTWVTQGKYELTHQSAEILKTKYQNLLNATSLDFGGHFFALEQP  425

Query  1429  KDFSDDVFKAVKAFR  1473
             K FS++V  A+KAFR
Sbjct  426   KFFSENVLTALKAFR  440


> ta_transcript45224_1
Length=598

 Score =   325 bits (832),  Expect = 1e-102, Method: Compositional matrix adjust.
 Identities = 178/457 (39%), Positives = 262/457 (57%), Gaps = 29/457 (6%)
 Frame = +1

Query  118   IISYLFVIKALFTIYCIYLVYVSLTNVPDLPKVDVNVRWGVDN--NTHDTRIRPYRVIFS  291
             +++    ++ AL  I    L+  + T V +   +D+   WG  +     DT IR  ++ FS
Sbjct  15    VVTRPSIMDALLPILSSVLI-ANDTAVFNRSAIDLQEWWGPKDLQGNEDTSIRSAKIHFS  73

Query  292   DAMESEIRALFEDYRLMERKIKSFKNTAWTYGVHSDAFAQFFSHWIFKYKFRERVKFLNK  471
              +M  +++   + +   +  ++    ++ + YG +S A    +  +W  +Y F ER  FLN+
Sbjct  74    KSMVRDLKQRLKQHVPFQPPLE---DSGFNYGFNSGAMDYWVKYWSEEYPFEEREAFLNQ  130

Query  472   YDHFLTNIQGLDIHFVRVKPKADKNVKVVPLLLLHGWPGSVREFYEAIPLLTTPRPDYDF  651
             +  F TNIQGLD+HF+ VKP    N  V+PLL+LHGWPGS+REFY AIPLLT   PD DF
Sbjct  131   FPQFKTNIQGLDMHFIHVKPNVTGNKPVIPLLILHGWPGSIREFYGAIPLLTADSPDRDF  190

Query  652   VFEVIAPSLPGFVFSEAPTRPGLDTYEMAIIMRNLMRRLGYTQYYIQGGDFGHMIGSHIA  831
               EV+ P LPGF FS+  ++ GL    +MA+I+RNLM RLG+ Q+Y QGGD+G +IGS+IA
Sbjct  191   AVEVVVPCLPGFGFSDGTSKIGLGAVQMAVILRNLMHRLGHKQFYTQGGDWGGIIGSYIA  250

Query  832   TIFPSEVLGFHTNFPVNtsklslltwllggllwPSYFGNGIEDRMYPLKDKMEFYLEETG  1011
             TIFP E LGFH N+ V                    S  G      +  LK + E  L + G
Sbjct  251   TIFPEENLGFHANWGV----------------VLSSVG------LNSLKPQAEIVLLKGG  288

Query  1012  YSHLQSTKPDTIGIVLTDSPVALGSYILDRFMIFTNHTNKFEDEGGIDKYYDFDKLLDNI  1191
             Y HL +TKPDT+G+ LTDSP  L ++IL++F      + N    +GG+   +    L+D++
Sbjct  289   YLHLAATKPDTVGMALTDSPTGLLAFILEKFSGAVTNANNDLADGGLRNTFPAVALIDDL  348

Query  1192  MLYWVSGSITTSLRIYKETF-AGSRLNNLAQVPTSVPTWALRLKYELFQHPDYMLRWKYT  1368
             M YW    IT +LR+Y ETF    +R   + +  + VP W  + K E  +    +L+ KY
Sbjct  349   MFYWTERKITNTLRLYAETFNKKTRALGIDEARSPVPIWVTQGKAEFTKQTPEILKTKYD  408

Query  1369  NLLGSTNLDYGGHFAAFERPKDFSDDVFKAVKAFRNF  1479
```

```
             NLL + +L  G HF A E P+ ++D+VF  +KAF +F
Sbjct  409   NLLHAESLQVGNHFLALELPQVYTDNVFNGLKAFIDF  445


> ta_transcript58145_1
Length=369

 Score =   266 bits (681),  Expect = 4e-83, Method: Compositional matrix adjust.
 Identities = 137/281 (49%), Positives = 183/281 (65%), Gaps = 3/281 (1%)
 Frame = +1

Query  646   DFVFEVIAPSLPGFVFSEAPTRPGLDTYEMAIIMRNLMRRLGYTQYYIQGGDFGHMIGSH  825
             +F  E+I PSLPGF +S+A  RPGL   E+A+IMRNLM RLGY QYYIQGGD G  IG
Sbjct  7     NFALELIIPSLPGFGWSDAAVRPGLGAAEIAVIMRNLMHRLGYKQYYIQGGDAGATIGKI  66

Query  826   IATIFPSEVLGFHTNFPVNtsklslltwllggllw-PSYFGN-GIEDRMYPLKDKMEFYL  999
             + T+FP EVLG+HTNF    S       L     + PS F    + DRMYPLK    F +
Sbjct  67    MITLFPKEVLGYHTNFLFYVSSSPKALLLEWIYSFWPSLFVEPELVDRMYPLKKTYSFLI  126

Query  1000  EETGYSHLQSTKPDTIGIVLTDSPVALGSYILDRFMIFTNHTNKFEDEGGIDKYYDFDKL  1179
             EETGY H+Q+TKPDT+G+ L+DSP  L +YIL++F   TN   +   +GG+ KY+  ++L
Sbjct  127   EETGYFHIQATKPDTVGVALSDSPAGLLAYILEKFSTGTNRNFRSLPDGGLTKYFTKEQL  186

Query  1180  LDNIMLYWVSGSITTSLRIYKETFAGSRLN-NLAQVPTSVPTWALRLKYELFQHPDYMLR  1356
             +DN+M+YW + S+TTS+R Y ETF    L      +PT+VPTW ++ K+E+   P  +LR
Sbjct  187   IDNLMVYWSTQSVTTSMRYYAETFNKRHLGLKFDSIPTTVPTWGMQAKHEMSYQPASILR  246

Query  1357  WKYTNLLGSTNLDYGGHFAAFERPKDFSDDVFKAVKAFRNF  1479
              K+ NL+  T L+ GGHF A + P  F+DDV K+V AFR +
Sbjct  247   LKFLNLVNLTVLEDGGHFMALQMPDTFADDVLKSVAAFREW  287


> ta_transcript58144_1
Length=440

 Score =   267 bits (682),  Expect = 2e-82, Method: Compositional matrix adjust.
 Identities = 137/281 (49%), Positives = 183/281 (65%), Gaps = 3/281 (1%)
 Frame = +1

Query  646   DFVFEVIAPSLPGFVFSEAPTRPGLDTYEMAIIMRNLMRRLGYTQYYIQGGDFGHMIGSH  825
             +F  E+I PSLPGF +S+A  RPGL   E+A+IMRNLM RLGY QYYIQGGD G  IG
Sbjct  7     NFALELIIPSLPGFGWSDAAVRPGLGAAEIAVIMRNLMHRLGYKQYYIQGGDAGATIGKI  66

Query  826   IATIFPSEVLGFHTNFPVNtsklslltwllggllw-PSYFGN-GIEDRMYPLKDKMEFYL  999
             + T+FP EVLG+HTNF    S       L     + PS F    + DRMYPLK    F +
Sbjct  67    MITLFPKEVLGYHTNFLFYVSSSPKALLLEWIYSFWPSLFVEPELVDRMYPLKKTYSFLI  126

Query  1000  EETGYSHLQSTKPDTIGIVLTDSPVALGSYILDRFMIFTNHTNKFEDEGGIDKYYDFDKL  1179
             EETGY H+Q+TKPDT+G+ L+DSP  L +YIL++F   TN   +   +GG+ KY+  ++L
Sbjct  127   EETGYFHIQATKPDTVGVALSDSPAGLLAYILEKFSTGTNRNFRSLPDGGLTKYFTKEQL  186

Query  1180  LDNIMLYWVSGSITTSLRIYKETFAGSRLN-NLAQVPTSVPTWALRLKYELFQHPDYMLR  1356
             +DN+M+YW + S+TTS+R Y ETF    L      +PT+VPTW ++ K+E+   P  +LR
Sbjct  187   IDNLMVYWSTQSVTTSMRYYAETFNKRHLGLKFDSIPTTVPTWGMQAKHEMSYQPASILR  246

Query  1357  WKYTNLLGSTNLDYGGHFAAFERPKDFSDDVFKAVKAFRNF  1479
              K+ NL+  T L+ GGHF A + P  F+DDV K+V AFR +
Sbjct  247   LKFLNLVNLTVLEDGGHFMALQMPDTFADDVLKSVAAFREW  287


> ta_transcript35275_1
Length=728

 Score =   139 bits (349),  Expect = 3e-34, Method: Compositional matrix adjust.
 Identities = 162/442 (37%), Positives = 205/442 (46%), Gaps = 29/442 (7%)
 Frame = -2

Query  1460  TALNTSSLKSFGRSKAAK*PP*SKLVEPKRLVYFQRSI*SGCWKSSYFKRSAHVGTEVG-  1284
             TA   SS  + G S AAK PP S  V+ +  VYF     SG    S       VGT VG
Sbjct  212   TASKISSASTAGNSIAAKWPPSSTAVD*R*FVYFCFKTMSGV*IMSCLNLIQAVGTGVGI  271

Query  1283  ---T*ARLFNLEPAKVSL*ILSDVVILPETQYNIMlssnlsks*ylSMPPSSSNLLVWFV  1113
                  A+LF       SL* L DVV+L   Q  I LSS+     + S  PS++      V
Sbjct  272   SSICKAKLFT-----ASL*TLIDVVMLLLAQ*IITLSSSWFSENFWSRFPSAACRCWGLV  326

Query  1112  KIMNLSKIYDPKATGESVRTIPIVSGFVD*RCEYPVSSK*NSILSFRGYI--LSSMPLPK  939
             ++ N S++   +  GESV   PIVSG V   C  PVS    +  S  G     SS
```

```
Sbjct  327   QVENFSRM*AARPAGESVSATPIVSGLVACMCVNPVSWSRKARWSVSGARRGTSSGRSTT  386

Query  938   YEGHNNPPRSQVNKDSLDVLTGKFVWKPSTSEGKIVAIWDPIMCPKSPP*I*YWV*PSLR  759
              EG+ +P +    N    L + +G  VW PST+ G  VA+ DP    P SPP +*Y +*P
Sbjct  387   QEGNTDPIK**-NSACL*MTSGVLVWSPSTAVGSSVAMLDPTA*PTSPPCM*YPL*PMRC  445

Query  758   IKFRIIMAISYVSNPGRVGASENTKPGKLGAITSNTKS*SGLGVVRSGIAS*NSLTEPGQ  579
             I+   +  AI      P R    SE   KPG+LG ITSN        GV+   G+ S* SL +PG
Sbjct  446   IRLCMTTAIWTGPRPERTAPSEYPKPGRLGTITSNANLYPSRGVISFGMIS*YSLKDPGH  505

Query  578   PCRSRRGTTFTFLSAFGLTRTKCISKP*ILVKK*SYLFKNFTRSLNLYLNIQCEKNCANA  399
             PC    RGTT  T LS+  G T   KCIS P ILV    * YL  +   +RS   LYL  Q     C
Sbjct  506   PCSISRGTTVTPLSSAGRTLIKCISSPCILVL**LYLLR*SSRSRKLYLFFQ*SSIC---  562

Query  398   SEWTP*VQAVFLKLFIFLSMRR*SSKSALISD------SIASLNITL*GLILVSCVLLS-  240
                   V+ + LK ++          K             +I S N+    GL+ VS ++ S
Sbjct  563   ------VRYIVLKPYVEPESSNGGVKGRW*RRRSRRSFTITSSNLISNGLMEVSGLVSSG  616

Query  239   -TPHRTLTSTLGRSGTFVSDTY  177
               PH     S  G SG  V  +Y
Sbjct  617   YEPHHCSKSICGTSGCDVISSY  638
```

****

Query= gi|295424203:63-1451 Bombyx mori juvenile hormone epoxide hydrolase-like protein 5 (jheh-lp5), mRNA

Length=1389

```
                                                                      Score     E
Sequences producing significant alignments:                          (Bits)  Value

  ta_transcript53251_1                                                  539    0.0
  ta_transcript53250_1                                                  540    0.0
  ta_transcript29071_1                                                  423    2e-142
  ta_transcript45224_1                                                  418    2e-139
  ta_transcript58145_1                                                  330    3e-108
  ta_transcript58144_1                                                  331    2e-107
  ta_transcript35275_1                                                  194    9e-54
```

```
> ta_transcript53251_1
Length=624

 Score =   539 bits (1389),  Expect = 0.0, Method: Compositional matrix adjust.
 Identities = 249/433 (58%), Positives = 326/433 (75%), Gaps = 0/433 (0%)
 Frame = +1

Query  61    FNWLQPPKPPQLDPNAWWGPIELKGREDISIRPFEIKFEKKVIDDLKYRLKNHRPFAPPL  240
             F  L+ P   P+LD NAWWGP   LK ++D S+RP ++KF+K +I DL+   LK  R FAPPL
Sbjct  118   FALLRAPPLPELDLNAWWGPDSLKTKQDDSVRPMKLKFKKPMIQDLQQYLKTTRKFAPPL  177

Query  241   EGVTFEYGFNTAALEPWLKYWAEEYKFADREKFFNQFPHFKTSIKGLDIHFIRVKPQVSA  420
             EGV FEYGFN+   ++ WLKYWAEEY F +RE+FFNQ+   ++T I+GL+IHFI VKPQV A
Sbjct  178   EGVGFEYGFNSNNMDSWLKYWAEEYNFEERERFFNQYESYRTLIQGLNIHFIHVKPQVPA  237

Query  421   GVQTLPLLLLHGWPGSVREFYEAIPLLTSQNSGYDFAFEVIVPSLPGYGFSDAAVRPGLS  600
             GV+ +P+LLLHGWPGSVREFYEAIPLLT+ +    +FA E+I+PSLPGYG+SDAAVRPGL
Sbjct  238   GVEVVPMLLLHGWPGSVREFYEAIPLLTAVDKTRNFALELIIPSLPGYGWSDAAVRPGLG  297

Query  601   LPYVADIFRILMKRLGHDKFYIQGGDWGAAIASAMVTLFPEDVLGHHSNSAVTQHPHALL  780
                VA + + LM RLG+ ++Y+QGGDWGA I +A+ TLFP +VLG+H+N       P A
Sbjct  298   AAEVAVVMKNLMNRLGYKQYYLQGGDWGAVICTALSTLFPNEVLGYHTNMLFNMSPAATA  357

Query  781   RTLLGALIPSLIVEDHLAERMYPLSKHLAYLLEEFGYFHLQATKPDTVGVSLTDSPSGLL  960
                L +L P+L +E  L +R YPLSK  ++L+EE GY H+QATKPDTVGV+L+DSP+GLL
Sbjct  358   LEWLFSLWPTLAIEPELVDRAYPLSKTYSHLMEEMGYMHIQATKPDTVGVALSDSPAGLL  417

Query  961   AYILEKFAVWTRKEHKFKSDGGLGFRFSKEKLIDNLMVYWITNSITSSMRFYSENMSNKF  1140
             AYILEKF+ WT +       DGGL    F+KE+L+DNLMVYW T SIT+SMR Y+E+ + +
Sbjct  418   AYILEKFSTWTDRNLISSKDGGLTKHFTKEQLVDNLMVYWSTQSITTSMRLYAESFNKRH  477

Query  1141  REMNLEAFTSPVPTWALQARDELVYQPPAVLRAKYPNLLNVTVLDDGGHFLALELPEVFA  1320
               + L++   + VP W +QA+ E+ YQPP +LR K+PNLL+ TVL++GGHF A +LP++FA
```

```
Sbjct  478   LALELDSIPTTVPVWGIQAKHEISYQPPVILRLKFPNLLHTTVLEEGGHFFAFQLPKIFA  537

Query  1321  EDVFKAVHAFKKW  1359
             EDV K V AF+ W
Sbjct  538   EDVLKGVSAFRDW  550


> ta_transcript53250_1
Length=647

 Score =   540 bits (1391),  Expect = 0.0, Method: Compositional matrix adjust.
 Identities = 249/433 (58%), Positives = 326/433 (75%), Gaps = 0/433 (0%)
 Frame = +1

Query  61    FNWLQPPKPPQLDPNAWWGPIELKGREDISIRPFEIKFEKKVIDDLKYRLKNHRPFAPPL  240
             F  L+ P  P+LD NAWWGP  LK ++D S+RP ++KF+K +I DL+  LK  R FAPPL
Sbjct  118   FALLRAPPLPELDLNAWWGPDSLKTKQDDSVRPMKLKFKKPMIQDLQQYLKTTRKFAPPL  177

Query  241   EGVTFEYGFNTAALEPWLKYWAEEYKFADREKFFNQFPHFKTSIKGLDIHFIRVKPQVSA  420
             EGV FEYGFN+  ++ WLKYWAEEY F +RE+FFNQ+  ++T I+GL+IHFI VKPQV A
Sbjct  178   EGVGFEYGFNSNNMDSWLKYWAEEYNFEERERFFNQYESYRTLIQGLNIHFIHVKPQVPA  237

Query  421   GVQTLPLLLLHGWPGSVREFYEAIPLLTSQNSGYDFAFEVIVPSLPGYGFSDAAVRPGLS  600
             GV+ +P+LLLHGWPGSVREFYEAIPLLT+ +   +FA E+I+PSLPGYG+SDAAVRPGL
Sbjct  238   GVEVVPMLLLHGWPGSVREFYEAIPLLTAVDKTRNFALELIIPSLPGYGWSDAAVRPGLG  297

Query  601   LPYVADIFRILMKRLGHDKFYIQGGDWGAAIASAMVTLFPEDVLGHHSNSAVTQHPHALL  780
                VA + + LM RLG+ ++Y+QGGDWGA I +A+ TLFP +VLG+H+N      P A
Sbjct  298   AAEVAVVMKNLMNRLGYKQYYLQGGDWGAVICTALSTLFPNEVLGYHTNMLFNMSPAATA  357

Query  781   RTLLGALIPSLIVEDHLAERMYPLSKHLAYLLEEFGYFHLQATKPDTVGVSLTDSPSGLL  960
                L +L P+L +E  L +R YPLSK  ++L+EE GY H+QATKPDTVGV+L+DSP+GLL
Sbjct  358   LEWLFSLWPTLAIEPELVDRAYPLSKTYSHLMEEMGYMHIQATKPDTVGVALSDSPAGLL  417

Query  961   AYILEKFAVWTRKEHKFKSDGGLGFRFSKEKLIDNLMVYWITNSITSSMRFYSENMSNKF  1140
             AYILEKF+ WT +      DGGL   F+KE+L+DNLMVYW T SIT+SMR Y+E+ + +
Sbjct  418   AYILEKFSTWTDRNLISSKDGGLTKHFTKEQLVDNLMVYWSTQSITTSMRLYAESFNKRH  477

Query  1141  REMNLEAFTSPVPTWALQARDELVYQPPAVLRAKYPNLLNVTVLDDGGHFLALELPEVFA  1320
               + L++  + VP W +QA+ E+ YQPP +LR K+PNLL+ TVL++GGHF A +LP++FA
Sbjct  478   LALELDSIPTTVPVWGIQAKHEISYQPPVILRLKFPNLLHTTVLEEGGHFFAFQLPKIFA  537

Query  1321  EDVFKAVHAFKKW  1359
             EDV K V AF+ W
Sbjct  538   EDVLKGVSAFRDW  550


> ta_transcript29071_1
Length=520

 Score =   423 bits (1088),  Expect = 2e-142, Method: Compositional matrix adjust.
 Identities = 214/425 (50%), Positives = 281/425 (66%), Gaps = 21/425 (5%)
 Frame = +1

Query  97    DPNAWWGPIELKGREDISIRPFEIKFEKKVIDDLKYRLKNHRPFAPPLEGVTFEYGFNTA  276
             D   WWGP  L+G ED SIRP +I FE K+I DL+ RL+    F PPLE   F YGFN+
Sbjct  43    DIQEWWGPKSLQGHEDTSIRPAKIHFENKMIKDLQGRLRKTISFQPPLEDSGFTYGFNSD  102

Query  277   ALEPWLKYWAEEYKFADREKFFNQFPHFKTSIKGLDIHFIRVKPQVSAGVQTLPLLLLHG  456
             ++  WLKYWAEEY F +RE + NQFP FKT+I+GLDIHFI VKP+V      +PLLLLHG
Sbjct  103   SIGYWLKYWAEEYPFKEREAYLNQFPQFKTNIQGLDIHFIHVKPKVQTHKNVIPLLLLHG  162

Query  457   WPGSVREFYEAIPLLTSQNSGYDFAFEVIVPSLPGYGFSDAAVRPGLSLPYVADIFRILM  636
             W GS+REFY+AIPLLT+ +   DFA E+I P LPG+GFSD  +R GL    +A I R LM
Sbjct  163   WAGSIREFYDAIPLLTADSKDRDFAVELIAPCLPGFGFSDPTLRQGLGAAQMAIIMRNLM  222

Query  637   KRLGHDKFYIQGGDWGAAIASAMVTLFPEDVLGHHSNSAVTQHPHALLRTLLGALIPSLI  816
              RLG  +FYIQGGDWG  I S + TLFP++VLG H+N A+       L +L
Sbjct  223   HRLGFKRFYIQGGDWGGFIGSDIATLFPQEVLGFHTNWALVTSSAPALNSL---------  273

Query  817   VEDHLAERMYPLSKHLAYLLEEFGYFHLQATKPDTVGVSLTDSPSGLLAYILEKFAVWTR  996
                   + + PL      ++EE GY H+ AT+PDTVG++LTDSP+GLLA++LEKF+   +
Sbjct  274   ------QPVAPL------IMEETGYLHIAATRPDTVGIALTDSPAGLLAFLLEKFSTSVK  321

Query  997   KEHKFKSDGGLGFRFSKEKLIDNLMVYWITNSITSSMRFYSENMSNKFREMNLEAFTSPV  1176
              E++  +DGGL   F+ E LID++M YW T S+ +S+R Y+EN + K++ + +E   + V
Sbjct  322   HENRQLADGGLHNTFTPETLIDDVMFYWTTRSMPTSLRLYAENFNKKYQALGVERTPTTV  381
```

```
Query  1177  PTWALQARDELVYQPPAVLRAKYPNLLNVTVLDDGGHFLALELPEVFAEDVFKAVHAFKK  1356
             PTW  Q + EL +Q   +L+ KY NLLN T LD GGHF ALE P+ F+E+V  A+ AF+
Sbjct  382   PTWVTQGKYELTHQSAEILKTKYQNLLNATSLDFGGHFFALEQPKFFSENVLTALKAFRI  441

Query  1357  WRSTH  1371
             W  TH
Sbjct  442   WHQTH  446


> ta_transcript45224_1
Length=598

 Score =   418 bits (1075),  Expect = 2e-139, Method: Compositional matrix adjust.
 Identities = 209/432 (48%), Positives = 272/432 (63%), Gaps = 22/432 (5%)
 Frame = +1

Query  94    LDPNAWWGPIELKGREDISIRPFEIKFEKKVIDDLKYRLKNHRPFAPPLEGVTFEYGFNT  273
             +D   WWGP +L+G ED SIR  +I F K ++ DLK RLK H PF PPLE   F YGFN+
Sbjct  46    IDLQEWWGPKDLQGNEDTSIRSAKIHFSKSMVRDLKQRLKQHVPFQPPLEDSGFNYGFNS  105

Query  274   AALEPWLKYWAEEYKFADREKFFNQFPHFKTSIKGLDIHFIRVKPQVSAGVQTLPLLLLH  453
              A++ W+KYW+EEY F +RE F NQFP FKT+I+GLD+HFI VKP V+     +PLL+LH
Sbjct  106   GAMDYWVKYWSEEYPFEEREAFLNQFPQFKTNIQGLDMHFIHVKPNVTGNKPVIPLLILH  165

Query  454   GWPGSVREFYEAIPLLTSQNSGYDFAFEVIVPSLPGYGFSDAAVRPGLSLPYVADIFRIL  633
             GWPGS+REFY AIPLLT+ +   DFA EV+VP LPG+GFSD   + GL    +A I R L
Sbjct  166   GWPGSIREFYGAIPLLTADSPDRDFAVEVVVPCLPGFGFSDGTSKIGLGAVQMAVILRNL  225

Query  634   MKRLGHDKFYIQGGDWGAAIASAMVTLFPEDVLGHHSNSAVTQHPHALLRTLLGALIPSL  813
             M RLGH +FY QGGDWG  I S + T+FPE+ LG H+N  V       L    L +L P
Sbjct  226   MHRLGHKQFYTQGGDWGGIIGSYIATIFPEENLGFHANWGV-----VLSSVGLNSLKPQA  280

Query  814   IVEDHLAERMYPLSKHLAYLLEEFGYFHLQATKPDTVGVSLTDSPSGLLAYILEKFAVWT  993
              +                 +L + GY HL ATKPDTVG++LTDSP+GLLA+ILEKF+
Sbjct  281   EI-----------------VLLKGGYLHLAATKPDTVGMALTDSPTGLLAFILEKFSGAV  323

Query  994   RKEHKFKSDGGLGFRFSKEKLIDNLMVYWITNSITSSMRFYSENMSNKFREMNLEAFTSP  1173
                +   +DGGL   F    LID+LM YW    IT+++R Y+E  + K R + ++   SP
Sbjct  324   TNANNDLADGGLRNTFPAVALIDDLMFYWTERKITNTLRLYAETFNKKTRALGIDEARSP  383

Query  1174  VPTWALQARDELVYQPPAVLRAKYPNLLNVTVLDDGGHFLALELPEVFAEDVFKAVHAFK  1353
             VP W  Q + E   Q P +L+ KY NLL+   L  G HFLALELP+V+ ++VF  + AF
Sbjct  384   VPIWVTQGKAEFTKQTPEILKTKYDNLLHAESLQVGNHFLALELPQVYTDNVFNGLKAFI  443

Query  1354  KWRSTHSKTDH*  1389
              +  + + T +*
Sbjct  444   DFHKSKASTSN*  455


> ta_transcript58145_1
Length=369

 Score =   330 bits (847),  Expect = 3e-108, Method: Compositional matrix adjust.
 Identities = 156/285 (55%), Positives = 213/285 (75%), Gaps = 2/285 (1%)
 Frame = +1

Query  523   DFAFEVIVPSLPGYGFSDAAVRPGLSLPYVADIFRILMKRLGHDKFYIQGGDWGAAIASA  702
             +FA E+I+PSLPG+G+SDAAVRPGL    +A I R LM RLG+ ++YIQGGD GA I
Sbjct  7     NFALELIIPSLPGFGWSDAAVRPGLGAAEIAVIMRNLMHRLGYKQYYIQGGDAGATIGKI  66

Query  703   MVTLFPEDVLGHHSNSA--VTQHPHALLRTLLGALIPSLIVEDHLAERMYPLSKHLAYLL  876
             M+TLFP++VLG+H+N    V+  P ALL   + + PSL VE  L +RMYPL K  ++L+
Sbjct  67    MITLFPKEVLGYHTNFLFYVSSSPKALLLEWIYSFWPSLFVEPELVDRMYPLKKTYSFLI  126

Query  877   EEFGYFHLQATKPDTVGVSLTDSPSGLLAYILEKFAVWTRKEHKFKSDGGLGFRFSKEKL  1056
             EE GYFH+QATKPDTVGV+L+DSP+GLLAYILEKF+  T +  +   DGGL   F+KE+L
Sbjct  127   EETGYFHIQATKPDTVGVALSDSPAGLLAYILEKFSTGTNRNFRSLPDGGLTKYFTKEQL  186

Query  1057  IDNLMVYWITNSITSSMRFYSENMSNKFREMNLEAFTSPVPTWALQARDELVYQPPAVLR  1236
             IDNLMVYW T S+T+SMR+Y+E  + +  +  ++  + VPTW +QA+ E+ YQP ++LR
Sbjct  187   IDNLMVYWSTQSVTTSMRYYAETFNKRHLGLKFDSIPTTVPTWGMQAKHEMSYQPASILR  246

Query  1237  AKYPNLLNVTVLDDGGHFLALELPEVFAEDVFKAVHAFKKWRSTH  1371
              K+ NL+N+TVL+DGGHF+AL++P+ FA+DV K+V AF++W   H
Sbjct  247   LKFLNLVNLTVLEDGGHFMALQMPDTFADDVLKSVAAFREWHKIH  291
```

```
> ta_transcript58144_1
Length=440

 Score =   331 bits (848),  Expect = 2e-107, Method: Compositional matrix adjust.
 Identities = 156/285 (55%), Positives = 213/285 (75%), Gaps = 2/285 (1%)
 Frame = +1

Query  523   DFAFEVIVPSLPGYGFSDAAVRPGLSLPYVADIFRILMKRLGHDKFYIQGGDWGAAIASA  702
             +FA E+I+PSLPG+G+SDAAVRPGL    +A I R LM RLG+ ++YIQGGD GA I
Sbjct  7     NFALELIIPSLPGFGWSDAAVRPGLGAAEIAVIMRNLMHRLGYKQYYIQGGDAGATIGKI  66

Query  703   MVTLFPEDVLGHHSNSA--VTQHPHALLRTLLGALIPSLIVEDHLAERMYPLSKHLAYLL  876
             M+TLFP++VLG+H+N    V+  P ALL   + +  PSL VE  L +RMYPL K  ++L+
Sbjct  67    MITLFPKEVLGYHTNFLFYVSSSPKALLLEWIYSFWPSLFVEPELVDRMYPLKKTYSFLI  126

Query  877   EEFGYFHLQATKPDTVGVSLTDSPSGLLAYILEKFAVWTRKEHKFKSDGGLGFRFSKEKL  1056
             EE GYFH+QATKPDTVGV+L+DSP+GLLAYILEKF+  T +  +   DGGL   F+KE+L
Sbjct  127   EETGYFHIQATKPDTVGVALSDSPAGLLAYILEKFSTGTNRNFRSLPDGGLTKYFTKEQL  186

Query  1057  IDNLMVYWITNSITSSMRFYSENMSNKFREMNLEAFTSPVPTWALQARDELVYQPPAVLR  1236
             IDNLMVYW T S+T+SMR+Y+E  + +   +  ++  + VPTW +QA+ E+ YQP ++LR
Sbjct  187   IDNLMVYWSTQSVTTSMRYYAETFNKRHLGLKFDSIPTTVPTWGMQAKHEMSYQPASILR  246

Query  1237  AKYPNLLNVTVLDDGGHFLALELPEVFAEDVFKAVHAFKKWRSTH  1371
              K+ NL+N+TVL+DGGHF+AL++P+ FA+DV K+V AF++W   H
Sbjct  247   LKFLNLVNLTVLEDGGHFMALQMPDTFADDVLKSVAAFREWHKIH  291


> ta_transcript35275_1
Length=728

 Score =   194 bits (494),  Expect = 9e-54, Method: Compositional matrix adjust.
 Identities = 180/415 (43%), Positives = 217/415 (52%), Gaps = 7/415 (2%)
 Frame = -2

Query  1340  TALKTSSANTSGSSRAKKCPPSSKTVTLRRFGYFARKTAGGW*TSSSLAWRAHVGTGDVK  1161
             TA K SSA+T+G+S A K PPSS  V  R F YF  KT  G *  S L     VGTG
Sbjct  212   TASKISSASTAGNSIAAKWPPSSTAVD*R*FVYFCFKTMSGV*IMSCLNLIQAVGTGVGI  271

Query  1160  ASKFISLNLLLMFSE*NLIELVIELVIQ*TIRLSISFSLLNLKPRPPSLLNLCSLRVQTA  981
             +S  I    L    S * LI++V+ L+ Q* I LS S+    N   R PS    C   VQ
Sbjct  272   SS--ICKAKLFTASL*TLIDVVMLLLAQ*IITLSSSWFSENFWSRFPSAACRCWGLVQVE  329

Query  980   NFSKIYARRPDGESVRETPTVSGFVACKWKYPNSSNKYARCFESGY---ILSAR*SSTIS  810
             NFS++ A RP GESV  TP VSG VAC    P S ++ AR   SG      S R  ST
Sbjct  330   NFSRM*AARPAGESVSATPIVSGLVACMCVNPVSWSRKARWSVSGARRGTSSGR--STTQ  387

Query  809   DGIKAPSRVLSRA*GCCVTAELL*CPRTSSGNRVTMAEAMAAPQSPPCM*NLS*PNLFIN  630
             +G   P +  + A     +  L+  P T+ G+ V M +  A P SPPCM*   *P   I
Sbjct  388   EGNTDPIK**NSACL*MTSGVLVWSPSTAVGSSVAMLDPTA*PTSPPCM*YPL*PMRCIR  447

Query  629   ILKISAT*GKDKPGLTAASEKPYPGRLGTMTSKAKSYPEF*LVSRGMAS*NSLTEPGQPC  450
             +   +A     +P  TA SE P PGRLGT+TS A  YP   ++S GM S* SL +PG PC
Sbjct  448   LCMTTAIWTGPRPERTAPSEYPKPGRLGTITSNANLYPSRGVISFGMIS*YSLKDPGHPC  507

Query  449   NRRRGRVWTPADT*GLTLMK*ISRPLMLVLKCGN*LKNFSRSANLYSSAQYFSQGSRAAV  270
             +  RG   TP  + G TL+K IS P +LVL     L+  SRS  LY   Q  S   R  V
Sbjct  508   SISRGTTVTPLSSAGRTLIKCISSPCILVL**LYLLR*SSRSRKLYLFFQ*SSICVRYIV  567

Query  269   LKPYSKVTPSSGGAKGR*FLRRYFKSSITFFSNLISKGLMLMSSLPFSSIGPHHA  105
             LKPY +   S+GG KGR   RR  +S     SNLIS GLM +S L  S   PHH
Sbjct  568   LKPYVEPESSNGGVKGRW*RRRSRRSFTITSSNLISNGLMEVSGLVSSGYEPHHC  622


*****************************************************************************************************
*************************************************************************

Query= gi|112983177:33-1745 Bombyx mori juvenile hormone esterase 1 (jhe1), mRNA

Length=1713
                                                                      Score     E
Sequences producing significant alignments:                          (Bits)  Value

  ta_transcript82619_1                                                  230   7e-65
  ta_transcript82614_1                                                  229   2e-64
```

```
  ta_transcript82616_1                                            224   1e-62
  ta_transcript82623_1                                            202   6e-57
  ta_transcript58915_1                                            205   7e-57
  ta_transcript58914_1                                            205   1e-56
  ta_transcript75783_1                                            189   8e-51
  ta_transcript48580_1                                            187   3e-50
  ta_transcript48579_1                                            188   4e-50
  ta_transcript48393_1                                            147   4e-37
  ta_transcript84821_1                                            146   7e-37
  ta_transcript84818_1                                            146   2e-36
  ta_transcript84816_1                                            145   1e-35
  ta_transcript29751_1                                            140   2e-35


> ta_transcript82619_1
Length=801

 Score =   230 bits (586),  Expect = 7e-65, Method: Compositional matrix adjust.
 Identities = 227/566 (40%), Positives = 267/566 (47%), Gaps = 45/566 (8%)
 Frame = -2

Query  1676  SKKFFISSCSLVRLVWWHKFGVIMSWYCSSRGVTGGHAVVSSHIGALHRIKFVTNVVIQY  1497
             SK   +SSCS+V LV +   G ++ +  +   + G H  V    GA   IK  T   I+
Sbjct  198   SKNRTMSSCSVVGLVSFVSLGEVI-FQ*ARCLLMGDHDRVE---GAPVLIKLTT*SFIRC  253

Query  1496  FI-SSSAFCDELVPDAEFTFSTYVKSSACPAP-LKCK*KACLNTL*PS*E---NWYRNTG  1332
             FI SSS F   L    E T +  V SS  PAP +  +  A L  L  S E   N Y   G
Sbjct  254   FICSSSEFLGTLT---ELTLNM*VISSTWPAPPV*LRPSARLTGL--SSELYANSYIYAG  308

Query  1331  ASPAADLFSANFMAGYL**LSVHILIKPSRSTLPLKYLWLI*FETKAIVSGVEEYSRLEG  1152
             A P   L SA F AGY   +S H     S    PLKY            SG      +
Sbjct  309   APPVFAL*SATFSAGYWKNVSEHNFKYSSMVGAPLKYFSETSLARSRATSGATLNKTVGD  368

Query  1151  TKIAGFSLILDTISICSNLCWNIWHSEFVKPIIKGMSLPRPCWTRIFGSSSNIVVIPGCG  972
                 G  LIL T+S+  NL   + HS FV PI  G+ LP+P ++ +  SS      PG G
Sbjct  369   NTNVGSFLILSTMSMSENLFLKLSHSIFVIPIRSGI*LPKPFFSNV*VSSGRTASTPGRG  428

Query  971   VSTTGKKVVRPI*SINILLASFSFSTGMATSFSCISSGLVELTPRMENKILLMDWEKIGD  792
              S TGKK   P+ S + L  +   S G++ S   I+SG V L  R    +    + KIGD
Sbjct  429   DSMTGKKNAMPLASCSALFTAMIASRGISASCWWIASGSVALMFRALR*LSAAYFAKIGD  488

Query  791   VVKKVDVPAPLINIALETKPSVALDKVRR*aaaapalwpAKVTLSGLPPKALAFLFTQRS  612
             VV       PLI IAL   P    D V +  A APAL PA+VT SG PPK LA   TQRS
Sbjct  489   VV*NKGSAVPLIMIALWKSPCAGGDSVSKCTADAPAL*PARVTSSGSPPKLLALSRTQRS  548

Query  611   RVTMSLRPLFPGTLGVSNEKNPKKFNL*LKVMTTTFLVTRYSGPYRSASPDPNAKPPP*I  432
             +V MS RPL PGTL V  E+ P  FNL*L VMT T   T+ SGP  S+SP+PNAKPPP
Sbjct  549   KVIMSRRPLLPGTLVVFRERKPNTFNL*LNVMTMTPFSTKCSGPCTSSSPEPNAKPPPCT  608

Query  431   KTNIGRP-------GF------------------------VGVSPAAGRAFKGTCILA*M  345
              T IGRP       G                         V  S   GRA KGT +LA M
Sbjct  609   NTRIGRPLVK*DSSGMFLLYRTRGVSVSTSLTGRPWLVIVVRASVIEGRALKGTWMLAKM  668

Query  344   HASSMP*GFIRLP*RTSVW*QTGPSSVASR*SVHGSAGCNSLNRSSPTGCLA*GTPRKLA  165
             H S MP G IR P*+TS   QT P  VAS      S G +S N   P G  A*GTP K A
Sbjct  669   HDSLMPVGVIRGP*KTSSCGQTLPGLVASNIPCQDSNGFSSWNLRGPKGGSA*GTPLKDA  728

Query  164   YKVLASARRVRPHTQPDSVCATTLHD  87
             Y       R  P T P  V   T HD
Sbjct  729   YCRPEGVTRRAPQT*PALVRKGTAHD  754


> ta_transcript82614_1
Length=867

 Score =   229 bits (584),  Expect = 2e-64, Method: Compositional matrix adjust.
 Identities = 227/566 (40%), Positives = 267/566 (47%), Gaps = 45/566 (8%)
 Frame = -2

Query  1676  SKKFFISSCSLVRLVWWHKFGVIMSWYCSSRGVTGGHAVVSSHIGALHRIKFVTNVVIQY  1497
             SK   +SSCS+V LV +   G ++ +  +   + G H  V    GA   IK  T   I+
Sbjct  264   SKNRTMSSCSVVGLVSFVSLGEVI-FQ*ARCLLMGDHDRVE---GAPVLIKLTT*SFIRC  319

Query  1496  FI-SSSAFCDELVPDAEFTFSTYVKSSACPAP-LKCK*KACLNTL*PS*E---NWYRNTG  1332
             FI SSS F   L    E T +  V SS  PAP +  +  A L  L  S E   N Y   G
Sbjct  320   FICSSSEFLGTLT---ELTLNM*VISSTWPAPPV*LRPSARLTGL--SSELYANSYIYAG  374
```

```
Query  1331  ASPAADLFSANFMAGYL**LSVHILIKPSRSTLPLKYLWLI*FETKAIVSGVEEYSRLEG  1152
             A P   L SA F AGY   +S H     S    PLKY            SG      +
Sbjct  375   APPVFAL*SATFSAGYWKNVSEHNFKYSSMVGAPLKYFSETSLARSRATSGATLNKTVGD  434

Query  1151  TKIAGFSLILDTISICSNLCWNIWHSEFVKPIIKGMSLPRPCWTRIFGSSSNIVVIPGCG  972
                 G  LIL T+S+  NL   + HS FV PI  G+ LP+P ++ +  SS      PG G
Sbjct  435   NTNVGSFLILSTMSMSENLFLKLSHSIFVIPIRSGI*LPKPFFSNV*VSSGRTASTPGRG  494

Query  971   VSTTGKKVVRPI*SINILLASFSFSTGMATSFSCISSGLVELTPRMENKILLMDWEKIGD  792
              S TGKK   P+ S + L  +   S G++ S   I+SG V L  R    +    + KIGD
Sbjct  495   DSMTGKKNAMPLASCSALFTAMIASRGISASCWWIASGSVALMFRALR*LSAAYFAKIGD  554

Query  791   VVKKVDVPAPLINIALETKPSVALDKVRR*aaaapalwpAKVTLSGLPPKALAFLFTQRS  612
             VV       PLI IAL   P    D V +  A APAL PA+VT SG PPK LA   TQRS
Sbjct  555   VV*NKGSAVPLIMIALWKSPCAGGDSVSKCTADAPAL*PARVTSSGSPPKLLALSRTQRS  614

Query  611   RVTMSLRPLFPGTLGVSNEKNPKKFNL*LKVMTTTFLVTRYSGPYRSASPDPNAKPPP*I  432
             +V MS RPL PGTL V  E+ P  FNL*L VMT T   T+ SGP  S+SP+PNAKPPP
Sbjct  615   KVIMSRRPLLPGTLVVFRERKPNTFNL*LNVMTMTPFSTKCSGPCTSSSPEPNAKPPPCT  674

Query  431   KTNIGRP-------GF------------------------VGVSPAAGRAFKGTCILA*M  345
              T IGRP       G                         V  S   GRA KGT +LA M
Sbjct  675   NTRIGRPLVK*DSSGMFLLYRTRGVSVSTSLTGRPWLVIVVRASVIEGRALKGTWMLAKM  734

Query  344   HASSMP*GFIRLP*RTSVW*QTGPSSVASR*SVHGSAGCNSLNRSSPTGCLA*GTPRKLA  165
             H S MP G IR P*+TS   QT P  VAS      S G +S N   P G  A*GTP K A
Sbjct  735   HDSLMPVGVIRGP*KTSSCGQTLPGLVASNIPCQDSNGFSSWNLRGPKGGSA*GTPLKDA  794

Query  164   YKVLASARRVRPHTQPDSVCATTLHD  87
             Y       R  P T P  V   T HD
Sbjct  795   YCRPEGVTRRAPQT*PALVRKGTAHD  820


> ta_transcript82616_1
Length=859

 Score =   224 bits (571),  Expect = 1e-62, Method: Compositional matrix adjust.
 Identities = 225/566 (40%), Positives = 264/566 (47%), Gaps = 45/566 (8%)
 Frame = -2

Query  1676  SKKFFISSCSLVRLVWWHKFGVIMSWYCSSRGVTGGHAVVSSHIGALHRIKFVTNVVIQY  1497
             SK   +SSCS+V  V       ++ + +   + G H  V    GA   IK  T   I+
Sbjct  256   SKNRTMSSCSVVGFVSLVSLEEVI-FQ*ARCLLMGDHDRVE---GAPVLIKLTT*SFIRC  311

Query  1496  FI-SSSAFCDELVPDAEFTFSTYVKSSACPAP-LKCK*KACLNTL*PS*E---NWYRNTG  1332
             FI SSS F   L    E T +  V SS  PAP +  +  A L  L  S E   N Y   G
Sbjct  312   FICSSSEFLGTLT---ELTLNM*VISSTWPAPPV*LRPSARLTGL--SSELYANSYIYAG  366

Query  1331  ASPAADLFSANFMAGYL**LSVHILIKPSRSTLPLKYLWLI*FETKAIVSGVEEYSRLEG  1152
             A P   L SA F AGY   +S H     S    PLKY            SG      +
Sbjct  367   APPVFAL*SATFSAGYWKNVSEHNFKYSSMVGAPLKYFSETSLARSRATSGATLNKTVGD  426

Query  1151  TKIAGFSLILDTISICSNLCWNIWHSEFVKPIIKGMSLPRPCWTRIFGSSSNIVVIPGCG  972
                 G  LIL T+S+  NL   + HS FV PI  G+ LP+P ++ +  SS      PG G
Sbjct  427   NTNVGSFLILSTMSMSENLFLKLSHSIFVIPIRSGI*LPKPFFSNV*VSSGRTASTPGRG  486

Query  971   VSTTGKKVVRPI*SINILLASFSFSTGMATSFSCISSGLVELTPRMENKILLMDWEKIGD  792
              S TGKK   P+ S + L  +   S G++ S   I+SG V L  R    +    + KIGD
Sbjct  487   DSMTGKKNAMPLASCSALFTAMIASRGISASCWWIASGSVALMFRALR*LSAAYFAKIGD  546

Query  791   VVKKVDVPAPLINIALETKPSVALDKVRR*aaaapalwpAKVTLSGLPPKALAFLFTQRS  612
             VV       PLI IAL   P    D V +  A APAL PA+VT SG PPK LA   TQRS
Sbjct  547   VV*NKGSAVPLIMIALWKSPCAGGDSVSKCTADAPAL*PARVTSSGSPPKLLALSRTQRS  606

Query  611   RVTMSLRPLFPGTLGVSNEKNPKKFNL*LKVMTTTFLVTRYSGPYRSASPDPNAKPPP*I  432
             +V MS RPL PGTL V  E+ P  FNL*L VMT T   T+ SGP  S+SP+PNAKPPP
Sbjct  607   KVIMSRRPLLPGTLVVFRERKPNTFNL*LNVMTMTPFSTKCSGPCTSSSPEPNAKPPPCT  666

Query  431   KTNIGRP-------GF------------------------VGVSPAAGRAFKGTCILA*M  345
              T IGRP       G                         V  S   GRA KGT +LA M
Sbjct  667   NTRIGRPLVK*DSSGMFLLYRTRGVSVSTSLTGRPWLVIVVRASVIEGRALKGTWMLAKM  726

Query  344   HASSMP*GFIRLP*RTSVW*QTGPSSVASR*SVHGSAGCNSLNRSSPTGCLA*GTPRKLA  165
             H S MP G IR P*+TS   QT P  VAS      S G +S N   P G  A*GTP K A
Sbjct  727   HDSLMPVGVIRGP*KTSSCGQTLPGLVASNIPCQDSNGFSSWNLRGPKGGSA*GTPLKDA  786
```

```
Query  164   YKVLASARRVRPHTQPDSVCATTLHD  87
             Y       R  P T P  V   T HD
Sbjct  787   YCRPEGVTRRAPQT*PALVRKGTAHD  812


> ta_transcript82623_1
Length=454

 Score =  202 bits (513),  Expect = 6e-57, Method: Compositional matrix adjust.
 Identities = 168/388 (43%), Positives = 196/388 (51%), Gaps = 32/388 (8%)
 Frame = -2

Query  1157  EGTKIAGFSLILDTISICSNLCWNIWHSEFVKPIIKGMSLPRPCWTRIFGSSSNIVVIPG  978
             + T +  F LIL T+S+  NL   + HS FV PI  G+ LP+P ++ +  SS      PG
Sbjct  21    DNTNVGSF-LILSTMSMSENLFLKLSHSIFVIPIRSGI*LPKPFFSNV*VSSGRTASTPG  79

Query  977   CGVSTTGKKVVRPI*SINILLASFSFSTGMATSFSCISSGLVELTPRMENKILLMDWEKI  798
              G S TGKK   P+ S + L  +   S G++ S   I+SG V L  R    +    + KI
Sbjct  80    RGDSMTGKKNAMPLASCSALFTAMIASRGISASCWWIASGSVALMFRALR*LSAAYFAKI  139

Query  797   GDVVKKVDVPAPLINIALETKPSVALDKVRR*aaaapalwpAKVTLSGLPPKALAFLFTQ  618
             GDVV       PLI IAL   P    D V +  A APAL PA+VT SG PPK LA   TQ
Sbjct  140   GDVV*NKGSAVPLIMIALWKSPCAGGDSVSKCTADAPAL*PARVTSSGSPPKLLALSRTQ  199

Query  617   RSRVTMSLRPLFPGTLGVSNEKNPKKFNL*LKVMTTTFLVTRYSGPYRSASPDPNAKPPP  438
             RS+V MS RPL PGTL V  E+ P  FNL*L VMT T   T+ SGP  S+SP+PNAKPPP
Sbjct  200   RSKVIMSRRPLLPGTLVVFRERKPNTFNL*LNVMTMTPFSTKCSGPCTSSSPEPNAKPPP  259

Query  437   *IKTNIGRP-------GF------------------------VGVSPAAGRAFKGTCILA  351
                T IGRP       G                         V  S   GRA KGT +LA
Sbjct  260   CTNTRIGRPLVK*DSSGMFLLYRTRGVSVSTSLTGRPWLVIVVRASVIEGRALKGTWMLA  319

Query  350   *MHASSMP*GFIRLP*RTSVW*QTGPSSVASR*SVHGSAGCNSLNRSSPTGCLA*GTPRK  171
              MH S MP G IR P*+TS   QT P  VAS      S G +S N   P G  A*GTP K
Sbjct  320   KMHDSLMPVGVIRGP*KTSSCGQTLPGLVASNIPCQDSNGFSSWNLRGPKGGSA*GTPLK  379

Query  170   LAYKVLASARRVRPHTQPDSVCATTLHD  87
              AY       R  P T P  V   T HD
Sbjct  380   DAYCRPEGVTRRAPQT*PALVRKGTAHD  407


> ta_transcript58915_1
Length=606

 Score =  205 bits (521),  Expect = 7e-57, Method: Compositional matrix adjust.
 Identities = 170/524 (32%), Positives = 259/524 (49%), Gaps = 44/524 (8%)
 Frame = +1

Query  106   QTESGWVCG-RTRRAEASTLYASFRGVPYAKQPVGELRFKELQPAEPWTDYLDATEEGPV  282
             Q E GW+ G +         + SF+G+PYA  PVG+LRFK  QP   W     AT+ GP
Sbjct  33    QVEQGWLEGEQLDSVSGDGQFFSFKGIPYAAPPVGKLRFKAPQPPVSWQGVRKATQHGPK  92

Query  283   CYQTDVLYGSLMKPHGMDEACIYANIHVPLNALPAAGETPTKPGLPILVFIHgggfafgs  462
             C Q D+    ++      E C+Y N++            +P  P LP++VFIHGGGF  GS
Sbjct  93    CPQRDIFSKQIIPG---SEDCLYLNVY-------TKDISPKSP-LPVMVFIHGGGFKSGS  141

Query  463   gdadLYGPEYLVTRNVVVITFNYRLNFFGFFSLDTPKVPGNNGLRDMVTLLRWVKRNARA  642
             GD D YG ++LV   VV++T NYRL+  GF  LDT +VPGN GL+D V  L+WV++N  A
Sbjct  142   GDEDFYGGDFLVHHGVVLVTINYRLDALGFLCLDTEEVPGNAGLKDQVAALKWVQKNIFA  201

Query  643   FGGNPDNVtlagqsagaaaahlltlsKATEGLVSRAILMSGAGtstffttspifsqsINK  822
             FGG+P NVT+ G+SAG ++  L  LS  ++GL  RAI MSG     +        ++
Sbjct  202   FGGDPTNVTIFGESAGGSSTALHVLSPLSKGLFKRAIPMSGVPFCDWSIPFEPRKRAFT-  260

Query  823   ILFSILGVNSTNPDEIHEKLVAMPVEKLNEANRILI-------DQIGLTTFFPVVETPHP  981
              L  ILG  + +P  + E L ++P EK  + N  ++       + + +  F PVVE  +
Sbjct  261   -LGKILGFETEDPKALLEYLQSLPAEKFVDTNPTIMGFEEKSYNMLKMYHFTPVVEK-NF  318

Query  982   GITTILDEDPNILVQQGRGKDIPLIIGFTNSECHMFQHRFEQIDIVSKINENPAILVPSN  1161
             G    + E+P   ++QG   D+ ++IG T+ E  +    F  +D++   +  P + VP
Sbjct  319   GQHHFMTEEPLEALKQGHVNDVDVLIGNTDQETIVGIPSF--VDLLKMYDRYPEVFVPRK  376

Query  1162  LLYSSTPETIALVSNQISQRYF-NGSVD-------LEGFINMC-TDSYYKYPAMKLAEKR  1314
             +L  STP  I  ++ +I + YF +  +D       +  F  +C T    KY  + L+ K
Sbjct  377   ILNKSTPGKILEIAERIRKHYFGDKPIDKTSMKEAVTYFSEVCFTYDVNKYTNLLLSGK-  435

Query  1315  SAAGDAPVFLYQFS----YDGYSVFKQAFHLHFNGAGHADDLTYVLKVNSASGTSSSQKA  1482
```

```
                G + V+ Y+FS     + +  + Q + +   GA H +DL Y+    S
Sbjct  436   --PGSSKVYRYRFSCVSERNIFGKYGQEYGI--TGASHLEDLMYLFDAKSVKLPLDRNSK  491

Query  1483  DDEMKYWMTTFVTNFMRCSAPMCDETTA--WPPVTPRELQYQDI  1608
               +M        TNF +   P  D +    WP    ++  + DI
Sbjct  492   SYKMIQQTCALFTNFAKYGNPTPDSSLGFTWPEYDIKDQSFVDI  535


> ta_transcript58914_1
Length=650

 Score =   205 bits (521),  Expect = 1e-56, Method: Compositional matrix adjust.
 Identities = 170/525 (32%), Positives = 259/525 (49%), Gaps = 44/525 (8%)
 Frame = +1

Query  103   AQTESGWVCG-RTRRAEASTLYASFRGVPYAKQPVGELRFKELQPAEPWTDYLDATEEGP  279
              Q E GW+ G +         + SF+G+PYA  PVG+LRFK  QP   W     AT+ GP
Sbjct  32    VQVEQGWLEGEQLDSVSGDGQFFSFKGIPYAAPPVGKLRFKAPQPPVSWQGVRKATQHGP  91

Query  280   VCYQTDVLYGSLMKPHGMDEACIYANIHVPLNALPAAGETPTKPGLPILVFIHgggfafg  459
              C Q D+    ++      E C+Y N++           +P  P LP++VFIHGGGF  G
Sbjct  92    KCPQRDIFSKQIIPG---SEDCLYLNVY-------TKDISPKSP-LPVMVFIHGGGFKSG  140

Query  460   sgdadLYGPEYLVTRNVVVITFNYRLNFFGFFSLDTPKVPGNNGLRDMVTLLRWVKRNAR  639
             SGD D YG ++LV   VV++T NYRL+  GF  LDT +VPGN GL+D V  L+WV++N
Sbjct  141   SGDEDFYGGDFLVHHGVVLVTINYRLDALGFLCLDTEEVPGNAGLKDQVAALKWVQKNIF  200

Query  640   AFGGNPDNVtlagqsagaaaahlltlsKATEGLVSRAILMSGAGtstfttspifsqsIN  819
             AFGG+P NVT+ G+SAG ++  L  LS  ++GL  RAI MSG     +        ++
Sbjct  201   AFGGDPTNVTIFGESAGGSSTALHVLSPLSKGLFKRAIPMSGVPFCDWSIPFEPRKRAFT  260

Query  820   KILFSILGVNSTNPDEIHEKLVAMPVEKLNEANRILI-------DQIGLTTFFPVVETPH  978
               L  ILG  + +P  + E L ++P EK  + N  ++       + + +  F PVVE  +
Sbjct  261   --LGKILGFETEDPKALLEYLQSLPAEKFVDTNPTIMGFEEKSYNMLKMYHFTPVVEK-N  317

Query  979   PGITTILDEDPNILVQQGRGKDIPLIIGFTNSECHMFQHRFEQIDIVSKINENPAILVPS  1158
              G    + E+P   ++QG   D+ ++IG T+ E  +    F  +D++   +  P + VP
Sbjct  318   FGQHHFMTEEPLEALKQGHVNDVDVLIGNTDQETIVGIPSF--VDLLKMYDRYPEVFVPR  375

Query  1159  NLLYSSTPETIALVSNQISQRYF-NGSVD-------LEGFINMC-TDSYYKYPAMKLAEK  1311
              +L  STP  I  ++ +I + YF +  +D       +  F  +C T    KY  + L+ K
Sbjct  376   KILNKSTPGKILEIAERIRKHYFGDKPIDKTSMKEAVTYFSEVCFTYDVNKYTNLLLSGK  435

Query  1312  RSAAGDAPVFLYQFS----YDGYSVFKQAFHLHFNGAGHADDLTYVLKVNSASGTSSSQK  1479
                 G + V+ Y+FS     + +  + Q + +   GA H +DL Y+    S
Sbjct  436   ---PGSSKVYRYRFSCVSERNIFGKYGQEYGI--TGASHLEDLMYLFDAKSVKLPLDRNS  490

Query  1480  ADDEMKYWMTTFVTNFMRCSAPMCDETTA--WPPVTPRELQYQDI  1608
                +M        TNF +   P  D +    WP    ++  + DI
Sbjct  491   KSYKMIQQTCALFTNFAKYGNPTPDSSLGFTWPEYDIKDQSFVDI  535


> ta_transcript75783_1
Length=733

 Score =   189 bits (479),  Expect = 8e-51, Method: Compositional matrix adjust.
 Identities = 162/525 (31%), Positives = 260/525 (50%), Gaps = 46/525 (9%)
 Frame = +1

Query  103   AQTESGWVCGRTRRAEASTLYASFRGVPYAKQPVGELRFKELQPAEPWTDYLDATEEGPV  282
              +   G + G   + E    Y SF+G+P+A+ PVG+LRFK   PA+PW     AT+ GP+
Sbjct  126   VKVSEGILQGELVQNEFGGSYYSFKGIPFAEPPVGDLRFKAPIPAKPWKGVKTATKFGPI  185

Query  283   CYQTDVLYGSLMKPHGMDEACIYANIHVPLNALPAAGETPTKPGLPILVFIHgggfafgs  462
              YQ DV       P    E C+Y N++ P        E      LP++V+IHGGG+ +GS
Sbjct  186   NYQFDVFKKD---PPSGSEDCLYLNVYTP--------EIKPITLLPVMVWIHGGGYFWGS  234

Query  463   gdadLYGPEYLVTRNVVVITFNYRLNFFGFFSLDTPKVPGNNGLRDMVTLLRWVKRNARA  642
             G+ D+YGPE+LV   V+++TFNYRL   GF SLDT +VPGN G++D V  L+WVK N
Sbjct  235   GNDDVYGPEFLVRHGVILVTFNYRLGVLGFLSLDTAEVPGNAGMKDQVAALKWVKSNIHF  294

Query  643   FGGNPDNVtlagqsagaaaahlltlsKATEGLVSRAILMSGAGtstfttspifsqsINK  822
             FGG+P+N+T+ G+SAGA A  L  LS  ++GL  RAI+ SG+ T+ +     I  +SI
Sbjct  295   FGGDPENITIFGESAGAGAVSLHLLSPMSKGLFKRAIIQSGSATAYWAQAFEIKEKSI--  352

Query  823   ILFSILGVNSTNPDEIHEKL----------VAMPVEKLNEANRILIDQIGLTTFFPVVET  972
              L   LG+ + +  +++E            +++PV    E   + I+      F P +E
```

```
Sbjct  353   ALARKLGLVTDDDQKLYEFFGNISKELLINISVPVT-FAEQEEVNIE------FAPTIEK  405

Query  973   PHPGITTILDEDPNILVQQGRGKDIPLIIGFTNSECHMFQHRFEQIDI-VSKINENPAIL  1149
                G    L +D   ++QQG  K + +++G+   E  ++ +  E +D  + +IN     L
Sbjct  406   DF-GQERFLTDDSYKILQQGIHKGVDVMMGYVEDEGVLYLNSLEILDKEIERINNFLEAL  464

Query  1150  VPSNLLYSSTPETIALVSNQISQRYFNGSV----DLEGFINMCTDSYYKYPAMKLAEKRS  1317
             VP N+      +    V  ++ + YF+  +    D        + + + Y  ++   +++
Sbjct  465   VPRNMKREIPIKQQLKVGRKMKKFYFDNKLVTRNDWRTIARYISLNVFSYDVIQ--SQKN  522

Query  1318  AAGDAPVFLYQFS-YDGYSVFKQAF-----HLHFNGAGHADDLTYVLKVNSASGTSSSQK  1479
              A     F Y+F+     + FK+ F         +   H DDL Y+  V   + + +
Sbjct  523   IAKSQNSFFYKFTCKSKRNFFKKVFTDDEITKDVDVVCHCDDLAYLFPVKFMNQSVAKDS  582

Query  1480  ADDEMKYWMTTFVTNFMRCSAPMCDET--TAWPPVTPRELQYQDI  1608
              + ++   +T   TNF +   P  D +    W P T +   Y DI
Sbjct  583   REFQLIDKVTKLWTNFAKYGNPTPDNSLGVKWEPFTLQHQHYLDI  627


> ta_transcript48580_1
Length=834

 Score =   187 bits (476),  Expect = 3e-50, Method: Compositional matrix adjust.
 Identities = 172/525 (33%), Positives = 262/525 (50%), Gaps = 35/525 (7%)
 Frame = +1

Query  100   VAQTESGWVCGR--TRRAEASTLYASFRGVPYAKQPVGELRFKELQPAEPWTDYLDATEE  273
             +  TE G + GR    R  A T Y SF+G+ YA+ P G LRFK   P EPW+   DA EE
Sbjct  155   IVMTELGALRGRRVVARTTAQTPYFSFKGIRYAQPPRGSLRFKPPVPLEPWSGVRDALEE  214

Query  274   GPVCYQTDVLYGSLMKPHGMDEACIYANIHVPLNALPAAGETPTKPGLPILVFIHgggfa  453
             G VC    +L+ +    +  DE C++ N++ P  ALP    T   P L ++V+IHGG FA
Sbjct  215   GAVCPHRFMLFDT----YKGDEDCLFLNVYTP--ALPEK-ITGFNPKLAVMVWIHGGAFA  267

Query  454   fgsgdadLYGPEYLVTRNVVVITFNYRLNFFGFFSLDTPKVPGNNGLRDMVTLLRWVKRN  633
              GSG+A LYGP++L+  N+V++T NYRL   GF S++  +V GN GL+D V  L+WV+ N
Sbjct  268   VGSGNAFLYGPDHLMGGNIVLVTLNYRLGALGFLSMENDEVSGNMGLKDQVLALKWVRDN  327

Query  634   ARAFGGNPDNVtlagqsagaaaahlltlsKATEGLVSRAILMSGAGtstffttspifsqs  813
                FGG+   VT+ G+SAGAA+ HL  LS A++GL  RAI  SG   S +  T     ++
Sbjct  328   IEYFGGDASRVTIFGESAGAASVHLHMLSPASKGLFHRAIAQSGLALSPWALTRSPRERA  387

Query  814   INKILFSILGVNSTNPDEIHEKLVAMPVEKLNEANRILIDQIGL------TTFFPVVETP  975
                 L   LG+++ +  E+   L A P E L +A   L    G       T   P V T
Sbjct  388   FE--LGRELGIDTNSTAELLGYLRATPSELLVKAGARLAGAPGKSVDLHSTVALPFVPTV  445

Query  976   HPGI-TTILDEDPNILVQQGRGKDIPLIIGFTNSECHMFQHRFEQI-DIVSKINENPAIL  1149
              P +    L + P  L+    G D+PL+ G+   E  +   R ++   ++++++     +
Sbjct  446   EPDVPDAFLTQHPRNLMP---GADVPLLTGYNAQEGIILFRRLQRYPKLLTELDREFRRV  502

Query  1150  VPSNLLYSSTPETIALVSNQISQRYFNGS-VD---LEGFINMCTDSYYKYPAMK-LAEKR  1314
             VP  LL     +    V++ I   YF    VD   ++  I++ TD  +  P ++ L  +
Sbjct  503   VPPELLTGDEAQN-RRVADHIRAFYFQQRPVDIRNIDSLIDLFTDVMFLRPLLETLRIQG  561

Query  1315  SAAGDAPVFLYQFSYDG-YSVFKQAFHLHFNGAGHADDLTYVLKVNSASGTSSSQKADDE  1491
                  +P +LY+F++DG   +FK+   ++  GA H D++ Y+   +  +        +
Sbjct  562   KTNRTSPTYLYRFAFDGALGLFKRMLGINHPGACHGDEMGYLFYFSRLNYRLDDDSTELA  621

Query  1492  MKYWMTTFVTNFMRCSAPMCDETT-----AWPPVT-PRELQYQDI  1608
             +   M    TNF +   P   +        W PV     L Y DI
Sbjct  622   VSKGMVRMWTNFAKTGNPTIGDHDDPIDFKWSPVNDTTHLTYLDI  666


> ta_transcript48579_1
Length=905

 Score =   188 bits (477),  Expect = 4e-50, Method: Compositional matrix adjust.
 Identities = 172/525 (33%), Positives = 262/525 (50%), Gaps = 35/525 (7%)
 Frame = +1

Query  100   VAQTESGWVCGR--TRRAEASTLYASFRGVPYAKQPVGELRFKELQPAEPWTDYLDATEE  273
             +  TE G + GR    R  A T Y SF+G+ YA+ P G LRFK   P EPW+   DA EE
Sbjct  155   IVMTELGALRGRRVVARTTAQTPYFSFKGIRYAQPPRGSLRFKPPVPLEPWSGVRDALEE  214

Query  274   GPVCYQTDVLYGSLMKPHGMDEACIYANIHVPLNALPAAGETPTKPGLPILVFIHgggfa  453
             G VC    +L+ +    +  DE C++ N++ P  ALP    T   P L ++V+IHGG FA
Sbjct  215   GAVCPHRFMLFDT----YKGDEDCLFLNVYTP--ALPEK-ITGFNPKLAVMVWIHGGAFA  267
```

```
Query  454   fgsgdadLYGPEYLVTRNVVVITFNYRLNFFGFFSLDTPKVPGNNGLRDMVTLLRWVKRN  633
              GSG+A LYGP++L+  N+V++T NYRL   GF S++  +V GN GL+D V  L+WV+ N
Sbjct  268   VGSGNAFLYGPDHLMGGNIVLVTLNYRLGALGFLSMENDEVSGNMGLKDQVLALKWVRDN  327

Query  634   ARAFGGNPDNVtlagqsagaaaahlltlsKATEGLVSRAILMSGAGtstffttspifsqs  813
                 FGG+   VT+ G+SAGAA+ HL  LS A++GL  RAI  SG   S +  T     ++
Sbjct  328   IEYFGGDASRVTIFGESAGAASVHLHMLSPASKGLFHRAIAQSGLALSPWALTRSPRERA  387

Query  814   INKILFSILGVNSTNPDEIHEKLVAMPVEKLNEANRILIDQIGL------TTFFPVVETP  975
                  L   LG+++ +  E+   L A P E L +A   L    G       T   P V T
Sbjct  388   FE--LGRELGIDTNSTAELLGYLRATPSELLVKAGARLAGAPGKSVDLHSTVALPFVPTV  445

Query  976   HPGI-TTILDEDPNILVQQGRGKDIPLIIGFTNSECHMFQHRFEQI-DIVSKINENPAIL  1149
               P +    L + P  L+    G D+PL+ G+   E  +   R ++   ++++++     +
Sbjct  446   EPDVPDAFLTQHPRNLMP---GADVPLLTGYNAQEGIILFRRLQRYPKLLTELDREFRRV  502

Query  1150  VPSNLLYSSTPETIALVSNQISQRYFNGS-VD---LEGFINMCTDSYYKYPAMK-LAEKR  1314
             VP  LL     +    V++ I   YF    VD   ++  I++ TD  +  P ++ L  +
Sbjct  503   VPPELLTGDEAQN-RRVADHIRAFYFQQRPVDIRNIDSLIDLFTDVMFLRPLLETLRIQG  561

Query  1315  SAAGDAPVFLYQFSYDG-YSVFKQAFHLHFNGAGHADDLTYVLKVNSASGTSSSQKADDE  1491
                  +P +LY+F++DG   +FK+   ++  GA H D++ Y+   +  +        +
Sbjct  562   KTNRTSPTYLYRFAFDGALGLFKRMLGINHPGACHGDEMGYLFYFSRLNYRLDDDSTELA  621

Query  1492  MKYWMTTFVTNFMRCSAPMCDETT-----AWPPVT-PRELQYQDI  1608
             +   M    TNF +   P   +        W PV     L Y DI
Sbjct  622   VSKGMVRMWTNFAKTGNPTIGDHDDPIDFKWSPVNDTTHLTYLDI  666


> ta_transcript48393_1
Length=666

 Score =   147 bits (372),  Expect = 4e-37, Method: Compositional matrix adjust.
 Identities = 143/455 (31%), Positives = 221/455 (49%), Gaps = 34/455 (7%)
 Frame = +1

Query  100   VAQTESGWVCGRTRRAEASTLYASFRGVPYAKQPVGELRFKELQPAEPWTDYLDATEEGP  279
             + +T  G V  R  R     L+A F  +PYA  P G L+F        WT+  +A ++
Sbjct  95    IVETTQGPV--RGYRDPEGGLFA-FYSIPYATAPSGPLKFTAPITPPTWTEPFEAVDKRI  151

Query  280   VCYQTDVLYGSLMKPHGMDEACIYANIHVPLNALPAAGETPTKPGLPILVFIHgggfafg  459
             +C Q   +     K   M   C+ AN++VP            +  LP++V++HGG F  G
Sbjct  152   ICPQ---MKSGFTKQMTMQVDCLIANVYVP---------DTKETKLPVIVYVHGGAFQVG  199

Query  460   sgdadLYGPEYLV-TRNVVVITFNYRLNFFGFFSLDTPKVPGNNGLRDMVTLLRWVKRNA  636
              G+  L  P+ LV ++N++ +TFNYRL   GF  L T   PGN GL+D V  LRWV +N
Sbjct  200   FGN--LMTPKNLVRSKNIIAVTFNYRLGAHGFLCLGTEDAPGNAGLKDQVAALRWVNKNI  257

Query  637   RAFGGNPDNVtlagqsagaaaahlltlsKATEGLVSRAILMSGAGtstffttspifsqsI  816
                GGNPD+VT+AG SAGA +  L+ +SK+ EGL S+ I+ SG+        +     +
Sbjct  258   IKLGGNPDDVTIAGYSAGAISVDLMAISKSAEGLFSKIIIESGSSLFAGSIVTDPLENA-  316

Query  817   NKILFSILGVNSTNPDEIHEKLV-AMPVEKLNEANRILIDQIGLTTFF-PVVETPHPGIT  990
              KI  + LG N+ +     EK   A   + L +     +D+   T  F P VE    G+
Sbjct  317   -KIFAARLGFNNIDDVYALEKFYKAASYDSLTKGE--FMDRPNSTLLFSPCVERDDDGL-  372

Query  991   TILDEDPNILVQQGRGKDIPLIIGFTNSECHMFQHRFEQIDIVSKINENPAILVPSNLLY  1170
               L E P  ++++G    +P+I GF+N E    +  FE   +  K+NE  +  +P +L +
Sbjct  373   AFLSEPPINILKEGSYTQVPMIYGFSNMEGLFREPFFEFWKL--KMNEQFSEFIPGDLHF  430

Query  1171  SSTPETIALVSNQISQRYF-----NGSVDLEGFINMCTDSYYKYPAMKLAEKRSAAGDAP  1335
              S  +    ++ +I + YF     NG   L+ +I+   D   KYPA++  +     G
Sbjct  431   ESKHDR-ERIAKRIKKFYFWDSPINGENILK-YIDYFGDVLVKYPALRTVKMHIQNGHDK  488

Query  1336  VFLYQFSYDGYSVFKQAFHLHFNGAGHADDLTYVL  1440
             ++LY++SY   +V       +  GA H      VL
Sbjct  489   LYLYEYSYVDENVPAVIHTTNTRGANHCAQTVAVL  523


> ta_transcript84821_1
Length=611

 Score =   146 bits (368),  Expect = 7e-37, Method: Compositional matrix adjust.
 Identities = 121/362 (33%), Positives = 193/362 (53%), Gaps = 31/362 (9%)
 Frame = +1
```

```
Query  169   SFRGVPYAKQPVGELRFKELQPAEPWTDYLDATEEGPVCYQTDVLYGSLMKPHGMDEACI  348
             +F  +PYA  P G+ RFK   PA  W    +A + G VC+Q D+    +       E C+
Sbjct  59   TFYNIPYATAPTGKDRFKAPLPAPVWITTREAVDRGVVCHQIDLAAVGIFNKT-KQEDCL  117

Query  349  YANIHVPLNALPAAGETPTKPGLPILVFIHgggfafgsgdadLYGPEYLV-TRNVVVITF  525
             AN++VP            K  LP++VF+HGG +  G G+  +  P+++V T NV+ +TF
Sbjct  118  IANVYVP---------DTDKKNLPVVVFVHGGAYLAGFGE--VLTPKHMVRTNNVIYVTF  166

Query  526  NYRLNFFGFFSLDTPKVPGNNGLRDMVTLLRWVKRNARAFGGNPDNVtlagqsagaaaah  705
             NYRL   GF  L TP  PGN G++DMV LLRWVK+N  +FGGNPD+VT+ G SAG++A
Sbjct  167  NYRLGVQGFLCLGTPDAPGNAGMKDMVALLRWVKKNIASFGGNPDDVTIDGYSAGSSAVD  226

Query  706  lltlsKATEGLVSRAILMSGAGtstffttspifsqsINKILFSILGVNSTNPDEIHEKLV  885
             LL LSKAT+GL  +AI  SGA           ++S  ++ I  +     S +  EI + +
Sbjct  227  LLLLSKATKGLFHKAIPESGAN-------VAMWSVQVDPIANAKEYAKSEHGFEILDDIY  279

Query  886  A-------MPVEKLNEAN-RILIDQIGLTTFFPVVETPHPGITTILDEDPNILVQQGRGK  1041
             A       +P +++ ++   ++        F P VE    G+   LD+ P  ++++G
Sbjct  280  ALEDFYKSLPYDEMYKSGMELMFKNKNNFLFSPCVER-DTGVEKFLDDTPVNILKKGDYN  338

Query  1042 DIPLIIGFTNSECHMFQHRFEQIDIVSKINENPAILVPSNLLYSSTPETIALVSNQISQR  1221
              +P++ GF N E  +F+        +S++N+  +  +P +L +    E    V++++ +
Sbjct  339  KVPVLYGFANME-GLFRVGMGYEYFMSQLNDKFSENLPHDLEFKDEEEKRK-VADEVKKF  396

Query  1222 YF  1227
             YF
Sbjct  397  YF  398


> ta_transcript84818_1
Length=733

 Score =   146 bits (368),  Expect = 2e-36, Method: Compositional matrix adjust.
 Identities = 121/362 (33%), Positives = 193/362 (53%), Gaps = 31/362 (9%)
 Frame = +1

Query  169  SFRGVPYAKQPVGELRFKELQPAEPWTDYLDATEEGPVCYQTDVLYGSLMKPHGMDEACI  348
             +F  +PYA  P G+ RFK   PA  W    +A + G VC+Q D+    +       E C+
Sbjct  59   TFYNIPYATAPTGKDRFKAPLPAPVWITTREAVDRGVVCHQIDLAAVGIFNKT-KQEDCL  117

Query  349  YANIHVPLNALPAAGETPTKPGLPILVFIHgggfafgsgdadLYGPEYLV-TRNVVVITF  525
             AN++VP            K  LP++VF+HGG +  G G+  +  P+++V T NV+ +TF
Sbjct  118  IANVYVP---------DTDKKNLPVVVFVHGGAYLAGFGE--VLTPKHMVRTNNVIYVTF  166

Query  526  NYRLNFFGFFSLDTPKVPGNNGLRDMVTLLRWVKRNARAFGGNPDNVtlagqsagaaaah  705
             NYRL   GF  L TP  PGN G++DMV LLRWVK+N  +FGGNPD+VT+ G SAG++A
Sbjct  167  NYRLGVQGFLCLGTPDAPGNAGMKDMVALLRWVKKNIASFGGNPDDVTIDGYSAGSSAVD  226

Query  706  lltlsKATEGLVSRAILMSGAGtstffttspifsqsINKILFSILGVNSTNPDEIHEKLV  885
             LL LSKAT+GL  +AI  SGA           ++S  ++ I  +     S +  EI + +
Sbjct  227  LLLLSKATKGLFHKAIPESGAN-------VAMWSVQVDPIANAKEYAKSEHGFEILDDIY  279

Query  886  A-------MPVEKLNEAN-RILIDQIGLTTFFPVVETPHPGITTILDEDPNILVQQGRGK  1041
             A       +P +++ ++   ++        F P VE    G+   LD+ P  ++++G
Sbjct  280  ALEDFYKSLPYDEMYKSGMELMFKNKNNFLFSPCVER-DTGVEKFLDDTPVNILKKGDYN  338

Query  1042 DIPLIIGFTNSECHMFQHRFEQIDIVSKINENPAILVPSNLLYSSTPETIALVSNQISQR  1221
              +P++ GF N E  +F+        +S++N+  +  +P +L +    E    V++++ +
Sbjct  339  KVPVLYGFANME-GLFRVGMGYEYFMSQLNDKFSENLPHDLEFKDEEEKRK-VADEVKKF  396

Query  1222 YF  1227
             YF
Sbjct  397  YF  398


> ta_transcript84816_1
Length=947

 Score =   145 bits (365),  Expect = 1e-35, Method: Compositional matrix adjust.
 Identities = 121/362 (33%), Positives = 193/362 (53%), Gaps = 31/362 (9%)
 Frame = +1

Query  169  SFRGVPYAKQPVGELRFKELQPAEPWTDYLDATEEGPVCYQTDVLYGSLMKPHGMDEACI  348
             +F  +PYA  P G+ RFK   PA  W    +A + G VC+Q D+    +       E C+
Sbjct  59   TFYNIPYATAPTGKDRFKAPLPAPVWITTREAVDRGVVCHQIDLAAVGIFNK-TKQEDCL  117

Query  349  YANIHVPLNALPAAGETPTKPGLPILVFIHgggfafgsgdadLYGPEYLV-TRNVVVITF  525
```

```
                AN++VP             K  LP++VF+HGG +  G G+   +   P+++V T NV+ +TF
Sbjct  118   IANVYVP---------DTDKKNLPVVVFVHGGAYLAGFGE--VLTPKHMVRTNNVIYVTF  166

Query  526   NYRLNFFGFFSLDTPKVPGNNGLRDMVTLLRWVKRNARAFGGNPDNVtlagqsagaaaah  705
             NYRL   GF  L TP  PGN G++DMV LLRWVK+N  +FGGNPD+VT+ G SAG++A
Sbjct  167   NYRLGVQGFLCLGTPDAPGNAGMKDMVALLRWVKKNIASFGGNPDDVTIDGYSAGSSAVD  226

Query  706   lltlsKATEGLVSRAILMSGAGtstffttspifsqsINKILFSILGVNSTNPDEIHEKLV  885
             LL LSKAT+GL  +AI  SGA           ++S  ++ I  +     S +  EI + +
Sbjct  227   LLLLSKATKGLFHKAIPESGAN-------VAMWSVQVDPIANAKEYAKSEHGFEILDDIY  279

Query  886   A-------MPVEKLNEAN-RILIDQIGLTTFFPVVETPHPGITTILDEDPNILVQQGRGK  1041
             A       +P +++ ++   ++         F P VE    G+    LD+ P  ++++G
Sbjct  280   ALEDFYKSLPYDEMYKSGMELMFKNKNNFLFSPCVER-DTGVEKFLDDTPVNILKKGDYN  338

Query  1042  DIPLIIGFTNSECHMFQHRFEQIDIVSKINENPAILVPSNLLYSSTPETIALVSNQISQR  1221
              +P++ GF N E  +F++         +S++N+  +  +P +L +    E    V++++ +
Sbjct  339   KVPVLYGFANME-GLFRVGMGYEYFMSQLNDKFSENLPHDLEFKDEEEKRK-VADEVKKF  396

Query  1222  YF  1227
             YF
Sbjct  397   YF  398


> ta_transcript29751_1
Length=506

 Score =   140 bits (354),  Expect = 2e-35, Method: Compositional matrix adjust.
 Identities = 136/438 (31%), Positives = 212/438 (48%), Gaps = 39/438 (9%)
 Frame = +1

Query  172   FRGVPYAKQPVGELRFKELQPAEPWTDYLDATEEGPVCYQTDVLYGSLMKPHGMDEACIY  351
             F  +PYA  P  + RFK       W   DA +   VC Q  +  GS      + E C+
Sbjct  53    FYNIPYATAPTSKDRFKAPLAPPVWVTIRDAVDREVVCPQM-LETGS---HKNIQEDCLI  108

Query  352   ANIHVPLNALPAAGETPTKPGLPILVFIHgggfafgsgdadLYGPEYLV-TRNVVVITFN  528
             AN+ VP           ++  L ++V +HGGG+  G G+     P+ +V  + ++ +TFN
Sbjct  109   ANVFVP---------DTSEKNLSVVVLVHGGGYLSGFGNRQT--PKDMVRDKKLIYVTFN  157

Query  529   YRLNFFGFFSLDTPKVPGNNGLRDMVTLLRWVKRNARAFGGNPDNVtlagqsagaaaahl  708
             YRL   GF  L T  VPGN G++DMV LLRWVK+N  +FGGNPD+VT+ G S+G+++  L
Sbjct  158   YRLGVHGFLCLGTADVPGNAGMKDMVALLRWVKKNIASFGGNPDDVTIDGHSSGSSSVDL  217

Query  709   ltlsKATEGLVSRAILMSGAGtstfttspifsqsINKILFSILGVNSTNPDEIHEKLVA  888
             L LSKATEGL  +AI  SG+           ++S  I+ I  +     +  E  + + A
Sbjct  218   LLLSKATEGLFHKAIPESGSN-------VAMWSVQIDPIAHAKEFARDEHSFESGDDIYA  270

Query  889   -------MPVEKLNEANRILI--DQIGLTTFFPVVETPHPGITTILDEDPNILVQQGRGK  1041
                    +P + + ++   L     +     F P VE    G+    LD+ P  +++ G
Sbjct  271   LEDFYKTLPFDVMYKSGMSLRYNSSVKNILFQPCVER-DTGVEKFLDDFPVNILKSGDYN  329

Query  1042  DIPLIIGFTNSECHMFQHRFEQI-DIVSKINENPAILVPSNLLYSSTPETIALVSNQISQ  1218
              +P++ GFTN E  M    F       +S INE  +  +P++L +    E    V+  + Q
Sbjct  330   KVPMLYGFTNMEGLMRVSDFVNYRSFMSDINEKFSDYLPNDLQFRDKEEK-EKVARDVKQ  388

Query  1219  RYFNGSV----DLEGFINMCTDSYYKYPAMKLAEKRSAAGDAPVFLYQFSYDGYSVFKQA  1386
              YF   V      +  +I+  +D+ + YP ++  + +  AG+  V+LYQFSY    +  +
Sbjct  389   FYFGDKVINNDTIFEYIDYFSDNMFAYPTLRSLKLQVEAGNNQVYLYQFSYPFENQAPEG  448

Query  1387  FHLHFNGAGHADDLTYVL  1440
               L   G+ H      VL
Sbjct  449   VMLKLEGSSHTAQTHVVL  466
```

****

Query= gi|512902947|ref|XM_004925442.1| PREDICTED: Bombyx mori juvenile hormone esterase-like (LOC101744318), mRNA

Length=1629

```
                                                                     Score     E
Sequences producing significant alignments:                          (Bits)  Value

  ta_transcript58915_1                                                  508    4e-173
```

```
  ta_transcript58914_1                                              507   4e-172
  ta_transcript75783_1                                              395   1e-127
  ta_transcript48580_1                                              288   5e-86
  ta_transcript48579_1                                              287   2e-85
  ta_transcript84821_1                                              196   4e-54
  ta_transcript84818_1                                              197   1e-53
  ta_transcript84816_1                                              196   5e-53
  ta_transcript29751_1                                              182   7e-50
  ta_transcript48393_1                                              176   6e-47
  ta_transcript49309_1                                              159   4e-41
  ta_transcript30390_1                                              135   9e-33


> ta_transcript58915_1
Length=606

 Score =   508 bits (1309),  Expect = 4e-173, Method: Compositional matrix adjust.
 Identities = 257/533 (48%), Positives = 356/533 (67%), Gaps = 5/533 (1%)
 Frame = +1

Query  40    DQGILEGEELANEVDGSTLYSFKGIPYAQPPVGNLRFKAPQPVSSWEGVKNATRHGAVCP  219
             +QG LEGE+L +       +SFKGIPYA PPVG LRFKAPQP  SW+GV+ AT+HG  CP
Sbjct  35    EQGWLEGEQLDSVSGDGQFFSFKGIPYAAPPVGKLRFKAPQPPVSWQGVRKATQHGPKCP  94

Query  220   QFDILTNQIIPGDEDCLFLNIYTPDLNPATPLPVMFFIHGGGYVSGSGNDDFFGPDFIVR  399
             Q DI + QIIPG EDCL+LN+YT D++P +PLPVM FIHGGG+ SGSG++DF+G DF+V
Sbjct  95    QRDIFSKQIIPGSEDCLYLNVYTKDISPKSPLPVMVFIHGGGFKSGSGDEDFYGGDFLVH  154

Query  400   KNVILVTINYRLGDLGFLTLDTEEVPGNAGLKDQVLALKWVNENIAHFGGDPKLLTIIGQ  579
               V+LVTINYRL  LGFL LDTEEVPGNAGLKDQV ALKWV +NI  FGGDP  +TI G+
Sbjct  155   HGVVLVTINYRLDALGFLCLDTEEVPGNAGLKDQVAALKWVQKNIFAFGGDPTNVTIFGE  214

Query  580   SAGAASVLYHLGSSLTKGLFNRAIALSGVPTCDFNYSYKPIKRPYILAKLLGNDTTDLAI  759
             SAG +S   H+ S L+KGLF RAI +SGVP CD++  ++P KR + L K+LG +T D
Sbjct  215   SAGGSSTALHVLSPLSKGLFKRAIPMSGVPFCDWSIPFEPRKRAFTLGKILGFETEDPKA  274

Query  760   ALEFLQSVNVQLLIQPDVSLLATESVSTGNIFRSYYFLPVVEKDFGTEQYLTKNYLELIQ  939
              LE+LQS+  +  +  + +++  E  S  N+ + Y+F PVVEK+FG   ++T+  LE ++
Sbjct  275   LLEYLQSLPAEKFVDTNPTIMGFEEKSY-NMLKMYHFTPVVEKNFGQHHFMTEEPLEALK  333

Query  940   NGSFHDVDVYLGSTNLETAVVASNIDALIQEIIENPQLIVPREILNQVTPDVSLEIADSL  1119
              G  +DVDV +G+T+ ET V   +   L++     P++ VPR+ILN+ TP   LEIA+ +
Sbjct  334   QGHVNDVDVLIGNTDQETIVGIPSFVDLLKMYDRYPEVFVPRKILNKSTPGKILEIAERI  393

Query  1120  LQNYYGDKRLTVENIQeyltytletyFTAAIRRFADLI---AKVSSKVYFYKFESFSSRN  1290
              ++Y+GDK +   +++E +TY  E  FT  + ++ +L+      SSKVY Y+F   S RN
Sbjct  394   RKHYFGDKPIDKTSMKEAVTYFSEVCFTYDVNKYTNLLLSGKPGSSKVYRYRFSCVSERN  453

Query  1291  YYGQATVPFGFYAASHLDDLMYFMYPKSLNMTVDVSSTEFKMVDLTTNVITNFVKHGTPT  1470
              +G+    +G   ASHL+DLMY    KS+ + +D +S  +KM+  T  + TNF K+G PT
Sbjct  454   IFGKYGQEYGITGASHLEDLMYLFDAKSVKLPLDRNSKSYKMIQQTCALFTNFAKYGNPT  513

Query  1471  PDNSLGLIWPVYDTTNKAYVTISNDTLIPGSDPDSSGTAFRIGLFEKTGINY*  1629
             PD+SLG  WP YD  ++++V I+ D L  G   D+    F   +++   I Y*
Sbjct  514   PDSSLGFTWPEYDIKDQSFVDIA-DQLTVGRHLDADAVKFWESIYQYADIGY*  565


> ta_transcript58914_1
Length=650

 Score =   507 bits (1306),  Expect = 4e-172, Method: Compositional matrix adjust.
 Identities = 257/533 (48%), Positives = 356/533 (67%), Gaps = 5/533 (1%)
 Frame = +1

Query  40    DQGILEGEELANEVDGSTLYSFKGIPYAQPPVGNLRFKAPQPVSSWEGVKNATRHGAVCP  219
             +QG LEGE+L +       +SFKGIPYA PPVG LRFKAPQP  SW+GV+ AT+HG  CP
Sbjct  35    EQGWLEGEQLDSVSGDGQFFSFKGIPYAAPPVGKLRFKAPQPPVSWQGVRKATQHGPKCP  94

Query  220   QFDILTNQIIPGDEDCLFLNIYTPDLNPATPLPVMFFIHGGGYVSGSGNDDFFGPDFIVR  399
             Q DI + QIIPG EDCL+LN+YT D++P +PLPVM FIHGGG+ SGSG++DF+G DF+V
Sbjct  95    QRDIFSKQIIPGSEDCLYLNVYTKDISPKSPLPVMVFIHGGGFKSGSGDEDFYGGDFLVH  154

Query  400   KNVILVTINYRLGDLGFLTLDTEEVPGNAGLKDQVLALKWVNENIAHFGGDPKLLTIIGQ  579
               V+LVTINYRL  LGFL LDTEEVPGNAGLKDQV ALKWV +NI  FGGDP  +TI G+
Sbjct  155   HGVVLVTINYRLDALGFLCLDTEEVPGNAGLKDQVAALKWVQKNIFAFGGDPTNVTIFGE  214
```

```
Query  580   SAGAASVLYHLGSSLTKGLFNRAIALSGVPTCDFNYSYKPIKRPYILAKLLGNDTTDLAI  759
             SAG +S   H+ S L+KGLF RAI +SGVP CD++  ++P KR + L K+LG +T D
Sbjct  215   SAGGSSTALHVLSPLSKGLFKRAIPMSGVPFCDWSIPFEPRKRAFTLGKILGFETEDPKA  274

Query  760   ALEFLQSVNVQLLIQPDVSLLATESVSTGNIFRSYYFLPVVEKDFGTEQYLTKNYLELIQ  939
              LE+LQS+  +  +  + +++  E  S  N+ + Y+F PVVEK+FG   ++T+  LE ++
Sbjct  275   LLEYLQSLPAEKFVDTNPTIMGFEEKSY-NMLKMYHFTPVVEKNFGQHHFMTEEPLEALK  333

Query  940   NGSFHDVDVYLGSTNLETAVVASNIDALIQEIIENPQLIVPREILNQVTPDVSLEIADSL  1119
              G  +DVDV +G+T+ ET V   +   L++     P++ VPR+ILN+ TP   LEIA+ +
Sbjct  334   QGHVNDVDVLIGNTDQETIVGIPSFVDLLKMYDRYPEVFVPRKILNKSTPGKILEIAERI  393

Query  1120  LQNYYGDKRLTVENIQeyltytletyFTAAIRRFADLI---AKVSSKVYFYKFESFSSRN  1290
              ++Y+GDK +   +++E +TY  E  FT  + ++ +L+      SSKVY Y+F   S RN
Sbjct  394   RKHYFGDKPIDKTSMKEAVTYFSEVCFTYDVNKYTNLLLSGKPGSSKVYRYRFSCVSERN  453

Query  1291  YYGQATVPFGFYAASHLDDLMYFMYPKSLNMTVDVSSTEFKMVDLTTNVITNFVKHGTPT  1470
              +G+    +G   ASHL+DLMY    KS+ + +D +S  +KM+  T  + TNF K+G PT
Sbjct  454   IFGKYGQEYGITGASHLEDLMYLFDAKSVKLPLDRNSKSYKMIQQTCALFTNFAKYGNPT  513

Query  1471  PDNSLGLIWPVYDTTNKAYVTISNDTLIPGSDPDSSGTAFRIGLFEKTGINY*  1629
             PD+SLG  WP YD  ++++V I+ D L  G   D+    F   +++   I Y*
Sbjct  514   PDSSLGFTWPEYDIKDQSFVDIA-DQLTVGRHLDADAVKFWESIYQYADIGY*  565


> ta_transcript75783_1
Length=733

 Score =   395 bits (1015),  Expect = 1e-127, Method: Compositional matrix adjust.
 Identities = 225/533 (42%), Positives = 315/533 (59%), Gaps = 15/533 (3%)
 Frame = +1

Query  34    KRDQGILEGEELANEVDGSTLYSFKGIPYAQPPVGNLRFKAPQPVSSWEGVKNATRHGAV  213
             K  +GIL+GE + NE  GS  YSFKGIP+A+PPVG+LRFKAP P   W+GVK AT+ G +
Sbjct  127   KVSEGILQGELVQNEFGGS-YYSFKGIPFAEPPVGDLRFKAPIPAKPWKGVKTATKFGPI  185

Query  214   CPQFDILTNQIIPGDEDCLFLNIYTPDLNPATPLPVMFFIHGGGYVSGSGNDDFFGPDFI  393
               QFD+       G EDCL+LN+YTP++  P T LPVM +IHGGGY  GSGNDD +GP+F+
Sbjct  186   NYQFDVFKKDPPSGSEDCLYLNVYTPEIKPITLLPVMVWIHGGGYFWGSGNDDVYGPEFL  245

Query  394   VRKNVILVTINYRLGDLGFLTLDTEEVPGNAGLKDQVLALKWVNENIAHFGGDPKLLTII  573
             VR  VILVT NYRLG LGFL+LDT EVPGNAG+KDQV ALKWV  NI  FGGDP+ +TI
Sbjct  246   VRHGVILVTFNYRLGVLGFLSLDTAEVPGNAGMKDQVAALKWVKSNIHFFGGDPENITIF  305

Query  574   GQSAGAASVLYHLGSSLTKGLFNRAIALSGVPTCDFNYSYKPIKRPYILAKLLGNDTTDL  753
             G+SAGA +V  HL S ++KGLF RAI  SG  T  +  +++  ++   LA+ LG  T D
Sbjct  306   GESAGAGAVSLHLLSPMSKGLFKRAIIQSGSATAYWAQAFEIKEKSIALARKLGLVTDDD  365

Query  754   AIALEFLQSVNVQLLIQPDVSLLATESVSTGNIFRSYYFLPVVEKDFGTEQYLTKNYLEL  933
                  EF  +++ +LLI   V +   E     NI     F P +EKDFG E++LT +  ++
Sbjct  366   QKLYEFFGNISKELLINISVPVTFAEQEEV-NI----EFAPTIEKDFGQERFLTDDSYKI  420

Query  934   IQNGSFHDVDVYLGSTNLETAVVASNIDALIQEI--IEN-PQLIVPREILNQVTPDVSLE  1104
             +Q G    VDV +G    E  + ++++ L +EI  I N  + +VPR +  ++     L+
Sbjct  421   LQQGIHKGVDVMMGYVEDEGVLYLNSLEILDKEIERINNFLEALVPRNMKREIPIKQQLK  480

Query  1105  IADSLLQNYYGDKRLTVENIQeyltytletyFTAAIRRFADLIAKVSSKVYFYKFESFSS  1284
             +   + + Y+ +K +T  + +    Y     F+  + +    IAK S   +FYKF   S
Sbjct  481   VGRKMKKFYFDNKLVTRNDWRTIARYISLNVFSYDVIQSQKNIAK-SQNSFFYKFTCKSK  539

Query  1285  RNYYGQA----TVPFGFYAASHLDDLMYFMYPKSLNMTVDVSSTEFKMVDLTTNVITNFV  1452
             RN++ +      +        H DDL Y     K +N +V   S EF++D  T + TNF
Sbjct  540   RNFFKKVFTDDEITKDVDVVCHCDDLAYLFPVKFMNQSVAKDSREFQLIDKVTKLWTNFA  599

Query  1453  KHGTPTPDNSLGLIWPVYDTTNKAYVTISNDTLIPGSDPDSSGTAFRIGLFEK  1611
             K+G PTPDNSLG+ W  +   ++ Y+ I  D L+  + PD     F   L+ +
Sbjct  600   KYGNPTPDNSLGVKWEPFTLQHQHYLDIGED-LVLKTKPDDEDIQFWEELYRE  651


> ta_transcript48580_1
Length=834

 Score =   288 bits (737),  Expect = 5e-86, Method: Compositional matrix adjust.
 Identities = 198/546 (36%), Positives = 284/546 (52%), Gaps = 34/546 (6%)
 Frame = +1

Query  46    GILEGEELANEVDGSTLY-SFKGIPYAQPPVGNLRFKAPQPVSSWEGVKNATRHGAVCPQ  222
```

```
             G L G +        T Y SFKGI YAQPP G+LRFK P P+  W GV++A   GAVCP
Sbjct  161   GALRGRRVVARTTAQTPYFSFKGIRYAQPPRGSLRFKPPVPLEPWSGVRDALEEGAVCPH  220

Query  223   FDILTNQIIPGDEDCLFLNIYTPDL-------NPATPLPVMFFIHGGGYVSGSGNDDFFG  381
               +L +    GDEDCLFLN+YTP L       NP   L VM +IHGG +  GSGN   +G
Sbjct  221   RFMLFDTY-KGDEDCLFLNVYTPALPEKITGFNPK--LAVMVWIHGGAFAVGSGNAFLYG  277

Query  382   PDFIVRKNVILVTINYRLGDLGFLTLDTEEVPGNAGLKDQVLALKWVNENIAHFGGDPKL  561
             PD ++  N++LVT+NYRLG LGFL+++ +EV GN GLKDQVLALKWV +NI +FGGD
Sbjct  278   PDHLMGGNIVLVTLNYRLGALGFLSMENDEVSGNMGLKDQVLALKWVRDNIEYFGGDASR  337

Query  562   LTIIGQSAGAASVLYHLGSSLTKGLFNRAIALSGVPTCDFNYSYKPIKRPYILAKLLGND  741
             +TI G+SAGAASV  H+ S  +KGLF+RAIA SG+    +  +  P +R + L + LG D
Sbjct  338   VTIFGESAGAASVHLHMLSPASKGLFHRAIAQSGLALSPWALTRSPRERAFELGRELGID  397

Query  742   TTDLAIALEFLQSVNVQLLIQPDVSLLAT--ESVSTGNIFRSYYFLPVVEKDFGTEQYLT  915
             T   A  L +L++   +LL++    L     +SV   +    + F+P VE D   + +LT
Sbjct  398   TNSTAELLGYLRATPSELLVKAGARLAGAPGKSVDLHSTV-ALPFVPTVEPDV-PDAFLT  455

Query  916   KNYLELIQNGSFHDVDVYLGSTNLETAVVASNID---ALIQEIIENPQLIVPREILNQVT  1086
             ++   L+      DV +  G    E  ++   +     L+ E+    + +VP E+L
Sbjct  456   QHPRNLMPGA---DVPLLTGYNAQEGIILFRRLQRYPKLLTELDREFRRVVPPELLTGDE  512

Query  1087  PDVSLEIADSLLQNYYGDKRLTVENIQeyltytletyFTAAIRRFADLIAKV--SSKVYF  1260
                +  +AD +   Y+  + + + NI   +    +  F   +     +  K   +S  Y
Sbjct  513   AQ-NRRVADHIRAFYFQQRPVDIRNIDSLIDLFTDVMFLRPLLETLRIQGKTNRTSPTYL  571

Query  1261  YKFESFSSRNYYGQATVPFGFYAASHLDDLMYFMYPKSLNMTVDVSSTEFKMVDLTTNVI  1440
             Y+F +F       +  +      A H D++ Y  Y   LN  +D  STE  +      +
Sbjct  572   YRF-AFDGALGLFKRMLGINHPGACHGDEMGYLFYFSRLNYRLDDDSTELAVSKGMVRMW  630

Query  1441  TNFVKHGTPT---PDNSLGLIW-PVYDTTNKAYVTISNDTLIPGSDPDSSGTAFRIGLFE  1608
             TNF K G PT    D+ +   W PV DTT+  Y+ I N       DP+    A R+ L++
Sbjct  631   TNFAKTGNPTIGDHDDPIDFKWSPVNDTTHLTYLDI-NGHFTQKQDPE----ARRVRLWD  685

Query  1609  KTGINY  1626
                 NY
Sbjct  686   WLYENY  691


> ta_transcript48579_1
Length=905

 Score =   287 bits (735),  Expect = 2e-85, Method: Compositional matrix adjust.
 Identities = 198/546 (36%), Positives = 284/546 (52%), Gaps = 34/546 (6%)
 Frame = +1

Query  46    GILEGEELANEVDGSTLY-SFKGIPYAQPPVGNLRFKAPQPVSSWEGVKNATRHGAVCPQ  222
             G L G +        T Y SFKGI YAQPP G+LRFK P P+  W GV++A   GAVCP
Sbjct  161   GALRGRRVVARTTAQTPYFSFKGIRYAQPPRGSLRFKPPVPLEPWSGVRDALEEGAVCPH  220

Query  223   FDILTNQIIPGDEDCLFLNIYTPDL-------NPATPLPVMFFIHGGGYVSGSGNDDFFG  381
               +L +    GDEDCLFLN+YTP L       NP   L VM +IHGG +  GSGN   +G
Sbjct  221   RFMLFDTY-KGDEDCLFLNVYTPALPEKITGFNPK--LAVMVWIHGGAFAVGSGNAFLYG  277

Query  382   PDFIVRKNVILVTINYRLGDLGFLTLDTEEVPGNAGLKDQVLALKWVNENIAHFGGDPKL  561
             PD ++  N++LVT+NYRLG LGFL+++ +EV GN GLKDQVLALKWV +NI +FGGD
Sbjct  278   PDHLMGGNIVLVTLNYRLGALGFLSMENDEVSGNMGLKDQVLALKWVRDNIEYFGGDASR  337

Query  562   LTIIGQSAGAASVLYHLGSSLTKGLFNRAIALSGVPTCDFNYSYKPIKRPYILAKLLGND  741
             +TI G+SAGAASV  H+ S  +KGLF+RAIA SG+    +  +  P +R + L + LG D
Sbjct  338   VTIFGESAGAASVHLHMLSPASKGLFHRAIAQSGLALSPWALTRSPRERAFELGRELGID  397

Query  742   TTDLAIALEFLQSVNVQLLIQPDVSLLAT--ESVSTGNIFRSYYFLPVVEKDFGTEQYLT  915
             T   A  L +L++   +LL++    L     +SV   +    + F+P VE D   + +LT
Sbjct  398   TNSTAELLGYLRATPSELLVKAGARLAGAPGKSVDLHSTV-ALPFVPTVEPDV-PDAFLT  455

Query  916   KNYLELIQNGSFHDVDVYLGSTNLETAVVASNID---ALIQEIIENPQLIVPREILNQVT  1086
             ++   L+      DV +  G    E  ++   +     L+ E+    + +VP E+L
Sbjct  456   QHPRNLMPGA---DVPLLTGYNAQEGIILFRRLQRYPKLLTELDREFRRVVPPELLTGDE  512

Query  1087  PDVSLEIADSLLQNYYGDKRLTVENIQeyltytletyFTAAIRRFADLIAKV--SSKVYF  1260
                +  +AD +   Y+  + + + NI   +    +  F   +     +  K   +S  Y
Sbjct  513   AQ-NRRVADHIRAFYFQQRPVDIRNIDSLIDLFTDVMFLRPLLETLRIQGKTNRTSPTYL  571

Query  1261  YKFESFSSRNYYGQATVPFGFYAASHLDDLMYFMYPKSLNMTVDVSSTEFKMVDLTTNVI  1440
             Y+F +F       +  +      A H D++ Y  Y   LN  +D  STE  +      +
```

```
Sbjct  572   YRF-AFDGALGLFKRMLGINHPGACHGDEMGYLFYFSRLNYRLDDDSTELAVSKGMVRMW  630

Query  1441  TNFVKHGTPT---PDNSLGLIW-PVYDTTNKAYVTISNDTLIPGSDPDSSGTAFRIGLFE  1608
             TNF K G PT    D+ +   W PV DTT+  Y+ I N       DP+    A R+ L++
Sbjct  631   TNFAKTGNPTIGDHDDPIDFKWSPVNDTTHLTYLDI-NGHFTQKQDPE----ARRVRLWD  685

Query  1609  KTGINY  1626
                 NY
Sbjct  686   WLYENY  691


> ta_transcript84821_1
Length=611

 Score =   196 bits (499),  Expect = 4e-54, Method: Compositional matrix adjust.
 Identities = 133/380 (35%), Positives = 195/380 (51%), Gaps = 17/380 (4%)
 Frame = +1

Query  25    ERNKRDQGILEGEELANEVDGSTLYSFKGIPYAQPPVGNLRFKAPQPVSSWEGVKNATRH  204
             + N R   I +G+    +     L++F  IPYA  P G  RFKAP P   W   + A
Sbjct  34    DENSRTVEIQQGKLRGYKDPEGGLFTFYNIPYATAPTGKDRFKAPLPAPVWITTREAVDR  93

Query  205   GAVCPQFDILTNQII--PGDEDCLFLNIYTPDLNPATPLPVMFFIHGGGYVSGSGNDDFF  378
             G VC Q D+    I      EDCL  N+Y PD +    LPV+ F+HGG Y++G G  +
Sbjct  94    GVVCHQIDLAAVGIFNKTKQEDCLIANVYVPDTDKKN-LPVVVFVHGGAYLAGFG--EVL  150

Query  379   GPDFIVR-KNVILVTINYRLGDLGFLTLDTEEVPGNAGLKDQVLALKWVNENIAHFGGDP  555
              P  +VR  NVI VT NYRLG  GFL L T + PGNAG+KD V  L+WV +NIA FGG+P
Sbjct  151   TPKHMVRTNNVIYVTFNYRLGVQGFLCLGTPDAPGNAGMKDMVALLRWVKKNIASFGGNP  210

Query  556   KLLTIIGQSAGAASVLYHLGSSLTKGLFNRAIALSGVPTCDFNYSYKPIKRPYILAKL-L  732
               +TI G SAG+++V   L S  TKGLF++AI  SG     ++    PI      AK
Sbjct  211   DDVTIDGYSAGSSAVDLLLLSKATKGLFHKAIPESGANVAMWSVQVDPIANAKEYAKSEH  270

Query  733   GNDTTDLAIALE-FLQSVNVQLLIQPDVSLLATESVSTGNIFRSYYFLPVVEKDFGTEQY  909
             G +  D   ALE F +S+    + +  + L+            ++ F P VE+D G E++
Sbjct  271   GFEILDDIYALEDFYKSLPYDEMYKSGMELMFKNK-------NNFLFSPCVERDTGVEKF  323

Query  910   LTKNYLELIQNGSFHDVDVYLGSTNLETAV-VASNIDALIQEIIENPQLIVPREILNQVT  1086
             L    + +++ G ++ V V  G  N+E    V    +  + ++ +     +P + L
Sbjct  324   LDDTPVNILKKGDYNKVPVLYGFANMEGLFRVGMGYEYFMSQLNDKFSENLPHD-LEFKD  382

Query  1087  PDVSLEIADSLLQNYYGDKR  1146
              +   ++AD + + Y+GDK+
Sbjct  383   EEEKRKVADEVKKFYFGDKK  402


> ta_transcript84818_1
Length=733

 Score =   197 bits (500),  Expect = 1e-53, Method: Compositional matrix adjust.
 Identities = 133/380 (35%), Positives = 195/380 (51%), Gaps = 17/380 (4%)
 Frame = +1

Query  25    ERNKRDQGILEGEELANEVDGSTLYSFKGIPYAQPPVGNLRFKAPQPVSSWEGVKNATRH  204
             + N R   I +G+    +     L++F  IPYA  P G  RFKAP P   W   + A
Sbjct  34    DENSRTVEIQQGKLRGYKDPEGGLFTFYNIPYATAPTGKDRFKAPLPAPVWITTREAVDR  93

Query  205   GAVCPQFDILTNQII--PGDEDCLFLNIYTPDLNPATPLPVMFFIHGGGYVSGSGNDDFF  378
             G VC Q D+    I      EDCL  N+Y PD +    LPV+ F+HGG Y++G G  +
Sbjct  94    GVVCHQIDLAAVGIFNKTKQEDCLIANVYVPDTDKKN-LPVVVFVHGGAYLAGFG--EVL  150

Query  379   GPDFIVR-KNVILVTINYRLGDLGFLTLDTEEVPGNAGLKDQVLALKWVNENIAHFGGDP  555
              P  +VR  NVI VT NYRLG  GFL L T + PGNAG+KD V  L+WV +NIA FGG+P
Sbjct  151   TPKHMVRTNNVIYVTFNYRLGVQGFLCLGTPDAPGNAGMKDMVALLRWVKKNIASFGGNP  210

Query  556   KLLTIIGQSAGAASVLYHLGSSLTKGLFNRAIALSGVPTCDFNYSYKPIKRPYILAKL-L  732
               +TI G SAG+++V   L S  TKGLF++AI  SG     ++    PI      AK
Sbjct  211   DDVTIDGYSAGSSAVDLLLLSKATKGLFHKAIPESGANVAMWSVQVDPIANAKEYAKSEH  270

Query  733   GNDTTDLAIALE-FLQSVNVQLLIQPDVSLLATESVSTGNIFRSYYFLPVVEKDFGTEQY  909
             G +  D   ALE F +S+    + +  + L+            ++ F P VE+D G E++
Sbjct  271   GFEILDDIYALEDFYKSLPYDEMYKSGMELMFKNK-------NNFLFSPCVERDTGVEKF  323

Query  910   LTKNYLELIQNGSFHDVDVYLGSTNLETAV-VASNIDALIQEIIENPQLIVPREILNQVT  1086
             L    + +++ G ++ V V  G  N+E    V    +  + ++ +     +P + L
Sbjct  324   LDDTPVNILKKGDYNKVPVLYGFANMEGLFRVGMGYEYFMSQLNDKFSENLPHD-LEFKD  382
```

```
Query  1087  PDVSLEIADSLLQNYYGDKR  1146
              +   ++AD + + Y+GDK+
Sbjct  383   EEEKRKVADEVKKFYFGDKK  402


> ta_transcript84816_1
Length=947

 Score =   196 bits (499),  Expect = 5e-53, Method: Compositional matrix adjust.
 Identities = 133/380 (35%), Positives = 195/380 (51%), Gaps = 17/380 (4%)
 Frame = +1

Query  25    ERNKRDQGILEGEELANEVDGSTLYSFKGIPYAQPPVGNLRFKAPQPVSSWEGVKNATRH  204
             + N R   I +G+    +     L++F  IPYA  P G  RFKAP P   W   + A
Sbjct  34    DENSRTVEIQQGKLRGYKDPEGGLFTFYNIPYATAPTGKDRFKAPLPAPVWITTREAVDR  93

Query  205   GAVCPQFDILTNQII--PGDEDCLFLNIYTPDLNPATPLPVMFFIHGGGYVSGSGNDDFF  378
             G VC Q D+    I      EDCL  N+Y PD +    LPV+ F+HGG Y++G G  +
Sbjct  94    GVVCHQIDLAAVGIFNKTKQEDCLIANVYVPDTDKKN-LPVVVFVHGGAYLAGFG--EVL  150

Query  379   GPDFIVR-KNVILVTINYRLGDLGFLTLDTEEVPGNAGLKDQVLALKWVNENIAHFGGDP  555
              P  +VR  NVI VT NYRLG  GFL L T + PGNAG+KD V  L+WV +NIA FGG+P
Sbjct  151   TPKHMVRTNNVIYVTFNYRLGVQGFLCLGTPDAPGNAGMKDMVALLRWVKKNIASFGGNP  210

Query  556   KLLTIIGQSAGAASVLYHLGSSLTKGLFNRAIALSGVPTCDFNYSYKPIKRPYILAKL-L  732
               +TI G SAG+++V   L S  TKGLF++AI  SG     ++    PI      AK
Sbjct  211   DDVTIDGYSAGSSAVDLLLLSKATKGLFHKAIPESGANVAMWSVQVDPIANAKEYAKSEH  270

Query  733   GNDTTDLAIALE-FLQSVNVQLLIQPDVSLLATESVSTGNIFRSYYFLPVVEKDFGTEQY  909
             G +  D   ALE F +S+    + +  + L+             ++ F P VE+D G E++
Sbjct  271   GFEILDDIYALEDFYKSLPYDEMYKSGMELMFKNK-------NNFLFSPCVERDTGVEKF  323

Query  910   LTKNYLELIQNGSFHDVDVYLGSTNLETAV-VASNIDALIQEIIENPQLIVPREILNQVT  1086
             L    + +++ G ++ V V  G  N+E    V    +  + ++ +     +P + L
Sbjct  324   LDDTPVNILKKGDYNKVPVLYGFANMEGLFRVGMGYEYFMSQLNDKFSENLPHD-LEFKD  382

Query  1087  PDVSLEIADSLLQNYYGDKR  1146
               +   ++AD + + Y+GDK+
Sbjct  383   EEEKRKVADEVKKFYFGDKK  402


> ta_transcript29751_1
Length=506

 Score =   182 bits (463),  Expect = 7e-50, Method: Compositional matrix adjust.
 Identities = 161/493 (33%), Positives = 247/493 (50%), Gaps = 30/493 (6%)
 Frame = +1

Query  40    DQGILEGEELANEVDGSTLYSFKGIPYAQPPVGNLRFKAPQPVSSWEGVKNATRHGAVCP  219
             +QG + G +   + DG  LY F  IPYA  P    RFKAP     W  +++A     VCP
Sbjct  36    EQGPVRGYK---DPDGG-LYVFYNIPYATAPTSKDRFKAPLAPPVWVTIRDAVDREVVCP  91

Query  220   QFDILTNQIIPGDEDCLFLNIYTPDLNPATPLPVMFFIHGGGYVSGSGNDDFFGPDFIVR  399
             Q  + T       EDCL  N++ PD +    L V+  +HGGGY+SG GN      P  +VR
Sbjct  92    QM-LETGSHKNIQEDCLIANVFVPDTSEKN-LSVVVLVHGGGYLSGFGNRQ--TPKDMVR  147

Query  400   -KNVILVTINYRLGDLGFLTLDTEEVPGNAGLKDQVLALKWVNENIAHFGGDPKLLTIIG  576
              K +I VT NYRLG  GFL L T +VPGNAG+KD V  L+WV +NIA FGG+P  +TI G
Sbjct  148   DKKLIYVTFNYRLGVHGFLCLGTADVPGNAGMKDMVALLRWVKKNIASFGGNPDDVTIDG  207

Query  577   QSAGAASVLYHLGSSLTKGLFNRAIALSGVPTCDFNYSYKPIKRPYILAKLLGN-DTTDL  753
              S+G++SV   L S  T+GLF++AI  SG     ++    PI      A+   + ++ D
Sbjct  208   HSSGSSSVDLLLLSKATEGLFHKAIPESGSNVAMWSVQIDPIAHAKEFARDEHSFESGDD  267

Query  754   AIALE-FLQSVNVQLLIQPDVSLLATESVSTGNIFRSYYFLPVVEKDFGTEQYLTKNYLE  930
               ALE F +++   ++ +  +SL    SV      ++  F P VE+D G E++L    +
Sbjct  268   IYALEDFYKTLPFDVMYKSGMSLRYNSSV------KNILFQPCVERDTGVEKFLDDFPVN  321

Query  931   LIQNGSFHDVDVYLGSTNLETAVVAS---NIDALIQEIIENPQLIVPREILNQVTPDVSL  1101
             ++++G ++ V +  G TN+E  +  S   N  + + +I E     +P + L     +
Sbjct  322   ILKSGDYNKVPMLYGFTNMEGLMRVSDFVNYRSFMSDINEKFSDYLPND-LQFRDKEEKE  380

Query  1102  EIADSLLQNYYGDKRLTVENIQeyltytletyFT-AAIRRFADLIAKVSSKVYFYKFESF  1278
             ++A  + Q Y+GDK +  + I EY+ Y  +  F    +R     +   +++VY Y+F S+
Sbjct  381   KVARDVKQFYFGDKVINNDTIFEYIDYFSDNMFAYPTLRSLKLQVEAGNNQVYLYQF-SY  439
```

```
Query  1279  SSRNYYGQATVPFGFYAASHLDDLMYFMYPKSLNMTVDVSSTEFKMVDLTTNVITNFVKH  1458
                N   +  V      +SH       +   +LN   D++       ++T     NF+
Sbjct  440   PFENQAPEG-VMLKLEGSSHTAQTHVVL--DTLNEENDLAIIR----NITRQFWANFITT  492

Query  1459  GTPTPDNSLGLIW  1497
             GTP P  S    W
Sbjct  493   GTPVPAGSELPAW  505
```

```
> ta_transcript48393_1
Length=666

 Score =   176 bits (447),  Expect = 6e-47, Method: Compositional matrix adjust.
 Identities = 124/365 (34%), Positives = 181/365 (50%), Gaps = 27/365 (7%)
 Frame = +1

Query  94    LYSFKGIPYAQPPVGNLRFKAPQPVSSWEGVKNATRHGAVCPQFDILTNQIIPGDEDCLF  273
             L++F  IPYA  P G L+F AP    +W     A     +CPQ      + +    DCL
Sbjct  113   LFAFYSIPYATAPSGPLKFTAPITPPTWTEPFEAVDKRIICPQMKSGFTKQMTMQVDCLI  172

Query  274   LNIYTPDLNPATPLPVMFFIHGGGYVSGSGNDDFFGPDFIVR-KNVILVTINYRLGDLGF  450
              N+Y PD    T LPV+ ++HGG +  G GN     P  +VR KN+I VT NYRLG  GF
Sbjct  173   ANVYVPDTK-ETKLPVIVYVHGGAFQVGFGN--LMTPKNLVRSKNIIAVTFNYRLGAHGF  229

Query  451   LTLDTEEVPGNAGLKDQVLALKWVNENIAHFGGDPKLLTIIGQSAGAASVLYHLGSSLTK  630
             L L TE+ PGNAGLKDQV AL+WVN+NI   GG+P  +TI G SAGA SV     S   +
Sbjct  230   LCLGTEDAPGNAGLKDQVAALRWVNKNIIKLGGNPDDVTIAGYSAGAISVDLMAISKSAE  289

Query  631   GLFNRAIALSGVPTCDFNYSYKPIKRPYILAKLLGNDTTDLAIALE-FLQSVNV------  789
             GLF++ I  SG      +    P++   I A  LG +  D   ALE F ++ +
Sbjct  290   GLFSKIIIESGSSLFAGSIVTDPLENAKIFAARLGFNNIDDVYALEKFYKAASYDSLTKG  349

Query  790   QLLIQPDVSLLATESVSTGNIFRSYYFLPVVEKDFGTEQYLTKNYLELIQNGSFHDVDVY  969
             + + +P+ +LL               F P VE+D     +L++  + +++ GS+  V +
Sbjct  350   EFMDRPNSTLL---------------FSPCVERDDDGLAFLSEPPINILKEGSYTQVPMI  394

Query  970   LGSTNLETAVVASNIDALIQEIIENPQLIVPREILNQVTPDVSLEIADSLLQNYYGDKRL  1149
              G +N+E        +    ++ E     +P ++  +   D    IA  + + Y+ D  +
Sbjct  395   YGFSNMEGLFREPFFEFWKLKMNEQFSEFIPGDLHFESKHDRE-RIAKRIKKFYFWDSPI  453

Query  1150  TVENI  1164
               ENI
Sbjct  454   NGENI  458
```

```
> ta_transcript49309_1
Length=664

 Score =   159 bits (401),  Expect = 4e-41, Method: Compositional matrix adjust.
 Identities = 92/215 (43%), Positives = 120/215 (56%), Gaps = 29/215 (13%)
 Frame = +1

Query  100  SFKGIPYAQPPVGNLRFKAPQPVS--SWEGVKNATRHGAVCPQFD---------------  228
            ++ GIPYAQPP+  LRF  P+ ++   WEGV NAT     C Q D
Sbjct  52   AYVGIPYAQPPIDRLRFMPPEYLNPPQWEGVYNATIFAPDCMQSDPKKDDVQSTLKKHDE  111

Query  229  ----ILTNQI-IPGD----EDCLFLNIYTPDLNPATPLPVMFFIHGGGYVSGSGNDDFFG  381
                +L +Q+  P +    EDCL+LN+Y PD        PVM + HGG +V GS N  +
Sbjct  112  LFMKLLDSQLETPREKNYSEDCLYLNVYVPDDYKVEGYPVMVWFHGGEFVRGSPN--YMN  169

Query  382  P-DFIVRKNVILVTINYRLGDLGFLTLDTEEVPGNAGLKDQVLALKWVNENIAHFGGDPK  558
            P  ++++ VI V++ YRL   GF T    E  GN GL DQV AL WV  NI HFGGDP+
Sbjct  170  PFHLVLKQKVIFVSVAYRLNIFGFFTTLDHEALGNFGLHDQVAALSWVKTNIEHFGGDPE  229

Query  559   LLTIIGQSAGAASVLYHLGSSLTKGLFNRAIALSG  663
              + I G  AGA SV  HL S+ + GLF++AIA+SG
Sbjct  230  NICIFGHDAGAVSVGLHLVSTYSPGLFHKAIAMSG  264
```

```
> ta_transcript30390_1
Length=839

 Score =   135 bits (340),  Expect = 9e-33, Method: Compositional matrix adjust.
 Identities = 92/246 (37%), Positives = 127/246 (52%), Gaps = 13/246 (5%)
 Frame = +1

Query  97   YSFKGIPYAQPPVGNLRFKAPQPVSSW-EGVKNATRHGAVCPQFDILTNQIIPGDEDCLF  273
```

```
            YSF GI YA+PPVG  RF+  +PV     G   A ++ + CPQ D L    I G EDCL
Sbjct  184  YSFFGIRYAEPPVGERRFQ--RPVRRLLAGEMTAQQYCSPCPQVDPLNPYRIIGSEDCLC  241

Query  274  LNIYTPDLNPATP-LPVMFFIHGGGYVSGSGNDDFFGPDFIVRKNVILVTINYRLGDLGF  450
            LN+YTP +       PV+FFIHGG Y +GS +    +G    +  K+ ILVT  YRLG LG+
Sbjct  242  LNVYTPKMPGGEKGSPVIFFIHGGNYKTGSASA--YGGQHLTAKDTILVTAQYRLGSLGY  299

Query  451  LTLDTEEVPGNAGLKDQVLALKWVNENIAHFGGDPKLLTIIGQ-SAGAASVLYHLGSS--  621
             +   +   GNAGL D   A+ W+ E I  F GDP  + ++GQ S G+A+ L  L +
Sbjct  300  FSTAEKAASGNAGLFDLRAAMTWIKEYIEFFNGDPTRVVVMGQGSGGSAASLLALSNEGR  359

Query  622  LTKGLFNRAIALSGVPTCDFNYSYKPIKRPYILAKLLGNDTTDLAIALEFLQSVNVQLLI  801
               GL     ALSG P         P K    LAK      +     +  L+ + ++ ++
Sbjct  360  TATGL----AALSGTPLSPGAVRPDPAKFAKELAKRTNCPESPAIRLVNCLKKLPMEKIV  415

Query  802  QPDVSL  819
              DV++
Sbjct  416  LADVNM  421

*************************************************************************************************
***********************************************************************

Query= gi|512903791:16-1749 PREDICTED: Bombyx mori juvenile hormone esterase-like
(LOC101743778), mRNA

Length=1734
                                                                      Score     E
Sequences producing significant alignments:                          (Bits)  Value

  ta_transcript48580_1                                                  254    2e-73
  ta_transcript48579_1                                                  254    5e-73
  ta_transcript75783_1                                                  238    4e-68
  ta_transcript58915_1                                                  234    2e-67
  ta_transcript58914_1                                                  234    3e-67
  ta_transcript84818_1                                                  173    1e-45
  ta_transcript84821_1                                                  172    2e-45
  ta_transcript84816_1                                                  174    4e-45
  ta_transcript48393_1                                                  171    8e-45
  ta_transcript29751_1                                                  166    6e-44
  ta_transcript82619_1                                                  160    5e-41
  ta_transcript82614_1                                                  160    9e-41
  ta_transcript82616_1                                                  160    9e-41
  ta_transcript82623_1                                                  135    8e-34
  ta_transcript49309_1                                                  126    8e-30

> ta_transcript48580_1
Length=834

 Score =   254 bits (650),  Expect = 2e-73, Method: Compositional matrix adjust.
 Identities = 180/528 (34%), Positives = 281/528 (53%), Gaps = 45/528 (9%)
 Frame = +1

Query  73    GVLVNIDSGPVCGKVEIAENS--TKYYSFQGIPYGKPPTGARRFAELEPVEPWTDLLYAY  246
             G +V  + G + G+  +A  +  T Y+SF+GI Y +PP G+ RF    P+EPW+ +  A
Sbjct  153   GPIVMTELGALRGRRVVARTTAQTPYFSFKGIRYAQPPRGSLRFKPPVLEPWSGVRDAL  212

Query  247   EEGPACPSRDIIYGSITIKAKGVSEDCIYANVFVPATAQLNTDELSEVSLLPILVNIHGG  426
             EEG  CP R +++ +     KG  EDC++ NV+ PA  +  T    +   L ++V IHGG
Sbjct  213   EEGAVCPHRFMLFDTY----KG-DEDCLFLNVYTPALPEKITGFNPK---LAVMVWIHGG  264

Query  427   AFNAGSGNRDLHGPELLMVKDVIVINFNYRLAVFGFLSLASDKIPGNNGLRDMVTLLRWV  606
             AF  GSGN  L+GP+ LM  +++++  NYRL   GFLS+ +D++ GN GL+D V  L+WV
Sbjct  265   AFAVGSGNAFLYGPDHLMGGNIVLVTLNYRLGALGFLSMENDEVSGNMGLKDQVLALKWV  324

Query  607   QRNARVFGGDPDKVTVLGESAGAASVNLLMMSDAAKGLFNKAIIMSGTAFPSFYSTSAVY  786
             + N   FGGD  +VT+ GESAGAASV+L M+S A+KGLF++AI  SG A   +  T +
Sbjct  325   RDNIEYFGGDASRVTIFGESAGAASVHLHMLSPASKGLFHRAIAQSGLALSPWALTRSPR  384

Query  787   AKYVADFFLG-QLGVNSTDPDEIHQILIELPLEDIMKA--------NDVVQYNSGIA-SF  936
              +    F LG +LG+++    E+   L   P E ++KA           V  +S +A  F
Sbjct  385   ER---AFELGRELGIDTNSTAELLGYLRATPSELLVKAGARLAGAPGKSVDLHSTVALPF  441

Query  937   VPVVEIEGHNYTRIIDDDPIVLIAEGRGKDIPLLMGFNQNEGET-FRWLLTIMDALTRYK  1113
             VP VE +  +    +   P  L+    G D+PLL G+N  EG   FR L      LT
Sbjct  442   VPTVEPDVPD--AFLTQHPRNLMP---GADVPLLTGYNAQEGIILFRRLQRYPKLLTELD  496

Query  1114  TNPAAILSPRLAYELSTeealakaelvakR--YFDGE----VTMDGFIKSVTDQLFQYPI  1275
```

```
                  ++ P L   L+ +EA  +      R  YF         +D  I   TD +F  P+
Sbjct  497   REFRRVVPPEL---LTGDEAQNRRVADHIRAFYFQQRPVDIRNIDSLIDLFTDVMFLRPL  553

Query  1276  IKLAQWRTLLN-SAPTFLYVFSYQGDISIIKRSNWLSYNGTGHVEDLTYVFRTTTFLRDH  1452
             ++  + +   N ++PT+LY F++ G + + KR   +++ G  H +++ Y+F    F R +
Sbjct  554   LETLRIQGKTNRTSPTYLYRFAFDGALGLFKRMLGINHPGACHGDEMGYLF---YFSRLN  610

Query  1453  VSIPPQTRDDHMRDWMSTLFSNYVKCNNPTCTESDDP---QWPPINQT  1587
               +   + +  +   M  +++N+ K  NPT  + DDP   +W P+N T
Sbjct  611   YRLDDDSTELAVSKGMVRMWTNFAKTGNPTIGDHDDPIDFKWSPVNDT  658


> ta_transcript48579_1
Length=905

 Score =   254 bits (650),  Expect = 5e-73, Method: Compositional matrix adjust.
 Identities = 180/528 (34%), Positives = 281/528 (53%), Gaps = 45/528 (9%)
 Frame = +1

Query  73    GVLVNIDSGPVCGKVEIAENS--TKYYSFQGIPYGKPPTGARRFAELEPVEPWTDLLYAY  246
             G +V  + G + G+  +A  +  T Y+SF+GI Y +PP G+ RF    P+EPW+ +  A
Sbjct  153   GPIVMTELGALRGRRVVARTTAQTPYFSFKGIRYAQPPRGSLRFKPPVPLEPWSGVRDAL  212

Query  247   EEGPACPSRDIIYGSITIKAKGVSEDCIYANVFVPATAQLNTDELSEVSLLPILVNIHGG  426
             EEG  CP R +++ +     KG  EDC++ NV+ PA  +  T    +   L ++V IHGG
Sbjct  213   EEGAVCPHRFMLFDTY----KG-DEDCLFLNVYTPALPEKITGFNPK---LAVMVWIHGG  264

Query  427   AFNAGSGNRDLHGPELLMVKDVIVINFNYRLAVFGFLSLASDKIPGNNGLRDMVTLLRWV  606
             AF  GSGN  L+GP+ LM  +++++  NYRL   GFLS+ +D++ GN GL+D V  L+WV
Sbjct  265   AFAVGSGNAFLYGPDHLMGGNIVLVTLNYRLGALGFLSMENDEVSGNMGLKDQVLALKWV  324

Query  607   QRNARVFGGDPDKVTVLGESAGAASVNLLMMSDAAKGLFNKAIIMSGTAFPSFYSTSAVY  786
             + N   FGGD  +VT+ GESAGAASV+L M+S A+KGLF++AI  SG A   +  T +
Sbjct  325   RDNIEYFGGDASRVTIFGESAGAASVHLHMLSPASKGLFHRAIAQSGLALSPWALTRSPR  384

Query  787   AKYVADFFLG-QLGVNSTDPDEIHQILIELPLEDIMKA--------NDVVQYNSGIA-SF  936
              +    F LG +LG+++    E+   L   P E ++KA           V  +S +A  F
Sbjct  385   ER---AFELGRELGIDTNSTAELLGYLRATPSELLVKAGARLAGAPGKSVDLHSTVALPF  441

Query  937   VPVVEIEGHNYTRIIDDDPIVLIAEGRGKDIPLLMGFNQNEGET-FRWLLTIMDALTRYK  1113
             VP VE +  +    +   P  L+    G D+PLL G+N  EG   FR L      LT
Sbjct  442   VPTVEPDVPD--AFLTQHPRNLMP---GADVPLLTGYNAQEGIILFRRLQRYPKLLTELD  496

Query  1114  TNPAAILSPRLAYELSTeealakaelvakR--YFDGE----VTMDGFIKSVTDQLFQYPI  1275
                  ++ P L   L+ +EA  +      R  YF         +D  I   TD +F  P+
Sbjct  497   REFRRVVPPEL---LTGDEAQNRRVADHIRAFYFQQRPVDIRNIDSLIDLFTDVMFLRPL  553

Query  1276  IKLAQWRTLLN-SAPTFLYVFSYQGDISIIKRSNWLSYNGTGHVEDLTYVFRTTTFLRDH  1452
             ++  + +   N ++PT+LY F++ G + + KR   +++ G  H +++ Y+F    F R +
Sbjct  554   LETLRIQGKTNRTSPTYLYRFAFDGALGLFKRMLGINHPGACHGDEMGYLF---YFSRLN  610

Query  1453  VSIPPQTRDDHMRDWMSTLFSNYVKCNNPTCTESDDP---QWPPINQT  1587
               +   + +  +   M  +++N+ K  NPT  + DDP   +W P+N T
Sbjct  611   YRLDDDSTELAVSKGMVRMWTNFAKTGNPTIGDHDDPIDFKWSPVNDT  658


> ta_transcript75783_1
Length=733

 Score =   238 bits (608),  Expect = 4e-68, Method: Compositional matrix adjust.
 Identities = 158/524 (30%), Positives = 262/524 (50%), Gaps = 33/524 (6%)
 Frame = +1

Query  82    VNIDSGPVCGKVEIAENSTKYYSFQGIPYGKPPTGARRFAELEPVEPWTDLLYAYEEGPA  261
             V +  G + G++   E    YYSF+GIP+ +PP G  RF    P +PW  +  A + GP
Sbjct  126   VKVSEGILQGELVQNEFGGSYYSFKGIPFAEPPVGDLRFKAPIPAKPWKGVKTATKFGPI  185

Query  262   CPSRDIIYGSITIKAKGVSEDCIYANVFVPATAQLNTDELSEVSLLPILVNIHGGAFNAG  441
                 D+            SEDC+Y NV+ P        E+  ++LLP++V IHGG +  G
Sbjct  186   NYQFDVFKKD----PPSGSEDCLYLNVYTP--------EIKPITLLPVMVWIHGGGYFWG  233

Query  442   SGNRDLHGPELLMVKDVIVINFNYRLAVFGFLSLASDKIPGNNGLRDMVTLLRWVQRNAR  621
             SGN D++GPE L+   VI++ FNYRL V GFLSL + ++PGN G++D V  L+WV+ N
Sbjct  234   SGNDDVYGPEFLVRHGVILVTFNYRLGVLGFLSLDTAEVPGNAGMKDQVAALKWVKSNIH  293

Query  622   VFGGDPDKVTVLGESAGAASVNLLMMSDAAKGLFNKAIIMSGTAFPSFYSTSAVYAKYVA  801
              FGGDP+ +T+ GESAGA +V+L ++S  +KGLF +AII SG+A   +     +  K +A
```

```
Sbjct  294   FFGGDPENITIFGESAGAGAVSLHLLSPMSKGLFKRAIIQSGSATAYWAQAFEIKEKSIA  353

Query  802   DFFLGQLGVNSTDPDEIHQILIELPLEDIMKANDVVQYNSGIA---SFVPVVEIEGHNYT  972
                  +LG+ + D  ++++    +  E ++  +  V +         F P +E +
Sbjct  354   --LARKLGLVTDDDQKLYEFFGNISKELLINISVPVTFAEQEEVNIEFAPTIE-KDFGQE  410

Query  973   RIIDDDPIVLIAEGRGKDIPLLMGFNQNEGETFRWLLTIMDALTRYKTNPAAILSPR-LA  1149
             R + DD   ++ +G  K + ++MG+ ++EG  +   L I+D       N    L PR +
Sbjct  411   RFLTDDSYKILQQGIHKGVDVMMGYVEDEGVLYLNSLEILDKEIERINNFLEALVPRNMK  470

Query  1150  YELSTeealakaelvakRYFDGEVTMDG----FIKSVTDQLFQYPIIKLAQWRTLLNSAP  1317
              E+  ++ L     + K YFD ++           + ++  +F Y +I+    + +  S
Sbjct  471   REIPIKQQLKVGRKMKKFYFDNKLVTRNDWRTIARYISLNVFSYDVIQ--SQKNIAKSQN  528

Query  1318  TFLYVFSYQGDISIIKR-----SNWLSYNGTGHVEDLTYVFRTTTFLRDHVSIPPQTRDD  1482
             +F Y F+ +   +  K+            +   H +DL Y+F    F+    S+   +R+
Sbjct  529   SFFYKFTCKSKRNFFKKVFTDDEITKDVDVVCHCDDLAYLF-PVKFMNQ--SVAKDSREF  585

Query  1483  HMRDWMSTLFSNYVKCNNPTCTESDDPQWPPINQTELMYQLIKE  1614
              + D ++ L++N+ K  NPT   S   +W P       Y  I E
Sbjct  586   QLIDKVTKLWTNFAKYGNPTPDNSLGVKWEPFTLQHQHYLDIGE  629
```

```
> ta_transcript58915_1
Length=606

 Score =   234 bits (597),  Expect = 2e-67, Method: Compositional matrix adjust.
 Identities = 163/518 (31%), Positives = 251/518 (48%), Gaps = 47/518 (9%)
 Frame = +1

Query  82    VNIDSGPVCG-KVEIAENSTKYYSFQGIPYGKPPTGARRFAELEPVEPWTDLLYAYEEGP  258
             V ++ G + G +++      +++SF+GIPY  PP G  RF   +P   W  +  A + GP
Sbjct  32    VQVEQGWLEGEQLDSVSGDGQFFSFKGIPYAAPPVGKLRFKAPQPPVSWQGVRKATQHGP  91

Query  259   ACPSRDIIYGSITIKAKGVSEDCIYANVFVPATAQLNTDELSEVSLLPILVNIHGGAFNA  438
              CP RDI    I       SEDC+Y NV+        T ++S  S LP++V IHGG F +
Sbjct  92    KCPQRDIFSKQIIPG----SEDCLYLNVY--------TKDISPKSPLPVMVFIHGGGFKS  139

Query  439   GSGNRDLHGPELLMVKDVIVINFNYRLAVFGFLSLASDKIPGNNGLRDMVTLLRWVQRNA  618
             GSG+ D +G + L+   V+++  NYRL   GFL L ++++PGN GL+D V  L+WVQ+N
Sbjct  140   GSGDEDFYGGDFLVHHGVVLVTINYRLDALGFLCLDTEEVPGNAGLKDQVAALKWVQKNI  199

Query  619   RVFGGDPDKVTVLGESAGAASVNLLMMSDAAKGLFNKAIIMSGTAFPSFYSTSAVYAKYV  798
               FGGDP  VT+ GESAG +S  L ++S  +KGLF +AI MSG  F  +   S  +
Sbjct  200   FAFGGDPTNVTIFGESAGGSSTALHVLSPLSKGLFKRAIPMSGVPFCDW---SIPFEPRK  256

Query  799   ADFFLGQ-LGVNSTDPDEIHQILIELPLEDIMKANDVV------QYNS-GIASFVPVVEI  954
               F LG+ LG  + DP  + + L  LP E  +  N  +       YN   +  F PVVE
Sbjct  257   RAFTLGKILGFETEDPKALLEYLQSLPAEKFVDTNPTIMGFEEKSYNMLKMYHFTPVVE-  315

Query  955   EGHNYTRIIDDDPIVLIAEGRGKDIPLLMGFNQNEGETFRWLLTIMDALTRYKTNPAAIL  1134
             +       + ++P+  + +G  D+ +L+G    + ET   + + +D L  Y   P  +
Sbjct  316   KNFGQHHFMTEEPLEALKQGHVNDVDVLIG--NTDQETIVGIPSFVDLLKMYDRYP-EVF  372

Query  1135  SPRLAYELSTeealakaelvakRYFDGE-----VTMDGFIKSVTDQLFQYPIIKLAQWRT  1299
              PR     ST   + +    ++++ G+      +M   +   ++  F Y + K
Sbjct  373   VPRKILNKSTPGKILEIAERIRKHYFGDKPIDKTSMKEAVTYFSEVCFTYDVNKYTN--L  430

Query  1300  LLNSAP----TFLYVFSYQGDISII-KRSNWLSYNGTGHVEDLTYVFRTTTFLRDHVSIP  1464
             LL+  P     + Y FS   + +I  K         G  H+EDL Y+F   +     V +P
Sbjct  431   LLSGKPGSSKVYRYRFSCVSERNIFGKYGQEYGITGASHLEDLMYLFDAKS-----VKLP  485

Query  1465  --PQTRDDHMRDWMSTLFSNYVKCNNPTCTESDDPQWP  1572
                 ++   M      LF+N+ K  NPT   S    WP
Sbjct  486   LDRNSKSYKMIQQTCALFTNFAKYGNPTPDSSLGFTWP  523
```

```
> ta_transcript58914_1
Length=650

 Score =   234 bits (597),  Expect = 3e-67, Method: Compositional matrix adjust.
 Identities = 163/518 (31%), Positives = 251/518 (48%), Gaps = 47/518 (9%)
 Frame = +1

Query  82    VNIDSGPVCG-KVEIAENSTKYYSFQGIPYGKPPTGARRFAELEPVEPWTDLLYAYEEGP  258
             V ++ G + G +++      +++SF+GIPY  PP G  RF   +P   W  +  A + GP
Sbjct  32    VQVEQGWLEGEQLDSVSGDGQFFSFKGIPYAAPPVGKLRFKAPQPPVSWQGVRKATQHGP  91
```

```
Query  259    ACPSRDIIYGSITIKAKGVSEDCIYANVFVPATAQLNTDELSEVSLLPILVNIHGGAFNA  438
               CP RDI    I       SEDC+Y NV+        T ++S  S LP++V IHGG F +
Sbjct  92     KCPQRDIFSKQIIPG----SEDCLYLNVY--------TKDISPKSPLPVMVFIHGGGFKS  139

Query  439    GSGNRDLHGPELLMVKDVIVINFNYRLAVFGFLSLASDKIPGNNGLRDMVTLLRWVQRNA  618
              GSG+ D +G + L+   V+++  NYRL   GFL L ++++PGN GL+D V  L+WVQ+N
Sbjct  140    GSGDEDFYGGDFLVHHGVVLVTINYRLDALGFLCLDTEEVPGNAGLKDQVAALKWVQKNI  199

Query  619    RVFGGDPDKVTVLGESAGAASVNLLMMSDAAKGLFNKAIIMSGTAFPSFYSTSAVYAKYV  798
                FGGDP  VT+ GESAG +S  L ++S  +KGLF +AI MSG  F  +   S  +
Sbjct  200    FAFGGDPTNVTIFGESAGGSSTALHVLSPLSKGLFKRAIPMSGVPFCDW---SIPFEPRK  256

Query  799    ADFFLGQ-LGVNSTDPDEIHQILIELPLEDIMKANDVV------QYNS-GIASFVPVVEI  954
                F LG+ LG  + DP  + + L  LP E  +  N  +       YN   +  F PVVE
Sbjct  257    RAFTLGKILGFETEDPKALLEYLQSLPAEKFVDTNPTIMGFEEKSYNMLKMYHFTPVVE-  315

Query  955    EGHNYTRIIDDDPIVLIAEGRGKDIPLLMGFNQNEGETFRWLLTIMDALTRYKTNPAAIL  1134
              +       + ++P+  + +G   D+ +L+G    + ET   + + +D L  Y   P  +
Sbjct  316    KNFGQHHFMTEEPLEALKQGHVNDVDVLIG--NTDQETIVGIPSFVDLLKMYDRYP-EVF  372

Query  1135   SPRLAYELSTeealakaelvakRYFDGE-----VTMDGFIKSVTDQLFQYPIIKLAQWRT  1299
               PR     ST   + +     ++++ G+      +M   +   ++  F Y + K
Sbjct  373    VPRKILNKSTPGKILEIAERIRKHYFGDKPIDKTSMKEAVTYFSEVCFTYDVNKYTN--L  430

Query  1300   LLNSAP----TFLYVFSYQGDISII-KRSNWLSYNGTGHVEDLTYVFRTTTFLRDHVSIP  1464
              LL+  P     + Y FS   + +I  K        G  H+EDL Y+F   +     V +P
Sbjct  431    LLSGKPGSSKVYRYRFSCVSERNIFGKYGQEYGITGASHLEDLMYLFDAKS-----VKLP  485

Query  1465   --PQTRDDHMRDWMSTLFSNYVKCNNPTCTESDDPQWP  1572
                  ++   M      LF+N+ K  NPT   S    WP
Sbjct  486    LDRNSKSYKMIQQTCALFTNFAKYGNPTPDSSLGFTWP  523


> ta_transcript84818_1
Length=733

 Score =   173 bits (439),  Expect = 1e-45, Method: Compositional matrix adjust.
 Identities = 110/308 (36%), Positives = 163/308 (53%), Gaps = 16/308 (5%)
 Frame = +1

Query  145    YSFQGIPYGKPPTGARRFAELEPVEPWTDLLYAYEEGPACPSRDIIYGSITIKAKGVSED  324
              ++F  IPY   PTG  RF    P   W     A + G  C   D+   ++ I  K    ED
Sbjct  58     FTFYNIPYATAPTGKDRFKAPLPAPVWITTREAVDRGVVCHQIDL--AAVGIFNKTKQED  115

Query  325    CIYANVFVPATAQLNTDELSEVSLLPILVNIHGGAFNAGSGNRDLHGPELLMVKDVIVIN  504
              C+ ANV+VP T + N         LP++V +HGGA+ AG G   L    ++   +VI +
Sbjct  116    CLIANVYVPDTDKKN---------LPVVVFVHGGAYLAGFGEV-LTPKHMVRTNNVIYVT  165

Query  505    FNYRLAVFGFLSLASDKIPGNNGLRDMVTLLRWVQRNARVFGGDPDKVTVLGESAGAASV  684
              FNYRL V GFL L +   PGN G++DMV LLRWV++N   FGG+PD VT+ G SAG+++V
Sbjct  166    FNYRLGVQGFLCLGTPDAPGNAGMKDMVALLRWVKKNIASFGGNPDDVTIDGYSAGSSAV  225

Query  685    NLLMMSDAAKGLFNKAIIMSGTAFPSFYSTSAVYAKYVADFFLGQLGVNSTDPD-EIHQI  861
              +LL++S A KGLF+KAI  SG A  + +S          ++   + G    D    +
Sbjct  226    DLLLLSKATKGLFHKAIPESG-ANVAMWSVQVDPIANAKEYAKSEHGFEILDDIYALEDF  284

Query  862    LIELPLEDIMKAN-DVVQYNSGIASFVPVVEIEGHNYTRIIDDDPIVLIAEGRGKDIPLL  1038
                 LP +++ K+  +++  N     F P VE +     + +DD P+ ++ +G    +P+L
Sbjct  285    YKSLPYDEMYKSGMELMFKNKNNFLFSPCVERD-TGVEKFLDDTPVNILKKGDYNKVPVL  343

Query  1039   MGFNQNEG  1062
               GF   EG
Sbjct  344    YGFANMEG  351


> ta_transcript84821_1
Length=611

 Score =   172 bits (436),  Expect = 2e-45, Method: Compositional matrix adjust.
 Identities = 110/308 (36%), Positives = 163/308 (53%), Gaps = 16/308 (5%)
 Frame = +1

Query  145    YSFQGIPYGKPPTGARRFAELEPVEPWTDLLYAYEEGPACPSRDIIYGSITIKAKGVSED  324
              ++F  IPY   PTG  RF    P   W     A + G  C   D+   ++ I  K    ED
Sbjct  58     FTFYNIPYATAPTGKDRFKAPLPAPVWITTREAVDRGVVCHQIDL--AAVGIFNKTKQED  115
```

```
Query  325   CIYANVFVPATAQLNTDELSEVSLLPILVNIHGGAFNAGSGNRDLHGPELLMVKDVIVIN  504
             C+ ANV+VP T + N         LP++V +HGGA+ AG G   L    ++   +VI +
Sbjct  116   CLIANVYVPDTDKKN---------LPVVVFVHGGAYLAGFGEV-LTPKHMVRTNNVIYVT  165

Query  505   FNYRLAVFGFLSLASDKIPGNNGLRDMVTLLRWVQRNARVFGGDPDKVTVLGESAGAASV  684
             FNYRL V GFL L +   PGN G++DMV LLRWV++N   FGG+PD VT+ G SAG+++V
Sbjct  166   FNYRLGVQGFLCLGTPDAPGNAGMKDMVALLRWVKKNIASFGGNPDDVTIDGYSAGSSAV  225

Query  685   NLLMMSDAAKGLFNKAIIMSGTAFPSFYSTSAVYAKYVADFFLGQLGVNSTDPD-EIHQI  861
             +LL++S A KGLF+KAI  SG A  + +S          ++   + G    D    +
Sbjct  226   DLLLLSKATKGLFHKAIPESG-ANVAMWSVQVDPIANAKEYAKSEHGFEILDDIYALEDF  284

Query  862   LIELPLEDIMKAN-DVVQYNSGIASFVPVVEIEGHNYTRIIDDDPIVLIAEGRGKDIPLL  1038
                LP +++ K+  +++  N     F P VE +     + +DD P+ ++ +G    +P+L
Sbjct  285   YKSLPYDEMYKSGMELMFKNKNNFLFSPCVERD-TGVEKFLDDTPVNILKKGDYNKVPVL  343

Query  1039  MGFNQNEG  1062
              GF   EG
Sbjct  344   YGFANMEG  351


> ta_transcript84816_1
Length=947

 Score =   174 bits (440),  Expect = 4e-45, Method: Compositional matrix adjust.
 Identities = 110/308 (36%), Positives = 162/308 (53%), Gaps = 16/308 (5%)
 Frame = +1

Query  145   YSFQGIPYGKPPTGARRFAELEPVEPWTDLLYAYEEGPACPSRDIIYGSITIKAKGVSED  324
             ++F  IPY   PTG  RF    P   W     A + G  C   D+   ++ I  K   ED
Sbjct  58    FTFYNIPYATAPTGKDRFKAPLPAPVWITTREAVDRGVVCHQIDL--AAVGIFNKTKQED  115

Query  325   CIYANVFVPATAQLNTDELSEVSLLPILVNIHGGAFNAGSGNRDLHGPELLMVKDVIVIN  504
             C+ ANV+VP T + N         LP++V +HGGA+ AG G   L    ++   +VI +
Sbjct  116   CLIANVYVPDTDKKN---------LPVVVFVHGGAYLAGFGEV-LTPKHMVRTNNVIYVT  165

Query  505   FNYRLAVFGFLSLASDKIPGNNGLRDMVTLLRWVQRNARVFGGDPDKVTVLGESAGAASV  684
             FNYRL V GFL L +   PGN G++DMV LLRWV++N   FGG+PD VT+ G SAG+++V
Sbjct  166   FNYRLGVQGFLCLGTPDAPGNAGMKDMVALLRWVKKNIASFGGNPDDVTIDGYSAGSSAV  225

Query  685   NLLMMSDAAKGLFNKAIIMSGTAFPSFYSTSAVYAKYVADFFLGQLGVNSTDPD-EIHQI  861
             +LL++S A KGLF+KAI  SG A  + +S          ++   + G    D    +
Sbjct  226   DLLLLSKATKGLFHKAIPESG-ANVAMWSVQVDPIANAKEYAKSEHGFEILDDIYALEDF  284

Query  862   LIELPLEDIMKAN-DVVQYNSGIASFVPVVEIEGHNYTRIIDDDPIVLIAEGRGKDIPLL  1038
                LP +++ K+  +++  N     F P VE       + +DD P+ ++ +G    +P+L
Sbjct  285   YKSLPYDEMYKSGMELMFKNKNNFLFSPCVE-RDTGVEKFLDDTPVNILKKGDYNKVPVL  343

Query  1039  MGFNQNEG  1062
              GF   EG
Sbjct  344   YGFANMEG  351


> ta_transcript48393_1
Length=666

 Score =   171 bits (432),  Expect = 8e-45, Method: Compositional matrix adjust.
 Identities = 127/433 (29%), Positives = 202/433 (47%), Gaps = 43/433 (10%)
 Frame = +1

Query  79    LVNIDSGPVCGKVEIAENSTKYYSFQGIPYGKPPTGARRFAELEPVEP--WTDLLYAYEE  252
             +V    GPV G     +     ++F  IPY   P+G  +F    P+ P  WT+   A ++
Sbjct  95    IVETTQGPVRG---YRDPEGGLFAFYSIPYATAPSGPLKFTA--PITPPTWTEPFEAVDK  149

Query  253   GPACPSRDIIYGSITIKAKGVSEDCIYANVFVPATAQLNTDELSEVSLLPILVNIHGGAF  432
                CP       S   K   +  DC+ ANV+VP T +         + LP++V +HGGAF
Sbjct  150   RIICPQMK----SGFTKQMTMQVDCLIANVYVPDTKE---------TKLPVIVYVHGGAF  196

Query  433   NAGSGNRDLHGPE-LLMVKDVIVINFNYRLAVFGFLSLASDKIPGNNGLRDMVTLLRWVQ  609
               G GN  L  P+ L+  K++I + FNYRL   GFL L ++  PGN GL+D V  LRWV
Sbjct  197   QVGFGN--LMTPKNLVRSKNIIAVTFNYRLGAHGFLCLGTEDAPGNAGLKDQVAALRWVN  254

Query  610   RNARVFGGDPDKVTVLGESAGAASVNLLMMSDAAKGLFNKAIIMSGTAFPSFYSTSAVYA  789
             +N    GG+PD VT+ G SAGA SV+L+ +S +A+GLF+K II SG++   F  +
Sbjct  255   KNIIKLGGNPDDVTIAGYSAGAISVDLMAISKSAEGLFSKIIIESGSSL--FAGSIVTDP  312

Query  790   KYVADFFLGQLGVNSTDPDEIHQILIELPLEDIMKANDVVQYNSGIASFVPVVEIEGHNY  969
```

```
                A  F  +LG N+ D     +   +    D +   + +   +    F P VE +
Sbjct  313   LENAKIFAARLGFNNIDDVYALEKFYKAASYDSLTKGEFMDRPNSTLLFSPCVERDDDGL  372

Query  970   TRIIDDDPIVLIAEGRGKDIPLLMGFNQNEGETFR------WLLTIMDALTRYKTNPAAI  1131
                + + PI ++ EG    +P++ GF+  EG  FR      W L + +  + +
Sbjct  373   A-FLSEPPINILKEGSYTQVPMIYGFSNMEG-LFREPFFEFWKLKMNEQFSEF-------  423

Query  1132  LSPRLAYELSTeealakaelvakRYFDGEVTMDGFIKSVT---DQLFQYPIIKLAQWRTL  1302
             +   L +E   +       +    ++D  +  +  +K +    D L +YP ++  +
Sbjct  424   IPGDLHFESKHDRERIAKRIKKFYFWDSPINGENILKYIDYFGDVLVKYPALRTVKMHIQ  483

Query  1303  LNSAPTFLYVFSY  1341
                   +LY +SY
Sbjct  484   NGHDKLYLYEYSY  496


> ta_transcript29751_1
Length=506

 Score =   166 bits (420),  Expect = 6e-44, Method: Compositional matrix adjust.
 Identities = 156/511 (31%), Positives = 240/511 (47%), Gaps = 53/511 (10%)
 Frame = +1

Query  82    VNIDSGPVCGKVEIAENSTKYYSFQGIPYGKPPTGARRFAELEPVEP--WTDLLYAYEEG  255
             V I+ GPV G     +     Y F  IPY   PT   RF    P+ P  W  +  A +
Sbjct  33    VVIEQGPVRG---YKDPDGGLYVFYNIPYATAPTSKDRFKA--PLAPPVWVTIRDAVDRE  87

Query  256   PACPSRDIIYGSITIKAKGVSEDCIYANVFVPATAQLNTDELSEVSLLPILVNIHGGAFN  435
               CP   +  GS     K + EDC+ ANVFVP T++ N         L ++V +HGG +
Sbjct  88    VVCPQM-LETGS----HKNIQEDCLIANVFVPDTSEKN---------LSVVVLVHGGGYL  133

Query  436   AGSGNRDLHGPELLMVKDVIVINFNYRLAVFGFLSLASDKIPGNNGLRDMVTLLRWVQRN  615
             +G GNR     +++  K +I + FNYRL V GFL L +  +PGN G++DMV LLRWV++N
Sbjct  134   SGFGNRQT-PKDMVRDKKLIYVTFNYRLGVHGFLCLGTADVPGNAGMKDMVALLRWVKKN  192

Query  616   ARVFGGDPDKVTVLGESAGAASVNLLMMSDAAKGLFNKAIIMSGTAFPSFYSTSAVYAKY  795
                FGG+PD VT+ G S+G++SV+LL++S A +GLF+KAI  SG+   + +S       +
Sbjct  193   IASFGGNPDDVTIDGHSSGSSSVDLLLLSKATEGLFHKAIPESGSNV-AMWSVQIDPIAH  251

Query  796   VADFFLGQLGVNSTDPD-EIHQILIELPLEDIMKANDVVQYNSGIAS--FVPVVEIEGHN  966
               +F   +    S D    +      LP + + K+   ++YNS + +  F P VE +
Sbjct  252   AKEFARDEHSFESGDDIYALEDFYKTLPFDVMYKSGMSLRYNSSVKNILFQPCVERDT-G  310

Query  967   YTRIIDDDPIVLIAEGRGKDIPLLMGFNQNEGETFRWLLTIMDALTRYKTNPAAI---LS  1137
               + +DD P+ ++  G    +P+L GF   EG     L+ + D    Y++  + I    S
Sbjct  311   VEKFLDDFPVNILKSGDYNKVPMLYGFTNMEG-----LMRVSD-FVNYRSFMSDINEKFS  364

Query  1138  PRLAYELS---TeealakaelvakRYFDGEV----TMDGFIKSVTDQLFQYPIIKLAQWR  1296
               L  +L     EE    A  V + YF  +V    T+  +I   +D +F YP ++  + +
Sbjct  365   DYLPNDLQFRDKEEKEKVARDVKQFYFGDKVINNDTIFEYIDYFSDNMFAYPTLRSLKLQ  424

Query  1297  TLLNSAPTFLYVFSYQGDISIIKRSNWLSYNGTGHVEDLTYVFRTTTFLRDHVSIPPQTR  1476
                 +   +LY FSY  + +       L   G+ H      V  T     D   I   TR
Sbjct  425   VEAGNNQVYLYQFSYPFE-NQAPEGVMLKLEGSSHTAQTHVVLDTLNEENDLAIIRNITR  483

Query  1477  DDHMRDWMSTLFSNYVKCNNPTCTESDDPQW  1569
                        ++N++    P    S+ P W
Sbjct  484   ---------QFWANFITTGTPVPAGSELPAW  505


> ta_transcript82619_1
Length=801

 Score =   160 bits (406),  Expect = 5e-41, Method: Compositional matrix adjust.
 Identities = 167/460 (36%), Positives = 212/460 (46%), Gaps = 32/460 (7%)
 Frame = -2

Query  1355  LMSP*YENTYRKVGAEFSNVLHCASFIIGYWNN*SVTLFMKPSIVTSPSKYLFATNSALA  1176
             L S  Y N+Y   GA     L  A+F  GYW N S   F   S+V +P KY   T+ A +
Sbjct  295   LSSELYANSYIYAGAPPVFAL*SATFSAGYWKNVSEHNFKYSSMVGAPLKYFSETSLARS  354

Query  1175  KASSVDNS*ANLGDNIAAGLVL*RVNASIMVKSHLNVSPSF*LKPISKGISLPRPSAMRT  996
             +A+S       +GDN   G  L     S+     L +S S  + PI  GI LP+P
Sbjct  355   RATSGATLNKTVGDNTNVGSFLILSTMSMSENLFLKLSHSIFVIPIRSGI*LPKPFFSNV  414

Query  995   MGsssiilv*l*psiSTTGTNEAIPELYCTTSLAFMMsskgssiki**issgSVELTPNC  816
               SS           S TG   A+P   C+     M++S+G S     I+SGSV L
```

```
Sbjct  415   *VSSGRTASTPGRGDSMTGKKNAMPLASCSALFTAMIASRGISASCWWIASGSVALMFRA  474

Query  815   PRKKSATYLAYTAEVL*KLGKAVPLIIMALLKSPLAASDIIKRLTDAAPALSPSTVTLSG  636
              R  SA Y A   +V+*  G AVPLI++AL KSP A  D + + T  APAL P+ VT SG
Sbjct  475   LR*LSAAYFAKIGDVV*NKGSAVPLIMIALWKSPCAGGDSVSKCTADAPAL*PARVTSSG  534

Query  635   SPPKTRAFL*TQRSSVTMSRRPLLPGILSEARDKNPKTANL*LKLITITSFTINNSGPCK  456
             SPPK  A   TQRS V MSRRPLLPG L   R++ P T NL*L ++T+T F+   SGPC
Sbjct  535   SPPKLLALSRTQRSKVIMSRRPLLPGTLVVFRERKPNTFNL*LNVMTMTPFSTKCSGPCT  594

Query  455   SRLPDPALKAPPW----------------MFtsmgskltslsssvf--------------  366
             S  P+P  K PP                 MF    ++  S+S+S+
Sbjct  595   SSSPEPNAKPPPCTNTRIGRPLVK*DSSGMFLLYRTRGVSVSTSLTGRPWLVIVVRASVI  654

Query  365   -s*avaGTKTLA*IQSSLTPLALIVMLP*IMSLEGQAGPSS*AYNRSVHGSTGSNSANLL  189
                A+ GT  LA +  SL P+  ++  P*  S  GQ  P   A N     S G +S NL
Sbjct  655   EGRALKGTWMLAKMHDSLMPVG-VIRGP*KTSSCGQTLPGLVASNIPCQDSNGFSSWNLR  713

Query  188   APVGGFPYGIPWKL*YLVLFSAISTFPHTGPESMFTNTPH  69
              P GG   G P K  Y          P T P +    T H
Sbjct  714   GPKGGSA*GTPLKDAYCRPEGVTRRAPQT*PALVRKGTAH  753


> ta_transcript82614_1
Length=867

 Score =   160 bits (405),  Expect = 9e-41, Method: Compositional matrix adjust.
 Identities = 167/460 (36%), Positives = 212/460 (46%), Gaps = 32/460 (7%)
 Frame = -2

Query  1355  LMSP*YENTYRKVGAEFSNVLHCASFIIGYWNN*SVTLFMKPSIVTSPSKYLFATNSALA  1176
             L S  Y N+Y   GA     L  A+F  GYW N S   F   S+V +P KY   T+ A +
Sbjct  361   LSSELYANSYIYAGAPPVFAL*SATFSAGYWKNVSEHNFKYSSMVGAPLKYFSETSLARS  420

Query  1175  KASSVDNS*ANLGDNIAAGLVL*RVNASIMVKSHLNVSPSF*LKPISKGISLPRPSAMRT  996
             +A+S       +GDN   G  L     S+     L +S S  + PI  GI LP+P
Sbjct  421   RATSGATLNKTVGDNTNVGSFLILSTMSMSENLFLKLSHSIFVIPIRSGI*LPKPFFSNV  480

Query  995   MGsssiilv*l*psiSTTGTNEAIPELYCTTSLAFMMsskgssiki**issgSVELTPNC  816
               SS           S TG   A+P   C+     M++S+G S     I+SGSV L
Sbjct  481   *VSSGRTASTPGRGDSMTGKKNAMPLASCSALFTAMIASRGISASCWWIASGSVALMFRA  540

Query  815   PRKKSATYLAYTAEVL*KLGKAVPLIIMALLKSPLAASDIIKRLTDAAPALSPSTVTLSG  636
              R  SA Y A   +V+*  G AVPLI++AL KSP A  D + + T  APAL P+ VT SG
Sbjct  541   LR*LSAAYFAKIGDVV*NKGSAVPLIMIALWKSPCAGGDSVSKCTADAPAL*PARVTSSG  600

Query  635   SPPKTRAFL*TQRSSVTMSRRPLLPGILSEARDKNPKTANL*LKLITITSFTINNSGPCK  456
             SPPK  A   TQRS V MSRRPLLPG L   R++ P T NL*L ++T+T F+   SGPC
Sbjct  601   SPPKLLALSRTQRSKVIMSRRPLLPGTLVVFRERKPNTFNL*LNVMTMTPFSTKCSGPCT  660

Query  455   SRLPDPALKAPPW----------------MFtsmgskltslsssvf--------------  366
             S  P+P  K PP                 MF    ++  S+S+S+
Sbjct  661   SSSPEPNAKPPPCTNTRIGRPLVK*DSSGMFLLYRTRGVSVSTSLTGRPWLVIVVRASVI  720

Query  365   -s*avaGTKTLA*IQSSLTPLALIVMLP*IMSLEGQAGPSS*AYNRSVHGSTGSNSANLL  189
                A+ GT  LA +  SL P+  ++  P*  S  GQ  P   A N     S G +S NL
Sbjct  721   EGRALKGTWMLAKMHDSLMPVG-VIRGP*KTSSCGQTLPGLVASNIPCQDSNGFSSWNLR  779

Query  188   APVGGFPYGIPWKL*YLVLFSAISTFPHTGPESMFTNTPH  69
              P GG   G P K  Y          P T P +    T H
Sbjct  780   GPKGGSA*GTPLKDAYCRPEGVTRRAPQT*PALVRKGTAH  819


> ta_transcript82616_1
Length=859

 Score =   160 bits (404),  Expect = 9e-41, Method: Compositional matrix adjust.
 Identities = 167/460 (36%), Positives = 212/460 (46%), Gaps = 32/460 (7%)
 Frame = -2

Query  1355  LMSP*YENTYRKVGAEFSNVLHCASFIIGYWNN*SVTLFMKPSIVTSPSKYLFATNSALA  1176
             L S  Y N+Y   GA     L  A+F  GYW N S   F   S+V +P KY   T+ A +
Sbjct  353   LSSELYANSYIYAGAPPVFAL*SATFSAGYWKNVSEHNFKYSSMVGAPLKYFSETSLARS  412

Query  1175  KASSVDNS*ANLGDNIAAGLVL*RVNASIMVKSHLNVSPSF*LKPISKGISLPRPSAMRT  996
             +A+S       +GDN   G  L     S+     L +S S  + PI  GI LP+P
Sbjct  413   RATSGATLNKTVGDNTNVGSFLILSTMSMSENLFLKLSHSIFVIPIRSGI*LPKPFFSNV  472
```

```
Query  995   MGsssiilv*l*psiSTTGTNEAIPELYCTTSLAFMMsskgssiki**issgSVELTPNC  816
               SS           S TG   A+P   C+     M++S+G S     I+SGSV L
Sbjct  473   *VSSGRTASTPGRGDSMTGKKNAMPLASCSALFTAMIASRGISASCWWIASGSVALMFRA  532

Query  815   PRKKSATYLAYTAEVL*KLGKAVPLIIMALLKSPLAASDIIKRLTDAAPALSPSTVTLSG  636
              R  SA Y A   +V+*  G AVPLI++AL KSP A  D + + T  APAL P+ VT SG
Sbjct  533   LR*LSAAYFAKIGDVV*NKGSAVPLIMIALWKSPCAGGDSVSKCTADAPAL*PARVTSSG  592

Query  635   SPPKTRAFL*TQRSSVTMSRRPLLPGILSEARDKNPKTANL*LKLITITSFTINNSGPCK  456
             SPPK  A   TQRS V MSRRPLLPG L   R++ P T NL*L ++T+T F+   SGPC
Sbjct  593   SPPKLLALSRTQRSKVIMSRRPLLPGTLVVFRERKPNTFNL*LNVMTMTPFSTKCSGPCT  652

Query  455   SRLPDPALKAPPW----------------MFtsmgskltslsssvf--------------  366
             S  P+P  K PP                 MF    ++  S+S+S+
Sbjct  653   SSSPEPNAKPPPCTNTRIGRPLVK*DSSGMFLLYRTRGVSVSTSLTGRPWLVIVVRASVI  712

Query  365   -s*avaGTKTLA*IQSSLTPLALIVMLP*IMSLEGQAGPSS*AYNRSVHGSTGSNSANLL  189
                A+ GT  LA +  SL P+  ++  P*  S  GQ  P   A N     S G +S NL
Sbjct  713   EGRALKGTWMLAKMHDSLMPVG-VIRGP*KTSSCGQTLPGLVASNIPCQDSNGFSSWNLR  771

Query  188   APVGGFPYGIPWKL*YLVLFSAISTFPHTGPESMFTNTPH  69
              P GG   G P K  Y          P T P  +   T H
Sbjct  772   GPKGGSA*GTPLKDAYCRPEGVTRRAPQT*PALVRKGTAH  811


> ta_transcript82623_1
Length=454

 Score =   135 bits (340),  Expect = 8e-34, Method: Compositional matrix adjust.
 Identities = 147/406 (36%), Positives = 187/406 (46%), Gaps = 32/406 (8%)
 Frame = -2

Query  1193  TNSALAKASSVDNS*ANLGDNIAAGLVL*RVNASIMVKSHLNVSPSF*LKPISKGISLPR  1014
             T+ A +KA+S       +GDN   G  L     S+     L +S S  + PI  GI LP+
Sbjct  2     TSFARSKATSGATLNKTVGDNTNVGSFLILSTMSMSENLFLKLSHSIFVIPIRSGI*LPK  61

Query  1013  PSAMRTMGsssiilv*l*psiSTTGTNEAIPELYCTTSLAFMMsskgssiki**issgSV  834
             P       SS           S TG   A+P   C+     M++S+G S     I+SGSV
Sbjct  62    PFFSNV*VSSGRTASTPGRGDSMTGKKNAMPLASCSALFTAMIASRGISASCWWIASGSV  121

Query  833   ELTPNCPRKKSATYLAYTAEVL*KLGKAVPLIIMALLKSPLAASDIIKRLTDAAPALSPS  654
              L     R  SA Y A   +V+*  G AVPLI++AL KSP A  D + + T  APAL P+
Sbjct  122   ALMFRALR*LSAAYFAKIGDVV*NKGSAVPLIMIALWKSPCAGGDSVSKCTADAPAL*PA  181

Query  653   TVTLSGSPPKTRAFL*TQRSSVTMSRRPLLPGILSEARDKNPKTANL*LKLITITSFTIN  474
              VT SGSPPK  A   TQRS V MSRRPLLPG L   R++ P T NL*L ++T+T F+
Sbjct  182   RVTSSGSPPKLLALSRTQRSKVIMSRRPLLPGTLVVFRERKPNTFNL*LNVMTMTPFSTK  241

Query  473   NSGPCKSRLPDPALKAPPW----------------MFtsmgskltslsss----------  372
              SGPC S  P+P  K PP                 MF    ++  S+S+S
Sbjct  242   CSGPCTSSSPEPNAKPPPCTNTRIGRPLVK*DSSGMFLLYRTRGVSVSTSLTGRPWLVIV  301

Query  371   -----vfs*avaGTKTLA*IQSSLTPLALIVMLP*IMSLEGQAGPSS*AYNRSVHGSTGS  207
                  +   A+ GT  LA +  SL P+  ++  P*  S  GQ  P   A N     S G
Sbjct  302   VRASVIEGRALKGTWMLAKMHDSLMPVG-VIRGP*KTSSCGQTLPGLVASNIPCQDSNGF  360

Query  206   NSANLLAPVGGFPYGIPWKL*YLVLFSAISTFPHTGPESMFTNTPH  69
             +S NL  P GG   G P K  Y          P T P  +   T H
Sbjct  361   SSWNLRGPKGGSA*GTPLKDAYCRPEGVTRRAPQT*PALVRKGTAH  406


> ta_transcript49309_1
Length=664

 Score =   126 bits (316),  Expect = 8e-30, Method: Compositional matrix adjust.
 Identities = 78/222 (35%), Positives = 111/222 (50%), Gaps = 31/222 (14%)
 Frame = +1

Query  148  SFQGIPYGKPPTGARRFAELEPVEP--WTDLLYAYEEGPAC----PSRDIIYGSI-----  294
            ++ GIPY +PP    RF   E + P  W  +  A    P C    P +D +  ++
Sbjct  52   AYVGIPYAQPPIDRLRFMPPEYLNPPQWEGVYNATIFAPDCMQSDPKKDDVQSTLKKHDE  111

Query  295  -----------TIKAKGVSEDCIYANVFVPATAQLNTDELSEVSLLPILVNIHGGAFNAG  441
                       T + K  SEDC+Y NV+VP   +        V   P++V  HGG F  G
Sbjct  112  LFMKLLDSQLETPREKNYSEDCLYLNVYVPDDYK--------VEGYPVMVWFHGGEFVRG  163
```

```
Query  442   SGNRDLHGPELLMVKDVIVINFNYRLAVFGFLSLASDKIPGNNGLRDMVTLLRWVQRNAR  621
             S N  ++   L++ + VI ++  YRL +FGF +    +  GN GL D V  L WV+ N
Sbjct  164   SPNY-MNPFHLVLKQKVIFVSVAYRLNIFGFFTTLDHEALGNFGLHDQVAALSWVKTNIE  222

Query  622   VFGGDPDKVTVLGESAGAASVNLLMMSDAAKGLFNKAIIMSG  747
              FGGDP+ + + G  AGA SV L ++S   + GLF+KAI MSG
Sbjct  223   HFGGDPENICIFGHDAGAVSVGLHLVSTYSPGLFHKAIAMSG  264
```

****
****

Query= gi|512885949|ref|XM_004921624.1| PREDICTED: Bombyx mori juvenile hormone esterase-like (LOC101739057), mRNA

```
Length=1605
                                                                      Score     E
Sequences producing significant alignments:                          (Bits)  Value

  ta_transcript48580_1                                                  238   5e-68
  ta_transcript48579_1                                                  238   1e-67
  ta_transcript75783_1                                                  214   1e-59
  ta_transcript58915_1                                                  205   4e-57
  ta_transcript58914_1                                                  204   8e-57
  ta_transcript48393_1                                                  197   3e-54
  ta_transcript29751_1                                                  180   7e-49
  ta_transcript84821_1                                                  157   1e-40
  ta_transcript84818_1                                                  157   3e-40
  ta_transcript84816_1                                                  155   2e-39
  ta_transcript15565_1                                                  123   6e-30


> ta_transcript48580_1
Length=834

 Score =   238 bits (607),  Expect = 5e-68, Method: Compositional matrix adjust.
 Identities = 173/546 (32%), Positives = 266/546 (49%), Gaps = 46/546 (8%)
 Frame = +1

Query  64    PLVDTQRGLIRGLR-----SENGKFAKFLGIPYALVDENN-PFGPSVPHPGFEETFEAYD  225
             P+V T+ G +RG R     +     +  F GI YA     +  F P VP   +    +A +
Sbjct  154   PIVMTELGALRGRRVVARTTAQTPYFSFKGIRYAQPPRGSLRFKPPVPLEPWSGVRDALE  213

Query  226   DSVVCPQ--VTKGVGVGSLQCLNLNVY---VPNTATSRN-KRPVMIWIHGGSFATGSGTG  387
             +  VCP   +       G   CL LNVY   +P   T  N K  VM+WIHGG+FA GSG
Sbjct  214   EGAVCPHRFMLFDTYKGDEDCLFLNVYTPALPEKITGFNPKLAVMVWIHGGAFAVGSGNA  273

Query  388   RDFSYDDLVRHDVIVVSVNYRLGPYGFLCLDSPDIPGNQGLKDQALALRWIKENIEAFGG  567
               +   D L+   ++++V++NYRLG  GFL +++ ++ GN GLKDQ LAL+W+++NIE FGG
Sbjct  274   FLYGPDHLMGGNIVLVTLNYRLGALGFLSMENDEVSGNMGLKDQVLALKWVRDNIEYFGG  333

Query  568   DVSKITLFGESAGGVAVDLHLLTDKDE-LFNQVIIQSGSAFFAGGI-RKPNNRVPIEIAS  741
             D S++T+FGESAG  +V LH+L+    + LF++ I QSG A     + R P  R   E+
Sbjct  334   DASRVTIFGESAGAASVHLHMLSPASKGLFHRAIAQSGLALSPWALTRSPRERA-FELGR  392

Query  742   QLGFETDNFAEAIKFLVDQDPHLVVAASTSQRSTLGGNT---------LRPCLENKYDGV  894
             +LG +T++ AE + +L      L+V A        G +             P +E   D
Sbjct  393   ELGIDTNSTAELLGYLRATPSELLVKAGARLAGAPGKSVDLHSTVALPFVPTVEP--DVP  450

Query  895   DSFLSDFPENLR-ARNIPAIYGVNNKEFLTLHAFVTPEDYEVTGIRAFLERAFN------  1053
             D+FL+  P NL    ++P + G N +E + L   F   + Y    +   L+R F
Sbjct  451   DAFLTQHPRNLMPGADVPLLTGYNAQEGIIL--FRRLQRYP--KLLTELDREFRRVVPPE  506

Query  1054  -LTDSEME-----DHVRHFYIGDETLTEKFFDEFIDFASDYYYNYGVQRSIK-KSLADGN  1212
              LT  E +     DH+R FY      +  +  D  ID  +D  +   +   +++ +   +
Sbjct  507   LLTGDEAQNRRVADHIRAFYFQQRPVDIRNIDSLIDLFTDVMFLRPLLETLRIQGKTNRT  566

Query  1213  KEVYYYVFSYDGGRNAMKQYLGITAEGAAHADELGYLLAVDAVPGQHIAEEDQLIIDR-I  1389
                Y Y F++DG     K+ LGI    GA H DE+GYL     +  +   +   +L + + +
Sbjct  567   SPTYLYRFAFDGALGLFKRMLGINHPGACHGDEMGYLFYFSRLNYRLDDDSTELAVSKGM  626

Query  1390  TTLWANFAKYGYQPTPEPTDLLPVVWTTV-EGNKRPYLDIDTDLQLRSRPFHHRMAFWDL  1566
               +W NFAK G      +  D +   W+ V +     YLDI+     +   P   R+  WD
Sbjct  627   VRMWTNFAKTGNPTIGDHDDPIDFKWSPVNDTTHLTYLDINGHFTQKQDPEARRVRLWDW  686

Query  1567  FYKLYG  1584
              Y+ Y
Sbjct  687   LYENYA  692
```

```
> ta_transcript48579_1
Length=905

 Score =   238 bits (606),  Expect = 1e-67, Method: Compositional matrix adjust.
 Identities = 173/546 (32%), Positives = 266/546 (49%), Gaps = 46/546 (8%)
 Frame = +1

Query  64    PLVDTQRGLIRGLR-----SENGKFAKFLGIPYALVDENN-PFGPSVPHPGFEETFEAYD  225
             P+V T+ G +RG R     +    +  F GI YA     +  F P VP   +    +A +
Sbjct  154   PIVMTELGALRGRRVVARTTAQTPYFSFKGIRYAQPPRGSLRFKPPVPLEPWSGVRDALE  213

Query  226   DSVVCPQ--VTKGVGVGSLQCLNLNVY---VPNTATSRN-KRPVMIWIHGGSFATGSGTG  387
             +  VCP   +      G   CL LNVY   +P   T  N K  VM+WIHGG+FA GSG
Sbjct  214   EGAVCPHRFMLFDTYKGDEDCLFLNVYTPALPEKITGFNPKLAVMVWIHGGAFAVGSGNA  273

Query  388   RDFSYDDLVRHDVIVVSVNYRLGPYGFLCLDSPDIPGNQGLKDQALALRWIKENIEAFGG  567
               +  D L+  ++++V++NYRLG  GFL +++ ++ GN GLKDQ LAL+W+++NIE FGG
Sbjct  274   FLYGPDHLMGGNIVLVTLNYRLGALGFLSMENDEVSGNMGLKDQVLALKWVRDNIEYFGG  333

Query  568   DVSKITLFGESAGGVAVDLHLLTDKDE-LFNQVIIQSGSAFFAGGI-RKPNNRVPIEIAS  741
             D S++T+FGESAG  +V LH+L+   + LF++ I QSG A     + R P  R   E+
Sbjct  334   DASRVTIFGESAGAASVHLHMLSPASKGLFHRAIAQSGLALSPWALTRSPRERA-FELGR  392

Query  742   QLGFETDNFAEAIKFLVDQDPHLVVAASTSQRSTLGGNT---------LRPCLENKYDGV  894
             +LG +T++ AE + +L      L+V A        G +            P +E   D
Sbjct  393   ELGIDTNSTAELLGYLRATPSELLVKAGARLAGAPGKSVDLHSTVALPFVPTVEP--DVP  450

Query  895   DSFLSDFPENLR-ARNIPAIYGVNNKEFLTLHAFVTPEDYEVTGIRAFLERAFN------  1053
             D+FL+  P NL    ++P + G N +E + L  F   + Y    +   L+R F
Sbjct  451   DAFLTQHPRNLMPGADVPLLTGYNAQEGIIL--FRRLQRYP--KLLTELDREFRRVVPPE  506

Query  1054  -LTDSEME-----DHVRHFYIGDETLTEKFFDEFIDFASDYYYNYGVQRSIK-KSLADGN  1212
              LT  E +     DH+R FY     +  +  D  ID  +D  +   +  +++ +   +
Sbjct  507   LLTGDEAQNRRVADHIRAFYFQQRPVDIRNIDSLIDLFTDVMFLRPLLETLRIQGKTNRT  566

Query  1213  KEVYYYVFSYDGGRNAMKQYLGITAEGAAHADELGYLLAVDAVPGQHIAEEDQLIIDR-I  1389
                Y Y F++DG     K+ LGI   GA H DE+GYL     +  +   +  +L + + +
Sbjct  567   SPTYLYRFAFDGALGLFKRMLGINHPGACHGDEMGYLFYFSRLNYRLDDDSTELAVSKGM  626

Query  1390  TTLWANFAKYGYQPTPEPTDLLPVVWTTV-EGNKRPYLDIDTDLQLRSRPFHHRMAFWDL  1566
               +W NFAK G     +  D +   W+ V +     YLDI+     +  P   R+  WD
Sbjct  627   VRMWTNFAKTGNPTIGDHDDPIDFKWSPVNDTTHLTYLDINGHFTQKQDPEARRVRLWDW  686

Query  1567  FYKLYG  1584
              Y+ Y
Sbjct  687   LYENYA  692


> ta_transcript75783_1
Length=733

 Score =   214 bits (544),  Expect = 1e-59, Method: Compositional matrix adjust.
 Identities = 173/546 (32%), Positives = 251/546 (46%), Gaps = 53/546 (10%)
 Frame = +1

Query  58    IDPLVDTQRGLIRGLRSEN---GKFAKFLGIPYALVDENNPFG--------PSVPHPGFE  204
             +D  V    G+++G   +N   G +  F GIP+A      P G        P+ P  G +
Sbjct  122   LDNTVKVSEGILQGELVQNEFGGSYYSFKGIPFA----EPPVGDLRFKAPIPAKPWKGVK  177

Query  205   ETFEAYDDSVVCPQVTKGVGVGSLQCLNLNVYVPNTATSRNKRPVMIWIHGGSFATGSGT  384
                +    +       K    GS  CL LNVY P         PVM+WIHGG +  GSG
Sbjct  178   TATKFGPINYQFDVFKKDPPSGSEDCLYLNVYTPEIKPI-TLLPVMVWIHGGGYFWGSGN  236

Query  385   GRDFSYDDLVRHDVIVVSVNYRLGPYGFLCLDSPDIPGNQGLKDQALALRWIKENIEAFG  564
                +  + LVRH VI+V+ NYRLG  GFL LD+ ++PGN G+KDQ  AL+W+K NI  FG
Sbjct  237   DDVYGPEFLVRHGVILVTFNYRLGVLGFLSLDTAEVPGNAGMKDQVAALKWVKSNIHFFG  296

Query  565   GDVSKITLFGESAGGVAVDLHLLTDKDE-LFNQVIIQSGSAFFAGGIRKPNNRVPIEIAS  741
             GD   IT+FGESAG  AV LHLL+   + LF + IIQSGSA              I +A
Sbjct  297   GDPENITIFGESAGAGAVSLHLLSPMSKGLFKRAIIQSGSATAYWAQAFEIKEKSIALAR  356

Query  742   QLGFETDNFAEAIKFLVDQDPHLVVAAS---TSQRSTLGGNTLRPCLENKYDGVDSFLSD  912
             +LG  TD+  +  +F  +    L++  S   T            P +E  + G + FL+D
Sbjct  357   KLGLVTDDDQKLYEFFGNISKELLINISVPVTFAEQEEVNIEFAPTIEKDF-GQERFLTD  415
```

```
Query  913   FPENLRARNI----PAIYGVNNKEFLTLHAFVTPEDYEVTGIRAFLERAFNLTDSEMEDH  1080
                 +  + I      + G    E +     +    D E+  I  FLE     L    M+
Sbjct  416   DSYKILQQGIHKGVDVMMGYVEDEGVLYLNSLEILDKEIERINNFLEA---LVPRNMKRE  472

Query  1081  V------------RHFYIGDETLTEKFFDEFIDFASDYYYNYGVQRSIKKSLADGNKEVY  1224
             +            + FY  ++ +T    +     + S    ++Y V +S K      N   +
Sbjct  473   IPIKQQLKVGRKMKKFYFDNKLVTRNDWRTIARYISLNVFSYDVIQSQKNIAKSQNS--F  530

Query  1225  YYVFSYDGGRNAMKQYLG---IT--AEGAAHADELGYLLAVDAVPGQHIAEEDQ--LIID  1383
             +Y F+    RN  K+      IT    +    H D+L YL  V  +  Q +A++ +   +ID
Sbjct  531   FYKFTCKSKRNFFKKVFTDDEITKDVDVVCHCDDLAYLFPVKFM-NQSVAKDSREFQLID  589

Query  1384  RITTLWANFAKYGYQPTPEPTDLLPVVWTTVEGNKRPYLDIDTDLQLRSRPFHHRMAFWD  1563
             ++T LW NFAKYG    P P + L V W       + YLDI  DL L+++P    + FW+
Sbjct  590   KVTKLWTNFAKYG---NPTPDNSLGVKWEPFTLQHQHYLDIGEDLVLKTKPDDEDIQFWE  646

Query  1564  LFYKLY  1581
               Y+ Y
Sbjct  647   ELYREY  652


> ta_transcript58915_1
Length=606

 Score =   205 bits (521),  Expect = 4e-57, Method: Compositional matrix adjust.
 Identities = 163/536 (30%), Positives = 261/536 (49%), Gaps = 43/536 (8%)
 Frame = +1

Query  70    VDTQRGLIRGLR----SENGKFAKFLGIPYALVDENN-PFGPSVPHPGFEETFEAYDDSV  234
             V  ++G + G +    S +G+F  F GIPYA         F    P   ++    +A
Sbjct  32    VQVEQGWLEGEQLDSVSGDGQFFSFKGIPYAAPPVGKLRFKAPQPPVSWQGVRKATQHGP  91

Query  235   VCPQ---VTKGVGVGSLQCLNLNVYVPNTATSRNKRPVMIWIHGGSFATGSGTGRDFSYD  405
              CPQ    +K +  GS  CL LNVY  +   + ++  PVM++IHGG F +GSG    +  D
Sbjct  92    KCPQRDIFSKQIIPGSEDCLYLNVYTKDI-SPKSPLPVMVFIHGGGFKSGSGDEDFYGGD  150

Query  406   DLVRHDVIVVSVNYRLGPYGFLCLDSPDIPGNQGLKDQALALRWIKENIEAFGGDVSKIT  585
              LV H V++V++NYRL   GFLCLD+ ++PGN GLKDQ  AL+W+++NI AFGGD + +T
Sbjct  151   FLVHHGVVLVTINYRLDALGFLCLDTEEVPGNAGLKDQVAALKWVQKNIFAFGGDPTNVT  210

Query  586   LFGESAGGVAVDLHLLTDKDE-LFNQVIIQSGSAFFAGGIRKPNNRVPIEIASQLGFETD  762
             +FGESAGG +  LH+L+    + LF + I  SG  F    I     +    +   LGFET+
Sbjct  211   IFGESAGGSSTALHVLSPLSKGLFKRAIPMSGVPFCDWSIPFEPRKRAFTLGKILGFETE  270

Query  763   NFAEAIKFL--------VDQDPHLVVAASTSQRSTLGGNTLRPCLENKYDGVDSFLSDFP  918
             +    +++L         VD +P  ++        + L      P +E  + G   F+++ P
Sbjct  271   DPKALLEYLQSLPAEKFVDTNP-TIMGFEEKSYNMLKMYHFTPVVEKNF-GQHHFMTEEP  328

Query  919   -ENLR---ARNIPAIYGVNNKEFLT-LHAFVTPEDY-----EVTGIRAFLERAFNLTDSE  1068
              E L+     ++  + G  ++E +  + +FV          EV   R  L ++      E
Sbjct  329   LEALKQGHVNDVDVLIGNTDQETIVGIPSFVDLLKMYDRYPEVFVPRKILNKSTPGKILE  388

Query  1069  MEDHVRHFYIGDETLTEKFFDEFIDFASDYYYNYGVQRSIKKSLAD--GNKEVYYYVFSY  1242
             + + +R  Y GD+ + +    E + + S+  + Y V +     L+  G+ +VY Y FS
Sbjct  389   IAERIRKHYFGDKPIDKTSMKEAVTYFSEVCFTYDVNKYTNLLLSGKPGSSKVYRYRFSC  448

Query  1243  DGGRNAMKQY---LGITAEGAAHADELGYLLAVDAV--PGQHIAEEDQLIIDRITTLWAN  1407
                RN   +Y    GIT  GA+H ++L YL    +V  P   ++  ++ I +   L+ N
Sbjct  449   VSERNIFGKYGQEYGIT--GASHLEDLMYLFDAKSVKLPLDRNSKSYKM-IQQTCALFTN  505

Query  1408  FAKYGYQPTPEPTDLLPVVWTTVEGNKRPYLDIDTDLQLRSRPFHHRMAFWDLFYK  1575
             FAKYG    P P   L    W  +    + ++DI    L  +          + FW+  Y+
Sbjct  506   FAKYG---NPTPDSSLGFTWPEYDIKDQSFVDIADQLTVGRHLDADAVKFWESIYQ  558


> ta_transcript58914_1
Length=650

 Score =   204 bits (520),  Expect = 8e-57, Method: Compositional matrix adjust.
 Identities = 163/536 (30%), Positives = 261/536 (49%), Gaps = 43/536 (8%)
 Frame = +1

Query  70    VDTQRGLIRGLR----SENGKFAKFLGIPYALVDENN-PFGPSVPHPGFEETFEAYDDSV  234
             V  ++G + G +    S +G+F  F GIPYA         F    P   ++    +A
Sbjct  32    VQVEQGWLEGEQLDSVSGDGQFFSFKGIPYAAPPVGKLRFKAPQPPVSWQGVRKATQHGP  91

Query  235   VCPQ---VTKGVGVGSLQCLNLNVYVPNTATSRNKRPVMIWIHGGSFATGSGTGRDFSYD  405
```

```
             CPQ    +K +  GS  CL LNVY  + +  ++  PVM++IHGG F +GSG    +  D
Sbjct  92    KCPQRDIFSKQIIPGSEDCLYLNVYTKDIS-PKSPLPVMVFIHGGGFKSGSGDEDFYGGD  150

Query  406   DLVRHDVIVVSVNYRLGPYGFLCLDSPDIPGNQGLKDQALALRWIKENIEAFGGDVSKIT  585
              LV H V++V++NYRL   GFLCLD+ ++PGN GLKDQ  AL+W+++NI AFGGD + +T
Sbjct  151   FLVHHGVVLVTINYRLDALGFLCLDTEEVPGNAGLKDQVAALKWVQKNIFAFGGDPTNVT  210

Query  586   LFGESAGGVAVDLHLLTDKDE-LFNQVIIQSGSAFFAGGIRKPNNRVPIEIASQLGFETD  762
             +FGESAGG +  LH+L+   + LF + I  SG  F    I     +    +   LGFET+
Sbjct  211   IFGESAGGSSTALHVLSPLSKGLFKRAIPMSGVPFCDWSIPFEPRKRAFTLGKILGFETE  270

Query  763   NFAEAIKFL--------VDQDPHLVVAASTSQRSTLGGNTLRPCLENKYDGVDSFLSDFP  918
             +    +++L        VD +P  ++       + L      P +E  + G   F+++ P
Sbjct  271   DPKALLEYLQSLPAEKFVDTNP-TIMGFEEKSYNMLKMYHFTPVVEKNF-GQHHFMTEEP  328

Query  919   -ENLR---ARNIPAIYGVNNKEFLT-LHAFVTPEDY-----EVTGIRAFLERAFNLTDSE  1068
              E L+     ++  + G  ++E +  + +FV           EV   R  L ++      E
Sbjct  329   LEALKQGHVNDVDVLIGNTDQETIVGIPSFVDLLKMYDRYPEVFVPRKILNKSTPGKILE  388

Query  1069  MEDHVRHFYIGDETLTEKFFDEFIDFASDYYYNYGVQRSIKKSLAD--GNKEVYYYVFSY  1242
             + + +R  Y GD+ + +    E + + S+  + Y V +     L+   G+ +VY Y FS
Sbjct  389   IAERIRKHYFGDKPIDKTSMKEAVTYFSEVCFTYDVNKYTNLLLSGKPGSSKVYRYRFSC  448

Query  1243  DGGRNAMKQY---LGITAEGAAHADELGYLLAVDAV--PGQHIAEEDQLIIDRITTLWAN  1407
                RN   +Y    GIT  GA+H ++L YL    +V  P    ++  ++ I +   L+ N
Sbjct  449   VSERNIFGKYGQEYGIT--GASHLEDLMYLFDAKSVKLPLDRNSKSYKM-IQQTCALFTN  505

Query  1408  FAKYGYQPTPEPTDLLPVVWTTVEGNKRPYLDIDTDLQLRSRPFHHRMAFWDLFYK  1575
             FAKYG    P P   L   W   +   + ++DI   L +         + FW+  Y+
Sbjct  506   FAKYG---NPTPDSSLGFTWPEYDIKDQSFVDIADQLTVGRHLDADAVKFWESIYQ  558


> ta_transcript48393_1
Length=666

 Score =   197 bits (501),  Expect = 3e-54, Method: Compositional matrix adjust.
 Identities = 145/419 (35%), Positives = 212/419 (51%), Gaps = 44/419 (11%)
 Frame = +1

Query  67    LVDTQRGLIRGLRSENGKFAKFLGIPYALVDENNPFGP-----SVPHPGFEETFEAYDDS  231
             +V+T +G +RG R   G    F  IPYA      P GP      +  P + E FEA D
Sbjct  95    IVETTQGPVRGYRDPEGGLFAFYSIPYATA----PSGPLKFTAPITPPTWTEPFEAVDKR  150

Query  232   VVCPQVTKGVG---VGSLQCLNLNVYVPNTATSRNKRPVMIWIHGGSFATGSGTGRDFSY  402
             ++CPQ+  G         + CL  NVYVP+T   +  K PV++++HGG+F  G G     +
Sbjct  151   IICPQMKSGFTKQMTMQVDCLIANVYVPDTKET--KLPVIVYVHGGAFQVGFGN--LMTP  206

Query  403   DDLVR-HDVIVVSVNYRLGPYGFLCLDSPDIPGNQGLKDQALALRWIKENIEAFGGDVSK  579
              +LVR  ++I V+ NYRLG +GFLCL + D PGN GLKDQ  ALRW+ +NI   GG+
Sbjct  207   KNLVRSKNIIAVTFNYRLGAHGFLCLGTEDAPGNAGLKDQVAALRWVNKNIIKLGGNPDD  266

Query  580   ITLFGESAGGVAVDLHLLTDKDE-LFNQVIIQSGSAFFAGGI-RKPNNRVPIEIASQLGF  753
             +T+ G SAG ++VDL  ++   E LF+++II+SGS+ FAG I   P     I  A++LGF
Sbjct  267   VTIAGYSAGAISVDLMAISKSAEGLFSKIIIESGSSLFAGSIVTDPLENAKI-FAARLGF  325

Query  754   ET-DNFAEAIKFLVDQDPHLVVAASTSQR--STLGGNTLRPCLENKYDGVDSFLSDFPEN  924
                D+     KF        +       R  STL       PC+E   DG+ +FLS+ P N
Sbjct  326   NNIDDVYALEKFYKAASYDSLTKGEFMDRPNSTL---LFSPCVERDDDGL-AFLSEPPIN  381

Query  925   LRAR----NIPAIYGVNNKEFLTLHAFVTPEDYEVTGIRAFLERAFNLTDSEMED-----  1077
             +         +P IYG +N E L    F   E +++     F E  F   D   E
Sbjct  382   ILKEGSYTQVPMIYGFSNMEGLFREPFF--EFWKLKMNEQFSE--FIPGDLHFESKHDRE  437

Query  1078  ----HVRHFYIGDETLTEKFFDEFIDFASDYYYNYGVQRSIKKSLADGNKEVYYYVFSY  1242
                  ++ FY  D  +  +   ++ID+  D    Y   R++K  + +G+ ++Y Y +SY
Sbjct  438   RIAKRIKKFYFWDSPINGENILKYIDYFGDVLVKYPALRTVKMHIQNGHDKLYLYEYSY  496


> ta_transcript29751_1
Length=506

 Score =   180 bits (456),  Expect = 7e-49, Method: Compositional matrix adjust.
 Identities = 153/491 (31%), Positives = 241/491 (49%), Gaps = 51/491 (10%)
 Frame = +1

Query  70    VDTQRGLIRGLRSENGKFAKFLGIPYALV-DENNPFGPSVPHPGFEETFEAYDDSVVCPQ  246
             V  ++G +RG +  +G    F  IPYA      + F   +  P +    +A D  VVCPQ
```

```
Sbjct  33    VVIEQGPVRGYKDPDGGLYVFYNIPYATAPTSKDRFKAPLAPPVWVTIRDAVDREVVCPQ  92

Query  247   VTKGVGVGSLQ--CLNLNVYVPNTATSRNKRPVMIWIHGGSFATGSGTGRDFSYDDLVR-  417
             + +     ++Q  CL  NV+VP+T  S     V++ +HGG + +G G  +  +  D+VR
Sbjct  93    MLETGSHKNIQEDCLIANVFVPDT--SEKNLSVVVLVHGGGYLSGFGNRQ--TPKDMVRD  148

Query  418   HDVIVVSVNYRLGPYGFLCLDSPDIPGNQGLKDQALALRWIKENIEAFGGDVSKITLFGE  597
               +I V+ NYRLG +GFLCL + D+PGN G+KD    LRW+K+NI +FGG+   +T+ G
Sbjct  149   KKLIYVTFNYRLGVHGFLCLGTADVPGNAGMKDMVALLRWVKKNIASFGGNPDDVTIDGH  208

Query  598   SAGGVAVDLHLLTDKDE-LFNQVIIQSGSAFFAGGIRKPNNRVPIEIASQLG-----FET  759
             S+G  +VDL LL+   E LF++ I +SGS      ++      PI  A +       FE+
Sbjct  209   SSGSSSVDLLLLSKATEGLFHKAIPESGSNVAMWSVQID----PIAHAKEFARDEHSFES  264

Query  760   DNFAEAIKFLVDQDPHLVV---AASTSQRSTLGGNTLRPCLENKYDGVDSFLSDFPENLR  930
              +   A++      P  V+     S    S++     +PC+E +  GV+ FL DFP N+
Sbjct  265   GDDIYALEDFYKTLPFDVMYKSGMSLRYNSSVKNILFQPCVE-RDTGVEKFLDDFPVNIL  323

Query  931   A----RNIPAIYGVNNKE-------FLTLHAFVTPEDYEVTGIRAFLERAFNLTDSEMED  1077
                    +P +YG  N E       F+   +F++  + + +    +L       D E ++
Sbjct  324   KSGDYNKVPMLYGFTNMEGLMRVSDFVNYRSFMSDINEKFSD---YLPNDLQFRDKEEKE  380

Query  1078  H----VRHFYIGDETLTEKFFDEFIDFASDYYYNYGVQRSIKKSLADGNKEVYYYVFSYD  1245
                  V+ FY GD+ +      E+ID+ SD  + Y   RS+K  +  GN +VY Y FSY
Sbjct  381   KVARDVKQFYFGDKVINNDTIFEYIDYFSDNMFAYPTLRSLKLQVEAGNNQVYLYQFSYP  440

Query  1246  GGRNAMKQYLGITAEGAAHADELGYLLAVDAVPGQHIAEEDQLIIDRITT--LWANFAKY  1419
                N   + + +  EG++H  +   +L         + EE+ L I R  T   WANF
Sbjct  441   -FENQAPEGVMLKLEGSSHTAQTHVVL-------DTLNEENDLAIIRNITRQFWANFITT  492

Query  1420  GYQPTPEPTDL  1452
             G  P P  ++L
Sbjct  493   G-TPVPAGSEL  502


> ta_transcript84821_1
Length=611

 Score =   157 bits (396),  Expect = 1e-40, Method: Compositional matrix adjust.
 Identities = 130/387 (34%), Positives = 192/387 (50%), Gaps = 29/387 (7%)
 Frame = +1

Query  70    VDTQRGLIRGLRSENGKFAKFLGIPYALVDE-NNPFGPSVPHPGFEETFEAYDDSVVCPQ  246
             V+ Q+G +RG +    G    F  IPYA      + F   +P P +  T EA D  VVC Q
Sbjct  40    VEIQQGKLRGYKDPEGGLFTFYNIPYATAPTGKDRFKAPLPAPVWITTREAVDRGVVCHQ  99

Query  247   V-TKGVGV----GSLQCLNLNVYVPNTATSRNKRPVMIWIHGGSFATGSGTGRDFSYDDL  411
             +    VG+        CL  NVYVP+T   +   PV++++HGG++   +G G   +   +
Sbjct  100   IDLAAVGIFNKTKQEDCLIANVYVPDT--DKKNLPVVVFVHGGAYL--AGFGEVLTPKHM  155

Query  412   VR-HDVIVVSVNYRLGPYGFLCLDSPDIPGNQGLKDQALALRWIKENIEAFGGDVSKITL  588
             VR ++VI V+ NYRLG  GFLCL +PD PGN G+KD    LRW+K+NI +FGG+   +T+
Sbjct  156   VRTNNVIYVTFNYRLGVQGFLCLGTPDAPGNAGMKDMVALLRWVKKNIASFGGNPDDVTI  215

Query  589   FGESAGGVAVDLHLLTDKDE-LFNQVIIQSGSAFFAGGIR-KPNNRVPIEIASQLGFETD  762
              G SAG  AVDL LL+   + LF++ I +SG+      ++  P         S+ GFE
Sbjct  216   DGYSAGSSAVDLLLLSKATKGLFHKAIPESGANVAMWSVQVDPIANAKEYAKSEHGFEIL  275

Query  763   NFAEAIKFLVDQDPHLVVAASTSQRSTLGGNT--LRPCLENKYDGVDSFLSDFPENLRAR  936
             +   A++      P+  +  S  +      N     PC+E +  GV+ FL D P N+  +
Sbjct  276   DDIYALEDFYKSLPYDEMYKSGMELMFKNKNNFLFSPCVE-RDTGVEKFLDDTPVNILKK  334

Query  937   ----NIPAIYGVNNKEFLTLHAFVTPEDYEVTGIRAFLERAFNLT-DSEMEDHVRHFYIG  1101
                  +P +YG  N E L    F     YE    +   + + NL  D E +D      +
Sbjct  335   GDYNKVPVLYGFANMEGL----FRVGMGYEYFMSQLNDKFSENLPHDLEFKDEEEKRKVA  390

Query  1102  DETLTEKFFDEFIDFASDYYYNYGVQR  1182
             DE   +KF+  F D  +   YN G+ R
Sbjct  391   DE--VKKFY--FGDKKN**RYNLGIHR  413


> ta_transcript84818_1
Length=733

 Score =   157 bits (396),  Expect = 3e-40, Method: Compositional matrix adjust.
 Identities = 130/387 (34%), Positives = 192/387 (50%), Gaps = 29/387 (7%)
 Frame = +1
```

```
Query  70    VDTQRGLIRGLRSENGKFAKFLGIPYALVDE-NNPFGPSVPHPGFEETFEAYDDSVVCPQ  246
             V+ Q+G +RG +   G    F  IPYA      + F   +P P +  T EA D  VVC Q
Sbjct  40    VEIQQGKLRGYKDPEGGLFTFYNIPYATAPTGKDRFKAPLPAPVWITTREAVDRGVVCHQ  99

Query  247   V-TKGVGV----GSLQCLNLNVYVPNTATSRNKRPVMIWIHGGSFATGSGTGRDFSYDDL  411
             +    VG+         CL  NVYVP+T   +   PV++++HGG++   +G G   +   +
Sbjct  100   IDLAAVGIFNKTKQEDCLIANVYVPDT--DKKNLPVVVFVHGGAYL--AGFGEVLTPKHM  155

Query  412   VR-HDVIVVSVNYRLGPYGFLCLDSPDIPGNQGLKDQALALRWIKENIEAFGGDVSKITL  588
             VR ++VI V+ NYRLG  GFLCL +PD PGN G+KD    LRW+K+NI +FGG+   +T+
Sbjct  156   VRTNNVIYVTFNYRLGVQGFLCLGTPDAPGNAGMKDMVALLRWVKKNIASFGGNPDDVTI  215

Query  589   FGESAGGVAVDLHLLTDKDE-LFNQVIIQSGSAFFAGGIR-KPNNRVPIEIASQLGFETD  762
              G SAG  AVDL LL+   + LF++ I +SG+      ++  P         S+ GFE
Sbjct  216   DGYSAGSSAVDLLLLSKATKGLFHKAIPESGANVAMWSVQVDPIANAKEYAKSEHGFEIL  275

Query  763   NFAEAIKFLVDQDPHLVVAASTSQRSTLGGNT--LRPCLENKYDGVDSFLSDFPENLRAR  936
             +   A++      P+           N     PC+E +  GV+ FL D P N+  +
Sbjct  276   DDIYALEDFYKSLPYDEMYKSGMELMFKNKNNFLFSPCVE-RDTGVEKFLDDTPVNILKK  334

Query  937   ----NIPAIYGVNNKEFLTLHAFVTPEDYEVTGIRAFLERAFNLT-DSEMEDHVRHFYIG  1101
                  +P +YG  N E L    F     YE    +    + + NL  D E +D      +
Sbjct  335   GDYNKVPVLYGFANMEGL----FRVGMGYEYFMSQLNDKFSENLPHDLEFKDEEEKRKVA  390

Query  1102  DETLTEKFFDEFIDFASDYYYNYGVQR  1182
             DE   +KF+  F D  +   YN G+ R
Sbjct  391   DE--VKKFY--FGDKKN**RYNLGIHR  413
```

> ta_transcript84816_1
Length=947

```
 Score =  155 bits (393),  Expect = 2e-39, Method: Compositional matrix adjust.
 Identities = 124/371 (33%), Positives = 179/371 (48%), Gaps = 34/371 (9%)
 Frame = +1

Query  70    VDTQRGLIRGLRSENGKFAKFLGIPYALVDE-NNPFGPSVPHPGFEETFEAYDDSVVCPQ  246
             V+ Q+G +RG +   G    F  IPYA      + F   +P P +  T EA D  VVC Q
Sbjct  40    VEIQQGKLRGYKDPEGGLFTFYNIPYATAPTGKDRFKAPLPAPVWITTREAVDRGVVCHQ  99

Query  247   V-TKGVGV----GSLQCLNLNVYVPNTATSRNKRPVMIWIHGGSFATGSGTGRDFSYDDL  411
             +    VG+         CL  NVYVP+T   +   PV++++HGG++   +G G   +   +
Sbjct  100   IDLAAVGIFNKTKQEDCLIANVYVPDT--DKKNLPVVVFVHGGAYL--AGFGEVLTPKHM  155

Query  412   VR-HDVIVVSVNYRLGPYGFLCLDSPDIPGNQGLKDQALALRWIKENIEAFGGDVSKITL  588
             VR ++VI V+ NYRLG  GFLCL +PD PGN G+KD    LRW+K+NI +FGG+   +T+
Sbjct  156   VRTNNVIYVTFNYRLGVQGFLCLGTPDAPGNAGMKDMVALLRWVKKNIASFGGNPDDVTI  215

Query  589   FGESAGGVAVDLHLLTDKDE-LFNQVIIQSGSAFFAGGIR-KPNNRVPIEIASQLGFETD  762
              G SAG  AVDL LL+   + LF++ I +SG+      ++  P         S+ GFE
Sbjct  216   DGYSAGSSAVDLLLLSKATKGLFHKAIPESGANVAMWSVQVDPIANAKEYAKSEHGFEIL  275

Query  763   NFAEAIKFLVDQDPHLVVAASTSQRSTLGGNT--LRPCLENKYDGVDSFLSDFPENLRAR  936
             +   A++      P+  +  S  +      N     PC+E    GV+ FL D P N+  +
Sbjct  276   DDIYALEDFYKSLPYDEMYKSGMELMFKNKNNFLFSPCVERD-TGVEKFLDDTPVNILKK  334

Query  937   ----NIPAIYGVNNKEFLTLHAFVTPEDYEV------TGIRAFLERAFNLTDSE----ME  1074
                  +P +YG  N E L    F     YE              L       D E    +
Sbjct  335   GDYNKVPVLYGFANMEGL----FRVGMGYEYFMSQLNDKFSENLPHDLEFKDEEEKRKVA  390

Query  1075  DHVRHFYIGDE  1107
             D V+ FY GD+
Sbjct  391   DEVKKFYFGDK  401
```

> ta_transcript15565_1
Length=424

```
 Score =  123 bits (309),  Expect = 6e-30, Method: Compositional matrix adjust.
 Identities = 105/386 (27%), Positives = 181/386 (47%), Gaps = 47/386 (12%)
 Frame = +1

Query  505   GLKDQALALRWIKENIEAFGGDVSKITLFGESAGGVAVDLHLLTDKDE-LFNQVIIQSGS  681
             G+KD    LRW+  NI AFGG+ + +TL G SAG V+V+L +L+   + LF++ I +SGS
Sbjct  1     GMKDMVALLRWVNRNIAAFGGNPNDVTLEGWSAGSVSVELLMLSKAAKGLFHKAIPESGS  60
```

```
Query  682   AF--FAGGIRKPNNRVPIEIASQLGF-ETDNFAEAIKF--------LVDQDPHLVVAAST  828
             +F  F+ G+  P         S LGF + DN +   +F        +  Q P ++   +
Sbjct  61    SFAPFSVGV-DPIGNAKDFARSDLGFKDVDNISALEEFYKTVPFKDMYQQGPLIIFRHNF  119

Query  829   SQRSTLGGNTLRPCLENKYDGVDSFLSDFPENLRAR----NIPAIYGVNNKEFLTLHAFV  996
                      T  PC+E    G D FL D P N+  +     +P +YG +NKE   L+
Sbjct  120   ---------TFTPCVERNI-GQDVFLDDTPYNILKKGDYMKVPMLYGFSNKE--GLYRVD  167

Query  997   TPEDYEVTGIRA----FLERAFNLTDSEMEDHV----RHFYIGDETLTEKFFDEFIDFAS  1152
                 + + G+      +L       D   ++ V    + FY G++ + +    E+ID+ +
Sbjct  168   YGYQFFIEGMNKKFSDYLPNDLQFKDENEKEQVATMIKEFYFGNKKIDQDTVLEYIDYFT  227

Query  1153  DYYYNYGVQRSIKKSLADGNKEVYYYVFSYDGGRNAMKQYLGITAEGAAHADE----LGY  1320
             D  +     + ++  +A GN ++Y Y FSY    N + + + +   G+ H  E    L
Sbjct  228   DSMFAGPSLKEVQLHVAAGNNQIYLYQFSYP-YENKVPESIKVKVIGSNHCGETMTVLDN  286

Query  1321  LLAVDAVPGQHIAEEDQLIIDRITTLWANFAKYGYQPTPEPTDLLPVVWTTVEGNKRPYL  1500
              L    +P +  ++ +Q + +    +W NF K G +P P+ ++L    W   +G+  PY+
Sbjct  287   WLGGAEMPAERNSDLEQ-VRNVTRQIWGNFIKTG-KPVPQGSNL--PTWPATDGSGSPYM  342

Query  1501  DIDTDLQLRSRPFH-HRMAFWDLFYK  1575
              I    +L+ +PF    + FW   Y+
Sbjct  343   VITNKPELKGQPFQPMSVMFWTEIYQ  368




Query= gi|530233691|ref|NM_001281891.1| Bombyx mori juvenile hormone esterase-like
(LOC101743505), mRNA

Length=1596
                                                                      Score     E
Sequences producing significant alignments:                          (Bits)  Value

  ta_transcript58915_1                                                  206   8e-58
  ta_transcript58914_1                                                  206   2e-57
  ta_transcript48393_1                                                  199   9e-55
  ta_transcript29751_1                                                  194   3e-54
  ta_transcript75783_1                                                  196   1e-53
  ta_transcript48580_1                                                  186   4e-50
  ta_transcript48579_1                                                  186   1e-49
  ta_transcript84821_1                                                  162   2e-42
  ta_transcript84818_1                                                  162   3e-42
  ta_transcript84816_1                                                  162   1e-41


> ta_transcript58915_1
Length=606

 Score =   206 bits (525),  Expect = 8e-58, Method: Compositional matrix adjust.
 Identities = 146/484 (30%), Positives = 223/484 (46%), Gaps = 48/484 (10%)
 Frame = +1

Query  91    QGLVRGHK----AADGDYSIFLGIPYAKVDVNN-PFSTATDPIAFPEIIHDAVDEVRCPQ  255
             QG + G +    + DG +  F GIPYA   V    F     P+++  +        +CPQ
Sbjct  36    QGWLEGEQLDSVSGDGQFFSFKGIPYAAPPVGKLRFKAPQPPVSWQGVRKATQHGPKCPQ  95

Query  256   T---SSSVGEQTLNCLRLNIYVPSAASSRNPMPVLVWIHGGDFATGSASD--YGVKNIVR  420
                 S  +   + +CL LN+Y     S ++P+PV+V+IHGG F +GS  +  YG   +V
Sbjct  96    RDIFSKQIIPGSEDCLYLNVYTKDI-SPKSPLPVMVFIHGGGFKSGSGDEDFYGGDFLVH  154

Query  421   HGVLVVTMNYRLGPYGFLCLDVPTASGNQGLRDQYKALSWIRNNIASFGGNPYNVTIAGQ  600
             HGV++VT+NYRL   GFLCLD     GN GL+DQ  AL W++ NI +FGG+P NVTI G+
Sbjct  155   HGVVLVTINYRLDALGFLCLDTEEVPGNAGLKDQVAALKWVQKNIFAFGGDPTNVTIFGE  214

Query  601   DAGATSVLLQLYSDNEK-LFHKVIIESGTPQSEGMFVNADVEAAIKLAVHLGLNTTETEE  777
              AG +S  L + S   K LF + I  SG P  +        + A  L   LG  T + +
Sbjct  215   SAGGSSTALHVLSPLSKGLFKRAIPMSGVPFCDWSIPFEPRKRAFTLGKILGFETEDPKA  274

Query  778   AIQFL--------TKTSPDLVVAAAKELEL----QLKPCRERSFSGISNFVESDPYSLTN  921
              +++L          T+P ++    K   +       P  E++F G  +F+  +P
Sbjct  275   LLEYLQSLPAEKFVDTNPTIMGFEEKSYNMLKMYHFTPVVEKNF-GQHHFMTEEPLEALK  333

Query  922   KKKVRNTPIMIGNTNKEKKSLSADYFSTDPFYEKLKSNF-------NLDEAQIEKAAKNV  1080
             +  V +  ++IGNT++E       +      Y++    F            +I + A+ +
Sbjct  334   QGHVNDVDVLIGNTDQETIVGIPSFVDLLKMYDRYPEVFVPRKILNKSTPGKILEIAERI  393
```

```
Query  1081  RHFYIGDSSLSAEVASELQDFESDFVYNHPSQRTITQLLDENAG--KIYEYLFSYSGELE  1254
             R  Y GD  +      E   + S+  + +   +    LL    G  K+Y Y FS   E
Sbjct  394   RKHYFGDKPIDKTSMKEAVTYFSEVCFTYDVNKYTNLLLSGKPGSSKVYRYRFSCVSERN  453

Query  1255  -----------DGAEHSSELNYLFETAGGS---DRTEEEQLMVDRITTLWTNFVKFGNPT  1392
                         GA H  +L YLF+        DR  +   M+ +   L+TNF K+GNPT
Sbjct  454   IFGKYGQEYGITGASHLEDLMYLFDAKSVKLPLDRNSKSYKMIQQTCALFTNFAKYGNPT  513

Query  1393  PKST  1404
             P S+
Sbjct  514   PDSS  517
```

> ta_transcript58914_1
Length=650

```
 Score =   206 bits (525),  Expect = 2e-57, Method: Compositional matrix adjust.
 Identities = 146/484 (30%), Positives = 223/484 (46%), Gaps = 48/484 (10%)
 Frame = +1

Query  91    QGLVRGHK----AADGDYSIFLGIPYAKVDVNN-PFSTATDPIAFPEIIHDAVDEVRCPQ  255
             QG + G +    + DG +  F GIPYA   V    F     P+++  +        +CPQ
Sbjct  36    QGWLEGEQLDSVSGDGQFFSFKGIPYAAPPVGKLRFKAPQPPVSWQGVRKATQHGPKCPQ  95

Query  256   T---SSSVGEQTLNCLRLNIYVPSAASSRNPMPVLVWIHGGDFATGSASD--YGVKNIVR  420
                 S  +   + +CL LN+Y     S ++P+PV+V+IHGG F +GS  +  YG   +V
Sbjct  96    RDIFSKQIIPGSEDCLYLNVYT-KDISPKSPLPVMVFIHGGGFKSGSGDEDFYGGDFLVH  154

Query  421   HGVLVVTMNYRLGPYGFLCLDVPTASGNQGLRDQYKALSWIRNNIASFGGNPYNVTIAGQ  600
             HGV++VT+NYRL   GFLCLD     GN GL+DQ  AL W++ NI +FGG+P NVTI G+
Sbjct  155   HGVVLVTINYRLDALGFLCLDTEEVPGNAGLKDQVAALKWVQKNIFAFGGDPTNVTIFGE  214

Query  601   DAGATSVLLQLYSDNEK-LFHKVIIESGTPQSEGMFVNADVEAAIKLAVHLGLNTTETEE  777
              AG +S  L + S   K LF + I  SG P  +        + A  L   LG  T + +
Sbjct  215   SAGGSSTALHVLSPLSKGLFKRAIPMSGVPFCDWSIPFEPRKRAFTLGKILGFETEDPKA  274

Query  778   AIQFL--------TKTSPDLVVAAAKELEL----QLKPCRERSFSGISNFVESDPYSLTN  921
              +++L          T+P ++    K   +       P  E++F G  +F+  +P
Sbjct  275   LLEYLQSLPAEKFVDTNPTIMGFEEKSYNMLKMYHFTPVVEKNF-GQHHFMTEEPLEALK  333

Query  922   KKKVRNTPIMIGNTNKEKKSLSADYFSTDPFYEKLKSNF-------NLDEAQIEKAAKNV  1080
             +  V +  ++IGNT++E       +      Y++    F            +I + A+ +
Sbjct  334   QGHVNDVDVLIGNTDQETIVGIPSFVDLLKMYDRYPEVFVPRKILNKSTPGKILEIAERI  393

Query  1081  RHFYIGDSSLSAEVASELQDFESDFVYNHPSQRTITQLLDENAG--KIYEYLFSYSGELE  1254
             R  Y GD  +      E   + S+  + +   +    LL    G  K+Y Y FS   E
Sbjct  394   RKHYFGDKPIDKTSMKEAVTYFSEVCFTYDVNKYTNLLLSGKPGSSKVYRYRFSCVSERN  453

Query  1255  -----------DGAEHSSELNYLFETAGGS---DRTEEEQLMVDRITTLWTNFVKFGNPT  1392
                         GA H  +L YLF+        DR  +   M+ +   L+TNF K+GNPT
Sbjct  454   IFGKYGQEYGITGASHLEDLMYLFDAKSVKLPLDRNSKSYKMIQQTCALFTNFAKYGNPT  513

Query  1393  PKST  1404
             P S+
Sbjct  514   PDSS  517
```

> ta_transcript48393_1
Length=666

```
 Score =   199 bits (505),  Expect = 9e-55, Method: Compositional matrix adjust.
 Identities = 135/416 (32%), Positives = 201/416 (48%), Gaps = 36/416 (9%)
 Frame = +1

Query  76    VLISNQGLVRGHKAADGDYSIFLGIPYAKVDVNNP--FSTATDPIAFPEIIHDAVDEVRC  249
             ++ + QG VRG++  +G    F  IPYA    + P  F+    P  + E        + C
Sbjct  95    IVETTQGPVRGYRDPEGGLFAFYSIPYATAP-SGPLKFTAPITPPTWTEPFEAVDKRIIC  153

Query  250   PQTSSSVGEQT---LNCLRLNIYVPSAASSRNPMPVLVWIHGGDFATGSASDYGVKNIVR  420
             PQ  S   +Q    ++CL  N+YVP    ++  +PV+V++HGG F  G  +    KN+VR
Sbjct  154   PQMKSGFTKQMTMQVDCLIANVYVPDTKETK--LPVIVYVHGGAFQVGFGNLMTPKNLVR  211

Query  421   -HGVLVVTMNYRLGPYGFLCLDVPTASGNQGLRDQYKALSWIRNNIASFGGNPYNVTIAG  597
                ++ VT NYRLG +GFLCL    A GN GL+DQ  AL W+  NI   GGNP +VTIAG
Sbjct  212   SKNIIAVTFNYRLGAHGFLCLGTEDAPGNAGLKDQVAALRWVNKNIIKLGGNPDDVTIAG  271
```

```
Query  598   QDAGATSV-LLQLYSDNEKLFHKVIIESGTPQSEGMFVNADVEAAIKLAVHLGLNTTETE  774
               AGA SV L+ +    E LF K+IIESG+    G  V   +E A   A  LG N  +
Sbjct  272   YSAGAISVDLMAISKSAEGLFSKIIIESGSSLFAGSIVTDPLENAKIFAARLGFNNIDDV  331

Query  775   EAIQFLTKTSPDLVVAAAKELE-----LQLKPCRERSFSGISNFVESDPYSLTNKKKVRN  939
              A++   K +    +   + ++     L   PC ER   G++ F+   P ++   +
Sbjct  332   YALEKFYKAASYDSLTKGEFMDRPNSTLLFSPCVERDDDGLA-FLSEPPINILKEGSYTQ  390

Query  940   TPIMIGNTNKEKKSLSADYFSTDPFYE--KLKSNFNLDE-----------AQIEKAAKNV  1080
              P++ G +N E            +PF+E  KLK N    E              E+ AK +
Sbjct  391   VPMIYGFSNMEG-------LFREPFFEFWKLKMNEQFSEFIPGDLHFESKHDRERIAKRI  443

Query  1081  RHFYIGDSSLSAEVASELQDFESDFVYNHPSQRTITQLLDENAGKIYEYLFSYSGE  1248
             + FY  DS ++ E   +  D+  D +  +P+ RT+   +     K+Y Y +SY  E
Sbjct  444   KKFYFWDSPINGENILKYIDYFGDVLVKYPALRTVKMHIQNGHDKLYLYEYSYVDE  499


> ta_transcript29751_1
Length=506

 Score =   194 bits (494),  Expect = 3e-54, Method: Compositional matrix adjust.
 Identities = 152/499 (30%), Positives = 244/499 (49%), Gaps = 51/499 (10%)
 Frame = +1

Query  22    LLLMLSYVQANLVRVDPLVLISNQGLVRGHKAADGDYSIFLGIPYAKVDVNNPFSTATDP  201
             +L  L++ Q      D   ++  QG VRG+K  DG   +F  IPYA    +     A  P
Sbjct  19    ILCALAHAQE-----DSRTVVIEQGPVRGYKDPDGGLYVFYNIPYATAPTSKDRFKA--P  71

Query  202   IAFPE--IIHDAVD-EVRCPQTSSSVGEQTL--NCLRLNIYVPSAASSRNPMPVLVWIHG  366
             +A P    I DAVD EV CPQ    +    + +  +CL  N++VP   +S   + V+V +HG
Sbjct  72    LAPPVWVTIRDAVDREVVCPQMLETGSHKNIQEDCLIANVFVPD--TSEKNLSVVVLVHG  129

Query  367   GDFATGSASDYGVKNIVRHGVLV-VTMNYRLGPYGFLCLDVPTASGNQGLRDQYKALSWI  543
             G + +G  +    K++VR   L+ VT NYRLG +GFLCL       GN G++D    L W+
Sbjct  130   GGYLSGFGNRQTPKDMVRDKKLIYVTFNYRLGVHGFLCLGTADVPGNAGMKDMVALLRWV  189

Query  544   RNNIASFGGNPYNVTIAGQDAGATSVLLQLYSD-NEKLFHKVIIESGTPQSEGMFVNADV  720
             + NIASFGGNP +VTI G  +G++SV L L S   E LFHK I ESG+  +         +
Sbjct  190   KKNIASFGGNPDDVTIDGHSSGSSSVDLLLLSKATEGLFHKAIPESGSNVAMWSVQIDPI  249

Query  721   EAAIKLAV-HLGLNTTETEEAIQFLTKTSP-DLVVAAAKELE-------LQLKPCRERSF  873
               A + A       + +   A++    KT P D++  +   L         +  +PC ER
Sbjct  250   AHAKEFARDEHSFESGDDIYALEDFYKTLPFDVMYKSGMSLRYNSSVKNILFQPCVERD-  308

Query  874   SGISNFVESDPYSLTNKKKVRNTPIMIGNTNKEKKSLSADYFSTDPFYEKLKSNFN----  1041
             +G+  F++  P ++         P++ G TN E     +D+ +   F   +  F+
Sbjct  309   TGVEKFLDDFPVNILKSGDYNKVPMLYGFTNMEGLMRVSDFVNYRSFMSDINEKFSDYLP  368

Query  1042  -----LDEAQIEKAAKNVRHFYIGDSSLSAEVASELQDFESDFVYNHPSQRTITQLLDEN  1206
                   D+ + EK A++V+ FY GD  ++ +   E  D+ SD ++ +P+ R++   ++
Sbjct  369   NDLQFRDKEEKEKVARDVKQFYFGDKVINNDTIFEYIDYFSDNMFAYPTLRSLKLQVEAG  428

Query  1207  AGKIYEYLFSYSGELE---------DGAEHSSELNYLFETAGGSDRTEEEQLMVDRITT-  1356
               ++Y Y FSY  E +         +G+ H+++ + + +T       EE  L + R  T
Sbjct  429   NNQVYLYQFSYPFENQAPEGVMLKLEGSSHTAQTHVVLDTL-----NEENDLAIIRNITR  483

Query  1357  -LWTNFVKFGNPTPKSTDL  1410
               W NF+  G P P  ++L
Sbjct  484   QFWANFITTGTPVPAGSEL  502


> ta_transcript75783_1
Length=733

 Score =   196 bits (499),  Expect = 1e-53, Method: Compositional matrix adjust.
 Identities = 155/547 (28%), Positives = 259/547 (47%), Gaps = 63/547 (12%)
 Frame = +1

Query  52    NLVRVDPLVLISNQGLVRGHKAAD---GDYSIFLGIPYAKVDVNN----------PFSTA  192
             N + +D  V +S +G+++G    +    G Y  F GIP+A+  V +           P+
Sbjct  118   NKMSLDNTVKVS-EGILQGELVQNEFGGSYYSFKGIPFAEPPVGDLRFKAPIPAKPWKGV  176

Query  193   TDPIAFPEIIHDAVDEVRCPQTSSSVGEQTLNCLRLNIYVPSAASSRNPMPVLVWIHGGD  372
                  F  I +     + P + S       +CL LN+Y P        +PV+VWIHGG
Sbjct  177   KTATKFGPINYQFDVFKKDPPSGSE------DCLYLNVYTPEIKPI-TLLPVMVWIHGGG  229

Query  373   FATGSASD--YGVKNIVRHGVLVVTMNYRLGPYGFLCLDVPTASGNQGLRDQYKALSWIR  546
```

```
             +  GS +D  YG + +VRHGV++VT NYRLG  GFL LD     GN G++DQ  AL W++
Sbjct  230   YFWGSGNDDVYGPEFLVRHGVILVTFNYRLGVLGFLSLDTAEVPGNAGMKDQVAALKWVK  289

Query  547   NNIASFGGNPYNVTIAGQDAGATSVLLQLYSDNEK-LFHKVIIESGTPQSEGMFVNADVE  723
             +NI  FGG+P N+TI G+ AGA +V L L S   K LF + II+SG+  +         E
Sbjct  290   SNIHFFGGDPENITIFGESAGAGAVSLHLLSPMSKGLFKRAIIQSGSATAYWAQAFEIKE  349

Query  724   AAIKLAVHLGLNTTETEEAIQFLTKTSPDLVV--------AAAKELELQLKPCRERSFSG  879
              +I LA  LGL T + ++  +F    S +L++        A  +E+ ++  P  E+ F G
Sbjct  350   KSIALARKLGLVTDDDQKLYEFFGNISKELLINISVPVTFAEQEEVNIEFAPTIEKDF-G  408

Query  880   ISNFVESDPYSLTNKKKVRNTPIMIGNTNKEK-------KSLSADYFSTDPFYEKLKSNF  1038
                F+  D Y +  +   +   +M+G    E        + L  +    + F E L
Sbjct  409   QERFLTDDSYKILQQGIHKGVDVMMGYVEDEGVLYLNSLEILDKEIERINNFLEALVPRN  468

Query  1039  NLDEAQIE---KAAKNVRHFYIGDSSLSAEVASELQDFESDFVYNH---PSQRTIT----  1188
                E  I+   K  + ++ FY  +  ++      +  + S  V+++    SQ+ I
Sbjct  469   MKREIPIKQQLKVGRKMKKFYFDNKLVTRNDWRTIARYISLNVFSYDVIQSQKNIAKSQN  528

Query  1189  ----QLLDENAGKIYEYLFSYSGELE---DGAEHSSELNYLFETAGGSD---RTEEEQLM  1338
                 +   ++    ++ +F+   E+    D   H  +L YLF     +    +   E  +
Sbjct  529   SFFYKFTCKSKRNFFKKVFT-DDEITKDVDVVCHCDDLAYLFPVKFMNQSVAKDSREFQL  587

Query  1339  VDRITTLWTNFVKFGNPTPKSTDLIPTIWEPVTADTRPVLVLDKDVRLDSRIERQRMAYW  1518
             +D++T LWTNF K+GNPTP ++  +   WEP T   +  L + +D+ L ++ + + + +W
Sbjct  588   IDKVTKLWTNFAKYGNPTPDNS--LGVKWEPFTLQHQYLDIGEDLVLKTKPDDEDIQFW  645

Query  1519  DLFFTTY  1539
             +  +  Y
Sbjct  646   EELYREY  652


> ta_transcript48580_1
Length=834

 Score =   186 bits (473),  Expect = 4e-50, Method: Compositional matrix adjust.
 Identities = 162/546 (30%), Positives = 241/546 (44%), Gaps = 65/546 (12%)
 Frame = +1

Query  76    VLISNQGLVRGHKA-----ADGDYSIFLGIPYAKVDVNNPFSTATDPIAFPEIIHDAVDE  240
             ++++  G +RG +      A   Y  F GI YA+    +       P+     + DA++E
Sbjct  155   IVMTELGALRGRRVVARTTAQTPYFSFKGIRYAQPPRGSLRFKPPVPLEPWSGVRDALEE  214

Query  241   -VRCPQT----SSSVGEQTLNCLRLNIYVPS---AASSRNP-MPVLVWIHGGDFATGSAS  393
                CP       +  G++  +CL LN+Y P+     +  NP + V+VWIHGG FA GS +
Sbjct  215   GAVCPHRFMLFDTYKGDE--DCLFLNVYTPALPEKITGFNPKLAVMVWIHGGAFAVGSGN  272

Query  394   D--YGVKNIVRHGVLVVTMNYRLGPYGFLCLDVPTASGNQGLRDQYKALSWIRNNIASFG  567
                YG  +++   +++VT+NYRLG  GFL ++    SGN GL+DQ  AL W+R+NI  FG
Sbjct  273   AFLYGPDHLMGGNIVLVTLNYRLGALGFLSMENDEVSGNMGLKDQVLALKWVRDNIEYFG  332

Query  568   GNPYNVTIAGQDAGATSVLLQLYSDNEK-LFHKVIIESGTPQSEGMFVNADVEAAIKLAV  744
             G+   VTI G+ AGA SV L + S   K LFH+ I +SG   S      +  E A +L
Sbjct  333   GDASRVTIFGESAGAASVHLHMLSPASKGLFHRAIAQSGLALSPWALTRSPRERAFELGR  392

Query  745   HLGLNTTETEEAIQFLTKTSPDLVVAAAKEL--------------ELQLKPCRERSFSGI  882
              LG++T  T E + +L  T  +L+V A   L               L   P  E
Sbjct  393   ELGIDTNSTAELLGYLRATPSELLVKAGARLAGAPGKSVDLHSTVALPFVPTVEPDVP--  450

Query  883   SNFVESDPYSLTNKKKVRNTPIMIGNTNKEKKSLSADYFSTDPFYEKLKSNFNL------  1044
               F+   P +L     V   P++ G   +E   L            +L   F
Sbjct  451   DAFLTQHPRNLMPGADV---PLLTGYNAQEGIILFRRLQRYPKLLTELDREFRRVVPPEL  507

Query  1045  ---DEAQIEKAAKNVRHFYIGDSSLSAEVASELQDFESDFVYNHPSQRTI-TQLLDENAG  1212
                DEAQ  + A ++R FY     +       L D  +D ++  P   T+  Q
Sbjct  508   LTGDEAQNRRVADHIRAFYFQQRPVDIRNIDSLIDLFTDVMFLRPLLETLRIQGKTNRTS  567

Query  1213  KIYEYLFSYSGEL----------EDGAEHSSELNYLFETAGGSDRTEEE--QLMVDR-IT  1353
               Y Y F++ G L            GA H  E+ YLF  +  + R +++  +L V + +
Sbjct  568   PTYLYRFAFDGALGLFKRMLGINHPGACHGDEMGYLFYFSRLNYRLDDDSTELAVSKGMV  627

Query  1354  TLWTNFVKFGNPT-PKSTDLIPTIWEPVTADTRPVLVLDKDVRLDSR--IERQRMAYWDL  1524
              +WTNF K GNPT     D I   W PV  DT  +  LD +     +   E +R+  WD
Sbjct  628   RMWTNFAKTGNPTIGDHDDPIDFKWSPVN-DTTHLTYLDINGHFTQKQDPEARRVRLWDW  686

Query  1525  FFTTYG  1542
              +  Y
```

```
Sbjct  687   LYENYA  692


> ta_transcript48579_1
Length=905

 Score =   186 bits (471),  Expect = 1e-49, Method: Compositional matrix adjust.
 Identities = 162/546 (30%), Positives = 241/546 (44%), Gaps = 65/546 (12%)
 Frame = +1

Query  76    VLISNQGLVRGHKA-----ADGDYSIFLGIPYAKVDVNNPFSTATDPIAFPEIIHDAVDE  240
             ++++  G +RG +      A   Y  F GI YA+    +       P+   +   DA++E
Sbjct  155   IVMTELGALRGRRVVARTTAQTPYFSFKGIRYAQPPRGSLRFKPPVPLEPWSGVRDALEE  214

Query  241   -VRCPQT----SSSVGEQTLNCLRLNIYVPS---AASSRNP-MPVLVWIHGGDFATGSAS  393
                CP       +  G++  +CL LN+Y P+     +  NP + V+VWIHGG FA GS +
Sbjct  215   GAVCPHRFMLFDTYKGDE--DCLFLNVYTPALPEKITGFNPKLAVMVWIHGGAFAVGSGN  272

Query  394   D--YGVKNIVRHGVLVVTMNYRLGPYGFLCLDVPTASGNQGLRDQYKALSWIRNNIASFG  567
                YG  +++   +++VT+NYRLG  GFL ++    SGN GL+DQ  AL W+R+NI  FG
Sbjct  273   AFLYGPDHLMGGNIVLVTLNYRLGALGFLSMENDEVSGNMGLKDQVLALKWVRDNIEYFG  332

Query  568   GNPYNVTIAGQDAGATSVLLQLYSDNEK-LFHKVIIESGTPQSEGMFVNADVEAAIKLAV  744
             G+   VTI G+ AGA SV L + S   K LFH+ I +SG   S      +  E A +L
Sbjct  333   GDASRVTIFGESAGAASVHLHMLSPASKGLFHRAIAQSGLALSPWALTRSPRERAFELGR  392

Query  745   HLGLNTTETEEAIQFLTKTSPDLVVAAAKEL--------------ELQLKPCRERSFSGI  882
              LG++T  T E + +L  T  +L+V A   L               L   P  E
Sbjct  393   ELGIDTNSTAELLGYLRATPSELLVKAGARLAGAPGKSVDLHSTVALPFVPTVEPDVP--  450

Query  883   SNFVESDPYSLTNKKKVRNTPIMIGNTNKEKKSLSADYFSTDPFYEKLKSNFNL------  1044
               F+   P +L     V   P++ G   +E   L            +L   F
Sbjct  451   DAFLTQHPRNLMPGADV---PLLTGYNAQEGIILFRRLQRYPKLLTELDREFRRVVPPEL  507

Query  1045  ---DEAQIEKAAKNVRHFYIGDSSLSAEVASELQDFESDFVYNHPSQRTI-TQLLDENAG  1212
                DEAQ  + A ++R FY     +       L D  +D ++  P   T+  Q
Sbjct  508   LTGDEAQNRRVADHIRAFYFQQRPVDIRNIDSLIDLFTDVMFLRPLLETLRIQGKTNRTS  567

Query  1213  KIYEYLFSYSGEL----------EDGAEHSSELNYLFETAGGSDRTEEE--QLMVDR-IT  1353
               Y Y F++ G L            GA H  E+ YLF  +  + R +++   +L V + +
Sbjct  568   PTYLYRFAFDGALGLFKRMLGINHPGACHGDEMGYLFYFSRLNYRLDDDSTELAVSKGMV  627

Query  1354  TLWTNFVKFGNPT-PKSTDLIPTIWEPVTADTRPVLVLDKDVRLDSRI--ERQRMAYWDL  1524
              +WTNF K GNPT     D I   W PV  DT  +  LD +     +   E +R+  WD
Sbjct  628   RMWTNFAKTGNPTIGDHDDPIDFKWSPVN-DTTHLTYLDINGHFTQKQDPEARRVRLWDW  686

Query  1525  FFTTYG  1542
              +  Y
Sbjct  687   LYENYA  692


> ta_transcript84821_1
Length=611

 Score =   162 bits (411),  Expect = 2e-42, Method: Compositional matrix adjust.
 Identities = 123/360 (34%), Positives = 176/360 (49%), Gaps = 26/360 (7%)
 Frame = +1

Query  91    QGLVRGHKAADGDYSIFLGIPYAKVDVNNPFSTATDPIAFPEIIHDAVDE-VRCPQTS-S  264
             QG +RG+K  +G    F  IPYA          A  P        +AVD  V C Q   +
Sbjct  44    QGKLRGYKDPEGGLFTFYNIPYATAPTGKDRFKAPLPAPVWITTREAVDRGVVCHQIDLA  103

Query  265   SVG----EQTLNCLRLNIYVPSAASSRNPMPVLVWIHGGDFATGSASDYGVKNIVR-HGV  429
             +VG     +  +CL  N+YVP   + +  +PV+V++HGG +  G       K++VR + V
Sbjct  104   AVGIFNKTKQEDCLIANVYVPD--TDKKNLPVVVFVHGGAYLAGFGEVLTPKHMVRTNNV  161

Query  430   LVVTMNYRLGPYGFLCLDVPTASGNQGLRDQYKALSWIRNNIASFGGNPYNVTIAGQDAG  609
             + VT NYRLG  GFLCL  P A GN G++D    L W++ NIASFGGNP +VTI G  AG
Sbjct  162   IYVTFNYRLGVQGFLCLGTPDAPGNAGMKDMVALLRWVKKNIASFGGNPDDVTIDGYSAG  221

Query  610   ATSVLLQLYSDNEK-LFHKVIIESGTPQSE-GMFVNADVEAAIKLAVHLGLNTTETEEAI  783
             +++V L L S   K LFHK I ESG   +   + V+    A        G    +   A+
Sbjct  222   SSAVDLLLLSKATKGLFHKAIPESGANVAMWSVQVDPIANAKEYAKSEHGFEILDDIYAL  281

Query  784   QFLTKTSP-DLVVAAAKELELQLK------PCRERSFSGISNFVESDPYSLTNKKKVRNT  942
             +   K+ P D +  +  EL  + K      PC ER  +G+  F++  P ++  K
Sbjct  282   EDFYKSLPYDEMYKSGMELMFKNKNNFLFSPCVERD-TGVEKFLDDTPVNILKKGDYNKV  340
```

```
Query  943    PIMIGNTNKE---KKSLSADYFST---DPFYEKLKSNFNL-DEAQIEKAAKNVRHFYIGD  1101
              P++ G  N E    +  +  +YF +    D F E L  +    DE +  K A  V+ FY GD
Sbjct  341    PVLYGFANMEGLFRVGMGYEYFMSQLNDKFSENLPHDLEFKDEEEKRKVADEVKKFYFGD  400


> ta_transcript84818_1
Length=733

 Score =  162 bits (411),  Expect = 3e-42, Method: Compositional matrix adjust.
 Identities = 123/360 (34%), Positives = 176/360 (49%), Gaps = 26/360 (7%)
 Frame = +1

Query  91     QGLVRGHKAADGDYSIFLGIPYAKVDVNNPFSTATDPIAFPEIIHDAVDE-VRCPQTS-S  264
              QG +RG+K  +G    F  IPYA           A  P        +AVD  V C Q   +
Sbjct  44     QGKLRGYKDPEGGLFTFYNIPYATAPTGKDRFKAPLPAPVWITTREAVDRGVVCHQIDLA  103

Query  265    SVG----EQTLNCLRLNIYVPSAASSRNPMPVLVWIHGGDFATGSASDYGVKNIVR-HGV  429
              +VG      +   +CL  N+YVP    + +   +PV+V++HGG +  G        K++VR + V
Sbjct  104    AVGIFNKTKQEDCLIANVYVPD--TDKKNLPVVVFVHGGAYLAGFGEVLTPKHMVRTNNV  161

Query  430    LVVTMNYRLGPYGFLCLDVPTASGNQGLRDQYKALSWIRNNIASFGGNPYNVTIAGQDAG  609
              + VT NYRLG  GFLCL   P A GN G++D     L W++ NIASFGGNP +VTI G  AG
Sbjct  162    IYVTFNYRLGVQGFLCLGTPDAPGNAGMKDMVALLRWVKKNIASFGGNPDDVTIDGYSAG  221

Query  610    ATSVLLQLYSDNEK-LFHKVIIESGTPQSE-GMFVNADVEAAIKLAVHLGLNTTETEEAI  783
              +++V L L S    K LFHK I ESG   +    + V+    A          G     +  A+
Sbjct  222    SSAVDLLLLSKATKGLFHKAIPESGANVAMWSVQVDPIANAKEYAKSEHGFEILDDIYAL  281

Query  784    QFLTKTSP-DLVVAAAKELELQLK------PCRERSFSGISNFVESDPYSLTNKKKVRNT  942
              +    K+ P D +  +  EL  + K        PC ER  +G+   F++   P ++   K
Sbjct  282    EDFYKSLPYDEMYKSGMELMFKNKNNFLFSPCVERD-TGVEKFLDDTPVNILKKGDYNKV  340

Query  943    PIMIGNTNKE---KKSLSADYFST---DPFYEKLKSNFNL-DEAQIEKAAKNVRHFYIGD  1101
              P++ G  N E    +  +  +YF +    D F E L  +    DE +  K A  V+ FY GD
Sbjct  341    PVLYGFANMEGLFRVGMGYEYFMSQLNDKFSENLPHDLEFKDEEEKRKVADEVKKFYFGD  400


> ta_transcript84816_1
Length=947

 Score =  162 bits (410),  Expect = 1e-41, Method: Compositional matrix adjust.
 Identities = 123/360 (34%), Positives = 176/360 (49%), Gaps = 26/360 (7%)
 Frame = +1

Query  91     QGLVRGHKAADGDYSIFLGIPYAKVDVNNPFSTATDPIAFPEIIHDAVDE-VRCPQTS-S  264
              QG +RG+K  +G    F  IPYA           A  P        +AVD  V C Q   +
Sbjct  44     QGKLRGYKDPEGGLFTFYNIPYATAPTGKDRFKAPLPAPVWITTREAVDRGVVCHQIDLA  103

Query  265    SVG----EQTLNCLRLNIYVPSAASSRNPMPVLVWIHGGDFATGSASDYGVKNIVR-HGV  429
              +VG      +   +CL  N+YVP    + +   +PV+V++HGG +  G        K++VR + V
Sbjct  104    AVGIFNKTKQEDCLIANVYVPD--TDKKNLPVVVFVHGGAYLAGFGEVLTPKHMVRTNNV  161

Query  430    LVVTMNYRLGPYGFLCLDVPTASGNQGLRDQYKALSWIRNNIASFGGNPYNVTIAGQDAG  609
              + VT NYRLG  GFLCL   P A GN G++D     L W++ NIASFGGNP +VTI G  AG
Sbjct  162    IYVTFNYRLGVQGFLCLGTPDAPGNAGMKDMVALLRWVKKNIASFGGNPDDVTIDGYSAG  221

Query  610    ATSVLLQLYSDNEK-LFHKVIIESGTPQSE-GMFVNADVEAAIKLAVHLGLNTTETEEAI  783
              +++V L L S    K LFHK I ESG   +    + V+    A          G     +  A+
Sbjct  222    SSAVDLLLLSKATKGLFHKAIPESGANVAMWSVQVDPIANAKEYAKSEHGFEILDDIYAL  281

Query  784    QFLTKTSP-DLVVAAAKELELQLK------PCRERSFSGISNFVESDPYSLTNKKKVRNT  942
              +    K+ P D +  +  EL  + K        PC ER  +G+   F++   P ++   K
Sbjct  282    EDFYKSLPYDEMYKSGMELMFKNKNNFLFSPCVERD-TGVEKFLDDTPVNILKKGDYNKV  340

Query  943    PIMIGNTNKE---KKSLSADYFST---DPFYEKLKSNFNL-DEAQIEKAAKNVRHFYIGD  1101
              P++ G  N E    +  +  +YF +    D F E L  +    DE +  K A  V+ FY GD
Sbjct  341    PVLYGFANMEGLFRVGMGYEYFMSQLNDKFSENLPHDLEFKDEEEKRKVADEVKKFYFGD  400

***************************************************************************************************
************************************************************************

Query= gi|512897630:468-1769 PREDICTED: Bombyx mori juvenile hormone esterase-like
(LOC101737052), mRNA

Length=1302
```

```
                                                                   Score     E
Sequences producing significant alignments:                       (Bits)  Value

  ta_transcript58915_1                                               192   9e-54
  ta_transcript58914_1                                               193   1e-53
  ta_transcript75783_1                                               189   4e-52
  ta_transcript48580_1                                               162   2e-42
  ta_transcript48579_1                                               162   3e-42
  ta_transcript29751_1                                               157   2e-41
  ta_transcript48393_1                                               151   4e-39
  ta_transcript84821_1                                               147   8e-38
  ta_transcript84818_1                                               147   2e-37
  ta_transcript84816_1                                               146   7e-37
  ta_transcript15565_1                                               135   3e-34


> ta_transcript58915_1
Length=606

 Score =   192 bits (489),  Expect = 9e-54, Method: Compositional matrix adjust.
 Identities = 147/453 (32%), Positives = 223/453 (49%), Gaps = 44/453 (10%)
 Frame = +1

Query  10    IPSNTSFPVMVFIHgggfgtgsgsptfygpQYLVSHGVVLVTVNYRLNAYGFLNLHTKDA  189
             I   +  PVMVFIHGGGF +GSG   FYG  +LV HGVVLVT+NYRL+A GFL L T++
Sbjct  120   ISPKSPLPVMVFIHGGGFKSGSGDEDFYGGDFLVHHGVVLVTINYRLDALGFLCLDTEEV  179

Query  190   PGNAGLKDIRAALRWIQKNIANFNGDPDNVTVFGQGTGGTAAIYMAFSDSTQGLFHKIIS  369
             PGNAGLKD  AAL+W+QKNI  F GDP NVT+FG+  GG++      S  ++GLF + I
Sbjct  180   PGNAGLKDQVAALKWVQKNIFAFGGDPTNVTIFGESAGGSSTALHVLSPLSKGLFKRAIP  239

Query  370   ESGTPFAPQSFDPDPLATAKQVARSLELITEEPSKLFKLYNDAPVVKLEEA----IGMQM  537
              SG PF   S   +P   A  + + L   TE+P  L +      P  K  +     +G +
Sbjct  240   MSGVPFCDWSIPFEPRKRAFTLGKILGFETEDPKALLEYLQSLPAEKFVDTNPTIMGFEE  299

Query  538   NSRSV-----FVPSVETVFDDEEPFLIDTPYNILKKKKFQSCPMIMG---MNTVEGLTST  693
              S ++     F P VE  F  +  F+ + P   LK+       +++G     T+ G+ S
Sbjct  300   KSYNMLKMYHFTPVVEKNF-GQHHFMTEEPLEALKQGHVNDVDVLIGNTDQETIVGIPSF  358

Query  694   MDFFTVTSQMNRIMNEDYSALDQRSMVVPKK--------EREEFREVLKETYFSNTTSDE  849
             +D   +  +   +              VP+K        +  E  E +++ YF +   D+
Sbjct  359   VDLLKMYDRYPEVF-------------VPRKILNKSTPGKILEIAERIRKHYFGDKPIDK  405

Query  850   ALIGGIINLNSDFSCVGPMSLFAEMY--ANSSSQPMYQYIFNYIGTRNL-GKLLTNSSLP  1020
               +   +   S+      ++ +  +       S  +Y+Y F+ +  RN+ GK      +
Sbjct  406   TSMKEAVTYFSEVCFTYDVNKYTNLLLSGKPGSSKVYRYRFSCVSERNIFGKYGQEYGIT  465

Query  1021  ATSNLDELFYIFELERMPLPLDENDA--RIITFMTMMWTNFAKTGCPTPDPENG-EWLPY  1191
               S+L++L Y+F+ + + LPLD N    ++I     ++TNFAK G PTPD   G  W  Y
Sbjct  466   GASHLEDLMYLFDAKSVKLPLDRNSKSYKMIQQTCALFTNFAKYGNPTPDSSLGFTWPEY  525

Query  1192  PHN----LAIALEPLYVAPLTSDRAHFWRTLYE  1278
                    + IA +      L +D   FW ++Y+
Sbjct  526   DIKDQSFVDIADQLTVGRHLDADAVKFWESIYQ  558


> ta_transcript58914_1
Length=650

 Score =   193 bits (490),  Expect = 1e-53, Method: Compositional matrix adjust.
 Identities = 147/453 (32%), Positives = 223/453 (49%), Gaps = 44/453 (10%)
 Frame = +1

Query  10    IPSNTSFPVMVFIHgggfgtgsgsptfygpQYLVSHGVVLVTVNYRLNAYGFLNLHTKDA  189
             I   +  PVMVFIHGGGF +GSG   FYG  +LV HGVVLVT+NYRL+A GFL L T++
Sbjct  120   ISPKSPLPVMVFIHGGGFKSGSGDEDFYGGDFLVHHGVVLVTINYRLDALGFLCLDTEEV  179

Query  190   PGNAGLKDIRAALRWIQKNIANFNGDPDNVTVFGQGTGGTAAIYMAFSDSTQGLFHKIIS  369
             PGNAGLKD  AAL+W+QKNI  F GDP NVT+FG+  GG++      S  ++GLF + I
Sbjct  180   PGNAGLKDQVAALKWVQKNIFAFGGDPTNVTIFGESAGGSSTALHVLSPLSKGLFKRAIP  239

Query  370   ESGTPFAPQSFDPDPLATAKQVARSLELITEEPSKLFKLYNDAPVVKLEEA----IGMQM  537
              SG PF   S   +P   A  + + L   TE+P  L +      P  K  +     +G +
Sbjct  240   MSGVPFCDWSIPFEPRKRAFTLGKILGFETEDPKALLEYLQSLPAEKFVDTNPTIMGFEE  299

Query  538   NSRSV-----FVPSVETVFDDEEPFLIDTPYNILKKKKFQSCPMIMG---MNTVEGLTST  693
              S ++     F P VE  F  +  F+ + P   LK+       +++G     T+ G+ S
```

```
Sbjct  300   KSYNMLKMYHFTPVVEKNF-GQHHFMTEEPLEALKQGHVNDVDVLIGNTDQETIVGIPSF  358

Query  694   MDFFTVTSQMNRIMNEDYSALDQRSMVVPKK--------EREEFREVLKETYFSNTTSDE  849
             +D   +  +   +              VP+K        +  E  E +++ YF +   D+
Sbjct  359   VDLLKMYDRYPEVF-------------VPRKILNKSTPGKILEIAERIRKHYFGDKPIDK  405

Query  850   ALIGGIINLNSDFSCVGPMSLFAEMY--ANSSSQPMYQYIFNYIGTRNL-GKLLTNSSLP  1020
               +   +   S+      ++ +  +       S  +Y+Y F+ +  RN+ GK      +
Sbjct  406   TSMKEAVTYFSEVCFTYDVNKYTNLLLSGKPGSSKVYRYRFSCVSERNIFGKYGQEYGIT  465

Query  1021  ATSNLDELFYIFELERMPLPLDENDA--RIITFMTMMWTNFAKTGCPTPDPENG-EWLPY  1191
               S+L++L Y+F+ + + LPLD N    ++I     ++TNFAK G PTPD   G  W  Y
Sbjct  466   GASHLEDLMYLFDAKSVKLPLDRNSKSYKMIQQTCALFTNFAKYGNPTPDSSLGFTWPEY  525

Query  1192  PHN----LAIALEPLYVAPLTSDRAHFWRTLYE  1278
                    + IA +      L +D   FW ++Y+
Sbjct  526   DIKDQSFVDIADQLTVGRHLDADAVKFWESIYQ  558


> ta_transcript75783_1
Length=733

 Score =   189 bits (480),  Expect = 4e-52, Method: Compositional matrix adjust.
 Identities = 150/451 (33%), Positives = 229/451 (51%), Gaps = 33/451 (7%)
 Frame = +1

Query  4     PKIPSNTSFPVMVFIHgggfgtgsgsptfygpQYLVSHGVVLVTVNYRLNAYGFLNLHTK  183
             P+I   T  PVMV+IHGGG+  GSG+   YGP++LV HGV+LVT NYRL   GFL+L T
Sbjct  211   PEIKPITLLPVMVWIHGGGYFWGSGNDDVYGPEFLVRHGVILVTFNYRLGVLGFLSLDTA  270

Query  184   DAPGNAGLKDIRAALRWIQKNIANFNGDPDNVTVFGQGTGGTAAIYMAFSDSTQGLFHKI  363
             + PGNAG+KD  AAL+W++ NI  F GDP+N+T+FG+  G  A      S  ++GLF +
Sbjct  271   EVPGNAGMKDQVAALKWVKSNIHFFGGDPENITIFGESAGAGAVSLHLLSPMSKGLFKRA  330

Query  364   ISESG--TPFAPQSFDPDPLATAKQVARSLELITEEPSKLFKLYNDAPV-----VKLEEA  522
             I +SG  T +  Q+F+    + A  +AR L L+T++  KL++ + +        + +
Sbjct  331   IIQSGSATAYWAQAFEIKEKSIA--LARKLGLVTDDDQKLYEFFGNISKELLINISVPVT  388

Query  523   IGMQMNSRSVFVPSVETVFDDEEPFLIDTPYNILKKKKFQSCPMIMGMNTVEGLTSTMDF  702
                Q      F P++E  F  +E FL D  Y IL++   +   ++MG    EG+
Sbjct  389   FAEQEEVNIEFAPTIEKDF-GQERFLTDDSYKILQQGIHKGVDVMMGYVEDEGVLYLNSL  447

Query  703   FTVTSQMNRIMNEDYSALDQRSMV--VPKKEREEFREVLKETYFSN---TTSDEALIGGI  867
               +  ++ RI N    AL  R+M   +P K++ +    +K+ YF N   T +D   I
Sbjct  448   EILDKEIERI-NNFLEALVPRNMKREIPIKQQLKVGRKMKKFYFDNKLVTRNDWRTIARY  506

Query  868   INLNSDFSCVGPMSLFAEMYANSSSQPMYQYIFNYIGTRN-LGKLLTNSSLP----ATSN  1032
             I+LN     V    +       + SQ  + Y F     RN   K+ T+  +        +
Sbjct  507   ISLN-----VFSYDVIQSQKNIAKSQNSFFYKFTCKSKRNFFKKVFTDDEITKDVDVVCH  561

Query  1033  LDELFYIFELERM--PLPLDENDARIITFMTMMWTNFAKTGCPTPDPENG-EWLPY----  1191
              D+L Y+F ++ M   +  D  + ++I  +T +WTNFAK G PTPD   G +W P+
Sbjct  562   CDDLAYLFPVKFMNQSVAKDSREFQLIDKVTKLWTNFAKYGNPTPDNSLGVKWEPFTLQH  621

Query  1192  PHNLAIALEPLYVAPLTSDRAHFWRTLYEKY  1284
              H L I  + +       +   FW  LY +Y
Sbjct  622   QHYLDIGEDLVLKTKPDDEDIQFWEELYREY  652


> ta_transcript48580_1
Length=834

 Score =   162 bits (410),  Expect = 2e-42, Method: Compositional matrix adjust.
 Identities = 138/455 (30%), Positives = 211/455 (46%), Gaps = 46/455 (10%)
 Frame = +1

Query  19    NTSFPVMVFIHgggfgtgsgsptfygpQYLVSHGVVLVTVNYRLNAYGFLNLHTKDAPGN  198
             N    VMV+IHGG F  GSG+   YGP +L+   +VLVT+NYRL A GFL++   +  GN
Sbjct  252   NPKLAVMVWIHGGAFAVGSGNAFLYGPDHLMGGNIVLVTLNYRLGALGFLSMENDEVSGN  311

Query  199   AGLKDIRAALRWIQKNIANFNGDPDNVTVFGQGTGGTAAIYMAFSDSTQGLFHKIISESG  378
              GLKD   AL+W++ NI  F GD   VT+FG+  G  +      S +++GLFH+ I++SG
Sbjct  312   MGLKDQVLALKWVRDNIEYFGGDASRVTIFGESAGAASVHLHMLSPASKGLFHRAIAQSG  371

Query  379   TPFAPQSFDPDPLATAKQVARSLELITEEPSKLFKLYNDAPV-------VKLEEAIGMQM  537
                +P +    P   A ++ R L + T   ++L       P         +L  A G  +
Sbjct  372   LALSPWALTRSPRERAFELGRELGIDTNSTAELLGYLRATPSELLVKAGARLAGAPGKSV  431
```

```
Query  538   NSRSV----FVPSVETVFDDEEPFLIDTPYNILKKKKFQSCPMIMGMNTVEGLTSTMDFF  705
             +  S     FVP+VE   D  + FL   P N++        P++ G N  EG+      F
Sbjct  432   DLHSTVALPFVPTVEP--DVPDAFLTQHPRNLMPG---ADVPLLTGYNAQEGII----LF  482

Query  706   TVTSQMNRIMNEDYSALDQ--RSMVVP------KKEREEFREVLKETYFSNTTSDEALIG  861
                 +  +++ E    LD+  R +V P      + +     + ++  YF     D   I
Sbjct  483   RRLQRYPKLLTE----LDREFRRVVPPELLTGDEAQNRRVADHIRAFYFQQRPVDIRNID  538

Query  862   GIINLNSDFSCVGPM--SLFAEMYANSSSQPMYQYIFNYIGTRNLGKLLTNSSLPATSNL  1035
              +I+L +D   + P+  +L  +   N +S P Y Y F + G   L K +   + P   +
Sbjct  539   SLIDLFTDVMFLRPLLETLRIQGKTNRTS-PTYLYRFAFDGALGLFKRMLGINHPGACHG  597

Query  1036  DELFYIFELERMPLPLDENDARIITF--MTMMWTNFAKTGCPT----PDPENGEWLPY--  1191
             DE+ Y+F    R+   LD++    +     M  MWTNFAKTG PT     DP + +W P
Sbjct  598   DEMGYLFYFSRLNYRLDDDSTELAVSKGMVRMWTNFAKTGNPTIGDHDDPIDFKWSPVND  657

Query  1192  -PHNLAIALEPLYVAPL--TSDRAHFWRTLYEKYG  1287
               H   + +   +       + R   W  LYE Y
Sbjct  658   TTHLTYLDINGHFTQKQDPEARRVRLWDWLYENYA  692
```

```
> ta_transcript48579_1
Length=905

 Score =   162 bits (410),  Expect = 3e-42, Method: Compositional matrix adjust.
 Identities = 138/455 (30%), Positives = 211/455 (46%), Gaps = 46/455 (10%)
 Frame = +1

Query  19    NTSFPVMVFIHgggfgtgsgsptfygpQYLVSHGVVLVTVNYRLNAYGFLNLHTKDAPGN  198
             N    VMV+IHGG F  GSG+    YGP +L+    +VLVT+NYRL A GFL++    +  GN
Sbjct  252   NPKLAVMVWIHGGAFAVGSGNAFLYGPDHLMGGNIVLVTLNYRLGALGFLSMENDEVSGN  311

Query  199   AGLKDIRAALRWIQKNIANFNGDPDNVTVFGQGTGGTAAIYMAFSDSTQGLFHKIISESG  378
              GLKD   AL+W++ NI   F GD    VT+FG+  G   +       S +++GLFH+ I++SG
Sbjct  312   MGLKDQVLALKWVRDNIEYFGGDASRVTIFGESAGAASVHLHMLSPASKGLFHRAIAQSG  371

Query  379   TPFAPQSFDPDPLATAKQVARSLELITEEPSKLFKLYNDAPV-------VKLEEAIGMQM  537
                +P +     P   A ++ R L + T    ++L       P         +L  A G  +
Sbjct  372   LALSPWALTRSPRERAFELGRELGIDTNSTAELLGYLRATPSELLVKAGARLAGAPGKSV  431

Query  538   NSRSV----FVPSVETVFDDEEPFLIDTPYNILKKKKFQSCPMIMGMNTVEGLTSTMDFF  705
             +  S     FVP+VE   D  + FL   P N++        P++ G N  EG+      F
Sbjct  432   DLHSTVALPFVPTVEP--DVPDAFLTQHPRNLMPG---ADVPLLTGYNAQEGII----LF  482

Query  706   TVTSQMNRIMNEDYSALDQ--RSMVVP------KKEREEFREVLKETYFSNTTSDEALIG  861
                 +  +++ E    LD+  R +V P      + +     + ++  YF     D   I
Sbjct  483   RRLQRYPKLLTE----LDREFRRVVPPELLTGDEAQNRRVADHIRAFYFQQRPVDIRNID  538

Query  862   GIINLNSDFSCVGPM--SLFAEMYANSSSQPMYQYIFNYIGTRNLGKLLTNSSLPATSNL  1035
              +I+L +D   + P+  +L  +   N +S P Y Y F + G   L K +   + P   +
Sbjct  539   SLIDLFTDVMFLRPLLETLRIQGKTNRTS-PTYLYRFAFDGALGLFKRMLGINHPGACHG  597

Query  1036  DELFYIFELERMPLPLDENDARIITF--MTMMWTNFAKTGCPT----PDPENGEWLPY--  1191
             DE+ Y+F    R+   LD++    +     M  MWTNFAKTG PT     DP + +W P
Sbjct  598   DEMGYLFYFSRLNYRLDDDSTELAVSKGMVRMWTNFAKTGNPTIGDHDDPIDFKWSPVND  657

Query  1192  -PHNLAIALEPLYVAPL--TSDRAHFWRTLYEKYG  1287
               H   + +   +       + R   W  LYE Y
Sbjct  658   TTHLTYLDINGHFTQKQDPEARRVRLWDWLYENYA  692
```

```
> ta_transcript29751_1
Length=506

 Score =   157 bits (396),  Expect = 2e-41, Method: Compositional matrix adjust.
 Identities = 124/358 (35%), Positives = 181/358 (51%), Gaps = 22/358 (6%)
 Frame = +1

Query  121   VVLVTVNYRLNAYGFLNLHTKDAPGNAGLKDIRAALRWIQKNIANFNGDPDNVTVFGQGT  300
             ++ VT NYRL   +GFL L T D PGNAG+KD+ A LRW++KNIA+F G+PD+VT+ G +
Sbjct  151   LIYVTFNYRLGVHGFLCLGTADVPGNAGMKDMVALLRWVKKNIASFGGNPDDVTIDGHSS  210

Query  301   GGTAAIYMAFSDSTQGLFHKIISESGTPFAPQSFDPDPLATAKQVARSLELITEEPSKLF  480
             G ++    +  S +T+GLFHK I ESG+  A  S   DP+A AK+ AR  E   E    ++
Sbjct  211   GSSSVDLLLLSKATEGLFHKAIPESGSNVAMWSVQIDPIAHAKEFARD-EHSFESGDDIY  269
```

```
Query  481   KL---YNDAPV-VKLEEAIGMQMNSRS---VFVPSVETVFDDEEPFLIDTPYNILKKKKF  639
              L   Y   P  V  +  + ++ NS     +F P VE      E FL D P NILK   +
Sbjct  270   ALEDFYKTLPFDVMYKSGMSLRYNSSVKNILFQPCVERD-TGVEKFLDDFPVNILKSGDY  328

Query  640   QSCPMIMGMNTVEGLTSTMDFFTVTSQMNRIMNEDYSA-LDQRSMVVPKKEREEFREVLK  816
                PM+ G   +EGL    DF    S M+ I NE +S  L        K+E+E+    +K
Sbjct  329   NKVPMLYGFTNMEGLMRVSDFVNYRSFMSDI-NEKFSDYLPNDLQFRDKEEKEKVARDVK  387

Query  817   ETYFSNTTSDEALIGGIINLNSD--FSCVGPMSLFAEMYANSSSQPMYQYIFNYIGTRNL  990
             + YF +   +   I   I+  SD  F+     SL  ++ A ++   +YQ+ + +
Sbjct  388   QFYFGDKVINNDTIFEYIDYFSDNMFAYPTLRSLKLQVEAGNNQVYLYQFSYPFENQAPE  447

Query  991   GKLLTNSSLPATSNLDELFYIFELERMPLPLDENDARIITFMT-MMWTNFAKTGCPTP  1161
             G +L    L  +S+  +   + +        +END  II  +T   W NF  TG P P
Sbjct  448   GVML---KLEGSSHTAQTHVVLDTLN-----EENDLAIIRNITRQFWANFITTGTPVP  497


> ta_transcript48393_1
Length=666

 Score =   151 bits (381),  Expect = 4e-39, Method: Compositional matrix adjust.
 Identities = 116/323 (36%), Positives = 161/323 (50%), Gaps = 13/323 (4%)
 Frame = +1

Query  22   TSFPVMVFIHgggfgtgsgsptfygpQYLVSHGVVLVTVNYRLNAYGFLNLHTKDAPGNA  201
            T  PV+V++HGG F  G G+        + S  ++ VT NYRL A+GFL L T+DAPGNA
Sbjct  183  TKLPVIVYVHGGAFQVGFGNLMTPK-NLVRSKNIIAVTFNYRLGAHGFLCLGTEDAPGNA  241

Query  202  GLKDIRAALRWIQKNIANFNGDPDNVTVFGQGTGGTAAIYMAFSDSTQGLFHKIISESGT  381
            GLKD  AALRW+ KNI    G+PD+VT+ G   G  +   MA S S +GLF KII ESG+
Sbjct  242  GLKDQVAALRWVNKNIIKLGGNPDDVTIAGYSAGAISVDLMAISKSAEGLFSKIIIESGS  301

Query  382  PFAPQSFDPDPLATAKQVARSLELIT-EEPSKLFKLYNDAPVVKLEEAIGMQM-NSRSVF  555
                 S   DPL  AK  A  L     ++   L K Y  A    L +   M    NS +F
Sbjct  302  SLFAGSIVTDPLENAKIFAARLGFNNIDDVYALEKFYKAASYDSLTKGEFMDRPNSTLLF  361

Query  556  VPSVETVFDDEEPFLIDTPYNILKKKKFQSCPMIMGMNTVEGLTST--MDFFTVTSQMNR  729
             P VE   DD   FL + P NILK+  +   PMI G + +EGL      +F+ +
Sbjct  362  SPCVERD-DDGLAFLSEPPINILKEGSYTQVPMIYGFSNMEGLFREPFFEFWKLK-----  415

Query  730  IMNEDYSALDQRSM-VVPKKEREEFREVLKETYFSNTTSDEALIGGIINLNSDFSCVGPM  906
             MNE +S      +    K +RE   + +K+ YF ++  +   I   I+   D     P
Sbjct  416  -MNEQFSEFIPGDLHFESKHDRERIAKRIKKFYFWDSPINGENILKYIDYFGDVLVKYPA  474

Query  907  SLFAEMYANSSSQPMYQYIFNYI  975
                +M+  +    +Y Y ++Y+
Sbjct  475  LRTVKMHIQNGHDKLYLYEYSYV  497


> ta_transcript84821_1
Length=611

 Score =   147 bits (370),  Expect = 8e-38, Method: Compositional matrix adjust.
 Identities = 103/277 (37%), Positives = 153/277 (55%), Gaps = 13/277 (5%)
 Frame = +1

Query  25   SFPVMVFIHgggfgtgsgsptfygpQYLVSHGVVLVTVNYRLNAYGFLNLHTKDAPGNAG  204
            + PV+VF+HGG +  G G         + ++ V+ VT NYRL   GFL L T DAPGNAG
Sbjct  130  NLPVVVFVHGGAYLAGFGEVLTPK-HMVRTNNVIYVTFNYRLGVQGFLCLGTPDAPGNAG  188

Query  205  LKDIRAALRWIQKNIANFNGDPDNVTVFGQGTGGTAAIYMAFSDSTQGLFHKIISESGTP  384
            +KD+ A LRW++KNIA+F G+PD+VT+ G   G +A   +  S +T+GLFHK I ESG
Sbjct  189  MKDMVALLRWVKKNIASFGGNPDDVTIDGYSAGSSAVDLLLLSKATKGLFHKAIPESGAN  248

Query  385  FAPQSFDPDPLATAKQVARSLE--LITEEPSKLFKLYNDAPVVKLEEAIGMQMNSRS---  549
             A  S   DP+A AK+ A+S     I ++   L   Y   P  ++ ++ GM++  ++
Sbjct  249  VAMWSVQVDPIANAKEYAKSEHGFEILDDIYALEDFYKSLPYDEMYKS-GMELMFKNKNN  307

Query  550  -VFVPSVETVFDDEEPFLIDTPYNILKKKKFQSCPMIMGMNTVEGLTST-MDFFTVTSQM  723
             +F P VE      E FL DTP NILKK  +   P++ G   +EGL    M +    SQ+
Sbjct  308  FLFSPCVERD-TGVEKFLDDTPVNILKKGDYNKVPVLYGFANMEGLFRVGMGYEYFMSQL  366

Query  724  NRIMNEDYSALDQRSMVVPKKEREEFREVLKETYFSN  834
            N   +E+   L        ++E+ +  + +K+ YF +
Sbjct  367  NDKFSEN---LPHDLEFKDEEEKRKVADEVKKFYFGD  400
```

```
> ta_transcript84818_1
Length=733

 Score =   147 bits (370),  Expect = 2e-37, Method: Compositional matrix adjust.
 Identities = 103/277 (37%), Positives = 153/277 (55%), Gaps = 13/277 (5%)
 Frame = +1

Query  25   SFPVMVFIHgggfgtgsgsptfygpQYLVSHGVVLVTVNYRLNAYGFLNLHTKDAPGNAG  204
            + PV+VF+HGG +  G G         + ++ V+ VT NYRL   GFL L T DAPGNAG
Sbjct  130  NLPVVVFVHGGAYLAGFGEVLTPK-HMVRTNNVIYVTFNYRLGVQGFLCLGTPDAPGNAG  188

Query  205  LKDIRAALRWIQKNIANFNGDPDNVTVFGQGTGGTAAIYMAFSDSTQGLFHKIISESGTP  384
            +KD+ A LRW++KNIA+F G+PD+VT+ G   G +A   +  S +T+GLFHK I ESG
Sbjct  189  MKDMVALLRWVKKNIASFGGNPDDVTIDGYSAGSSAVDLLLLSKATKGLFHKAIPESGAN  248

Query  385  FAPQSFDPDPLATAKQVARSLE--LITEEPSKLFKLYNDAPVVKLEEAIGMQMNSRS---  549
             A  S   DP+A AK+ A+S      I ++   L   Y   P  ++ ++ GM++  ++
Sbjct  249  VAMWSVQVDPIANAKEYAKSEHGFEILDDIYALEDFYKSLPYDEMYKS-GMELMFKNKNN  307

Query  550  -VFVPSVETVFDDEEPFLIDTPYNILKKKKFQSCPMIMGMNTVEGLTST-MDFFTVTSQM  723
             +F P VE      E FL DTP NILKK  +   P++ G   +EGL    M +    SQ+
Sbjct  308  FLFSPCVERD-TGVEKFLDDTPVNILKKGDYNKVPVLYGFANMEGLFRVGMGYEYFMSQL  366

Query  724  NRIMNEDYSALDQRSMVVPKKEREEFREVLKETYFSN  834
            N   +E+   L        ++E+ +  + +K+ YF +
Sbjct  367  NDKFSEN---LPHDLEFKDEEEKRKVADEVKKFYFGD  400


> ta_transcript84816_1
Length=947

 Score =   146 bits (369),  Expect = 7e-37, Method: Compositional matrix adjust.
 Identities = 103/277 (37%), Positives = 153/277 (55%), Gaps = 13/277 (5%)
 Frame = +1

Query  25   SFPVMVFIHgggfgtgsgsptfygpQYLVSHGVVLVTVNYRLNAYGFLNLHTKDAPGNAG  204
            + PV+VF+HGG +  G G         + ++ V+ VT NYRL   GFL L T DAPGNAG
Sbjct  130  NLPVVVFVHGGAYLAGFGEVLTPK-HMVRTNNVIYVTFNYRLGVQGFLCLGTPDAPGNAG  188

Query  205  LKDIRAALRWIQKNIANFNGDPDNVTVFGQGTGGTAAIYMAFSDSTQGLFHKIISESGTP  384
            +KD+ A LRW++KNIA+F G+PD+VT+ G   G +A   +  S +T+GLFHK I ESG
Sbjct  189  MKDMVALLRWVKKNIASFGGNPDDVTIDGYSAGSSAVDLLLLSKATKGLFHKAIPESGAN  248

Query  385  FAPQSFDPDPLATAKQVARSLE--LITEEPSKLFKLYNDAPVVKLEEAIGMQMNSRS---  549
             A  S   DP+A AK+ A+S      I ++   L   Y   P  ++ ++ GM++  ++
Sbjct  249  VAMWSVQVDPIANAKEYAKSEHGFEILDDIYALEDFYKSLPYDEMYKS-GMELMFKNKNN  307

Query  550  -VFVPSVETVFDDEEPFLIDTPYNILKKKKFQSCPMIMGMNTVEGLTST-MDFFTVTSQM  723
             +F P VE      E FL DTP NILKK  +   P++ G   +EGL    M +    SQ+
Sbjct  308  FLFSPCVERD-TGVEKFLDDTPVNILKKGDYNKVPVLYGFANMEGLFRVGMGYEYFMSQL  366

Query  724  NRIMNEDYSALDQRSMVVPKKEREEFREVLKETYFSN  834
            N   +E+   L        ++E+ +  + +K+ YF +
Sbjct  367  NDKFSEN---LPHDLEFKDEEEKRKVADEVKKFYFGD  400


> ta_transcript15565_1
Length=424

 Score =   135 bits (339),  Expect = 3e-34, Method: Compositional matrix adjust.
 Identities = 117/397 (29%), Positives = 177/397 (45%), Gaps = 63/397 (16%)
 Frame = +1

Query  202   GLKDIRAALRWIQKNIANFNGDPDNVTVFGQGTGGTAAIYMAFSDSTQGLFHKIISESGT  381
             G+KD+ A LRW+ +NIA F G+P++VT+ G   G  +   +  S + +GLFHK I ESG+
Sbjct  1     GMKDMVALLRWVNRNIAAFGGNPNDVTLEGWSAGSVSVELLMLSKAAKGLFHKAIPESGS  60

Query  382   PFAPQSFDPDPLATAKQVARSLELI--TEEPSKLFKLYNDAPVVKL-EEAIGMQMNSRSV  552
              FAP S   DP+  AK  ARS        +  S L + Y   P   + ++   +
Sbjct  61    SFAPFSVGVDPIGNAKDFARSDLGFKDVDNISALEEFYKTVPFKDMYQQGPLIIFRHNFT  120

Query  553   FVPSVETVFDDEEPFLIDTPYNILKKKKFQSCPMIMGMNTVEGLTST---MDFFTVTSQM  723
             F P VE     ++ FL DTPYNILKK  +   PM+ G +  EGL         FF     M
Sbjct  121   FTPCVERNI-GQDVFLDDTPYNILKKGDYMKVPMLYGFSNKEGLYRVDYGYQFF--IEGM  177

Query  724   NRIMNEDYSALDQRSMVVPKKEREEFREVLKETYFSNTTSDEALIGGIINLNSDFSCVGP  903
             N+  + DY   D +     + E+E+   ++KE YF N   D+  +   I+  +D    GP
```

```
Sbjct  178   NKKFS-DYLPNDLQ--FKDENEKEQVATMIKEFYFGNKKIDQDTVLEYIDYFTDSMFAGP  234

Query  904   MSLFAEMYANSSSQPMYQYIFNY--------------IGTRNLGKLLT-------NSSLP  1020
                  +++  + +  +Y Y F+Y              IG+ + G+ +T        + +P
Sbjct  235   SLKEVQLHVAAGNNQIYLYQFSYPYENKVPESIKVKVIGSNHCGETMTVLDNWLGGAEMP  294

Query  1021  ATSNLDELFYIFELERMPLPLDENDARIITFMTMMWTNFAKTGCPTPDPEN--------G  1176
             A  N D       LE++         R +T    +W NF KTG P P   N        G
Sbjct  295   AERNSD-------LEQV---------RNVT--RQIWGNFIKTGKPVPQGSNLPTWPATDG  336

Query  1177  EWLPYPHNLAIALEP-LYVAPLTSDRAHFWRTLYEKY  1284
                PY   + I  +P L   P       FW  +Y+++
Sbjct  337   SGSPY---MVITNKPELKGQPFQPMSVMFWTEIYQRH  370
```

****
****

Query= gi|512897844:207-1901 PREDICTED: Bombyx mori juvenile hormone esterase-like (LOC101746607), transcript variant X2, mRNA

```
Length=1695
                                                                      Score     E
Sequences producing significant alignments:                          (Bits)  Value

  ta_transcript58915_1                                                  273   5e-82
  ta_transcript58914_1                                                  272   3e-81
  ta_transcript75783_1                                                  261   1e-76
  ta_transcript74072_1                                                  249   3e-71
  ta_transcript74071_1                                                  248   1e-70
  ta_transcript84821_1                                                  216   8e-61
  ta_transcript20178_1                                                  216   8e-61
  ta_transcript29751_1                                                  212   2e-60
  ta_transcript84818_1                                                  214   9e-60
  ta_transcript84816_1                                                  214   4e-59
  ta_transcript48580_1                                                  211   4e-58
  ta_transcript48579_1                                                  211   4e-58
  ta_transcript48393_1                                                  197   4e-54
  ta_transcript15565_1                                                  159   6e-42
  ta_transcript49309_1                                                  133   3e-32

> ta_transcript58915_1
Length=606

 Score =   273 bits (697),  Expect = 5e-82, Method: Compositional matrix adjust.
 Identities = 197/541 (36%), Positives = 289/541 (53%), Gaps = 39/541 (7%)
 Frame = +1

Query  115   ATVRVSNGLLRG----AVAHDGSYVQYNGIPFGSV-TDETKFQAPGPEPKWDGIYDAIHE  279
             A V+V  G L G    +V+ DG +  + GIP+ +    + +F+AP P   W G+  A
Sbjct  30    AKVQVEQGWLEGEQLDSVSGDGQFFSFKGIPYAAPPVGKLRFKAPQPPVSWQGVRKATQH  89

Query  280   EIRCNQQ--FSTNLTIGRRDCLTLNIYTPFNTDPGDKLVVMffihgggffqgsgssffyg  453
               +C Q+  FS  +  G  DCL LN+YT  +  P   L VM FIHGGGF  GSG   FYG
Sbjct  90    GPKCPQRDIFSKQIIPGSEDCLYLNVYTK-DISPKSPLPVMVFIHGGGFKSGSGDEDFYG  148

Query  454   PRYLVSKGVILVTINYRLNIQGFLCLGIKEAPGNAALKDMVAALKWVQRNIASFGGDPDN  633
               +LV  GV+LVTINYRL+  GFLCL  +E PGNA LKD VAALKWVQ+NI +FGGDP N
Sbjct  149   GDFLVHHGVVLVTINYRLDALGFLCLDTEEVPGNAGLKDQVAALKWVQKNIFAFGGDPTN  208

Query  634   VTIFGESAGAASVSFLVLSPMAKGLFHKAITQSGSSLAPFGLQFKPIFLASLLAKVMGFI  813
             VTIFGESAG +S +  VLSP++KGLF +AI  SG     + + F+P   A  L K++GF
Sbjct  209   VTIFGESAGGSSTALHVLSPLSKGLFKRAIPMSGVPFCDWSIPFEPRKRAFTLGKILGFE  268

Query  814   SQDPVKLYEYFMTKSDDELILTRVP----RKEGHVIISEILYTPCVEKVIEGVEPFLIQS  981
             ++DP  L EY  +   ++ + T        ++ + ++    +TP VEK   G   F+ +
Sbjct  269   TEDPKALLEYLQSLPAEKFVDTNPTIMGFEEKSYNMLKMYHFTPVVEKNF-GQHHFMTEE  327

Query  982   PQEALSKGEFNKVPMIIGSNTQEGIMILEMDNSTMRNNIKIEKSLP------KNLNITSQ  1143
             P EAL +G  N V ++IG+  QE I+ +     +  + +K+    P      K LN ++
Sbjct  328   PLEALKQGHVNDVDVLIGNTDQETIVGI----PSFVDLLKMYDRYPEVFVPRKILNKSTP  383

Query  1144  EAVTEIVGNFKKIYMGDGDSFGDLVSLSK---WYGEPYLNYPSLEETELIL--KSSKQPI  1308
               + EI    +K Y GD     D  S+ +   ++ E    Y   + T L+L  K     +
Sbjct  384   GKILEIAERIRKHYFGDKPI--DKTSMKEAVTYFSEVCFTYDVNKYTNLLLSGKPGSSKV  441
```

```
Query  1309  YNYLFNYSGWRNLPKLGGGSAYKDVPGATHADDLFYMFSQEWLSGFFEN-----KMIERM  1473
             Y Y F+    RN+   G       + GA+H +DL Y+F  + +    +      KMI++
Sbjct  442   YRYRFSCVSERNI--FGKYGQEYGITGASHLEDLMYLFDAKSVKLPLDRNSKSYKMIQQT  499

Query  1474  TTLWTNFAKYGDPTPEATDLLPVKWYPTNLTTPMNFVLDEAFSTVPLWYNDAMKYWKQVY  1653
               L+TNFAKYG+PTP+++  L   W   ++       + +  +       DA+K+W+ +Y
Sbjct  500   CALFTNFAKYGNPTPDSS--LGFTWPEYDIKDQSFVDIADQLTVGRHLDADAVKFWESIY  557

Query  1654  Q  1656
             Q
Sbjct  558   Q  558


> ta_transcript58914_1
Length=650

 Score =   272 bits (695),  Expect = 3e-81, Method: Compositional matrix adjust.
 Identities = 197/541 (36%), Positives = 289/541 (53%), Gaps = 39/541 (7%)
 Frame = +1

Query  115   ATVRVSNGLLRG----AVAHDGSYVQYNGIPFGSV-TDETKFQAPGPEPKWDGIYDAIHE  279
             A V+V  G L G    +V+ DG +  + GIP+ +    + +F+AP P   W G+  A
Sbjct  30    AKVQVEQGWLEGEQLDSVSGDGQFFSFKGIPYAAPPVGKLRFKAPQPPVSWQGVRKATQH  89

Query  280   EIRCNQQ--FSTNLTIGRRDCLTLNIYTPFNTDPGDKLVVMffihgggffqgsgssffyg  453
               +C Q+  FS  +  G  DCL LN+YT  +  P   L VM FIHGGGF  GSG   FYG
Sbjct  90    GPKCPQRDIFSKQIIPGSEDCLYLNVYTK-DISPKSPLPVMVFIHGGGFKSGSGDEDFYG  148

Query  454   PRYLVSKGVILVTINYRLNIQGFLCLGIKEAPGNAALKDMVAALKWVQRNIASFGGDPDN  633
               +LV  GV+LVTINYRL+  GFLCL  +E PGNA LKD VAALKWVQ+NI +FGGDP N
Sbjct  149   GDFLVHHGVVLVTINYRLDALGFLCLDTEEVPGNAGLKDQVAALKWVQKNIFAFGGDPTN  208

Query  634   VTIFGESAGAASVSFLVLSPMAKGLFHKAITQSGSSLAPFGLQFKPIFLASLLAKVMGFI  813
             VTIFGESAG +S +  VLSP++KGLF +AI  SG     + + F+P   A  L K++GF
Sbjct  209   VTIFGESAGGSSTALHVLSPLSKGLFKRAIPMSGVPFCDWSIPFEPRKRAFTLGKILGFE  268

Query  814   SQDPVKLYEYFMTKSDDELILTRVP----RKEGHVIISEILYTPCVEKVIEGVEPFLIQS  981
             ++DP  L EY  +   ++ + T        ++ + ++    +TP VEK   G   F+ +
Sbjct  269   TEDPKALLEYLQSLPAEKFVDTNPTIMGFEEKSYNMLKMYHFTPVVEKNF-GQHHFMTEE  327

Query  982   PQEALSKGEFNKVPMIIGSNTQEGIMILEMDNSTMRNNIKIEKSLP------KNLNITSQ  1143
             P EAL +G  N V ++IG+  QE I+ +     +  + +K+    P      K LN ++
Sbjct  328   PLEALKQGHVNDVDVLIGNTDQETIVGI----PSFVDLLKMYDRYPEVFVPRKILNKSTP  383

Query  1144  EAVTEIVGNFKKIYMGDGDSFGDLVSLSK---WYGEPYLNYPSLEETELIL--KSSKQPI  1308
               + EI    +K Y GD     D  S+ +   ++ E    Y   + T L+L  K     +
Sbjct  384   GKILEIAERIRKHYFGDKPI--DKTSMKEAVTYFSEVCFTYDVNKYTNLLLSGKPGSSKV  441

Query  1309  YNYLFNYSGWRNLPKLGGGSAYKDVPGATHADDLFYMFSQEWLSGFFEN-----KMIERM  1473
             Y Y F+    RN+   G       + GA+H +DL Y+F  + +    +      KMI++
Sbjct  442   YRYRFSCVSERNI--FGKYGQEYGITGASHLEDLMYLFDAKSVKLPLDRNSKSYKMIQQT  499

Query  1474  TTLWTNFAKYGDPTPEATDLLPVKWYPTNLTTPMNFVLDEAFSTVPLWYNDAMKYWKQVY  1653
               L+TNFAKYG+PTP+++  L   W   ++       + +  +       DA+K+W+ +Y
Sbjct  500   CALFTNFAKYGNPTPDSS--LGFTWPEYDIKDQSFVDIADQLTVGRHLDADAVKFWESIY  557

Query  1654  Q  1656
             Q
Sbjct  558   Q  558


> ta_transcript75783_1
Length=733

 Score =   261 bits (667),  Expect = 1e-76, Method: Compositional matrix adjust.
 Identities = 200/538 (37%), Positives = 285/538 (53%), Gaps = 33/538 (6%)
 Frame = +1

Query  118   TVRVSNGLLRGAVAHD---GSYVQYNGIPFGSV-TDETKFQAPGPEPKWDGIYDAIHEEI  285
             TV+VS G+L+G +  +   GSY  + GIPF      + +F+AP P   W G+  A
Sbjct  125   TVKVSEGILQGELVQNEFGGSYYSFKGIPFAEPPVGDLRFKAPIPAKPWKGVKTATKFG-  183

Query  286   RCNQQFST---NLTIGRRDCLTLNIYTPFNTDPGDKLVVMffihgggffqgsgssffygP  456
               N QF     +   G  DCL LN+YTP    P   L VM +IHGGG+F GSG+   YGP
Sbjct  184   PINYQFDVFKKDPPSGSEDCLYLNVYTP-EIKPITLLPVMVWIHGGGYFWGSGNDDVYGP  242

Query  457   RYLVSKGVILVTINYRLNIQGFLCLGIKEAPGNAALKDMVAALKWVQRNIASFGGDPDNV  636
```

```
            +LV  GVILVT NYRL + GFL L   E PGNA +KD VAALKWV+ NI  FGGDP+N+
Sbjct  243   EFLVRHGVILVTFNYRLGVLGFLSLDTAEVPGNAGMKDQVAALKWVVKSNIHFFGGDPENI  302

Query  637   TIFGESAGAASVSFLVLSPMAKGLFHKAITQSGSSLAPFGLQFKPIFLASLLAKVMGFIS  816
             TIFGESAGA +VS  +LSPM+KGLF +AI QSGS+ A +   F+    +  LA+ +G ++
Sbjct  303   TIFGESAGAGAVSLHLLSPMSKGLFKRAIIQSGSATAYWAQAFEIKEKSIALARKLGLVT  362

Query  817   QDPVKLYEYFMTKSDDELILTRVPRKEGHVIISEILYTPCVEKVIEGVEPFLIQSPQEAL  996
              D  KLYE+F   S + LI   VP          I + P +EK   G E FL     + L
Sbjct  363   DDDQKLYEFFGNISKELLINISVPVTFAEQEEVNIEFAPTIEKDF-GQERFLTDDSYKIL  421

Query  997   SKGEFNKVPMIIGSNTQEGIMILE----MDNSTMRNNIKIEKSLPKNLN----ITSQEAV  1152
              +G    V +++G    EG++ L     +D    R N  +E  +P+N+     I  Q  V
Sbjct  422   QQGIHKGVDVMMGYVEDEGVLYLNSLEILDKEIERINNFLEALVPRNMKREIPIKQQLKV  481

Query  1153  TEIVGNFKKIYMGDG-DSFGDLVSLSKWYGEPYLNYPSLEETELILKSSKQPIYNYLFNY  1329
                    KK Y  +   +  D  +++++      +Y  ++  + I KS  Q  + Y F
Sbjct  482   GR---KMKKFYFDNKLVTRNDWRTIARYISLNVFSYDVIQSQKNIAKS--QNSFFYKFTC  536

Query  1330  SGWRNLPK--LGGGSAYKDVPGATHADDLFYMFSQEWLSGFF-----ENKMIERMTTLWT  1488
                RN  K          KDV    H DDL Y+F  ++++         E ++I+++T LWT
Sbjct  537   KSKRNFFKKVFTDDEITKDVDVVCHCDDLAYLFPVKFMNQSVAKDSREFQLIDKVTKLWT  596

Query  1489  NFAKYGDPTPEATDLLPVKWYPTNLTTPMNFVLDEAFSTVPLWYNDAMKYWKQVYQKY  1662
             NFAKYG+PTP+ +   L VKW P  L       + E         ++ +++W+++Y++Y
Sbjct  597   NFAKYGNPTPDNS--LGVKWEPFTLQHQHYLDIGEDLVLKTKPDDEDIQFWEELYREY  652
```

```
> ta_transcript74072_1
Length=938

 Score =   249 bits (636),  Expect = 3e-71, Method: Compositional matrix adjust.
 Identities = 183/377 (49%), Positives = 216/377 (57%), Gaps = 1/377 (0%)
 Frame = -2

Query  1190  PMYIFLKFPTISVTASCEVIFKFFGSDFSILILFRIVLLSISKIMIPS*VLEPIIIGTLL  1011
             P   F K    S  +S + I   FG D S +IL  +V  S    +  PS +L PII+GTLL
Sbjct  226   PCKAFFKVFATSFLSSTDGITMSFGKDSSKVILSNVVSFSKFAMKCPSSLLLPIIMGTLL  285

Query  1010  NSPFESASCGDCIKNGSTPSITFSTHGVYKISDIITCPSFLGTLVNINSSSDFVMKYSYN  831
             N P    S G  +KNGS+PS++ S  G Y  SD    PSFLGTLVNI+SSS   +K
Sbjct  286   NFPLVRTS*GIYLKNGSSPSMSISAQGAYNTSDTFVLPSFLGTLVNISSSSVLFIKIL*I  345

Query  830   LTGS*LINPITLASKLARKIGLNCKPNGANEDPLCVIAL*NNPLAIGDKTKNETDAAPAL  651
               GS +  P  L  KLA   G N     A +DPL  IAL  +P+ IGDKT  +  A PAL
Sbjct  346   SCGSSVSYPKVLVIKLAIYTGRNWNAQAARDDPLIAIALWKSPVDIGDKT**DIAAPPAL  405

Query  650   SPNIVTLSGSPPKLAIFLCTHFKAATISFKAAFPGASLIPKQRNPCILSL*LIVTKITPL  471
             SP IVTLSGSPPK  +FL TH KAAT SF AAFPGAS + K R PC+ +L* IVTKI PL
Sbjct  406   SPKIVTLSGSPPKTLMFL*THLKAATWSFIAAFPGASFVLKHRKPCMFNL*FIVTKIIPL  465

Query  470   DTRYLGp*knedpdp*knppp*iknITTSLSPGSVLNGVYMFRVKQSRRPIVRLVENCWL  291
             DT+Y GP* N    +P K  PP*    +T +    G    GV     +KQS  P       C
Sbjct  466   DTKYFGP*INLAFEPAKKAPP*TYTMTGNFDSGLAP*GV*TLSIKQSS*PKTMFPSPC-R  524

Query  290   HLiss*ias*IPSHFGSGPGAWNLVSSVTLPNGIPLYCTYDPSCATAPRSSPLLTRTVAV  111
             HLI S +AS IPSHFG GPGA NL S +T+P GIP      PS  T PR SP   R  +V
Sbjct  525   HLIFSSMASKIPSHFGGGPGALNLYSELTVP*GIPR*EE*TPSADTYPRISPESMRNSSV  584

Query  110   G*RTSRAAHSDHSTNHF  60
             G RT+ AA   H+  HF
Sbjct  585   GCRTNLAAQRLHNITHF  601
```

```
> ta_transcript74071_1
Length=1143

 Score =   248 bits (634),  Expect = 1e-70, Method: Compositional matrix adjust.
 Identities = 183/378 (48%), Positives = 216/378 (57%), Gaps = 1/378 (0%)
 Frame = -2

Query  1190  PMYIFLKFPTISVTASCEVIFKFFGSDFSILILFRIVLLSISKIMIPS*VLEPIIIGTLL  1011
             P   F K    S  +S + I   FG D S +IL  +V  S    +  PS +L PII+GTLL
Sbjct  226   PCKAFFKVFATSFLSSTDGITMSFGKDSSKVILSNVVSFSKFAMKCPSSLLLPIIMGTLL  285

Query  1010  NSPFESASCGDCIKNGSTPSITFSTHGVYKISDIITCPSFLGTLVNINSSSDFVMKYSYN  831
             N P    S G  +KNGS+PS++ S  G Y  SD    PSFLGTLVNI+SSS   +K
```

```
Sbjct  286   NFPLVRTS*GIYLKNGSSPSMSISAQGAYNTSDTFVLPSFLGTLVNISSSSVLFIKIL*I  345

Query  830   LTGS*LINPITLASKLARKIGLNCKPNGANEDPLCVIAL*NNPLAIGDKTKNETDAAPAL  651
               GS +  P  L  KLA   G N     A +DPL  IAL  +P+ IGDKT  +  A PAL
Sbjct  346   SCGSSVSYPKVLVIKLAIYTGRNWNAQAARDDPLIAIALWKSPVDIGDKT**DIAAPPAL  405

Query  650   SPNIVTLSGSPPKLAIFLCTHFKAATISFKAAFPGASLIPKQRNPCILSL*LIVTKITPL  471
             SP IVTLSGSPPK  +FL TH KAAT SF AAFPGAS + K R PC+ +L* IVTKI PL
Sbjct  406   SPKIVTLSGSPPKTLMFL*THLKAATWSFIAAFPGASFVLKHRKPCMFNL*FIVTKIIPL  465

Query  470   DTRYLGp*knedpdp*knppp*iknITTSLSPGSVLNGVYMFRVKQSRRPIVRLVENCWL  291
             DT+Y GP* N   +P K  PP*   +T +   G    GV    +KQS  P       C
Sbjct  466   DTKYFGP*INLAFEPAKKAPP*TYTMTGNFDSGLAP*GV*TLSIKQSS*PKTMFPSPC-R  524

Query  290   HLiss*ias*IPSHFGSGPGAWNLVSSVTLPNGIPLYCTYDPSCATAPRSSPLLTRTVAV  111
             HLI S +AS IPSHFG GPGA NL S +T+P GIP      PS  T PR SP   R  +V
Sbjct  525   HLIFSSMASKIPSHFGGGPGALNLYSELTVP*GIPR*EE*TPSADTYPRISPESMRNSSV  584

Query  110   G*RTSRAAHSDHSTNHFA  57
             G RT+ AA   H+  HF
Sbjct  585   GCRTNLAAQRLHNITHFQ  602


> ta_transcript84821_1
Length=611

 Score =   216 bits (549),  Expect = 8e-61, Method: Compositional matrix adjust.
 Identities = 145/408 (36%), Positives = 216/408 (53%), Gaps = 39/408 (10%)
 Frame = +1

Query  13    CCIVNRNIVCVMCVRAKWLVLWSLWAARLVRQPTATVRVSNGLLRGAVAHDGSYVQYNGI  192
             CC++     C +              A+   + + TV +  G LRG    +G    +  I
Sbjct  18    CCVLAIGCACAL--------------AQPDDENSRTVEIQQGKLRGYKDPEGGLFTFYNI  63

Query  193   PFGSV-TDETKFQAPGPEPKWDGIYDAIHEEIRCNQQFSTNLTI----GRRDCLTLNIYT  357
             P+ +  T + +F+AP P P W    +A+   + C+Q     + I     + DCL  N+Y
Sbjct  64    PYATAPTGKDRFKAPLPAPVWITTREAVDRGVVCHQIDLAAVGIFNKTKQEDCLIANVYV  123

Query  358   PFNTDPGDKLVVMffihgggffqgsgssffygPRYLV-SKGVILVTINYRLNIQGFLCLG  534
             P +TD  +  VV+F   G              P+++V +  VI VT NYRL +QGFLCLG
Sbjct  124   P-DTDKKNLPVVVFVHGGAYLAGFGEVLT---PKHMVRTNNVIYVTFNYRLGVQGFLCLG  179

Query  535   IKEAPGNAALKDMVAALKWVQRNIASFGGDPDNVTIFGESAGAASVSFLVLSPMAKGLFH  714
               +APGNA +KDMVA L+WV++NIASFGG+PD+VTI G SAG+++V  L+LS   KGLFH
Sbjct  180   TPDAPGNAGMKDMVALLRWVKKNIASFGGNPDDVTIDGYSAGSSAVDLLLLSKATKGLFH  239

Query  715   KAITQSGSSLAPFGLQFKPIFLASLLAKV-MGF-ISQDPVKLYEYFMTKSDDELILTRVP  888
             KAI +SG+++A + +Q  PI  A   AK   GF I  D   L +++ +   DE+
Sbjct  240   KAIPESGANVAMWSVQVDPIANAKEYAKSEHGFEILDDIYALEDFYKSLPYDEMY-----  294

Query  889   RKEGHVII----SEILYTPCVEKVIEGVEPFLIQSPQEALSKGEFNKVPMIIGSNTQEGI  1056
              K G  ++    +  L++PCVE+   GVE FL  +P   L KG++NKVP++ G    EG+
Sbjct  295   -KSGMELMFKNKNNFLFSPCVERDT-GVEKFLDDTPVNILKKGDYNKVPVLYGFANMEGL  352

Query  1057  MILEMDNSTMRN--NIKIEKSLPKNLNITSQEAVTEIVGNFKKIYMGD  1194
               + M      +  N K  ++LP +L    +E   ++    KK Y GD
Sbjct  353   FRVGMGYEYFMSQLNDKFSENLPHDLEFKDEEEKRKVADEVKKFYFGD  400


> ta_transcript20178_1
Length=614

 Score =   216 bits (549),  Expect = 8e-61, Method: Compositional matrix adjust.
 Identities = 217/534 (41%), Positives = 268/534 (50%), Gaps = 13/534 (2%)
 Frame = -2

Query  1661  YF*YTCFQYFIASLYQSGTVENASSRTKFIGVVKLVGYHLTGRRSVASGVGSPYFAKFVH  1482
             YF*Y   Q      YQSG V     R    G +   G H       +SG GSPYFAKFV
Sbjct  57    YF*YISLQKRREGEYQSGCVCEVP-RISERGSILSAGSH-------SSGFGSPYFAKFVQ  108

Query  1481  KVVILSIILFSKNPDNHS*ENI*NKSSACVAPGTSLYADPPPNFGRFRQPE*lnk*l*ig  1302
              VVI+SIILFS         N+  +SSACVAPG++ + +P  N  +F  P *L K*L I
Sbjct  109   YVVIISIILFSNKEGAIHGLNMYMRSSACVAPGSNNHRNPD*NLTKFLHPL*LKK*LYIR  168

Query  1301  cllLFSINSVSSKEG*LR*GSPYHFDKLTKSPKLSPSPMYIFLKFPTISVTASCEVIFKF  1122
              LL+F   SV S  G +R  SP + +         PSP+  FL F      +      K
Sbjct  169   SLLVFKNRSVCSTTGYVRNKSPKYLE*PIILSATFPSPI*NFLIFRATVFLSLSFGKAKS  228
```

```
Query  1121  FGSDFSILILFRIVLLSISKIMIPS*VLEPIIIGTLLNSPFESASCGDCIKNGSTPSITF  942
              G  S  I    V  S      PS +L PI +GTL   P  +   G  + NG +PSITF
Sbjct  229   MGIATSKFIFCNSVAFSCEAK*YPSSLLCPINVGTLA*LPLSNML*GKSVMNGFSPSITF  288

Query  941   STHGVYKISDIITCPSFLGTLVNINSSSDFVMKYSYNLTGS*LINPITLASKLARKIGLN  762
             STHG+    ++   P   G L   +SS D + K   +  G  ++ P   A++LA  IG
Sbjct  289   STHGINITWEMYVPPIGFGILTAYSSSIDLLEKML*SSCGFLVL*PSASANRLAVSIGSG  348

Query  761   CKPNGANEDPLCVIAL*NNPLAIGDKTKNETDAAPALSPNIVTLSGSPPKLAIFLCTHFK  582
             C P G    PLC+IAL* +P  IG     +T+AAPALSP IVTLSGSPP   IFL TH +
Sbjct  349   CIPQGVTAVPLCIIAL*KSPFDIGASI*KDTEAAPALSPKIVTLSGSPPNALIFL*THLR  408

Query  581   AATISFKAAFPGASLIPKQRNPCILSL*LIVTKITPLDTRYLGp*knedpdp*knppp*i  402
             AAT SF    PG S +P+ +NP + +L*L VT    L TRY GP +N +PDP KNPP
Sbjct  409   AAT*SFNPILPGTSFVPRHKNPRMDNL*LNVTSTISLVTRYSGPCRNLEPDPNKNPPSWK  468

Query  401   knITTSLSPGSVLNGVYMFRVKQSRRPIVRLVENCWLHLiss*ias*IPSHFGSGPGAWN  222
               IT   +    L GV  F  +Q   P V +    W   I S IAS    H GS     N
Sbjct  469   YTITG--NDFLALTGV*TFMTRQFS*P-VTIFPIFW*QYICSSIASNTLFHLGSSLAT*N  525

Query  221   LVSSVTLPNGIPLYCTYDPSCATAPRSSPLLTRTVAVG*RTSRAAHSDHSTNHF  60
             L S+V    GIP      PS  T P ++P L RT++VG  T   A  D ST HF
Sbjct  526   LSSTVA--*GIPR*F*CVPSGDTDPLNAPELIRTISVGCLTILIAQRDQSTTHF  577


> ta_transcript29751_1
Length=506

 Score =   212 bits (540),  Expect = 2e-60, Method: Compositional matrix adjust.
 Identities = 169/509 (33%), Positives = 259/509 (51%), Gaps = 31/509 (6%)
 Frame = +1

Query  43    VMCVRAKWLVLWSLWAARLVRQPTATVRVSNGLLRGAVAHDGS-YVQYNGIPFGSV-TDE  216
             V+CV    ++L +L  A+   + + TV +   G +RG    DG  YV YN IP+ +  T +
Sbjct  13    VICVA---IILCALAHAQ---EDSRTVVIEQGPVRGYKDPDGGLYVFYN-IPYATAPTSK  65

Query  217   TKFQAPGPEPKWDGIYDAIHEEIRCNQQFSTNLTIG-RRDCLTLNIYTPFNTDPGDKLVV  393
              +F+AP   P W  I DA+  E+ C Q   T       + DCL  N++ P  ++    +VV
Sbjct  66    DRFKAPLAPPVWVTIRDAVDREVVCPQMLETGSHKNIQEDCLIANVFVPDTSEKNLSVVV  125

Query  394   MffihgggffqgsgssffygPRYLV-SKGVILVTINYRLNIQGFLCLGIKEAPGNAALKD  570
             +    G     G+  +     P+ +V  K +I VT NYRL + GFLCLG  + PGNA +KD
Sbjct  126   LVHGGGYLSGFGNRQT----PKDMVRDKKLIYVTFNYRLGVHGFLCLGTADVPGNAGMKD  181

Query  571   MVAALKWVQRNIASFGGDPDNVTIFGESAGAASVSFLVLSPMAKGLFHKAITQSGSSLAP  750
             MVA L+WV++NIASFGG+PD+VTI G S+G++SV  L+LS   +GLFHKAI +SGS++A
Sbjct  182   MVALLRWVKKNIASFGGNPDDVTIDGHSSGSSSVDLLLLSKATEGLFHKAIPESGSNVAM  241

Query  751   FGLQFKPIFLASLLAK-VMGFISQDPVKLYEYFMTKSDDELILTRVPRKEGHVIISEILY  927
             + +Q  PI  A   A+    F S D +   E F      +++          +  +  IL+
Sbjct  242   WSVQIDPIAHAKEFARDEHSFESGDDIYALEDFYKTLPFDVMYKSGMSLRYNSSVKNILF  301

Query  928   TPCVEKVIEGVEPFLIQSPQEALSKGEFNKVPMIIGSNTQEGIMILEMDNSTMRN-----  1092
              PCVE+   GVE FL   P   L  G++NKVPM+ G    EG+M +  D    R+
Sbjct  302   QPCVERDT-GVEKFLDDFPVNILKSGDYNKVPMLYGFTNMEGLMRVS-DFVNYRSFMSDI  359

Query  1093  NIKIEKSLPKNLNITSQEAVTEIVGNFKKIYMGDGDSFGDLV-SLSKWYGEPYLNYPSLE  1269
             N K    LP +L    +E   ++  + K+ Y GD     D +     ++ +    YP+L
Sbjct  360   NEKFSDYLPNDLQFRDKEEKEKVARDVKQFYFGDKVINNDTIFEYIDYFSDNMFAYPTLR  419

Query  1270  ETELILKSSKQPIYNYLFNYSGWRNLPKLGGGSAYKDVPGATHADDLFYMFSQEWLSGFF  1449
              +L +++     +Y Y F+Y       P+   G   K + G++H      +   + L+
Sbjct  420   SLKLQVEAGNNQVYLYQFSYPFENQAPE---GVMLK-LEGSSHTAQTHVVL--DTLNEEN  473

Query  1450  ENKMIERMT-TLWTNFAKYGDPTPEATDL  1533
             +  +I  +T   W NF   G P P  ++L
Sbjct  474   DLAIIRNITRQFWANFITTGTPVPAGSEL  502


> ta_transcript84818_1
Length=733

 Score =   214 bits (546),  Expect = 9e-60, Method: Compositional matrix adjust.
 Identities = 145/408 (36%), Positives = 216/408 (53%), Gaps = 39/408 (10%)
 Frame = +1
```

```
Query  13    CCIVNRNIVCVMCVRAKWLVLWSLWAARLVRQPTATVRVSNGLLRGAVAHDGSYVQYNGI  192
             CC++     C +              A+  + + TV +  G LRG    +G    +  I
Sbjct  18    CCVLAIGCACAL--------------AQPDDENSRTVEIQQGKLRGYKDPEGGLFTFYNI  63

Query  193   PFGSV-TDETKFQAPGPEPKWDGIYDAIHEEIRCNQQFSTNLTI----GRRDCLTLNIYT  357
             P+ +  T + +F+AP P P W    +A+    + C+Q     + I     + DCL  N+Y
Sbjct  64    PYATAPTGKDRFKAPLPAPVWITTREAVDRGVVCHQIDLAAVGIFNKTKQEDCLIANVYV  123

Query  358   PFNTDPGDKLVVMffihgggffqgsgssffygPRYLV-SKGVILVTINYRLNIQGFLCLG  534
             P +TD  +  VV+F   G              P+++V +  VI VT NYRL +QGFLCLG
Sbjct  124   P-DTDKKNLPVVVFVHGGAYLAGFGEVLT---PKHMVRTNNVIYVTFNYRLGVQGFLCLG  179

Query  535   IKEAPGNAALKDMVAALKWVQRNIASFGGDPDNVTIFGESAGAASVSFLVLSPMAKGLFH  714
               +APGNA +KDMVA L+WV++NIASFGG+PD+VTI G SAG+++V  L+LS   KGLFH
Sbjct  180   TPDAPGNAGMKDMVALLRWVKKNIASFGGNPDDVTIDGYSAGSSAVDLLLLSKATKGLFH  239

Query  715   KAITQSGSSLAPFGLQFKPIFLASLLAKV-MGF-ISQDPVKLYEYFMTKSDDELILTRVP  888
             KAI +SG+++A + +Q  PI  A   AK   GF I  D    L +++ +   DE+
Sbjct  240   KAIPESGANVAMWSVQVDPIANAKEYAKSEHGFEILDDIYALEDFYKSLPYDEMY-----  294

Query  889   RKEGHVII----SEILYTPCVEKVIEGVEPFLIQSPQEALSKGEFNKVPMIIGSNTQEGI  1056
              K G  ++    +  L++PCVE+   GVE FL  +P   L KG++NKVP++ G    EG+
Sbjct  295   -KSGMELMFKNKNNFLFSPCVERDT-GVEKFLDDTPVNILKKGDYNKVPVLYGFANMEGL  352

Query  1057  MILEMDNSTMRN--NIKIEKSLPKNLNITSQEAVTEIVGNFKKIYMGD  1194
               + M      +  N K  ++LP +L    +E   ++    KK Y GD
Sbjct  353   FRVGMGYEYFMSQLNDKFSENLPHDLEFKDEEEKRKVADEVKKFYFGD  400


> ta_transcript84816_1
Length=947

 Score =   214 bits (545),  Expect = 4e-59, Method: Compositional matrix adjust.
 Identities = 141/377 (37%), Positives = 208/377 (55%), Gaps = 25/377 (7%)
 Frame = +1

Query  106   QPTATVRVSNGLLRGAVAHDGSYVQYNGIPFGSV-TDETKFQAPGPEPKWDGIYDAIHEE  282
             + + TV +  G LRG    +G    +  IP+ +  T + +F+AP P P W    +A+
Sbjct  35    ENSRTVEIQQGKLRGYKDPEGGLFTFYNIPYATAPTGKDRFKAPLPAPVWITTREAVDRG  94

Query  283   IRCNQQFSTNLTI----GRRDCLTLNIYTPFNTDPGDKLVVMffihgggffqgsgssffy  450
             + C+Q     + I     + DCL  N+Y P +TD  +  VV+F   G
Sbjct  95    VVCHQIDLAAVGIFNKTKQEDCLIANVYVP-DTDKKNLPVVVFVHGGAYLAGFGEVLT--  151

Query  451   gPRYLV-SKGVILVTINYRLNIQGFLCLGIKEAPGNAALKDMVAALKWVQRNIASFGGDP  627
              P+++V +  VI VT NYRL +QGFLCLG  +APGNA +KDMVA L+WV++NIASFGG+P
Sbjct  152   -PKHMVRTNNVIYVTFNYRLGVQGFLCLGTPDAPGNAGMKDMVALLRWVKKNIASFGGNP  210

Query  628   DNVTIFGESAGAASVSFLVLSPMAKGLFHKAITQSGSSLAPFGLQFKPIFLASLLAKV-M  804
             D+VTI G SAG+++V  L+LS   KGLFHKAI +SG+++A + +Q  PI  A   AK
Sbjct  211   DDVTIDGYSAGSSAVDLLLLSKATKGLFHKAIPESGANVAMWSVQVDPIANAKEYAKSEH  270

Query  805   GF-ISQDPVKLYEYFMTKSDDELILTRVPRKEGHVII----SEILYTPCVEKVIEGVEPF  969
             GF I  D    L +++ +   DE+       K G  ++    +  L++PCVE+   GVE F
Sbjct  271   GFEILDDIYALEDFYKSLPYDEMY------KSGMELMFKNKNNFLFSPCVER-DTGVEKF  323

Query  970   LIQSPQEALSKGEFNKVPMIIGSNTQEGIMILEMDNSTMRN--NIKIEKSLPKNLNITSQ  1143
             L  +P   L KG++NKVP++ G    EG+  + M      +  N K  ++LP +L    +
Sbjct  324   LDDTPVNILKKGDYNKVPVLYGFANMEGLFRVGMGYEYFMSQLNDKFSENLPHDLEFKDE  383

Query  1144  EAVTEIVGNFKKIYMGD  1194
             E   ++    KK Y GD
Sbjct  384   EEKRKVADEVKKFYFGD  400


> ta_transcript48580_1
Length=834

 Score =   211 bits (536),  Expect = 4e-58, Method: Compositional matrix adjust.
 Identities = 191/567 (34%), Positives = 275/567 (49%), Gaps = 54/567 (10%)
 Frame = +1

Query  88    AARLVRQPTATVRVSNG--------LLRG--AVAHDGS---YVQYNGI-----PFGSVTD  213
             AARLV      +RV  G         LRG  VA   +   Y  + GI     P GS+
Sbjct  137   AARLVHALVDRMRVGGGPIVMTELGALRGRRVVARTTAQTPYFSFKGIRYAQPPRGSL--  194

Query  214   ETKFQAPGPEPKWDGIYDAIHEEIRCNQQFSTNLTI-GRRDCLTLNIYTPF----NTDPG  378
```

```
                 +F+ P P   W G+ DA+ E   C  +F    T  G  DCL LN+YTP      T
Sbjct  195   --RFKPPVPLEPWSGVRDALEEGAVCPHRFMLFDTYKGDEDCLFLNVYTPALPEKITGFN  252

Query  379   DKLVVMffihgggffqgsgssffygPRYLVSKGVILVTINYRLNIQGFLCLGIKEAPGNA  558
              KL VM +IHGG F  GSG++F YGP +L+   ++LVT+NYRL   GFL +   E  GN
Sbjct  253   PKLAVMVWIHGGAFAVGSGNAFLYGPDHLMGGNIVLVTLNYRLGALGFLSMENDEVSGNM  312

Query  559   ALKDMVAALKWVQRNIASFGGDPDNVTIFGESAGAASVSFLVLSPMAKGLFHKAITQSGS  738
              LKD V ALKWV+ NI  FGGD   VTIFGESAGAASV   +LSP +KGLFH+AI QSG
Sbjct  313   GLKDQVLALKWVRDNIEYFGGDASRVTIFGESAGAASVHLHMLSPASKGLFHRAIAQSGL  372

Query  739   SLAPFGLQFKPIFLASLLAKVMGFISQDPVKLYEYFMTKSDDELILTRVPRKEGHVIISE  918
             +L+P+ L   P   A  L + +G  +    +L  Y +  +  EL++    R  G    S
Sbjct  373   ALSPWALTRSPRERAFELGRELGIDTNSTAELLGY-LRATPSELLVKAGARLAGAPGKSV  431

Query  919   ILYTPCVEKVIEGVEP-----FLIQSPQEALSKGEFNKVPMIIGSNTQEGIMILEMDNST  1083
              L++      +  VEP     FL Q P+  +   +   VP++ G N QEGI++
Sbjct  432   DLHSTVALPFVPTVEPDVPDAFLTQHPRNLMPGAD---VPLLTGYNAQEGIILFRRLQRY  488

Query  1084  MRNNIKIEKS----LPKNLNITSQEAVTEIVGNFKKIYMGDGD--SFGDLVSLSKWYGEP  1245
              +   ++++     +P  L +T  EA    V +  + +          ++ SL   + +
Sbjct  489   PKLLTELDREFRRVVPPEL-LTGDEAQNRRVADHIRAFYFQQRPVDIRNIDSLIDLFTDV  547

Query  1246  YLNYPSLEETELILKSSK-QPIYNYLFNYSGWRNLPKLGGGSAYKDVPGATHADDLFYMF  1422
                 P LE   +  K+++  P Y Y F + G   L K   G  +   PGA H D++ Y+F
Sbjct  548   MFLRPLLETLRIQGKTNRTSPTYLYRFAFDGALGLFKRMLGINH---PGACHGDEMGYLF  604

Query  1423  SQEWLSGFFENKMIER-----MTTLWTNFAKYGDPT-PEATDLLPVKWYPTNLTTPMNFV  1584
                 L+   ++   E      M  +WTNFAK G+PT  +  D +  KW P N TT + ++
Sbjct  605   YFSRLNYRLDDDSTELAVSKGMVRMWTNFAKTGNPTIGDHDDPIDFKWSPVNDTTHLTYL  664

Query  1585  -LDEAFSTVPLWYNDAMKYWKQVYQKY  1662
              ++  F+         ++ W  +Y+ Y
Sbjct  665   DINGHFTQKQDPEARRVRLWDWLYENY  691


> ta_transcript48579_1
Length=905

 Score =   211 bits (537),  Expect = 4e-58, Method: Compositional matrix adjust.
 Identities = 192/571 (34%), Positives = 276/571 (48%), Gaps = 62/571 (11%)
 Frame = +1

Query  88    AARLVRQPTATVRVSNG--------LLRG--AVAHDGS---YVQYNGI-----PFGSVTD  213
             AARLV      +RV  G         LRG   VA   +   Y +  GI     P GS+
Sbjct  137   AARLVHALVDRMRVGGGPIVMTELGALRGRRVVARTTAQTPYFSFKGIRYAQPPRGSL--  194

Query  214   ETKFQAPGPEPKWDGIYDAIHEEIRCNQQFSTNLTI-GRRDCLTLNIYTP--------FN  366
               +F+ P P   W G+ DA+ E   C  +F    T  G  DCL LN+YTP        FN
Sbjct  195   --RFKPPVPLEPWSGVRDALEEGAVCPHRFMLFDTYKGDEDCLFLNVYTPALPEKITGFN  252

Query  367   TDPGDKLVVMffihgggffqgsgssffygPRYLVSKGVILVTINYRLNIQGFLCLGIKEA  546
                  KL VM +IHGG F  GSG++F YGP +L+   ++LVT+NYRL   GFL +   E
Sbjct  253   P----KLAVMVWIHGGAFAVGSGNAFLYGPDHLMGGNIVLVTLNYRLGALGFLSMENDEV  308

Query  547   PGNAALKDMVAALKWVQRNIASFGGDPDNVTIFGESAGAASVSFLVLSPMAKGLFHKAIT  726
              GN  LKD V ALKWV+ NI  FGGD   VTIFGESAGAASV   +LSP +KGLFH+AI
Sbjct  309   SGNMGLKDQVLALKWVRDNIEYFGGDASRVTIFGESAGAASVHLHMLSPASKGLFHRAIA  368

Query  727   QSGSSLAPFGLQFKPIFLASLLAKVMGFISQDPVKLYEYFMTKSDDELILTRVPRKEGHV  906
             QSG +L+P+ L   P   A  L + +G  +    +L  Y +  +  EL++    R  G
Sbjct  369   QSGLALSPWALTRSPRERAFELGRELGIDTNSTAELLGY-LRATPSELLVKAGARLAGAP  427

Query  907   IISEILYTPCVEKVIEGVEP-----FLIQSPQEALSKGEFNKVPMIIGSNTQEGIMILEM  1071
               S  L++      +  VEP     FL Q P+  +   +   VP++ G N QEGI++
Sbjct  428   GKSVDLHSTVALPFVPTVEPDVPDAFLTQHPRNLMPGAD---VPLLTGYNAQEGIILFRR  484

Query  1072  DNSTMRNNIKIEKS----LPKNLNITSQEAVTEIVGNFKKIYMGDGD--SFGDLVSLSKW  1233
                  +   ++++     +P  L +T  EA    V +  + +          ++ SL
Sbjct  485   LQRYPKLLTELDREFRRVVPPEL-LTGDEAQNRRVADHIRAFYFQQRPVDIRNIDSLIDL  543

Query  1234  YGEPYLNYPSLEETELILKSSK-QPIYNYLFNYSGWRNLPKLGGGSAYKDVPGATHADDL  1410
             + +     P LE   +  K+++  P Y Y F + G   L K   G  +   PGA H D++
Sbjct  544   FTDVMFLRPLLETLRIQGKTNRTSPTYLYRFAFDGALGLFKRMLGINH---PGACHGDEM  600

Query  1411  FYMFSQEWLSGFFENKMIER-----MTTLWTNFAKYGDPT-PEATDLLPVKWYPTNLTTP  1572
              Y+F    L+   ++   E      M  +WTNFAK G+PT  +  D +  KW P N TT
```

```
Sbjct  601   GYLFYFSRLNYRLDDDSTELAVSKGMVRMWTNFAKTGNPTIGDHDDPIDFKWSPVNDTTH  660

Query  1573  MNFV-LDEAFSTVPLWYNDAMKYWKQVYQKY  1662
             + ++ ++  F+         ++ W  +Y+ Y
Sbjct  661   LTYLDINGHFTQKQDPEARRVRLWDWLYENY  691


> ta_transcript48393_1
Length=666

 Score =   197 bits (502),  Expect = 4e-54, Method: Compositional matrix adjust.
 Identities = 134/411 (33%), Positives = 213/411 (52%), Gaps = 18/411 (4%)
 Frame = +1

Query  121   VRVSNGLLRGAVAHDGSYVQYNGIPFGSV-TDETKFQAPGPEPKWDGIYDAIHEEIRCNQ  297
             V  + G +RG    +G    +  IP+ +  +   KF AP   P W   ++A+ + I C Q
Sbjct  96    VETTQGPVRGYRDPEGGLFAFYSIPYATAPSGPLKFTAPITPPTWTEPFEAVDKRIICPQ  155

Query  298   Q---FSTNLTIGRRDCLTLNIYTPFNTDPGDKLVVMffihgggffqgsgssffygPRYLV  468
                 F+  +T+ + DCL  N+Y P    +    ++V            G+  +    P+ LV
Sbjct  156   MKSGFTKQMTM-QVDCLIANVYVPDTKETKLPVIVYVHGGAFQVGFGNLMT----PKNLV  210

Query  469   -SKGVILVTINYRLNIQGFLCLGIKEAPGNAALKDMVAALKWVQRNIASFGGDPDNVTIF  645
              SK +I VT NYRL   GFLCLG ++APGNA LKD VAAL+WV +NI   GG+PD+VTI
Sbjct  211   RSKNIIAVTFNYRLGAHGFLCLGTEDAPGNAGLKDQVAALRWVNKNIIKLGGNPDDVTIA  270

Query  646   GESAGAASVSFLVLSPMAKGLFHKAITQSGSSLAPFGLQFKPIFLASLLAKVMGFISQDP  825
             G SAGA SV   + +S  A+GLF K I +SGSSL    +    P+  A + A  +GF + D
Sbjct  271   GYSAGAISVDLMAISKSAEGLFSKIIIESGSSLFAGSIVTDPLENAKIFAARLGFNNIDD  330

Query  826   V-KLYEYFMTKSDDELILTRVPRKEGHVIISEILYTPCVEKVIEGVEPFLIQSPQEALSK  1002
             V  L +++   S D L       +      S +L++PCVE+  +G+  FL + P   L +
Sbjct  331   VYALEKFYKAASYDSLTKGEFMDRPN----STLLFSPCVERDDDGLA-FLSEPPINILKE  385

Query  1003  GEFNKVPMIIGSNTQEGIMILE-MDNSTMRNNIKIEKSLPKNLNITSQEAVTEIVGNFKK  1179
             G + +VPMI G +  EG+      +   ++ N +  + +P +L+  S+     I    KK
Sbjct  386   GSYTQVPMIYGFSNMEGLFREPFFEFWKLKMNEQFSEFIPGDLHFESKHDRERIAKRIKK  445

Query  1180  IYMGDGDSFGD-LVSLSKWYGEPYLNYPSLEETELILKSSKQPIYNYLFNY  1329
              Y  D    G+ ++    ++G+  + YP+L   ++ +++    +Y Y ++Y
Sbjct  446   FYFWDSPINGENILKYIDYFGDVLVKYPALRTVKMHIQNGHDKLYLYEYSY  496


> ta_transcript15565_1
Length=424

 Score =   159 bits (401),  Expect = 6e-42, Method: Compositional matrix adjust.
 Identities = 120/386 (31%), Positives = 193/386 (50%), Gaps = 28/386 (7%)
 Frame = +1

Query  559   ALKDMVAALKWVQRNIASFGGDPDNVTIFGESAGAASVSFLVLSPMAKGLFHKAITQSGS  738
              +KDMVA L+WV RNIA+FGG+P++VT+ G SAG+ SV  L+LS  AKGLFHKAI +SGS
Sbjct  1     GMKDMVALLRWVNRNIAAFGGNPNDVTLEGWSAGSVSVELLMLSKAAKGLFHKAIPESGS  60

Query  739   SLAPFGLQFKPIFLASLLAKV-MGFISQDPVKLYEYFMTKSDDELILTRVPRKEGHVII-  912
             S APF +    PI  A   A+ +GF   D +   E F      + +  +   ++G +II
Sbjct  61    SFAPFSVGVDPIGNAKDFARSDLGFKDVDNISALEEFY-----KTVPFKDMYQQGPLIIF  115

Query  913   -SEILYTPCVEKVIEGVEPFLIQSPQEALSKGEFNKVPMIIGSNTQEGIMILEMDNSTMR  1089
                  +TPCVE+ I G + FL  +P   L KG++ KVPM+ G + +EG+  ++
Sbjct  116   RHNFTFTPCVERNI-GQDVFLDDTPYNILKKGDYMKVPMLYGFSNKEGLYRVDYGYQFFI  174

Query  1090  N--NIKIEKSLPKNLNITSQEAVTEIVGNFKKIYMGDGDSFGDLV-SLSKWYGEPYLNYP  1260
                N K    LP +L    +    ++    K+ Y G+     D V     ++ +     P
Sbjct  175   EGMNKKFSDYLPNDLQFKDENEKEQVATMIKEFYFGNKKIDQDTVLEYIDYFTDSMFAGP  234

Query  1261  SLEETELILKSSKQPIYNYLFNYSGWRNLPKLGGGSAYKDVPGATHADDLFYMFSQEWLS  1440
             SL+E +L + +    IY Y F+Y     +P+    S    V G+ H  +   +    WL
Sbjct  235   SLKEVQLHVAAGNNQIYLYQFSYPYENKVPE----SIKVKVIGSNHCGETMTVLDN-WLG  289

Query  1441  G----FFENKMIERMTT----LWTNFAKYGDPTPEATDLLPVKWYPTNLT-TPMNFVLDE  1593
             G       N  +E++       +W NF K G P P+ ++L    W  T+ + +P   + ++
Sbjct  290   GAEMPAERNSDLEQVRNVTRQIWGNFIKTGKPVPQGSNL--PTWPATDGSGSPYMVITNK  347

Query  1594  AFSTVPLWYNDAMKYWKQVYQKYRRR  1671
                   +   ++ +W ++YQ++ R+
Sbjct  348   PELKGQPFQPMSVMFWTEIYQRHYRQ  373
```

```
> ta_transcript49309_1
Length=664

 Score =   133 bits (335),  Expect = 3e-32, Method: Compositional matrix adjust.
 Identities = 146/556 (26%), Positives = 222/556 (40%), Gaps = 89/556 (16%)
 Frame = +1

Query  181   YNGIPFGSV-TDETKFQAPG--PEPKWDGIYDAIHEEIRCNQQ-----------------  300
             Y GIP+     D  +F  P     P+W+G+Y+A      C Q
Sbjct  53    YVGIPYAQPPIDRLRFMPPEYLNPPQWEGVYNATIFAPDCMQSDPKKDDVQSTLKKHDEL  112

Query  301   ----FSTNLTIGRR-----DCLTLNIYTPFNTDPGDKLVVMffihgggffqgsgssffyg  453
                   + L   R       DCL LN+Y P +       V+++F  G
Sbjct  113   FMKLLDSQLETPREKNYSEDCLYLNVYVPDDYKVEGYPVMVWFHGGEFVRGSPNYMN---  169

Query  454   PRYLVSK-GVILVTINYRLNIQGFLCLGIKEAPGNAALKDMVAALKWVQRNIASFGGDPD  630
             P +LV K  VI V++ YRLNI GF      EA GN  L D VAAL WV+ NI  FGGDP+
Sbjct  170   PFHLVLKQKVIFVSVAYRLNIFGFFTTLDHEALGNFGLHDQVAALSWVKTNIEHFGGDPE  229

Query  631   NVTIFGESAGAASVSFLVLSPMAKGLFHKAITQSGSSLAP--FGLQFKPIFLASLLAKVM  804
             N+ IFG  AGA SV   ++S  + GLFHKAI  SG+ L+P    +  K +     +A
Sbjct  230   NICIFGHDAGAVSVGLHLVSTYSPGLFHKAIAMSGNVLSPDTVAIARKELITVDRVASAF  289

Query  805   GFISQDPVKLYEYFMTKSDDELILTRVPRKEGHVIISEILYTPCVEKVIEGVE-PFLIQS  981
                 +   +L +         L+    P  E         + P V+     +  PFL
Sbjct  290   SCFRKPTFQLLDCLRRVPSQALLDLGAPLAE---------WKPIVDSGFSNISVPFLSDI  340

Query  982   PQEALSKGEFNKVPMIIGSNTQEGIMIL-------------EMDNSTMRNNIKIEKSLPK  1122
             P        F  VP++ G    E  ++L             + +  +MR  + +
Sbjct  341   PSRLFKDEAFLSVPVLSGYTNMEDALLLVKEGDDESNAGLSQREFDSMREEVILADITVD  400

Query  1123  NLN-ITSQEAVTEIVGNFKKIYMGDGDSFGDLVSLSKWYGEPYLNYPSLEETELILK--S  1293
             N +  T+Q  + E V  F K      +       L K + + Y +      T  + K  S
Sbjct  401   NTSCFTNQHHIQEAVAFFYKPIPPTQNE----TILRKLFLDFYTDKVHGATTYALAKYLS  456

Query  1294  SKQPIYNYLFNYSGWRNLPKLGGGSAYKDVPG---ATHADDLFYMFSQEWL-----SGFF  1449
             S  P+Y Y F+   + ++       A +D+P      H  DL + F   +L     SG +
Sbjct  457   SHAPVYLYRFDLKPFSDI-------ATEDIPDWVKVAHNYDLIFAFGLPYLALPDDSGKW  509

Query  1450  ENK---MIERMTTLWTNFAKYGDPTPEATDLLPVKWYPTNLTTPMNFVLDEA---FSTVP  1611
             + +   + + +  +W+NFA Y +PT      + ++W P  +  P   ++D A    ST
Sbjct  510   DKRDKSISDIIMKMWSNFAWYSNPTNSG---VFIEWNPFEIENPGYLIIDRANFTMSTPE  566

Query  1612  LWYNDAMKYWKQVYQK  1659
                  A ++W   Y K
Sbjct  567   TINYRAFEFWADFYPK  582

************************************************************************************************
***********************************************************************

Query= gi|512885543:14-1618 PREDICTED: Bombyx mori juvenile hormone esterase-like
(LOC101741415), mRNA

Length=1605
                                                                      Score     E
Sequences producing significant alignments:                          (Bits)  Value

  ta_transcript48580_1                                                  223   1e-62
  ta_transcript48579_1                                                  223   2e-62
  ta_transcript75783_1                                                  207   3e-57
  ta_transcript58915_1                                                  202   3e-56
  ta_transcript58914_1                                                  202   7e-56
  ta_transcript48393_1                                                  178   1e-47
  ta_transcript29751_1                                                  167   2e-44
  ta_transcript84821_1                                                  148   9e-38
  ta_transcript84818_1                                                  148   3e-37
  ta_transcript84816_1                                                  147   1e-36
  ta_transcript15565_1                                                  132   8e-33


> ta_transcript48580_1
Length=834

 Score =   223 bits (569),  Expect = 1e-62, Method: Compositional matrix adjust.
 Identities = 172/540 (32%), Positives = 261/540 (48%), Gaps = 30/540 (6%)
```

```
 Frame = +1

Query  64    PLVETQQGLIRGLR-----SENGKFSKFLGIPYALVDEKN-PFGPSVPHPGFEETFEAYD  225
             P+V T+ G +RG R     +    +  F GI YA     +  F P VP    +   +A +
Sbjct  154   PIVMTELGALRGRRVVARTTAQTPYFSFKGIRYAQPPRGSLRFKPPVPLEPWSGVRDALE  213

Query  226   DSVVCPQVIKVTKVGIGSLQCLNLNVY---VPNTATSRN-KRPVMVWIHgggfttgsgts  393
             +  VCP    +     G   CL LNVY   +P   T  N K  VMVWIHGG F  GSG +
Sbjct  214   EGAVCPHRFMLFDTYKGDEDCLFLNVYTPALPEKITGFNPKLAVMVWIHGGAFAVGSGNA  273

Query  394   RDYSYDDLVRHDVIVVSVNYRLGPYGFLCLDSPDVPGNQELKDQVLALKWTKENIEAFGG  573
               Y  D L+  ++++V++NYRLG  GFL +++ +V GN  LKDQVLALKW ++NIE FGG
Sbjct  274   FLYGPDHLMGGNIVLVTLNYRLGALGFLSMENDEVSGNMGLKDQVLALKWVRDNIEYFGG  333

Query  574   DASKITIFGESAGGQAVELHLLTDQDK-LFNQVIVQSGSIYKPGIINKPDNSVPIQIASR  750
             DAS++TIFGESAG  +V LH+L+   K LF++ I QSG    P  + +       ++
Sbjct  334   DASRVTIFGESAGAASVHLHMLSPASKGLFHRAIAQSGLALSPWALTRSPRERAFELGRE  393

Query  751   LGFETEDFVAAISFLADQDPHLVVAAST----SEGSTV--FSGSAYRACVENEFDGIHSY  912
             LG +T      + +L      L+V A      + G +V   S  A       E D   ++
Sbjct  394   LGIDTNSTAELLGYLRATPSELLVKAGARLAGAPGKSVDLHSTVALPFVPTVEPDVPDAF  453

Query  913   LSDSPQNLKI-KNIPVIYGVNSKEYLTLHASLVPEQYEVSGF-KSFLKLAFN--LTDDDM  1080
             L+  P+NL    ++P++ G N++E + L   L      ++   + F ++     LT D+
Sbjct  454   LTQHPRNLMPGADVPLLTGYNAQEGIILFRRLQRYPKLLTELDREFRRVVPPELLTGDEA  513

Query  1081  E-----EDVRHFYIGDETLTEELFDEFITFASDYYYGYPVERSIK-KSLADGNKEIYYYV  1242
             +     + +R FY     +     D  I   +D  +  P+  +++ +   +     Y Y
Sbjct  514   QNRRVADHIRAFYFQQRPVDIRNIDSLIDLFTDVMFLRPLLETLRIQGKTNRTSPTYLYR  573

Query  1243  FSYDGGRNAMKEYLGITAAGVAHADELGYLLAVDAVPGQHIAEEDQLIIDR-ITTLWANF  1419
             F++DG     K  LGI   G  H DE+GYL     +  +   +  +L + + +  +W NF
Sbjct  574   FAFDGALGLFKRMLGINHPGACHGDEMGYLFYFSRLNYRLDDDSTELAVSKGMVRMWTNF  633

Query  1420  AKYGNPT-PEPTDLLPVVWSTVEGNKR-PYLDIDTDLQLKGRPFHQRIAFWDLFYKLYGE  1593
             AK GNPT  +  D +   WS V       YLDI+     K  P  +R+  WD  Y+ Y
Sbjct  634   AKTGNPTIGDHDDPIDFKWSPVNDTTHLTYLDINGHFTQKQDPEARRVRLWDWLYENYAN  693


> ta_transcript48579_1
Length=905

 Score =   223 bits (568),  Expect = 2e-62, Method: Compositional matrix adjust.
 Identities = 172/540 (32%), Positives = 261/540 (48%), Gaps = 30/540 (6%)
 Frame = +1

Query  64    PLVETQQGLIRGLR-----SENGKFSKFLGIPYALVDEKN-PFGPSVPHPGFEETFEAYD  225
             P+V T+ G +RG R     +    +  F GI YA     +  F P VP    +   +A +
Sbjct  154   PIVMTELGALRGRRVVARTTAQTPYFSFKGIRYAQPPRGSLRFKPPVPLEPWSGVRDALE  213

Query  226   DSVVCPQVIKVTKVGIGSLQCLNLNVY---VPNTATSRN-KRPVMVWIHgggfttgsgts  393
             +  VCP    +     G   CL LNVY   +P   T  N K  VMVWIHGG F  GSG +
Sbjct  214   EGAVCPHRFMLFDTYKGDEDCLFLNVYTPALPEKITGFNPKLAVMVWIHGGAFAVGSGNA  273

Query  394   RDYSYDDLVRHDVIVVSVNYRLGPYGFLCLDSPDVPGNQELKDQVLALKWTKENIEAFGG  573
               Y  D L+  ++++V++NYRLG  GFL +++ +V GN  LKDQVLALKW ++NIE FGG
Sbjct  274   FLYGPDHLMGGNIVLVTLNYRLGALGFLSMENDEVSGNMGLKDQVLALKWVRDNIEYFGG  333

Query  574   DASKITIFGESAGGQAVELHLLTDQDK-LFNQVIVQSGSIYKPGIINKPDNSVPIQIASR  750
             DAS++TIFGESAG  +V LH+L+   K LF++ I QSG    P  + +       ++
Sbjct  334   DASRVTIFGESAGAASVHLHMLSPASKGLFHRAIAQSGLALSPWALTRSPRERAFELGRE  393

Query  751   LGFETEDFVAAISFLADQDPHLVVAAST----SEGSTV--FSGSAYRACVENEFDGIHSY  912
             LG +T      + +L      L+V A      + G +V   S  A       E D   ++
Sbjct  394   LGIDTNSTAELLGYLRATPSELLVKAGARLAGAPGKSVDLHSTVALPFVPTVEPDVPDAF  453

Query  913   LSDSPQNLKI-KNIPVIYGVNSKEYLTLHASLVPEQYEVSGF-KSFLKLAFN--LTDDDM  1080
             L+  P+NL    ++P++ G N++E + L   L      ++   + F ++     LT D+
Sbjct  454   LTQHPRNLMPGADVPLLTGYNAQEGIILFRRLQRYPKLLTELDREFRRVVPPELLTGDEA  513

Query  1081  E-----EDVRHFYIGDETLTEELFDEFITFASDYYYGYPVERSIK-KSLADGNKEIYYYV  1242
             +     + +R FY     +     D  I   +D  +  P+  +++ +   +     Y Y
Sbjct  514   QNRRVADHIRAFYFQQRPVDIRNIDSLIDLFTDVMFLRPLLETLRIQGKTNRTSPTYLYR  573

Query  1243  FSYDGGRNAMKEYLGITAAGVAHADELGYLLAVDAVPGQHIAEEDQLIIDR-ITTLWANF  1419
             F++DG     K  LGI   G  H DE+GYL     +  +   +  +L + + +  +W NF
Sbjct  574   FAFDGALGLFKRMLGINHPGACHGDEMGYLFYFSRLNYRLDDDSTELAVSKGMVRMWTNF  633
```

```
Query  1420  AKYGNPT-PEPTDLLPVVWSTVEGNKR-PYLDIDTDLQLKGRPFHQRIAFWDLFYKLYGE  1593
             AK GNPT  +  D +   WS V       YLDI+     K  P  +R+  WD  Y+ Y
Sbjct  634   AKTGNPTIGDHDDPIDFKWSPVNDTTHLTYLDINGHFTQKQDPEARRVRLWDWLYENYAN  693


> ta_transcript75783_1
Length=733

 Score =   207 bits (526),  Expect = 3e-57, Method: Compositional matrix adjust.
 Identities = 186/551 (34%), Positives = 260/551 (47%), Gaps = 57/551 (10%)
 Frame = +1

Query  52    LRIDPLVETQQGLIRGLRSEN---GKFSKFLGIPYALVDEKNPFG--------PSVPHPG  198
             + +D  V+  +G+++G   +N   G +  F GIP+A    + P G        P+ P  G
Sbjct  120   MSLDNTVKVSEGILQGELVQNEFGGSYYSFKGIPFA----EPPVGDLRFKAPIPAKPWKG  175

Query  199   FEETFEAYDDSVVCPQVIKVTKVGIGSLQCLNLNVYVPNTATSRNKRPVMVWIHgggftt  378
              + T   +        V K      GS  CL LNVY P         PVMVWIHGGG+
Sbjct  176   VK-TATKFGPINYQFDVFKKDPPS-GSEDCLYLNVYTPEIKPI-TLLPVMVWIHGGGYFW  232

Query  379   gsgtsRDYSYDDLVRHDVIVVSVNYRLGPYGFLCLDSPDVPGNQELKDQVLALKWTKENI  558
             GSG    Y  + LVRH VI+V+ NYRLG  GFL LD+ +VPGN  +KDQV ALKW K NI
Sbjct  233   GSGNDDVYGPEFLVRHGVILVTFNYRLGVLGFLSLDTAEVPGNAGMKDQVAALKWVKSNI  292

Query  559   EAFGGDASKITIFGESAGGQAVELHLLTDQDK-LFNQVIVQSGSIYKPGIINKPDNSVPI  735
               FGGD   ITIFGESAG  AV LHLL+   K LF + I+QSGS               I
Sbjct  293   HFFGGDPENITIFGESAGAGAVSLHLLSPMSKGLFKRAIIQSGSATAYWAQAFEIKEKSI  352

Query  736   QIASRLGFETEDFVAAISFLADQDPHLVVAAST----SEGSTVFSGSAYRACVENEFDGI  903
              +A +LG  T+D      F  +    L++  S     +E   V     +   +E +F G
Sbjct  353   ALARKLGLVTDDDQKLYEFFGNISKELLINISVPVTFAEQEEV--NIEFAPTIEKDF-GQ  409

Query  904   HSYLSDSP----QNLKIKNIPVIYGVNSKEYLTLHASLVPEQYEVSGFKSFLKLAFNLTD  1071
               +L+D      Q    K + V+ G    E +    SL     E+    +FL+    L
Sbjct  410   ERFLTDDSYKILQQGIHKGVDVMMGYVEDEGVLYLNSLEILDKEIERINNFLEA---LVP  466

Query  1072  DDMEEDV------------RHFYIGDETLTEELFDEFITFASDYYYGYPVERSIKKSLAD  1215
              +M+ ++            + FY  ++ +T   +     + S   + Y V +S K
Sbjct  467   RNMKREIPIKQQLKVGRKMKKFYFDNKLVTRNDWRTIARYISLNVFSYDVIQSQKNIAKS  526

Query  1216  GNKEIYYYVFSYDGGRNAMKEYLG---IT--AAGVAHADELGYLLAVDAVPGQHIAEEDQ  1380
              N   ++Y F+    RN  K+      IT     V H D+L YL  V  +  Q +A++ +
Sbjct  527   QNS--FFYKFTCKSKRNFFKKVFTDDEITKDVDVVCHCDDLAYLFPVKFM-NQSVAKDSR  583

Query  1381  --LIIDRITTLWANFAKYGNPTPEPTDLLPVVWSTVEGNKRPYLDIDTDLQLKGRPFHQR  1554
                +ID++T LW NFAKYGNPTP+ +  L V W       + YLDI  DL LK +P  +
Sbjct  584   EFQLIDKVTKLWTNFAKYGNPTPDNS--LGVKWEPFTLQHQHYLDIGEDLVLKTKPDDED  641

Query  1555  IAFWDLFYKLY  1587
             I FW+  Y+ Y
Sbjct  642   IQFWEELYREY  652


> ta_transcript58915_1
Length=606

 Score =   202 bits (514),  Expect = 3e-56, Method: Compositional matrix adjust.
 Identities = 178/541 (33%), Positives = 269/541 (50%), Gaps = 51/541 (9%)
 Frame = +1

Query  70    VETQQGLIRGLR----SENGKFSKFLGIPYALVD-EKNPFGPSVPHPGFEETFEAYDDSV  234
             V+ +QG + G +    S +G+F  F GIPYA     K  F    P   ++   +A
Sbjct  32    VQVEQGWLEGEQLDSVSGDGQFFSFKGIPYAAPPVGKLRFKAPQPPVSWQGVRKATQHGP  91

Query  235   VCPQVIKVTKVGI-GSLQCLNLNVYVPNTATSRNKRPVMVWIHgggfttgsgtsRDYSYD  411
              CPQ    +K  I GS  CL LNVY  +  + ++  PVMV+IHGGGF +GSG    Y  D
Sbjct  92    KCPQRDIFSKQIIPGSEDCLYLNVYTKDI-SPKSPLPVMVFIHGGGFKSGSGDEDFYGGD  150

Query  412   DLVRHDVIVVSVNYRLGPYGFLCLDSPDVPGNQELKDQVLALKWTKENIEAFGGDASKIT  591
              LV H V++V++NYRL   GFLCLD+ +VPGN  LKDQV ALKW ++NI AFGGD + +T
Sbjct  151   FLVHHGVVLVTINYRLDALGFLCLDTEEVPGNAGLKDQVAALKWVQKNIFAFGGDPTNVT  210

Query  592   IFGESAGGQAVELHLLTDQDK-LFNQVIVQSGSIYKPGIINKPDNSVPIQIASR------  750
             IFGESAGG +  LH+L+   K LF + I  SG  +        D S+P +   R
Sbjct  211   IFGESAGGSSTALHVLSPLSKGLFKRAIPMSGVPF-------CDWSIPFEPRKRAFTLGK  263
```

```
Query  751   -LGFETEDFVAAISFLADQ------DPHLVVAASTSEGSTVFSGSAYRACVENEFDGIHS  909
              LGFETED  A + +L         D +  +      +   +      +   VE  F G H
Sbjct  264   ILGFETEDPKALLEYLQSLPAEKFVDTNPTIMGFEEKSYNMLKMYHFTPVVEKNF-GQHH  322

Query  910   YLSDSP-QNLK---IKNIPVIYGVNSKEYLTLHASLVP-----EQY-EVSGFKSFLKLAF  1059
             ++++ P + LK   + ++ V+ G   +E +     S V      ++Y EV   +  L  +
Sbjct  323   FMTEEPLEALKQGHVNDVDVLIGNTDQETIVGIPSFVDLLKMYDRYPEVFVPRKILNKST  382

Query  1060  NLTDDDMEEDVRHFYIGDETLTEELFDEFITFASDYYYGYPVERSIKKSLAD--GNKEIY  1233
                  ++ E +R  Y GD+ + +    E +T+ S+  + Y V +     L+   G+ ++Y
Sbjct  383   PGKILEIAERIRKHYFGDKPIDKTSMKEAVTYFSEVCFTYDVNKYTNLLLSGKPGSSKVY  442

Query  1234  YYVFSYDGGRNAMKEY---LGITAAGVAHADELGYLLAVDAV--PGQHIAEEDQLIIDRI  1398
               Y FS    RN   +Y    GIT  G +H ++L YL    +V  P    ++  ++ I +
Sbjct  443   RYRFSCVSERNIFGKYGQEYGIT--GASHLEDLMYLFDAKSVKLPLDRNSKSYKM-IQQT  499

Query  1399  TTLWANFAKYGNPTPEPTDLLPVVWSTVEGNKRPYLDIDTDLQLKGRPFHQRIAFWDLFY  1578
                L+ NFAKYGNPTP+ +  L   W   +    + ++DI   L +        + FW+  Y
Sbjct  500   CALFTNFAKYGNPTPDSS--LGFTWPEYDIKDQSFVDIADQLTVGRHLDADAVKFWESIY  557

Query  1579  K  1581
             +
Sbjct  558   Q  558


> ta_transcript58914_1
Length=650

 Score =   202 bits (513),  Expect = 7e-56, Method: Compositional matrix adjust.
 Identities = 178/541 (33%), Positives = 269/541 (50%), Gaps = 51/541 (9%)
 Frame = +1

Query  70    VETQQGLIRGLR----SENGKFSKFLGIPYALVD-EKNPFGPSVPHPGFEETFEAYDDSV  234
             V+ +QG + G +     S +G+F  F GIPYA     K  F     P   ++    +A
Sbjct  32    VQVEQGWLEGEQLDSVSGDGQFFSFKGIPYAAPPVGKLRFKAPQPPVSWQGVRKATQHGP  91

Query  235   VCPQVIKVTKVGI-GSLQCLNLNVYVPNTATSRNKRPVMVWIHgggfttgsgtsRDYSYD  411
              CPQ    +K  I GS  CL LNVY  +  + ++  PVMV+IHGGGF +GSG    Y  D
Sbjct  92    KCPQRDIFSKQIIPGSEDCLYLNVYTKDI-SPKSPLPVMVFIHGGGFKSGSGDEDFYGGD  150

Query  412   DLVRHDVIVVSVNYRLGPYGFLCLDSPDVPGNQELKDQVLALKWTKENIEAFGGDASKIT  591
              LV H V++V++NYRL   GFLCLD+ +VPGN  LKDQV ALKW ++NI AFGGD + +T
Sbjct  151   FLVHHGVVLVTINYRLDALGFLCLDTEEVPGNAGLKDQVAALKWVQKNIFAFGGDPTNVT  210

Query  592   IFGESAGGQAVELHLLTDQDK-LFNQVIVQSGSIYKPGIINKPDNSVPIQIASR------  750
             IFGESAGG +  LH+L+   K LF + I  SG  +        D S+P +   R
Sbjct  211   IFGESAGGSSTALHVLSPLSKGLFKRAIPMSGVPF-------CDWSIPFEPRKRAFTLGK  263

Query  751   -LGFETEDFVAAISFLADQ------DPHLVVAASTSEGSTVFSGSAYRACVENEFDGIHS  909
              LGFETED  A + +L         D +  +      +   +      +   VE  F G H
Sbjct  264   ILGFETEDPKALLEYLQSLPAEKFVDTNPTIMGFEEKSYNMLKMYHFTPVVEKNF-GQHH  322

Query  910   YLSDSP-QNLK---IKNIPVIYGVNSKEYLTLHASLVP-----EQY-EVSGFKSFLKLAF  1059
             ++++ P + LK   + ++ V+ G   +E +     S V      ++Y EV   +  L  +
Sbjct  323   FMTEEPLEALKQGHVNDVDVLIGNTDQETIVGIPSFVDLLKMYDRYPEVFVPRKILNKST  382

Query  1060  NLTDDDMEEDVRHFYIGDETLTEELFDEFITFASDYYYGYPVERSIKKSLAD--GNKEIY  1233
                  ++ E +R  Y GD+ + +    E +T+ S+  + Y V +     L+   G+ ++Y
Sbjct  383   PGKILEIAERIRKHYFGDKPIDKTSMKEAVTYFSEVCFTYDVNKYTNLLLSGKPGSSKVY  442

Query  1234  YYVFSYDGGRNAMKEY---LGITAAGVAHADELGYLLAVDAV--PGQHIAEEDQLIIDRI  1398
               Y FS    RN   +Y    GIT  G +H ++L YL    +V  P    ++  ++ I +
Sbjct  443   RYRFSCVSERNIFGKYGQEYGIT--GASHLEDLMYLFDAKSVKLPLDRNSKSYKM-IQQT  499

Query  1399  TTLWANFAKYGNPTPEPTDLLPVVWSTVEGNKRPYLDIDTDLQLKGRPFHQRIAFWDLFY  1578
                L+ NFAKYGNPTP+ +  L   W   +    + ++DI   L +        + FW+  Y
Sbjct  500   CALFTNFAKYGNPTPDSS--LGFTWPEYDIKDQSFVDIADQLTVGRHLDADAVKFWESIY  557

Query  1579  K  1581
             +
Sbjct  558   Q  558


> ta_transcript48393_1
Length=666

 Score =   178 bits (452),  Expect = 1e-47, Method: Compositional matrix adjust.
```

```
 Identities = 141/416 (34%), Positives = 214/416 (51%), Gaps = 35/416 (8%)
 Frame = +1

Query  67    LVETQQGLIRGLRSENGKFSKFLGIPYALVDEKNPFGP-----SVPHPGFEETFEAYDDS  231
             +VET QG +RG R   G    F  IPYA      P GP      +  P + E FEA D
Sbjct  95    IVETTQGPVRGYRDPEGGLFAFYSIPYATA----PSGPLKFTAPITPPTWTEPFEAVDKR  150

Query  232   VVCPQVIK-VTKVGIGSLQCLNLNVYVPNTATSRNKRPVMVWIHgggfttgsgtsRDYSY  408
             ++CPQ+    TK     + CL  NVYVP+T  +  K PV+V++HGG F  G G     +
Sbjct  151   IICPQMKSGFTKQMTMQVDCLIANVYVPDTKET--KLPVIVYVHGGAFQVGFGNL--MTP  206

Query  409   DDLVR-HDVIVVSVNYRLGPYGFLCLDSPDVPGNQELKDQVLALKWTKENIEAFGGDASK  585
              +LVR  ++I V+ NYRLG +GFLCL + D PGN  LKDQV AL+W  +NI   GG+
Sbjct  207   KNLVRSKNIIAVTFNYRLGAHGFLCLGTEDAPGNAGLKDQVAALRWVNKNIIKLGGNPDD  266

Query  586   ITIFGESAGGQAVELHLLTDQ-DKLFNQVIVQSG-SIYKPGIINKPDNSVPIQIASRLGF  759
             +TI G SAG  +V+L  ++   + LF+++I++SG S++   I+  P  +  I  A+RLGF
Sbjct  267   VTIAGYSAGAISVDLMAISKSAEGLFSKIIIESGSSLFAGSIVTDPLENAKI-FAARLGF  325

Query  760   ETEDFVAAISFL---ADQDPHLVVAASTSEGSTVFSGSAYRACVENEFDGIHSYLSDSPQ  930
                D V A+      A  D            ST+     +  CVE + DG+ ++LS+ P
Sbjct  326   NNIDDVYALEKFYKAASYDSLTKGEFMDRPNSTLL----FSPCVERDDDGL-AFLSEPPI  380

Query  931   NL----KIKNIPVIYGVNSKEYLTLHASLVPEQYEVS-GFKSFL--KLAFNLTDD--DME  1083
             N+        +P+IYG ++ E L         + +++  F  F+   L F    D   +
Sbjct  381   NILKEGSYTQVPMIYGFSNMEGLFREPFFEFWKLKMNEQFSEFIPGDLHFESKHDRERIA  440

Query  1084  EDVRHFYIGDETLTEELFDEFITFASDYYYGYPVERSIKKSLADGNKEIYYYVFSY  1251
             + ++ FY  D  +  E   ++I +  D    YP  R++K  + +G+ ++Y Y +SY
Sbjct  441   KRIKKFYFWDSPINGENILKYIDYFGDVLVKYPALRTVKMHIQNGHDKLYLYEYSY  496


> ta_transcript29751_1
Length=506

 Score =   167 bits (422),  Expect = 2e-44, Method: Compositional matrix adjust.
 Identities = 156/490 (32%), Positives = 237/490 (48%), Gaps = 47/490 (10%)
 Frame = +1

Query  70    VETQQGLIRGLRSENGKFSKFLGIPYALV-DEKNPFGPSVPHPGFEETFEAYDDSVVCPQ  246
             V  +QG +RG +  +G    F  IPYA     K+ F   +  P +    +A D  VVCPQ
Sbjct  33    VVIEQGPVRGYKDPDGGLYVFYNIPYATAPTSKDRFKAPLAPPVWVTIRDAVDREVVCPQ  92

Query  247   VIKVTKVGIGSLQCLNLNVYVPNTATSRNKRPVMVWIHgggfttgsgtsRDYSYDDLVR-  423
             +++           CL  NV+VP+T  S     V+V +HGGG+ +G G  +  +  D+VR
Sbjct  93    MLETGSHKNIQEDCLIANVFVPDT--SEKNLSVVVLVHGGGYLSGFGNRQ--TPKDMVRD  148

Query  424   HDVIVVSVNYRLGPYGFLCLDSPDVPGNQELKDQVLALKWTKENIEAFGGDASKITIFGE  603
               +I V+ NYRLG +GFLCL + DVPGN  +KD V  L+W K+NI +FGG+   +TI G
Sbjct  149   KKLIYVTFNYRLGVHGFLCLGTADVPGNAGMKDMVALLRWVKKNIASFGGNPDDVTIDGH  208

Query  604   SAGGQAVELHLLTD-QDKLFNQVIVQSGSIYKPGIINKPDNSV---PIQIASRLG-----  756
             S+G  +V+L LL+   + LF++ I +SGS       N    SV   PI  A
Sbjct  209   SSGSSSVDLLLLSKATEGLFHKAIPESGS-------NVAMWSVQIDPIAHAKEFARDEHS  261

Query  757   FETEDFVAAISFLADQDPHLVVAAS--TSEGSTVFSGSAYRACVENEFDGIHSYLSDSPQ  930
             FE+ D + A+       P  V+  S  +   ++      ++ CVE +  G+  +L D P
Sbjct  262   FESGDDIYALEDFYKTLPFDVMYKSGMSLRYNSSVKNILFQPCVERD-TGVEKFLDDFPV  320

Query  931   NL----KIKNIPVIYGVNSKEYLTLHASLVPEQYEVS----GFKSFLKLAFNLTDDDMEE  1086
             N+        +P++YG  + E L    +  V  +  +S     F  +L       D + +E
Sbjct  321   NILKSGDYNKVPMLYGFTNMEGLMRVSDFVNYRSFMSDINEKFSDYLPNDLQFRDKEEKE  380

Query  1087  ----DVRHFYIGDETLTEELFDEFITFASDYYYGYPVERSIKKSLADGNKEIYYYVFSYD  1254
                 DV+ FY GD+ +  +   E+I + SD  + YP  RS+K  +  GN ++Y Y FSY
Sbjct  381   KVARDVKQFYFGDKVINNDTIFEYIDYFSDNMFAYPTLRSLKLQVEAGNNQVYLYQFSYP  440

Query  1255  GGRNAMKEYLGITAAGVAHADELGYLLAVDAVPGQHIAEEDQLIIDRITT--LWANFAKY  1428
                N   E + +   G +H  +   +L          + EE+ L I R  T   WANF
Sbjct  441   -FENQAPEGVMLKLEGSSHTAQTHVVLDT-------LNEENDLAIIRNITRQFWANFITT  492

Query  1429  GNPTPEPTDL  1458
             G P P  ++L
Sbjct  493   GTPVPAGSEL  502


> ta_transcript84821_1
```

```
Length=611

 Score =   148 bits (374),  Expect = 9e-38, Method: Compositional matrix adjust.
 Identities = 134/380 (35%), Positives = 189/380 (50%), Gaps = 49/380 (13%)
 Frame = +1

Query  70    VETQQGLIRGLRSENGKFSKFLGIPYALVDE-KNPFGPSVPHPGFEETFEAYDDSVVCPQ  246
             VE QQG +RG +   G    F  IPYA     K+ F   +P P +  T EA D  VVC Q
Sbjct  40    VEIQQGKLRGYKDPEGGLFTFYNIPYATAPTGKDRFKAPLPAPVWITTREAVDRGVVCHQ  99

Query  247   VIKVTKVGI----GSLQCLNLNVYVPNTATSRNKRPVMVWIHgggfttgsgtsRDYSYDD  414
              I +  VGI         CL  NVYVP+T    +   PV+V++HGG +  G G     +
Sbjct  100   -IDLAAVGIFNKTKQEDCLIANVYVPDT--DKKNLPVVVFVHGGAYLAGFGEV--LTPKH  154

Query  415   LVR-HDVIVVSVNYRLGPYGFLCLDSPDVPGNQELKDQVLALKWTKENIEAFGGDASKIT  591
             +VR ++VI V+ NYRLG  GFLCL +PD PGN  +KD V  L+W K+NI +FGG+   +T
Sbjct  155   MVRTNNVIYVTFNYRLGVQGFLCLGTPDAPGNAGMKDMVALLRWVKKNIASFGGNPDDVT  214

Query  592   IFGESAGGQAVELHLLTDQDK-LFNQVIVQSGS-IYKPGIINKPDNSVPIQIASRLGFET  765
             I G SAG  AV+L LL+   K LF++ I +SG+ +    +   P  +      S  GFE
Sbjct  215   IDGYSAGSSAVDLLLLSKATKGLFHKAIPESGANVAMWSVQVDPIANAKEYAKSEHGFEI  274

Query  766   EDFVAAISFLADQDPHLVVAASTSEGSTVFSGS---AYRACVENEFDGIHSYLSDSPQNL  936
              D + A+       P+  +  S  E   +F       +  CVE +  G+  +L D+P N+
Sbjct  275   LDDIYALEDFYKSLPYDEMYKSGME--LMFKNKNNFLFSPCVERD-TGVEKFLDDTPVNI  331

Query  937   KIK----NIPVIYGVNSKEYLTLHASLVPEQYEVS-GFKSFL-----KLAFNLTDD----  1074
               K     +PV+YG  + E L          + V  G++ F+     K + NL  D
Sbjct  332   LKKGDYNKVPVLYGFANMEGL----------FRVGMGYEYFMSQLNDKFSENLPHDLEFK  381

Query  1075  DMEE------DVRHFYIGDE  1116
             D EE      +V+ FY GD+
Sbjct  382   DEEEKRKVADEVKKFYFGDK  401


> ta_transcript84818_1
Length=733

 Score =   148 bits (373),  Expect = 3e-37, Method: Compositional matrix adjust.
 Identities = 134/380 (35%), Positives = 189/380 (50%), Gaps = 49/380 (13%)
 Frame = +1

Query  70    VETQQGLIRGLRSENGKFSKFLGIPYALVDE-KNPFGPSVPHPGFEETFEAYDDSVVCPQ  246
             VE QQG +RG +   G    F  IPYA     K+ F   +P P +  T EA D  VVC Q
Sbjct  40    VEIQQGKLRGYKDPEGGLFTFYNIPYATAPTGKDRFKAPLPAPVWITTREAVDRGVVCHQ  99

Query  247   VIKVTKVGI----GSLQCLNLNVYVPNTATSRNKRPVMVWIHgggfttgsgtsRDYSYDD  414
              I +  VGI         CL  NVYVP+T    +   PV+V++HGG +  G G     +
Sbjct  100   -IDLAAVGIFNKTKQEDCLIANVYVPDT--DKKNLPVVVFVHGGAYLAGFGEV--LTPKH  154

Query  415   LVR-HDVIVVSVNYRLGPYGFLCLDSPDVPGNQELKDQVLALKWTKENIEAFGGDASKIT  591
             +VR ++VI V+ NYRLG  GFLCL +PD PGN  +KD V  L+W K+NI +FGG+   +T
Sbjct  155   MVRTNNVIYVTFNYRLGVQGFLCLGTPDAPGNAGMKDMVALLRWVKKNIASFGGNPDDVT  214

Query  592   IFGESAGGQAVELHLLTDQDK-LFNQVIVQSGS-IYKPGIINKPDNSVPIQIASRLGFET  765
             I G SAG  AV+L LL+   K LF++ I +SG+ +    +   P  +      S  GFE
Sbjct  215   IDGYSAGSSAVDLLLLSKATKGLFHKAIPESGANVAMWSVQVDPIANAKEYAKSEHGFEI  274

Query  766   EDFVAAISFLADQDPHLVVAASTSEGSTVFSGS---AYRACVENEFDGIHSYLSDSPQNL  936
              D + A+       P+  +  S  E   +F       +  CVE +  G+  +L D+P N+
Sbjct  275   LDDIYALEDFYKSLPYDEMYKSGME--LMFKNKNNFLFSPCVERD-TGVEKFLDDTPVNI  331

Query  937   KIK----NIPVIYGVNSKEYLTLHASLVPEQYEVS-GFKSFL-----KLAFNLTDD----  1074
               K     +PV+YG  + E L          + V  G++ F+     K + NL  D
Sbjct  332   LKKGDYNKVPVLYGFANMEGL----------FRVGMGYEYFMSQLNDKFSENLPHDLEFK  381

Query  1075  DMEE------DVRHFYIGDE  1116
             D EE      +V+ FY GD+
Sbjct  382   DEEEKRKVADEVKKFYFGDK  401


> ta_transcript84816_1
Length=947

 Score =   147 bits (371),  Expect = 1e-36, Method: Compositional matrix adjust.
 Identities = 134/380 (35%), Positives = 190/380 (50%), Gaps = 49/380 (13%)
 Frame = +1
```

```
Query  70    VETQQGLIRGLRSENGKFSKFLGIPYALVDE-KNPFGPSVPHPGFEETFEAYDDSVVCPQ  246
             VE QQG +RG +   G    F  IPYA     K+ F   +P P +   T EA D  VVC Q
Sbjct  40    VEIQQGKLRGYKDPEGGLFTFYNIPYATAPTGKDRFKAPLPAPVWITTREAVDRGVVCHQ  99

Query  247   VIKVTKVGIGSLQ----CLNLNVYVPNTATSRNKRPVMVWIHgggfttgsgtsRDYSYDD  414
              I +  VGI +      CL  NVYVP+T    +   PV+V++HGG +  G G      +
Sbjct  100   -IDLAAVGIFNKTKQEDCLIANVYVPDT--DKKNLPVVVFVHGGAYLAGFGEV--LTPKH  154

Query  415   LVR-HDVIVVSVNYRLGPYGFLCLDSPDVPGNQELKDQVLALKWTKENIEAFGGDASKIT  591
             +VR ++VI V+ NYRLG  GFLCL +PD PGN  +KD V  L+W K+NI +FGG+   +T
Sbjct  155   MVRTNNVIYVTFNYRLGVQGFLCLGTPDAPGNAGMKDMVALLRWVKKNIASFGGNPDDVT  214

Query  592   IFGESAGGQAVELHLLTDQDK-LFNQVIVQSGS-IYKPGIINKPDNSVPIQIASRLGFET  765
             I G SAG  AV+L LL+   K LF++ I +SG+ +     +    P  +       S  GFE
Sbjct  215   IDGYSAGSSAVDLLLLSKATKGLFHKAIPESGANVAMWSVQVDPIANAKEYAKSEHGFEI  274

Query  766   EDFVAAISFLADQDPHLVVAASTSEGSTVFSGS---AYRACVENEFDGIHSYLSDSPQNL  936
              D + A+       P+  +  S  E   +F        +  CVE +  G+  +L D+P N+
Sbjct  275   LDDIYALEDFYKSLPYDEMYKSGME--LMFKNKNNFLFSPCVERD-TGVEKFLDDTPVNI  331

Query  937   KIK----NIPVIYGVNSKEYLTLHASLVPEQYEVS-GFKSFL-----KLAFNLTDD----  1074
               K      +PV+YG  + E L           + V  G++ F+      K + NL  D
Sbjct  332   LKKGDYNKVPVLYGFANMEGL----------FRVGMGYEYFMSQLNDKFSENLPHDLEFK  381

Query  1075  DMEE------DVRHFYIGDE  1116
             D EE      +V+ FY GD+
Sbjct  382   DEEEKRKVADEVKKFYFGDK  401


> ta_transcript15565_1
Length=424

 Score =   132 bits (331),  Expect = 8e-33, Method: Compositional matrix adjust.
 Identities = 106/386 (27%), Positives = 182/386 (47%), Gaps = 45/386 (12%)
 Frame = +1

Query  514   LKDQVLALKWTKENIEAFGGDASKITIFGESAGGQAVELHLLTDQDK-LFNQVIVQSGSI  690
             +KD V  L+W   NI AFGG+ + +T+ G SAG  +VEL +L+   K LF++ I +SGS
Sbjct  2     MKDMVALLRWVNRNIAAFGGNPNDVTLEGWSAGSVSVELLMLSKAAKGLFHKAIPESGSS  61

Query  691   YKPGIIN-KPDNSVPIQIASRLGFE-------TEDFVAAISF--LADQDPHLVVAASTSE  840
             + P  +    P  +      S LGF+         E+F    + F  +  Q P ++    + +
Sbjct  62    FAPFSVGVDPIGNAKDFARSDLGFKDVDNISALEEFYKTVPFKDMYQQGPLIIFRHNFT-  120

Query  841   GSTVFSGSAYRACVENEFDGIHSYLSDSPQNLKIK----NIPVIYGVNSKEYLTLHASLV  1008
                      +  CVE     G    +L D+P N+  K      +P++YG ++KE    L+
Sbjct  121   ---------FTPCVERNI-GQDVFLDDTPYNILKKGDYMKVPMLYGFSNKE--GLYRVDY  168

Query  1009  PEQYEVSG----FKSFLKLAFNLTDDDMEEDV----RHFYIGDETLTEELFDEFITFASD  1164
               Q+ + G     F   +L        D++ +E V     + FY G++ + ++    E+I + +D
Sbjct  169   GYQFFIEGMNKKFSDYLPNDLQFKDENEKEQVATMIKEFYFGNKKIDQDTVLEYIDYFTD  228

Query  1165  YYYGYPVERSIKKSLADGNKEIYYYVFSYDGGRNAMKEYLGITAAGVAHADE----LGYL  1332
               +   P   + ++  +A GN +IY Y FSY     N + E + +    G   H   E     L
Sbjct  229   SMFAGPSLKEVQLHVAAGNNQIYLYQFSYP-YENKVPESIKVKVIGSNHCGETMTVLDNW  287

Query  1333  LAVDAVPGQHIAEEDQLIIDRITTLWANFAKYGNPTPEPTDLLPVVWSTVEGNKRPYLDI  1512
             L     +P +   ++ +Q + +   +W NF  K G P P+ ++L    W   +G+   PY+ I
Sbjct  288   LGGAEMPAERNSDLEQ-VRNVTRQIWGNFIKTGKPVPQGSNL--PTWPATDGSGSPYMVI  344

Query  1513  DTDLQLKGRPFH-QRIAFWDLFYKLY  1587
                 +LKG+PF     + FW    Y+ +
Sbjct  345   TNKPELKGQPFQPMSVMFWTEIYQRH  370


***********************************************************************************************
**********************************************************************
Query= gi|512903969|ref|XM_004925690.1| PREDICTED: Bombyx mori juvenile hormone esterase-like
(LOC101737581), mRNA

Length=1422
                                                                      Score     E
Sequences producing significant alignments:                          (Bits)  Value

  ta_transcript75783_1                                                  207    3e-58
```

```
  ta_transcript58915_1                                                197   4e-55
  ta_transcript58914_1                                                197   8e-55
  ta_transcript48580_1                                                196   8e-54
  ta_transcript48579_1                                                196   1e-53
  ta_transcript84818_1                                                161   4e-42
  ta_transcript84821_1                                                160   5e-42
  ta_transcript48393_1                                                159   9e-42
  ta_transcript84816_1                                                160   2e-41
  ta_transcript29751_1                                                142   4e-36
  ta_transcript82623_1                                                122   8e-30


> ta_transcript75783_1
Length=733

 Score =   207 bits (528),  Expect = 3e-58, Method: Compositional matrix adjust.
 Identities = 123/340 (36%), Positives = 183/340 (54%), Gaps = 17/340 (5%)
 Frame = +1

Query  109   VNIDSGPVCGREETAENNTKYYSFQGIPYAKPPVGSKRFAELEPIESWSDPFYAYEEGPA  288
             V +  G + G     E    YYSF+GIP+A+PPVG  RF    P + W     A + GP
Sbjct  126   VKVSEGILQGELVQNEFGGSYYSFKGIPFAEPPVGDLRFKAPIPAKPWKGVKTATKFGPI  185

Query  289   CPSRDLVYGSITVKPKGMSEDCIYVNVFVPATAcldcdclkddllPILVNIHGGTFNTGS  468
                 D+        P   SEDC+Y+NV+ P    +           P++V IHGG +  GS
Sbjct  186   NYQFDV----FKKDPPSGSEDCLYLNVYTPEIKPITLL-------PVMVWIHGGGYFWGS  234

Query  469   GNRDLHGPELLMVKNVIVINFNFRLAVFGYLSLASGKIPGNNSLRDMVTLLQWVQRNARA  648
             GN D++GPE L+   VI++ FN+RL V G+LSL + ++PGN  ++D V  L+WV+ N
Sbjct  235   GNDDVYGPEFLVRHGVILVTFNYRLGVLGFLSLDTAEVPGNAGMKDQVAALKWVKSNIHF  294

Query  649   FGGDPRRVTLLGESSGAASVHLLMLSQAARGLFEKGIIMSGSAMANFYTASPIYAKMIAE  828
             FGGDP  +T+ GES+GA +V L +LS  ++GLF++ II SGSA A +  A  I  K IA
Sbjct  295   FGGDPENITIFGESAGAGAVSLHLLSPMSKGLFKRAIIQSGSATAYWAQAFEIKEKSIA-  353

Query  829   MFLQELGLNCTDPDEIHKTLTELPLEDIMRANDVVQYKTGP---TSFAPVVEIEDHEYTR  999
                ++LGL   D  ++++    +  E ++  +  V +         FAP +E +D    R
Sbjct  354   -LARKLGLVTDDDQKLYEFFGNISKELLINISVPVTFAEQEEVNIEFAPTIE-KDFGQER  411

Query  1000  IIDDDPIALIAQGRAKDIPLLMGFNRDEGEFAKWIIMILD  1119
              + DD   ++ QG  K + ++MG+  DEG      + ILD
Sbjct  412   FLTDDSYKILQQGIHKGVDVMMGYVEDEGVLYLNSLEILD  451


> ta_transcript58915_1
Length=606

 Score =   197 bits (502),  Expect = 4e-55, Method: Compositional matrix adjust.
 Identities = 117/358 (33%), Positives = 187/358 (52%), Gaps = 24/358 (7%)
 Frame = +1

Query  109   VNIDSGPVCGRE-ETAENNTKYYSFQGIPYAKPPVGSKRFAELEPIESWSDPFYAYEEGP  285
             V ++ G + G + ++   + +++SF+GIPYA PPVG  RF   +P  SW     A + GP
Sbjct  32    VQVEQGWLEGEQLDSVSGDGQFFSFKGIPYAAPPVGKLRFKAPQPPVSWQGVRKATQHGP  91

Query  286   ACPSRDLVYGSITVKPKGMSEDCIYVNVFVPATAcldcdclkddllPILVNIHGGTFNTG  465
              CP RD+    I       SEDC+Y+NV+    +            P++V IHGG F +G
Sbjct  92    KCPQRDIFSKQIIPG----SEDCLYLNVYTKDISPKSPL-------PVMVFIHGGGFKSG  140

Query  466   SGNRDLHGPELLMVKNVIVINFNFRLAVFGYLSLASGKIPGNNSLRDMVTLLQWVQRNAR  645
             SG+ D +G + L+   V+++  N+RL   G+L L + ++PGN  L+D V  L+WVQ+N
Sbjct  141   SGDEDFYGGDFLVHHGVVLVTINYRLDALGFLCLDTEEVPGNAGLKDQVAALKWVQKNIF  200

Query  646   AFGGDPRRVTLLGESSGAASVHLLMLSQAARGLFEKGIIMSGSAMANFYTASPIYAKMIA  825
             AFGGDP  VT+ GES+G +S  L +LS  ++GLF++ I MSG    ++  + P   +  A
Sbjct  201   AFGGDPTNVTIFGESAGGSSTALHVLSPLSKGLFKRAIPMSGVPFCDW--SIPFEPRKRA  258

Query  826   EMFLQELGLNCTDPDEIHKTLTELPLEDIMRANDVVQYKTGPT-------SFAPVVEIED  984
                 + LG    DP  + + L  LP E  +  N  +      +        F PVVE ++
Sbjct  259   FTLGKILGFETEDPKALLEYLQSLPAEKFVDTNPTIMGFEEKSYNMLKMYHFTPVVE-KN  317

Query  985   HEYTRIIDDDPIALIAQGRAKDIPLLMGFNRDEGEFAKWIIMILDVVNRYKSNPAIIL  1158
                   + ++P+  + QG   D+ +L+G N D+ E    I   +D++  Y   P + +
Sbjct  318   FGQHHFMTEEPLEALKQGHVNDVDVLIG-NTDQ-ETIVGIPSFVDLLKMYDRYPEVFV  373


> ta_transcript58914_1
```

```
Length=650

 Score =   197 bits (501),  Expect = 8e-55, Method: Compositional matrix adjust.
 Identities = 117/358 (33%), Positives = 187/358 (52%), Gaps = 24/358 (7%)
 Frame = +1

Query  109   VNIDSGPVCGRE-ETAENNTKYYSFQGIPYAKPPVGSKRFAELEPIESWSDPFYAYEEGP  285
             V ++ G + G + ++   + +++SF+GIPYA PPVG  RF   +P  SW     A + GP
Sbjct  32    VQVEQGWLEGEQLDSVSGDGQFFSFKGIPYAAPPVGKLRFKAPQPPVSWQGVRKATQHGP  91

Query  286   ACPSRDLVYGSITVKPKGMSEDCIYVNVFVPATAcldcdclkddllPILVNIHGGTFNTG  465
              CP RD+    I       SEDC+Y+NV+    +            P++V IHGG F +G
Sbjct  92    KCPQRDIFSKQIIPG----SEDCLYLNVYTKDISPKSPL-------PVMVFIHGGGFKSG  140

Query  466   SGNRDLHGPELLMVKNVIVINFNFRLAVFGYLSLASGKIPGNNSLRDMVTLLQWVQRNAR  645
             SG+ D +G + L+   V+++  N+RL   G+L L + ++PGN  L+D V  L+WVQ+N
Sbjct  141   SGDEDFYGGDFLVHHGVVLVTINYRLDALGFLCLDTEEVPGNAGLKDQVAALKWVQKNIF  200

Query  646   AFGGDPRRVTLLGESSGAASVHLLMLSQAARGLFEKGIIMSGSAMANFYTASPIYAKMIA  825
             AFGGDP  VT+ GES+G +S  L +LS  ++GLF++ I MSG    ++  + P   +  A
Sbjct  201   AFGGDPTNVTIFGESAGGSSTALHVLSPLSKGLFKRAIPMSGVPFCDW--SIPFEPRKRA  258

Query  826   EMFLQELGLNCTDPDEIHKTLTELPLEDIMRANDVVQYKTGPT-------SFAPVVEIED  984
                 + LG    DP  + + L  LP E  +  N  +      +        F PVVE ++
Sbjct  259   FTLGKILGFETEDPKALLEYLQSLPAEKFVDTNPTIMGFEEKSYNMLKMYHFTPVVE-KN  317

Query  985   HEYTRIIDDDPIALIAQGRAKDIPLLMGFNRDEGEFAKWIIMILDVVNRYKSNPAIIL  1158
                 + ++P+  + QG   D+ +L+G N D+ E    I   +D++  Y   P + +
Sbjct  318   FGQHHFMTEEPLEALKQGHVNDVDVLIG-NTDQ-ETIVGIPSFVDLLKMYDRYPEVFV  373


> ta_transcript48580_1
Length=834

 Score =   196 bits (497),  Expect = 8e-54, Method: Compositional matrix adjust.
 Identities = 131/359 (36%), Positives = 185/359 (52%), Gaps = 43/359 (12%)
 Frame = +1

Query  109   VNIDSGPVCGREETAEN--NTKYYSFQGIPYAKPPVGSKRFAELEPIESWSDPFYAYEEG  282
             V  + G + GR   A     T Y+SF+GI YA+PP GS RF    P+E WS    A EEG
Sbjct  156   VMTELGALRGRRVVARTTAQTPYFSFKGIRYAQPPRGSLRFKPPVPLEPWSGVRDALEEG  215

Query  283   PACPSRDLVYGSITVKPKGMSEDCIYVNVFVPATAcldcdclkddllPILVNIHGGTFNT  462
               CP R +++ +     KG  EDC+++NV+ PA                ++V IHGG F
Sbjct  216   AVCPHRFMLFDTY----KG-DEDCLFLNVYTPALPEKITGFNPKLA--VMVWIHGGAFAV  268

Query  463   GSGNRDLHGPELLMVKNVIVINFNFRLAVFGYLSLASGKIPGNNSLRDMVTLLQWVQRNA  642
             GSGN  L+GP+ LM  N++++  N+RL   G+LS+ + ++ GN  L+D V  L+WV+ N
Sbjct  269   GSGNAFLYGPDHLMGGNIVLVTLNYRLGALGFLSMENDEVSGNMGLKDQVLALKWVRDNI  328

Query  643   RAFGGDPRRVTLLGESSGAASVHLLMLSQAARGLFEKGIIMSGSAMANF-YTASPIYAKM  819
               FGGD  RVT+ GES+GAASVHL MLS A++GLF + I  SG A++ +   T SP   +
Sbjct  329   EYFGGDASRVTIFGESAGAASVHLHMLSPASKGLFHRAIAQSGLALSPWALTRSP---RE  385

Query  820   IAEMFLQELGLNCTDPDEIHKTLTELPLEDIMRA---------NDVVQYKTGPTSFAPVV  972
              A    +ELG++     E+   L   P E +++A           V  + T    F P V
Sbjct  386   RAFELGRELGIDTNSTAELLGYLRATPSELLVKAGARLAGAPGKSVDLHSTVALPFVPTV  445

Query  973   EIEDHEYTRIIDDDPIALIAQG-----RAKDIPLLMGFNRDEGEFAKWIIMILDVVNRY  1134
             E           D P A + Q         D+PLL G+N  EG      I++    + RY
Sbjct  446   E----------PDVPDAFLTQHPRNLMPGADVPLLTGYNAQEG------IILFRRLQRY  488


> ta_transcript48579_1
Length=905

 Score =   196 bits (497),  Expect = 1e-53, Method: Compositional matrix adjust.
 Identities = 130/354 (37%), Positives = 183/354 (52%), Gaps = 43/354 (12%)
 Frame = +1

Query  124   GPVCGREETAEN--NTKYYSFQGIPYAKPPVGSKRFAELEPIESWSDPFYAYEEGPACPS  297
             G + GR   A     T Y+SF+GI YA+PP GS RF    P+E WS    A EEG  CP
Sbjct  161   GALRGRRVVARTTAQTPYFSFKGIRYAQPPRGSLRFKPPVPLEPWSGVRDALEEGAVCPH  220

Query  298   RDLVYGSITVKPKGMSEDCIYVNVFVPATAcldcdclkddllPILVNIHGGTFNTGSGNR  477
             R +++ +     KG  EDC+++NV+ PA                ++V IHGG F  GSGN
Sbjct  221   RFMLFDTY----KG-DEDCLFLNVYTPALPEKITGFNPKLA--VMVWIHGGAFAVGSGNA  273
```

```
Query  478   DLHGPELLMVKNVIVINFNFRLAVFGYLSLASGKIPGNNSLRDMVTLLQWVQRNARAFGG  657
              L+GP+ LM  N++++  N+RL   G+LS+ + ++ GN  L+D V  L+WV+ N   FGG
Sbjct  274   FLYGPDHLMGGNIVLVTLNYRLGALGFLSMENDEVSGNMGLKDQVLALKWVRDNIEYFGG  333

Query  658   DPRRVTLLGESSGAASVHLLMLSQAARGLFEKGIIMSGSAMANF-YTASPIYAKMIAEMF  834
             D  RVT+ GES+GAASVHL MLS A++GLF + I  SG A++ +  T SP   +  A
Sbjct  334   DASRVTIFGESAGAASVHLHMLSPASKGLFHRAIAQSGLALSPWALTRSP---RERAFEL  390

Query  835   LQELGLNCTDPDEIHKTLTELPLEDIMRA---------NDVVQYKTGPTSFAPVVEIEDH  987
              +ELG++     E+   L   P E +++A           V  + T    F P VE
Sbjct  391   GRELGIDTNSTAELLGYLRATPSELLVKAGARLAGAPGKSVDLHSTVALPFVPTVE----  446

Query  988   EYTRIIDDDPIALIAQG-----RAKDIPLLMGFNRDEGEFAKWIIMILDVVNRY  1134
                    D P A + Q         D+PLL G+N  EG      I++   + RY
Sbjct  447   ------PDVPDAFLTQHPRNLMPGADVPLLTGYNAQEG------IILFRRLQRY  488


> ta_transcript84818_1
Length=733

 Score =   161 bits (407),  Expect = 4e-42, Method: Compositional matrix adjust.
 Identities = 109/314 (35%), Positives = 163/314 (52%), Gaps = 25/314 (8%)
 Frame = +1

Query  172   YSFQGIPYAKPPVGSKRFAELEPIESWSDPFYAYEEGPACPSRDLVYGSITVKPKGMSED  351
             ++F  IPYA  P G  RF    P   W     A + G  C   DL    I  K K   ED
Sbjct  58    FTFYNIPYATAPTGKDRFKAPLPAPVWITTREAVDRGVVCHQIDLAAVGIFNKTK--QED  115

Query  352   CIYVNVFVPATAcldcdclkddllPILVNIHGGTFNTGSGNRDLHGPELLMVKNVIVINF  531
             C+  NV+VP T            LP++V +HGG +  G G   L    ++   NVI + F
Sbjct  116   CLIANVYVPDT--------DKKNLPVVVFVHGGAYLAGFGEV-LTPKHMVRTNNVIYVTF  166

Query  532   NFRLAVFGYLSLASGKIPGNNSLRDMVTLLQWVQRNARAFGGDPRRVTLLGESSGAASVH  711
             N+RL V G+L L +   PGN  ++DMV LL+WV++N  +FGG+P  VT+ G S+G+++V
Sbjct  167   NYRLGVQGFLCLGTPDAPGNAGMKDMVALLRWVKKNIASFGGNPDDVTIDGYSAGSSAVD  226

Query  712   LLMLSQAARGLFEKGIIMSGSAMANF-YTASPIY-AKMIAEMFLQELGLNCTDP----DE  873
             LL+LS+A +GLF K I  SG+ +A +     PI  AK  A+    E G    D     ++
Sbjct  227   LLLLSKATKGLFHKAIPESGANVAMWSVQVDPIANAKEYAK---SEHGFEILDDIYALED  283

Query  874   IHKTLTELPLEDIMRANDVVQYKTGPTS-FAPVVEIEDHEYTRIIDDDPIALIAQGRAKD  1050
              +K+   LP +++ ++   + +K      F+P VE  D    + +DD P+ ++ +G
Sbjct  284   FYKS---LPYDEMYKSGMELMFKNKNNFLFSPCVE-RDTGVEKFLDDTPVNILKKGDYNK  339

Query  1051  IPLLMGFNRDEGEF  1092
             +P+L GF   EG F
Sbjct  340   VPVLYGFANMEGLF  353


> ta_transcript84821_1
Length=611

 Score =   160 bits (404),  Expect = 5e-42, Method: Compositional matrix adjust.
 Identities = 109/314 (35%), Positives = 163/314 (52%), Gaps = 25/314 (8%)
 Frame = +1

Query  172   YSFQGIPYAKPPVGSKRFAELEPIESWSDPFYAYEEGPACPSRDLVYGSITVKPKGMSED  351
             ++F  IPYA  P G  RF    P   W     A + G  C   DL    I  K K   ED
Sbjct  58    FTFYNIPYATAPTGKDRFKAPLPAPVWITTREAVDRGVVCHQIDLAAVGIFNKTK--QED  115

Query  352   CIYVNVFVPATAcldcdclkddllPILVNIHGGTFNTGSGNRDLHGPELLMVKNVIVINF  531
             C+  NV+VP T            LP++V +HGG +  G G   L    ++   NVI + F
Sbjct  116   CLIANVYVPDT--------DKKNLPVVVFVHGGAYLAGFGEV-LTPKHMVRTNNVIYVTF  166

Query  532   NFRLAVFGYLSLASGKIPGNNSLRDMVTLLQWVQRNARAFGGDPRRVTLLGESSGAASVH  711
             N+RL V G+L L +   PGN  ++DMV LL+WV++N  +FGG+P  VT+ G S+G+++V
Sbjct  167   NYRLGVQGFLCLGTPDAPGNAGMKDMVALLRWVKKNIASFGGNPDDVTIDGYSAGSSAVD  226

Query  712   LLMLSQAARGLFEKGIIMSGSAMANF-YTASPIY-AKMIAEMFLQELGLNCTDP----DE  873
             LL+LS+A +GLF K I  SG+ +A +     PI  AK  A+    E G    D     ++
Sbjct  227   LLLLSKATKGLFHKAIPESGANVAMWSVQVDPIANAKEYAK---SEHGFEILDDIYALED  283

Query  874   IHKTLTELPLEDIMRANDVVQYKTGPTS-FAPVVEIEDHEYTRIIDDDPIALIAQGRAKD  1050
              +K+   LP +++ ++   + +K      F+P VE  D    + +DD P+ ++ +G
Sbjct  284   FYKS---LPYDEMYKSGMELMFKNKNNFLFSPCVE-RDTGVEKFLDDTPVNILKKGDYNK  339
```

```
Query  1051  IPLLMGFNRDEGEF  1092
             +P+L GF   EG F
Sbjct  340   VPVLYGFANMEGLF  353


> ta_transcript48393_1
Length=666

 Score =   159 bits (403),  Expect = 9e-42, Method: Compositional matrix adjust.
 Identities = 113/356 (32%), Positives = 167/356 (47%), Gaps = 27/356 (8%)
 Frame = +1

Query  43    KHICKFITAVYGEFCITECEVP----VNIDSGPVCGREETAENNTKYYSFQGIPYAKPPV  210
             K I   I+   G F     + P    V    GPV G  +        ++F  IPYA  P
Sbjct  70    KTITLVISLCLGLFSTVSGDEPRSRIVETTQGPVRGYRDPEGG---LFAFYSIPYATAPS  126

Query  211   GSKRFAELEPIESWSDPFYAYEEGPACPSRDLVYGSITVKPKGMSEDCIYVNVFVPATAc  390
             G  +F       +W++PF A ++   CP       S   K   M  DC+  NV+VP T
Sbjct  127   GPLKFTAPITPPTWTEPFEAVDKRIICPQMK----SGFTKQMTMQVDCLIANVYVPDTKE  182

Query  391   ldcdclkddllPILVNIHGGTFNTGSGNRDLHGPE-LLMVKNVIVINFNFRLAVFGYLSL  567
                        P++V +HGG F  G GN  L  P+ L+  KN+I + FN+RL   G+L L
Sbjct  183   TKL--------PVIVYVHGGAFQVGFGN--LMTPKNLVRSKNIIAVTFNYRLGAHGFLCL  232

Query  568   ASGKIPGNNSLRDMVTLLQWVQRNARAFGGDPRRVTLLGESSGAASVHLLMLSQAARGLF  747
              +   PGN  L+D V  L+WV +N    GG+P  VT+ G S+GA SV L+ +S++A GLF
Sbjct  233   GTEDAPGNAGLKDQVAALRWVNKNIIKLGGNPDDVTIAGYSAGAISVDLMAISKSAEGLF  292

Query  748   EKGIIMSGSAM-ANFYTASPIYAKMIAEMFLQELGLNCTDPDEIHKTLTELPLEDIMRAN  924
              K II SGS++ A      P+     A++F   LG N  D     +   +    D +
Sbjct  293   SKIIIESGSSLFAGSIVTDPLEN---AKIFAARLGFNNIDDVYALEKFYKAASYDSLTKG  349

Query  925   DVVQYKTGPTSFAPVVEIEDHEYTRIIDDDPIALIAQGRAKDIPLLMGFNRDEGEF  1092
             + +        F+P VE  D +    + + PI ++ +G    +P++ GF+  EG F
Sbjct  350   EFMDRPNSTLLFSPCVE-RDDDGLAFLSEPPINILKEGSYTQVPMIYGFSNMEGLF  404


> ta_transcript84816_1
Length=947

 Score =   160 bits (406),  Expect = 2e-41, Method: Compositional matrix adjust.
 Identities = 107/314 (34%), Positives = 164/314 (52%), Gaps = 25/314 (8%)
 Frame = +1

Query  172   YSFQGIPYAKPPVGSKRFAELEPIESWSDPFYAYEEGPACPSRDLVYGSITVKPKGMSED  351
             ++F  IPYA  P G  RF    P   W     A + G  C   DL   ++ +  K   ED
Sbjct  58    FTFYNIPYATAPTGKDRFKAPLPAPVWITTREAVDRGVVCHQIDL--AAVGIFNKTKQED  115

Query  352   CIYVNVFVPATAcldcdclkddllPILVNIHGGTFNTGSGNRDLHGPELLMVKNVIVINF  531
             C+  NV+VP T            LP++V +HGG +  G G   L    ++   NVI + F
Sbjct  116   CLIANVYVPDT--------DKKNLPVVVFVHGGAYLAGFGEV-LTPKHMVRTNNVIYVTF  166

Query  532   NFRLAVFGYLSLASGKIPGNNSLRDMVTLLQWVQRNARAFGGDPRRVTLLGESSGAASVH  711
             N+RL V G+L L +   PGN  ++DMV LL+WV++N  +FGG+P  VT+ G S+G+++V
Sbjct  167   NYRLGVQGFLCLGTPDAPGNAGMKDMVALLRWVKKNIASFGGNPDDVTIDGYSAGSSAVD  226

Query  712   LLMLSQAARGLFEKGIIMSGSAMANF-YTASPIY-AKMIAEMFLQELGLNCTDP----DE  873
             LL+LS+A +GLF K I  SG+ +A +     PI  AK  A+    E G    D     ++
Sbjct  227   LLLLSKATKGLFHKAIPESGANVAMWSVQVDPIANAKEYAK---SEHGFEILDDIYALED  283

Query  874   IHKTLTELPLEDIMRANDVVQYKTGPTS-FAPVVEIEDHEYTRIIDDDPIALIAQGRAKD  1050
              +K+   LP +++ ++   + +K      F+P VE  D    + +DD P+ ++ +G
Sbjct  284   FYKS---LPYDEMYKSGMELMFKNKNNFLFSPCVE-RDTGVEKFLDDTPVNILKKGDYNK  339

Query  1051  IPLLMGFNRDEGEF  1092
              +P+L GF   EG F
Sbjct  340   VPVLYGFANMEGLF  353


> ta_transcript29751_1
Length=506

 Score =   142 bits (357),  Expect = 4e-36, Method: Compositional matrix adjust.
 Identities = 122/350 (35%), Positives = 184/350 (53%), Gaps = 34/350 (10%)
 Frame = +1

Query  109   VNIDSGPVCGREETAENNTKYYSFQGIPYAKPPVGSKRFAELEPIESWSDPFYAYEEGP  285
```

```
             V I+ GPV G ++    +   Y F  IPYA  P    RF A L P   W     A +
Sbjct  33    VVIEQGPVRGYKDP---DGGLYVFYNIPYATAPTSKDRFKAPLAP-PVWVTIRDAVDREV  88

Query  286   ACPSRDLVYGSITVKPKGMSEDCIYVNVFVPATAcldcdclkddllPILVNIHGGTFNTG  465
              CP   L  GS     K + EDC+  NVFVP T+  +          ++V +HGG + +G
Sbjct  89    VCPQM-LETGS----HKNIQEDCLIANVFVPDTSEKNLS--------VVVLVHGGGYLSG  135

Query  466   SGNRDLHGPELLMVKNVIVINFNFRLAVFGYLSLASGKIPGNNSLRDMVTLLQWVQRNAR  645
              GNR     +++  K +I + FN+RL V G+L L +  +PGN  ++DMV LL+WV++N
Sbjct  136   FGNRQT-PKDMVRDKKLIYVTFNYRLGVHGFLCLGTADVPGNAGMKDMVALLRWVKKNIA  194

Query  646   AFGGDPRRVTLLGESSGAASVHLLMLSQAARGLFEKGIIMSGSAMANFYTA-SPI-YAKM  819
             +FGG+P  VT+ G SSG++SV LL+LS+A  GLF K I  SGS +A +     PI +AK
Sbjct  195   SFGGNPDDVTIDGHSSGSSSVDLLLLSKATEGLFHKAIPESGSNVAMWSVQIDPIAHAKE  254

Query  820   IA-EMFLQELGLNCTDPDEIHKTLTELPLEDIMRANDVVQYKTGPTS--FAPVVEIEDHE  990
              A +    E G +    ++ +KT   LP + + ++   ++Y +   +  F P VE  D
Sbjct  255   FARDEHSFESGDDIYALEDFYKT---LPFDVMYKSGMSLRYNSSVKNILFQPCVE-RDTG  310

Query  991   YTRIIDDDPIALIAQGRAKDIPLLMGFNRDEGEFAKWIIMILDVVNRYKS  1140
               + +DD P+ ++  G    +P+L GF   EG     ++ + D VN Y+S
Sbjct  311   VEKFLDDFPVNILKSGDYNKVPMLYGFTNMEG-----LMRVSDFVN-YRS  354


> ta_transcript82623_1
Length=454

 Score =   122 bits (307),  Expect = 8e-30, Method: Compositional matrix adjust.
 Identities = 106/285 (37%), Positives = 131/285 (46%), Gaps = 39/285 (14%)
 Frame = -2

Query  836  RNISAIILAYIGDAV*KFAIAEPLIIIPFSKSPLAAWDNIKR*TDAAPELSPSNVTRLGS  657
            R +SA   A IGD V*    A PLI+I   KSP A  D++ + T  AP L P+ VT  GS
Sbjct  129  R*LSAAYFAKIGDVV*NKGSAVPLIMIALWKSPCAGGDSVSKCTADAPAL*PARVTSSGS  188

Query  656  PPKARAFL*THCNSVTMSRRLLLPGILPEARDKYPNTANLKLKLITMTFFTINNSGPCKS  477
            PPK  A   T  + V MSRR LLPG L   R++ PNT NL L ++TMT F+   SGPC S
Sbjct  189  PPKLLALSRTQRSKVIMSRRPLLPGTLVVFRERKPNTFNL*LNVMTMTPFSTKCSGPCTS  248

Query  476  LFPDPVLNVPP*MFTSIGS-----------------------------------RSSLRQ  402
              P+P    PP   T IG                                    R+S+ +
Sbjct  249  SSPEPNAKPPPCTNTRIGRPLVK*DSSGMFLLYRTRGVSVSTSLTGRPWLVIVVRASVIE  308

Query  401  SQSKHAVAGTKTLT*IQSSLMPLGFTVMLP*TRSLDGQAGPSS*A*NGSLHDSIGSSSAN  222
             +   A+ GT  L  +  SLMP+G  +  P*  S  GQ P    A N    DS G SS N
Sbjct  309  GR---ALKGTWMLAKMHDSLMPVGV-IRGP*KTSSCGQTLPGLVASNIPCQDSNGFSSWN  364

Query  221  LLDPTGGFAYGIP*KL*YFVLFSAVSSLPHTGPESIFTGTSHSVM  87
            L  P GG A G P K  Y          P T P  +  GT+H ++
Sbjct  365  LRGPKGGSA*GTPLKDAYCRPEGVTRRAPQT*PALVRKGTAHDLV  409


************************************************************************************************
**********************************************************************

Query= gi|512931402|ref|XM_004932237.1| PREDICTED: Bombyx mori juvenile hormone esterase-like
(LOC101736068), mRNA

Length=1395
                                                                      Score     E
Sequences producing significant alignments:                          (Bits)  Value

  ta_transcript48580_1                                                  234    3e-67
  ta_transcript48579_1                                                  233    7e-67
  ta_transcript75783_1                                                  192    1e-52
  ta_transcript58915_1                                                  189    5e-52
  ta_transcript58914_1                                                  189    6e-52
  ta_transcript84821_1                                                  169    2e-45
  ta_transcript84818_1                                                  170    3e-45
  ta_transcript84816_1                                                  170    2e-44
  ta_transcript29751_1                                                  150    4e-39
  ta_transcript48393_1                                                  150    1e-38
  ta_transcript15565_1                                                  124    2e-30


> ta_transcript48580_1
Length=834
```

```
 Score =   234 bits (596),  Expect = 3e-67, Method: Compositional matrix adjust.
 Identities = 148/415 (36%), Positives = 218/415 (53%), Gaps = 21/415 (5%)
 Frame = +1

Query  7     EDCLRINVYVPVKKTNTS----HLPVFAFIHGGAYRILSKDSDVIYDPYFFMKKGIIVVT  174
             EDCL +NVY P      +     L V  +IHGGA+ + S ++  +Y P   M   I++VT
Sbjct  232   EDCLFLNVYTPALPEKITGFNPKLAVMVWIHGGAFAVGSGNA-FLYGPDHLMGGNIVLVT  290

Query  175   MNYRLGALGFLSLKTPGASGNNGLKDL-----WVKENIGKFGGDSTKITVGGNSSGSSMA  339
             +NYRLGALGFLS++    SGN GLKD      WV++NI  FGGD++++T+ G S+G++
Sbjct  291   LNYRLGALGFLSMENDEVSGNMGLKDQVLALKWVRDNIEYFGGDASRVTIFGESAGAASV  350

Query  340   NYLMLSRKTTGLIHRVIMFSGSALNFRFLQRYPIENARELASGLGLSINDSDVLLQNLKT  519
             +  MLS  + GL HR I  SG AL+   L R P E A EL   LG+  N +  LL  L+
Sbjct  351   HLHMLSPASKGLFHRAIAQSGLALSPWALTRSPRERAFELGRELGIDTNSTAELLGYLRA  410

Query  520   VNPFDIVDAQENMY---KKSRNPIITFA-PFVPTIEPDSADAFLTEDPTSMVKKGLIQNV  687
                  +V A   +     KS +   T A PFVPT+EPD  DAFLT+ P +++      +V
Sbjct  411   TPSELLVKAGARLAGAPGKSVDLHSTVALPFVPTVEPDVPDAFLTQHPRNLMPGA---DV  467

Query  688   PVLAGINSLEGITFYHKIVNNKTLLNDLENNIHLTVPNDIEYPRNSTEFRNLVQSIKELY  867
             P+L G N+ EGI  + ++     LL +L+      VP ++    +  + R +   I+  Y
Sbjct  468   PLLTGYNAQEGIILFRRLQRYPKLLTELDREFRRVVPPEL-LTGDEAQNRRVADHIRAFY  526

Query  868   FNS--TDTSALNKYFRIFSDIAFTHKVDYFIYLHKRNPQSNKVFYYEFDFDGDLNWGKLY  1041
             F     D   ++    +F+D+ F   +   + +  +  +++  + Y F FDG L   K
Sbjct  527   FQQRPVDIRNIDSLIDLFTDVMFLRPLLETLRIQGKTNRTSPTYLYRFAFDGALGLFKRM  586

Query  1042  HDIQYPGTCHSDELGYLFVTNATKENLKTLDNKSRHVLNIMVSVMSNFIKFGNPT  1206
               I +PG CH DE+GYLF  +     L   D+    V   MV + +NF K GNPT
Sbjct  587   LGINHPGACHGDEMGYLFYFSRLNYRLDD-DSTELAVSKGMVRMWTNFAKTGNPT  640


> ta_transcript48579_1
Length=905

 Score =   233 bits (595),  Expect = 7e-67, Method: Compositional matrix adjust.
 Identities = 148/415 (36%), Positives = 218/415 (53%), Gaps = 21/415 (5%)
 Frame = +1

Query  7     EDCLRINVYVPVKKTNTS----HLPVFAFIHGGAYRILSKDSDVIYDPYFFMKKGIIVVT  174
             EDCL +NVY P      +     L V  +IHGGA+ + S ++  +Y P   M   I++VT
Sbjct  232   EDCLFLNVYTPALPEKITGFNPKLAVMVWIHGGAFAVGSGNA-FLYGPDHLMGGNIVLVT  290

Query  175   MNYRLGALGFLSLKTPGASGNNGLKDL-----WVKENIGKFGGDSTKITVGGNSSGSSMA  339
             +NYRLGALGFLS++    SGN GLKD      WV++NI  FGGD++++T+ G S+G++
Sbjct  291   LNYRLGALGFLSMENDEVSGNMGLKDQVLALKWVRDNIEYFGGDASRVTIFGESAGAASV  350

Query  340   NYLMLSRKTTGLIHRVIMFSGSALNFRFLQRYPIENARELASGLGLSINDSDVLLQNLKT  519
             +  MLS  + GL HR I  SG AL+   L R P E A EL   LG+  N +  LL  L+
Sbjct  351   HLHMLSPASKGLFHRAIAQSGLALSPWALTRSPRERAFELGRELGIDTNSTAELLGYLRA  410

Query  520   VNPFDIVDAQENMY---KKSRNPIITFA-PFVPTIEPDSADAFLTEDPTSMVKKGLIQNV  687
                  +V A   +     KS +   T A PFVPT+EPD  DAFLT+ P +++      +V
Sbjct  411   TPSELLVKAGARLAGAPGKSVDLHSTVALPFVPTVEPDVPDAFLTQHPRNLMPGA---DV  467

Query  688   PVLAGINSLEGITFYHKIVNNKTLLNDLENNIHLTVPNDIEYPRNSTEFRNLVQSIKELY  867
             P+L G N+ EGI  + ++     LL +L+      VP ++    +  + R +   I+  Y
Sbjct  468   PLLTGYNAQEGIILFRRLQRYPKLLTELDREFRRVVPPEL-LTGDEAQNRRVADHIRAFY  526

Query  868   FNS--TDTSALNKYFRIFSDIAFTHKVDYFIYLHKRNPQSNKVFYYEFDFDGDLNWGKLY  1041
             F     D   ++    +F+D+ F   +   + +  +  +++  + Y F FDG L   K
Sbjct  527   FQQRPVDIRNIDSLIDLFTDVMFLRPLLETLRIQGKTNRTSPTYLYRFAFDGALGLFKRM  586

Query  1042  HDIQYPGTCHSDELGYLFVTNATKENLKTLDNKSRHVLNIMVSVMSNFIKFGNPT  1206
               I +PG CH DE+GYLF  +     L   D+    V   MV + +NF K GNPT
Sbjct  587   LGINHPGACHGDEMGYLFYFSRLNYRLDD-DSTELAVSKGMVRMWTNFAKTGNPT  640


> ta_transcript75783_1
Length=733

 Score =   192 bits (487),  Expect = 1e-52, Method: Compositional matrix adjust.
 Identities = 145/466 (31%), Positives = 233/466 (50%), Gaps = 33/466 (7%)
 Frame = +1
```

```
Query  7     EDCLRINVYVPVKKTNTSHLPVFAFIHGGAYRILSKDSDVIYDPYFFMKKGIIVVTMNYR  186
             EDCL +NVY P  K  T  LPV  +IHGG Y   S + DV Y P F ++ G+I+VT NYR
Sbjct  201   EDCLYLNVYTPEIKPITL-LPVMVWIHGGGYFWGSGNDDV-YGPEFLVRHGVILVTFNYR  258

Query  187   LGALGFLSLKTPGASGNNGLKDL-----WVKENIGKFGGDSTKITVGGNSSGSSMANYLM  351
             LG LGFLSL T    GN G+KD      WVK NI  FGGD   IT+ G S+G+   +  +
Sbjct  259   LGVLGFLSLDTAEVPGNAGMKDQVAALKWVKSNIHFFGGDPENITIFGESAGAGAVSLHL  318

Query  352   LSRKTTGLIHRVIMFSGSALNFRFLQRYPI-ENARELASGLGLSINDSDVLLQNLKTVNP  528
             LS  + GL  R I+ SGSA  + + Q + I E +  LA  LGL  +D   L +    ++
Sbjct  319   LSPMSKGLFKRAIIQSGSATAY-WAQAFEIKEKSIALARKLGLVTDDDQKLYEFFGNISK  377

Query  529   FDIVDAQENMYKKSRNPI-ITFAPFVPTIEPD-SADAFLTEDPTSMVKKGLIQNVPVLAG  702
               +++    +    +  + I FA    PTIE D   + FLT+D   ++++G+ + V V+ G
Sbjct  378   ELLINISVPVTFAEQEEVNIEFA---PTIEKDFGQERFLTDDSYKILQQGIHKGVDVMMG  434

Query  703   INSLEGITFYHKIVNNKTLLNDLENNIHLTVPNDIEYPRNSTEFRNLVQSIKELYFNST-  879
                 EG+ + + +      +  + N +   VP +++      +   + + +K+ YF++
Sbjct  435   YVEDEGVLYLNSLEILDKEIERINNFLEALVPRNMKREIPIKQQLKVGRKMKKFYFDNKL  494

Query  880   ----DTSALNKYFRIFSDIAFTHKVDYFIYLHKRNPQSNKVFYYEFDFDGDLN-WGKLYH  1044
                 D   + +Y  +     F++ V   I   K   +S   F+Y+F      N + K++
Sbjct  495   VTRNDWRTIARYISL---NVFSYDV---IQSQKNIAKSQNSFFYKFTCKSKRNFFKKVFT  548

Query  1045  DIQYPG----TCHSDELGYLFVTNATKENLKTLDNKSRHVLNIMVSVMSNFIKFGNPTPS  1212
             D +        CH D+L YLF      +++   D++   +++ +  + +NF K+GNPTP
Sbjct  549   DDEITKDVDVVCHCDDLAYLFPVKFMNQSVAK-DSREFQLIDKVTKLWTNFAKYGNPTPD  607

Query  1213  CDMGICWTESNCSRNHIV-LKEIPVLKNNSPYPERFTFWSEVYKQF  1347
               +G+ W         H + + E  VLK   P  E   FW E+Y+++
Sbjct  608   NSLGVKWEPFTLQHQHYLDIGEDLVLK-TKPDDEDIQFWEELYREY  652
```

> ta_transcript58915_1
Length=606

Score =   189 bits (479), Expect = 5e-52, Method: Compositional matrix adjust.
Identities = 143/458 (31%), Positives = 215/458 (47%), Gaps = 20/458 (4%)
Frame = +1

```
Query  7     EDCLRINVYVPVKKTNTSHLPVFAFIHGGAYRILSKDSDVIYDPYFFMKKGIIVVTMNYR  186
             EDCL +NVY     +  S LPV  FIHGG ++  S D D  Y    F +  G+++VT+NYR
Sbjct  108   EDCLYLNVYTK-DISPKSPLPVMVFIHGGGFKSGSGDED-FYGGDFLVHHGVVLVTINYR  165

Query  187   LGALGFLSLKTPGASGNNGLKDL-----WVKENIGKFGGDSTKITVGGNSSGSSMANYLM  351
             L ALGFL L T    GN GLKD      WV++NI  FGGD T +T+ G S+G S     +
Sbjct  166   LDALGFLCLDTEEVPGNAGLKDQVAALKWVQKNIFAFGGDPTNVTIFGESAGGSSTALHV  225

Query  352   LSRKTTGLIHRVIMFSGSALNFRFLQRYPIENARELASGLGLSINDSDVLLQNLKTVNPF  531
             LS  + GL  R I  SG        +    P + A  L   LG    D   LL+ L+++
Sbjct  226   LSPLSKGLFKRAIPMSGVPFCDWSIPFEPRKRAFTLGKILGFETEDPKALLEYLQSLPAE  285

Query  532   DIVDAQENMY---KKSRNPIITFAPFVPTIEPD-SADAFLTEDPTSMVKKGLIQNVPVLA  699
               VD    +    +KS N ++    F P +E +     F+TE+P   +K+G + +V VL
Sbjct  286   KFVDTNPTIMGFEEKSYN-MLKMYHFTPVVEKNFGQHHFMTEEPLEALKQGHVNDVDVLI  344

Query  700   GINSLEGITFYHKIVNNKTLLNDLENNIHLTVPNDIEYPRNSTEFRNLVQSIKELYFNST  879
             G    E I       V+   LL    +    + VP  I        +    + + I++ YF
Sbjct  345   GNTDQETIVGIPSFVD---LLKMYDRYPEVFVPRKILNKSTPGKILEIAERIRKHYFGDK  401

Query  880   --DTSALNKYFRIFSDIAFTHKVD-YFIYLHKRNPQSNKVFYYEFDFDGDLN-WGKLYHD  1047
               D +++ +     FS++ FT+ V+ Y    L    P S+KV+ Y F    + N +GK   +
Sbjct  402   PIDKTSMKEAVTYFSEVCFTYDVNKYTNLLLSGKPGSSKVYRYRFSCVSERNIFGKYGQE  461

Query  1048  IQYPGTCHSDELGYLFVTNATKENLKTLDNKSRHVLNIMVSVMSNFIKFGNPTPSCDMGI  1227
                 G  H ++L YLF    + K  L   ++KS  ++    ++ +NF K+GNPTP   +G
Sbjct  462   YGITGASHLEDLMYLFDAKSVKLPLDR-NSKSYKMIQQTCALFTNFAKYGNPTPDSSLGF  520

Query  1228  CWTESNCSRNHIVLKEIPVLKNNSPYPERFTFWSEVYK  1341
              W E +      V      +        +   FW  +Y+
Sbjct  521   TWPEYDIKDQSFVDIADQLTVGRHLDADAVKFWESIYQ  558
```

> ta_transcript58914_1
Length=650

Score =   189 bits (480), Expect = 6e-52, Method: Compositional matrix adjust.

```
 Identities = 143/458 (31%), Positives = 215/458 (47%), Gaps = 20/458 (4%)
 Frame = +1

Query  7     EDCLRINVYVPVKKTNTSHLPVFAFIHGGAYRILSKDSDVIYDPYFFMKKGIIVVTMNYR  186
             EDCL +NVY     +  S LPV  FIHGG ++  S D D  Y   F +  G+++VT+NYR
Sbjct  108   EDCLYLNVYTK-DISPKSPLPVMVFIHGGGFKSGSGDED-FYGGDFLVHHGVVLVTINYR  165

Query  187   LGALGFLSLKTPGASGNNGLKDL-----WVKENIGKFGGDSTKITVGGNSSGSSMANYLM  351
             L ALGFL L T    GN GLKD      WV++NI  FGGD T +T+ G S+G S     +
Sbjct  166   LDALGFLCLDTEEVPGNAGLKDQVAALKWVQKNIFAFGGDPTNVTIFGESAGGSSTALHV  225

Query  352   LSRKTTGLIHRVIMFSGSALNFRFLQRYPIENARELASGLGLSINDSDVLLQNLKTVNPF  531
             LS  + GL  R I  SG       +   P + A  L   LG    D   LL+ L+++
Sbjct  226   LSPLSKGLFKRAIPMSGVPFCDWSIPFEPRKRAFTLGKILGFETEDPKALLEYLQSLPAE  285

Query  532   DIVDAQENMY---KKSRNPIITFAPFVPTIEPD-SADAFLTEDPTSMVKKGLIQNVPVLA  699
               VD    +    +KS N ++    F P +E +     F+TE+P   +K+G + +V VL
Sbjct  286   KFVDTNPTIMGFEEKSYN-MLKMYHFTPVVEKNFGQHHFMTEEPLEALKQGHVNDVDVLI  344

Query  700   GINSLEGITFYHKIVNNKTLLNDLENNIHLTVPNDIEYPRNSTEFRNLVQSIKELYFNST  879
             G    E I       V+   LL   +    + VP  I        +   + + I++ YF
Sbjct  345   GNTDQETIVGIPSFVD---LLKMYDRYPEVFVPRKILNKSTPGKILEIAERIRKHYFGDK  401

Query  880   --DTSALNKYFRIFSDIAFTHKVD-YFIYLHKRNPQSNKVFYYEFDFDGDLN-WGKLYHD  1047
               D +++ +    FS++ FT+ V+ Y   L    P S+KV+ Y F    + N +GK   +
Sbjct  402   PIDKTSMKEAVTYFSEVCFTYDVNKYTNLLLSGKPGSSKVYRYRFSCVSERNIFGKYGQE  461

Query  1048  IQYPGTCHSDELGYLFVTNATKENLKTLDNKSRHVLNIMVSVMSNFIKFGNPTPSCDMGI  1227
                 G  H ++L YLF   + K  L   ++KS  ++    ++ +NF K+GNPTP   +G
Sbjct  462   YGITGASHLEDLMYLFDAKSVKLPLDR-NSKSYKMIQQTCALFTNFAKYGNPTPDSSLGF  520

Query  1228  CWTESNCSRNHIVLKEIPVLKNNSPYPERFTFWSEVYK  1341
              W E +      V     +        +   FW  +Y+
Sbjct  521   TWPEYDIKDQSFVDIADQLTVGRHLDADAVKFWESIYQ  558


> ta_transcript84821_1
Length=611

 Score =   169 bits (429),  Expect = 2e-45, Method: Compositional matrix adjust.
 Identities = 109/297 (37%), Positives = 163/297 (55%), Gaps = 21/297 (7%)
 Frame = +1

Query  7    EDCLRINVYVPVKKTNTSHLPVFAFIHGGAYRILSKDSDVIYDPYFFMKKGIIVVTMNYR  186
            EDCL  NVYVP   T+  +LPV  F+HGGAY  L+   +V+   +      +I VT NYR
Sbjct  114  EDCLIANVYVP--DTDKKNLPVVVFVHGGAY--LAGFGEVLTPKHMVRTNNVIYVTFNYR  169

Query  187  LGALGFLSLKTPGASGNNGLKDL-----WVKENIGKFGGDSTKITVGGNSSGSSMANYLM  351
            LG  GFL L TP A GN G+KD+     WVK+NI  FGG+   +T+ G S+GSS  + L+
Sbjct  170  LGVQGFLCLGTPDAPGNAGMKDMVALLRWVKKNIASFGGNPDDVTIDGYSAGSSAVDLLL  229

Query  352  LSRKTTGLIHRVIMFSGSALNFRFLQRYPIENARELA-SGLGLSINDSDVLLQNLKTVNP  528
            LS+ T GL H+ I  SG+ +     +Q  PI NA+E A S  G  I D    L++     P
Sbjct  230  LSKATKGLFHKAIPESGANVAMWSVQVDPIANAKEYAKSEHGFEILDDIYALEDFYKSLP  289

Query  529  FDIV--DAQENMYKKSRNPIITFAPFVPTIEPDSA-DAFLTEDPTSMVKKGLIQNVPVLA  699
            +D +     E M+K   N +     F P +E D+  + FL + P +++KKG    VPVL
Sbjct  290  YDEMYKSGMELMFKNKNNFL-----FSPCVERDTGVEKFLDDTPVNILKKGDYNKVPVLY  344

Query  700  GINSLEGITFYHKIVNNKTLLNDLENNIHLTVPNDIEYPRNSTEFRNLVQSIKELYF  870
            G  ++EG+  +    +  +  ++ L +     +P+D+E+ ++  E R +   +K+ YF
Sbjct  345  GFANMEGL--FRVGMGYEYFMSQLNDKFSENLPHDLEF-KDEEEKRKVADEVKKFYF  398


> ta_transcript84818_1
Length=733

 Score =   170 bits (430),  Expect = 3e-45, Method: Compositional matrix adjust.
 Identities = 109/297 (37%), Positives = 163/297 (55%), Gaps = 21/297 (7%)
 Frame = +1

Query  7    EDCLRINVYVPVKKTNTSHLPVFAFIHGGAYRILSKDSDVIYDPYFFMKKGIIVVTMNYR  186
            EDCL  NVYVP   T+  +LPV  F+HGGAY  L+   +V+   +      +I VT NYR
Sbjct  114  EDCLIANVYVP--DTDKKNLPVVVFVHGGAY--LAGFGEVLTPKHMVRTNNVIYVTFNYR  169

Query  187  LGALGFLSLKTPGASGNNGLKDL-----WVKENIGKFGGDSTKITVGGNSSGSSMANYLM  351
            LG  GFL L TP A GN G+KD+     WVK+NI  FGG+   +T+ G S+GSS  + L+
```

```
Sbjct  170  LGVQGFLCLGTPDAPGNAGMKDMVALLRWVKKNIASFGGNPDDVTIDGYSAGSSAVDLLL  229

Query  352  LSRKTTGLIHRVIMFSGSALNFRFLQRYPIENARELA-SGLGLSINDSDVLLQNLKTVNP  528
            LS+ T GL H+ I  SG+ +    +Q  PI NA+E A S  G  I D    L++     P
Sbjct  230  LSKATKGLFHKAIPESGANVAMWSVQVDPIANAKEYAKSEHGFEILDDIYALEDFYKSLP  289

Query  529  FDIV--DAQENMYKKSRNPIITFAPFVPTIEPDSA-DAFLTEDPTSMVKKGLIQNVPVLA  699
            +D +     E M+K   N +     F P +E D+  + FL + P +++KKG    VPVL
Sbjct  290  YDEMYKSGMELMFKNKNNFL-----FSPCVERDTGVEKFLDDTPVNILKKGDYNKVPVLY  344

Query  700  GINSLEGITFYHKIVNNKTLLNDLENNIHLTVPNDIEYPRNSTEFRNLVQSIKELYF  870
            G  ++EG+  +    +  +  ++ L +     +P+D+E+ ++  E R +    +K+ YF
Sbjct  345  GFANMEGL--FRVGMGYEYFMSQLNDKFSENLPHDLEF-KDEEEKRKVADEVKKFYF  398
```

```
> ta_transcript84816_1
Length=947

 Score =   170 bits (430),  Expect = 2e-44, Method: Compositional matrix adjust.
 Identities = 109/297 (37%), Positives = 163/297 (55%), Gaps = 21/297 (7%)
 Frame = +1

Query  7    EDCLRINVYVPVKKTNTSHLPVFAFIHGGAYRILSKDSDVIYDPYFFMKKGIIVVTMNYR  186
            EDCL  NVYVP   T+  +LPV  F+HGGAY  L+   +V+   +      +I VT NYR
Sbjct  114  EDCLIANVYVP--DTDKKNLPVVVFVHGGAY--LAGFGEVLTPKHMVRTNNVIYVTFNYR  169

Query  187  LGALGFLSLKTPGASGNNGLKDL-----WVKENIGKFGGDSTKITVGGNSSGSSMANYLM  351
            LG  GFL L TP A GN G+KD+     WVK+NI  FGG+   +T+ G S+GSS  + L+
Sbjct  170  LGVQGFLCLGTPDAPGNAGMKDMVALLRWVKKNIASFGGNPDDVTIDGYSAGSSAVDLLL  229

Query  352  LSRKTTGLIHRVIMFSGSALNFRFLQRYPIENARELA-SGLGLSINDSDVLLQNLKTVNP  528
            LS+ T GL H+ I  SG+ +    +Q  PI NA+E A S  G  I D    L++     P
Sbjct  230  LSKATKGLFHKAIPESGANVAMWSVQVDPIANAKEYAKSEHGFEILDDIYALEDFYKSLP  289

Query  529  FDIV--DAQENMYKKSRNPIITFAPFVPTIEPDSA-DAFLTEDPTSMVKKGLIQNVPVLA  699
            +D +     E M+K   N +     F P +E D+  + FL + P +++KKG    VPVL
Sbjct  290  YDEMYKSGMELMFKNKNNFL-----FSPCVERDTGVEKFLDDTPVNILKKGDYNKVPVLY  344

Query  700  GINSLEGITFYHKIVNNKTLLNDLENNIHLTVPNDIEYPRNSTEFRNLVQSIKELYF  870
            G  ++EG+  +    +  +  ++ L +     +P+D+E+ ++  E R +    +K+ YF
Sbjct  345  GFANMEGL--FRVGMGYEYFMSQLNDKFSENLPHDLEF-KDEEEKRKVADEVKKFYF  398
```

```
> ta_transcript29751_1
Length=506

 Score =   150 bits (379),  Expect = 4e-39, Method: Compositional matrix adjust.
 Identities = 128/424 (30%), Positives = 202/424 (48%), Gaps = 37/424 (9%)
 Frame = +1

Query  7     EDCLRINVYVPVKKTNTSHLPVFAFIHGGAYRILSKDSDVIYDPYFFMKKGIIVVTMNYR  186
             EDCL  NV+VP   T+  +L V   +HGG Y  LS   +          K +I VT NYR
Sbjct  104   EDCLIANVFVP--DTSEKNLSVVVLVHGGGY--LSGFGNRQTPKDMVRDKKLIYVTFNYR  159

Query  187   LGALGFLSLKTPGASGNNGLKDL-----WVKENIGKFGGDSTKITVGGNSSGSSMANYLM  351
             LG  GFL L T    GN G+KD+     WVK+NI  FGG+   +T+ G+SSGSS  + L+
Sbjct  160   LGVHGFLCLGTADVPGNAGMKDMVALLRWVKKNIASFGGNPDDVTIDGHSSGSSSVDLLL  219

Query  352   LSRKTTGLIHRVIMFSGSALNFRFLQRYPIENARELASGL-GLSINDSDVLLQNLKTVNP  528
             LS+ T GL H+ I  SGS +    +Q  PI +A+E A          D    L++     P
Sbjct  220   LSKATEGLFHKAIPESGSNVAMWSVQIDPIAHAKEFARDEHSFESGDDIYALEDFYKTLP  279

Query  529   FDIVDAQENMYKKSR----NPIITFAPFVPTIEPDSA-DAFLTEDPTSMVKKGLIQNVPV  693
             FD+      MYK       N  +    F P +E D+  + FL + P +++K G    VP+
Sbjct  280   FDV------MYKSGMSLRYNSSVKNILFQPCVERDTGVEKFLDDFPVNILKSGDYNKVPM  333

Query  694   LAGINSLEGITFYHKIVNNKTLLNDLENNIHLTVPNDIEYPRNSTEFRNLVQSIKELYFN  873
             L G  ++EG+      VN ++ ++D+       +PND+++ R+  E    + + +K+ YF
Sbjct  334   LYGFTNMEGLMRVSDFVNYRSFMSDINEKFSDYLPNDLQF-RDKEEKEKVARDVKQFYFG  392

Query  874   ST--DTSALNKYFRIFSDIAFTHKVDYFIYLHKRNPQSNKVFYYEFD--FDGDLNWGKLY  1041
                 +   + +Y   FSD  F +     + L +    +N+V+ Y+F   F+     G +
Sbjct  393   DKVINNDTIFEYIDYFSDNMFAYPTLRSLKL-QVEAGNNQVYLYQFSYPFENQAPEGVM-  450

Query  1042  HDIQYPGTCHSDELGYLFVTNATKENLKTLDNKSRHVLNIMVSVMSNFIKFGNPTPSCDM  1221
               ++  G+ H+ +   +  T   + +L  + N +R          +NFI  G P P+
Sbjct  451   --LKLEGSSHTAQTHVVLDTLNEENDLAIIRNITRQ-------FWANFITTGTPVPAGSE  501
```

```
Query  1222  GICW  1233
                W
Sbjct  502   LPAW  505


> ta_transcript48393_1
Length=666

 Score =   150 bits (378),  Expect = 1e-38, Method: Compositional matrix adjust.
 Identities = 118/394 (30%), Positives = 189/394 (48%), Gaps = 33/394 (8%)
 Frame = +1

Query  1     MGEDCLRINVYVPVKKTNTSHLPVFAFIHGGAYRILSKDSDVIYDPYFFMKKGIIVVTMN  180
             M  DCL  NVYVP   T  + LPV  ++HGGA+++     +++        K II VT N
Sbjct  166   MQVDCLIANVYVP--DTKETKLPVIVYVHGGAFQV--GFGNLMTPKNLVRSKNIIAVTFN  221

Query  181   YRLGALGFLSLKTPGASGNNGLKDL-----WVKENIGKFGGDSTKITVGGNSSGSSMANY  345
             YRLGA GFL L T  A GN GLKD      WV +NI K GG+   +T+ G S+G+   +
Sbjct  222   YRLGAHGFLCLGTEDAPGNAGLKDQVAALRWVNKNIIKLGGNPDDVTIAGYSAGAISVDL  281

Query  346   LMLSRKTTGLIHRVIMFSGSALNFRFLQRYPIENARELASGLGLSINDSDVLLQNLKTVN  525
             + +S+   GL  ++I+ SGS+L    +   P+ENA+  A+ LG +  D    L+
Sbjct  282   MAISKSAEGLFSKIIIESGSSLFAGSIVTDPLENAKIFAARLGFNNIDDVYALEKFYKAA  341

Query  526   PFDIVDAQENMYKKSRNPIITFAPFVPTIEPDSADAFLTEDPTSMVKKGLIQNVPVLAGI  705
              +D +   E M +   N  + F+P V     D   AFL+E P +++K+G    VP++ G
Sbjct  342   SYDSLTKGEFMDRP--NSTLLFSPCVE--RDDDGLAFLSEPPINILKEGSYTQVPMIYGF  397

Query  706   NSLEGI---TFYH--KIVNNKTLLNDLENNIHLTVPNDIEYPRNSTEFRNLVQSIKELYF  870
             +++EG+    F+   K+  N+     +  ++H    +D E          + + IK+ YF
Sbjct  398   SNMEGLFREPFFEFWKLKMNEQFSEFIPGDLHFESKHDRE---------RIAKRIKKFYF  448

Query  871   NSTDTSALN--KYFRIFSDIAFTHKVDYFIYLHKRNPQSNKVFYYEFDFDGDLNWGKLYH  1044
               +  +  N  KY   F D+   +     + +H +N   +K++ YE+ +  D N   + H
Sbjct  449   WDSPINGENILKYIDYFGDVLVKYPALRTVKMHIQNGH-DKLYLYEYSYV-DENVPAVIH  506

Query  1045  DIQYPGTCHSDELGYLFVTNATKENLKTLDNKSR  1146
                  G  H  +   + V +     LK     SR
Sbjct  507   TTNTRGANHCAQT--VAVLDGQNMTLKKRRQPSR  538


> ta_transcript15565_1
Length=424

 Score =   124 bits (310),  Expect = 2e-30, Method: Compositional matrix adjust.
 Identities = 107/397 (27%), Positives = 177/397 (45%), Gaps = 51/397 (13%)
 Frame = +1

Query  241   GLKDL-----WVKENIGKFGGDSTKITVGGNSSGSSMANYLMLSRKTTGLIHRVIMFSGS  405
             G+KD+     WV  NI  FGG+   +T+ G S+GS     LMLS+   GL H+ I  SGS
Sbjct  1     GMKDMVALLRWVNRNIAAFGGNPNDVTLEGWSAGSVSVELLMLSKAAKGLFHKAIPESGS  60

Query  406   ALNFRFLQRYPIENARELA-SGLGLSINDSDVLLQNLKTVNPFDIVDAQENMYKKSRNPI  582
             +     +   PI NA++ A S LG    D+   L+      PF      ++MY++   P+
Sbjct  61    SFAPFSVGVDPIGNAKDFARSDLGFKDVDNISALEEFYKTVPF------KDMYQQG--PL  112

Query  583   ITFA---PFVPTIEPD-SADAFLTEDPTSMVKKGLIQNVPVLAGINSLEGITFYHKIVNN  750
             I F     F P +E +   D FL + P +++KKG    VP+L G ++ EG+  Y
Sbjct  113   IIFRHNFTFTPCVERNIGQDVFLDDTPYNILKKGDYMKVPMLYGFSNKEGL--YRVDYGY  170

Query  751   KTLLNDLENNIHLTVPNDIEYPRNSTEFRNLVQSIKELYFNST--DTSALNKYFRIFSDI  924
             +  +  +       +PND+++ ++  E   +   IKE YF +   D   + +Y   F+D
Sbjct  171   QFFIEGMNKKFSDYLPNDLQF-KDENEKEQVATMIKEFYFGNKKIDQDTVLEYIDYFTDS  229

Query  925   AFTHKVDYFIYLHKRNPQSNKVFYYEFDFDGDLNWGKLYHDIQYPGTCHSDELGYLFVTN  1104
              F       + LH     +N+++ Y+F +           ++ + P +     +G    +N
Sbjct  230   MFAGPSLKEVQLHVAA-GNNQIYLYQFSYP---------YENKVPESIKVKVIG----SN  275

Query  1105  ATKENLKTLDN-------------KSRHVLNIMVSVMSNFIKFGNPTPSCDMGICWTESN  1245
                E +  LDN                 V N+   +  NFIK G P P       W  ++
Sbjct  276   HCGETMTVLDNWLGGAEMPAERNSDLEQVRNVTRQIWGNFIKTGKPVPQGSNLPTWPATD  335

Query  1246  CSRN-HIVLKEIPVLKNNSPYPERFTFWSEVYKQFYK  1353
              S + ++V+   P LK     P    FW+E+Y++ Y+
Sbjct  336   GSGSPYMVITNKPELKGQPFQPMSVMFWTEIYQRHYR  372
```

```
************************************************************************************************
***********************************************************************
Query= gi|112983019:25-576 Bombyx mori juvenile hormone diol kinase (Jhdk), mRNA

Length=552
                                                                      Score     E
Sequences producing significant alignments:                          (Bits)  Value

  ta_transcript24996_1                                                  206   2e-64


> ta_transcript24996_1
Length=354

 Score =   206 bits (525),  Expect = 2e-64, Method: Compositional matrix adjust.
 Identities = 93/181 (51%), Positives = 134/181 (74%), Gaps = 0/181 (0%)
 Frame = +1

Query  1    MVSEVRKKKLLHVFTVFFDSDKSGVVEKQDFELAAQNIAKLRGWAPGSPAYDILQESMIA  180
            MVS+ RKKK LHVF  FFD D +G V+KQDF +A  N+A+LRG+ PG   Y++++E + +
Sbjct  13   MVSDFRKKKYLHVFNSFFDVDGNGTVDKQDFLIANHNLAELRGYKPGDVTYELMKELIHS  72

Query  181  IWLGLQKQADADGDGKVTQDEWLALWDEYAKDPAAAKDWQNLLCKSIFQIQDSSNDGSVD  360
            IW GL  QAD DG G +  DEW+ LW++++K+P  A +WQ L CK+IFQ+ D++ DGS+D
Sbjct  73   IWDGLIAQADKDGSGNICVDEWVQLWEDFSKNPDNAAEWQQLYCKAIFQMLDATGDGSID  132

Query  361  VNEYVTVHESFGLNKEESTEAFKKLAKGKDSISWADFQELWKEYFSSDDPDVPGNYIFGR  540
             +EY TV +SFG ++ +  +AF+KL+ GK SIS A+F+ LW E+++S++P  PGNYIFG
Sbjct  133  ADEYATVFKSFGRDEAKCKQAFEKLSGGKPSISQAEFENLWSEFYTSENPSDPGNYIFGL  192

Query  541  L  543
            L
Sbjct  193  L  193

************************************************************************************************
*********************************************************************

Query= gi|114052405:74-805 Bombyx mori juvenile hormone esterase binding protein (LOC733014),
mRNA

Length=732
                                                                      Score     E
Sequences producing significant alignments:                          (Bits)  Value

  ta_transcript22729_1                                                84.0    5e-18


> ta_transcript22729_1
Length=374

 Score = 84.0 bits (206),  Expect = 5e-18, Method: Compositional matrix adjust.
 Identities = 80/185 (43%), Positives = 102/185 (55%), Gaps = 0/185 (0%)
 Frame = -3

Query  700  ND*QC*PTNClqlll*tl**ICSSK*ICYPGTGVELLKLLEACHRFPSYFSALLTHDNTL  521
            + * C PT+CL  L *TL *ICSS+*  +PG  V LL       +F ++      +  +T
Sbjct  47   SG*LCSPTSCLLHLW*TLG*ICSSR*TVFPGILVALLVKQAVSLQFLAFV*GPSSRGST*  106

Query  520  *FQPNAYPNHQKLLQLDKEMHKIYLLFLWLTIAAGTQKVETQQSSVH**HLYPPNPLEF*  341
            *  P+A    + LL  D+  H+   L+      A T  V T+ +SV   H    + L  *
Sbjct  107  *SPPSACLRRRTLLPPDRGKHRRCHLW*QPASVAVTY*VWTRPASVRQWHPSLQDLLRR*  166

Query  340  NHCFVVLLKQSELHVWKLQ*TRSD*ILDQNTVL**NLKTSNSLRIL*MKATLSAGIPSIE  161
            +H      LKQ ELHV KLQ*TR D*I  +NTV+ *+L+TSNSLR L*  AT  AG PS E
Sbjct  167  SHSAGGHLKQPELHV*KLQ*TRED*IRYRNTVVL*SLRTSNSLRSL*TMAT**AGTPSAE  226

Query  160  ATSVF  146
            A S F
Sbjct  227  AISAF  231


************************************************************************************************
**********************************************************************

Query= gi|112983363:40-1401 Bombyx mori neverland (Nvd), mRNA
```

```
Length=1362
                                                                      Score     E
Sequences producing significant alignments:                          (Bits)  Value

  ta_transcript14785_1                                                  152   4e-42



> ta_transcript14785_1
Length=218

 Score =   152 bits (385),  Expect = 4e-42, Method: Compositional matrix adjust.
 Identities = 100/185 (54%), Positives = 110/185 (59%), Gaps = 0/185 (0%)
 Frame = -2

Query  1337  ASLKPRLCSL*N*PNQFLKVRIVLSVLT*AGALTKRLLFHIVTSLSNMYASHLTRNAPTG  1158
              ASLKP LCSL*N  NQ L  RIVLS  T AGA T RLLFH+ TS SN YAS L    P G
Sbjct  34    ASLKPTLCSL*NCANQLLNARIVLSARTYAGAETYRLLFHMATSRSN*YASILFMITPKG  93

Query  1157  ALYAGEYTRWMTFCSRGPRGVTDCTM*KGPTLVFRNKRTCPGPICVTLTSMRAKSYLRTL  978
              AL  GE   W T C  G  GVTD     GP+ VFR +RTCPGPICVT TS        +L
Sbjct  94    AL*NGEKILWTTLCCNGAMGVTD*DTRIGPSGVFRWRRTCPGPICVTCTSTWKTLSFISL  153

Query  977   YSCVMCISDV*SSDFVQSAFHTCRPMISCSTGYLSPRslsseee*TAFR*GTSAPFSGIS  798
               SCV  +  V SS  V SA    +PM     GY S   ++S+   TAF+* TSAPFSGIS
Sbjct  154   *SCVSLMEAVWSSPLVHSAVQ*WQPMKFSRRGYFSASLVTSDVAWTAFK*ATSAPFSGIS  213

Query  797   CM*AL  783
               M* L
Sbjct  214   *M*QL  218

************************************************************************************************
***********************************************************************
```

Query= gi|162462655:125-1735 Bombyx mori cytochrome P450 (Cyp307a1), mRNA

```
Length=1611
                                                                      Score     E
Sequences producing significant alignments:                          (Bits)  Value

  ta_transcript75638_1                                                  152   4e-38
  ta_transcript75639_1                                                  151   5e-38
  ta_transcript75641_1                                                  151   6e-38
  ta_transcript75637_1                                                  151   6e-38
  ta_transcript75640_1                                                  151   7e-38
  ta_transcript75642_1                                                  150   8e-38
  ta_transcript75635_1                                                  149   4e-37
  ta_transcript75636_1                                                  149   4e-37
  ta_transcript75634_1                                                  149   4e-37
  ta_transcript75632_1                                                  149   4e-37
  ta_transcript75629_1                                                  149   4e-37
  ta_transcript75630_1                                                  149   4e-37
  ta_transcript75633_1                                                  149   4e-37
  ta_transcript75631_1                                                  149   5e-37

> ta_transcript75638_1
Length=944

 Score =   152 bits (383),  Expect = 4e-38, Method: Compositional matrix adjust.
 Identities = 176/477 (37%), Positives = 261/477 (55%), Gaps = 14/477 (3%)
 Frame = +3

Query  153   YRESTPSGRPRVALPGLH*AIEEIRRHfqrqigqrrlrgr*qfiSDQGSFKSKRQCgggt  332
              ++ S+P  + RVA+PG+H A   +  H QR      + G  QF +DQG  + +R+    T
Sbjct  79    WKSSSPR-KARVAVPGVHGAG*GVWGHLQRDSWLHEMPGGQQFGADQGGAEPEREILRWT  137

Query  333   tglpalp*alrrrpE*LSGPVRLVQSSAAQEEPGEAALRSQTAhrllrqnrhrrhl*ICR  512
                LP +P  LRRR E* +  + LVQ   AQEEPG + LR Q AHR LR++R RRHL   R
Sbjct  138   ARLPEVPQTLRRRQE*FTSAM*LVQPPGAQEEPGASPLRPQAAHRQLREDRQRRHLRSYR  197

Query  513   ANSNPqridqqi*rqHRSEADTDEVRHEHVLKLHV*rpf**rrpripEDCR-SLRRDILG  689
              A+ +PQ  D  +  Q + +A   + R+EHV  LHV R       R  +  R   RR++LG
Sbjct  198   AHPDPQAGDPSVRSQRQPQALPHDHRYEHV*PLHVRRALRS*HRRGVQKSRRPFRRNLLG  257

Query  690   DQPGLRCRFSAVAGAVLQKAHGEALQLVPGHPILHLIEDRGATRNKLRHGSSGEGLSGRP  869
```

```
              DQP +RC   AVA  VLQ+ HG+AL+LVPGHP LH  EDR    ++  +    +GL G
Sbjct  258   DQPRIRC*LPAVARTVLQETHGQALRLVPGHPFLHSFEDRRKKGDESGN*RPRKGLLGWL  317

Query  870   PQGATRRPDHG*EHYYLYARRLPRGAFVCW*LSHAVSDRSRQRPGGRQEDTSGNRRGDQR  1049
                 A+RR D   EH +L+A   P  +     L HA+ D   QR G  +++    +R  QR
Sbjct  318   APSASRRSDS*QEHNHLHA*GFPWRSLFRRQLGHALFDSCSQRSGSSEKN---QKRD*QR  374

Query  1050  ---QKTRRSHRPQPFAVHRGHHLGMSPVRVLAHRAARGHRERQYIRVRNRKGYCRFYQQL  1220
                 + +R S R +  A+HR +   M  +R  +HR    +R+ +  R+   K Y R ++QL
Sbjct  375   H*GKTSRISPRQKQSALHRSYCSRMLAIRFFSHRPTCRNRKCRCRRLWRGKRYYRVHKQL  434

Query  1221  RSEQFRTVRVRAGEIRSEPFPGEDQGQDETQLPV*LRPGVRLGESARRQA*RREGD----  1388
              R E FR +  +  EIR +   G  Q +++ Q+ V  R G+R  ++  R  *+ E +
Sbjct  435   RVEHFREILGKP*EIRPDKILGAHQAKNQAQITVRFRHGIR*RKN--RPY*QDERN*KRC  492

Query  1389  AFREKEHTPLHTLQHW*ENVYRSNYGHVDVFHDVREHHAVL*GRRREHQRPQTEAGV  1559
                R++++  L T+QHW EN++RS++G   V  DV  HH+ +*    + +RPQTE+ +
Sbjct  493   LLRQEKYPTLLTVQHWQENLHRSDHGDHYVVCDVC*HHSRV*CGSSQPRRPQTESSL  549


> ta_transcript75639_1
Length=940

 Score =   151 bits (382),  Expect = 5e-38, Method: Compositional matrix adjust.
 Identities = 176/477 (37%), Positives = 261/477 (55%), Gaps = 14/477 (3%)
 Frame = +3

Query  153   YRESTPSGRPRVALPGLH*AIEEIRRHfqrqigqrrlrgr*qfiSDQGSFKSKRQCgggt  332
              ++ S+P  + RVA+PG+H A   +  H QR      + G  QF +DQG  + +R+    T
Sbjct  79    WKSSSPR-KARVAVPGVHGAG*GVWGHLQRDSWLHEMPGGQQFGADQGGAEPEREILRWT  137

Query  333   tglpalp*alrrrpE*LSGPVRLVQSSAAQEEPGEAALRSQTAhrllrqnrhrrhl*ICR  512
                LP +P  LRRR E* +  + LVQ   AQEEPG + LR Q AHR LR++R RRHL   R
Sbjct  138   ARLPEVPQTLRRRQE*FTSAM*LVQPPGAQEEPGASPLRPQAAHRQLREDRQRRHLRSYR  197

Query  513   ANSNPqridqqi*rqHRSEADTDEVRHEHVLKLHV*rpf**rrpripEDCR-SLRRDILG  689
              A+ +PQ  D  +  Q + +A   + R+EHV  LHV R       R  +  R   RR++LG
Sbjct  198   AHPDPQAGDPSVRSQRQPQALPHDHRYEHV*PLHVRRALRS*HRRGVQKSRRPFRRNLLG  257

Query  690   DQPGLRCRFSAVAGAVLQKAHGEALQLVPGHPILHLIEDRGATRNKLRHGSSGEGLSGRP  869
              DQP +RC   AVA  VLQ+ HG+AL+LVPGHP LH  EDR    ++  +    +GL G
Sbjct  258   DQPRIRC*LPAVARTVLQETHGQALRLVPGHPFLHSFEDRRKKGDESGN*RPRKGLLGWL  317

Query  870   PQGATRRPDHG*EHYYLYARRLPRGAFVCW*LSHAVSDRSRQRPGGRQEDTSGNRRGDQR  1049
                 A+RR D   EH +L+A   P  +     L HA+ D   QR G  +++    +R  QR
Sbjct  318   APSASRRSDS*QEHNHLHA*GFPWRSLFRRQLGHALFDSCSQRSGSSEKN---QKRD*QR  374

Query  1050  ---QKTRRSHRPQPFAVHRGHHLGMSPVRVLAHRAARGHRERQYIRVRNRKGYCRFYQQL  1220
                 + +R S R +  A+HR +   M  +R  +HR    +R+ +  R+   K Y R ++QL
Sbjct  375   H*GKTSRISPRQKQSALHRSYCSRMLAIRFFSHRPTCRNRKCRCRRLWRGKRYYRVHKQL  434

Query  1221  RSEQFRTVRVRAGEIRSEPFPGEDQGQDETQLPV*LRPGVRLGESARRQA*RREGD----  1388
              R E FR +  +  EIR +   G  Q +++ Q+ V  R G+R  ++  R  *+ E +
Sbjct  435   RVEHFREILGKP*EIRPDKILGAHQAKNQAQITVRFRHGIR*RKN--RPY*QDERN*KRC  492

Query  1389  AFREKEHTPLHTLQHW*ENVYRSNYGHVDVFHDVREHHAVL*GRRREHQRPQTEAGV  1559
                R++++  L T+QHW EN++RS++G   V  DV  HH+ +*    + +RPQTE+ +
Sbjct  493   LLRQEKYPTLLTVQHWQENLHRSDHGDHYVVCDVC*HHSRV*CGSSQPRRPQTESSL  549


> ta_transcript75641_1
Length=890

 Score =   151 bits (381),  Expect = 6e-38, Method: Compositional matrix adjust.
 Identities = 176/477 (37%), Positives = 261/477 (55%), Gaps = 14/477 (3%)
 Frame = +3

Query  153   YRESTPSGRPRVALPGLH*AIEEIRRHfqrqigqrrlrgr*qfiSDQGSFKSKRQCgggt  332
              ++ S+P  + RVA+PG+H A   +  H QR      + G  QF +DQG  + +R+    T
Sbjct  79    WKSSSPR-KARVAVPGVHGAG*GVWGHLQRDSWLHEMPGGQQFGADQGGAEPEREILRWT  137

Query  333   tglpalp*alrrrpE*LSGPVRLVQSSAAQEEPGEAALRSQTAhrllrqnrhrrhl*ICR  512
                LP +P  LRRR E* +  + LVQ   AQEEPG + LR Q AHR LR++R RRHL   R
Sbjct  138   ARLPEVPQTLRRRQE*FTSAM*LVQPPGAQEEPGASPLRPQAAHRQLREDRQRRHLRSYR  197

Query  513   ANSNPqridqqi*rqHRSEADTDEVRHEHVLKLHV*rpf**rrpripEDCR-SLRRDILG  689
              A+ +PQ  D  +  Q + +A   + R+EHV  LHV R       R  +  R   RR++LG
```

```
Sbjct  198   AHPDPQAGDPSVRSQRQPQALPHDHRYEHV*PLHVRRALRS*HRRGVQKSRRPFRRNLLG  257

Query  690   DQPGLRCRFSAVAGAVLQKAHGEALQLVPGHPILHLIEDRGATRNKLRHGSSGEGLSGRP  869
             DQP +RC   AVA  VLQ+ HG+AL+LVPGHP LH  EDR    ++  +    +GL G
Sbjct  258   DQPRIRC*LPAVARTVLQETHGQALRLVPGHPFLHSFEDRRKKGDESGN*RPRKGLLGWL  317

Query  870   PQGATRRPDHG*EHYYLYARRLPRGAFVCW*LSHAVSDRSRQRPGGRQEDTSGNRRGDQR  1049
                A+RR D   EH +L+A   P  +     L HA+ D   QR G  +++    +R  QR
Sbjct  318   APSASRRSDS*QEHNHLHA*GFPWRSLFRRQLGHALFDSCSQRSGSSEKN---QKRD*QR  374

Query  1050  ---QKTRRSHRPQPFAVHRGHHLGMSPVRVLAHRAARGHRERQYIRVRNRKGYCRFYQQL  1220
                + +R S R +  A+HR +   M  +R  +HR    +R+ +  R+   K Y R ++QL
Sbjct  375   H*GKTSRISPRQKQSALHRSYCSRMLAIRFFSHRPTCRNRKCRCRRLWRGKRYYRVHKQL  434

Query  1221  RSEQFRTVRVRAGEIRSEPFPGEDQGQDETQLPV*LRPGVRLGESARRQA*RREGD----  1388
             R E FR +  +  EIR +   G  Q +++ Q+ V  R G+R  ++  R  *+ E +
Sbjct  435   RVEHFREILGKP*EIRPDKILGAHQAKNQAQITVRFRHGIR*RKN--RPY*QDERN*KRC  492

Query  1389  AFREKEHTPLHTLQHW*ENVYRSNYGHVDVFHDVREHHAVL*GRRREHQRPQTEAGV  1559
               R++++  L T+QHW EN++RS++G   V  DV  HH+ +*    + +RPQTE+ +
Sbjct  493   LLRQEKYPTLLTVQHWQENLHRSDHGDHYVVCDVC*HHSRV*CGSSQPRRPQTESSL  549


> ta_transcript75637_1
Length=947

 Score =   151 bits (381),  Expect = 6e-38, Method: Compositional matrix adjust.
 Identities = 176/477 (37%), Positives = 261/477 (55%), Gaps = 14/477 (3%)
 Frame = +3

Query  153   YRESTPSGRPRVALPGLH*AIEEIRRHfqrqigqrrlrgr*qfiSDQGSFKSKRQCgggt  332
             ++ S+P  + RVA+PG+H A   +  H QR      + G  QF +DQG  + +R+    T
Sbjct  79    WKSSSPR-KARVAVPGVHGAG*GVWGHLQRDSWLHEMPGGQQFGADQGGAEPEREILRWT  137

Query  333   tglpalp*alrrrpE*LSGPVRLVQSSAAQEEPGEAALRSQTAhrllrqnrhrrhl*ICR  512
               LP +P  LRRR E* +  + LVQ   AQEEPG + LR Q AHR LR++R RRHL   R
Sbjct  138   ARLPEVPQTLRRRQE*FTSAM*LVQPPGAQEEPGASPLRPQAAHRQLREDRQRRHLRSYR  197

Query  513   ANSNPqridqqi*rqHRSEADTDEVRHEHVLKLHV*rpf**rrpripEDCR-SLRRDILG  689
             A+ +PQ  D  +  Q + +A   + R+EHV  LHV R       R  +  R   RR++LG
Sbjct  198   AHPDPQAGDPSVRSQRQPQALPHDHRYEHV*PLHVRRALRS*HRRGVQKSRRPFRRNLLG  257

Query  690   DQPGLRCRFSAVAGAVLQKAHGEALQLVPGHPILHLIEDRGATRNKLRHGSSGEGLSGRP  869
             DQP +RC   AVA  VLQ+ HG+AL+LVPGHP LH  EDR    ++  +    +GL G
Sbjct  258   DQPRIRC*LPAVARTVLQETHGQALRLVPGHPFLHSFEDRRKKGDESGN*RPRKGLLGWL  317

Query  870   PQGATRRPDHG*EHYYLYARRLPRGAFVCW*LSHAVSDRSRQRPGGRQEDTSGNRRGDQR  1049
                A+RR D   EH +L+A   P  +     L HA+ D   QR G  +++    +R  QR
Sbjct  318   APSASRRSDS*QEHNHLHA*GFPWRSLFRRQLGHALFDSCSQRSGSSEKN---QKRD*QR  374

Query  1050  ---QKTRRSHRPQPFAVHRGHHLGMSPVRVLAHRAARGHRERQYIRVRNRKGYCRFYQQL  1220
                + +R S R +  A+HR +   M  +R  +HR    +R+ +  R+   K Y R ++QL
Sbjct  375   H*GKTSRISPRQKQSALHRSYCSRMLAIRFFSHRPTCRNRKCRCRRLWRGKRYYRVHKQL  434

Query  1221  RSEQFRTVRVRAGEIRSEPFPGEDQGQDETQLPV*LRPGVRLGESARRQA*RREGD----  1388
             R E FR +  +  EIR +   G  Q +++ Q+ V  R G+R  ++  R  *+ E +
Sbjct  435   RVEHFREILGKP*EIRPDKILGAHQAKNQAQITVRFRHGIR*RKN--RPY*QDERN*KRC  492

Query  1389  AFREKEHTPLHTLQHW*ENVYRSNYGHVDVFHDVREHHAVL*GRRREHQRPQTEAGV  1559
               R++++  L T+QHW EN++RS++G   V  DV  HH+ +*    + +RPQTE+ +
Sbjct  493   LLRQEKYPTLLTVQHWQENLHRSDHGDHYVVCDVC*HHSRV*CGSSQPRRPQTESSL  549


> ta_transcript75640_1
Length=937

 Score =   151 bits (381),  Expect = 7e-38, Method: Compositional matrix adjust.
 Identities = 176/477 (37%), Positives = 261/477 (55%), Gaps = 14/477 (3%)
 Frame = +3

Query  153   YRESTPSGRPRVALPGLH*AIEEIRRHfqrqigqrrlrgr*qfiSDQGSFKSKRQCgggt  332
             ++ S+P  + RVA+PG+H A   +  H QR      + G  QF +DQG  + +R+    T
Sbjct  79    WKSSSPR-KARVAVPGVHGAG*GVWGHLQRDSWLHEMPGGQQFGADQGGAEPEREILRWT  137

Query  333   tglpalp*alrrrpE*LSGPVRLVQSSAAQEEPGEAALRSQTAhrllrqnrhrrhl*ICR  512
               LP +P  LRRR E* +  + LVQ   AQEEPG + LR Q AHR LR++R RRHL   R
Sbjct  138   ARLPEVPQTLRRRQE*FTSAM*LVQPPGAQEEPGASPLRPQAAHRQLREDRQRRHLRSYR  197
```

```
Query  513   ANSNPqridqqi*rqHRSEADTDEVRHEHVLKLHV*rpf**rrpripEDCR-SLRRDILG  689
             A+ +PQ  D  +  Q + +A   + R+EHV  LHV R       R  +  R   RR++LG
Sbjct  198   AHPDPQAGDPSVRSQRQPQALPHDHRYEHV*PLHVRRALRS*HRRGVQKSRRPFRRNLLG  257

Query  690   DQPGLRCRFSAVAGAVLQKAHGEALQLVPGHPILHLIEDRGATRNKLRHGSSGEGLSGRP  869
             DQP +RC   AVA  VLQ+ HG+AL+LVPGHP LH  EDR    ++  +    +GL G
Sbjct  258   DQPRIRC*LPAVARTVLQETHGQALRLVPGHPFLHSFEDRRKKGDESGN*RPRKGLLGWL  317

Query  870   PQGATRRPDHG*EHYYLYARRLPRGAFVCW*LSHAVSDRSRQRPGGRQEDTSGNRRGDQR  1049
                A+RR D   EH +L+A   P  +     L HA+ D   QR G  +++    +R  QR
Sbjct  318   APSASRRSDS*QEHNHLHA*GFPWRSLFRRQLGHALFDSCSQRSGSSEKN---QKRD*QR  374

Query  1050  ---QKTRRSHRPQPFAVHRGHHLGMSPVRVLAHRAARGHRERQYIRVRNRKGYCRFYQQL  1220
                + +R S R +  A+HR +   M  +R  +HR    +R+ +  R+   K Y R ++QL
Sbjct  375   H*GKTSRISPRQKQSALHRSYCSRMLAIRFFSHRPTCRNRKCRCRRLWRGKRYYRVHKQL  434

Query  1221  RSEQFRTVRVRAGEIRSEPFPGEDQGQDETQLPV*LRPGVRLGESARRQA*RREGD----  1388
             R E FR +  +  EIR +   G  Q +++ Q+ V  R G+R  ++  R  *+ E +
Sbjct  435   RVEHFREILGKP*EIRPDKILGAHQAKNQAQITVRFRHGIR*RKN--RPY*QDERN*KRC  492

Query  1389  AFREKEHTPLHTLQHW*ENVYRSNYGHVDVFHDVREHHAVL*GRRREHQRPQTEAGV  1559
               R++++  L T+QHW EN++RS++G   V  DV  HH+ +*    + +RPQTE+ +
Sbjct  493   LLRQEKYPTLLTVQHWQENLHRSDHGDHYVVCDVC*HHSRV*CGSSQPRRPQTESSL  549


> ta_transcript75642_1
Length=887

 Score =   150 bits (380),  Expect = 8e-38, Method: Compositional matrix adjust.
 Identities = 176/477 (37%), Positives = 261/477 (55%), Gaps = 14/477 (3%)
 Frame = +3

Query  153   YRESTPSGRPRVALPGLH*AIEEIRRHfqrqigqrrlrgr*qfiSDQGSFKSKRQCgggt  332
             ++ S+P  + RVA+PG+H A   +  H QR      + G  QF +DQG  + +R+    T
Sbjct  79    WKSSSPR-KARVAVPGVHGAG*GVWGHLQRDSWLHEMPGGQQFGADQGGAEPEREILRWT  137

Query  333   tglpalp*alrrrpE*LSGPVRLVQSSAAQEEPGEAALRSQTAhrllrqnrhrrhl*ICR  512
               LP +P  LRRR E* +  + LVQ   AQEEPG + LR Q AHR LR++R RRHL   R
Sbjct  138   ARLPEVPQTLRRRQE*FTSAM*LVQPPGAQEEPGASPLRPQAAHRQLREDRQRRHLRSYR  197

Query  513   ANSNPqridqqi*rqHRSEADTDEVRHEHVLKLHV*rpf**rrpripEDCR-SLRRDILG  689
             A+ +PQ  D  +  Q + +A   + R+EHV  LHV R       R  +  R   RR++LG
Sbjct  198   AHPDPQAGDPSVRSQRQPQALPHDHRYEHV*PLHVRRALRS*HRRGVQKSRRPFRRNLLG  257

Query  690   DQPGLRCRFSAVAGAVLQKAHGEALQLVPGHPILHLIEDRGATRNKLRHGSSGEGLSGRP  869
             DQP +RC   AVA  VLQ+ HG+AL+LVPGHP LH  EDR    ++  +    +GL G
Sbjct  258   DQPRIRC*LPAVARTVLQETHGQALRLVPGHPFLHSFEDRRKKGDESGN*RPRKGLLGWL  317

Query  870   PQGATRRPDHG*EHYYLYARRLPRGAFVCW*LSHAVSDRSRQRPGGRQEDTSGNRRGDQR  1049
                A+RR D   EH +L+A   P  +     L HA+ D   QR G  +++    +R  QR
Sbjct  318   APSASRRSDS*QEHNHLHA*GFPWRSLFRRQLGHALFDSCSQRSGSSEKN---QKRD*QR  374

Query  1050  ---QKTRRSHRPQPFAVHRGHHLGMSPVRVLAHRAARGHRERQYIRVRNRKGYCRFYQQL  1220
                + +R S R +  A+HR +   M  +R  +HR    +R+ +  R+   K Y R ++QL
Sbjct  375   H*GKTSRISPRQKQSALHRSYCSRMLAIRFFSHRPTCRNRKCRCRRLWRGKRYYRVHKQL  434

Query  1221  RSEQFRTVRVRAGEIRSEPFPGEDQGQDETQLPV*LRPGVRLGESARRQA*RREGD----  1388
             R E FR +  +  EIR +   G  Q +++ Q+ V  R G+R  ++  R  *+ E +
Sbjct  435   RVEHFREILGKP*EIRPDKILGAHQAKNQAQITVRFRHGIR*RKN--RPY*QDERN*KRC  492

Query  1389  AFREKEHTPLHTLQHW*ENVYRSNYGHVDVFHDVREHHAVL*GRRREHQRPQTEAGV  1559
               R++++  L T+QHW EN++RS++G   V  DV  HH+ +*    + +RPQTE+ +
Sbjct  493   LLRQEKYPTLLTVQHWQENLHRSDHGDHYVVCDVC*HHSRV*CGSSQPRRPQTESSL  549


> ta_transcript75635_1
Length=1027

 Score =   149 bits (376),  Expect = 4e-37, Method: Compositional matrix adjust.
 Identities = 176/477 (37%), Positives = 261/477 (55%), Gaps = 14/477 (3%)
 Frame = +3

Query  153   YRESTPSGRPRVALPGLH*AIEEIRRHfqrqigqrrlrgr*qfiSDQGSFKSKRQCgggt  332
             ++ S+P  + RVA+PG+H A   +  H QR      + G  QF +DQG  + +R+    T
Sbjct  79    WKSSSPR-KARVAVPGVHGAG*GVWGHLQRDSWLHEMPGGQQFGADQGGAEPEREILRWT  137
```

```
Query  333   tglpalp*alrrrpE*LSGPVRLVQSSAAQEEPGEAALRSQTAhrllrqnrhrrhl*ICR  512
               LP +P  LRRR E* +  + LVQ   AQEEPG + LR Q AHR LR++R RRHL   R
Sbjct  138   ARLPEVPQTLRRRQE*FTSAM*LVQPPGAQEEPGASPLRPQAAHRQLREDRQRRHLRSYR  197

Query  513   ANSNPqridqqi*rqHRSEADTDEVRHEHVLKLHV*rpf**rrpripEDCR-SLRRDILG  689
             A+ +PQ  D  +  Q + +A   + R+EHV  LHV R       R  +  R   RR++LG
Sbjct  198   AHPDPQAGDPSVRSQRQPQALPHDHRYEHV*PLHVRRALRS*HRRGVQKSRRPFRRNLLG  257

Query  690   DQPGLRCRFSAVAGAVLQKAHGEALQLVPGHPILHLIEDRGATRNKLRHGSSGEGLSGRP  869
             DQP +RC   AVA  VLQ+ HG+AL+LVPGHP LH  EDR    ++  +    +GL G
Sbjct  258   DQPRIRC*LPAVARTVLQETHGQALRLVPGHPFLHSFEDRRKKGDESGN*RPRKGLLGWL  317

Query  870   PQGATRRPDHG*EHYYLYARRLPRGAFVCW*LSHAVSDRSRQRPGGRQEDTSGNRRGDQR  1049
                A+RR D   EH +L+A   P  +     L HA+ D   QR G  +++    +R  QR
Sbjct  318   APSASRRSDS*QEHNHLHA*GFPWRSLFRRQLGHALFDSCSQRSGSSEKN---QKRD*QR  374

Query  1050  ---QKTRRSHRPQPFAVHRGHHLGMSPVRVLAHRAARGHRERQYIRVRNRKGYCRFYQQL  1220
                + +R S R +  A+HR +   M  +R  +HR    +R+ +  R+   K Y R ++QL
Sbjct  375   H*GKTSRISPRQKQSALHRSYCSRMLAIRFFSHRPTCRNRKCRCRRLWRGKRYYRVHKQL  434

Query  1221  RSEQFRTVRVRAGEIRSEPFPGEDQGQDETQLPV*LRPGVRLGESARRQA*RREGD----  1388
             R E FR +  +  EIR +   G  Q +++ Q+ V  R G+R  ++  R  *+ E +
Sbjct  435   RVEHFREILGKP*EIRPDKILGAHQAKNQAQITVRFRHGIR*RKN--RPY*QDERN*KRC  492

Query  1389  AFREKEHTPLHTLQHW*ENVYRSNYGHVDVFHDVREHHAVL*GRRREHQRPQTEAGV  1559
               R++++  L T+QHW EN++RS++G   V  DV  HH+ +*    + +RPQTE+ +
Sbjct  493   LLRQEKYPTLLTVQHWQENLHRSDHGDHYVVCDVC*HHSRV*CGSSQPRRPQTESSL  549
```

```
> ta_transcript75636_1
Length=1024

 Score =   149 bits (376),  Expect = 4e-37, Method: Compositional matrix adjust.
 Identities = 176/477 (37%), Positives = 261/477 (55%), Gaps = 14/477 (3%)
 Frame = +3

Query  153   YRESTPSGRPRVALPGLH*AIEEIRRHfqrqigqrrlrgr*qfiSDQGSFKSKRQCgggt  332
             ++ S+P  + RVA+PG+H A   +  H QR      + G  QF +DQG  + +R+    T
Sbjct  79    WKSSSPR-KARVAVPGVHGAG*GVWGHLQRDSWLHEMPGGQQFGADQGGAEPEREILRWT  137

Query  333   tglpalp*alrrrpE*LSGPVRLVQSSAAQEEPGEAALRSQTAhrllrqnrhrrhl*ICR  512
               LP +P  LRRR E* +  + LVQ   AQEEPG + LR Q AHR LR++R RRHL   R
Sbjct  138   ARLPEVPQTLRRRQE*FTSAM*LVQPPGAQEEPGASPLRPQAAHRQLREDRQRRHLRSYR  197

Query  513   ANSNPqridqqi*rqHRSEADTDEVRHEHVLKLHV*rpf**rrpripEDCR-SLRRDILG  689
             A+ +PQ  D  +  Q + +A   + R+EHV  LHV R       R  +  R   RR++LG
Sbjct  198   AHPDPQAGDPSVRSQRQPQALPHDHRYEHV*PLHVRRALRS*HRRGVQKSRRPFRRNLLG  257

Query  690   DQPGLRCRFSAVAGAVLQKAHGEALQLVPGHPILHLIEDRGATRNKLRHGSSGEGLSGRP  869
             DQP +RC   AVA  VLQ+ HG+AL+LVPGHP LH  EDR    ++  +    +GL G
Sbjct  258   DQPRIRC*LPAVARTVLQETHGQALRLVPGHPFLHSFEDRRKKGDESGN*RPRKGLLGWL  317

Query  870   PQGATRRPDHG*EHYYLYARRLPRGAFVCW*LSHAVSDRSRQRPGGRQEDTSGNRRGDQR  1049
                A+RR D   EH +L+A   P  +     L HA+ D   QR G  +++    +R  QR
Sbjct  318   APSASRRSDS*QEHNHLHA*GFPWRSLFRRQLGHALFDSCSQRSGSSEKN---QKRD*QR  374

Query  1050  ---QKTRRSHRPQPFAVHRGHHLGMSPVRVLAHRAARGHRERQYIRVRNRKGYCRFYQQL  1220
                + +R S R +  A+HR +   M  +R  +HR    +R+ +  R+   K Y R ++QL
Sbjct  375   H*GKTSRISPRQKQSALHRSYCSRMLAIRFFSHRPTCRNRKCRCRRLWRGKRYYRVHKQL  434

Query  1221  RSEQFRTVRVRAGEIRSEPFPGEDQGQDETQLPV*LRPGVRLGESARRQA*RREGD----  1388
             R E FR +  +  EIR +   G  Q +++ Q+ V  R G+R  ++  R  *+ E +
Sbjct  435   RVEHFREILGKP*EIRPDKILGAHQAKNQAQITVRFRHGIR*RKN--RPY*QDERN*KRC  492

Query  1389  AFREKEHTPLHTLQHW*ENVYRSNYGHVDVFHDVREHHAVL*GRRREHQRPQTEAGV  1559
               R++++  L T+QHW EN++RS++G   V  DV  HH+ +*    + +RPQTE+ +
Sbjct  493   LLRQEKYPTLLTVQHWQENLHRSDHGDHYVVCDVC*HHSRV*CGSSQPRRPQTESSL  549
```

```
> ta_transcript75634_1
Length=1029

 Score =   149 bits (376),  Expect = 4e-37, Method: Compositional matrix adjust.
 Identities = 176/477 (37%), Positives = 261/477 (55%), Gaps = 14/477 (3%)
 Frame = +3

Query  153   YRESTPSGRPRVALPGLH*AIEEIRRHfqrqigqrrlrgr*qfiSDQGSFKSKRQCgggt  332
```

```
             ++ S+P  + RVA+PG+H A   +  H QR      + G  QF +DQG  + +R+    T
Sbjct  79    WKSSSPR-KARVAVPGVHGAG*GVWGHLQRDSWLHEMPGGQQFGADQGGAEPEREILRWT  137

Query  333   tglpalp*alrrrpE*LSGPVRLVQSSAAQEEPGEAALRSQTAhrllrqnrhrrhl*ICR  512
               LP +P  LRRR E* +  + LVQ   AQEEPG + LR Q AHR LR++R RRHL   R
Sbjct  138   ARLPEVPQTLRRRQE*FTSAM*LVQPPGAQEEPGASPLRPQAAHRQLREDRQRRHLRSYR  197

Query  513   ANSNPqridqqi*rqHRSEADTDEVRHEHVLKLHV*rpf**rrpripEDCR-SLRRDILG  689
             A+ +PQ  D  +  Q + +A   + R+EHV  LHV R       R  +  R   RR++LG
Sbjct  198   AHPDPQAGDPSVRSQRQPQALPHDHRYEHV*PLHVRRALRS*HRRGVQKSRRPFRRNLLG  257

Query  690   DQPGLRCRFSAVAGAVLQKAHGEALQLVPGHPILHLIEDRGATRNKLRHGSSGEGLSGRP  869
             DQP +RC   AVA  VLQ+ HG+AL+LVPGHP LH  EDR    ++  +    +GL G
Sbjct  258   DQPRIRC*LPAVARTVLQETHGQALRLVPGHPFLHSFEDRRKKGDESGN*RPRKGLLGWL  317

Query  870   PQGATRRPDHG*EHYYLYARRLPRGAFVCW*LSHAVSDRSRQRPGGRQEDTSGNRRGDQR  1049
                A+RR D   EH +L+A   P  +     L HA+ D   QR G  +++    +R  QR
Sbjct  318   APSASRRSDS*QEHNHLHA*GFPWRSLFRRQLGHALFDSCSQRSGSSEKN---QKRD*QR  374

Query  1050  ---QKTRRSHRPQPFAVHRGHHLGMSPVRVLAHRAARGHRERQYIRVRNRKGYCRFYQQL  1220
                + +R S R +  A+HR +   M  +R  +HR    +R+ +  R+   K Y R ++QL
Sbjct  375   H*GKTSRISPRQKQSALHRSYCSRMLAIRFFSHRPTCRNRKCRCRRLWRGKRYYRVHKQL  434

Query  1221  RSEQFRTVRVRAGEIRSEPFPGEDQGQDETQLPV*LRPGVRLGESARRQA*RREGD----  1388
             R E FR +  +  EIR +   G  Q +++ Q+ V  R G+R  ++  R  *+ E +
Sbjct  435   RVEHFREILGKP*EIRPDKILGAHQAKNQAQITVRFRHGIR*RKN--RPY*QDERN*KRC  492

Query  1389  AFREKEHTPLHTLQHW*ENVYRSNYGHVDVFHDVREHHAVL*GRRREHQRPQTEAGV  1559
               R++++  L T+QHW EN++RS++G   V  DV  HH+ +*    + +RPQTE+ +
Sbjct  493   LLRQEKYPTLLTVQHWQENLHRSDHGDHYVVCDVC*HHSRV*CGSSQPRRPQTESSL  549


> ta_transcript75632_1
Length=1032

 Score =   149 bits (376),  Expect = 4e-37, Method: Compositional matrix adjust.
 Identities = 176/477 (37%), Positives = 261/477 (55%), Gaps = 14/477 (3%)
 Frame = +3

Query  153   YRESTPSGRPRVALPGLH*AIEEIRRHfqrqigqrrlrgr*qfiSDQGSFKSKRQCgggt  332
             ++ S+P  + RVA+PG+H A   +  H QR      + G  QF +DQG  + +R+    T
Sbjct  79    WKSSSPR-KARVAVPGVHGAG*GVWGHLQRDSWLHEMPGGQQFGADQGGAEPEREILRWT  137

Query  333   tglpalp*alrrrpE*LSGPVRLVQSSAAQEEPGEAALRSQTAhrllrqnrhrrhl*ICR  512
               LP +P  LRRR E* +  + LVQ   AQEEPG + LR Q AHR LR++R RRHL   R
Sbjct  138   ARLPEVPQTLRRRQE*FTSAM*LVQPPGAQEEPGASPLRPQAAHRQLREDRQRRHLRSYR  197

Query  513   ANSNPqridqqi*rqHRSEADTDEVRHEHVLKLHV*rpf**rrpripEDCR-SLRRDILG  689
             A+ +PQ  D  +  Q + +A   + R+EHV  LHV R       R  +  R   RR++LG
Sbjct  198   AHPDPQAGDPSVRSQRQPQALPHDHRYEHV*PLHVRRALRS*HRRGVQKSRRPFRRNLLG  257

Query  690   DQPGLRCRFSAVAGAVLQKAHGEALQLVPGHPILHLIEDRGATRNKLRHGSSGEGLSGRP  869
             DQP +RC   AVA  VLQ+ HG+AL+LVPGHP LH  EDR    ++  +    +GL G
Sbjct  258   DQPRIRC*LPAVARTVLQETHGQALRLVPGHPFLHSFEDRRKKGDESGN*RPRKGLLGWL  317

Query  870   PQGATRRPDHG*EHYYLYARRLPRGAFVCW*LSHAVSDRSRQRPGGRQEDTSGNRRGDQR  1049
                A+RR D   EH +L+A   P  +     L HA+ D   QR G  +++    +R  QR
Sbjct  318   APSASRRSDS*QEHNHLHA*GFPWRSLFRRQLGHALFDSCSQRSGSSEKN---QKRD*QR  374

Query  1050  ---QKTRRSHRPQPFAVHRGHHLGMSPVRVLAHRAARGHRERQYIRVRNRKGYCRFYQQL  1220
                + +R S R +  A+HR +   M  +R  +HR    +R+ +  R+   K Y R ++QL
Sbjct  375   H*GKTSRISPRQKQSALHRSYCSRMLAIRFFSHRPTCRNRKCRCRRLWRGKRYYRVHKQL  434

Query  1221  RSEQFRTVRVRAGEIRSEPFPGEDQGQDETQLPV*LRPGVRLGESARRQA*RREGD----  1388
             R E FR +  +  EIR +   G  Q +++ Q+ V  R G+R  ++  R  *+ E +
Sbjct  435   RVEHFREILGKP*EIRPDKILGAHQAKNQAQITVRFRHGIR*RKN--RPY*QDERN*KRC  492

Query  1389  AFREKEHTPLHTLQHW*ENVYRSNYGHVDVFHDVREHHAVL*GRRREHQRPQTEAGV  1559
               R++++  L T+QHW EN++RS++G   V  DV  HH+ +*    + +RPQTE+ +
Sbjct  493   LLRQEKYPTLLTVQHWQENLHRSDHGDHYVVCDVC*HHSRV*CGSSQPRRPQTESSL  549


> ta_transcript75629_1
Length=1040

 Score =   149 bits (375),  Expect = 4e-37, Method: Compositional matrix adjust.
 Identities = 176/477 (37%), Positives = 261/477 (55%), Gaps = 14/477 (3%)
```

```
 Frame = +3

Query  153   YRESTPSGRPRVALPGLH*AIEEIRRHfqrqigqrrlrgr*qfiSDQGSFKSKRQCgggt  332
             ++ S+P  + RVA+PG+H A   +  H QR      + G  QF +DQG  + +R+    T
Sbjct  79    WKSSSPR-KARVAVPGVHGAG*GVWGHLQRDSWLHEMPGGQQFGADQGGAEPEREILRWT  137

Query  333   tglpalp*alrrrpE*LSGPVRLVQSSAAQEEPGEAALRSQTAhrllrqnrhrrhl*ICR  512
               LP +P  LRRR E* +  + LVQ   AQEEPG + LR Q AHR LR++R RRHL   R
Sbjct  138   ARLPEVPQTLRRRQE*FTSAM*LVQPPGAQEEPGASPLRPQAAHRQLREDRQRRHLRSYR  197

Query  513   ANSNPqridqqi*rqHRSEADTDEVRHEHVLKLHV*rpf**rrpripEDCR-SLRRDILG  689
             A+ +PQ  D  +  Q + +A   + R+EHV  LHV R       R  +  R   RR++LG
Sbjct  198   AHPDPQAGDPSVRSQRQPQALPHDHRYEHV*PLHVRRALRS*HRRGVQKSRRPFRRNLLG  257

Query  690   DQPGLRCRFSAVAGAVLQKAHGEALQLVPGHPILHLIEDRGATRNKLRHGSSGEGLSGRP  869
             DQP +RC   AVA  VLQ+ HG+AL+LVPGHP LH  EDR    ++  +    +GL G
Sbjct  258   DQPRIRC*LPAVARTVLQETHGQALRLVPGHPFLHSFEDRRKKGDESGN*RPRKGLLGWL  317

Query  870   PQGATRRPDHG*EHYYLYARRLPRGAFVCW*LSHAVSDRSRQRPGGRQEDTSGNRRGDQR  1049
                A+RR D   EH +L+A   P  +     L HA+ D   QR G  +++    +R  QR
Sbjct  318   APSASRRSDS*QEHNHLHA*GFPWRSLFRRQLGHALFDSCSQRSGSSEKN---QKRD*QR  374

Query  1050  ---QKTRRSHRPQPFAVHRGHHLGMSPVRVLAHRAARGHRERQYIRVRNRKGYCRFYQQL  1220
                + +R S R +  A+HR +   M  +R  +HR    +R+ +  R+   K Y R ++QL
Sbjct  375   H*GKTSRISPRQKQSALHRSYCSRMLAIRFFSHRPTCRNRKCRCRRLWRGKRYYRVHKQL  434

Query  1221  RSEQFRTVRVRAGEIRSEPFPGEDQGQDETQLPV*LRPGVRLGESARRQA*RREGD----  1388
             R E FR +  +  EIR +   G  Q +++ Q+ V  R G+R  ++  R  *+ E +
Sbjct  435   RVEHFREILGKP*EIRPDKILGAHQAKNQAQITVRFRHGIR*RKN--RPY*QDERN*KRC  492

Query  1389  AFREKEHTPLHTLQHW*ENVYRSNYGHVDVFHDVREHHAVL*GRRREHQRPQTEAGV  1559
               R++++  L T+QHW EN++RS++G   V  DV  HH+ +*    + +RPQTE+ +
Sbjct  493   LLRQEKYPTLLTVQHWQENLHRSDHGDHYVVCDVC*HHSRV*CGSSQPRRPQTESSL  549


> ta_transcript75630_1
Length=1037

 Score =   149 bits (375),  Expect = 4e-37, Method: Compositional matrix adjust.
 Identities = 176/477 (37%), Positives = 261/477 (55%), Gaps = 14/477 (3%)
 Frame = +3

Query  153   YRESTPSGRPRVALPGLH*AIEEIRRHfqrqigqrrlrgr*qfiSDQGSFKSKRQCgggt  332
             ++ S+P  + RVA+PG+H A   +  H QR      + G  QF +DQG  + +R+    T
Sbjct  79    WKSSSPR-KARVAVPGVHGAG*GVWGHLQRDSWLHEMPGGQQFGADQGGAEPEREILRWT  137

Query  333   tglpalp*alrrrpE*LSGPVRLVQSSAAQEEPGEAALRSQTAhrllrqnrhrrhl*ICR  512
               LP +P  LRRR E* +  + LVQ   AQEEPG + LR Q AHR LR++R RRHL   R
Sbjct  138   ARLPEVPQTLRRRQE*FTSAM*LVQPPGAQEEPGASPLRPQAAHRQLREDRQRRHLRSYR  197

Query  513   ANSNPqridqqi*rqHRSEADTDEVRHEHVLKLHV*rpf**rrpripEDCR-SLRRDILG  689
             A+ +PQ  D  +  Q + +A   + R+EHV  LHV R       R  +  R   RR++LG
Sbjct  198   AHPDPQAGDPSVRSQRQPQALPHDHRYEHV*PLHVRRALRS*HRRGVQKSRRPFRRNLLG  257

Query  690   DQPGLRCRFSAVAGAVLQKAHGEALQLVPGHPILHLIEDRGATRNKLRHGSSGEGLSGRP  869
             DQP +RC   AVA  VLQ+ HG+AL+LVPGHP LH  EDR    ++  +    +GL G
Sbjct  258   DQPRIRC*LPAVARTVLQETHGQALRLVPGHPFLHSFEDRRKKGDESGN*RPRKGLLGWL  317

Query  870   PQGATRRPDHG*EHYYLYARRLPRGAFVCW*LSHAVSDRSRQRPGGRQEDTSGNRRGDQR  1049
                A+RR D   EH +L+A   P  +     L HA+ D   QR G  +++    +R  QR
Sbjct  318   APSASRRSDS*QEHNHLHA*GFPWRSLFRRQLGHALFDSCSQRSGSSEKN---QKRD*QR  374

Query  1050  ---QKTRRSHRPQPFAVHRGHHLGMSPVRVLAHRAARGHRERQYIRVRNRKGYCRFYQQL  1220
                + +R S R +  A+HR +   M  +R  +HR    +R+ +  R+   K Y R ++QL
Sbjct  375   H*GKTSRISPRQKQSALHRSYCSRMLAIRFFSHRPTCRNRKCRCRRLWRGKRYYRVHKQL  434

Query  1221  RSEQFRTVRVRAGEIRSEPFPGEDQGQDETQLPV*LRPGVRLGESARRQA*RREGD----  1388
             R E FR +  +  EIR +   G  Q +++ Q+ V  R G+R  ++  R  *+ E +
Sbjct  435   RVEHFREILGKP*EIRPDKILGAHQAKNQAQITVRFRHGIR*RKN--RPY*QDERN*KRC  492

Query  1389  AFREKEHTPLHTLQHW*ENVYRSNYGHVDVFHDVREHHAVL*GRRREHQRPQTEAGV  1559
               R++++  L T+QHW EN++RS++G   V  DV  HH+ +*    + +RPQTE+ +
Sbjct  493   LLRQEKYPTLLTVQHWQENLHRSDHGDHYVVCDVC*HHSRV*CGSSQPRRPQTESSL  549


> ta_transcript75633_1
Length=1031
```

```
 Score =   149 bits (375),  Expect = 4e-37, Method: Compositional matrix adjust.
 Identities = 176/477 (37%), Positives = 261/477 (55%), Gaps = 14/477 (3%)
 Frame = +3

Query  153   YRESTPSGRPRVALPGLH*AIEEIRRHfqrqigqrrlrgr*qfiSDQGSFKSKRQCgggt  332
             ++ S+P  + RVA+PG+H A   +  H QR      + G  QF +DQG  + +R+    T
Sbjct  79    WKSSSPR-KARVAVPGVHGAG*GVWGHLQRDSWLHEMPGGQQFGADQGGAEPEREILRWT  137

Query  333   tglpalp*alrrrpE*LSGPVRLVQSSAAQEEPGEAALRSQTAhrllrqnrhrrhl*ICR  512
               LP +P  LRRR E* +  + LVQ   AQEEPG + LR Q AHR LR++R RRHL   R
Sbjct  138   ARLPEVPQTLRRRQE*FTSAM*LVQPPGAQEEPGASPLRPQAAHRQLREDRQRRHLRSYR  197

Query  513   ANSNPqridqqi*rqHRSEADTDEVRHEHVLKLHV*rpf**rrpripEDCR-SLRRDILG  689
             A+ +PQ  D  +  Q + +A   + R+EHV  LHV R       R  +  R   RR++LG
Sbjct  198   AHPDPQAGDPSVRSQRQPQALPHDHRYEHV*PLHVRRALRS*HRRGVQKSRRPFRRNLLG  257

Query  690   DQPGLRCRFSAVAGAVLQKAHGEALQLVPGHPILHLIEDRGATRNKLRHGSSGEGLSGRP  869
             DQP +RC   AVA  VLQ+ HG+AL+LVPGHP LH  EDR    +   +    +GL G
Sbjct  258   DQPRIRC*LPAVARTVLQETHGQALRLVPGHPFLHSFEDRRKKGDESGN*RPRKGLLGWL  317

Query  870   PQGATRRPDHG*EHYYLYARRLPRGAFVCW*LSHAVSDRSRQRPGGRQEDTSGNRRGDQR  1049
                A+RR D   EH +L+A   P  +      L HA+ D   QR G  +++     +R  QR
Sbjct  318   APSASRRSDS*QEHNHLHA*GFPWRSLFRRQLGHALFDSCSQRSGSSEKN---QKRD*QR  374

Query  1050  ---QKTRRSHRPQPFAVHRGHHLGMSPVRVLAHRAARGHRERQYIRVRNRKGYCRFYQQL  1220
                + +R S R +  A+HR +   M  +R  +HR    +R+ +  R+   K Y R ++QL
Sbjct  375   H*GKTSRISPRQKQSALHRSYCSRMLAIRFFSHRPTCRNRKCRCRRLWRGKRYYRVHKQL  434

Query  1221  RSEQFRTVRVRAGEIRSEPFPGEDQGQDETQLPV*LRPGVRLGESARRQA*RREGD----  1388
             R E FR +  +  EIR +   G  Q +++ Q+ V  R G+R  ++  R  *+ E +
Sbjct  435   RVEHFREILGKP*EIRPDKILGAHQAKNQAQITVRFRHGIR*RKN--RPY*QDERN*KRC  492

Query  1389  AFREKEHTPLHTLQHW*ENVYRSNYGHVDVFHDVREHHAVL*GRRREHQRPQTEAGV  1559
               R++++  L T+QHW EN++RS++G   V  DV  HH+ +*    + +RPQTE+ +
Sbjct  493   LLRQEKYPTLLTVQHWQENLHRSDHGDHYVVCDVC*HHSRV*CGSSQPRRPQTESSL  549


> ta_transcript75631_1
Length=1034

 Score =   149 bits (375),  Expect = 5e-37, Method: Compositional matrix adjust.
 Identities = 176/477 (37%), Positives = 261/477 (55%), Gaps = 14/477 (3%)
 Frame = +3

Query  153   YRESTPSGRPRVALPGLH*AIEEIRRHfqrqigqrrlrgr*qfiSDQGSFKSKRQCgggt  332
             ++ S+P  + RVA+PG+H A   +  H QR      + G  QF +DQG  + +R+    T
Sbjct  79    WKSSSPR-KARVAVPGVHGAG*GVWGHLQRDSWLHEMPGGQQFGADQGGAEPEREILRWT  137

Query  333   tglpalp*alrrrpE*LSGPVRLVQSSAAQEEPGEAALRSQTAhrllrqnrhrrhl*ICR  512
               LP +P  LRRR E* +  + LVQ   AQEEPG + LR Q AHR LR++R RRHL   R
Sbjct  138   ARLPEVPQTLRRRQE*FTSAM*LVQPPGAQEEPGASPLRPQAAHRQLREDRQRRHLRSYR  197

Query  513   ANSNPqridqqi*rqHRSEADTDEVRHEHVLKLHV*rpf**rrpripEDCR-SLRRDILG  689
             A+ +PQ  D  +  Q + +A   + R+EHV  LHV R       R  +  R   RR++LG
Sbjct  198   AHPDPQAGDPSVRSQRQPQALPHDHRYEHV*PLHVRRALRS*HRRGVQKSRRPFRRNLLG  257

Query  690   DQPGLRCRFSAVAGAVLQKAHGEALQLVPGHPILHLIEDRGATRNKLRHGSSGEGLSGRP  869
             DQP +RC   AVA  VLQ+ HG+AL+LVPGHP LH  EDR    ++  +    +GL G
Sbjct  258   DQPRIRC*LPAVARTVLQETHGQALRLVPGHPFLHSFEDRRKKGDESGN*RPRKGLLGWL  317

Query  870   PQGATRRPDHG*EHYYLYARRLPRGAFVCW*LSHAVSDRSRQRPGGRQEDTSGNRRGDQR  1049
                A+RR D   EH +L+A   P  +      L HA+ D   QR G  +++     +R  QR
Sbjct  318   APSASRRSDS*QEHNHLHA*GFPWRSLFRRQLGHALFDSCSQRSGSSEKN---QKRD*QR  374

Query  1050  ---QKTRRSHRPQPFAVHRGHHLGMSPVRVLAHRAARGHRERQYIRVRNRKGYCRFYQQL  1220
                + +R S R +  A+HR +   M  +R  +HR    +R+ +  R+   K Y R ++QL
Sbjct  375   H*GKTSRISPRQKQSALHRSYCSRMLAIRFFSHRPTCRNRKCRCRRLWRGKRYYRVHKQL  434

Query  1221  RSEQFRTVRVRAGEIRSEPFPGEDQGQDETQLPV*LRPGVRLGESARRQA*RREGD----  1388
             R E FR +  +  EIR +   G  Q +++ Q+ V  R G+R  ++  R  *+ E +
Sbjct  435   RVEHFREILGKP*EIRPDKILGAHQAKNQAQITVRFRHGIR*RKN--RPY*QDERN*KRC  492

Query  1389  AFREKEHTPLHTLQHW*ENVYRSNYGHVDVFHDVREHHAVL*GRRREHQRPQTEAGV  1559
               R++++  L T+QHW EN++RS++G   V  DV  HH+ +*    + +RPQTE+ +
Sbjct  493   LLRQEKYPTLLTVQHWQENLHRSDHGDHYVVCDVC*HHSRV*CGSSQPRRPQTESSL  549
```

```
************************************************************************************************
**********************************************************************

Query= gi|163838677:199-1815 Bombyx mori cytochrome P450 monooxygenase (Cyp306a1), mRNA

Length=1617
                                                                      Score     E
Sequences producing significant alignments:                          (Bits)  Value

  ta_transcript56351_1                                                  184   2e-49


> ta_transcript56351_1
Length=766

 Score =   184 bits (468),  Expect = 2e-49, Method: Compositional matrix adjust.
 Identities = 172/527 (33%), Positives = 262/527 (50%), Gaps = 7/527 (1%)
 Frame = +3

Query  42    VLDFQKNKGMAEFAPRTLGVTYRRLFAFH*SLSSTYHLDKFV*NIRSYLRSQNGQHICCS  221
             +L   K+K MA     +  V YRRL AF     +   +   V*NI   +R   GQ +
Sbjct  110   LLGV*KDKRMANITTWSHRVAYRRLLAFSRPQPTASDIGAIV*NIWPNVRH*YGQRLYSG  169

Query  222   VI*S*TCRRYVLKRQFFWTSTSLLNTWPYEWKWNYLCRRRFVEGPKEINNIVVEKFWNE*  401
              +*     R + +RQ   TS S+LN   Y WKW  LC RRFVE  +E +N VVE+ W+E
Sbjct  170   SV*PQADTRSLRQRQLQRTSASVLNARNYAWKWYNLC*RRFVERSEEADNNVVEELWHEQ  229

Query  402   AQCFPRKIGKTNRFRSIRNF---GKYRKNF*CCLGPSSYADEFFRKRCQ*DNIRL*VST*  572
             A    R+  +TNR R +R     G+  + F    G  ++A +F RK CQ*+ +R  V +
Sbjct  230   A*RLARQT*ETNRCRRLRITP*RGEK*RYF---NGSGTHAHQFTRKCCQ*NYVRFQVPSR  286

Query  573   R*NMAMVSSNTGGRMP*DGSRRCCKLLALYTPCFAINTKNN*SSSPWTGTDAYFVRKHDR  752
               N+A+V SN   R+P*DG  RC  L A+++  F ++ KN+      +G++   V KH +
Sbjct  287   *QNLALVPSNPRRRVP*DGRSRCR*LFAIHSVLFIVDKKNDGYIVTRSGSNTQTVCKHHQ  346

Query  753   *TKKNVGLREA*GSRICSSRKPFKTISKWPYQMHKIQQSLSEHRAFL*SQYSYSD*RR-L  929
             *  +N G  +   S  CS+    +      Y+M ++  +   H   L        *RR +
Sbjct  347   *ASQNAGFGDTQRS*ACSTC*LIQ*AP*RSYKMCEV**TCIRHGGTLFRP*DSYC*RR*V  406

Query  930   HTG*FSVGAKETI*EWRPNGTVYER*TVTFSTSGYVRCRTGYYIGDFSLVFAIHGFVSRR  1109
             + G    G +E +*+WR    + ER T       YV    G++     +V  I+G VS R
Sbjct  407   YLGQLFDGTEEKV*KWRRISLLCERRTNALPPGRYVWSGFGHHFCHIVVVLVIYGSVS*R  466

Query  1110  TGRNT*RNLIRISI*R*C**FKVTSSYGSNL*NSEDSIDCSSGNTPWLYRGRLLG*LQNP  1289
              G  T  + + I   R      +T   G  L*++++SI C  G++ W+Y   L+G L +
Sbjct  467   AGNCTKGDFVCIPRRRPSRERSITKDDGCYL*DTKNSIHCPCGHSSWMYSRHLVGKL*SS  526

Query  1290  KKCHGDPIAVGYSHGS*CLGRTRKIQTA*IFGSGW*ST*ASRIHSVSNW*ADVSG*RTVP  1469
              +C+G    VG ++GS C+G +  + T  + S   S+* +RI++ SN *A+V  *R  P
Sbjct  527   *RCYGSAFTVGTAYGSRCMGGS*GV*TKSVPRSRRDSS*TTRIYTFSNR*ANVPW*RAFP  586

Query  1470  YVVVWPRK*TIQKAAYSTRIKNTDSRRDAWNRRCYVGTSSGEILLRT  1610
             +VV+W    T+   AY T  + +  R DA    C+   +  EILLR+
Sbjct  587   HVVMWIGSATVPAQAYKTSFQPSV*RGDAGLGGCHSCATCCEILLRS  633

************************************************************************************************
*********************************************************************


Query= gi|112983259:39-1592 Bombyx mori cytochrome P450 302A1 (Cyp302a1),mRNA

Length=1554
                                                                      Score     E
Sequences producing significant alignments:                          (Bits)  Value

  ta_transcript46148_1                                                  210   9e-59
  ta_transcript46149_1                                                  196   3e-54


> ta_transcript46148_1
Length=700

 Score =   210 bits (535),  Expect = 9e-59, Method: Compositional matrix adjust.
 Identities = 179/497 (36%), Positives = 277/497 (56%), Gaps = 2/497 (0%)
 Frame = -1
```

```
Query  1542  WNKV**YRFVWFIYKQCLHSKLLAGPLDFKLTN*PH*DNANILLGQSSCNTRTGPVTKRQ  1363
             W K+**+R VWFI ++CL+S++ A P D KL+  P * + N+LL QS  N R+   +KR+
Sbjct  14    WTKL**HRLVWFIDQECLNSQITAHPFDVKLSKYP**SDTNVLLSQSPGNARSWSKSKRK  73

Query  1362  AQKRMNGLVLR*TTHPTFRLEG*GVPYKLRQP**RHVLRHYNSSLRNGISF*NYILLQPS  1183
              Q+ M  LV+ * + P+ RLE  G+   LR     H+L +Y    R  ++  + I LQ
Sbjct  74    TQEWM*ILVIW*PSQPSLRLELQGLSNILR*STCYHILSYYRGPFRYKVTLQHNIFLQ*P  133

Query  1182  TYSNRYRIKSQTLFNTASHV*SFRKNVSGYLIIFLW**CM*LFKHFVLASLITGYMVQYE  1003
              +SNR+R++    F+ A++V  F         IF        F  F+L   + G ++Q +
Sbjct  134   PHSNRHRVEP*AFFDAATNVTCFGHCFRCKN*IF*RHESHRFFVQFLLTGWVAGKVIQRK  193

Query  1002  ACRVGccidcsh*ninhhshdILVIQLGLLKKCF*QRFIVFTIIGFEEINLSNQYICGNF  823
             +C + CC+D  H NI+HH+  +L +Q+GLL +   +RFI      F ++N   Q  C N
Sbjct  194   SCGIRCCVDRCHQNIDHHAGYVLQVQVGLLYE*AQERFICDCAFLFVKVNSLVQDFCCNL  253

Query  822   F*IDLRIHEFLV*WSTKHFPEKSFIKLHDPRI*SECCRYRFAAATLGILLAQSETIETFV  643
               I L + + ++    +  PE+SF++LHD  + +ECC Y  AAA + ILLA  E +E+ +
Sbjct  254   GQIVLGVRQLVIERCPEEPPEESFVQLHDATVGAECCCYGSAAAGVSILLASGEAVESLI  313

Query  642   KSYYTYDF*Vkai*kr*kiIFR*HLVSFDPLNEFFCDIIGVRDVSFNTLWTREILLKYAS  463
             + +  Y F V++I  R KI+   +    +P++EF  D IG  +V  +TL T ++ LK A
Sbjct  314   EPHQPYHFQVQSIQIRQKIVVFRNFHLLNPVDEFSNDFIGCFNVLSDTLRTCKVFLKSAP  373

Query  462   ESPPIgsvgrkksssvVHIRFIGSVVVHSGMTAPRWIAVVLSEHRLNViriinmd*idnr  283
              SPP G+V  K+SSSVV +R   +VV+HS M A RW+ V+L EH  +V+R+ ++   D
Sbjct  374   *SPPFGAVSGKQSSSVVKVRLAQAVVLHSNMAASRWMPVILPEHGGDVVRVKHVQQPDAG  433

Query  282   fFSN*ATITSPIQYCILI*SFGVIVTN*R*VFMKRTNNGIASRSRYIFEIHEIISLFIDL  103
                    +  P Q  +L+*SFG IVTN   + ++ +   +  R+R I E H +I +
Sbjct  434   SLLGQTAVPPPCQPSVLV*SFGTIVTNQWEILIQSSY*WVTFRARNIVEFH-VIYIVSGA  492

Query  102   TGMFT-THKIFRRSSVN  55
             TG  T TH+ F R S N
Sbjct  493   TGSSTFTHRFFTRKSPN  509


> ta_transcript46149_1
Length=574

 Score =   196 bits (497),  Expect = 3e-54, Method: Compositional matrix adjust.
 Identities = 163/446 (37%), Positives = 252/446 (57%), Gaps = 0/446 (0%)
 Frame = -1

Query  1542  WNKV**YRFVWFIYKQCLHSKLLAGPLDFKLTN*PH*DNANILLGQSSCNTRTGPVTKRQ  1363
             W K+**+R VWFI ++CL+S++ A P D KL+  P * + N+LL QS  N R+   +KR+
Sbjct  14    WTKL**HRLVWFIDQECLNSQITAHPFDVKLSKYP**SDTNVLLSQSPGNARSWSKSKRK  73

Query  1362  AQKRMNGLVLR*TTHPTFRLEG*GVPYKLRQP**RHVLRHYNSSLRNGISF*NYILLQPS  1183
              Q+ M  LV+ * + P+ RLE  G+   LR     H+L +Y    R  ++  + I LQ
Sbjct  74    TQEWM*ILVIW*PSQPSLRLELQGLSNILR*STCYHILSYYRGPFRYKVTLQHNIFLQ*P  133

Query  1182  TYSNRYRIKSQTLFNTASHV*SFRKNVSGYLIIFLW**CM*LFKHFVLASLITGYMVQYE  1003
              +SNR+R++    F+ A++V  F         IF        F  F+L   + G ++Q +
Sbjct  134   PHSNRHRVEP*AFFDAATNVTCFGHCFRCKN*IF*RHESHRFFVQFLLTGWVAGKVIQRK  193

Query  1002  ACRVGccidcsh*ninhhshdILVIQLGLLKKCF*QRFIVFTIIGFEEINLSNQYICGNF  823
             +C + CC+D  H NI+HH+  +L +Q+GLL +   +RFI      F ++N   Q  C N
Sbjct  194   SCGIRCCVDRCHQNIDHHAGYVLQVQVGLLYE*AQERFICDCAFLFVKVNSLVQDFCCNL  253

Query  822   F*IDLRIHEFLV*WSTKHFPEKSFIKLHDPRI*SECCRYRFAAATLGILLAQSETIETFV  643
               I L + + ++    +  PE+SF++LHD  + +ECC Y  AAA + ILLA  E +E+ +
Sbjct  254   GQIVLGVRQLVIERCPEEPPEESFVQLHDATVGAECCCYGSAAAGVSILLASGEAVESLI  313

Query  642   KSYYTYDF*Vkai*kr*kiIFR*HLVSFDPLNEFFCDIIGVRDVSFNTLWTREILLKYAS  463
             + +  Y F V++I  R KI+   +    +P++EF  D IG  +V  +TL T ++ LK A
Sbjct  314   EPHQPYHFQVQSIQIRQKIVVFRNFHLLNPVDEFSNDFIGCFNVLSDTLRTCKVFLKSAP  373

Query  462   ESPPIgsvgrkksssvVHIRFIGSVVVHSGMTAPRWIAVVLSEHRLNViriinmd*idnr  283
              SPP G+V  K+SSSVV +R   +VV+HS M A RW+ V+L EH  +V+R+ ++   D
Sbjct  374   *SPPFGAVSGKQSSSVVKVRLAQAVVLHSNMAASRWMPVILPEHGGDVVRVKHVQQPDAG  433

Query  282   fFSN*ATITSPIQYCILI*SFGVIVT  205
                    +  P Q  +L+*SFG IVT
Sbjct  434   SLLGQTAVPPPCQPSVLV*SFGTIVT  459
```

```
****************************************************************************************************
*********************************************************************
Query= gi|163838681:81-1541 Bombyx mori cytochrome P450 (Cyp315a1), mRNA

Length=1461
                                                                      Score     E
Sequences producing significant alignments:                          (Bits)  Value

  ta_transcript32151_1                                                  138   2e-34
  ta_transcript32147_1                                                  138   2e-34

> ta_transcript32151_1
Length=638

 Score =   138 bits (348),  Expect = 2e-34, Method: Compositional matrix adjust.
 Identities = 143/422 (34%), Positives = 217/422 (51%), Gaps = 7/422 (2%)
 Frame = -1

Query  1395  SYSIHIRIVHRFQVKIFHNLTD*FCHL*HSHLFTDT*SGAEGKWQCGRWYMIY*ILLSLI  1216
             SY  +    V   Q+++  NL    F  L HS+  T+  S  + KW+      M++   LS +
Sbjct  138   SYRFYFSFVCGLQIEVI*NLLYYFGQLQHSYFLTNARS*TKCKWERC*RDMVFQRHLSRV  197

Query  1215  VPVPSIRQKLVCFAKILFVSTRCID*SYYWSPFLEQIRSQNTLFG*NSCKKWCYRI*SH-  1039
             + VPS+ Q+     AK+L +S R ++*+Y WS F + + S   T+F    SC+KW Y I SH
Sbjct  198   ISVPSVWQEFSGPAKVLLISARSVN*TYDWSSFFQCVWS*RTIFWQYSCEKWSYWIESHR  257

Query  1038  Clfnnifhvlvvvvhfs*dfsII*K*K*YPK*CISCRISSGDDKVSNNSCY*F---IRHI  868
                +      ++    +F      II K      P+ CIS RIS   + KVS    CY F    I H
Sbjct  258   FFQHIPHVTIIFRTNFIFYGFIINKQMKCPQSCISARISGSNHKVS---CYSFNHCIAHS  314

Query  867   FIL*SFYQTVTSVFCVQHFVYNFLSDRDGIINGLFEISPHF*IPSLAEFYG*SI*FCSC*  688
             F +       T++        HF  NF+S       ++  F++ P    + +L     G SI  F SC
Sbjct  315   FAVQFSN*TISLRLHR*HFFNNFMS*SKR*VHTFFKLPPLLSV*TL*PMNGKSIKFSSCL  374

Query  687   EYFLDSLRKQ*Q*HLVFWRGL**CTIPLKNC*NNVSGKSIKLRFQVRREVATLSSKFPVT  508
             +       RK       + +    **      +N   NN+S K +K RF +R E     + + P+
Sbjct  375   KNLFHFFRKTC*EFFIMFGCC**RFSAKQNVNNNIS*KFVKFRF*IRDETLLFTFRLPIV  434

Query  507   YKVKHSCSYRSS*PYI*IFSE*MFVHNSSINQPILSVHKEKssf*sv*fFIQYPRFW*NM  328
             Y++++S   YR S P+I IFS *MFV++ ++    P   SVH+EKSS   S+*F  I YPR   *NM
Sbjct  435   YELRNSVFYRRSQPFISIFS**MFVYSPTVI*PFFSVHEEKSSLKSI*FLI*YPRLR*NM  494

Query  327   CRIFSF*I*E*TFHVSWVADKDKFCLSC*SLVEYRS*LLVTHVDIFV*VFSSTCRK*VKF  148
              RIFSF + +   FH   W+   ++      S   + VEY + L ++ + IF+     S+ C K ++
Sbjct  495   *RIFSFQVQKQ*FH*LWITYEN*RTFSTQAFVEYWTQLFMSLIYIFMQPSSTACHKKIQL  554

Query  147   CS  142
              S
Sbjct  555   RS  556


> ta_transcript32147_1
Length=665

 Score =   138 bits (348),  Expect = 2e-34, Method: Compositional matrix adjust.
 Identities = 143/422 (34%), Positives = 217/422 (51%), Gaps = 7/422 (2%)
 Frame = -1

Query  1395  SYSIHIRIVHRFQVKIFHNLTD*FCHL*HSHLFTDT*SGAEGKWQCGRWYMIY*ILLSLI  1216
             SY  +    V   Q+++  NL    F  L HS+  T+  S  + KW+      M++   LS +
Sbjct  165   SYRFYFSFVCGLQIEVI*NLLYYFGQLQHSYFLTNARS*TKCKWERC*RDMVFQRHLSRV  224

Query  1215  VPVPSIRQKLVCFAKILFVSTRCID*SYYWSPFLEQIRSQNTLFG*NSCKKWCYRI*SH-  1039
             + VPS+ Q+     AK+L +S R ++*+Y WS F + + S   T+F    SC+KW Y I SH
Sbjct  225   ISVPSVWQEFSGPAKVLLISARSVN*TYDWSSFFQCVWS*RTIFWQYSCEKWSYWIESHR  284

Query  1038  Clfnnifhvlvvvvhfs*dfsII*K*K*YPK*CISCRISSGDDKVSNNSCY*F---IRHI  868
                +      ++    +F      II K      P+ CIS RIS   + KVS    CY F    I H
Sbjct  285   FFQHIPHVTIIFRTNFIFYGFIINKQMKCPQSCISARISGSNHKVS---CYSFNHCIAHS  341

Query  867   FIL*SFYQTVTSVFCVQHFVYNFLSDRDGIINGLFEISPHF*IPSLAEFYG*SI*FCSC*  688
             F +       T++        HF  NF+S       ++  F++ P    + +L     G SI  F SC
Sbjct  342   FAVQFSN*TISLRLHR*HFFNNFMS*SKR*VHTFFKLPPLLSV*TL*PMNGKSIKFSSCL  401

Query  687   EYFLDSLRKQ*Q*HLVFWRGL**CTIPLKNC*NNVSGKSIKLRFQVRREVATLSSKFPVT  508
             +       RK       + +    **      +N   NN+S K +K RF +R E     + + P+
```

```
Sbjct  402   KNLFHFFRKTC*EFFIMFGCC**RFSAKQNVNNNIS*KFVKFRF*IRDETLLFTFRLPIV  461

Query  507   YKVKHSCSYRSS*PYI*IFSE*MFVHNSSINQPILSVHKEKssf*sv*fFIQYPRFW*NM  328
             Y++++S  YR S P+I IFS *MFV++ ++  P  SVH+EKSS  S+*F I YPR  *NM
Sbjct  462   YELRNSVFYRRSQPFISIFS**MFVYSPTVI*PFFSVHEEKSSLKSI*FLI*YPRLR*NM  521

Query  327   CRIFSF*I*E*TFHVSWVADKDKFCLSC*SLVEYRS*LLVTHVDIFV*VFSSTCRK*VKF  148
              RIFSF + +  FH  W+  ++    S  + VEY + L ++ + IF+   S+ C K ++
Sbjct  522   *RIFSFQVQKQ*FH*LWITYEN*RTFSTQAFVEYWTQLFMSLIYIFMQPSSTACHKKIQL  581

Query  147   CS  142
              S
Sbjct  582   RS  583
```

****
****

Query= gi|163838669:38-1588 Bombyx mori ecdysone 20-hydroxylase (Cyp314a1),mRNA

Length=1551

```
                                                                      Score     E
Sequences producing significant alignments:                          (Bits)  Value

  ta_transcript86114_1                                                  147   2e-36
  ta_transcript86113_1                                                  146   2e-36
```

> ta_transcript86114_1
Length=1052

```
 Score =   147 bits (370),  Expect = 2e-36, Method: Compositional matrix adjust.
 Identities = 152/414 (37%), Positives = 185/414 (45%), Gaps = 4/414 (1%)
 Frame = +2

Query  233   TK*INCTRLMQICSSVMGQCSWRRRQVVSPWCR*PSGlllrpycvlllrnlTVRPLRSCK  412
             T * NC +L + CS   G+CSWR        WC  P+    RP C          RP RSC+
Sbjct  151   TG*TNCMKLTKTCSGGTGRCSWRAPPAGLQWCPSPTATPSRPCCERRPSAPIARPPRSCR  210

Query  413   CTAEADQTDTHQLDSSMSREKNGTTYGAT*LLI*RVHIQCKISFPN*TPSPMTSWSY*IL  592
              TA A  TDT   D S ++E+NG T G T*    *R    C+ S  +*  S  TS S
Sbjct  211   STAGAAPTDTPPQDLSTNKERNGIT*GRT*RRS*RARRPCRDSCRS*MASATTS*SCCAR  270

Query  593   LGSLMAQYTPSNS*QTEWVWNRYAV*CWVQDLGFSNDGCRDVQWLWLLLSRITFEHRGTL  772
               +  A    S S  TEW  NR+A *CW      S+  CR         LSR        T
Sbjct  271   AAAPTAPSLASTSSPTEWASNRFAR*CWACAWASSSGACRAAPPRSHPLSRCISARNATP  330

Query  773   ITVLRFGNLLLQRSTRHSLKARKLYTR**RS*WRRQNLKPLVWPKTRRYKRFSLRY*KTQ  952
              T  RFG+     RST  S +A++L+T *  S*WRR+         +T    R+S R   T
Sbjct  331   TTARRFGSSRPPRSTGRSSEAKRLFTL*CLS*WRRRATARTAKRRTTACARYSSRSSTTP  390

Query  953   HWTCATRRLPLSTS*LPVLRR*PTVWCFSCTY*VDVQIGKEKLIQNYHRMQCFALKIWQE  1132
              WTCA RR P STS LP   R *PT WCF CT *    + GK      +   R
Sbjct  391   SWTCAIRRPPSSTSSLPASRH*PTAWCFCCTC*AVGRTGKSGSGASCRRPARSVRSRSLP  450

Query  1133  HLRSELRSMKPLDYCLQLHFLQGS*IHR*RLEDTRSRLERLC--SPIRRRLVVEKKTSGA  1306
                S      +P D C Q    L G     R       T      R C  +P+RR  VV ++TSGA
Sbjct  451   RRPSTRPCRRPSDCCRQRPSLPGCWTRRSP*PVTSCLGGRSCYATPVRR--VVARRTSGA  508

Query  1307  PRNIFRSDGLKCRSRTLTR*WLHSDADAGCVQGNDL*NSNYICYSQR*CKNGEW  1468
               + F S G    RSRT     W  S    A C    +    + + I YS R* + GEW
Sbjct  509   RTSSFPSAGPSLRSRTPRPWWRRSAGGAACAPASGSSSCSCISYSPR*FRLGEW  562
```

> ta_transcript86113_1
Length=1061

```
 Score =   146 bits (369),  Expect = 2e-36, Method: Compositional matrix adjust.
 Identities = 152/414 (37%), Positives = 185/414 (45%), Gaps = 4/414 (1%)
 Frame = +2

Query  233   TK*INCTRLMQICSSVMGQCSWRRRQVVSPWCR*PSGlllrpycvlllrnlTVRPLRSCK  412
             T * NC +L + CS   G+CSWR        WC  P+    RP C          RP RSC+
Sbjct  151   TG*TNCMKLTKTCSGGTGRCSWRAPPAGLQWCPSPTATPSRPCCERRPSAPIARPPRSCR  210

Query  413   CTAEADQTDTHQLDSSMSREKNGTTYGAT*LLI*RVHIQCKISFPN*TPSPMTSWSY*IL  592
```

```
              TA A  TDT   D S ++E+NG T G T*   *R    C+ S  +*  S  TS S
Sbjct  211   STAGAAPTDTPPQDLSTNKERNGIT*GRT*RRS*RARRPCRDSCRS*MASATTS*SCCAR  270

Query  593   LGSLMAQYTPSNS*QTEWVWNRYAV*CWVQDLGFSNDGCRDVQWLWLLLSRITFEHRGTL  772
               +  A    S S  TEW  NR+A *CW      S+  CR         LSR        T
Sbjct  271   AAAPTAPSLASTSSPTEWASNRFAR*CWACAWASSSGACRAAPPRSHPLSRCISARNATP  330

Query  773   ITVLRFGNLLLQRSTRHSLKARKLYTR**RS*WRRQNLKPLVWPKTRRYKRFSLRY*KTQ  952
              T  RFG+     RST  S +A++L+T *   S*WRR+          +T     R+S R    T
Sbjct  331   TTARRFGSSRPPRSTGRSSEAKRLFTL*CLS*WRRRATARTAKRRTTACARYSSRSSTTP  390

Query  953   HWTCATRRLPLSTS*LPVLRR*PTVWCFSCTY*VDVQIGKEKLIQNYHRMQCFALKIWQE  1132
              WTCA RR P STS LP   R *PT WCF CT *    + GK      +  R
Sbjct  391   SWTCAIRRPPSSTSSLPASRH*PTAWCFCCTC*AVGRTGKSGSGASCRRPARSVRSRSLP  450

Query  1133  HLRSELRSMKPLDYCLQLHFLQGS*IHR*RLEDTRSRLERLC--SPIRRRLVVEKKTSGA  1306
                 S      +P D C Q    L G     R      T      R C  +P+RR  VV ++TSGA
Sbjct  451   RRPSTRPCRRPSDCCRQRPSLPGCWTRRSP*PVTSCLGGRSCYATPVRR--VVARRTSGA  508

Query  1307  PRNIFRSDGLKCRSRTLTR*WLHSDADAGCVQGNDL*NSNYICYSQR*CKNGEW  1468
               + F S G    RSRT     W  S    A C    +    + + I YS R* + GEW
Sbjct  509   RTSSFPSAGPSLRSRTPRPWWRRSAGGAACAPASGSSSCSCISYSPR*FRLGEW  562


************************************************************************************************
**********************************************************************
```

Query= gi|156119419:112-1737 Bombyx mori cytochrome P450 18a1 (Cyp18a1),mRNA

```
Length=1626
                                                                      Score     E
Sequences producing significant alignments:                          (Bits)  Value

  ta_transcript28089_1                                                  363    4e-114
  ta_transcript62517_1                                                  179    7e-48
  ta_transcript66869_1                                                  131    9e-32
  ta_transcript66866_1                                                  131    2e-31


> ta_transcript28089_1
Length=848

 Score =  363 bits (932),  Expect = 4e-114, Method: Compositional matrix adjust.
 Identities = 225/511 (44%), Positives = 334/511 (65%), Gaps = 3/511 (1%)
 Frame = -1

Query  1527  FRCNCSTYYALQRR*CVTFSHFQVELVHHTRKEHEHLFAGQYVAQTHSSADPEWHEIFRT  1348
             FR N    +   +Q R         QVEL+HH R+EH+HL A ++VAQ H++    E HE   R+
Sbjct  288   FRGNGRAHDTVQGRQRRARLGLQVELMHHAREEHKHLHAREHVAQAHAATHAEGHEELRS  347

Query  1347  SYLAGRVDESTRIEFVWFIPEIRIHVHAVNKWYNLRTRRNLIAVDLHIPSRRMCGCQRNN  1168
             S LA   VDE   RI+  V   +P++R+HVHAV+   ++LR     N  + V ++I  S   M    +R+N
Sbjct  348   SDLASLVDEPARIKLVRLVPQVRVHVHAVD*RHHLRACGNSVPV*VNILSG*MRSGKRHN  407

Query  1167  A**S*RFQNCSFGVWKTRYVVNRRETFSANDRIEFFLYTPFHVWVSQHEYYHPQKRRLYG  988
             A   +   F++  SF  VW+   ++  N  R+      +   I+F  L+   FHV  V++H+   HPQK   L G
Sbjct  408   ARHAKSFEDGSFRVWQVGHIFNCRKPLVTDHGIQFSLHATFHVRVAKHKNDHPQKG*LDG  467

Query  987   FHTSREKISKYLF*L*FMISSFEEFAGPALALRLLDFN*VDIDKVSGIIDVQSFAVLVYQ  808
             FHTSR+++++  LF  L  FMI  +F+       A      LLD  +  V+I+K+SG++D++  FA+  V  Q
Sbjct  468   FHTSRKQVAENLFQLQFMIPAFK*LPSSAFCFSLLDLDKVNINKISGVVDIECFAMFVDQ  527

Query  807   CLIEREHFIAIFCDlfls*sfSR*IIIQRNVFSVVNFSEKSHSFFDKMIESLEAYIAVTK  628
              L+E  EH  +A++      LS    F+R*I+++  NVFS++NFSE+SH+FFD+++ES  E   +++  +
Sbjct  528   GLVELEHLLAVPHYALLSLGFTR*IVVKGNVFSIMNFSEESHAFFDEIVESREPLVSMAE  587

Query  627   TDRHRDTADNVGHAQRKERIYVNRCALRSMQAYNQLVDFILDSILH*FFAVSHVSHAELP  448
             + RH  + AD  V +    K+  +Y+N   A   ++    +Q  V+FIL+S   H  F AVSH+  HA+
Sbjct  588   SHRHGNPADYVRYH*GKQGVYINGRAFGAV*IGHQFVNFILNSSFHDFLAVSHIGHAKFA  647

Query  447   *FFVQEPSLIFPEAALAIDDAKTI*SMH*GSAGTTGEFFASEGFSDNFVVTHNDGQLVse  268
              F  ++EP+L   P+AALAIDD+++    +H   SAG  GE  FASE  F+D   VV   + GQLV+E
Sbjct  648   QFLMKEPALTLPQAALAIDDSESFKCVHQRSAGAAGELFASERFADEAVVAEHHGQLVAE  707

Query  267   srreesSIVSGQLKERLVPDSEKR*VADDGWSPRSRGQLANVTY*LQELSQQQRRAGYND  88
             SRRE+SS+  + QL+ERLVP  + +R  VAD  G  SPR+R  QL   + +   ++   QQQ+RA
Sbjct  708   SRREQSSVAARQLQERLVPHAHEREVADHGRSPRTRRQLPQLLHQRRQSHQQQQRAHRQQ  767
```

```
Query  87    QHHRRTRYTIVHHLPKRPQNFRI---RKHSY  4
             Q HR  R T +HHLP+ PQ FRI   R+H Y
Sbjct  768   QRHRSLRRTPIHHLPQAPQ*FRICKDRQHCY  798


> ta_transcript62517_1
Length=712

 Score =   179 bits (454),  Expect = 7e-48, Method: Compositional matrix adjust.
 Identities = 134/460 (29%), Positives = 216/460 (47%), Gaps = 37/460 (8%)
 Frame = +1

Query  205   GVRHKTFLQLARNYGA-LFSARLGNQLTIVMSDYKIIREAFRREEFTGRPSTPLMHTL--  375
             G + +   QLA+ Y   +   +LG++L +V+   K IR+ F   EF GRP++  +
Sbjct  89    GSQWQALSQLAKQYSTQVLGLKLGSELVVVVYGEKNIRQIFTGLEFEGRPNSFFIRLRCL  148

Query  376   -DGLGIINSEGRLWKNQRRFLHEKLREFGMTYMGNGKKLMEDRIKNEIHELIVSLHRAQG  552
                LGI  ++G LW+  R F  ++LR  G      GK  ME  I+ E+  ++  +   +
Sbjct  149   GKRLGITFTDGPLWREHRCFTVKQLRNVGF-----GKSAMEKEIQGELRNILNYIKNNEN  203

Query  553   APIDVNPLLALCVSNVICGITMSVRFSNGDVRFERLNHLIE------EGMRLFGEVHYGE  714
              PI    +LA  V NV+       R     +  ERLN L++      +   + G   +
Sbjct  204   -PISPRKILAKSVMNVLWKFVAGER-----IEEERLNSLLDLLNARSKAFSMAGG--WLN  255

Query  715   YIPLYNYLPGKALAQEKVAKNRDEMFAFYQTLIDEHRETLDINNARDLIDVYLIEIEKAK  894
               P   ++         + K  +E+    +  I +H+         D I  ++ EI   K
Sbjct  256   QFPWSRFILPDISGYSLIKKMNEEISNIIEEAIQKHKS--QSVEGSDYIYSFIEEINLNK  313

Query  895   SEGRAGELFEGRDHELQLKQILGDLFSAGMETIKSSLLWMIVFMLRNPDVKRRVQEELDA  1074
             +             E QLK I  DL  AG +T  ++L + ++  LR+  +++++ +E+
Sbjct  314   TSFT----------EQQLKTICLDLLIAGSQTTSNALEFALLAALRHKHIQQKIHDEISK  363

Query  1075  VIGRERLPSIDDISSLPYTETTILETLRLSSIVPLATTHSPTRDVQINGYKIPAGSQVIP  1254
             VIG   +P   D   L YT   +LE  R  +IVP+A           I GY IP  + ++
Sbjct  364   VIGNN-IPCWADNYRLTYTSAFLLEVQRFYTIVPIAGPRRVLETTTIEGYTIPKETTILI  422

Query  1255  LINCVHMDPNLWDEPNKFNPSRFIDATGKIRRPEYFMPFGVGRRMCLGDVLARKEMFMFF  1434
                 +H DP LWDEP+ F P RFIDA G +R  E+  PFG+GRR C GD LA+  +F+ F
Sbjct  423   ATGDLHCDPELWDEPHIFKPERFIDANGTLRSVEHMYPFGLGRRRCPGDSLAKSFIFITF  482

Query  1435  SCMMHQFDLEMAEGDALPSLEGIVGATIAPKAFRVKFLAR  1554
               +M ++ +E   G   PS   ++G   AP+A+   F+ +
Sbjct  483   VGIMQKYFIECRNG-TYPSNNPVIGLIAAPEAYTADFIPK  521


> ta_transcript66869_1
Length=614

 Score =   131 bits (329),  Expect = 9e-32, Method: Compositional matrix adjust.
 Identities = 78/228 (34%), Positives = 121/228 (53%), Gaps = 7/228 (3%)
 Frame = +1

Query  799   YQTLIDEHRETLDINNARDLIDVYLIEIEKAKSEGRAGELFEGRDHELQLKQILGDLFSA  978
             +Q L++E  ET D +  R  +D+Y+ +++K + +  A   F      +     L    S
Sbjct  196   FQKLVNEALETHDESYDRHFLDIYISKMKK-EQKNNAKTTFSVEQLIMTCVDYLLPTASG  254

Query  979   GMETIKSSLLWMIVFMLRNPDVKRRVQEELDAVIGRERLPSIDDISSLPYTETTILETLR  1158
             G ET+ + LL  I   L  P+V+ RV EE+D V+GRERLP++DD  ++PYTE  I E++R
Sbjct  255   G-ETLLTILLEHI---LMQPEVQDRVHEEIDRVVGRERLPTLDDRHNMPYTEACIRESMR  310

Query  1159  LSSIVPLATTHSPTRDVQINGYKIPAGSQVIPLINCVHMDPNLWDEPNKFNPSRFIDATG  1338
             +  +VP+   H  T+     GY +P    +      +  D  LW +P  F P RFI+  G
Sbjct  311   MDPLVPMGVPHRSTKATTFAGYDLPENCALAVNYVGLGFDKELWGDPEVFRPERFIE-NG  369

Query  1339  KIR-RPEYFMPFGVGRRMCLGDVLARKEMFMFFSCMMHQFDLEMAEGD  1479
             +I    +  +PFG GRR+C G+  +R+ MF  F+ +M  F    A G
Sbjct  370   RISPAKDKSLPFGAGRRLCAGETFSRQIMFQVFAGLMQVFSFSPAGGQ  417


> ta_transcript66866_1
Length=703

 Score =   131 bits (329),  Expect = 2e-31, Method: Compositional matrix adjust.
 Identities = 78/228 (34%), Positives = 121/228 (53%), Gaps = 7/228 (3%)
 Frame = +1

Query  799   YQTLIDEHRETLDINNARDLIDVYLIEIEKAKSEGRAGELFEGRDHELQLKQILGDLFSA  978
```

```
             +Q L++E  ET D +  R  +D+Y+ +++K + +  A   F      +     L    S
Sbjct  196   FQKLVNEALETHDESYDRHFLDIYISKMKK-EQKNNAKTTFSVEQLIMTCVDYLLPTASG  254

Query  979   GMETIKSSLLWMIVFMLRNPDVKRRVQEELDAVIGRERLPSIDDISSLPYTETTILETLR  1158
             G ET+ + LL  I   L  P+V+ RV EE+D V+GRERLP++DD  ++PYTE  I E++R
Sbjct  255   G-ETLLTILLEHI---LMQPEVQDRVHEEIDRVVGRERLPTLDDRHNMPYTEACIRESMR  310

Query  1159  LSSIVPLATTHSPTRDVQINGYKIPAGSQVIPLINCVHMDPNLWDEPNKFNPSRFIDATG  1338
             +  +VP+   H  T+     GY +P    +      +  D  LW +P  F P RFI+  G
Sbjct  311   MDPLVPMGVPHRSTKATTFAGYDLPENCALAVNYVGLGFDKELWGDPEVFRPERFIE-NG  369

Query  1339  KIR-RPEYFMPFGVGRRMCLGDVLARKEMFMFFSCMMHQFDLEMAEGD  1479
             +I    +  +PFG GRR+C G+  +R+ MF  F+ +M  F    A G
Sbjct  370   RISPAKDKSLPFGAGRRLCAGETFSRQIMFQVFAGLMQVFSFSPAGGQ  417

************************************************************************************************
***********************************************************************
Query= gi|193082986:270-1907 Bombyx mori alpha-esterase 40 (ae40), mRNA

Length=1638
                                                                      Score     E
Sequences producing significant alignments:                          (Bits)  Value

  ta_transcript75783_1                                                  306   1e-93
  ta_transcript58915_1                                                  302   3e-93
  ta_transcript58914_1                                                  302   8e-93
  ta_transcript48580_1                                                  306   8e-93
  ta_transcript48579_1                                                  306   3e-92
  ta_transcript84821_1                                                  265   4e-79
  ta_transcript84818_1                                                  265   5e-78
  ta_transcript84816_1                                                  265   8e-77
  ta_transcript29751_1                                                  238   3e-70
  ta_transcript48393_1                                                  235   9e-68
  ta_transcript49309_1                                                  167   6e-44
  ta_transcript15565_1                                                  151   2e-39
  ta_transcript20178_1                                                  150   2e-38
  ta_transcript74072_1                                                  149   3e-37
  ta_transcript74071_1                                                  149   6e-37
  ta_transcript46992_1                                                  130   4e-31

> ta_transcript75783_1
Length=733

 Score =   306 bits (784),  Expect = 1e-93, Method: Compositional matrix adjust.
 Identities = 199/535 (37%), Positives = 298/535 (56%), Gaps = 30/535 (6%)
 Frame = +1

Query  73    VVIEQGILSGKISPD---GSFFEYVGIPYATSNI-NTRFKAPLPPPSWNGVFKAVEETSM  240
             V + +GIL G++  +   GS++ + GIP+A   + + RFKAP+P   W GV  A +   +
Sbjct  126   VKVSEGILQGELVQNEFGGSYYSFKGIPFAEPPVGDLRFKAPIPAKPWKGVKTATKFGPI  185

Query  241   CPQASIIGI---IGSEDCLTINVYIPALARKP---LPVMVYVHGGAFVLGSGGKLLYAPD  402
               Q  +       GSEDCL +NVY P +  KP   LPVMV++HGG +  GSG   +Y P+
Sbjct  186   NYQFDVFKKDPPSGSEDCLYLNVYTPEI--KPITLLPVMVWIHGGGYFWGSGNDDVYGPE  243

Query  403   FLVKHDVILVTFNYRLGALGFMCLGIKDAPGNAGLKDQIAALRWVKKNIAAFGGDVENIT  582
             FLV+H VILVTFNYRLG LGF+ L   + PGNAG+KDQ+AAL+WVK NI  FGGD ENIT
Sbjct  244   FLVRHGVILVTFNYRLGVLGFLSLDTAEVPGNAGMKDQVAALKWVKSNIHFFGGDPENIT  303

Query  583   LFGQSAGGTSVSLLLASEATSGLFKKAIVMSGSAISSWAINRQPIWIANLVAKELGYNTD  762
             +FG+SAG  +VSL L S  + GLFK+AI+ SGSA + WA   +    +  +A++LG  TD
Sbjct  304   IFGESAGAGAVSLHLLSPMSKGLFKRAIIQSGSATAYWAQAFEIKEKSIALARKLGLVTD  363

Query  763   NPNELYEIFSKIPFKELVRAKPEKPLGKYLDTQLLHLPCIEKNIPDVEPALTDLPYNLLT  942
             +  +LYE F  I  + L+         +  +  +   P IEK+    E  LTD  Y +L
Sbjct  364   DDQKLYEFFGNISKELLINISVPVTFAEQEEVNIEFAPTIEKDFGQ-ERFLTDDSYKILQ  422

Query  943   KKPKK-IPVMYGSASKEGLLII-SKDNEETVSERDSKYLFASDLEFQTEE----EAEKED  1104
             +   K + VM G    EG+L + S +  +   ER + +L A        E    +  K
Sbjct  423   QGIHKGVDVMMGYVEDEGVLYLNSLEILDKEIERINNFLEALVPRNMKREIPIKQQLKVG  482

Query  1105  NKARQLYFNGQRMSMNNIMNISDLMSHLYFEIPPILESEITLSTTADVAVFNYYFNYSGG  1284
              K ++ YF+ + ++ N+   I+  +S   F    +++S+  ++ + +   F Y F
Sbjct  483   RKMKKFYFDNKLVTRNDWRTIARYISLNVFSY-DVIQSQKNIAKSQN--SFFYKFTCKSK  539

Query  1285  RNFLKYLTG----FKNETGACHGDELLYLFRGDLWPFPISRKDK--KMIDWMTKLWSNFA  1446
             RNF K +       K+    CH D+L YLF        +++   +  ++ID +TKLW+NFA
```

```
Sbjct  540   RNFFKKVFTDDEITKDVDVVCHCDDLAYLFPVKFMNQSVAKDSREFQLIDKVTKLWTNFA  599

Query  1447  KYGDPTPEDASDLPIKWVPSKRNYLKFLYIEDDLSMGTIPSPEAYRLWKYMYEKY  1611
             KYG+PTP+++  L +KW P    +  +L I +DL + T P  E  + W+ +Y +Y
Sbjct  600   KYGNPTPDNS--LGVKWEPFTLQHQHYLDIGEDLVLKTKPDDEDIQFWEELYREY  652


> ta_transcript58915_1
Length=606

 Score =   302 bits (773),  Expect = 3e-93, Method: Compositional matrix adjust.
 Identities = 197/534 (37%), Positives = 279/534 (52%), Gaps = 28/534 (5%)
 Frame = +1

Query  70    EVVIEQGILSGK----ISPDGSFFEYVGIPYATSNINT-RFKAPLPPPSWNGVFKAVEET  234
             +V +EQG L G+    +S DG FF + GIPYA   +   RFKAP PP SW GV KA +
Sbjct  31    KVQVEQGWLEGEQLDSVSGDGQFFSFKGIPYAAPPVGKLRFKAPQPPVSWQGVRKATQHG  90

Query  235   SMCPQASIIG---IIGSEDCLTINVYIPALA-RKPLPVMVYVHGGAFVLGSGGKLLYAPD  402
               CPQ  I     I GSEDCL +NVY   ++ + PLPVMV++HGG F  GSG +  Y  D
Sbjct  91    PKCPQRDIFSKQIIPGSEDCLYLNVYTKDISPKSPLPVMVFIHGGGFKSGSGDEDFYGGD  150

Query  403   FLVKHDVILVTFNYRLGALGFMCLGIKDAPGNAGLKDQIAALRWVKKNIAAFGGDVENIT  582
             FLV H V+LVT NYRL ALGF+CL  ++ PGNAGLKDQ+AAL+WV+KNI AFGGD  N+T
Sbjct  151   FLVHHGVVLVTINYRLDALGFLCLDTEEVPGNAGLKDQVAALKWVQKNIFAFGGDPTNVT  210

Query  583   LFGQSAGGTSVSLLLASEATSGLFKKAIVMSGSAISSWAINRQPIWIANLVAKELGYNTD  762
             +FG+SAGG+S +L + S  + GLFK+AI MSG     W+I  +P   A  + K LG+ T+
Sbjct  211   IFGESAGGSSTALHVLSPLSKGLFKRAIPMSGVPFCDWSIPFEPRKRAFTLGKILGFETE  270

Query  763   NPNELYEIFSKIPFKELVRAKP------EKPLGKYLDTQLLHL-PCIEKNIPDVEPALTD  921
             +P  L E    +P ++ V   P      EK    Y   ++ H  P +EKN       +T+
Sbjct  271   DPKALLEYLQSLPAEKFVDTNPTIMGFEEK---SYNMLKMYHFTPVVEKNFGQ-HHFMTE  326

Query  922   LPYNLLTK-KPKKIPVMYGSASKEGLLIISK--DNEETVSERDSKYLFASDLEFQTEEEA  1092
              P  L +     + V+ G+  +E ++ I     D  +        ++    L   T  +
Sbjct  327   EPLEALKQGHVNDVDVLIGNTDQETIVGIPSFVDLLKMYDRYPEVFVPRKILNKSTPGKI  386

Query  1093  EKEDNKARQLYFNGQRMSMNNIMNISDLMSHLYFEIPPILESEITLSTT-ADVAVFNYYF  1269
              +   + R+ YF  + +   ++        S + F       + + LS       V+ Y F
Sbjct  387   LEIAERIRKHYFGDKPIDKTSMKEAVTYFSEVCFTYDVNKYTNLLLSGKPGSSKVYRYRF  446

Query  1270  NYSGGRNFLKYLTGFKNETGACHGDELLYLFRGDLWPFPISRKDK--KMIDWMTKLWSNF  1443
             +    RN           TGA H ++L+YLF       P+ R  K  KMI     L++NF
Sbjct  447   SCVSERNIFGKYGQEYGITGASHLEDLMYLFDAKSVKLPLDRNSKSYKMIQQTCALFTNF  506

Query  1444  AKYGDPTPEDASDLPIKWVPSKRNYLKFLYIEDDLSMGTIPSPEAYRLWKYMYE  1605
             AKYG+PTP+  S L   W          F+ I D L++G     +A  + W+ +Y+
Sbjct  507   AKYGNPTPD--SSLGFTWPEYDIKDQSFVDIADQLTVGRHLDADAVKFWESIYQ  558


> ta_transcript58914_1
Length=650

 Score =   302 bits (773),  Expect = 8e-93, Method: Compositional matrix adjust.
 Identities = 197/534 (37%), Positives = 279/534 (52%), Gaps = 28/534 (5%)
 Frame = +1

Query  70    EVVIEQGILSGK----ISPDGSFFEYVGIPYATSNINT-RFKAPLPPPSWNGVFKAVEET  234
             +V +EQG L G+    +S DG FF + GIPYA   +   RFKAP PP SW GV KA +
Sbjct  31    KVQVEQGWLEGEQLDSVSGDGQFFSFKGIPYAAPPVGKLRFKAPQPPVSWQGVRKATQHG  90

Query  235   SMCPQASIIG---IIGSEDCLTINVYIPALA-RKPLPVMVYVHGGAFVLGSGGKLLYAPD  402
               CPQ  I     I GSEDCL +NVY   ++ + PLPVMV++HGG F  GSG +  Y  D
Sbjct  91    PKCPQRDIFSKQIIPGSEDCLYLNVYTKDISPKSPLPVMVFIHGGGFKSGSGDEDFYGGD  150

Query  403   FLVKHDVILVTFNYRLGALGFMCLGIKDAPGNAGLKDQIAALRWVKKNIAAFGGDVENIT  582
             FLV H V+LVT NYRL ALGF+CL  ++ PGNAGLKDQ+AAL+WV+KNI AFGGD  N+T
Sbjct  151   FLVHHGVVLVTINYRLDALGFLCLDTEEVPGNAGLKDQVAALKWVQKNIFAFGGDPTNVT  210

Query  583   LFGQSAGGTSVSLLLASEATSGLFKKAIVMSGSAISSWAINRQPIWIANLVAKELGYNTD  762
             +FG+SAGG+S +L + S  + GLFK+AI MSG     W+I  +P   A  + K LG+ T+
Sbjct  211   IFGESAGGSSTALHVLSPLSKGLFKRAIPMSGVPFCDWSIPFEPRKRAFTLGKILGFETE  270

Query  763   NPNELYEIFSKIPFKELVRAKP------EKPLGKYLDTQLLHL-PCIEKNIPDVEPALTD  921
             +P  L E    +P ++ V   P      EK    Y   ++ H  P +EKN       +T+
Sbjct  271   DPKALLEYLQSLPAEKFVDTNPTIMGFEEK---SYNMLKMYHFTPVVEKNFGQ-HHFMTE  326
```

```
Query  922   LPYNLLTK-KPKKIPVMYGSASKEGLLIISK--DNEETVSERDSKYLFASDLEFQTEEEA  1092
              P   L +     + V+ G+  +E ++ I    D  +        ++    L   T  +
Sbjct  327   EPLEALKQGHVNDVDVLIGNTDQETIVGIPSFVDLLKMYDRYPEVFVPRKILNKSTPGKI  386

Query  1093  EKEDNKARQLYFNGQRMSMNNIMNISDLMSHLYFEIPPILESEITLSTT-ADVAVFNYYF  1269
              +   + R+ YF  + +   ++       S + F       + + LS       V+ Y F
Sbjct  387   LEIAERIRKHYFGDKPIDKTSMKEAVTYFSEVCFTYDVNKYTNLLLSGKPGSSKVYRYRF  446

Query  1270  NYSGGRNFLKYLTGFKNETGACHGDELLYLFRGDLWPFPISRKDK--KMIDWMTKLWSNF  1443
             +    RN           TGA H ++L+YLF       P+ R  K  KMI     L++NF
Sbjct  447   SCVSERNIFGKYGQEYGITGASHLEDLMYLFDAKSVKLPLDRNSKSYKMIQQTCALFTNF  506

Query  1444  AKYGDPTPEDASDLPIKWVPSKRNYLKFLYIEDDLSMGTIPSPEAYRLWKYMYE  1605
             AKYG+PTP+  S L   W         F+ I D L++G     +A + W+ +Y+
Sbjct  507   AKYGNPTPD--SSLGFTWPEYDIKDQSFVDIADQLTVGRHLDADAVKFWESIYQ  558


> ta_transcript48580_1
Length=834

 Score =   306 bits (784),  Expect = 8e-93, Method: Compositional matrix adjust.
 Identities = 192/545 (35%), Positives = 287/545 (53%), Gaps = 35/545 (6%)
 Frame = +1

Query  64    APEVVIEQGILSGK-----ISPDGSFFEYVGIPYATSNINT-RFKAPLPPPSWNGVFKAV  225
              P V+ E G L G+      +    +F + GI YA     + RFK P+P   W+GV  A+
Sbjct  153   GPIVMTELGALRGRRVVARTTAQTPYFSFKGIRYAQPPRGSLRFKPPVPLEPWSGVRDAL  212

Query  226   EETSMCPQASII--GIIGSEDCLTINVYIPALARK------PLPVMVYVHGGAFVLGSGG  381
             EE ++CP   ++     G EDCL +NVY PAL  K       L VMV++HGGAF +GSG
Sbjct  213   EEGAVCPHRFMLFDTYKGDEDCLFLNVYTPALPEKITGFNPKLAVMVWIHGGAFAVGSGN  272

Query  382   KLLYAPDFLVKHDVILVTFNYRLGALGFMCLGIKDAPGNAGLKDQIAALRWVKKNIAAFG  561
               LY PD L+  +++LVT NYRLGALGF+ +   +  GN GLKDQ+ AL+WV+ NI  FG
Sbjct  273   AFLYGPDHLMGGNIVLVTLNYRLGALGFLSMENDEVSGNMGLKDQVLALKWVRDNIEYFG  332

Query  562   GDVENITLFGQSAGGTSVSLLLASEATSGLFKKAIVMSGSAISSWAINRQPIWIANLVAK  741
             GD   +T+FG+SAG  SV L + S A+ GLF +AI  SG A+S WA+ R P   A  + +
Sbjct  333   GDASRVTIFGESAGAASVHLHMLSPASKGLFHRAIAQSGLALSPWALTRSPRERAFELGR  392

Query  742   ELGYNTDNPNELYEIFSKIPFKELVRA------KPEKPLGKYLDTQLLHLPCIEKNIPDV  903
             ELG +T++  EL       P + LV+A       P K +  +    L  +P +E ++PD
Sbjct  393   ELGIDTNSTAELLGYLRATPSELLVKAGARLAGAPGKSVDLHSTVALPFVPTVEPDVPDA  452

Query  904   EPALTDLPYNLLTKKPKKIPVMYGSASKEGLLIISKDNE--ETVSERDSKYLFASDLEFQ  1077
                LT  P NL+      +P++ G  ++EG+++  +     + ++E D ++      E
Sbjct  453   --FLTQHPRNLMPG--ADVPLLTGYNAQEGIILFRRLQRYPKLLTELDREFRRVVPPELL  508

Query  1078  TEEEAEKED--NKARQLYFNGQRMSMNNIMNISDLMSHLYFEIPPILES-EITLSTTADV  1248
             T +EA+     +  R  YF  + + + NI ++ DL + + F + P+LE+  I   T
Sbjct  509   TGDEAQNRRVADHIRAFYFQQRPVDIRNIDSLIDLFTDVMF-LRPLLETLRIQGKTNRTS  567

Query  1249  AVFNYYFNYSGGRNFLKYLTGFKNETGACHGDELLYLFRGDLWPFPISRKDKKMI--DWM  1422
               + Y F + G     K + G  N  GACHGDE+ YLF      + +     ++     M
Sbjct  568   PTYLYRFAFDGALGLFKRMLGI-NHPGACHGDEMGYLFYFSRLNYRLDDDSTELAVSKGM  626

Query  1423  TKLWSNFAKYGDPTPEDASD-LPIKWVP-SKRNYLKFLYIEDDLSMGTIPSPEAYRLWKY  1596
              ++W+NFAK G+PT  D  D +  KW P +   +L +L I    +    P     RLW +
Sbjct  627   VRMWTNFAKTGNPTIGDHDDPIDFKWSPVNDTTHLTYLDINGHFTQKQDPEARRVRLWDW  686

Query  1597  MYEKY  1611
             +YE Y
Sbjct  687   LYENY  691


> ta_transcript48579_1
Length=905

 Score =   306 bits (784),  Expect = 3e-92, Method: Compositional matrix adjust.
 Identities = 192/545 (35%), Positives = 287/545 (53%), Gaps = 35/545 (6%)
 Frame = +1

Query  64    APEVVIEQGILSGK-----ISPDGSFFEYVGIPYATS-NINTRFKAPLPPPSWNGVFKAV  225
              P V+ E G L G+      +    +F + GI YA     + RFK P+P   W+GV  A+
Sbjct  153   GPIVMTELGALRGRRVVARTTAQTPYFSFKGIRYAQPPRGSLRFKPPVPLEPWSGVRDAL  212
```

```
Query  226   EETSMCPQASII--GIIGSEDCLTINVYIPALARK------PLPVMVYVHGGAFVLGSGG  381
             EE ++CP    ++     G EDCL +NVY PAL  K       L VMV++HGGAF +GSG
Sbjct  213   EEGAVCPHRFMLFDTYKGDEDCLFLNVYTPALPEKITGFNPKLAVMVWIHGGAFAVGSGN  272

Query  382   KLLYAPDFLVKHDVILVTFNYRLGALGFMCLGIKDAPGNAGLKDQIAALRWVKKNIAAFG  561
               LY PD L+  +++LVT NYRLGALGF+ +   +  GN GLKDQ+ AL+WV+ NI  FG
Sbjct  273   AFLYGPDHLMGGNIVLVTLNYRLGALGFLSMENDEVSGNMGLKDQVLALKWVRDNIEYFG  332

Query  562   GDVENITLFGQSAGGTSVSLLLASEATSGLFKKAIVMSGSAISSWAINRQPIWIANLVAK  741
             GD   +T+FG+SAG  SV L + S A+ GLF +AI  SG A+S WA+ R P   A  + +
Sbjct  333   GDASRVTIFGESAGAASVHLHMLSPASKGLFHRAIAQSGLALSPWALTRSPRERAFELGR  392

Query  742   ELGYNTDNPNELYEIFSKIPFKELVRA------KPEKPLGKYLDTQLLHLPCIEKNIPDV  903
             ELG +T++  EL       P + LV+A       P K +  +    L  +P +E ++PD
Sbjct  393   ELGIDTNSTAELLGYLRATPSELLVKAGARLAGAPGKSVDLHSTVALPFVPTVEPDVPDA  452

Query  904   EPALTDLPYNLLTKKPKKIPVMYGSASKEGLLIISKDNE--ETVSERDSKYLFASDLEFQ  1077
               LT  P NL+      +P++ G  ++EG+++  +     + ++E D ++      E
Sbjct  453   --FLTQHPRNLMPG--ADVPLLTGYNAQEGIILFRRLQRYPKLLTELDREFRRVVPPELL  508

Query  1078  TEEEAEKED--NKARQLYFNGQRMSMNNIMNISDLMSHLYFEIPPILES-EITLSTTADV  1248
             T +EA+     +  R  YF  + + + NI ++ DL + + F + P+LE+  I   T
Sbjct  509   TGDEAQNRRVADHIRAFYFQQRPVDIRNIDSLIDLFTDVMF-LRPLLETLRIQGKTNRTS  567

Query  1249  AVFNYYFNYSGGRNFLKYLTGFKNETGACHGDELLYLFRGDLWPFPISRKDKKMI--DWM  1422
               + Y F + G     K + G  N  GACHGDE+ YLF      + +     ++     M
Sbjct  568   PTYLYRFAFDGALGLFKRMLGI-NHPGACHGDEMGYLFYFSRLNYRLDDDSTELAVSKGM  626

Query  1423  TKLWSNFAKYGDPTPEDASD-LPIKWVP-SKRNYLKFLYIEDDLSMGTIPSPEAYRLWKY  1596
               ++W+NFAK G+PT  D  D +  KW P +   +L +L I    +    P     RLW +
Sbjct  627   VRMWTNFAKTGNPTIGDHDDPIDFKWSPVNDTTHLTYLDINGHFTQKQDPEARRVRLWDW  686

Query  1597  MYEKY  1611
             +YE Y
Sbjct  687   LYENY  691


> ta_transcript84821_1
Length=611

 Score =   265 bits (676),  Expect = 4e-79, Method: Compositional matrix adjust.
 Identities = 157/374 (42%), Positives = 223/374 (60%), Gaps = 17/374 (5%)
 Frame = +1

Query  55    DQPAPEVVIEQGILSGKISPDGSFFEYVGIPYATSNINT-RFKAPLPPPSWNGVFKAVEE  231
             D+ +  V I+QG L G   P+G  F +  IPYAT+     RFKAPLP P W    +AV+
Sbjct  34    DENSRTVEIQQGKLRGYKDPEGGLFTFYNIPYATAPTGKDRFKAPLPAPVWITTREAVDR  93

Query  232   TSMCPQASI--IGIIG---SEDCLTINVYIPALARKPLPVMVYVHGGAFVLGSGGKLLYA  396
               +C Q  +  +GI       EDCL  NVY+P   +K LPV+V+VHGGA++ G G  L
Sbjct  94    GVVCHQIDLAAVGIFNKTKQEDCLIANVYVPDTDKKNLPVVVFVHGGAYLAGFGEVL--T  151

Query  397   PDFLVK-HDVILVTFNYRLGALGFMCLGIKDAPGNAGLKDQIAALRWVKKNIAAFGGDVE  573
             P  +V+ ++VI VTFNYRLG  GF+CLG  DAPGNAG+KD +A LRWVKKNIA+FGG+ +
Sbjct  152   PKHMVRTNNVIYVTFNYRLGVQGFLCLGTPDAPGNAGMKDMVALLRWVKKNIASFGGNPD  211

Query  574   NITLFGQSAGGTSVSLLLASEATSGLFKKAIVMSGSAISSWAINRQPIWIANLVAK-ELG  750
             ++T+ G SAG ++V LLL S+AT GLF KAI  SG+ ++ W++   PI  A  AK E G
Sbjct  212   DVTIDGYSAGSSAVDLLLLSKATKGLFHKAIPESGANVAMWSVQVDPIANAKEYAKSEHG  271

Query  751   YNT-DNPNELYEIFSKIPFKELVRAKPEKPLGKYLDTQLLHLPCIEKNIPDVEPALTDLP  927
             +   D+   L + +  +P+ E+ ++  E           L  PC+E++  VE  L D P
Sbjct  272   FEILDDIYALEDFYKSLPYDEMYKSGMELMFKN--KNNFLFSPCVERDT-GVEKFLDDTP  328

Query  928   YNLLTKKP-KKIPVMYGSASKEGLLIISKDNEETVSERDSKYL--FASDLEFQTEEEAEK  1098
              N+L K    K+PV+YG A+ EGL  +    E  +S+ + K+     DLEF+ EEE  K
Sbjct  329   VNILKKGDYNKVPVLYGFANMEGLFRVGMGYEYFMSQLNDKFSENLPHDLEFKDEEEKRK  388

Query  1099  EDNKARQLYFNGQR  1140
               ++ ++ YF  ++
Sbjct  389   VADEVKKFYFGDKK  402


> ta_transcript84818_1
Length=733

 Score =   265 bits (676),  Expect = 5e-78, Method: Compositional matrix adjust.
```

```
 Identities = 157/374 (42%), Positives = 223/374 (60%), Gaps = 17/374 (5%)
 Frame = +1

Query  55    DQPAPEVVIEQGILSGKISPDGSFFEYVGIPYATSNINT-RFKAPLPPPSWNGVFKAVEE  231
             D+ +  V I+QG L G   P+G  F +  IPYAT+     RFKAPLP P W    +AV+
Sbjct  34    DENSRTVEIQQGKLRGYKDPEGGLFTFYNIPYATAPTGKDRFKAPLPAPVWITTREAVDR  93

Query  232   TSMCPQASI--IGIIG---SEDCLTINVYIPALARKPLPVMVYVHGGAFVLGSGGKLLYA  396
               +C Q  +  +GI      EDCL  NVY+P   +K LPV+V+VHGGA++ G G  L
Sbjct  94    GVVCHQIDLAAVGIFNKTKQEDCLIANVYVPDTDKKNLPVVVFVHGGAYLAGFGEVL--T  151

Query  397   PDFLVK-HDVILVTFNYRLGALGFMCLGIKDAPGNAGLKDQIAALRWVKKNIAAFGGDVE  573
             P  +V+ ++VI VTFNYRLG  GF+CLG  DAPGNAG+KD +A LRWVKKNIA+FGG+ +
Sbjct  152   PKHMVRTNNVIYVTFNYRLGVQGFLCLGTPDAPGNAGMKDMVALLRWVKKNIASFGGNPD  211

Query  574   NITLFGQSAGGTSVSLLLASEATSGLFKKAIVMSGSAISSWAINRQPIWIANLVAK-ELG  750
             ++T+ G SAG ++V LLL S+AT GLF KAI  SG+ ++ W++   PI  A   AK E G
Sbjct  212   DVTIDGYSAGSSAVDLLLLSKATKGLFHKAIPESGANVAMWSVQVDPIANAKEYAKSEHG  271

Query  751   YNT-DNPNELYEIFSKIPFKELVRAKPEKPLGKYLDTQLLHLPCIEKNIPDVEPALTDLP  927
             +   D+   L + +  +P+ E+ ++  E           L  PC+E++   VE  L D P
Sbjct  272   FEILDDIYALEDFYKSLPYDEMYKSGMELMFKN--KNNFLFSPCVERDT-GVEKFLDDTP  328

Query  928   YNLLTKKP-KKIPVMYGSASKEGLLIISKDNEETVSERDSKYL--FASDLEFQTEEEAEK  1098
              N+L K    K+PV+YG A+ EGL  +    E  +S+ + K+      DLEF+ EEE  K
Sbjct  329   VNILKKGDYNKVPVLYGFANMEGLFRVGMGYEYFMSQLNDKFSENLPHDLEFKDEEEKRK  388

Query  1099  EDNKARQLYFNGQR  1140
               ++ ++ YF  ++
Sbjct  389   VADEVKKFYFGDKK  402


> ta_transcript84816_1
Length=947

 Score =   265 bits (676),  Expect = 8e-77, Method: Compositional matrix adjust.
 Identities = 157/374 (42%), Positives = 223/374 (60%), Gaps = 17/374 (5%)
 Frame = +1

Query  55    DQPAPEVVIEQGILSGKISPDGSFFEYVGIPYATSNINT-RFKAPLPPPSWNGVFKAVEE  231
             D+ +  V I+QG L G   P+G  F +  IPYAT+     RFKAPLP P W    +AV+
Sbjct  34    DENSRTVEIQQGKLRGYKDPEGGLFTFYNIPYATAPTGKDRFKAPLPAPVWITTREAVDR  93

Query  232   TSMCPQASI--IGIIG---SEDCLTINVYIPALARKPLPVMVYVHGGAFVLGSGGKLLYA  396
               +C Q  +  +GI      EDCL  NVY+P   +K LPV+V+VHGGA++ G G  L
Sbjct  94    GVVCHQIDLAAVGIFNKTKQEDCLIANVYVPDTDKKNLPVVVFVHGGAYLAGFGEVL--T  151

Query  397   PDFLVK-HDVILVTFNYRLGALGFMCLGIKDAPGNAGLKDQIAALRWVKKNIAAFGGDVE  573
             P  +V+ ++VI VTFNYRLG  GF+CLG  DAPGNAG+KD +A LRWVKKNIA+FGG+ +
Sbjct  152   PKHMVRTNNVIYVTFNYRLGVQGFLCLGTPDAPGNAGMKDMVALLRWVKKNIASFGGNPD  211

Query  574   NITLFGQSAGGTSVSLLLASEATSGLFKKAIVMSGSAISSWAINRQPIWIANLVAK-ELG  750
             ++T+ G SAG ++V LLL S+AT GLF KAI  SG+ ++ W++   PI  A   AK E G
Sbjct  212   DVTIDGYSAGSSAVDLLLLSKATKGLFHKAIPESGANVAMWSVQVDPIANAKEYAKSEHG  271

Query  751   YNT-DNPNELYEIFSKIPFKELVRAKPEKPLGKYLDTQLLHLPCIEKNIPDVEPALTDLP  927
             +   D+   L + +  +P+ E+ ++  E           L  PC+E++   VE  L D P
Sbjct  272   FEILDDIYALEDFYKSLPYDEMYKSGMELMFKN--KNNFLFSPCVERD-TGVEKFLDDTP  328

Query  928   YNLLTKKP-KKIPVMYGSASKEGLLIISKDNEETVSERDSKYL--FASDLEFQTEEEAEK  1098
              N+L K    K+PV+YG A+ EGL  +    E  +S+ + K+      DLEF+ EEE  K
Sbjct  329   VNILKKGDYNKVPVLYGFANMEGLFRVGMGYEYFMSQLNDKFSENLPHDLEFKDEEEKRK  388

Query  1099  EDNKARQLYFNGQR  1140
               ++ ++ YF  ++
Sbjct  389   VADEVKKFYFGDKK  402


> ta_transcript29751_1
Length=506

 Score =   238 bits (608),  Expect = 3e-70, Method: Compositional matrix adjust.
 Identities = 156/412 (38%), Positives = 232/412 (56%), Gaps = 16/412 (4%)
 Frame = +1

Query  73    VVIEQGILSGKISPDGSFFEYVGIPYATS-NINTRFKAPLPPPSWNGVFKAVEETSMCPQ  249
             VVIEQG + G   PDG  + +  IPYAT+     RFKAPL PP W  +  AV+   +CPQ
```

```
Sbjct  33    VVIEQGPVRGYKDPDGGLYVFYNIPYATAPTSKDRFKAPLAPPVWVTIRDAVDREVVCPQ  92

Query  250   ASIIGIIGS--EDCLTINVYIPALARKPLPVMVYVHGGAFVLGSGGKLLYAPDFLVKHDV  423
                 G   +  EDCL  NV++P  + K L V+V VHGG ++ G G +     D +    +
Sbjct  93    MLETGSHKNIQEDCLIANVFVPDTSEKNLSVVVLVHGGGYLSGFGNRQT-PKDMVRDKKL  151

Query  424   ILVTFNYRLGALGFMCLGIKDAPGNAGLKDQIAALRWVKKNIAAFGGDVENITLFGQSAG  603
             I VTFNYRLG  GF+CLG  D PGNAG+KD +A LRWVKKNIA+FGG+ +++T+ G S+G
Sbjct  152   IYVTFNYRLGVHGFLCLGTADVPGNAGMKDMVALLRWVKKNIASFGGNPDDVTIDGHSSG  211

Query  604   GTSVSLLLASEATSGLFKKAIVMSGSAISSWAINRQPIWIANLVAK-ELGYNT-DNPNEL  777
              +SV LLL S+AT GLF KAI  SGS ++ W++   PI  A   A+ E  + + D+   L
Sbjct  212   SSSVDLLLLSKATEGLFHKAIPESGSNVAMWSVQIDPIAHAKEFARDEHSFESGDDIYAL  271

Query  778   YEIFSKIPFKELVRAKPEKPLGKYLDTQLLHLPCIEKNIPDVEPALTDLPYNLL-TKKPK  954
              + +  +PF  + ++         +   +L  PC+E++   VE  L D P N+L +
Sbjct  272   EDFYKTLPFDVMYKSGMSLRYNSSV-KNILFQPCVERDT-GVEKFLDDFPVNILKSGDYN  329

Query  955   KIPVMYGSASKEGLLIIS-----KDNEETVSERDSKYLFASDLEFQTEEEAEKEDNKARQ  1119
             K+P++YG  + EGL+ +S     +     ++E+ S YL  +DL+F+ +EE EK     +Q
Sbjct  330   KVPMLYGFTNMEGLMRVSDFVNYRSFMSDINEKFSDYL-PNDLQFRDKEEKEKVARDVKQ  388

Query  1120  LYFNGQRMSMNNIMNISDLMSHLYFEIPPILESEITLSTTADVAVFNYYFNY  1275
              YF  + ++ + I    D  S   F  P +   ++ +    +  V+ Y F+Y
Sbjct  389   FYFGDKVINNDTIFEYIDYFSDNMFAYPTLRSLKLQVE-AGNNQVYLYQFSY  439


> ta_transcript48393_1
Length=666

 Score =   235 bits (600),  Expect = 9e-68, Method: Compositional matrix adjust.
 Identities = 153/451 (34%), Positives = 245/451 (54%), Gaps = 27/451 (6%)
 Frame = +1

Query  55    DQPAPEVV-IEQGILSGKISPDGSFFEYVGIPYATSNIN-TRFKAPLPPPSWNGVFKAVE  228
             D+P   +V   QG + G   P+G  F +  IPYAT+     +F AP+ PP+W   F+AV+
Sbjct  89    DEPRSRIVETTQGPVRGYRDPEGGLFAFYSIPYATAPSGPLKFTAPITPPTWTEPFEAVD  148

Query  229   ETSMCPQAS---IIGIIGSEDCLTINVYIPALARKPLPVMVYVHGGAFVLGSGGKLLYAP  399
             +  +CPQ        +     DCL  NVY+P      LPV+VYVHGGAF +G G   L  P
Sbjct  149   KRIICPQMKSGFTKQMTMQVDCLIANVYVPDTKETKLPVIVYVHGGAFQVGFGN--LMTP  206

Query  400   DFLVK-HDVILVTFNYRLGALGFMCLGIKDAPGNAGLKDQIAALRWVKKNIAAFGGDVEN  576
               LV+  ++I VTFNYRLGA GF+CLG +DAPGNAGLKDQ+AALRWV KNI   GG+ ++
Sbjct  207   KNLVRSKNIIAVTFNYRLGAHGFLCLGTEDAPGNAGLKDQVAALRWVNKNIIKLGGNPDD  266

Query  577   ITLFGQSAGGTSVSLLLASEATSGLFKKAIVMSGSAISSWAINRQPIWIANLVAKELGYN  756
             +T+ G SAG  SV L+  S++  GLF K I+ SGS++ + +I   P+  A + A  LG+N
Sbjct  267   VTIAGYSAGAISVDLMAISKSAEGLFSKIIIESGSSLFAGSIVTDPLENAKIFAARLGFN  326

Query  757   T-DNPNELYEIFSKIPFKELVRAKPEKPLGKYLD---TQLLHLPCIEKNIPDVEPALTDL  924
               D+   L + +    +  L +       G+++D   + LL  PC+E++  D    L++
Sbjct  327   NIDDVYALEKFYKAASYDSLTK-------GEFMDRPNSTLLFSPCVERD-DDGLAFLSEP  378

Query  925   PYNLLTKKP-KKIPVMYGSASKEGLLI--ISKDNEETVSERDSKYLFASDLEFQTEEEAE  1095
             P N+L +     ++P++YG ++ EGL      +  +  ++E+ S+++   DL F+++ + E
Sbjct  379   PINILKEGSYTQVPMIYGFSNMEGLFREPFFEFWKLKMNEQFSEFI-PGDLHFESKHDRE  437

Query  1096  KEDNKARQLYFNGQRMSMNNIMNISDLMSHLYFEIPPILESEITLSTTADVAVFNYYFNY  1275
             +   + ++ YF    ++  NI+   D    +  + P +   ++ +    D     Y + Y
Sbjct  438   RIAKRIKKFYFWDSPINGENILKYIDYFGDVLVKYPALRTVKMHIQNGHDKL---YLYEY  494

Query  1276  SGGRNFLKYLTGFKNETGACHGDELLYLFRG  1368
             S     +  +    N  GA H  + + +  G
Sbjct  495   SYVDENVPAVIHTTNTRGANHCAQTVAVLDG  525


> ta_transcript49309_1
Length=664

 Score =   167 bits (424),  Expect = 6e-44, Method: Compositional matrix adjust.
 Identities = 165/564 (29%), Positives = 244/564 (43%), Gaps = 106/564 (19%)
 Frame = +1

Query  133   YVGIPYATSNINT-RFKAP--LPPPSWNGVFKAVEETSMCPQAS----------------  255
             YVGIPYA   I+  RF  P  L PP W GV+ A      C Q+
Sbjct  53    YVGIPYAQPPIDRLRFMPPEYLNPPQWEGVYNATIFAPDCMQSDPKKDDVQSTLKKHDEL  112
```

```
Query  256   IIGIIGS-----------EDCLTINVYIPALAR-KPLPVMVYVHGGAFVLGSGGKLLYAP  399
              + ++ S           EDCL +NVY+P   + +  PVMV+ HGG FV GS   +   P
Sbjct  113   FMKLLDSQLETPREKNYSEDCLYLNVYVPDDYKVEGYPVMVWFHGGEFVRGSPNYM--NP  170

Query  400   DFLV-KHDVILVTFNYRLGALGFMCLGIKDAPGNAGLKDQIAALRWVKKNIAAFGGDVEN  576
               LV K  VI V+  YRL   GF      +A GN GL DQ+AAL WVK NI  FGGD EN
Sbjct  171   FHLVLKQKVIFVSVAYRLNIFGFFTTLDHEALGNFGLHDQVAALSWVKTNIEHFGGDPEN  230

Query  577   ITLFGQSAGGTSVSLLLASEATSGLFKKAIVMSGSAIS--SWAINRQPIWIANLVAKELG  750
             I +FG  AG  SV L L S  + GLF KAI MSG+ +S  + AI R+ +   + VA
Sbjct  231   ICIFGHDAGAVSVGLHLVSTYSPGLFHKAIAMSGNVLSPDTVAIARKELITVDRVASAFS  290

Query  751   YNTDNPNELYEIFSKIPFKELVRAKPEKPLGKYLDTQLLHLPCIEKNIPDVE-PALTDLP  927
                    +L +   ++P + L+      PL ++        P ++    ++  P L+D+P
Sbjct  291   CFRKPTFQLLDCLRRVPSQALLDLG--APLAEW-------KPIVDSGFSNISVPFLSDIP  341

Query  928   YNLLTKKP-KKIPVMYGSASKEGLLIISKDNEETVSERDSKYLFASDLEFQTEEEAEKED  1104
               L   +    +PV+ G  + E  L++ K+ ++  +   S          Q E ++ +E+
Sbjct  342   SRLFKDEAFLSVPVLSGYTNMEDALLLVKEGDDESNAGLS----------QREFDSMREE  391

Query  1105  NKARQLYFNGQRMSMNNIMNISDLMSHLYFEIPPILESEITL-------------STTAD  1245
                  +  +       N  +I + ++  Y  IPP     I               +TT
Sbjct  392   VILADITVDNTS-CFTNQHHIQEAVAFFYKPIPPTQNETILRKLFLDFYTDKVHGATTYA  450

Query  1246  VA--------VFNYYFNYSGGRNFLKYLTGFKNE-----TGACHGDELLYLFRGDLWPFP  1386
             +A        V+ Y F+       LK  +    E         H  +L++ F       P
Sbjct  451   LAKYLSSHAPVYLYRFD-------LKPFSDIATEDIPDWVKVAHNYDLIFAFGLPYLALP  503

Query  1387  -----ISRKDKKMIDWMTKLWSNFAKYGDPTPEDASDLPIKWVPSKRNYLKFLYIEDDLS  1551
                    ++DK + D + K+WSNFA Y +PT    S + I+W P +     +L I  D +
Sbjct  504   DDSGKWDKRDKSISDIIMKMWSNFAWYSNPT---NSGVFIEWNPFEIENPGYLII--DRA  558

Query  1552  MGTIPSPE-----AYRLWKYMYEK  1608
               T+ +PE     A+  W   Y K
Sbjct  559   NFTMSTPETINYRAFEFWADFYPK  582


> ta_transcript15565_1
Length=424

 Score =   151 bits (382),  Expect = 2e-39, Method: Compositional matrix adjust.
 Identities = 114/385 (30%), Positives = 198/385 (51%), Gaps = 26/385 (7%)
 Frame = +1

Query  502   GLKDQIAALRWVKKNIAAFGGDVENITLFGQSAGGTSVSLLLASEATSGLFKKAIVMSGS  681
             G+KD +A LRWV +NIAAFGG+  ++TL G SAG  SV LL+ S+A  GLF KAI  SGS
Sbjct  1     GMKDMVALLRWVNRNIAAFGGNPNDVTLEGWSAGSVSVELLMLSKAAKGLFHKAIPESGS  60

Query  682   AISSWAINRQPIWIANLVAK-ELGY-NTDNPNELYEIFSKIPFKELVRAKPEKPLGKYLD  855
             + + +++   PI  A   A+ +LG+ + DN + L E +  +PFK++ +  P
Sbjct  61    SFAPFSVGVDPIGNAKDFARSDLGFKDVDNISALEEFYKTVPFKDMYQQGPL----IIFR  116

Query  856   TQLLHLPCIEKNIPDVEPALTDLPYNLLTKKP-KKIPVMYGSASKEGLLIISKDNE---E  1023
                   PC+E+NI   +  L D PYN+L K    K+P++YG ++KEGL  +    +   E
Sbjct  117   HNFTFTPCVERNIGQ-DVFLDDTPYNILKKGDYMKVPMLYGFSNKEGLYRVDYGYQFFIE  175

Query  1024  TVSERDSKYLFASDLEFQTEEEAEKEDNKARQLYFNGQRMSMNNIMNISDLMSHLYFEIP  1203
              ++++ S YL  +DL+F+ E E E+     ++ YF  +++  + ++    D  +    F  P
Sbjct  176   GMNKKFSDYL-PNDLQFKDENEKEQVATMIKEFYFGNKKIDQDTVLEYIDYFTDSMFAGP  234

Query  1204  PILESEITLSTTADVAVFNYYFNYSGGRNFLKYLTGFKNETGACHGDELLYLFRGDLW--  1377
              + E ++ ++   +  ++ Y F+Y       + +       G+ H  E + +   D W
Sbjct  235   SLKEVQLHVA-AGNNQIYLYQFSYPYENKVPESIK--VKVIGSNHCGETMTVL--DNWLG  289

Query  1378  --PFPISRK-DKKMIDWMTK-LWSNFAKYGDPTPEDASDLPIKWVPSKRNYLKFLYIEDD  1545
                 P   R   D + +  +T+ +W NF K G P P+  S+LP  W  +  +   ++ I +
Sbjct  290   GAEMPAERNSDLEQVRNVTRQIWGNFIKTGKPVPQ-GSNLPT-WPATDGSGSPYMVITNK  347

Query  1546  LSMGTIP-SPEAYRLWKYMYEKYRK  1617
               +   P  P +   W  +Y+++ +
Sbjct  348   PELKGQPFQPMSVMFWTEIYQRHYR  372


> ta_transcript20178_1
Length=614
```

```
 Score =   150 bits (379),  Expect = 2e-38, Method: Compositional matrix adjust.
 Identities = 123/305 (40%), Positives = 150/305 (49%), Gaps = 2/305 (1%)
 Frame = -2

Query  926  GKSVKAGSTSGIFFSIHGKCNSWVSKYFPKGFSGLALTNSLKGILENISYSSFGLSVLYP  747
            GKSV  G +  I FS HG   +W     P GF  L   +S   +LE +  SS G  VL P
Sbjct  275  GKSVMNGFSPSITFSTHGINITWEMYVPPIGFGILTAYSSSIDLLEKML*SSCGFLVL*P  334

Query  746  SSLATRFAIQIG*RLIAQLDIAEPLITIAFLKSPLVASLANSSETDVPPALCPKRVIFST  567
            S+ A R A+ IG   I Q   A PL  IA  KSP     +    +T+  PAL PK V  S
Sbjct  335  SASANRLAVSIGSGCIPQGVTAVPLCIIAL*KSPFDIGASI*KDTEAAPALSPKIVTLSG  394

Query  566  SPPKAAIFFLTHLKAAIWSLSPAFPGASLIPKHIKPKAPSL*LNVTKITSCLTRKSGAYN  387
            SPP A IF  THL+AA  S +P  PG S +P+H  P+  +L*LNVT   S +TR SG
Sbjct  395  SPPNALIFL*THLRAAT*SFNPILPGTSFVPRHKNPRMDNL*LNVTSTISLVTRYSGPCR  454

Query  386  SLPPLPSTNAPP*T*TITGSGFLAKAGMYTFIVKQSSEPIIPIIDACGHIDVSSTALKTP  207
            +L P P+ N P    TITG+ FLA  G+ TF+ +Q S P+             SS A  T
Sbjct  455  NLEPDPNKNPPSWKYTITGNDFLALTGV*TFMTRQFS*PVTIFPIFW*QYICSSIASNTL  514

Query  206  FHDGGGNGALNLVLMLLVAYGIPTYSKNDPSGLILPLKMPCSITTSGAG*STKLVKNSVN  27
            FH G      NL     VA GIP      PSG    PL  P  I T   G  T L+
Sbjct  515  FHLGSSLAT*NLSST--VA*GIPR*F*CVPSGDTDPLNAPELIRTISVGCLTILIAQRDQ  572

Query  26   NTRYF  12
            +T +F
Sbjct  573  STTHF  577


> ta_transcript74072_1
Length=938

 Score =   149 bits (376),  Expect = 3e-37, Method: Compositional matrix adjust.
 Identities = 136/346 (39%), Positives = 164/346 (47%), Gaps = 6/346 (2%)
 Frame = -2

Query  1037  LSDTVSSLSFDIISNPSLLAEPYITGIFFGF-FVSKLYGKSVKAGSTSGIFFSIHGKCNS  861
             LS+ VS   F +   PS L  P I G    F V    G +K GS+  +  S  G  N+
Sbjct  258   LSNVVSFSKFAM-KCPSSLLLPIIMGTLLNFPLVRTS*GIYLKNGSSPSMSISAQGAYNT  316

Query  860   WVSKYFPKGFSGLALTNSLKGILENISYSSFGLSVLYPSSLATRFAIQIG*RLIAQLDIA  681
               +   P     L   +S   +   I   S G SV YP  L  + AI  G    AQ
Sbjct  317   SDTFVLPSFLGTLVNISSSSVLFIKIL*ISCGSSVSYPKVLVIKLAIYTGRNWNAQAARD  376

Query  680   EPLITIAFLKSPLVASLANSSETDVPPALCPKRVIFSTSPPKAAIFFLTHLKAAIWSLSP  501
             +PLI IA  KSP+         +   PPAL PK V  S SPPK  +F  THLKAA WS
Sbjct  377   DPLIAIALWKSPVDIGDKT**DIAAPPALSPKIVTLSGSPPKTLMFL*THLKAATWSFIA  436

Query  500   AFPGASLIPKHIKPKAPSL*LNVTKITSCLTRKSGAYNSLPPLPSTNAPP*T*TITG---  330
             AFPGAS + KH KP   +L*  VTKI    T+  G   +L   P+  APP*T T+TG
Sbjct  437   AFPGASFVLKHRKPCMFNL*FIVTKIIPLDTKYFGP*INLAFEPAKKAPP*TYTMTGNFD  496

Query  329   SGFLAKAGMYTFIVKQSSEPIIPIIDACGHIDVSSTALKTPFHDGGGNGALNLVLMLLVA  150
             SG LA  G+ T  +KQSS P        C H+  SS A K P H GGG GALNL   L V
Sbjct  497   SG-LAP*GV*TLSIKQSS*PKTMFPSPCRHLIFSSMASKIPSHFGGGPGALNLYSELTVP  555

Query  149   YGIPTYSKNDPSGLILPLKMPCSITTSGAG*STKLVKNSVNNTRYF  12
              GIP    +  PS     P   P S+  S  G  T L    ++N  +F
Sbjct  556   *GIPR*EE*TPSADTYPRISPESMRNSSVGCRTNLAAQRLHNITHF  601


> ta_transcript74071_1
Length=1143

 Score =   149 bits (375),  Expect = 6e-37, Method: Compositional matrix adjust.
 Identities = 136/346 (39%), Positives = 164/346 (47%), Gaps = 6/346 (2%)
 Frame = -2

Query  1037  LSDTVSSLSFDIISNPSLLAEPYITGIFFGF-FVSKLYGKSVKAGSTSGIFFSIHGKCNS  861
             LS+ VS   F +   PS L  P I G    F V    G +K GS+  +  S  G  N+
Sbjct  258   LSNVVSFSKFAM-KCPSSLLLPIIMGTLLNFPLVRTS*GIYLKNGSSPSMSISAQGAYNT  316

Query  860   WVSKYFPKGFSGLALTNSLKGILENISYSSFGLSVLYPSSLATRFAIQIG*RLIAQLDIA  681
               +   P     L   +S   +   I   S G SV YP  L  + AI  G    AQ
Sbjct  317   SDTFVLPSFLGTLVNISSSSVLFIKIL*ISCGSSVSYPKVLVIKLAIYTGRNWNAQAARD  376

Query  680   EPLITIAFLKSPLVASLANSSETDVPPALCPKRVIFSTSPPKAAIFFLTHLKAAIWSLSP  501
```

```
             +PLI IA  KSP+        +   PPAL PK V  S SPPK  +F  THLKAA WS
Sbjct  377   DPLIAIALWKSPVDIGDKT**DIAAPPALSPKIVTLSGSPPKTLMFL*THLKAATWSFIA  436

Query  500   AFPGASLIPKHIKPKAPSL*LNVTKITSCLTRKSGAYNSLPPLPSTNAPP*T*TITG---  330
             AFPGAS + KH KP   +L*  VTKI    T+  G   +L   P+  APP*T T+TG
Sbjct  437   AFPGASFVLKHRKPCMFNL*FIVTKIIPLDTKYFGP*INLAFEPAKKAPP*TYTMTGNFD  496

Query  329   SGFLAKAGMYTFIVKQSSEPIIPIIDACGHIDVSSTALKTPFHDGGGNGALNLVLMLLVA  150
             SG LA  G+ T  +KQSS P       C H+  SS A K P H GGG GALNL   L V
Sbjct  497   SG-LAP*GV*TLSIKQSS*PKTMFPSPCRHLIFSSMASKIPSHFGGGPGALNLYSELTVP  555

Query  149   YGIPTYSKNDPSGLILPLKMPCSITTSGAG*STKLVKNSVNNTRYF  12
              GIP   +  PS    P   P S+  S  G  T L    ++N  +F
Sbjct  556   *GIPR*EE*TPSADTYPRISPESMRNSSVGCRTNLAAQRLHNITHF  601


> ta_transcript46992_1
Length=699

 Score =   130 bits (326),  Expect = 4e-31, Method: Compositional matrix adjust.
 Identities = 166/512 (32%), Positives = 255/512 (50%), Gaps = 3/512 (1%)
 Frame = -1

Query  1563  YCTHRQIILYV*KL*IISFAGNPFNW*ITCIFWCRITIFSKIRPQLCHPINHFLVFSTYG  1384
             Y TH  + +Y+ KL I    G P +W*  C  W RIT+F K+ P  CH +N+ L+ +
Sbjct  92    YGTHFHMFIYIEKLHIPLHTGFPLDW*FAC-HWSRITVFGKVCP*FCHLVNYDLISTYLW  150

Query  1383  KWP*ISSEQVQ*FISMTSSGLIFEPSKVL*Eisttaiieiiiencniscca*CYL*L*NR  1204
               P ++ + +   I+MTS+ L+ + SK +    ++  I          S        L*
Sbjct  151   PRPELTFK*IHQIIAMTSTFLM-QISKSVFHHISSTTIFKE*IINRCSTVFQYCFGL*YS  209

Query  1203  RYLKV*VTH*VRYIHNIIHTHSLTIKv*lssfivlffsfflglef*I*SK*IFRISLRYC  1024
             R  K+ ++  V YI +  H     IK+ +S F+  F S F+  +F I*SK +
Sbjct  210   RNFKIKLSSEV*YIISQFHRVFFIIKIKISDFLCNFTSLFIRSKFKI*SKQMQIFLFFKF  269

Query  1023  FFIVFRYYKQSFFASGTVHYRDFLrffr**iIW*IS*GWFNIWYIFFDTRQMQ*LGV*IF  844
              FI    +K+SF    TV+Y +  R F   ++ *I    FN       TRQM *LG+  F
Sbjct  270   LFIFIIKHKKSFLIQSTVNYWNMFRIFSNKVVR*IICYCFNS*NFALYTRQMH*LGIKNF  329

Query  843   PQRFLRLSSY*FFKGYFREYFi*liriisivs*LFSYKICNPDWLTINRPTRYCRTTHND  664
                  +  +   F   F +YF+* + I SI S* F      P  + I  PT    +  ND
Sbjct  330   SIWSFKSYTKIVFVRSF*KYFV*FMWIFSIKS*FFGNSTSYPHRVDIQYPTCKTGSALND  389

Query  663   SLFKEPTSSFAGQ*Q*D*CSSSTLSEKSYILNIPTKSCDILLDPS*SCYLVFKSSVSWSV  484
                K    S     Q*   ++S L + SYI+ I T+S +I LDP     YLV KS ++  +
Sbjct  390   DFIK*SICSITCVKQ*CSYTTSALPKNSYIIRITTESSNIFLDPFKCGYLVSKSGITRCL  449

Query  483   FNTQAHKA*SSKSVVKCH*NYIMFD*EIRSV*QFTTTSEHKCSTVNIDHYW*WFSR*SGY  304
             F+TQA ++   K ++KC *N  MFD ++RS+ +F++ S  K +++N +H+  WF     +
Sbjct  450   FDTQAKESKYIKPIIKCD*NDTMFDKKLRSIEKFSSISVSKTTSMNENHHRQWFCCFDRH  509

Query  303   VHVYS*TVF*TNNPNNRCLRTHRCFFDCFENSIP*WWRQWRLEPRINVTSRIRYTDVFEK  124
             +++Y *T++ + +    + +RT   F  CF  + P  W  W L+    + + IRYT+VF
Sbjct  510   IYIYF*TIYFSPSFE*KEMRTGS-FLCCFVCTFPFRWWIWGLKQVTAIENSIRYTNVF*Y  568

Query  123   RPIRADFTAQNALFDHHFGCRLIN*VG*K*RK  28
              PI   +T+Q  LF  ++ C LIN V  K RK
Sbjct  569   CPICIHYTSQYPLFYLNWRCGLINQVNSKERK  600



  Database: herai/heraiprot.txt
    Posted date:  Aug 19, 2014  9:57 AM
  Number of letters in database: 49,077,216
  Number of sequences in database:  93,476


Matrix: BLOSUM62
Gap Penalties: Existence: 11, Extension: 1
Neighboring words threshold: 12
```

Window for multiple hits: 40
