# Supplementary material for: De novo transcriptome assembly and analysis to identify potential gene targets for RNAi-mediated control of the tomato leafminer (Tuta absoluta)
Source: BMC Genomics. 2015 Aug 26;16(1):635. doi: 10.1186/s12864-015-1841-5 (PMC4550053; doi:10.1186/s12864-015-1841-5)
Supplement: Additional file 11: Table S10. — Primers used to amplify target-gene fragments flanked by attL1 and attL2 sequences (underlined) for each gene, with expected amplicon size in base pairs (bp) and annealing temperature adopted. (PDF 49 kb) [file 12864_2015_1841_MOESM11_ESM.pdf]

Table S10. Primers used to amplify target-gene fragments flanked by *attL1* and *attL2* sequences (underlined) for each gene, with expected amplicon size in base pairs (bp) and annealing temperature adopted.

ID Contig	Sequence	Size (bp)	Annealing temperature
77	GGGGCCAACTTTGTACAAAAAAGCAGGCTGGCAAGTGCTTTTTGAGACC<br>GGGGCCAACTTTGTACAAGAAAGCTGGGTAATTGTTGAAGGTGGCCAAG	439	50°C
592	GGGGCCAACTTTGTACAAAAAAGCAGGCTAGTTCGCGCTTTTGTATGCT<br>GGGGCCAACTTTGTACAAGAAAGCTGGGTGTTGGCCAGTTCCATCAGTT	474	55°C
1360	GGGGCCAACTTTGTACAAAAAAGCAGGCTGACCCTGTCTCGTGCTAACG<br>GGGGCCAACTTTGTACAAGAAAGCTGGGTATCACCGCAAATGAAACACA	498	60°C
2132	GGGGCCAACTTTGTACAAAAAAGCAGGCTTCTCATTGGTGGTGGTCTCA<br>GGGGCCAACTTTGTACAAGAAAGCTGGGTCTTCGAAGCAGCTGGAGTG	500	50°C
2308	GGGGCCAACTTTGTACAAAAAAGCAGGCTGTTCGTCTTCGTATTTCTTGG<br>GGGGCCAACTTTGTACAAGAAAGCTGGGTAACAACAAGCGCCTGCTGGG	253	50°C
2352	GGGGCCAACTTTGTACAAAAAAGCAGGCTGATCGGTTTTGCGTCGTATC<br>GGGGCCAACTTTGTACAAGAAAGCTGGGTTTACCTCCATCAGGGGTCAG	415	45°C
2594	GGGGCCAACTTTGTACAAAAAAGCAGGCTTTGCTTCTCGTTTCCTTCGT<br>GGGGCCAACTTTGTACAAGAAAGCTGGGTACGTTACCCTCGCATTCAAC	465	45°C
2779	GGGGCCAACTTTGTACAAAAAAGCAGGCTGTTCATCGGGTCGATTGATT<br>GGGGCCAACTTTGTACAAGAAAGCTGGGTGCATCGCAGCACTACTTTGA	467	n.a
4303	GGGGCCAACTTTGTACAAGAAAGCTGGGTCCACACCGATTCACACAATG<br>GGGGCCAACTTTGTACAAAAAAGCAGGCTTCAACTCCCCCATCAACCTA	366	50°C
4623	GGGGCCAACTTTGTACAAAAAAGCAGGCTGCCCTCCACTACCACAGCTA<br>GGGGCCAACTTTGTACAAGAAAGCTGGGTCAGCGCGATCTAGCAAAGTA	416	50°C

9589	GGGGCCAACTTTGTACAAAAAAGCAGGCTAATTCAGACGGGTCCACTTG<br>GGGGCCAACTTTGTACAAGAAAGCTGGGTACACGGTGATCTGTCACCAA	468	55°C
